# The oocyte-enriched metabolite serotonin alleviates cellular senescence and aging phenotypes in the mouse

**DOI:** 10.1038/s44318-026-00832-x

**Published:** 2026-06-16

**Authors:** Yuyan Xu, Ling Zhang, Xufeng Liao, Yuhang Fan, Rouyi Shao, Mengchen Wu, Li Zhang, Min Zhang, Gang Wang, Hailing Zhang, Enze Li, Qixin Wu, Jing Zhao, Jia Zhang, Wenjie Wang, Limeng Cai, Jinjie Zhong, Lidan Hu, Jingjing Wang, Jianhua Mao, Bing Yang, Jin Zhang

**Affiliations:** 1 Bone Marrow Transplantation Center of the First Affiliated Hospital, and Center for Stem Cell and Regenerative Medicine, Department of Basic Medical Sciences, and Liangzhu Laboratory, Zhejiang University School of Medicine, Hangzhou, China; 2 Center for Reproductive Medicine, The First Affiliated Hospital, Zhejiang University School of Medicine, Hangzhou, China; 3 National Clinical Research Center of Kidney Diseases, Jinling Hospital, Nanjing University School of Medicine, Nanjing, China; 4 Department of Gynecology and Obstetrics, The First Affiliated Hospital, Zhejiang University School of Medicine, Hangzhou, China; 5 Department of Nephrology, The Children’s Hospital, Zhejiang University School of Medicine, National Clinical Research Center for Child Health, Hangzhou, China; 6 MOE Key Laboratory for Biosystems Homeostasis & Protection and Innovation Center for Cell Signaling Network, Life Sciences Institute, Zhejiang University, Hangzhou, China; 7 Center of Gene and Cell Therapy and Genome Medicine of Zhejiang Province, Hangzhou, China

**Keywords:** Development, Metabolism, Stem Cells & Regenerative Medicine

## Abstract

Whether metabolites enriched at early developmental stages affect cellular and organismal aging remains unclear. In this study, we comprehensively profiled the metabolic landscape of mouse oocytes in comparison to cleavage-stage embryos. Our analysis revealed that oocytes display accumulation of reductive metabolites that diminish following fertilization. Notably, we identified serotonin (5-hydroxytryptamine, 5-HT) as an oocyte-enriched metabolite with protective roles in aging. The underlying mechanisms operate through dual pathways: (i) in a canonical pathway serotonin acts via its receptor 5HTR1B to modulate mitochondrial function, and (ii) in a non-canonical pathway serotonin promotes serotonylation of HSP90β, which effectively reduces endoplasmic reticulum stress. Overall, our study demonstrates that oocyte-enriched metabolites including serotonin can alleviate aging-related cellular and systemic phenotypes, suggesting new avenues for anti-aging strategies.

## Synopsis

Whether metabolites enriched at early developmental stages affect cellular and organismal aging remains unclear. This study uncovers a protective role for the oocyte-accumulated metabolite serotonin (5-hydroxytryptamine, 5-HT) in attenuating cellular senescence, reinforcing oxidative energy metabolism and proteostasis.

- Targeted metabolomics uncovers accumulation of reductive metabolites in mouse oocytes, in comparison to cleavage-stage embryos.
- Oocyte-derived metabolite extracts decrease aging-related senescence phenotypes in induced pluripotent stem cells, and mesenchymal-like stem cells (MSCs).
- Serotonin mitigates aging hallmarks in aged MII oocytes and MSCs, and alleviates pathological and natural aging phenotypes in vivo at the organismal level.
- Serotonin alleviates aging-related molecular features by enhancing mitochondrial function and decreasing ER stress via serotonylation of HSP90β.

## Abstract (web summary)

Early developmental metabolites protect from cellular senescence and aging-related energy metabolism alterations.

## Graphical abstract

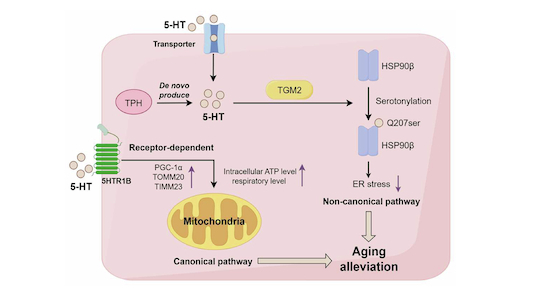

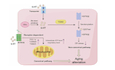

## Introduction

Aging is an inevitable process that affects all living organisms, characterized by multi-organ degeneration and a decline in physiological functions, ultimately leading to chronic diseases. Therefore, understanding the mechanisms underlying aging and developing interventions to prolong human lifespan is of great importance. Metabolism and aging are closely connected; for example, caloric restriction prevents age-related DNA methylation changes (Hahn et al, 2017), nicotinamide adenine dinucleotide (NAD^+^) production extends lifespan (Verdin, 2015), and metformin activates the AMPK pathway (Kulkarni et al, 2020). These examples underscore the diverse and vital roles of metabolic processes in aging. Consequently, there is a critical need to identify and explore potential metabolites that could contribute to lifespan extension.

Accumulating evidence suggests that the process of aging is linked to oxidative stress (Childs et al, 2015), and one of the strategies to alleviate aging is through intervention with small molecules including NMN (Miao et al, 2020; Mills et al, 2016), spermidine (Hofer et al, 2024; Zhang et al, 2023), metformin (Yang et al, 2024), taurine (Singh et al, 2023), and vitamin C (Li et al, 2016). The common feature of these metabolites is high reductive capacity. Previous studies demonstrated that embryos at earlier stages typically have reduced characteristics and gradually decrease with the development process (Zhao et al, 2021; Sharpley et al, 2021; Xu et al, 2025). However, these studies have focused on post-fertilization embryos, and whether oocytes have higher reductive capacity than subsequent cleavage embryos are unknown. What’s more, reprogramming factors, such as OSKM (OCT4, SOX2, KLF4, MYC) have been shown to significantly extend the lifespan of both prematurely and naturally aged mice (Sahu et al, 2024), reduce tissue aging (Chondronasiou et al, 2022), and reverse epigenetic clock markers, as well as metabolic and transcriptomic changes (Browder et al, 2022). Oocytes also have the ability to facilitate reprogramming with natural fertilization or somatic cell nuclear transfer (SCNT) while reprogramming factors like OCT4 are not highly expressed (Matoba and Zhang, 2018; Shinagawa et al, 2014), which further suggests there are other substances such as oocyte-specific metabolite to mediate the reprogramming and rejuvenation. All the above findings provide clues that oocytes may have unique metabolic characteristics and metabolites related to rejuvenation.

Here, we first depicted a comprehensive metabolomics landscape of oocyte to cleavage stage transition, demonstrating the reductive characteristics in oocyte compared with zygote or 2-cell embryo stage. Importantly, our findings discovered that serotonin or 5-hydroxytryptamine (5-HT), traditionally known as an antidepressant metabolite, was enriched in oocytes (compared with cleavage-stage) and could mitigate aging. We demonstrated that 5-HT contributes to alleviating cellular senescence by serotonylated heat shock protein HSP90β in addition to the classical way to regulate mitochondrial regeneration. These findings offer a promising new target for aging intervention.

## Results

### Unique reductive metabolic characteristics of oocytes

**Figure 1:**
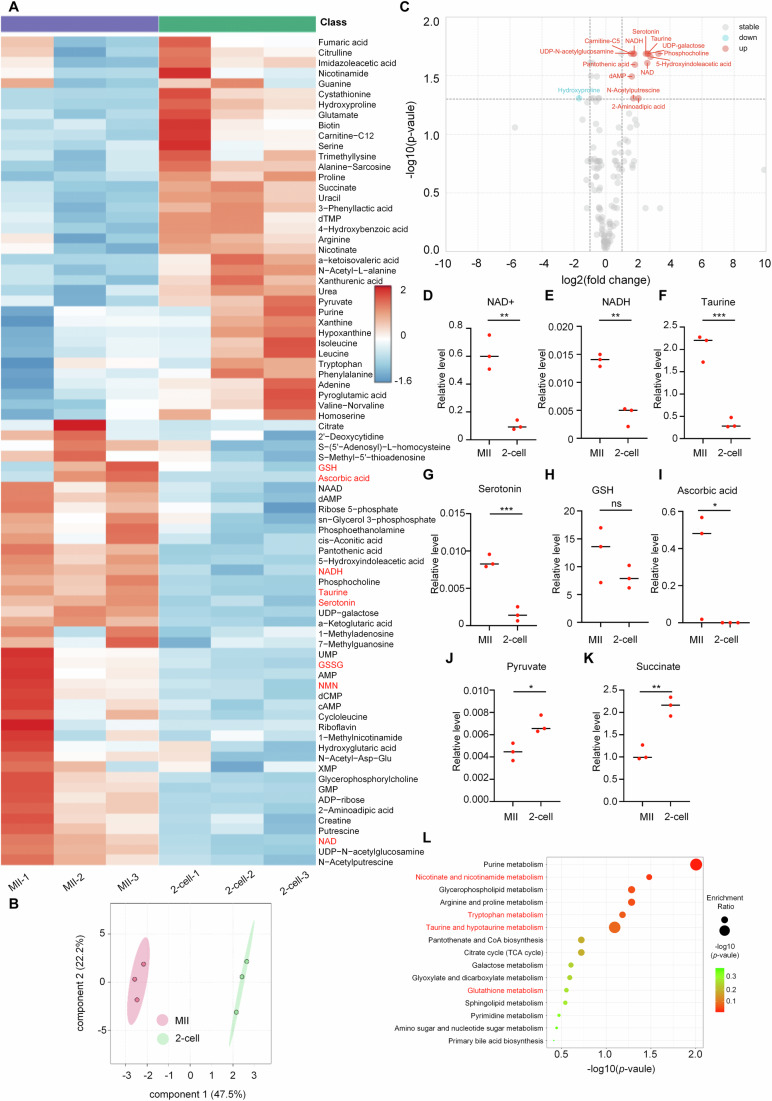
Metabolic landscape of oocytes and two-cell embryos. (**A**) Heatmap showing the relative abundance of metabolites in MII oocytes and 2-cell embryos. Peak areas for each detected metabolite were normalized against the total ion count of that sample. *n* =  3 biological replicates per stage. Each biological replicate was from 100 pooled embryos at either the MII or 2-cell stage. (**B**) A PCA plot of targeted metabolomics depicting the clustering of three biological replicates of oocytes and 2-cell embryos (*n* = 124 metabolites per sample). (**C**) Volcano plot analysis of oocytes and 2-cell embryos targeted metabolomics. Volcano plots were generated in MetaboAnalystR using a two-sample Student’s *t* test and fold change. Significant features were defined as |FC | ≥ 2 with adjusted *P* < 0.05. *n* = 3 biological repeats. (**D**–**K**) Comparison of the relative contents of metabolites in MII oocytes and 2-cell embryos. *P < 0.05, **P < 0.01, ***P < 0.001 according to the two-tailed unpaired *t* test. (**D**) *P* = 0.002; (**E**) *P* = 0.001; (**F**) *P* = 0.0008; (**G**) *P* = 0.0007; (**H**) *P* = 0.11; (**I**) *P* = 0.05; (**J**) *P* = 0.02; (**K**) *P* = 0.0024. *n* = 3 biological repeats. (**L**) KEGG pathway enrichment analysis of metabolites with higher content in MII oocytes compared to the 2-cell embryos. KEGG pathway enrichment was performed by over-representation analysis using a hypergeometric test (Fisher’s exact test), followed by Benjamini–Hochberg FDR correction (adjusted *P* values/*q* values). Data are presented as mean ± SD. See also Fig. EV1. Source data are available online for this figure.

**Figure EV1:**
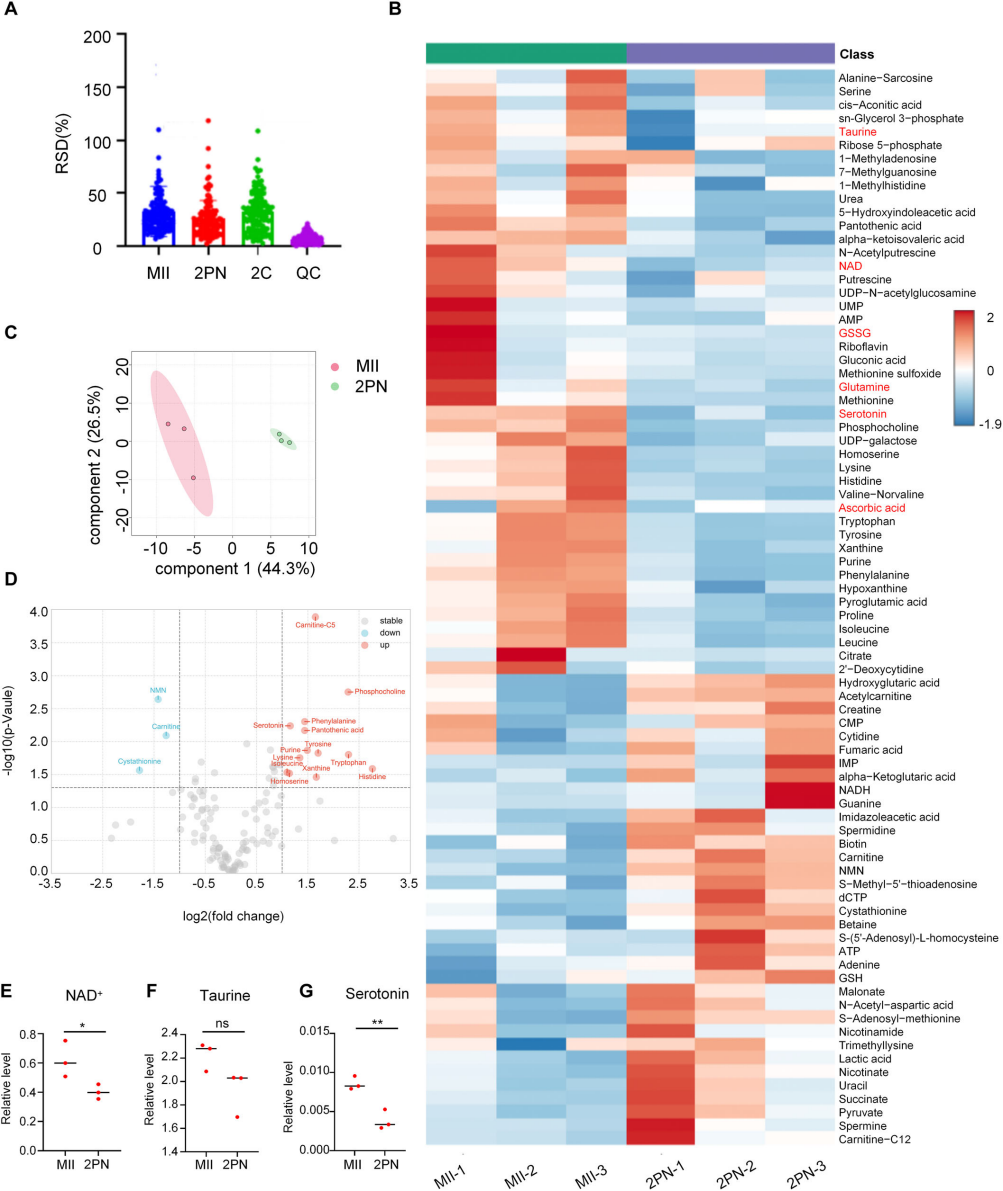
The metabolic landscape from oocytes to zygotes embryos stage. (**A**) QC chart of 10,000 FACS-sorted mouse cells. (**B**) Heatmap showing the relative abundance of metabolites between MII oocytes and 2PN zygote embryos. Peak areas for each detected metabolite were normalized against the total ion count of that sample. *n* =  3 biological replicates per stage. Each biological replicate was from 100 pooled embryos at either the MII or 2PN stage. (**C**) A PCA plot of MII oocytes and 2PN zygote embryos targeted metabolomics (*n* = 124 metabolites per sample). (**D**) Volcano plot analysis of MII oocytes and 2PN zygote embryos targeted metabolomics. Volcano plots were generated in MetaboAnalystR using a two-sample Student’s *t* test and fold change. Significant features were defined as |FC | ≥ 2 with a raw (unadjusted) *P *< 0.05. *n* = 3 biological repeats. (**E**–**G**) Comparison of the relative abundance of metabolites in MII and 2PN embryos. *P < 0.05, **P < 0.01, ***P < 0.001 according to the two-tailed unpaired *t* test. (**E**) *P* = 0.05; (**F**) *P* = 0.08; (**G**) *P* = 0.006. *n* = 3 biological repeats. Data are presented as mean ± SD. Source data are available online for this figure.

Metabolic composition of oocytes and early embryos was profiled using an established targeted metabolomics platform (Zhao et al, 2021). For each stage, one biological replicate consisted of a pooled sample of 100 oocytes or embryos, each with three biological replicates (Fig. 1A). Thus, the resulting metabolic signatures represent population-level trends across embryos rather than measurements of individual embryo variability. Due to the preciousness of embryo samples, 10,000 FACS-sorted mouse cells were used as QC to monitor instrument state stability during sample sequence collection. For both QC and embryo samples, over 99% of metabolites had a relative standard deviation (RSD) below 20%, indicative of excellent instrument stability and reproducibility (Fig. EV1A). Principal component analysis (PCA) showed that although inter-group variability existed, samples within each stage grouped closely together and were distinct from the others, which indicated that oocyte metabolite composition differed greatly from that of the 2C stage embryos (Fig. 1B). Comparative metabolomic analysis revealed substantial differences between oocytes and early embryos, with several key metabolites exhibiting significant variation in abundance. Notably, oocytes displayed substantially higher levels of NAD + , NADH, taurine, serotonin (5-HT), glutathione (GSH), and vitamin C (Fig. 1C–I), all of which are known to play crucial roles in cellular redox balance and antioxidant defense. Conversely, 2-cell embryos exhibited elevated concentrations of pyruvate and succinate, reflecting shifts in energy metabolism as development progresses (Fig. 1J,K). Subsequent metabolites set enrichment analysis identified several pathways predominantly enriched in oocytes relative to 2-cell embryos, including nicotinate and nicotinamide metabolism, tryptophan metabolism, taurine and hypotaurine metabolism, and glutathione metabolism (Fig. 1L). These metabolic distinctions persisted when comparing oocytes with zygotes, as evidenced by additional analysis (Fig. EV1C–G). Of particular significance, the metabolites preferentially abundant in oocytes are extensively involved in cellular redox homeostasis and possess well-documented antioxidant properties (Covarrubias et al, 2021; Froger et al, 2014; Yoo et al, 2017; Zia et al, 2021). These findings strongly suggest that oocytes maintain a more reductive intracellular environment compared to cleavage-stage embryos, lending support to the hypothesis that oocyte-derived metabolites may play crucial roles in cellular rejuvenation processes. Further investigations are warranted to elucidate the precise functions of these specific metabolites in embryonic development and to explore their potential applications in regenerative medicine strategies.

### Oocyte-derived metabolites decrease aging-related gene expression signatures and phenotypes in aged MSCs and oocytes

**Figure EV2:**
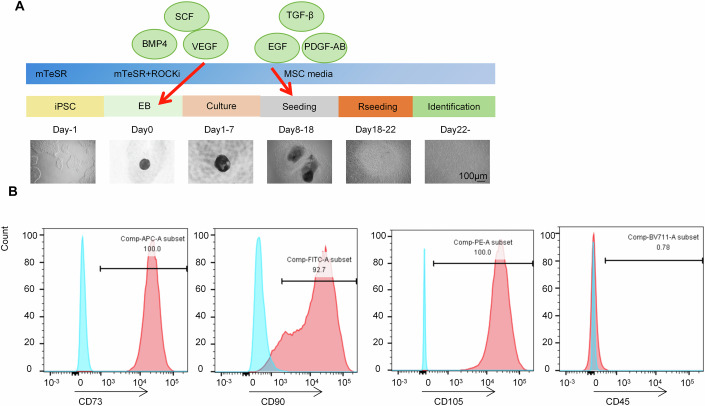
Process of differentiation of iPSC to MSC. (**A**) Flowchart of iPSC differentiation to MSCs(iPSC-derived mesenchymal stem/progenitor-like cells, CD73^+^/CD90^+^ /CD105^+^/CD45^−^). Scale bar, 100 μm. (**B**) Flow cytometry analysis of CD73, CD90, CD105 and CD45 for MSCs.

**Figure 2:**
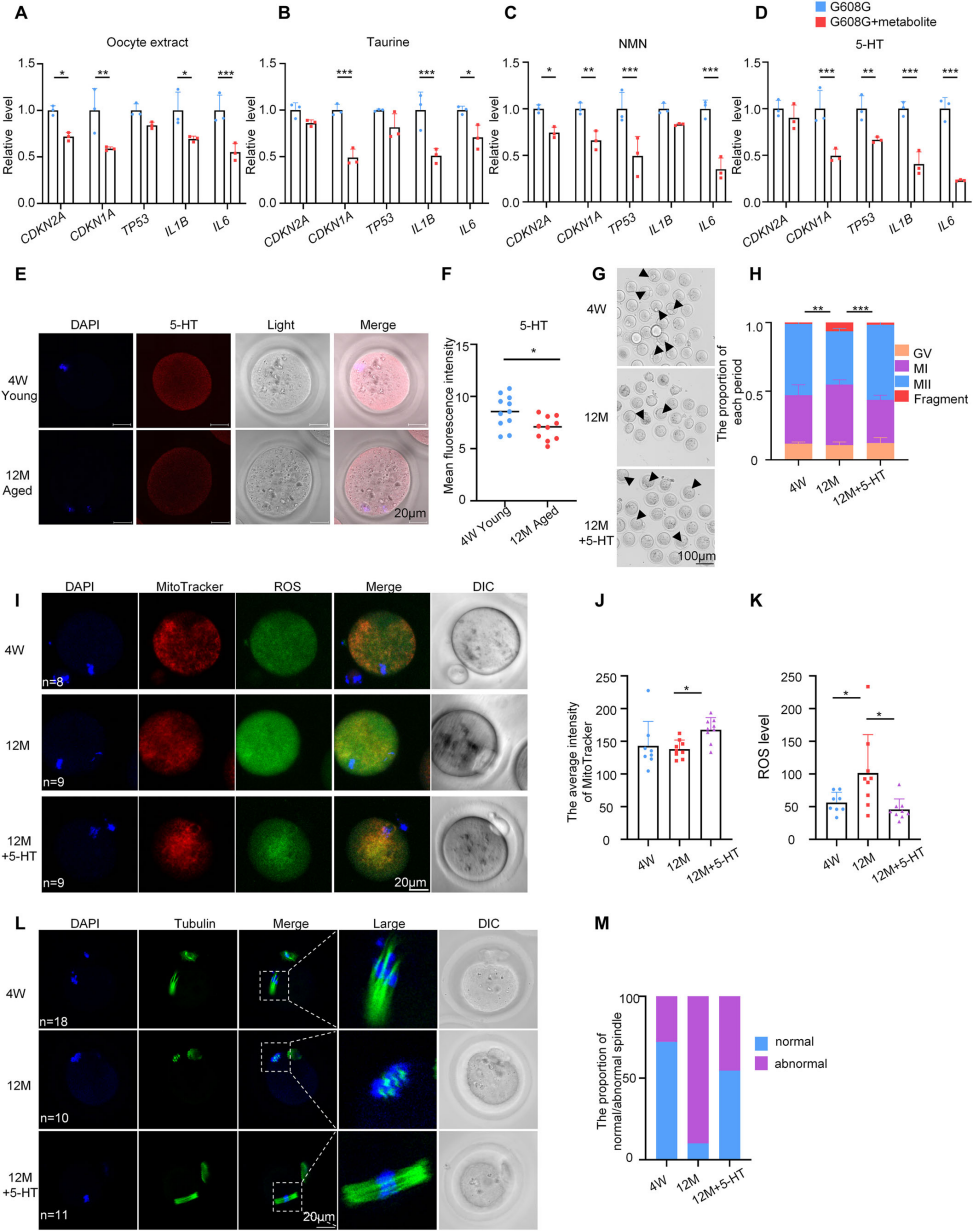
5-HT reverses the aging phenotype of aged oocyte. (**A**–**D**) RT-qPCR analysis of *CDKN2A*, *CDKN1A*, *TP53*, *IL1B*, and *IL6* mRNA expression in G608G MSC with or without metabolite treatment. *P < 0.05, **P < 0.01, ***P < 0.001 according to the two-way ANOVA test. *P* values from left to right: (**A**) *P* = 0.04, *P* = 0.002, *P* = 0.46, *P* = 0.03, *P* < 0.001; (**B**) *P* = 0.45, *P* < 0.001, *P* = 0.18, *P* < 0.001, *P* = 0.01; (**C**) *P* = 0.05, *P* = 0.006, *P* < 0.001, *P* = 0.34, *P* < 0.001; (**D**) *P* = 0.83, *P* < 0.001, *P* = 0.009, *P* < 0.001, *P* < 0.001. *n* = 3 biological repeats. (**E**, **F**) Immunofluorescence analysis (**E**) and quantification (**F**) of 5-HT of 4-week-old C57 and 12-month-old C57 MII oocytes. Scale bar, 20 μm. *P < 0.05, **P < 0.01, ***P < 0.001 according to the two-tailed unpaired *t* test. (**F**) *P* = 0.01. *n* = 10–11 biological repeats. (**G**) Bright-field imaging depicting the development status of GV from 4-week-old C57 mice and 12-month-old C57 mice treated with 5-HT or not. The arrowheads point to MII. Scale bar, 100 μm. (**H**) The proportion of GV, MI, and MII is analyzed in the form of overlapping graphs. *P < 0.05, **P < 0.01, ***P < 0.001 according to the two-way ANOVA test. *P* = 0.005, and *P* < 0.001. The analysis specifically focused on MII. *n* = 3 biological repeats. (**I**–**K**) Immunofluorescence analysis (**I**) of MitoTracker and DCFH-DA-based ROS levels in MII oocytes developed from GV of oocyte from 4-week-old and 12-month-old mice treated with or without 5-HT in vitro. Quantitative analysis of the fluorescence intensity of mitochondria (**J**) and the ROS level (**K**). Scale bar, 20 μm. *P < 0.05, **P < 0.01, ***P < 0.001 according to the one-way ANOVA test. (**J**) *P* = 0.69, and *P* = 0.02; (**K**) *P* = 0.04, and *P* = 0.01. *n* = 8–9 biological repeats. (**L**, **M**) Immunofluorescence analysis (**L**) of tubulin in oocytes stage (developed from GV in vitro) of 4-week-old and 12-month-old C57 mice treated with or without 5-HT in vitro. Quantitative analysis (**M**) of normal/abnormal spindle ratio of MII. Scale bar, 20 μm. Data are presented as mean ± SD. Source data are available online for this figure.

To investigate the potential role of oocyte-derived metabolites in counteracting cellular aging, we established an experimental model using mesenchymal-like stem cells (MSCs) differentiated from human induced pluripotent stem cell (iPSCs) (CD73^+^/CD90^+^/CD105^+^/CD45^−^; see Fig. EV2), hereafter referred to as iPSC-derived MSCs. Unless otherwise specified, “WT MSCs” and “G608G MSCs” refer to iPSC-derived MSC populations generated from healthy donors and Hutchinson–Gilford Progeria Syndrome (HGPS) patients, respectively. Treated MSCs with a comprehensive oocyte metabolite extract which was meticulously prepared from 100 MII embryos harvested from 4-week-old C57 mice. Following treatment, we observed a pronounced reduction in the expression of multiple senescence-associated factors, including the cell cycle inhibitors *CDKN2A/1A*, the tumor suppressor *TP53* (p53), and the pro-inflammatory cytokines *IL1B* and *IL6* (Fig. 2A). These molecular changes indicated a significant reversal of the senescent phenotype in the treated MSCs. To identify the potential bioactive components responsible for these anti-aging effects, we systematically evaluated individual oocyte-enriched metabolites. Treatment with taurine or nicotinamide mononucleotide (NMN) which is the precursor of NAD + , successfully recapitulated the anti-senescence effects observed with the complete extract (Fig. 2B,C). Most intriguingly, we found that 5-hydroxytryptamine (5-HT, serotonin), substantially decreased the expression of senescence-related markers in aged MSCs (Fig. 2D). This finding was particularly noteworthy as it identified a novel potential mediator of the anti-aging effects observed. While 5-HT has been extensively characterized for its antioxidant properties and its involvement in age-related cognitive decline (Kumar et al, 1998; Mattson et al, 2004; Morgan et al, 1987; Ramirez et al, 2014), its specific contribution to cellular senescence and potential rejuvenation pathways, distinct from its canonical functions as a neurotransmitter in the central nervous system, remains largely unexplored. Thus, this hypothesis will not only fill the critical knowledge gap about 5-HT but also provide a compelling foundation for exploring how oocyte-derived metabolites could be leveraged to develop next-generation anti-aging therapeutics.

To validate our hypothesis, we quantitatively assessed 5-HT (serotonin) content in metaphase II (MII) oocytes isolated from young (4-week-old) and aged (12-month-old) female mice. Immunofluorescence analysis revealed a significant reduction in 5-HT levels within aged MII oocytes compared to their younger counterparts (Fig. 2E,F). Subsequently, we investigated whether this age-related 5-HT deficiency functionally contributes to compromised oocyte quality. Notably, exogenous 5-HT supplementation significantly restored the maturation capacity of aged oocytes, as evidenced by enhanced first polar body extrusion rates (Fig. 2G,H). Furthermore, our comprehensive evaluation of oocyte quality parameters demonstrated that 5-HT treatment effectively ameliorated age-associated cellular dysfunctions, significantly improving mitochondrial activity and reducing reactive oxygen species (ROS) accumulation in aged oocytes (Fig. 2I–K). Morphological assessment also revealed that 5-HT supplementation restored normal spindle formation in aged oocytes (Fig. 2L,M). Collectively, these findings provide compelling evidence that 5-HT plays a critical role in counteracting age-related deterioration of oocyte quality through multiple cellular mechanisms, potentially offering insights into therapeutic strategies for age-associated reproductive decline.

### 5-HT mitigates the aging hallmarks at multiple cellular levels in both the pathological and natural aging contexts

**Figure 3:**
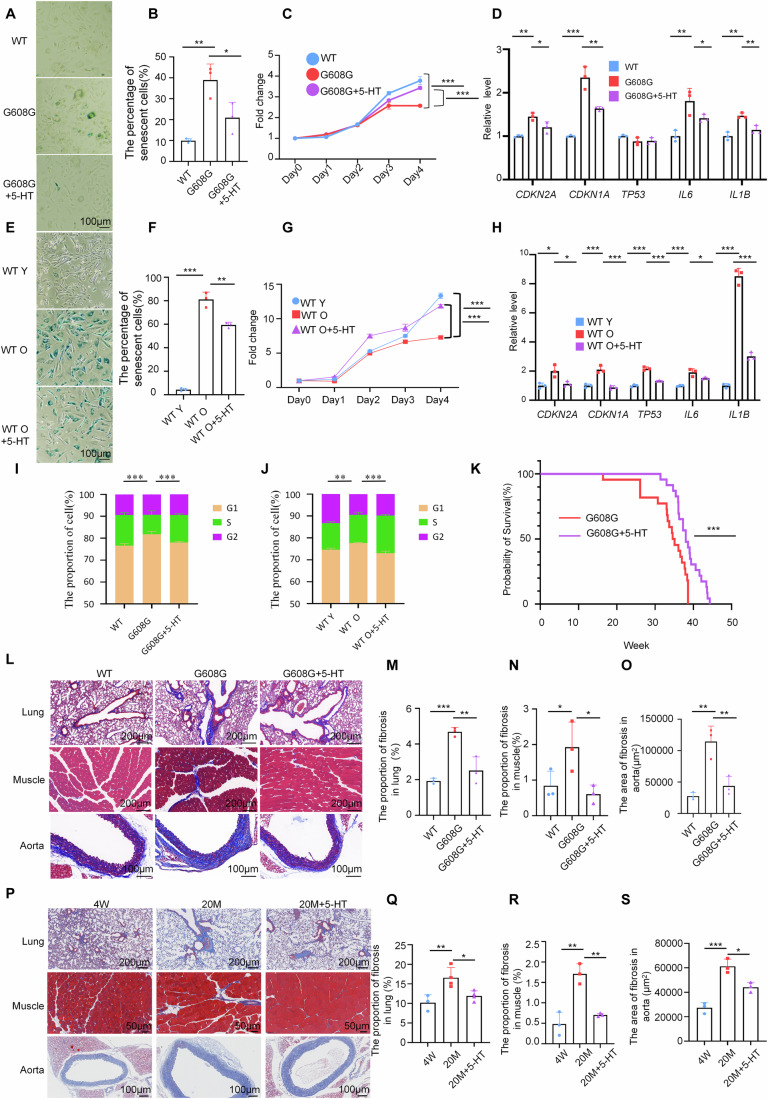
5-HT block the senescence phenotype of MSCs. (**A**, **B**) SA-β-Gal staining (**A**) and quantification (**B**) of WT MSC and G608G MSC with or without 5-HT treatment. *P < 0.05, **P < 0.01, ***P < 0.001 according to the one-way ANOVA test. (**B**) *P* = 0.001, and *P* = 0.01. *n* = 3 biological repeats. Scale bar, 100 μm. (**C**) CCK-8 evaluation of proliferation of WT MSC and G608G MSC with or without 5-HT treatment. *P < 0.05, **P < 0.01, ***P < 0.001 according to the two-way ANOVA test. WT vs G608G: *P* < 0.001; G608G vs G608G + 5-HT: *P* < 0.001. *n* = 3 biological repeats. (**D**) RT-qPCR analysis of *CDKN2A*, *CDKN1A*, *TP53*, *IL6*, and *IL1B* mRNA expression in WT MSC and G608G MSC with or without 5-HT treatment. *P < 0.05, **P < 0.01, ***P < 0.001 according to the one-way ANOVA test. *P* values from left to right: *P* = 0.002, *P* = 0.03; *P* < 0.001, *P* = 0.003; *P* = 0.16, *P* = 0.96; *P* = 0.002, *P* = 0.05; *P* = 0.002, *P* = 0.009. *n* = 3 biological repeats. (**E**, **F**) SA-β-Gal staining (**E**) and quantification (**F**) of young generations of WT MSC(WT Y), old generations of WT MSC(WT O), and old generations of WT MSC treated with 5-HT(WT O + 5-HT, 5 μM). *P < 0.05, **P < 0.01, ***P < 0.001 according to the one-way ANOVA test. (**F**) *P* < 0.001, and *P* = 0.001. *n* = 3 biological repeats. Scale bar, 100 μm. (**G**) CCK-8 evaluation of the proliferation of WT Y, WT O, and WT O + 5-HT. *P < 0.05, **P < 0.01, ***P < 0.001 according to the two-way ANOVA test. WT Y vs WT O: *P* < 0.001; WT O vs WT O + 5-HT: *P* < 0.001. *n* = 3 biological repeats. (**H**) RT-qPCR analysis of *CDKN2A*, *CDKN1A*, *TP53*, *IL6*, and *IL1B* mRNA expression of WT Y, WT O, and WT O + 5-HT. *P < 0.05, **P < 0.01, ***P < 0.001 according to the one-way ANOVA test. *P* values from left to right: *P* = 0.01, *P* = 0.02; *P* < 0.001, *P* < 0.001; *P* < 0.001, *P* < 0.001; *P* < 0.001, *P* = 0.04; *P* < 0.001, *P* < 0.001. *n* = 3 biological repeats. (**I**, **J**) Cell cycle distribution patterns of WT MSCs and G608G MSCs of identical passage number (**I**) and young and aged WT MSCs (**J**). *P < 0.05, **P < 0.01, ***P < 0.001 according to the two-way ANOVA test. (**I**) *P* < 0.001, and *P *< 0.001; (**J**) *P* = 0.002, and *P* < 0.001. *n* = 3 biological repeats. (**K**) Meier survival plot of G608G mice treated with 0.9% NaCl (*n* = 22) or 5-HT (*n* = 23). The Log-rank (Mantel–Cox) test was used for statistical analysis. *P* = 0.0007; *P < 0.05, **P < 0.01, ***P < 0.001. (**L**–**O**) Masson staining (**L**) and quantification of fibrosis area (**M**–**O**) of lung, muscle, and aorta in WT and G608G mice treated with 5-HT or not. *P < 0.05, **P < 0.01, ***P < 0.001 according to the one-way ANOVA test. (**M**) *P* < 0.001, and *P* = 0.003; (**N**) *P* = 0.04, and *P* = 0.02; (**O**) *P* = 0.002, and *P* = 0.006. *n* = 3 biological repeats. Scale bar, 200 and 100 μm. (**P**–**S**) Masson staining (**P**) and quantification of fibrosis area (**Q**–**S**) of lung, muscle, and aorta in 4-week-old and 20-month-old mice treated with 5-HT or not (from 12-month-old to 20-month-old). *P < 0.05, **P < 0.01, ***P < 0.001 according to the one-way ANOVA test. (**Q**) *P* = 0.009, and *P* = 0.03; (**R**) *P* = 0.001, and *P* = 0.003; (**S**) *P* < 0.001, and *P* = 0.01. *n* = 3–4 biological repeats. Scale bar, 200, 50 and 100 μm. Data are presented as mean ± SD. See also Fig. EV3. Source data are available online for this figure.

**Figure EV3:**
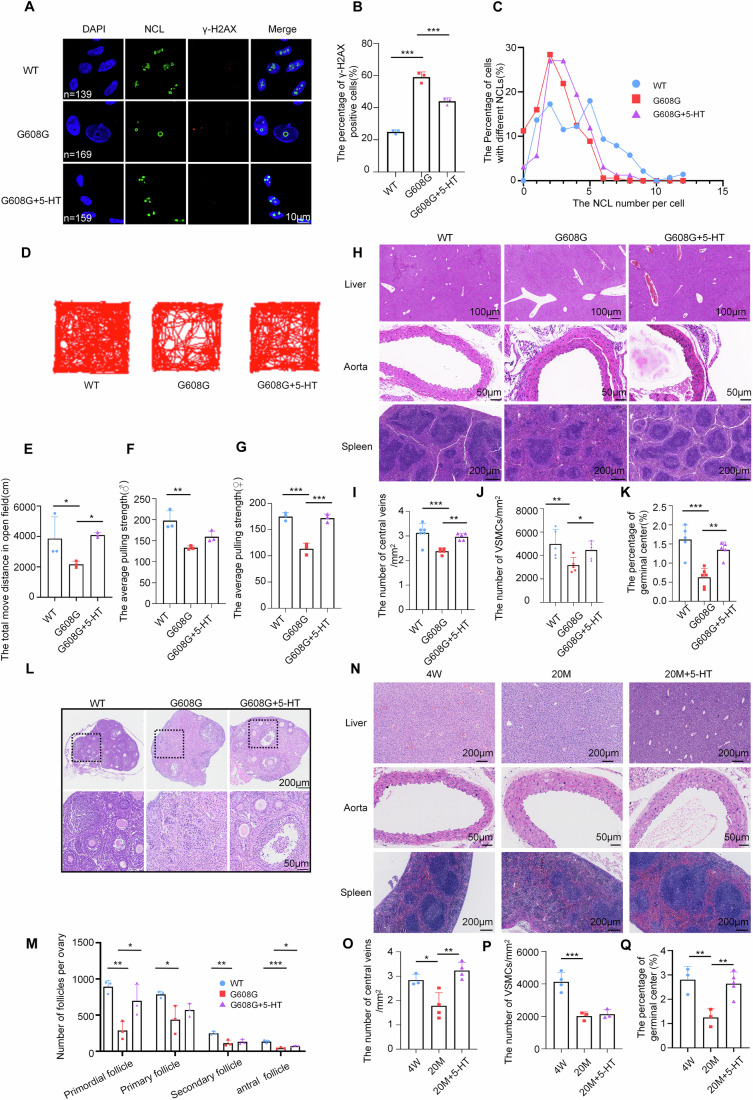
5-HT affects classic hallmarks associated with aging. (**A**–**C**) Immunofluorescence analysis (**A**) and quantification of γ-H2AX (**B**) and NCL (**C**) in WT MSC and G608G MSC with or without 5-HT treatment. *P < 0.05, **P < 0.01, ***P < 0.001 according to the one-way ANOVA test. (**B**) *P* < 0.001, and *P* < 0.001. *n* = 3 biological repeats. Scale bar, 10 μm. (**D**, **E**) Open-field trajectory diagram (**D**) of 5-month-old WT and G608G mice treated with 5-HT or not (from 1-month-old to 5-month-old). Quantitative analysis of the total distance covered (**E**). *P < 0.05, **P < 0.01, ***P < 0.001 according to the one-way ANOVA test. (**E**) *P *= 0.05, and *P* = 0.03. *n* = 3 biological repeats. (**F**, **G**) Quantitative analysis of the average pulling strength of male (**F**) and female (**G**) 5-month-old WT and G608G mice treated with 5-HT or not, *P < 0.05, **P < 0.01, ***P < 0.001 according to the one-way ANOVA test. (**F**) *P* = 0.003, and *P* = 0.09; (**G**) *P* < 0.001, and *P* < 0.001. *n* = 3 biological repeats. (**H**–**K**) Histological analysis (HE) (**H**) of the liver, spleen, and aorta of 5-month-old WT and G608G mice treated with 5-HT or not; Quantitative analysis of the number of central veins per unit area (mm^2^) in the liver (**I**), the average VSMC number per unit area (mm^2^) in the aorta (**J**), and the percentage of germinal center area in the spleen (**K**). *P < 0.05, **P < 0.01, ***P < 0.001 according to the one-way ANOVA test. (**I**) *P* < 0.001, and *P* = 0.006; (**J**) *P* = 0.008, and *P* = 0.05; (**K**) *P* < 0.001, and *P* = 0.003. *n* = 5–6 biological repeats. Scale bar, 100, 200 and 50 μm. (**L**) HE section of the ovary of 4-month-old WT and G608G mice treated with 5-HT or not. Scale bar, 200 and 50 μm. (**M**) Quantitative analysis of the number of follicles in each layer of ovaries of G608G mice treated with 5-HT or not. *P < 0.05, **P < 0.01, ***P < 0.001 according to the one-way ANOVA test. *P* values from left to right: *P* = 0.008, and *P* = 0.04; *P* = 0.03, and *P* = 0.44; *P* = 0.007, and *P* = 0.8; *P* < 0.001, and *P* = 0.04. *n* = 3 biological repeats. (**N**–**Q**) Histological analysis (HE) (**N**) of the liver, spleen, and aorta of 4-week-old and 20-month-old C57 mice treated with 5-HT or not (from 12-month-old to 20-month-old); Quantitative analysis of the number of central veins per unit area (mm^2^) in the liver (**O**), the average VSMC number per unit area (mm^2^) in the aorta (**P**), and the percentage of germinal center area in the spleen (**Q**). *P < 0.05, **P < 0.01, ***P < 0.001 according to the one-way ANOVA test. (**O**) *P* = 0.02, and *P* = 0.003; (**P**) *P* < 0.001, and *P* = 0.94; (**Q**) *P* = 0.009, and *P* = 0.009. *n* = 3–4 biological repeats. Scale bar, 200 and 50 μm. Data are presented as mean ± SD. Source data are available online for this figure.

To comprehensively study the role of 5-HT in contributing to cellular senescence, we conducted ex vivo and in vivo experiments to explore whether 5-HT intervention systematically influences aging in both pathological and natural aging contexts. By differentiating iPSCs derived from classic HGPS patients (heterozygous LMNA c. G608GC>T mutation) and healthy donors into MSCs (Fig. EV2) (Wu et al, 2018), we established two cell models of pathological aging and replicative aging. The former used WT MSCs of the same generation as the control, while using G608G MSCs as the experimental group, and the latter used WT MSCs of the younger generation (WT Y, P3-P5) as the control while using old WT MSCs (WT O, P12-P15) as the experimental group. The established MSCs recapitulated the aging phenotypes (Liu et al, 2011; Wu et al, 2018), such as the increased β-galactosidase-positive cells, reduced cell proliferation rate, and upregulated senescence-related gene expression. Our experiments demonstrated that 5-HT treatment at a 5 μM concentration effectively attenuated cellular senescence markers across multiple aging models. In both pathological aging and replicative aging models, 5-HT administration significantly decreased β-galactosidase expression while enhancing MSC proliferation capacity (Fig. 3A–C,E–G). Consistent with these phenotypic changes, we observed reduced mRNA expression of senescence marker genes in 5-HT-treated groups compared to controls (Fig. 3D,H). Further investigation revealed distinct cell cycle distribution patterns between WT MSCs and G608G MSCs of identical passage number, as well as between young and aged WT MSCs. Notably, these cell cycle disparities were substantially mitigated following 72 h of 5-HT treatment, suggesting that 5-HT intervention helps restore normal cell cycle progression in senescent cells (Fig. 3I,J). Moreover, the DNA damage marker γH2AX was significantly upregulated in G608G MSCs and reduced after supplementation of 5-HT (Fig. EV3A,B). This aligns with the observed decrease in nucleoli (NCL) number associated with senescence, which was also reversed by 5-HT supplementation (Fig. EV3C). Collectively, these findings suggest that 5-HT mitigates cellular senescence phenotypes.

### 5-HT alleviates pathological and natural aging phenotypes in vivo at the organismal level

**Figure EV4:**
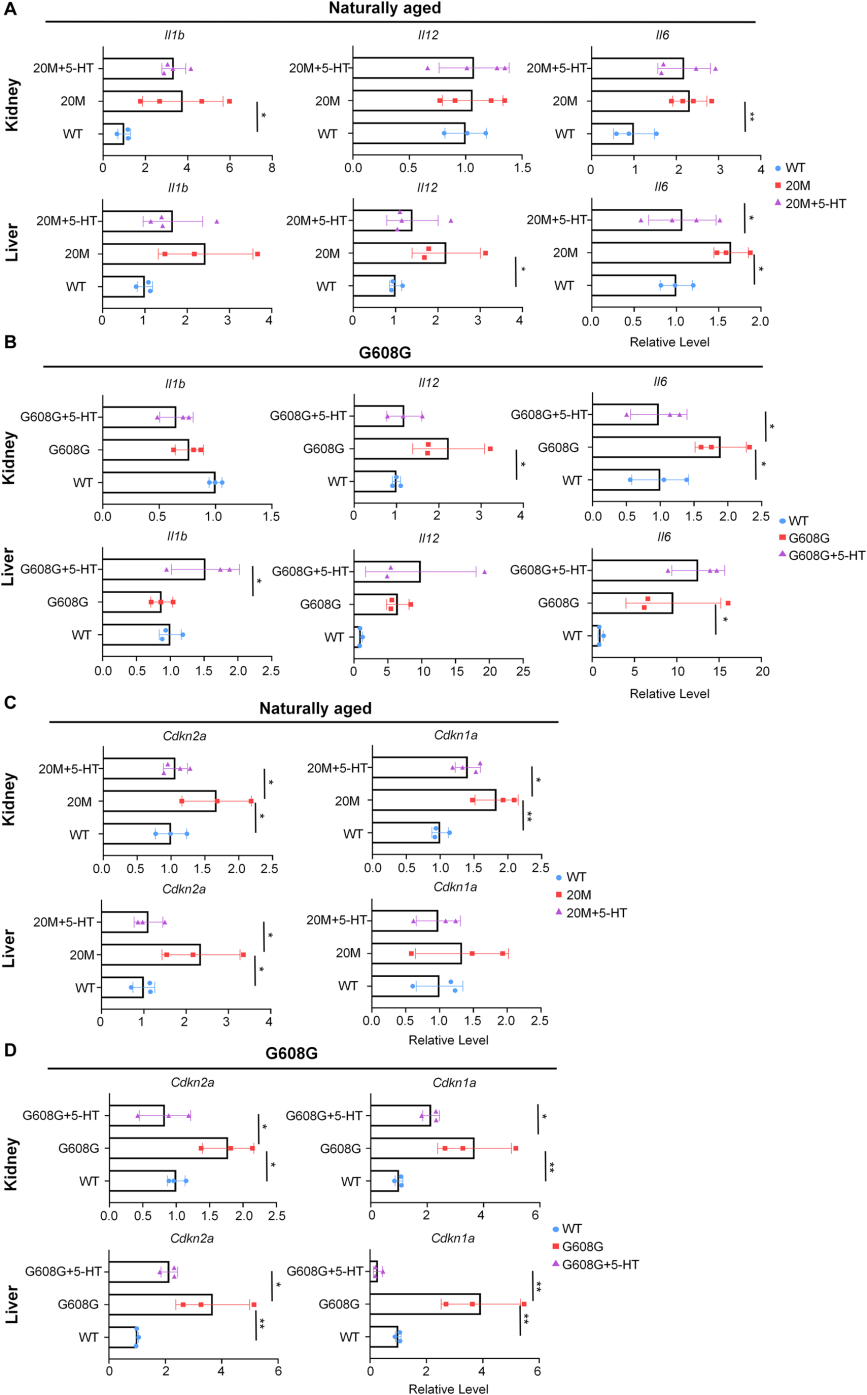
5-HT’s lifespan-extending effects in G608G mice. (**A**, **B**) RT-qPCR analysis of *Il1b*, *Il12* and *Il6* mRNA expression of liver and kidney in naturally aged and G608G mice treated with 5-HT or not. *P < 0.05, **P < 0.01, ***P < 0.001 according to the one-way ANOVA test. *P* values from left to right: (**A**) Kidney: *P *= 0.02, and *P* = 0.64; *P* = 0.77, and *P* = 0.95; *P* = 0.01, and *P* = 0.72. Liver: *P* = 0.12, and *P* = 0.43; *P* = 0.04, and *P* = 0.12; *P* = 0.03, and *P* = 0.04. (**B**) Kidney: *P* = 0.05, and *P* = 0.28; *P *= 0.03, and *P* = 0.06; *P* = 0.04, and *P* = 0.03. Liver: *P* = 0.65, and *P* = 0.05; *P* = 0.21, and *P* = 0.42; *P* = 0.03, and *P* = 0.37. *n* = 3–4 biological repeats. (**C**, **D**) RT-qPCR analysis of *Cdkn2a* and *Cdkn1a* mRNA expression of liver and kidney in naturally aged and G608G mice treated with 5-HT or not. *P < 0.05, **P < 0.01, ***P < 0.001 according to the one-way ANOVA test. (**C**) Kidney: *P* = 0.04, and *P* = 0.04; *P* = 0.002, and *P* = 0.04. Liver: *P* = 0.03, and *P* = 0.04; *P* = 0.43, and *P* = 0.41. (**D**) Kidney: *P* = 0.03, and *P* = 0.01; *P* = 0.006, and *P* = 0.05. Liver: *P* = 0.005, and *P* = 0.05; *P* = 0.005, and *P* = 0.002. *n* = 3–4 biological repeats. Data are presented as mean ± SD. Source data are available online for this figure.

In vivo assessment was conducted using G608G premature aging mice(homozygous), which carry a G608G mutation at the *LMNA* gene (referred to as G608G) (Varga et al, 2006), and wild-type mice. G608G mice were orally administered with 5-HT in water (0.375 mg/kg/day) (start feeding at 1 month old). The mice treated had significantly higher survival rates, and the maximum lifespan was also prolonged compared with the control group (Fig. 3K). Furthermore, assessments of 5-month-old mice revealed enhanced physical capabilities in the treated cohort, with tensile force testing demonstrating greater strength and open field testing showing increased total movement distance relative to controls (Fig. EV3D–G). Anatomical evidence demonstrated that 5-HT ameliorated various aging phenotypes at the tissue level in the G608G mice, including a reduced number of central hepatic veins and aortic vascular smooth muscles, loss of germinal center area in the spleen (Fig. EV3H–K), as well as an increased degree of lung, muscle and aortic fibrosis (Fig. 3L–O). Notably, 5-HT demonstrated potential to reverse premature ovarian failure. Whole-ovary biopsies revealed that 5-HT treatment successfully restored follicle numbers in prematurely aging G608G mice (Fig. EV3L,M), suggesting meaningful reproductive tissue preservation. To comprehensively evaluate the multifaceted nature of biological aging, we extended our investigation to naturally aged mice, providing a more physiologically relevant context for our findings. Wild-type C57BL/6J mice were administered with 5-hydroxytryptamine (5-HT) orally (0.375 mg/kg/day) from 12 months of age (equivalent to middle age in humans) through 20 months (corresponding to elderly status). Longitudinal analysis revealed that 5-HT-treated aged mice exhibited significantly more youthful tissue architecture and functionality compared to age-matched controls across multiple organ systems (Figs. 3P–S and  EV3N–Q). While previous literature has established 5-HT as a potent modulator of immune function, our in vivo transcriptome level analyses revealed tissue-specific and inconsistent alterations in pro-inflammatory cytokine expression (*Il1b, Il6, Il12*) across hepatic and kidney tissues following chronic 5-HT administration (Fig. EV4A,B). This heterogeneous inflammatory response aligns with 5-HT’s well-documented bidirectional regulatory capacity in inflammation (Ahern, 2011; Flanagan and Nichols, 2018; Herr et al, 2017), where context-dependent effects have been previously reported. In striking contrast, we observed robust and consistent downregulation of senescence-associated genes (*Cdkn2a* and *Cdkn1a*) in both hepatic and kidney tissues, suggesting that 5-HT’s lifespan-extending effects in vivo are unlikely to be predominantly mediated through immunomodulatory pathways (Fig. EV4C,D). These data collectively provide compelling evidence that 5-HT administration effectively attenuates aging-associated phenotypes in vivo, pointing to a more comprehensive physiological role for serotonin in counteracting aging at the organism level.

### 5-HT alleviates aging-related molecular features by regulating mitochondrial function

**Figure 4:**
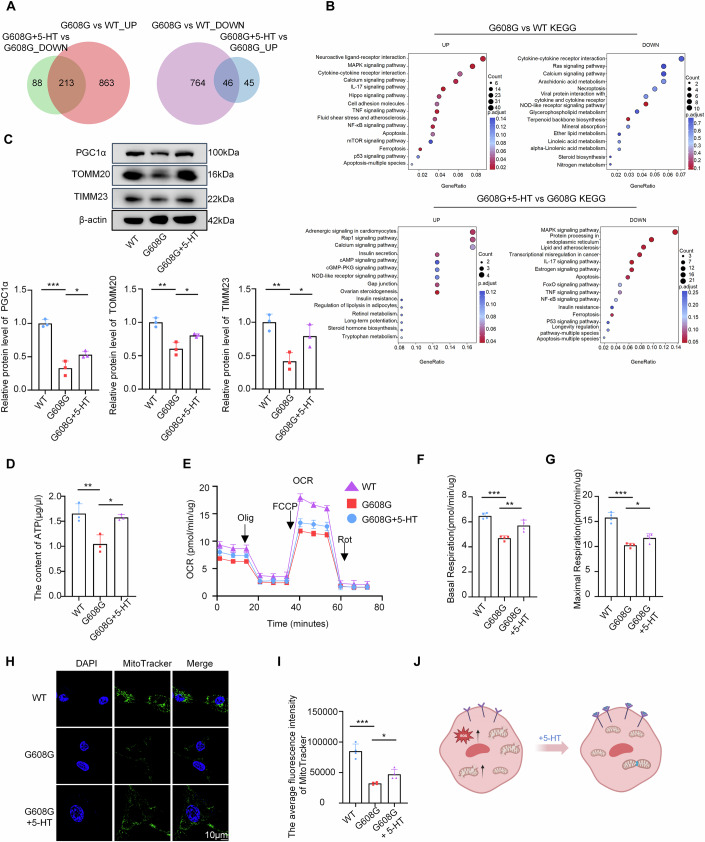
5-HT regulation the function of mitochondria. (**A**) Venn diagram showing the overlap of the differentially expressed genes (*P* value ≤ 0.05, |log2Foldchange | ≥1) between WT MSC and G608G MSC and between G608G MSC and G608G MSC treated with 5-HT, Red: G608G vs WT UP, Green: G608G + 5-HT vs G608G DOWN, Blue: G608G + 5-HT vs G608G UP, Purple: G608G vs WT DOWN. (**B**) Genome-wide KEGG pathways analysis of upregulated and downregulated genes between G608G MSC and WT MSC and between G608G MSC and G608G + 5-HT. KEGG pathway enrichment was performed by over-representation analysis using a hypergeometric test (Fisher’s exact test), followed by Benjamini–Hochberg FDR correction (adjusted *P* values/*q* values). (**C**) Western blots of PGC1α, TOMM20, and TIMM23 in WT MSC, G608G MSC, and G608G MSC treated with 5-HT. Lower: Quantification of the protein levels relative to β-actin. *P < 0.05, **P < 0.01, ***P < 0.001 according to the one-way ANOVA test. PGC1α: *P* < 0.001, and *P *= 0.04; TOMM20: *P* = 0.001, and *P* = 0.03; TIMM23: *P* = 0.006, and *P* = 0.04. *n* = 3 biological repeats. (**D**) The ATP levels of WT MSC, G608 MSC, and G608G were treated with 5-HT. *P < 0.05, **P < 0.01, ***P < 0.001 according to the one-way ANOVA test. *P* = 0.008, and *P* = 0.01. *n* = 3 biological repeats. (**E**–**G**) A representative seahorse plot (**E**) of WT MSC, G608G MSC, and 5-HT-treated G608G MSC, with measurements of OCR (normalized to protein), and following treatment of cells with oligomycin (Olig), fluorcarbonylcyanide phenylhydrazone (FCCP), and rotenone (Rot) as indicated. Quantitative analysis of the effects of 5-HT on basal respiration (**F**) and maximal respiration (**G**) (compiled results from *n* = 4). *P < 0.05, **P < 0.01, ***P < 0.001 according to the one-way ANOVA test. (**F**) *P* < 0.001, and *P* = 0.003; (**G**) *P* < 0.001, and *P* = 0.03. *n* = 4 biological repeats. (**H**, **I**) Immunofluorescence experiment of MitoTracker (**H**) and fluorescence intensity quantification in analysis (**I**) of WT MSC, G608G MSC, and 5-HT-treated G608G MSC. *P < 0.05, **P < 0.01, ***P < 0.001 according to the one-way ANOVA test. (**I**) *P* < 0.001 and *P* = 0.03. *n* = 4 biological repeats. Scale bar, 10 μm. (**J**) Schematics showing that 5-HT promote mitochondrial regeneration and regulate mitochondrial function. Data are presented as mean ± SD. See also Fig. EV5. Source data are available online for this figure.

**Figure EV5:**
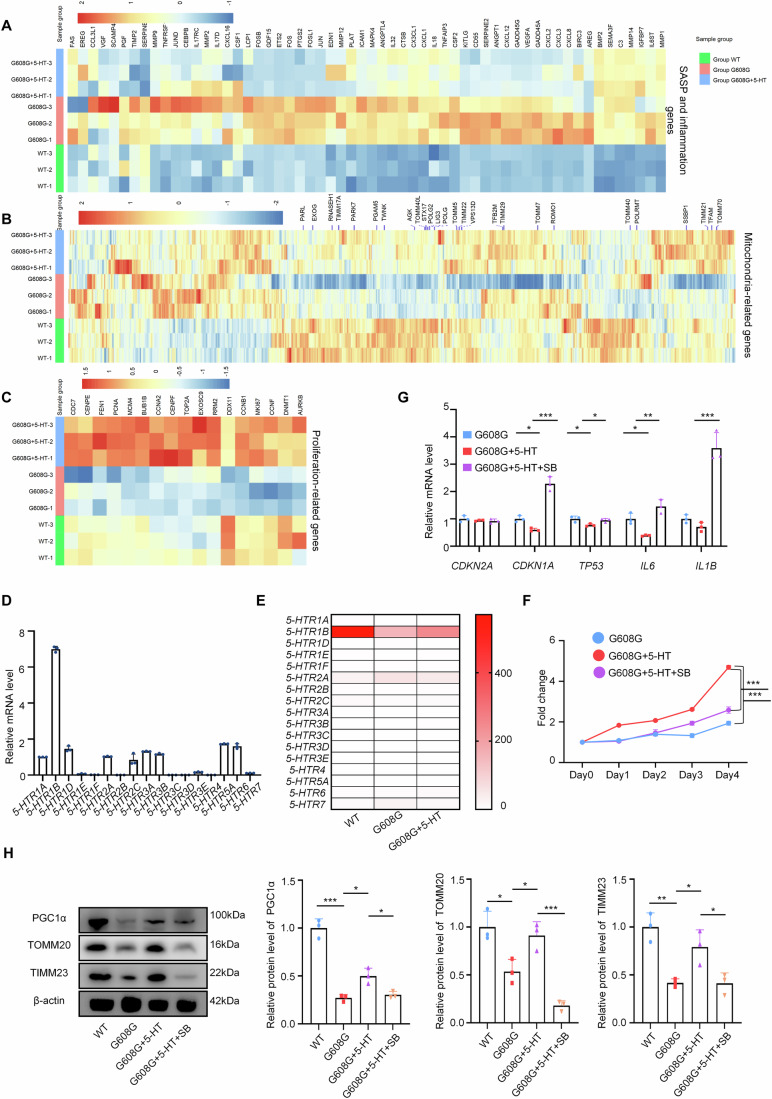
Effects of 5-HT supplementation on transcriptome of G608G MSC. (**A**–**C**) Heatmaps of the genes related to inflammation (**A**), mitochondria (**B**), and proliferation (**C**) of WT MSC and G608G MSC treated with 5-HT or not. (**D**) RT-qPCR analysis of 5-HT receptor subtypes in MSC. *n* = 3 biological repeats. (**E**) Heatmap of expression levels of all 5-HT receptors in MSC from RNA sequencing results mentioned above. (**F**) CCK-8 evaluation of proliferation of G608G MSC treated with 5-HT or 5-HT + SB (SB: SB-224289 hydrochloride). *P < 0.05, **P < 0.01, ***P < 0.001 according to the two-way ANOVA test. G608G vs G608G + 5-HT: *P* < 0.001. G608G + 5-HT vs G608G + 5-HT + SB: *P* < 0.001. *n* = 3 biological repeats. (**G**) RT-qPCR analysis of *CDKN2A*, *CDKN1A*, *TP53*, *IL6* and *IL1B* mRNA expression in G608G MSC treated with 5-HT or 5-HT + SB. *P < 0.05, **P < 0.01, ***P < 0.001 according to the one-way ANOVA test. *P* values from left to right: *P* = 0.69, and *P* = 0.93; *P* = 0.02, and *P* < 0.001; *P* = 0.01, and *P* = 0.03; *P* = 0.02, and *P* = 0.001; *P* = 0.42, and *P* < 0.001. *n* = 3 biological repeats. (**H**) Western blots of PGC1α, TOMM20, and TIMM23 protein levels in G608G MSC treated with 5-HT or 5-HT + SB. Right: Quantification of the protein levels relative to β-actin. *P < 0.05, **P < 0.01, ***P < 0.001 according to the one-way ANOVA test. PGC1α: *P* < 0.001, *P* = 0.01, and *P *= 0.03; TOMM20: *P* = 0.01, *P* = 0.03, and *P* < 0.001; TIMM23: *P* = 0.003, *P* = 0.03, and *P* = 0.03. *n* = 3 biological repeats. Data are presented as mean ± SD. Source data are available online for this figure.

To further elucidate the mechanisms by which 5-HT modulates cellular senescence, we performed transcriptomic analysis. Venn diagrams were constructed to visualize the overlap of differentially expressed genes (DEGs) between WT MSCs and G608G MSCs, as well as between G608G MSCs and those treated with 5-HT (Fig. 4A). Notably, G608G MSCs exhibited elevated gene expression in the IL17, NF-κB, and TP53 signaling pathways compared to WT MSCs, which was effectively normalized by 5-HT treatment (Fig. 4B). Heatmap analysis revealed a significant upregulation of factors associated with the senescence-associated secretory phenotype (SASP) in G608G MSCs, which was mitigated by 5-HT (Fig. EV5A). Conversely, genes linked to cell proliferation and mitochondrial function were downregulated in G608G MSCs, and their expression was restored upon 5-HT administration (Fig. EV5B,C).

We hypothesized that the aging-alleviating effect of 5-HT might be related to its canonical function in regulating mitochondria biogenesis, as 5-HT exposure significantly increased the protein levels of PGC-1α, TOMM20, and TIMM23 (Fig. 4C). This was accompanied by increased intracellular ATP levels (Fig. 4D) and enhanced respiratory capacity (Fig. 4E–G). Moreover, 5-HT treatment restored the punctate mitochondrial morphology and mitotracker intensity in G608G MSCs (Fig. 4H,I). Next, we examined whether 5-HT’s effects on mitochondria were receptor-dependent, like its mechanism in the nervous system. Analysis of 5-HT receptor expression revealed that 5-HTR1B was the most highly expressed subtype (Fig. EV5D,E). Using SB-224289 hydrochloride, a selective 5-HTR1B antagonist, we observed partial reversal of 5-HT’s effects, including its ability to promote cell proliferation (Fig. EV5F) and reduce SASP (Fig. EV5G), along with complete reversal of its enhancement of mitochondrial biogenesis (Fig. EV5H). Collectively, the above data suggests that 5-HT can promote mitochondrial regeneration in a 5-HTR1B-dependent manner, thereby affecting cell senescence (Fig. 4J).

### 5-HT promotes serotonylation of HSP90β to regulate ER stress

**Figure 5:**
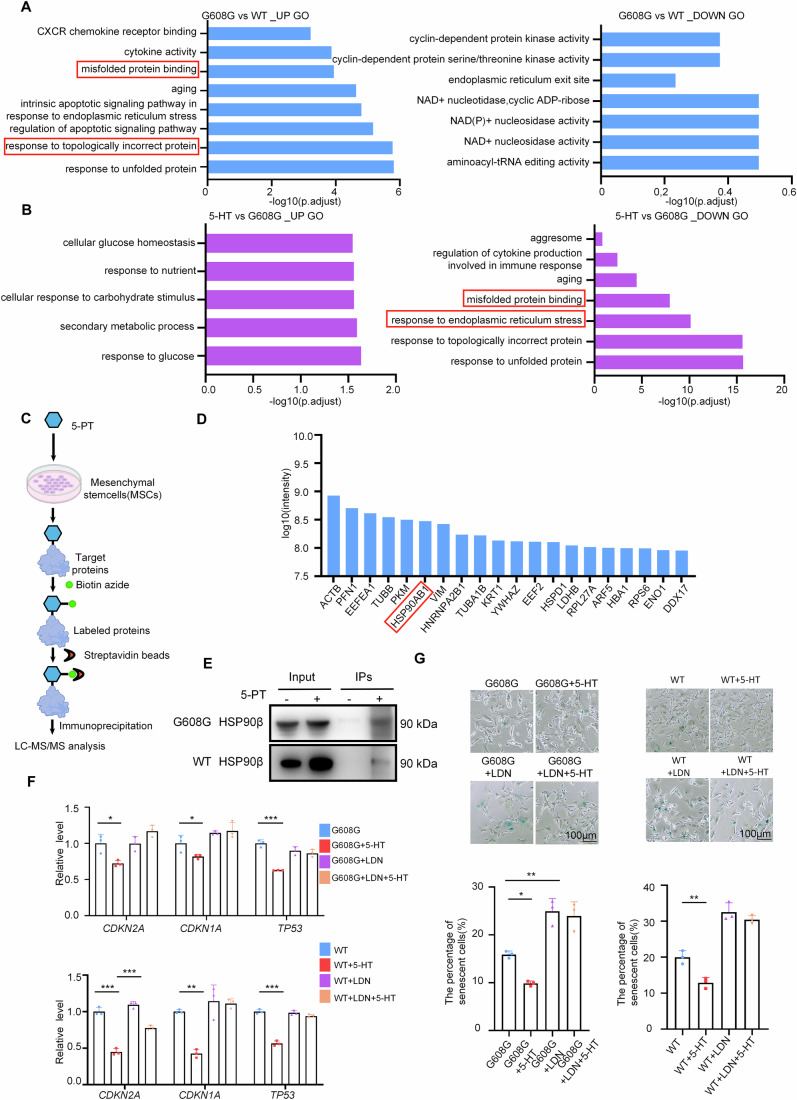
5-HT has post-translational modifications to HSP90β. (**A**, **B**) GO analysis of upregulated and downregulated genes between G608G MSC and WT MSC (**A**) and between G608G MSC with or without 5-HT treatment (**B**). GO enrichment was performed by over-representation analysis using a hypergeometric test (Fisher’s exact test), followed by Benjamini–Hochberg FDR correction (adjusted *P* values/q values). (**C**) Workflow for the discovery of serotonylated proteins by LC-MS/MS and IP. (**D**) Heatmap of the top 20 enriched proteins. (**E**) MSCs were cultured 72 h, with or without 5 μmol/L 5-PT. 5-PT was immunoprecipitated by beads, and HSP90β were detected. IP immunoprecipitation. Upper: G608G MSCs, Lower: WT MSCs. (**F**) RT-qPCR analysis of *CDKN2A*, *CDKN1A*, and *TP53* mRNA expression in MSCs treated with 5-HT, LDN or LDN + 5-HT, LDN: LDN-27219, TGM2 inhibitor. Upper: G608G MSCs, Lower: WT MSCs. *P < 0.05, **P < 0.01, ***P < 0.001 according to the one-way ANOVA test. G608G vs G608G + 5-HT: *CDKN2A*: *P* = 0.03; *CDKN1A*: *P* = 0.02; *TP53*: *P* < 0.001. WT vs WT + 5-HT: *CDKN2A*: *P* < 0.001; *CDKN1A*: *P* = 0.002; *TP53*: *P* < 0.001. WT + LDN vs WT + LDN + 5-HT: *CDKN2A*: *P* < 0.001. *n* = 3 biological repeats. (**G**) SA-β-Gal staining (upper) and quantification (lower) of MSCs treated with LDN or LDN + 5-HT. Left: G608G MSCs, Right: WT MSCs. *P < 0.05, **P < 0.01, ***P < 0.001 according to the one-way ANOVA test. G608G vs G608G + 5-HT: *P* = 0.03; G608G vs G608G + LDN: *P* = 0.003. WT vs WT + 5-HT: *P* = 0.006. *n* = 3 biological repeats. Scale bar, 100 μm. Data are presented as mean ± SD. See also Fig. EV7. Source data are available online for this figure.

**Figure EV6:**
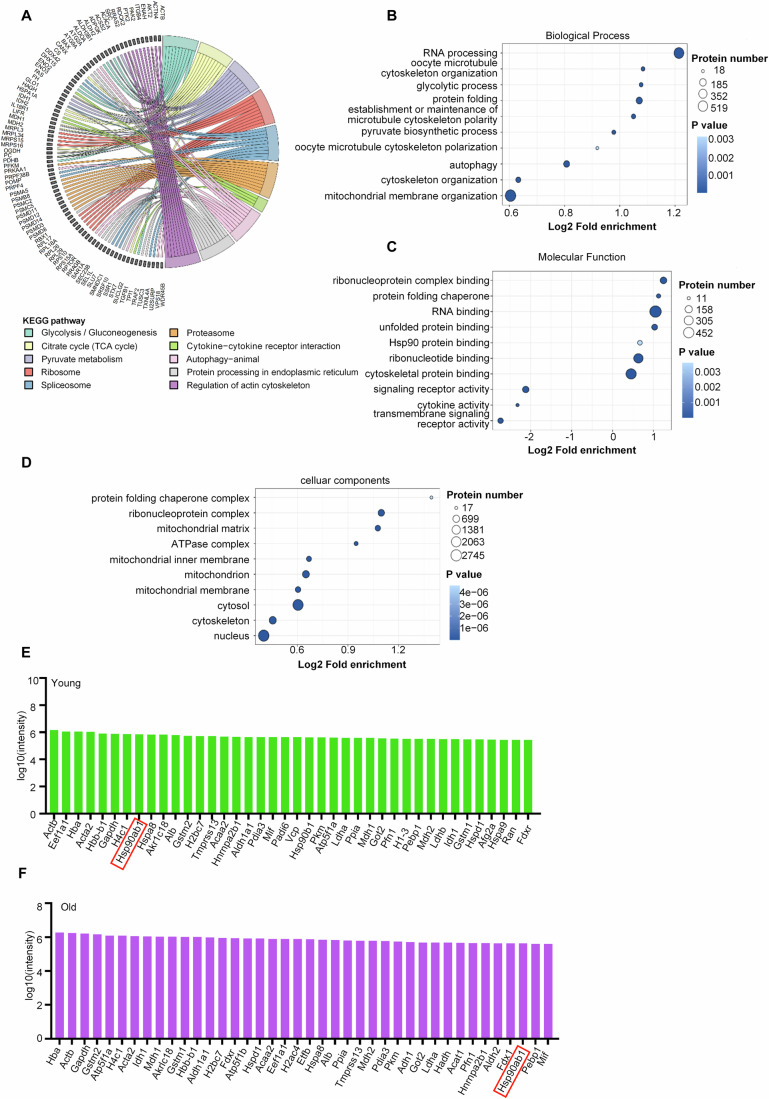
The potential targetes for serotonylation in MSCs. (**A**) KEGG analysis of seroyonylation proteins after mass spectrometry. (**B**–**D**) GO analysis of serotonylation proteins after mass spectrometry. GO enrichment was performed by over-representation analysis using a hypergeometric test (Fisher’s exact test), followed by Benjamini–Hochberg FDR correction (adjusted *P* values/*q* values). (**E**, **F**) Heatmap of the top 40 enriched proteins of the ovaries 4-week-old (Young) and 12-month-old mice (Old). Source data are available online for this figure.

Through comprehensive transcriptomic analysis comparing wild-type (WT) MSCs and G608G MSCs, we identified significant alterations in gene categories related to “misfolded protein binding” and “response to endoplasmic reticulum stress” (Fig. 5A). Notably, these molecular perturbations in G608G MSCs were effectively ameliorated by 5-HT supplementation (Fig. 5B). This observation prompted us to investigate the mechanistic role between 5-HT and unfolded protein response (UPR) pathways. Our findings suggest that 5-HT exerts its regulatory effects via covalent transamination mediated by transglutaminase 2 (TGM2), a post-translational modification that has been well-documented to influence gene expression profiles and metabolic activities (Bader, 2019; Farrelly et al, 2019; Jiang et al, 2021). Given that TGM2 is expressed in MSCs, we wondered whether serotonylation reaction occurred. Using click chemistry, propargylated 5-HT (5-PT) was delivered into cells to act as a bait to label target proteins, followed by immunoprecipitation to identify serotonylation proteins with liquid chromatography-tandem mass spectrometry (LC-MS/MS) (Fig. 5C), and the specific proteins captured by 5-PT are listed in Dataset EV2 (Lin et al, 2014). Global analyses showed that the top enriched proteins included those previously confirmed to be serotonylated, such as Actin (Jiang et al, 2021). Further KEGG analysis and GO analysis showed that these potential serotonylated proteins were mainly related to glycolysis, cytoskeleton, mitochondrial function, and endoplasmic reticulum function (Fig. EV6A–D). Interestingly, an endoplasmic reticulum stress and unfolded protein response-related protein HSP90β showed high intensity and was identified as a putative substrate for serotonylation (Fig. 5D). Western blotting after 5-PT pulldown further confirmed the results of LC-MS/MS in both WT and G608G cells (Fig. 5E). To preclude the possibility that the aforementioned modification takes place during the cell - culture process, we conducted intraperitoneal injections of 5-PT (at a concentration of 5 mM) into 4-week-old and 12-month-old mice for three consecutive days. Subsequently, we collected ovaries and utilized click chemistry to capture 5-PT-bound proteins for mass spectrometry analysis (Fig. EV6E,F), and the specific proteins captured by 5-PT are listed in Dataset EV3. The results demonstrated that the serotonylation modification of HSP90β is present not only in the in vitro setting but also in the in vivo environment. Notably, the abundance of this modification exhibits a downward trend as the age of mice increases.

**Figure EV7:**
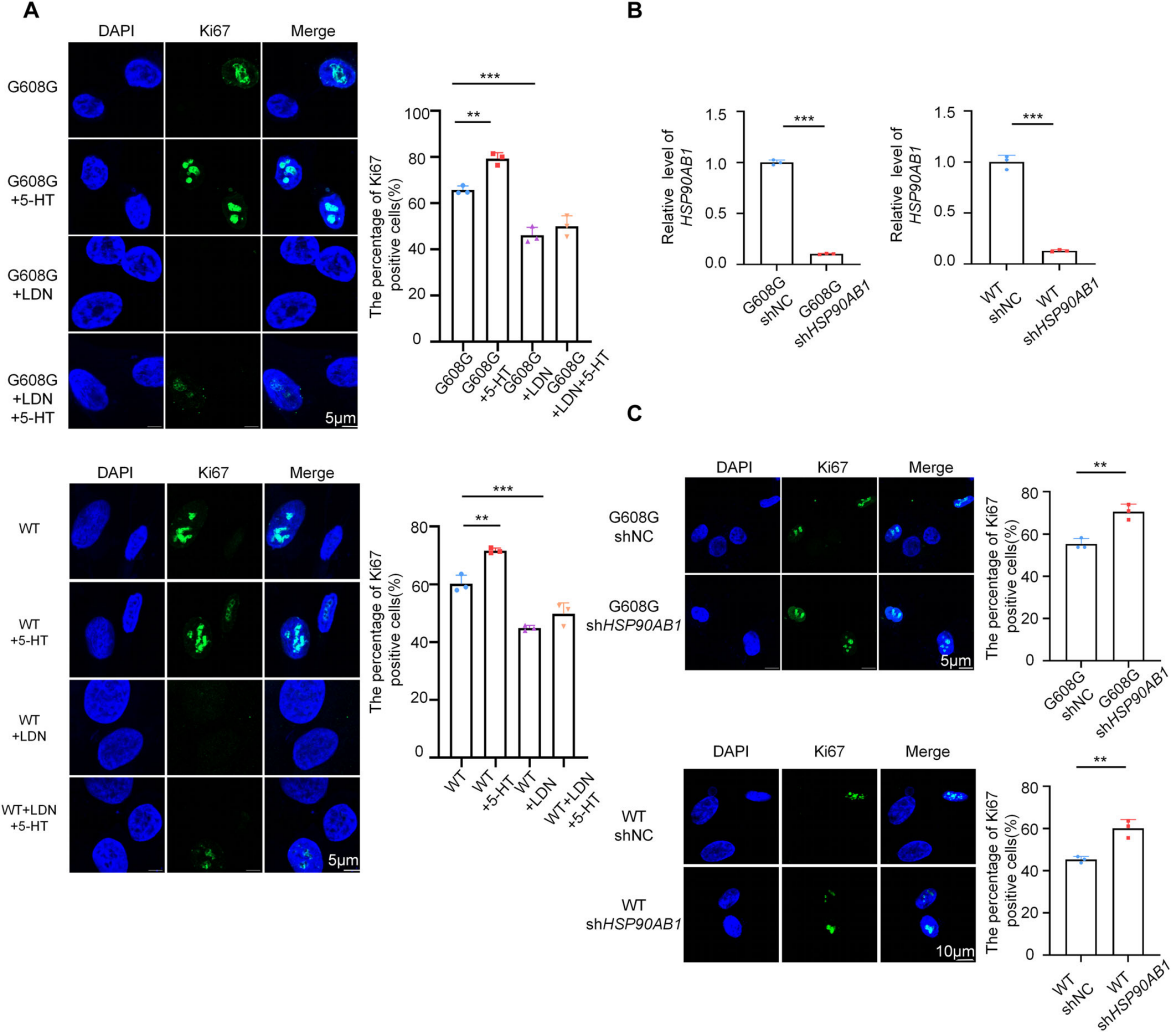
5-HT block cellular senescence by serotonylation of HSP90β. (**A**) Immunofluorescence analysis (left) and quantification of Ki67 (right) in MSCs treated with 5-HT, LDN or LDN + 5-HT. upper: G608G MSCs, lower: WT MSCs. *P < 0.05, **P < 0.01, ***P < 0.001 according to the one-way ANOVA test. G608G vs G608 + 5-HT: *P* = 0.004; G608G vs G608G + LDN: *P* < 0.001. WT vs WT + 5-HT: *P* = 0.002; WT vs WT + LDN: *P* < 0.001. *n* = 3 biological repeats. Scale bar, 5 μm. (**B**) RT-qPCR analysis of *HSP90AB1* mRNA expression in MSCs transfected with control shRNA or shRNA against HSP90AB1. Left: G608G MSCs, Right: WT MSCs. *P < 0.05, **P < 0.01, ***P < 0.001 according to the two-tailed unpaired *t* test. *P* < 0.001 (left), and *P* < 0.001 (right). *n* = 3 biological repeats. (**C**) Immunofluorescence analysis (left) and quantification of Ki67 (right) in MSCs transfected with control shRNA or shRNA against HSP90AB1. upper: G608G MSCs, lower: WT MSCs. *P < 0.05, **P < 0.01, ***P < 0.001 according to the two-tailed unpaired *t* test. *P* = 0.004 (upper), and *P* = 0.004 (lower). *n* = 3 biological repeats. Scale bar, 5 and 10 μm. Data are presented as mean ± SD. Source data are available online for this figure.

**Figure 6:**
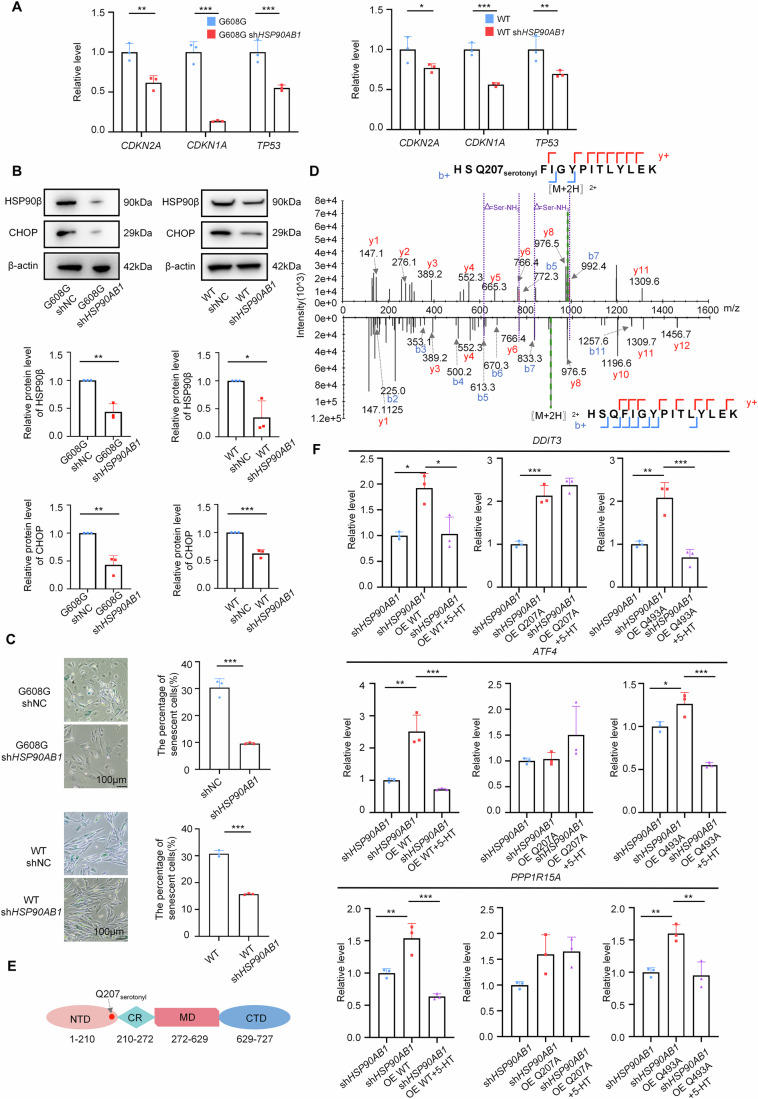
Serotonylation of Gln207 at HSP90β inhibits continuous stress function of HSP90β. (**A**) RT-qPCR analysis *CDKN2A*, *CDKN1A*, and *TP53* mRNA expression in MSCs transfected with control shRNA or shRNA against HSP90AB1. Left: G608G MSCs, Right: WT MSCs. *P < 0.05, **P < 0.01, ***P < 0.001 according to the two-way ANOVA test. *P* values from left to right: G608G MSCs: *P* = 0.002, *P* < 0.001, *P* < 0.001; WT MSCs: *P* = 0.02, *P* < 0.001, *P* = 0.004. *n* = 3 biological repeats. (**B**) Western blots of HSP90β, and CHOP protein levels in MSCs transfected with control shRNA or shRNA against HSP90AB1. Left: G608G MSCs, Right: WT MSCs. Lower: Quantification of the protein levels relative to β-actin. *P < 0.05, **P < 0.01, ***P < 0.001 according to the two-tailed unpaired *t* test. HSP90β: *P* = 0.002 (left), and *P* = 0.02 (right); CHOP: *P* = 0.004 (left), and *P* < 0.001 (right). *n* = 3 biological repeats. (**C**) SA-β-Gal staining (left) and quantification (right) of MSCs transfected with control shRNA or shRNA against HSP90AB1. Upper: G608G MSCs, Lower: WT MSCs. *P < 0.05, **P < 0.01, ***P < 0.001 according to the two-tailed unpaired *t* test. *P* < 0.001 (upper), and *P* < 0.001 (lower). *n* = 3 biological repeats. Scale bar, 100 μm. (**D**) LC-MS/MS analysis at Gln207 of TGM2-transamidated serotonin to full-length HSP90β, serotonylation peptide (top), unserotonylation peptide (below). ρ = Ser-NH3 is the difference in mass-charge ratio between b5 in the top and b5 in the bottom and between b7 in the top and b7 in the bottom, where the difference in mass-charge ratio is 159. (**E**) The domain of HSP90β and the representation of the serotonylation site. (**F**) shHSP90AB1 G608G MSCs were transfected with mRFP-fused lentiviruses expressing either WT-HSP90β or Gln to Ala(Q207A, Q493A) mutated HSP90β. Cells were treated with 5-HT or not. *DDIT3*, *ATF4*, and *PPP1R15A* mRNA expression was detected. *P < 0.05, **P < 0.01, ***P < 0.001 according to the one-way ANOVA test. *P* values from left to right: DDIT3: *P* = 0.01, and *P* = 0.01; *P* < 0.001, and *P* = 0.26; *P *= 0.003, and *P* < 0.001. ATF4: *P* = 0.002, and *P* < 0.001; *P* = 0.99, and *P* = 0.27; *P* = 0.02, and *P* < 0.001. PPP1R15A: *P* = 0.008, and *P* < 0.001; *P* = 0.08, and *P* = 0.97; *P* = 0.006, and *P* = 0.004. *n* = 3 biological repeats. Data are presented as mean ± SD. See also Figs. EV8 and 9. Source data are available online for this figure.

**Figure EV8:**
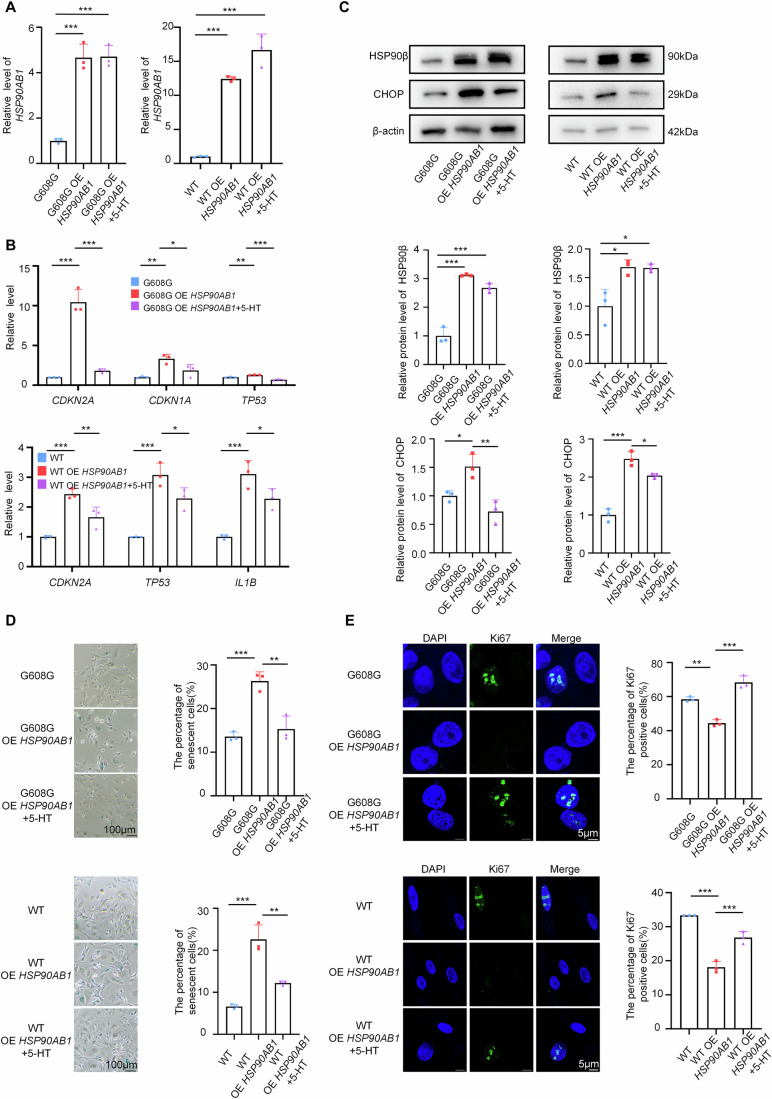
HSP90β is a potential target for inhibiting cell senescence. (**A**) RT-qPCR analysis of *HSP90AB1* mRNA expression in MSCs and HSP90AB1-overexpression MSCs with or without 5-HT treatment. Left: G608G MSCs, Right: WT MSCs. *P < 0.05, **P < 0.01, ***P < 0.001 according to the one-way ANOVA test. *P* values from left to right: *P* < 0.001, and *P* < 0.001; *P* < 0.001, and *P* < 0.001. *n* = 3 biological repeats. (**B**) RT-qPCR analysis of *CDKN2A*, *TP53*, and *IL1B* or *CDKN1A* mRNA expression in MSCs and HSP90AB1-overexpression MSCs with or without 5-HT treatment. Upper: G608G MSCs, Lower: WT MSCs. *P < 0.05, **P < 0.01, ***P < 0.001 according to the one-way ANOVA test. *P* values from left to right: G608G MSCs: *P* < 0.001, and *P* < 0.001; *P* = 0.005, and *P* = 0.04; *P* = 0.002, and *P* < 0.001. WT MSCs: *P* < 0.001, and *P* = 0.003; *P* < 0.001, *P* = 0.02; *P* < 0.001, and *P* = 0.02. *n* = 3 biological repeats. (**C**) Western blots of HSP90β, CHOP protein levels in MSCs and HSP90AB1-overexpression MSCs with or without 5-HT treatment. Left: G608G MSCs, Right: WT MSCs. Lower: Quantification of the protein levels relative to β-actin. *P < 0.05, **P < 0.01, ***P < 0.001 according to the one-way ANOVA test. HSP90β: *P* < 0.001, and *P* < 0.001 (left); *P* = 0.01, and *P* = 0.01 (right).CHOP: *P* = 0.03, and *P* = 0.004 (left); *P* < 0.001, *P* = 0.03 (right). *n* = 3 biological repeats. (**D**) SA-β-Gal staining (left) and quantification (right) of MSCs and HSP90AB1-overexpression MSCs with or without 5-HT treatment. Upper: G608G MSCs, Lower: WT MSCs. *P < 0.05, **P < 0.01, ***P < 0.001 according to the one-way ANOVA test. *P *< 0.001, and *P* = 0.002 (upper); *P* < 0.001, and *P* = 0.002 (lower). *n* = 3 biological repeats. Scale bar, 100 μm. (**E**) Immunofluorescence analysis (left) and quantification of Ki67 (right) in MSCs and HSP90AB1-overexpression MSCs with or without 5-HT treatment. upper: G608G MSCs, lower: WT MSCs. *P < 0.05, **P < 0.01, ***P < 0.001 according to the one-way ANOVA test. *P* = 0.002, and *P* < 0.001 (upper); *P* < 0.001, and *P* < 0.001 (lower). *n* = 3 biological repeats. Scale bar, 5 μm. Data are presented as mean ± SD. Source data are available online for this figure.

To evaluate the effect of TGM2-mediated serotonylation on the aging effects in the two pathological and natural aging ex vivo models, a TGM2 inhibitor LDN-27219 was employed. We observed the senescence-alleviating effect of 5-HT supplementation was almost completely reduced (Figs. 5F,G and  EV7A), indicating that TGM2-mediated serotonylation predominantly contributed to the alleviation of the aging effect. In order to elucidate the specific relationship between aging, HSP90β, and 5-HT, we performed a knockdown experiment of HSP90β (Figs. 6B, and  EV7B). It led to reduced senescence marker gene expression and β-gal staining (Fig. 6A–C) and increased Ki67 staining for both WT and G608G cells (Fig. EV7C). Accordingly, overexpression of HSP90β promoted the aging effect, and 5-HT supplementation reversed it (Fig. EV8A–E). HSP90β overexpression also caused upregulation of severe ER stress marker genes such as CHOP (*DDIT3*), which could be rescued by 5-HT (Fig. EV8C), and the result was confirmed by knocking down HSP90β (Fig. 6B). The above data suggest that 5-HT plays an important role through serotonylation and repression of HSP90β to prevent persistent ER stress.

**Figure EV9:**
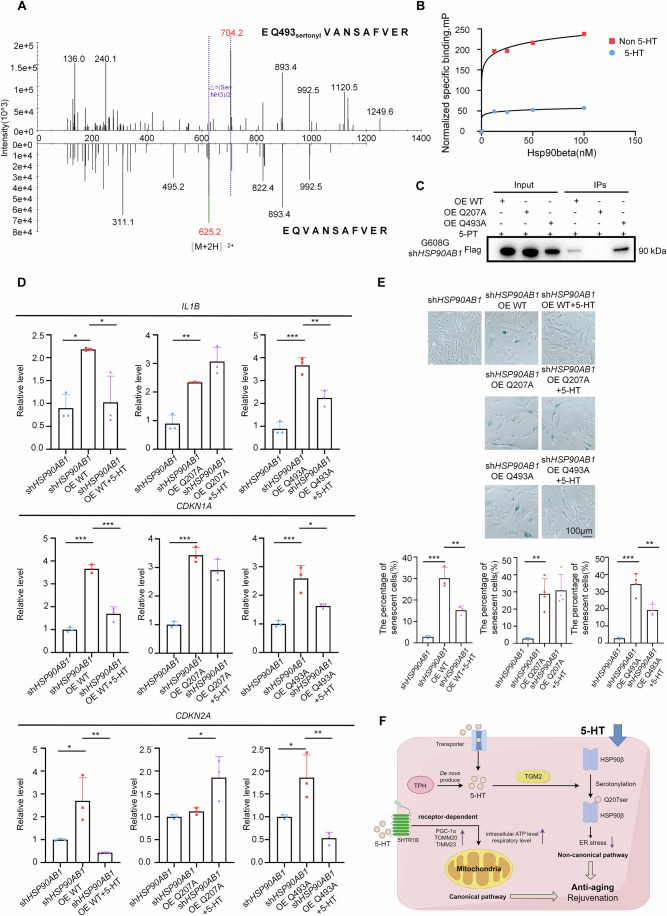
Mutation of Gln207 at HSP90β inhibits the role of 5-HT in rejuvenation effects. (**A**) LC-MS/MS analysis at Gln493 of TGM2-transamidated serotonin to full-length HSP90β, serotonylation peptide (top), unserotonylation peptide (below). ρ = Ser-NH3 is the difference in mass-charge ratio between two parent ion, where the difference in mass-charge ratio is 159. (**B**) The ATPase activity of HSP90β. Fluorescence was measured at λex 485 nm, λex 530 nm using a Bio-Tek fluorescent microplate reader. (**C**) shHSP90AB1 G608G MSCs were transfected with mRFP-fused lentiviruses expressing either WT-HSP90β or Gln to Ala(Q207A, Q493A) mutated HSP90β. Cells were treated with 5 μM 5-PT or not. 5-PT was immunoprecipitated by beads, and Flag-HSP90β were detected. IP immunoprecipitation. (**D**) shHSP90AB1 G608G MSCs were transfected with mRFP-fused lentiviruses expressing either WT-HSP90β or Gln to Ala(Q207A, Q493A) mutated HSP90β. Cells were treated with 5-HT or not. *IL1B*, *CDKN1A* and *CDKN2A* mRNA expression was detected. *P < 0.05, **P < 0.01, ***P < 0.001 according to the one-way ANOVA test. *P* values from left to right: *IL1B*: *P* = 0.01, and *P* = 0.02; *P* = 0.005, and *P* = 0.08; *P* < 0.001, and *P* = 0.005. *CDKN1A*: *P* < 0.001, and *P* < 0.001; *P* < 0.001, and *P* = 0.13; *P* < 0.001, and *P* = 0.01. *CDKN2A*: *P* = 0.03, and *P* = 0.007; *P* = 0.85, and *P* = 0.04; *P* = 0.03, and *P* = 0.004. *n* = 3 biological repeats. (**E**) SA-β-Gal staining (upper) and quantification (lower) of shHSP90AB1 G608G MSCs transfected with mRFP-fused lentiviruses expressing either WT-HSP90β or Gln to Ala (Q207A, Q493A) mutated HSP90β, cells were treated with 5-HT or not. *P < 0.05, **P < 0.01, ***P < 0.001 according to the one-way ANOVA test. *P* values from left to right: *P* < 0.001, and *P* = 0.003; *P* = 0.006, and *P* = 0.93; *P* < 0.001, and *P* = 0.002. *n* = 3–4 biological repeats. Scale bar, 100 μm. (**F**) Schematics showing that 5-HT regulates cellular aging through both classical and non-classical functions, by Figdraw. Data are presented as mean ± SD. Source data are available online for this figure.

**Figure 7:**
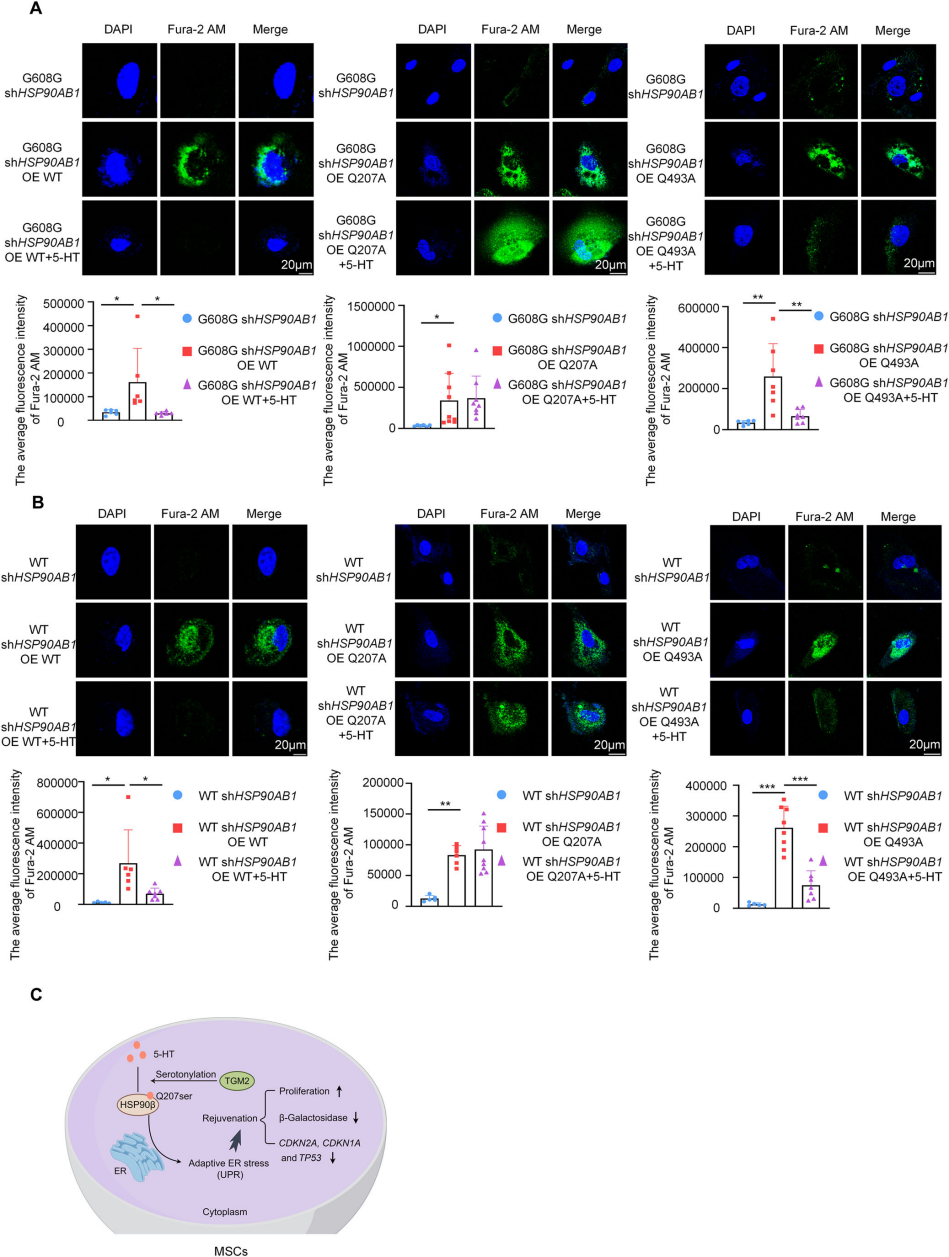
Serotonylation on Gln207 of HSP90β restrains the continuous stress function that HSP90β exerts. (**A**) shHSP90AB1 G608G MSCs were transfected with mRFP-fused lentiviruses expressing either WT-HSP90β or Gln to Ala(Q207A, Q493A) mutated HSP90β. Cells were treated with 5-HT or not. Immunofluorescence analysis and quantification of Fura-2 AM. *P < 0.05, **P < 0.01, ***P < 0.001 according to the one-way ANOVA test. *P* values from left to right: *P* = 0.04, and *P* = 0.04; *P* = 0.04, and *P* = 0.83; *P* = 0.002, and *P* = 0.005. *n* ≥ 3 biological repeats. Scale bar, 20 μm. (**B**) shHSP90AB1 WT MSCs were transfected with mRFP-fused lentiviruses expressing either WT-HSP90β or Gln to Ala(Q207A, Q493A) mutated HSP90β. Cells were treated with 5-HT or not. Immunofluorescence analysis and quantification of Fura-2 AM. *P < 0.05, **P < 0.01, ***P < 0.001 according to the one-way ANOVA test. *P* values from left to right: *P* = 0.01, and *P* = 0.03; *P* = 0.002, and *P* = 0.81; *P* < 0.001, and *P* < 0.001. *n* ≥ 3 biological repeats. Scale bar, 20 μm. (**C**) Schematics showing that 5-HT induced HSP90β Q207ser serotonylation and reduced ER stress in MSC cells (by Figdraw). Data are presented as mean ± SD. Source data are available online for this figure.

To further identify the sites of serotonylation on HSP90β, LC-MS/MS following in vitro serotonylation assays with 5-HT was performed. Specifically, the in vitro purified HSP90 protein was co-incubated with 5-HT and the recombinant TGM2 enzyme to allow modification, followed by mass spectrometry. The result revealed that the Gln (Q) residues at 207 and 493 were serotonylated (Figs. 6D and  EV9A). Gln207 resides in the N-terminal domain with ATPase activity of HSP90β (Fig. 6E), suggesting that 5-HT modification of HSP90β may affect the activity of HSP90β to abolish its role in aging. Further experiments assessing the ATPase activity of the N-terminal domain of HSP90β confirmed this hypothesis, revealing that the ATPase activity of HSP90β was significantly reduced following modification by 5-hydroxytryptamine (Fig. EV9B). To prove serotonylation of these sites contributed to the 5-HT’s cell senescence alleviation effects, mRFP-fused lentiviruses expressing either WT-HSP90β or Gln to Ala (Q207A, Q493A) mutated HSP90β were made and transduced into the cells which endogenous wild-type HSP90β knocked down. The results showed that Q207A-HSP90β mutated cells could not interact with 5PT (Fig. EV9C), abolished the 5-HT effect, and had worsened ER stress and dysregulated unfolded protein response genes upon 5-HT treatment, whereas this was not the case for the Q493A-HSP90β control mutant (Figs. 6F and  EV9D). We further quantified cytoplasmic calcium concentrations using Fura-2 AM fluorescence imaging, a reporter for ER stress. While 5-HT treatment significantly reduced cytosolic Ca²⁺ levels in WT-HSP90β and Q493A-HSP90β cells, Q207A-HSP90β mutants maintained persistently elevated calcium levels even after 5-HT administration (Fig. 7A,B). Consistent with these findings, senescence-associated β-galactosidase (SA-β-gal) activity assays demonstrated that 5-HT treatment reduced positive staining by 20% in WT-HSP90β cells and 17% in Q493A-HSP90β cells but produced no significant reduction in Q207A-HSP90β cells (Fig. EV9E). Altogether, these data demonstrated that 5-HT relieves cellular senescence through serotonylation of the Q207 amino acid of HSP90β and alleviation of ER stress (Fig. 7C).

## Discussion

In this study, we first characterized the reductive metabolic signature of oocyte and identified 5-HT as an oocyte-enriched metabolite capable of attenuating senescence phenotypes in MSCs and aged mouse tissues. Beyond its classical role as a neurotransmitter, 5-HT can covalently modify proteins through serotonylation. Here, we demonstrate that 5-HT acts through two complementary mechanisms: a classical pathway promoting mitochondrial regeneration, and a newly identified serotonylation of HSP90β at Q207 that modulates ER stress and cellular senescence (Fig. EV9F).

Employing pooled mouse oocytes and early embryos and MS-based ultra-sensitive targeted metabolomics approach, we provided a comprehensive metabolomics profiling of oocytes, zygotes and 2-cell stage embryos. KEGG enrichment pathway characterized the reductive metabolic signature of oocyte, notably, static metabolomic profiles are insufficient for directly assessing metabolic pathway activity, therefore, isotope tracing studies in embryos warrant further investigation. Among a range of oocyte-specific metabolites associated with reductive characteristics, we found that 5-HT, a metabolite previously known as a neurotransmitter, showed strong efficacy in attenuating aging defects in MSCs and tissues of aged mice. Most of the previous studies on 5-HT focused on its role in neuronal cells and mechanisms of brain cognition decline(Mattson et al, 2004; Ramirez et al, 2014), and the systematic role of 5-HT in the context of non-neuronal cells’ cellular senescence/rejuvenation has never been reported. Besides, an overlooked part of 5-HT’s function is that it can form a covalent bond between its amino group and the amide group on glutamine of proteins, namely serotonylation, and the modified protein will have altered activity of the targeted protein (Bader, 2019). A serotonylation target related to aging and cellular rejuvenation has never been reported before. We have not only demonstrated the classical pathway by which 5-HT regulates mitochondrial regeneration but also demonstrated HSP90β as a novel serotonylation target and elucidated the precise modified amino acid and its associated functions in regulating cellular ER stress and senescence (Fig. EV9F).

It is important to note that our metabolomics measurements were obtained from pooled samples of 100 oocytes or embryos per replicate. While this approach is necessary for early-embryo metabolomics due to extremely low per-cell metabolite abundance, it does not capture embryo-to-embryo variability and limits inference to population-level metabolic trends. These data therefore support broad metabolic differences between stages, but do not provide variance estimates at the level of individual embryos. What’s more, the pathway enrichment analysis in this study is descriptive and intrinsically constrained by the targeted metabolite panel. Therefore, enriched pathways should be viewed as hypothesis-supporting rather than discovery-level evidence, and further unbiased metabolomic profiling will be needed to confirm these trends.

A line of evidences further support the dual mechanisms in alleviating aging. For instance, HSP90 inhibitors have demonstrated significant anti-aging properties.(Luo et al, 2010) Janssens et al found that monorden and tanespimycin, two inhibitors of HSP90, extended lifespan and improved health in C. elegans (Janssens et al, 2019). Another inhibitor of HSP90, alvespimycin, effectively reduced kidney senescence in diabetic mice (Tesch et al, 2024). In addition, it has been revealed that 5-HTR1B knockout mice exhibit a significantly shortened lifespan and early-onset motor deficits, establishing 5-HTR1B signaling as a critical factor for healthy aging (Sibille et al, 2007). Although TGM2 inhibitors or 5-HTR1B antagonists have not been directly tested in G608G mice, our in vitro experiments suggest these interventions could reverse 5-HT’s effects in vivo, while acknowledging that these small molecules often have multiple targets with complex effects on lifespan. While SSRIs have been reported to affect aging-related phenotypes in some epidemiological or small clinical studies, these effects are likely mediated through central serotonergic pathways rather than through systemic 5-HT or peripheral serotonylation(Okamura et al, 2000). Because SSRIs selectively target neuronal SERT, their relevance to peripheral aging remains speculative, and our study was not designed to address this question.

Several proteins have been shown to undergo serotonylation modification after translation, including H3 Q5ser (Chen et al, 2024; Farrelly et al, 2019), GAPDH Q262ser (Wang et al, 2024), and HSP90β Q207ser in our study. These modifications are involved in various cellular processes, such as the activation of transcription factors (Bader, 2019), immune responses (Wang et al, 2024), and aging. What other proteins can undergo serotonylation modification, whether the modified protein assumes a unique structure, and whether each modification occurs in a specific cell type are all questions that need to be further explored in future studies.

Our conclusions are primarily based on iPSC-derived MSCs and mouse models, which may not fully recapitulate the complexity of human tissue aging in vivo. iPSC-derived MSCs represent a developmentally reset and culture-adapted cell population, and their epigenetic and metabolic states may differ from those of primary tissue-resident MSCs. Likewise, the doses and routes of 5-HT administration used in mice (chronic oral supplementation) may not be directly translatable to humans because of species-specific differences in serotonin metabolism, receptor expression profiles, and blood–brain barrier transport. In addition, aging is a multifactorial process, and our interventions likely modulate multiple parameters simultaneously, including oxidative stress, mitochondrial dynamics, ER stress, inflammatory signaling and cell-cycle regulation. We cannot exclude the possibility that changes in other oocyte-enriched metabolites, alterations in systemic metabolic state, or compensatory stress responses also contribute to the observed rejuvenation phenotypes. Future work using genetic or pharmacological tools to more selectively manipulate serotonylation or specific 5-HT receptor subtypes will be required to dissect the contribution of each pathway.

Although anti-aging therapies have achieved tremendous progress, novel metabolite-mediated aging alleviation therapies are worth further investigation. The identification of 5-HT and serotonylation as modulators of cellular senescence provides a conceptual framework and a set of tractable molecular targets for future mechanistic and preclinical studies, although substantial work will be needed before any clinical applications can be envisaged.

## Methods

**Table:** Reagents and tools table

Reagent/resource	Reference or source	Identifier or catalog number
**Experimental models**		
C57BL/6-Tg(LMNA*G608G)HClns/J	Jackson lab	#010667
C57BL/6J	Zhejiang University Animal House	
Human WT iPSC	Wang et al (2023)	
Human HGPS iPSC	Liu Zhihong lab	
HEK 293T	Wang et al (2023)	
**Recombinant DNA**		
hHSP90AB1 (NM_001271969)-pCDH-CMV-T2A-EGFP-EF1A-Puro	Fenghui Biotechnology Co., LTD	
Flag-hHSP90AB1 207gln-ala (NM_001271969)-pCDH-CMV-T2A-mRFP-EF1A-Puro	This study	
Flag-hHSP90AB1 493gln-ala (NM_001271969)-pCDH-CMV-T2A-mRFP-EF1A-Puro	This study	
hHSP90AB1-LV3(H1/GFP&Puro)	GemmaPharma	
LV3(H1/GFP&Puro)	GemmaPharma	
qPCR	This study	Table EV1
**Antibodies**		
Anti-β-Actin	CST	4970S
Anti-β-actin	HUABIO	HA722023
Anti-Hsp90 beta	HUABIO	ET1605-56
Anti-Serotoin	Sigma	S5545
Anti-DDit3	HUABIO	ET1703-05
Anti-TIMM23	Abconal	A8688
Anti-TOMM20	HUABIO	RT1609-25
Anti-p-H2AX	CST	9718S
Anti-NCL	CST	14574S
Anti-α-Tubulin-FITC	Sigma	F2168
Anti-Ki67	Boster	M00254-8
Brilliant Violet711^TM^ anti-human CD45	Biolegend	304049
PE anti-CD105	Biolegend	800504
FITC anti-human CD90	Biolegend	328108
APC anti-human CD73	Biolegend	344006
**Oligonucleotides and other sequence-based reagents**		
hHSP90AB1 shRNA	Zhang et al (2022)	5′-AGTAAACTAAGGGTGTCAACTCGAGTTGACACCCTTAGTTTACTGC-3′
**Chemicals, enzymes and other reagents**		
mTeSR™1 Basal Medium	STEMCELL	85851
Y-27632	Sigma	SCM075
Human VEGF	Abclonal	RP01150
Human BMP4	Abclonal	RP02512S1
Human SCF	Abclonal	RP00124
Hunab TGFβ	Proteintech	HZ-1011
Human bFGF	Proteintech	HZ-1285
DMSO	Sigma	D2650
5-PT	MCE	HY-156562A
5-HT	MCE	HY-B1473
5-HT	Sigma	14927
5-HT1B receptor	MCE	HY-101105A
TGM2 protein	MCE	HY-P76114
HSP90β protein	abcam	ab80033
**Software**		
GraphPad Prism 9.5.1	GraphPad China	RRID: SCR_002798
Fiji	https://imagej.net	RRID:SCR_002285
Endnote 9.0	https://endnote.com/	RRID: SCR_014001
3DHISTECH SlideViewer	https://www.3dhistech.com/downloads/	
ZEN 3.4 (blue edition)	https://www.zeiss.com/microscopy/en/service-support/downloads.html	RRID:SCR_013672
**Other**		

### Mouse oocyte collection and culturing

C57BL/6 and homozygous LMNA c. G608G C>T mice were housed in the Zhejiang University animal facility. The mice were maintained on a 12-h light/12-h dark cycle. The diet catalog number was 1010085 (Xietong). All mice were handled according to Zhejiang University Animal Research Institute Committee guidelines (protocol number: ZJU20250819). GV oocytes were collected from female C57BL/6 J mice (4 weeks old) that had each been intraperitoneally injected with 7.5 IU pregnant mare serum gonadotropin (PMSG) and killed by cervical dislocation 44 h post-PMSG injection. The MII oocytes were collected by injecting mice with 7.5 IU human chorionic gonadotropin (hCG) 48 h after PMSG priming. Cumulus-oocyte complexes (COCs) were isolated from the oviduct ampullae, and cumulus masses were removed in a medium containing 0.5 mg/mL hyaluronidase at 37 °C. Embryos at different stages were collected after the super-ovulated female mice had mated with C57BL/6 adult males overnight after hCG administration. The embryos were collected at 22–24 h (zygotes) and 40–44 h (mid-2C) post-hCG administration. The zygote collection was like that of the MII oocytes, and the 2 C embryos were collected from the oviducts.

### In vitro mouse embryo culture

Embryos were cultured by culturing GV and MII in a 30-µL drop of M16 medium (Millipore) in mineral oil (Sigma-Aldrich) at 37 °C, 5% CO_2_, and 100% humidity. For metabolite supplementation, pronuclear embryos were cultured in M16 medium (Millipore) containing 5-HT (5 μM). The MII maturity ratio was recorded at 20 h after milrinone removal.

### Cell lines and cell culture

No new human samples were collected for this study. All experiments were performed using pre‑existing human iPSC lines under approved IRB protocols. The HGPS‑iPSC line carrying the CG608GT LMNA mutation was kindly provided by Dr. Liu Zhihong. The use of all human iPSC lines in this study was reviewed and approved by the Institutional Review Board of Zhejiang University (IRB protocol number 2023‑IRB‑0186‑P‑01). All hiPSC were cultured in mTeSR Basal Medium with 5× Supplement (STEMCELL). All MSCs were cultured in Dulbecco’s modified Eagle’s medium (DMEM) containing 10% fetal bovine serum (FBS, Hakata), 2 mM L-glutamine, 0.1 mM NEAA (all, Gibco), and 1 ng/mL FGF-basic (PeproTech). All the cellular experiments used MSCs of the same generation (P12-P15); each passage was carried out in the same proportion. The WT MSCs of passages P3-P5 are defined as young, and P12–P15 is defined as old. HEK293T cells were cultured on DMEM with 4.5 g/L glucose, L-glutamine, and sodium pyruvate (Meilunbio) supplemented with 10% FBS and 1× penicillin/streptomycin (Gibco). 5-HT (5 μM, MCE, HY-B1473) was added to the medium, and the cells were treated for 72 h.

### MSC generation through iPSC differentiation

The MSCs were differentiated as previously described (Wu et al, 2018). Briefly, iPSC was dissociated into EBs and plated on Matrigel-coated plates in MSC differentiation medium [α-MEM+GlutaMAX (Gibco), 10% FBS (Hakata), 1% penicillin/streptomycin (Gibco), 10 ng/mL FGF2 (PeproTech), and 5 ng/mL TGFβ (PeproTech, 96-100-21-2)]. After 10 days, the confluent MSC-like cells were passed on a gelatin-coated plate and cultured in MSC culture medium [90%α-MEM+GlutaMAX (Gibco), 10% FBS (Hakata), 1% penicillin/streptomycin (Gibco), and 1 ng/mL FGF2]. The resulting adherent cells exhibiting typical spindle-shaped morphology were expanded for at least two passages and characterized by flow cytometry. These cells expressed the canonical MSC surface markers CD73, CD90, CD105 and CD45 (Figure EV2). In this study, these iPSC-derived mesenchymal progenitor-like populations are referred to as “MSCs” throughout the manuscript (Pan et al, 2016; Zhang et al, 2015).

### Metabolite extraction and targeted metabolomics analysis

The previously described method was followed for metabolite extraction and analysis (Zhao et al, 2021). For each developmental stage, 100 oocytes or embryos were pooled to generate one biological replicate. Owing to the extremely low metabolite abundance in single embryos and the volume constraints of extraction, pooling is required to achieve sufficient detection sensitivity, consistent with prior low‑input embryo metabolomics studies. In all analyses, the biological unit of inference is the pooled sample, not individual embryos. As is standard for early‑embryo metabolomics, formal power calculations were not feasible due to the necessity of pooling; therefore results are interpreted at the level of pooled‑sample biological replication. Embryos were stored in cold 80% methanol at −80 °C overnight. Following cell lysis, centrifugation at 14,000×*g* for 15 min at 4 °C was performed, and the resulting supernatant was transferred to a pre-chilled tube on dry ice and evaporated using a speed vacuum. The dried metabolites were then reconstituted in 30 μL of 0.03% formic acid in the water, vortexed, and centrifuged at 14,000×*g* for 15 min at 4 °C. Liquid chromatography-tandem MS (LC-MS/MS) analysis was carried out on the supernatant. The LC procedure utilized a UHPLC system (Nexera X2 LC-30A, Shimadzu) with an ACQUITY UPLC HSS-T3 UPLC column (150 mm × 2.1 mm, 1.8 μm, Waters). A gradient of 0–3 min with 1% mobile phase B, 3–15 min with 1%–99% B, 15–17 min with 99% B, 17–17.1 min with 99%–1% B, and 17.1–20 min with 1% B was employed, where mobile phase A consisted of 0.03% formic acid in water and mobile phase B comprised 0.03% formic acid in acetonitrile (ACN). The flow rate was set at 0.25 mL/min, the column temperature was maintained at 35 °C, and samples in the autosampler were kept at 4 °C. Injection volume was 20 μL. MS was performed using a triple-quadrupole mass spectrometer (QTRAP 6500 + , SCIEX) in multiple-reaction monitoring (MRM) mode, monitoring a total of 124 metabolites. Metabolites were identified by optimizing the MRM transitions of each metabolite and determining retention times using analytical standards. This method encompassed 124 metabolites from common metabolic pathways (energy metabolism, carbohydrate metabolism, amino acid metabolism, and nucleotide metabolism).

Chromatogram review and peak area integration were done using MultiQuant v.3.0 (SCIEX). Each area for detected metabolite was normalized against the total ion count of each sample, representing a fraction of the total detected metabolite content. This normalization method has been described in previous studies (Park et al, 2020). The results were further normalized to the total metabolite content, and normalized peak areas were utilized as variables for multivariate and univariate statistical analyses. In addition, all raw peak areas and the corresponding normalized data, including Q1, Q3, and CE information, are provided in Dataset EV1.

We acknowledge that our targeted metabolomics approach is based on a preselected metabolite pool (*n* = 124). However, this selection was carefully designed to cover major metabolic pathways, including central carbon metabolism, nucleotide metabolism, amino acid metabolism, one-carbon metabolism, and lipid metabolism. In addition, the metabolite pool was established based on previously published data capturing metabolites detectable in early embryonic tissues, oocytes, and embryonic stem cells (Zhao et al, 2021). By focusing on these well-characterized embryonic-relevant metabolites and pathways, we aimed to minimize potential bias while maintaining biological relevance to early development.

### Senescence-associated β-galactosidase (SA- β-Gal) staining

SA-β-Gal staining was performed using an SA-β-Gal staining kit (Beyotime, RG0039, China) according to the manufacturer’s protocol by Zhang et al, Briefly, 50,000/well cells were grown in 12-well plates. After washing once with phosphate-buffered saline (PBS), the cells were fixed in SA-β-Gal fresh fixative, incubated at room temperature for 15 min, then stained with SA-β-gal staining solution at pH 6.0 and 37 °C overnight. Senescent cells were identified under a light microscope.

### RNA extraction and RT-qPCR

Total RNA was isolated from cells or tissue using an RNA-Quick purification kit (ES-RN001, YISHAN) and a Tissue RNA purification kit (ES-RN002plus, YISHAN) according to the manufacturer’s protocol. RNA (500 ng) was reverse transcribed to complementary DNA (cDNA) with RTIII All-in-One mix with dsDNase (MR05101M, Monad). Target gene expression was measured with TB Green Premix (RR820A, TAKARA) on a LightCycler 480 Instrument II System (Roche) or a QuantStudio 6 Flex machine (ABI). Genes from the cells were normalized to the reference gene GAPDH. Tissue gene expression was calculated using the 18S rRNA gene as an endogenous control. Table EV1 lists all primers used.

### ATP measurement

The MSCs were cultured in a 12-well plate for 72 h. Whole cells were lysed with lysis buffer according to the ATP assay kit instructions (S0026, Beyotime). After centrifugation, the supernatant was added to the ATP detection solution, and luminescence was read using a Spark multimode microplate reader. The protein content was tested according to a Pierce bicinchoninic acid (BCA) protein assay kit.

### RNA sequencing (RNA-seq) analysis

Total RNA was isolated from cells as previously described. The RNA amount and purity were quantified using a Nanophotometer NP80. The RNA underwent commercial RNA-seq analysis (LC-Biotechnology Co., Ltd., Hangzhou, China). The average insert size for the final cDNA library was 300 ± 50 bp, and 2 × 150-bp paired-end sequencing (PE150) was performed on an Illumina NovaSeq™ 6000 following the vendor’s recommended protocol.

### Tissue/cell immunofluorescence

The mouse tissue was immersed in 4% paraformaldehyde (PFA) overnight at room temperature. Then, the tissue was dehydrated at 4 °C by 10% and 30% sucrose gradient solutions in sequence until the tissue had sunk to the bottom. The tissue was embedded in an optimal cutting temperature (OCT) compound and cut into 50-µm thick sections on the sagittal plane using a freezing microtome (CryoStar NX50, Thermo). The sections were permeabilized in 0.1% (v/v) Triton X in PBS for 30 min at room temperature and washed thrice with PBS. Then, the slides were blocked with a blocking solution [3% (v/v) bovine serum albumin (BSA)] for 2 h. After three washes in PBS, the slides were incubated with anti-goat primary antibodies overnight at 4 °C and washed. Subsequently, the sections were incubated in a secondary antibody for 2 h at room temperature and counterstained with DAPI to label the nuclei. Finally, the slices were mounted onto slides, and images were acquired with a fluorescence microscope. As the cells had been grown on gelatin-coated glass coverslips, dehydration, and embedding were not required. Nevertheless, all other steps were consistent with those used for the tissue samples.

### Western blotting

Protein extracts were obtained by radioimmunoprecipitation assay (RIPA) with a protease inhibitor cocktail (1:100, Meilunbio). The tissue samples were ground in an ultrasonication machine. The supernatant was collected, and the protein concentration was measured by a BCA protein assay kit (Thermo Fisher Scientific). The protein extract (30–60 µg) underwent electrophoresis on 10% and 15% Tris-glycine sodium dodecyl sulfate–polyacrylamide gel electrophoresis (Tris-Gly SDS-PAGE) and was transferred to a 0.22-μm PVDF membrane (Millipore). The membrane was blocked in TBST containing 5% skim milk for 1 h at room temperature and incubated in QuickBlock™ Primary Antibody Dilution Buffer (Beyotime) with primary antibody at 4 °C overnight. Then, the membrane was incubated in 5% skim milk with horseradish peroxidase (HRP)-conjugated secondary antibody and visualized using ECL chemiluminescence. The band intensity was measured using ImageJ (NIH).

### Cellular bioenergetics analysis

Real-time mitochondrial respiration was analyzed using the Seahorse XF96 Extracellular Flux Analyzer (Agilent, Santa Clara, USA) according to the manufacturer’s protocol. Briefly, the oxygen consumption rate (OCR) per minute in cell cultures subjected to sequential addition of the following respiration modulators was measured to determine the bioenergetics parameters: 1.5 µM oligomycin (ATP synthase inhibitor; Cat. No. 103015-100, Agilent), 1 µM carbonyl cyanide 4- (trifluoromethoxy) phenylhydrazone (FCCP, an ionophore and mitochondrial uncoupler), and 0.5 µM antimycin (a mitochondrial complex III inhibitor). The MSCs were plated at a density of 1.2 × 10^4^ cells/well in 150 µL of their corresponding seeding medium. Before the experiment, the medium was changed to DMEM without sodium bicarbonate supplemented with 1 mM pyruvate and 5 mM glucose. The mitochondrial respiratory parameters (basal respiratory capacity, maximal respiratory capacity, spare respiratory capacity, proton leak, and ATP-coupled respiration) were calculated using the OCR values. Three to four points per experimental condition were analyzed in at least three independent experiments per cell type.

### Lentivirus production

PEI (40 µL) was dissolved in 500 µL serum-free Opti-MEM (Gibco) and incubated in the solution for 5 min. The packaging vectors pMD2.G and psPAX2 and lentivirus vectors carrying the target gene were dissolved in 500 µL Opti-MEM (in total, 20 µg vector at a 2.5:7.5:10 ratio). Then, the two solutions were mixed, incubated for 25 min, and added to HEK293T cells cultured in a 10-cm cell culture dish at 70% confluence together with 4 mL Opti-MEM. After 4–6 h, the medium was changed to normal DMEM, and the supernatant was collected thrice every 24 h. PEG-8000 solution was filtered using a 0.45-μm filter, added to the supernatant to a final concentration of 0.1 g/mL, and incubated at 4 °C overnight. The solution was centrifuged at 4200 rpm for 30 min, and the supernatant was discarded. The lentivirus was resuspended in 1× PBS and stored at –80 °C.

### Cell counting kit-8 (CCK-8) assay

The cells (2000/well) were seeded on a 96-well transparent dish. The CCK-8 solution was added to the medium (1:100) and incubated at 37 °C for 4 h. Then, the absorbance was determined at 450 nm, and the cell number was derived from the standard curve.

### Next-generation sequencing data analysis

Clean data were obtained by processing RNA-seq raw data in fastq format using FastQC v0.23.2. The default parameters were used to trim reads containing adapters and filter low-quality reads and reads containing N bases. Then, the clean data were aligned to GRCm39/GRCh38 (UCSC) genome assemblies using STAR v.2.7.10a with default parameters. The uniquely mapped read pairs were counted using FeatureCounts v2.0.1. The output gene count matrix was quantile normalized using the R package DESeq2 v1.32.0. log transformation of the count matrix was performed for principal component analysis (PCA). The significance and fold change of DEGs between samples were estimated using the R package DEseq2, where an adjusted *P* value < 0.05 and absolute log2Foldchange value ≥ 1 were selected. KEGG and GO enrichment analysis of the DEGs were performed using clusterProfiler v4.6.0. The gene number and *P* value of the KEGG and GO terms were visualized using clusterProfiler v4.6.0. The heatmaps and bar plots of significant DEGs were constructed using heatmap v1.0.12 and ggplot2 v3.4.0, respectively.

### Embryo immunofluorescence analysis

The embryos were washed in PBS containing 3 mg/mL polyvinylpyrrolidone (PVP-PBS, Sigma), fixed in 4% PFA, then permeabilized with 0.25% Triton X-100 in PVP-PBS for 1 h. The samples were blocked by blocking buffer (0.2% BSA, 0.25% Triton X-100, 0.01% Tween 20, and 2% donkey serum in PVP-PBS) after washing. Then, the embryos were incubated with primary antibody diluted in blocking buffer at 4 °C overnight and in secondary antibody for 1 h at room temperature. The embryos were rinsed in PVP-PBS four times at 5 min per rinse. Then, the nuclei were stained with DAPI for 15 min. High-resolution images were captured by an Olympus FV3000 fluorescence microscope at ×60 magnification with an oil immersion objective. Z-sections were captured every 0.5 μm.

### Histological analysis of tissues

The tissue samples were fixed in 4% PFA overnight and dehydrated with 70% ethanol for at least 24 h. Then, the samples were embedded in paraffin and sliced before undergoing HE staining. The paraffin-embedded ovaries were sectioned at a thickness of 8 mm for hematoxylin and eosin (H&E) staining.

### Open field test

The locomotor activity of mice was assessed using an Open Field Test conducted within a white Plexiglas box measuring 40 cm in length, width, and height. Mice were positioned at the center of the box floor and given 10 min to explore freely, with their movements recorded via a camera. The total distance traveled by the mice and their trajectories were analyzed using AlsVision software (AniLab Scientific Instruments Co., Ltd., China).

### Mouse genotyping

The tail tips or toes were cut off from seven-day-old mice and boiled in 200 μL 50 mM/mL NaOH at 95 °C for 45 min to lyse the tissue and release the genomic DNA. The DNA extraction solution was neutralized by 16.7 μL Tris-HCl (pH 8.0). The solution was centrifuged at 12,000 rpm for 5 min at 4 °C to obtain the precipitation. The supernatant was used for PCR detection. Table EV1 lists the primer used to determine the genome type. The PCR product underwent electrophoresis in 1× TAE buffer at 120 V for 30 min on 1.5% agarose gel. The gel was prepared using 1× TAE buffer supplemented with 10,000× Gel Red solution. A fluorescence image was captured under ultraviolet light (UV) by the Tanon 3500 Gel Image System.

### Survival rate analysis

The mouse survival rate was calculated by drawing a survivorship curve. The software used was GraphPad Prism 9.5.1, and the analysis method was the Kaplan–Meier method.

### 5-PT immunoprecipitation

The method of clicking chemistry mediated immunoprecipitation was described previously (Wang et al, 2024). In brief, serotonylated proteins were labeled by using copper click chemistry, Alkyne-functionalized 5-HT derivative 5-PT was conjugated to a biotin–azide molecule and prepared in MedChemExpress (MCE®), cells were treated with 10 µM 5-PT for 72 h. Cell pellets were sonicated in 100 µL CLICK reaction buffer (Thermo, C10643), then centrifuged at 15,000 rpm *15 min at 4 °C, supernatant was then transferred to a new 1.5 ml EP tube. Adding the following order, 0.25 mM biotin azide, 0.1 mM CuSO_4_, and 50 μL of 1× CLICK additive to the lysates, and the final volumes were increased to ~500 μL with CLICK reaction buffer. Samples were incubated for 2–3 h at 4 °C. Magnetic streptavidin beads (Thermo Fisher, 11206D) were washed twice in PBS. The reaction volume was then increased to 1 mL with PBS, with 5% removed for input before the addition of washed beads. For each immunoprecipitation, 20 μL of bead slurry was used, and samples were rotated at room temperature for 3 h. After incubation, samples were placed on a magnetic stand to allow beads to separate from the supernatants which were carefully removed. The beads were then washed in cold RIPA buffer (10 mM Tris-Cl, pH 8.0, 1 mM EDTA, 1% Triton X-100, 0.1% sodium deoxycholate, 0.1% SDS, 150 mM NaCl, 1 mM PMSF) containing protease inhibitors for three times, followed by three times washes in cold DPBS to remove excess detergent. After the last wash, beads were added with 2*SDS and boiled for 8 min at 98 °C, followed by gel electrophoresis and incubation with appropriate primary and secondary antibodies.

### For LC-MS/MS analysis of 5-PT immunoprecipitation

#### Protein extraction

The sample was taken out at −80 °C, and an appropriate amount of enzymatic hydrolysis solution (100 mM ammonium bicarbonate) was added. Then thorough vortexing, a small amount of magnetic bead suspension was taken and added to the loading buffer. After heating at 95 °C for 15 min, silver staining was performed for detection. The final protein concentration was obtained by comparing the silver-staining gel chart with the HeLa standard. The protein amount is for reference only and should be based on the peptide amount after enzymatic hydrolysis.

#### Trypsin digestion

For digestion, each sample was subjected to on-beads enzyme digestion, and a final concentration of 20 ng/µL trypsin for the first digestion overnight. Then, the on beads protein solution was digested by reduced by 5 mM dithiothreitol for 60 min at 37 °C and alkylated with 11 mM iodoacetamide for 45 min at room temperature in darkness. Add a final concentration of 10 ng/μL trypsin for a second 4 h-digestion. Finally, the peptides were desalted by Strata X SPE column.

#### LS-MS analysis

The tryptic peptides were dissolved in solvent A, directly loaded onto a home-made reversed-phase analytical column (25-cm length, 100 μm i.d.). Home-made reversed-phase analytical column (100 μm i.d. × 25 cm) packed with 1.9 μm/120 Å ReproSil-PurC18 resins (Dr. Maisch GmbH, Ammerbuch, Germany). The mobile phase consisted of solvent A (0.1% formic acid, 2% acetonitrile/in water) and solvent B (0.1% formic acid, 90% acetonitrile/in water). Peptides were separated with the following gradient: 0–22.5 min, 6%–22%B；22.5–26.5 min, 22%–34%B；26.5–28.5 min, 34%–80%B；28.5–30 min, 80%B, and all at a constant flow rate of 700 nL/min on a EASY-nLC 1200 UPLC system (ThermoFisher Scientific).

The separated peptides were analyzed in Orbitrap Exploris 480 with a nano-electrospray ion source. The electrospray voltage applied was 2300 V. FAIMS compensate voltage (CV) was set as 45 V. Precursors and fragments were analyzed at the Orbitrap detector. The full MS scan resolution was set to 60,000 for a scan range of 350–1400 *m/z*. The MS/MS scan was fixed first mass as 120.0 *m/z* at a resolution of 15,000. The HCD fragmentation was performed at a normalized collision energy (NCE) of 27%. The automatic gain control (AGC) target was set at 1E6, with a maximum injection time of 22 m.

#### Database search

The DIA data was processed using DIA-NN search engine (v.1.8). Tandem mass spectra were searched against the Homo_sapiens_9606_SP_20231220.fasta (20429 entries) concatenated with reverse decoy database. Trypsin/P was specified as a cleavage enzyme allowing up to 1 missing cleavage. Excision on N-term Met and carbamidomethyl on Cys were specified as fixed modifications. FDR was adjusted to <1%.

### In vitro enzymatic serotonylation assays

The enzymatic assay was adapted according to the previously reported (Wang et al, 2024). Briefly, 5-HT (5 mmol/L) was transamidated to HSP90β (Abcam, ab80033) protein by 0.25 µg functional TGM2 protein (MCE, HY-P76114), in a final volume of 25 µL enzymatic buffer (25 mM Tris-HCl, pH 8 and 5 mM CaCl_2_, with protease inhibitors). The reaction buffer was incubated at 37 °C for 3 h in the dark, samples were boiled with 2* SDS for 10 min at 95 °C, serotonylated proteins were visualized by Coomassie staining, followed by LC-MS/MS analyses of serotonylation.

### LC-MS/MS analysis for in vitro enzymatic serotonylation assays

#### Sample preparation

The gel is cut into small pieces and decolorized using a solution of 25% acetonitrile (ACN) and 50 mM ammonium bicarbonate (ABC). Next, the gel pieces are dehydrated with ACN. The proteins are reduced by adding 5 mM dithiothreitol (DTT) and incubating at 37 °C for 1 h, followed by another dehydration step. Then, alkylation is performed with 11 mM iodoacetamide (IAM) for 45 min at room temperature in the dark.

After dehydration, 200 µL of trypsin solution (at a concentration of 10 ng/µL) is added and digestion is performed overnight. Subsequently, the peptides are eluted once with 150 µL of Elution Buffer I (50% ACN, 5% formic acid), followed by a single elution with 150 µL of Elution Buffer II (75% ACN, 0.1% formic acid), and then eluted twice with 150 µL of Elution Buffer III (100% ACN). The elutions are combined and dried down, then Stage-Tip desalting is performed. Finally, the peptide fragments are dissolved in 0.1% formic acid for analysis.

#### Tandem MS

MS experiments were performed on Q Exactive HF-X instrument (ThermoFisher) coupled with an Easy-nLC 1200 system. Mobile phases A and B were water and 80% acetonitrile, respectively, with 0.1% formic acid. Digested peptides were loaded directly onto an analytical column (75 μm × 15 cm, 1.9 μm C18, 5 μm tip) at a flow rate of 300 nL min–1. All peptides samples were separated using a linear gradient of 4–6% phase B for 1 min, 6–26% B for 42 min, 26–35% B in 12 min, 35%–90% B for 2 min and 90% B for 3 min. Survey scans of peptide precursors were performed from 400 to 1400 *m/z* at 60,000 full width at half-maximum resolution with a 3 × 10^6^ ion count target and a maximum injection time of 45 ms. The instrument was set to run in top-speed mode with 1-s cycles for the survey and the MS/MS scans. After a survey scan, tandem MS was then performed on the most abundant precursors exhibiting a charge state from 2 to 7 of greater than 1 × 10^5^ intensity by isolating them in the quadrupole mode at 1.6 *m/z*. Higher energy collisional dissociation fragmentation was applied with 27% collision energy and resulting fragments were detected in the Orbitrap detector at a resolution of 15,000. The maximum injection time limit was 22 ms and dynamic exclusion was set to 30 s with a 10-ppm mass tolerance around the precursor. Mass spectrometry data were searched by PD (ThermoFisher).

### Plasmids and cell lines

The plasmid for HSP90β was purchased from Fenghui Biotechnology Co., LTD. The LV3 lentiviral shRNA constructs against HSP90β with GFP marker was purchased from Jima Gene. The sequence of shRNA used in this study is listed in the Table EV1.

PCDH-HSP90β or shRNA against HSP90β was transfected into 293T cells for virus production, then cells were infected with the virus of PCDH-HSP90β or shRNA against HSP90, respectively. Forty-eight hours later, puromycin selection was performed.

### The ATPase activity of HSP90β

The HSP90β N-Terminal Assay Kit was purchased from BPS Bioscience (Cat #50294). Before the detection, we first conducted an in vitro protein recombination experiment. Samples were prepared by incubating the TDM2 protein with or without 5-HT. Subsequently, according to the instruction manual, the samples were added to the 96-well plate provided with the kit, and the detection was carried out on the Multifunctional microplate reader (TECAN).

### Statistical analysis

All statistical analyses were performed using GraphPad Prism 9.5.1 and R packages including DESeq2 and clusterProfiler. Unless otherwise indicated, data are presented as mean ± SD. For comparison between two groups, two-tailed unpaired *t* tests were used. For experiments involving more than two groups or multiple conditions, one-way ANOVA or two-way ANOVA was applied as appropriate, followed by Tukey’s post‑hoc test to determine statistical significance between groups. For cell proliferation assays (CCK-8), time-course data were analyzed using two-way ANOVA with repeated measures. Survival curves were analyzed using the Kaplan–Meier method with log-rank (Mantel–Cox) testing. Differential gene expression analysis was performed on RNA‑seq data using DESeq2, with adjusted *P* values calculated by the Benjamini–Hochberg false discovery rate (FDR) method; significance thresholds were set at adjusted *P* < 0.05 and |log2 fold change | ≥1. For metabolomics, principal component analysis and volcano plots were generated using normalized peak areas, and metabolite set enrichment was conducted through KEGG pathway analysis. All experiments were repeated independently at least three times unless specified otherwise. Statistical significance is indicated as *P < 0.05, **P < 0.01, and ***P < 0.001.

## Supplementary information

Table EV1

Peer Review File

Dataset EV1

Dataset EV2

Dataset EV3

Source data Fig. 1

Source data Fig. 2

Source data Fig. 3

Source data Fig. 4

Source data Fig. 5

Source data Fig. 6

Source data Fig. 7

Figure EV1 Source Data

Figure EV3 Source Data

Figure EV4 Source Data

Figure EV5 Source Data

Figure EV6 Source Data

Figure EV7 Source Data

Figure EV8 Source Data

Figure EV9 Source Data

Expanded View Figures

## Data Availability

The data that support the findings of this study are available in “Methods”, “Results”, and/or Supplemental Material of this article. RNA sequencing data have been deposited into the Gene Expression Omnibus (GEO) under the accession code GSE268560. The proteomic mass spectrometry data of serotonylation proteins in both in vivo, in vitro conditions and identification of HSP90AB1 serotonylation sites have been deposited into the China National Center for Bioinformation under the accession numbers OMIX011107, OMIX011121 and OMIX016492 (https://ngdc.cncb.ac.cn/omix: accession no.OMIX011107; https://ngdc.cncb.ac.cn/omix: accession no.OMIX011121; https://ngdc.cncb.ac.cn/omix: accession no.OMIX016492). The source data of this paper are collected in the following database record: biostudies:S-SCDT-10_1038-S44318-026-00832-x.
