# Supplementary figures and images for: The oocyte-enriched metabolite serotonin alleviates cellular senescence and aging phenotypes in the mouse (part 1 of 3)
Source: EMBO J. 2026 Jun 16;45(14):4849–86. doi: 10.1038/s44318-026-00832-x (PMC13373241; doi:10.1038/s44318-026-00832-x)

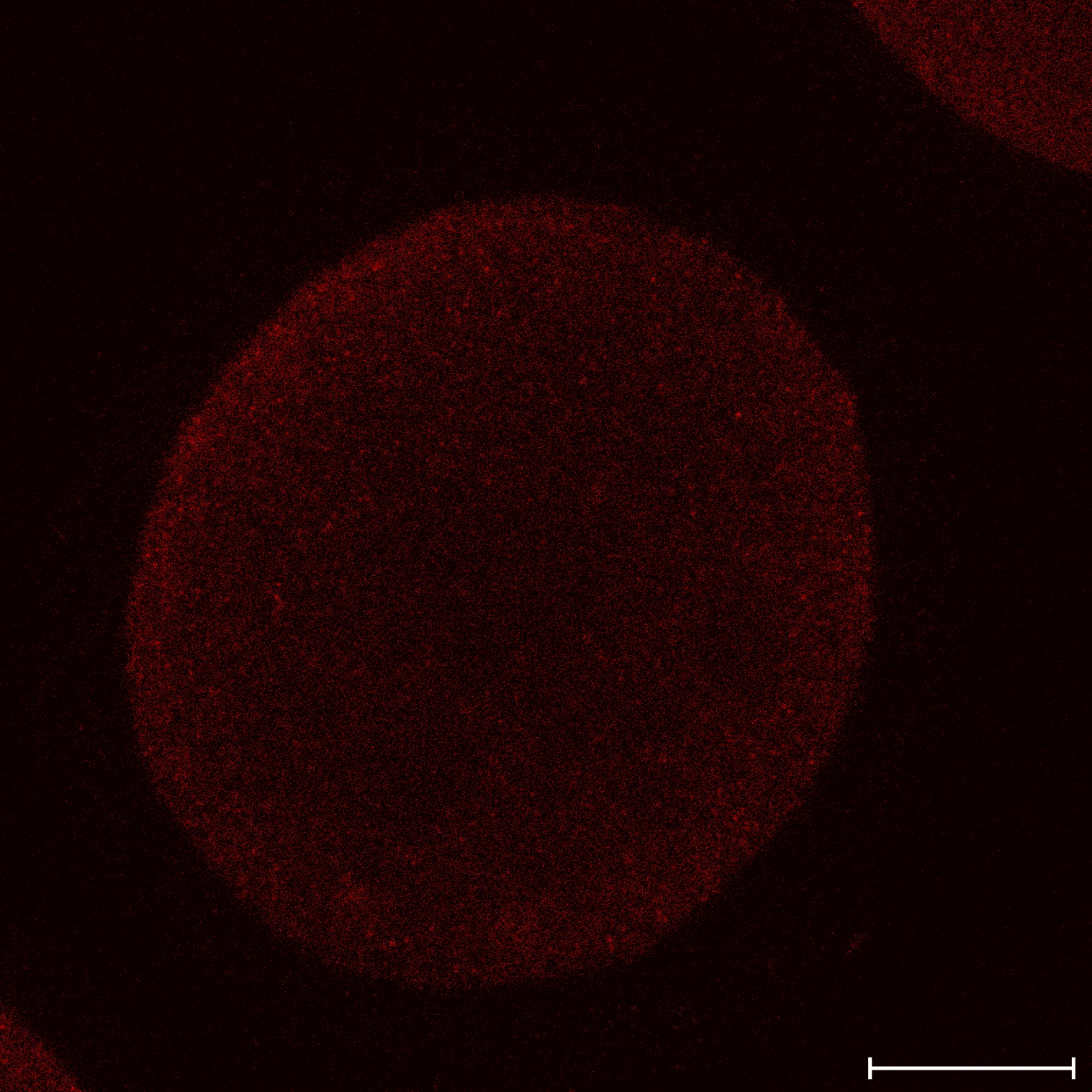

Supplement: Supplementary file 7 — Source data Fig. 2 [file 44318_2026_832_MOESM7_ESM.zip › E/old-5ht.jpg]

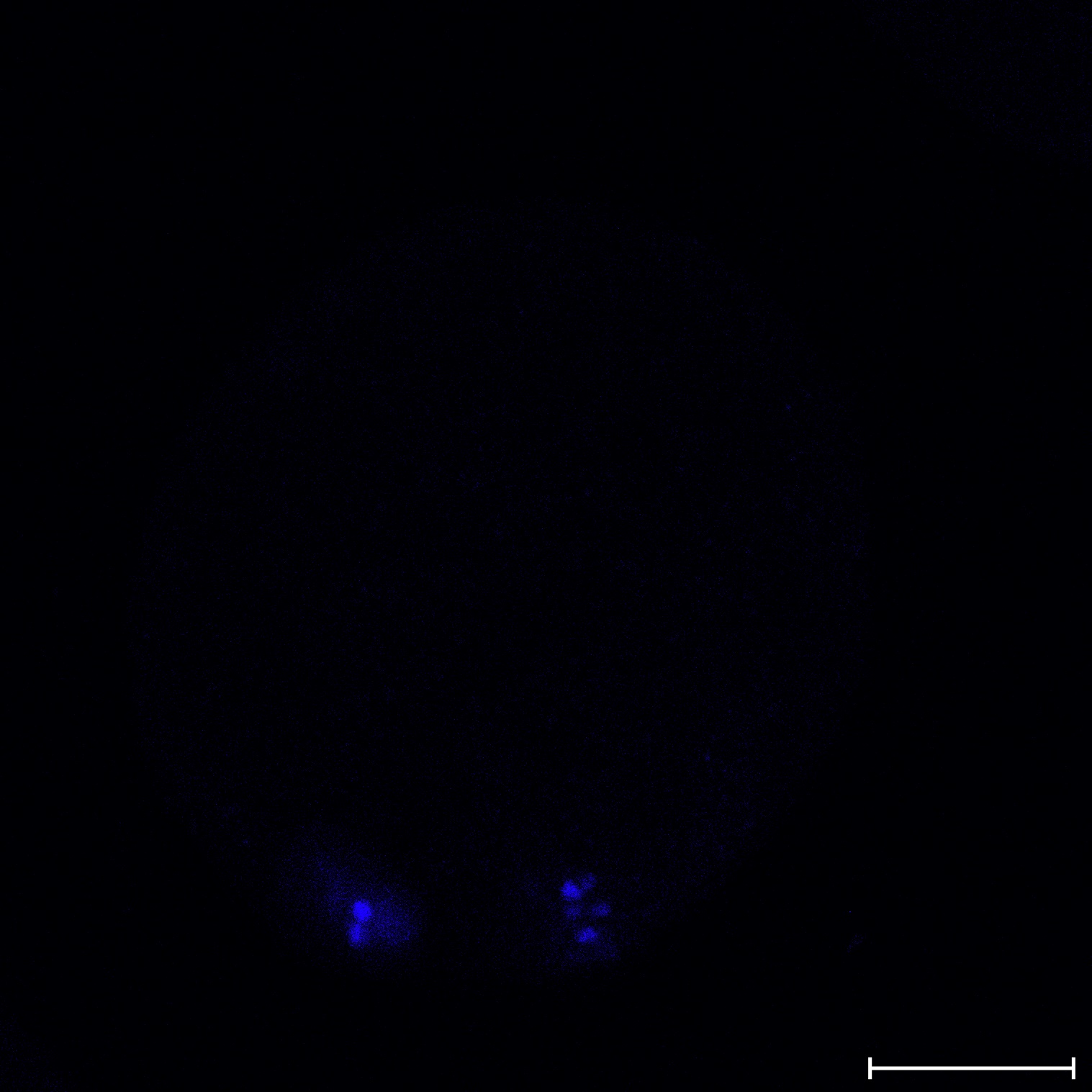

Supplement: Supplementary file 7 — Source data Fig. 2 [file 44318_2026_832_MOESM7_ESM.zip › E/old-dapi.jpg]

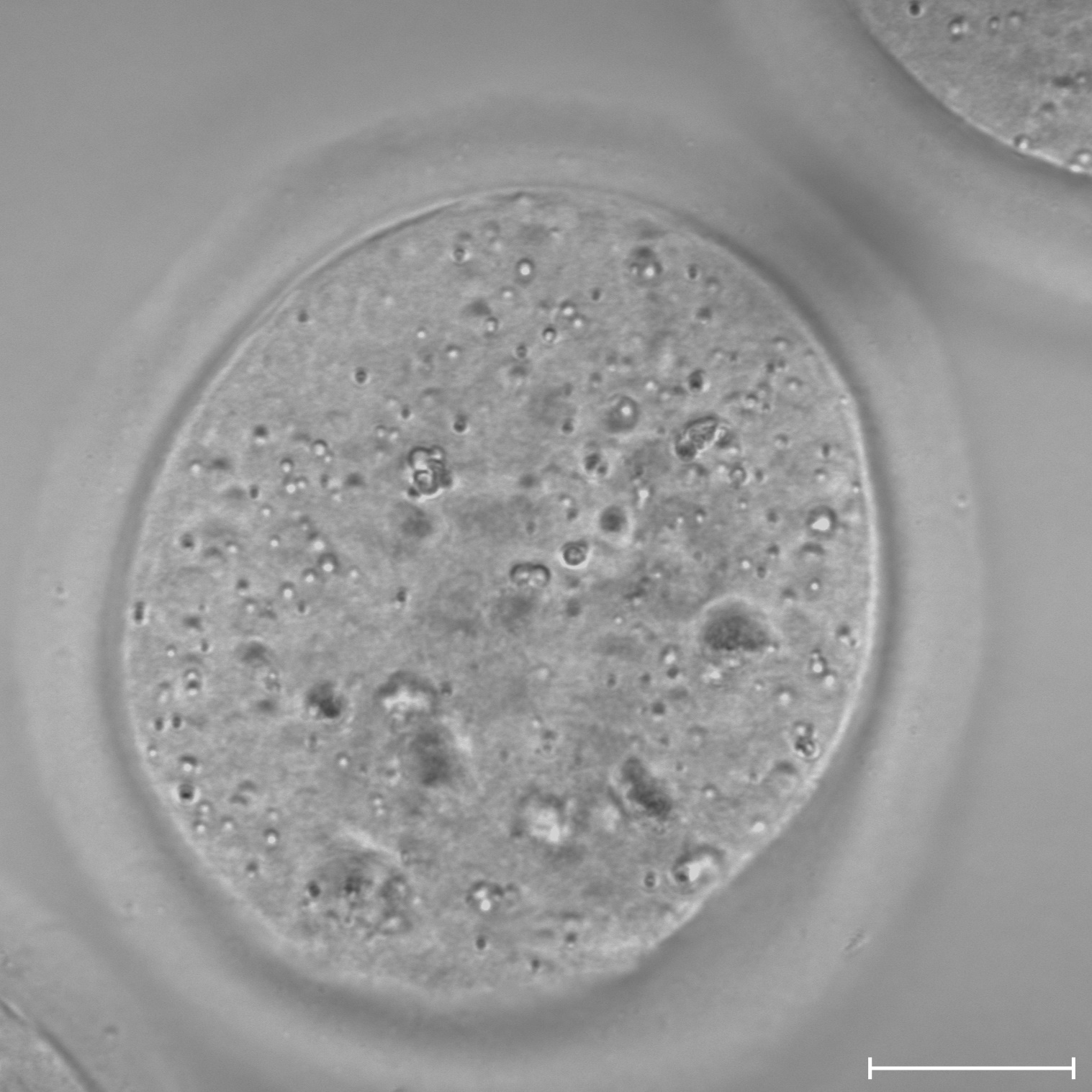

Supplement: Supplementary file 7 — Source data Fig. 2 [file 44318_2026_832_MOESM7_ESM.zip › E/old-light.jpg]

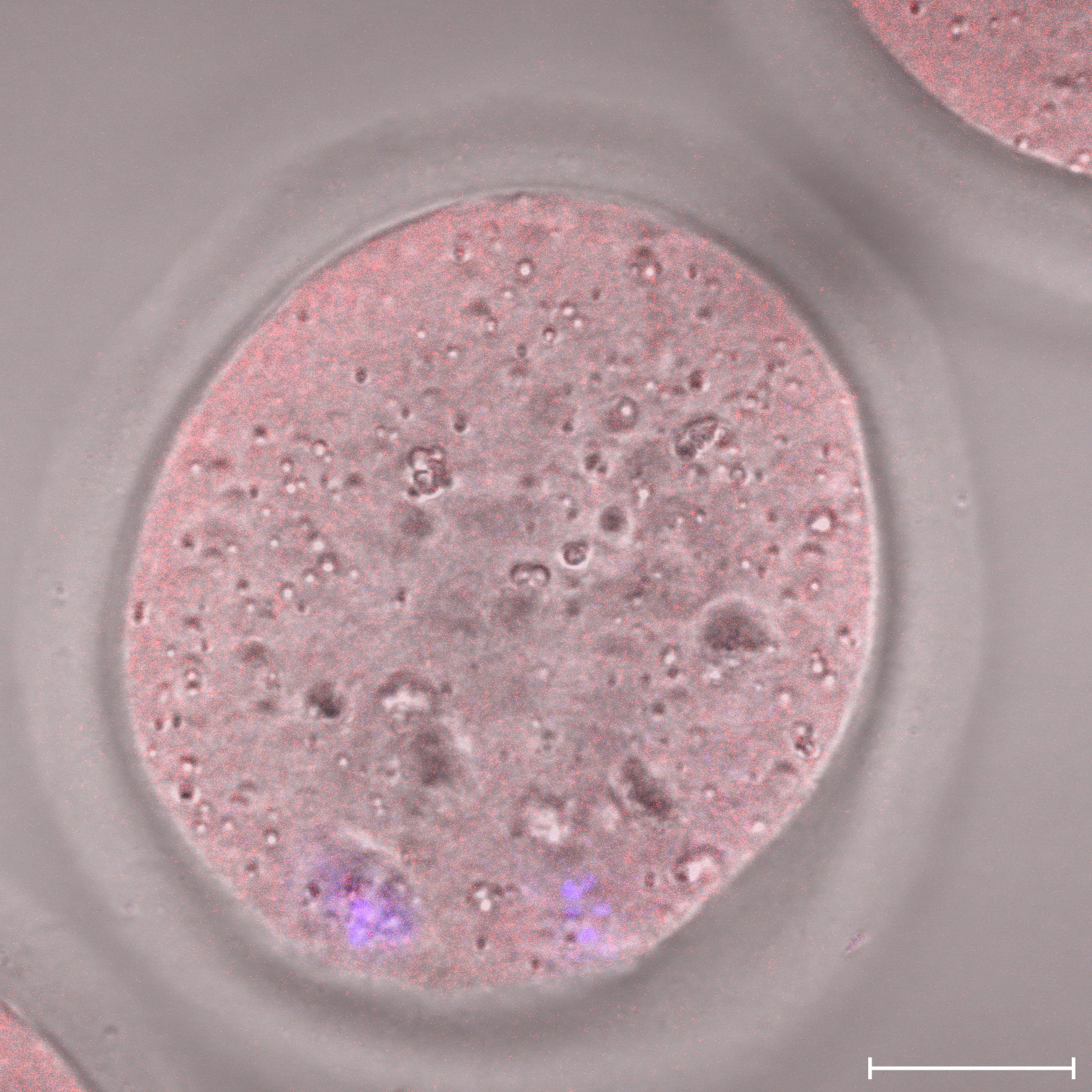

Supplement: Supplementary file 7 — Source data Fig. 2 [file 44318_2026_832_MOESM7_ESM.zip › E/old-merge.jpg]

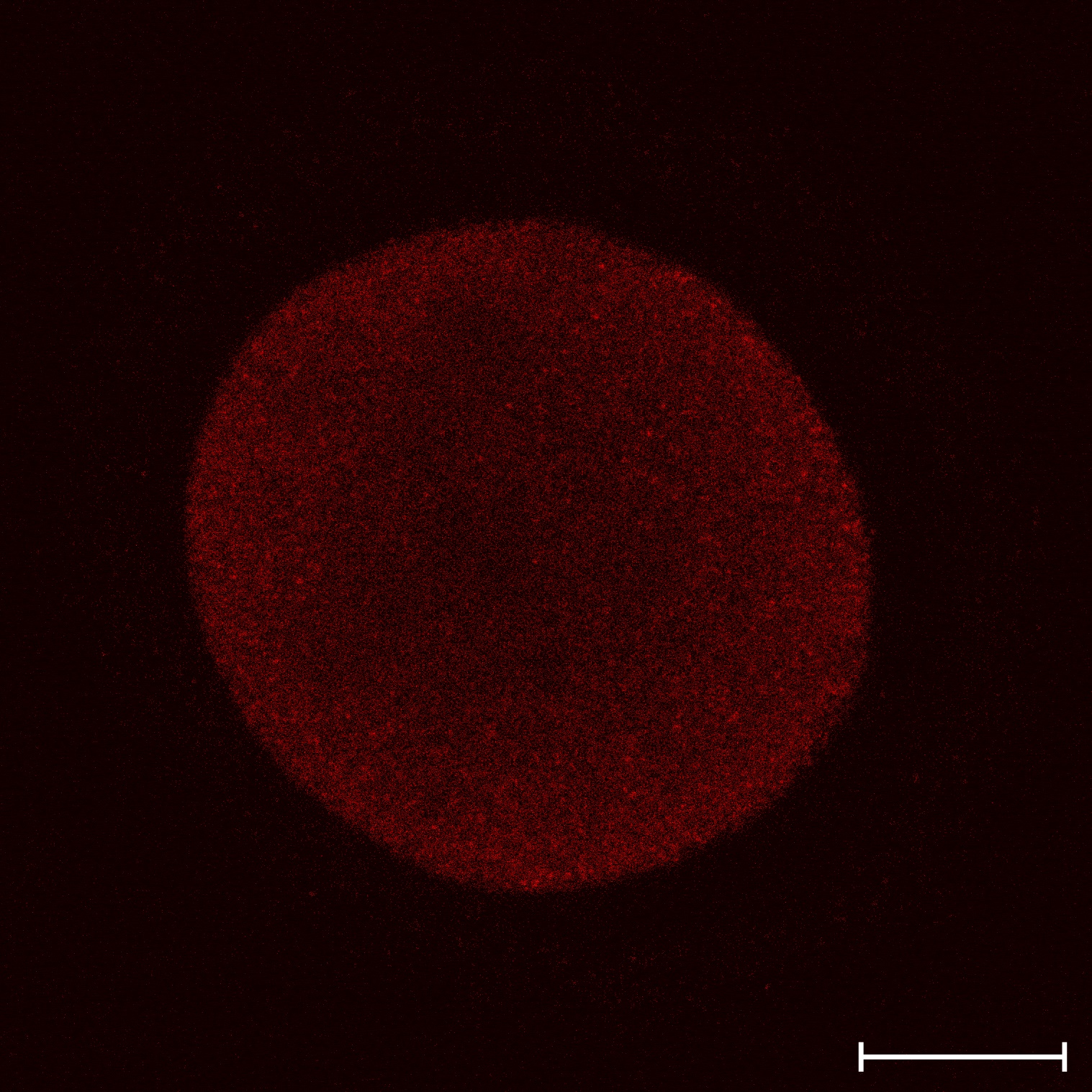

Supplement: Supplementary file 7 — Source data Fig. 2 [file 44318_2026_832_MOESM7_ESM.zip › E/young-5ht.jpg]

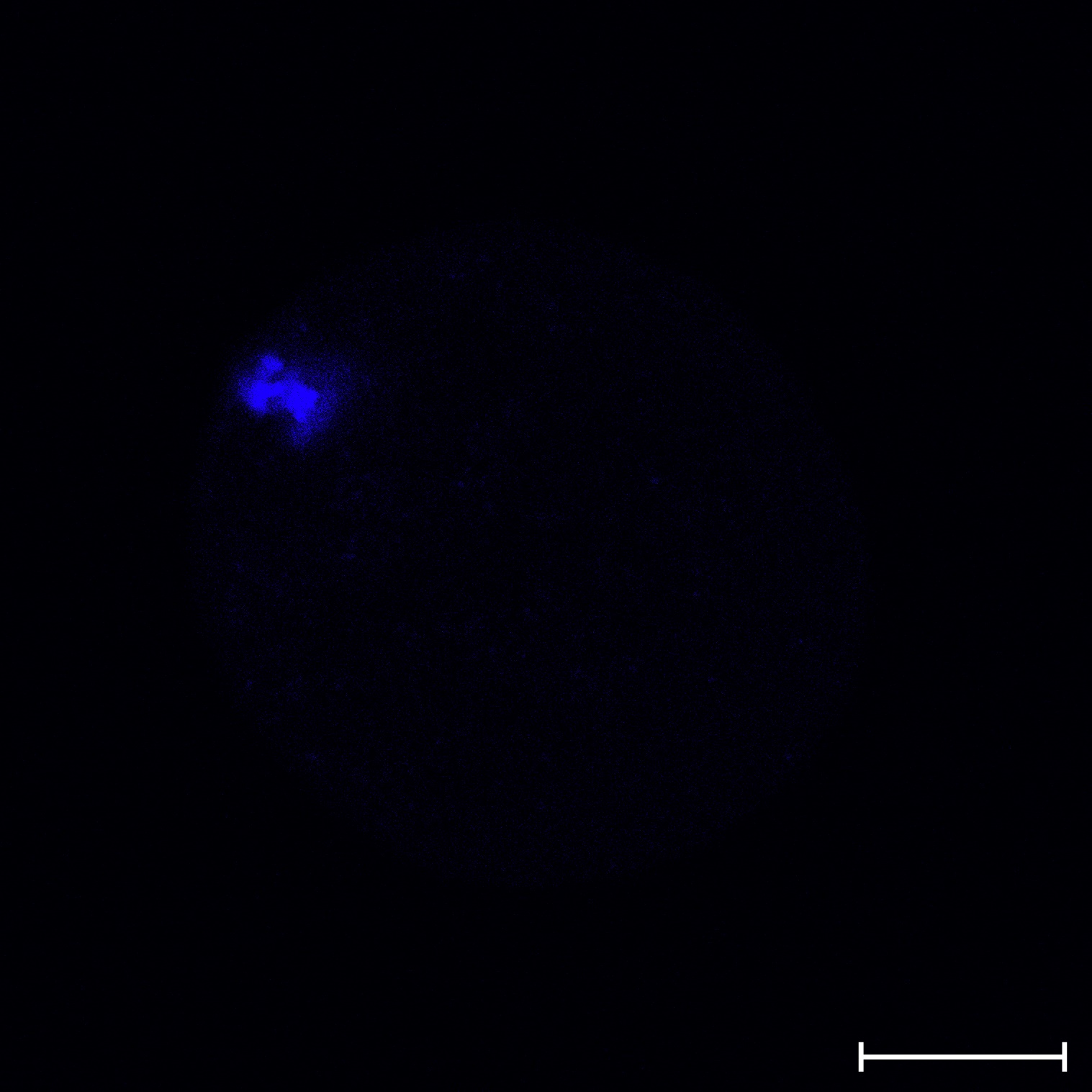

Supplement: Supplementary file 7 — Source data Fig. 2 [file 44318_2026_832_MOESM7_ESM.zip › E/young-dapi.jpg]

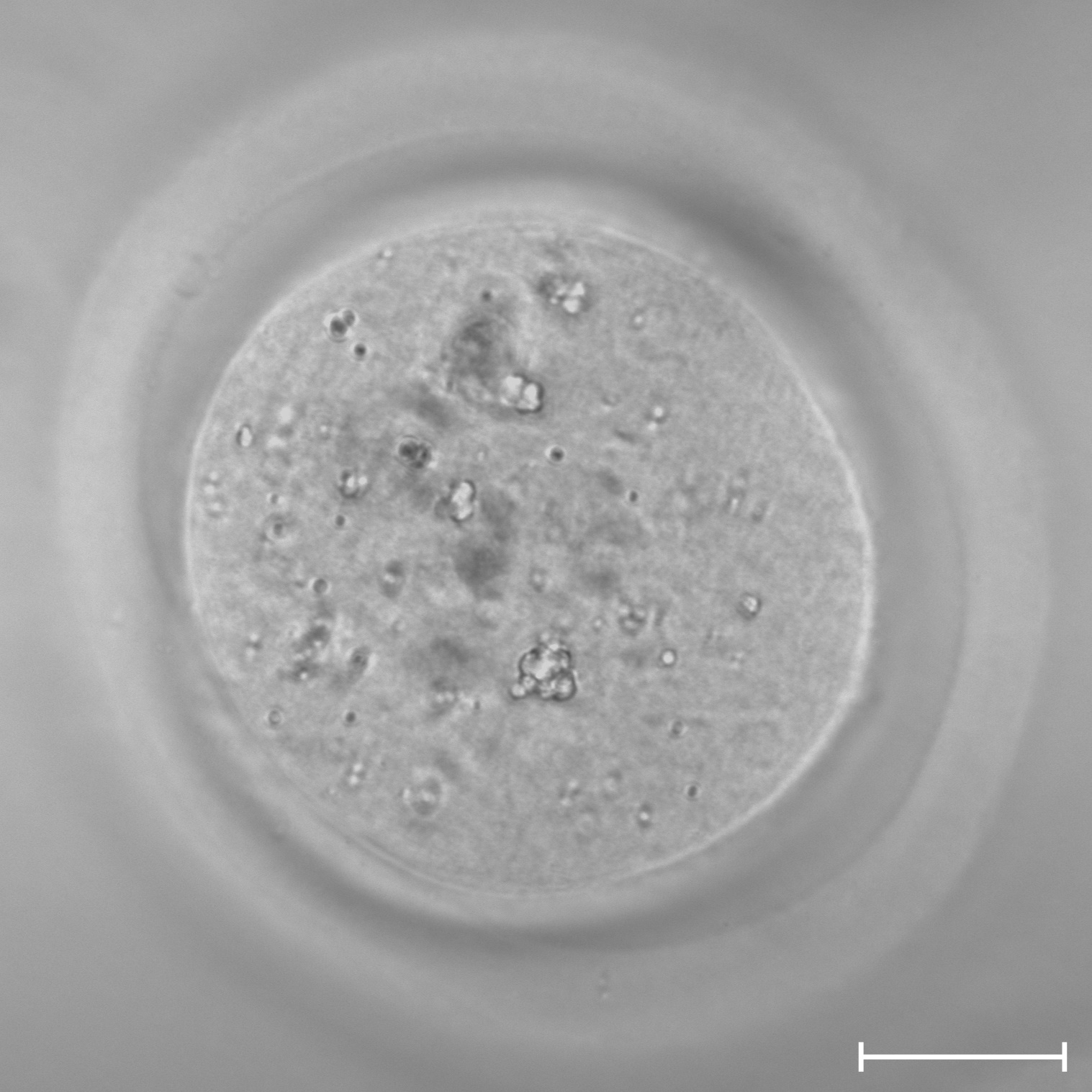

Supplement: Supplementary file 7 — Source data Fig. 2 [file 44318_2026_832_MOESM7_ESM.zip › E/young-light.jpg]

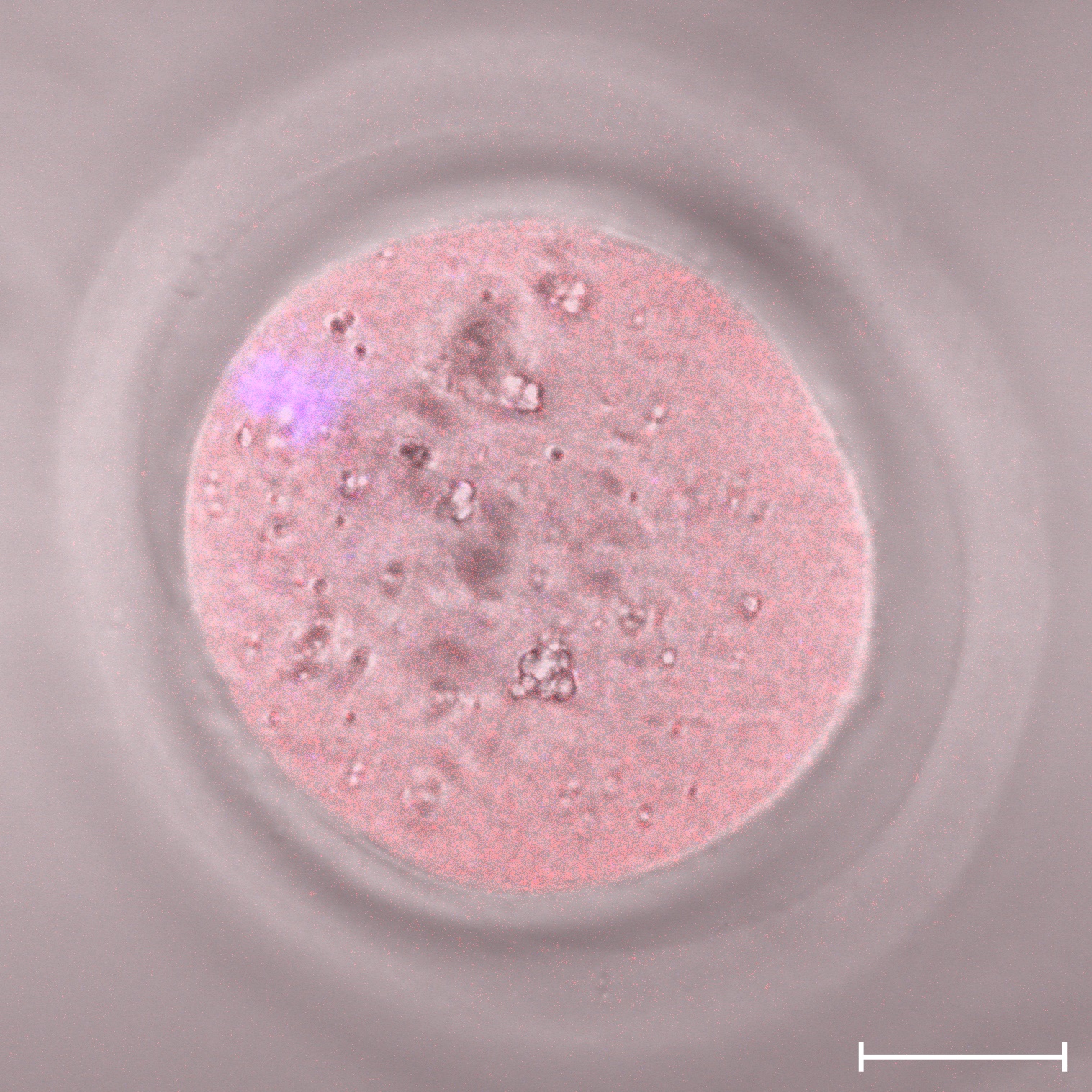

Supplement: Supplementary file 7 — Source data Fig. 2 [file 44318_2026_832_MOESM7_ESM.zip › E/young-merge.jpg]

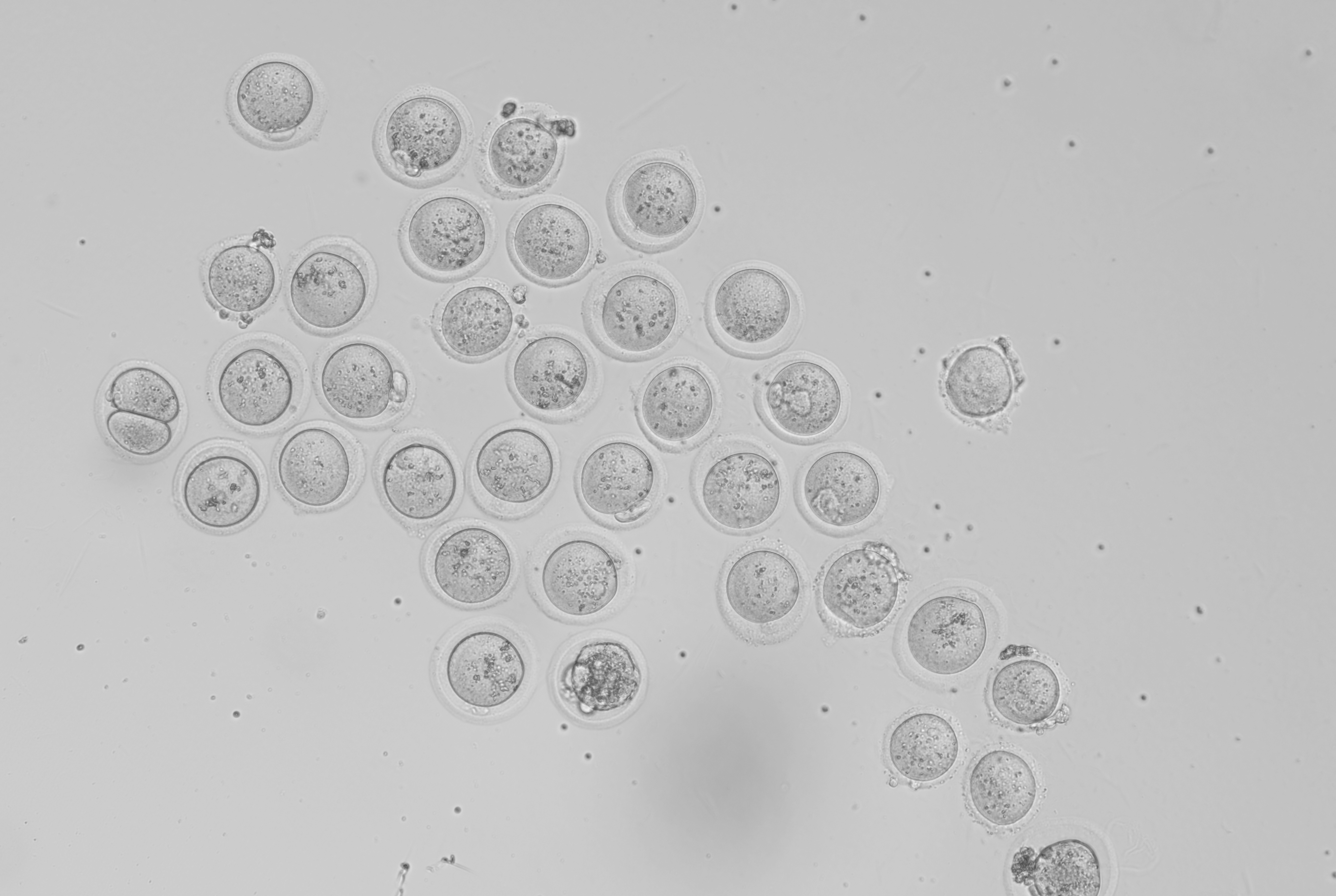

Supplement: Supplementary file 7 — Source data Fig. 2 [file 44318_2026_832_MOESM7_ESM.zip › G/12M+5-HT.tif]

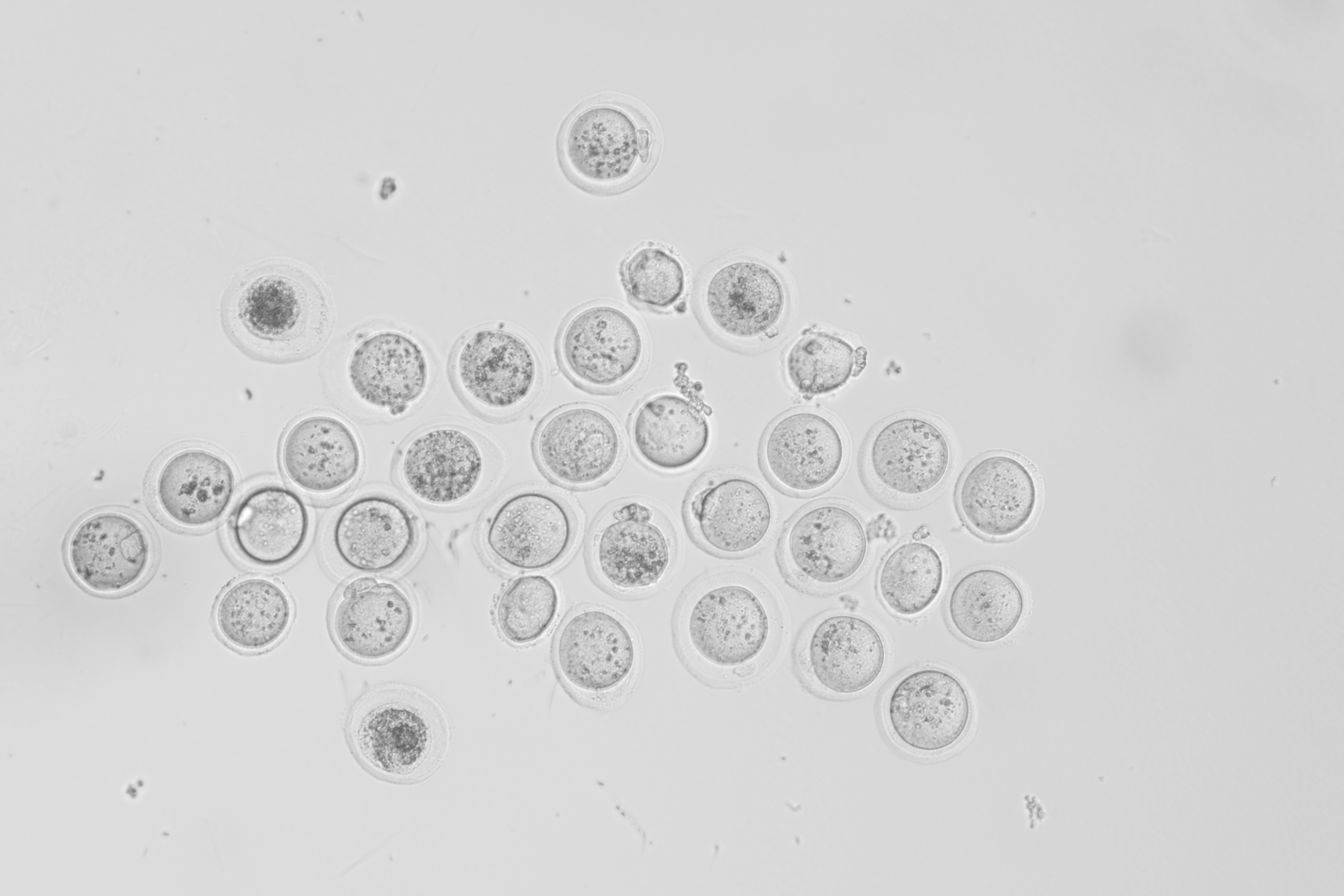

Supplement: Supplementary file 7 — Source data Fig. 2 [file 44318_2026_832_MOESM7_ESM.zip › G/12M.tif]

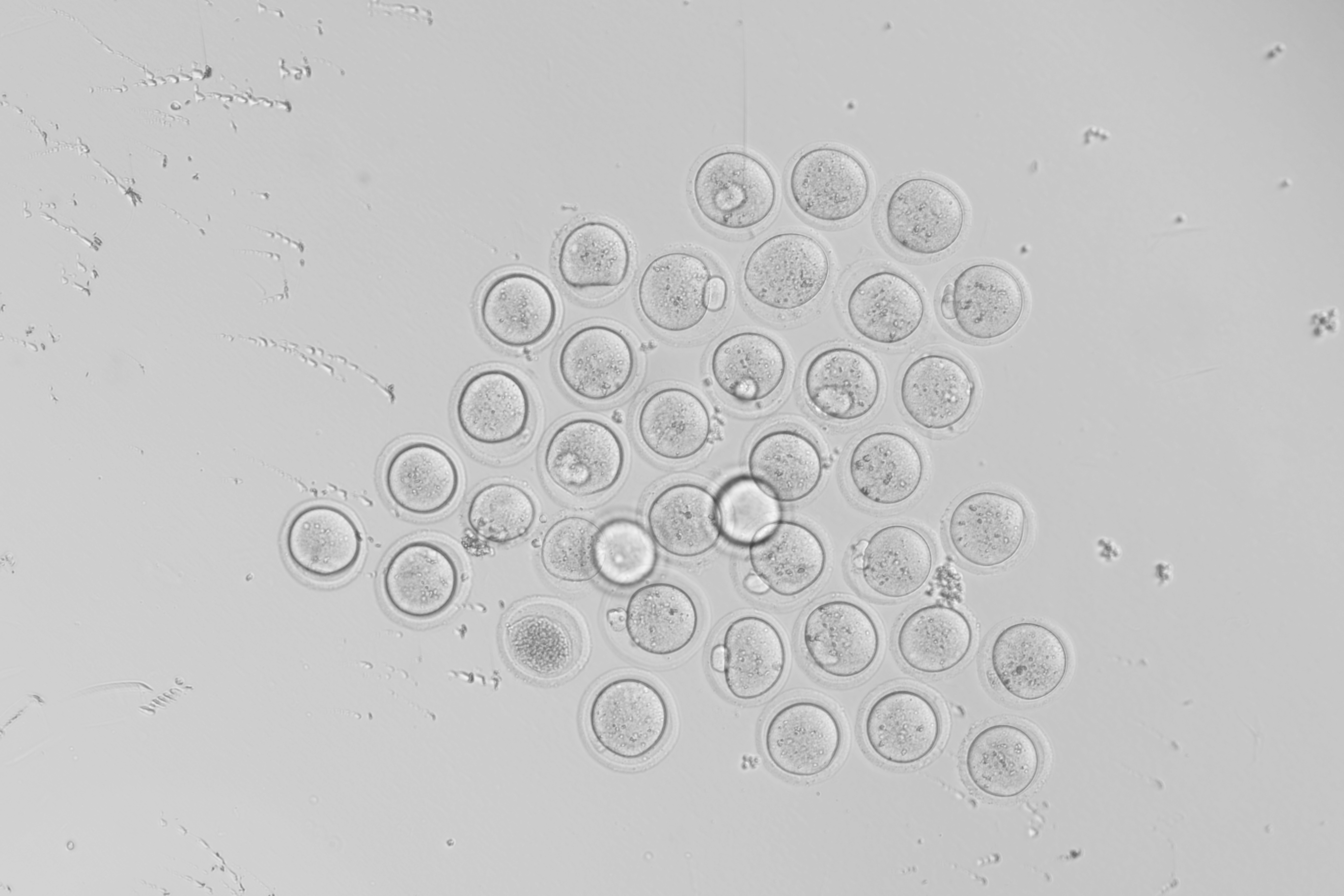

Supplement: Supplementary file 7 — Source data Fig. 2 [file 44318_2026_832_MOESM7_ESM.zip › G/4W.tif]

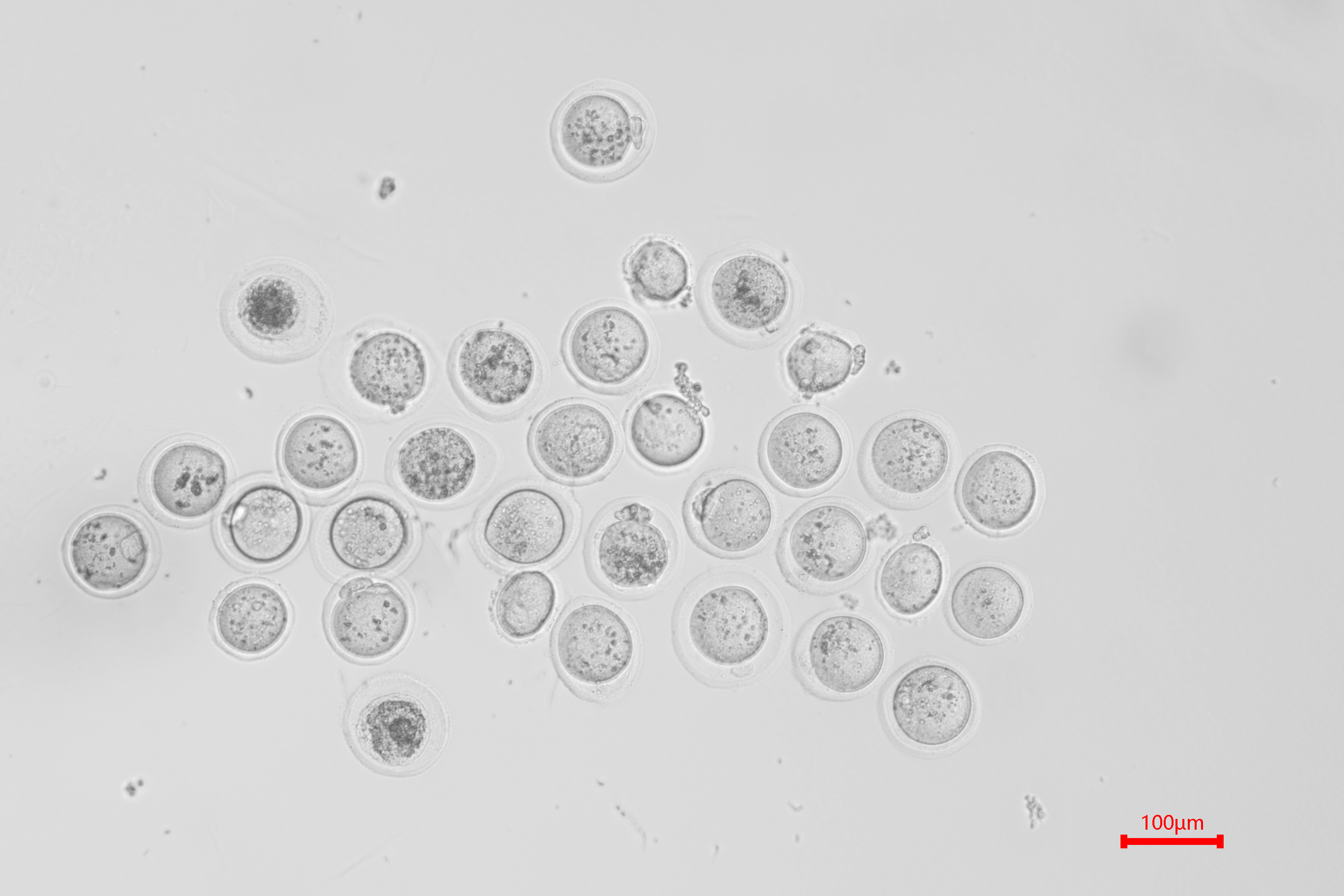

Supplement: Supplementary file 7 — Source data Fig. 2 [file 44318_2026_832_MOESM7_ESM.zip › G/scale bar.tif]

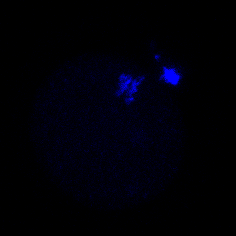

Supplement: Supplementary file 7 — Source data Fig. 2 [file 44318_2026_832_MOESM7_ESM.zip › I/12M+5ht-DAPI.tif]

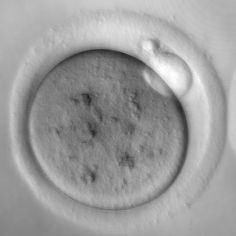

Supplement: Supplementary file 7 — Source data Fig. 2 [file 44318_2026_832_MOESM7_ESM.zip › I/12M+5ht-Light.tif]

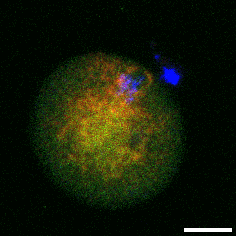

Supplement: Supplementary file 7 — Source data Fig. 2 [file 44318_2026_832_MOESM7_ESM.zip › I/12M+5ht-Merge.gif]

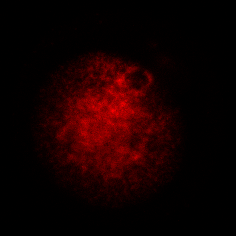

Supplement: Supplementary file 7 — Source data Fig. 2 [file 44318_2026_832_MOESM7_ESM.zip › I/12M+5ht-Mitotracker.tif]

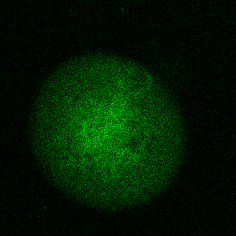

Supplement: Supplementary file 7 — Source data Fig. 2 [file 44318_2026_832_MOESM7_ESM.zip › I/12M+5ht-ROS.tif]

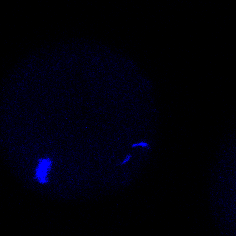

Supplement: Supplementary file 7 — Source data Fig. 2 [file 44318_2026_832_MOESM7_ESM.zip › I/12M-DAPI.tif]

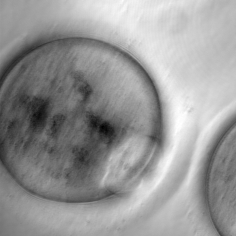

Supplement: Supplementary file 7 — Source data Fig. 2 [file 44318_2026_832_MOESM7_ESM.zip › I/12M-Light.tif]

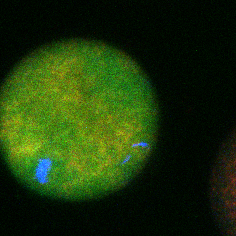

Supplement: Supplementary file 7 — Source data Fig. 2 [file 44318_2026_832_MOESM7_ESM.zip › I/12M-Merge.tif]

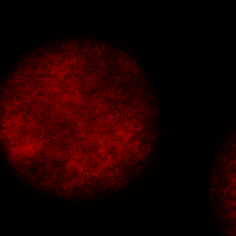

Supplement: Supplementary file 7 — Source data Fig. 2 [file 44318_2026_832_MOESM7_ESM.zip › I/12M-Mitotracker.tif]

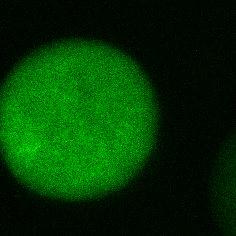

Supplement: Supplementary file 7 — Source data Fig. 2 [file 44318_2026_832_MOESM7_ESM.zip › I/12M-ROS.tif]

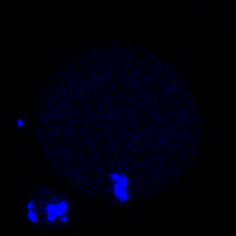

Supplement: Supplementary file 7 — Source data Fig. 2 [file 44318_2026_832_MOESM7_ESM.zip › I/4W-DAPI.tif]

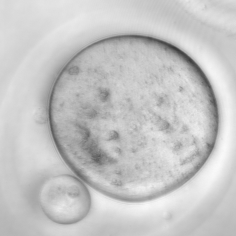

Supplement: Supplementary file 7 — Source data Fig. 2 [file 44318_2026_832_MOESM7_ESM.zip › I/4W-Light.tif]

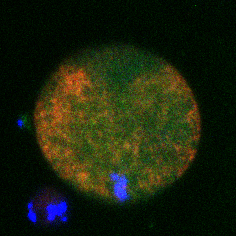

Supplement: Supplementary file 7 — Source data Fig. 2 [file 44318_2026_832_MOESM7_ESM.zip › I/4W-Merge.tif]

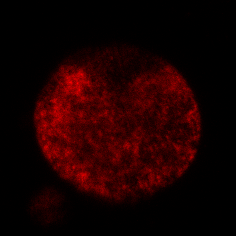

Supplement: Supplementary file 7 — Source data Fig. 2 [file 44318_2026_832_MOESM7_ESM.zip › I/4W-MItotracker.tif]

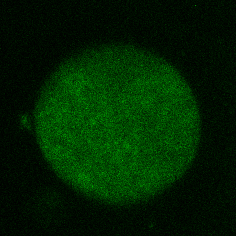

Supplement: Supplementary file 7 — Source data Fig. 2 [file 44318_2026_832_MOESM7_ESM.zip › I/4W-ROS.tif]

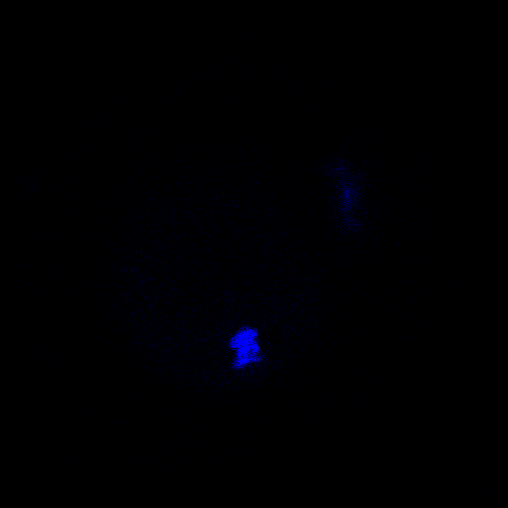

Supplement: Supplementary file 7 — Source data Fig. 2 [file 44318_2026_832_MOESM7_ESM.zip › L/12M+5-1.oir - C=0-1.tif]

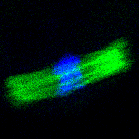

Supplement: Supplementary file 7 — Source data Fig. 2 [file 44318_2026_832_MOESM7_ESM.zip › L/12M+5ht-Large.tif]

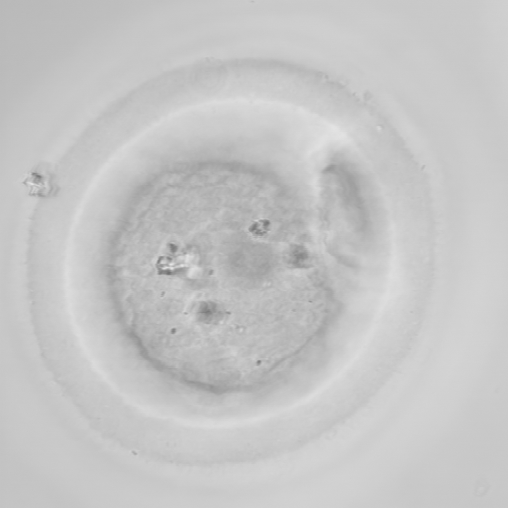

Supplement: Supplementary file 7 — Source data Fig. 2 [file 44318_2026_832_MOESM7_ESM.zip › L/12M+5ht-Light.tif]

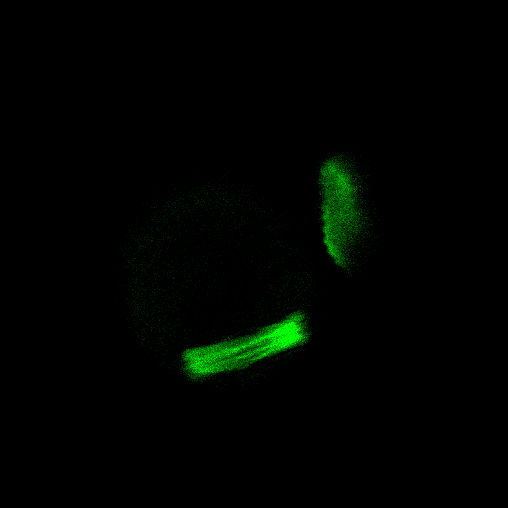

Supplement: Supplementary file 7 — Source data Fig. 2 [file 44318_2026_832_MOESM7_ESM.zip › L/12M+5ht-Tubulin.tif]

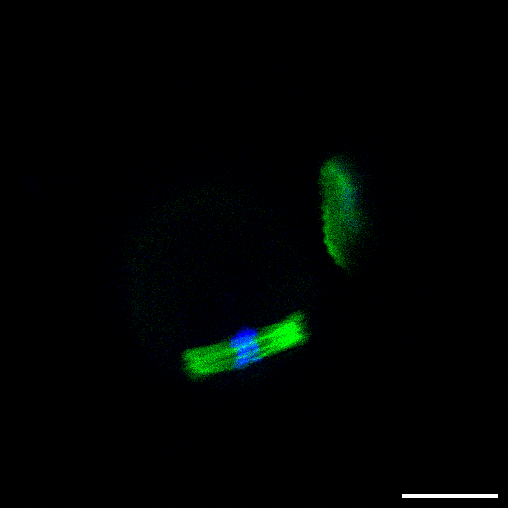

Supplement: Supplementary file 7 — Source data Fig. 2 [file 44318_2026_832_MOESM7_ESM.zip › L/12M+5ht.gif]

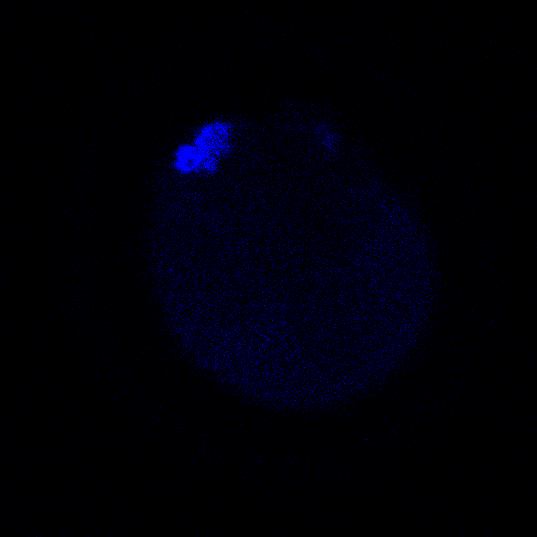

Supplement: Supplementary file 7 — Source data Fig. 2 [file 44318_2026_832_MOESM7_ESM.zip › L/12M-DAPI.tif]

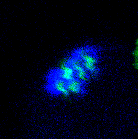

Supplement: Supplementary file 7 — Source data Fig. 2 [file 44318_2026_832_MOESM7_ESM.zip › L/12M-Large.tif]

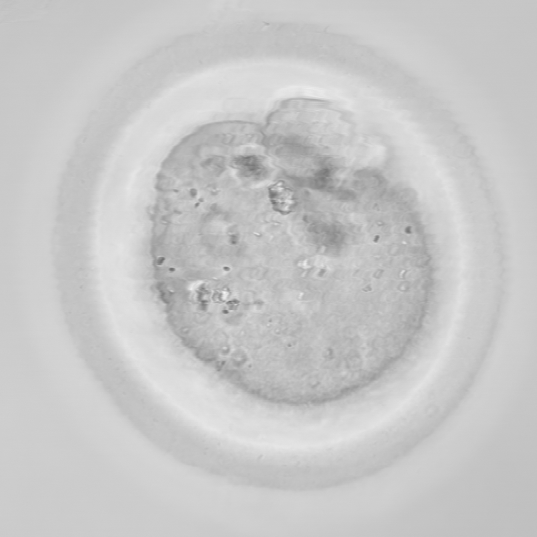

Supplement: Supplementary file 7 — Source data Fig. 2 [file 44318_2026_832_MOESM7_ESM.zip › L/12M-Light.tif]

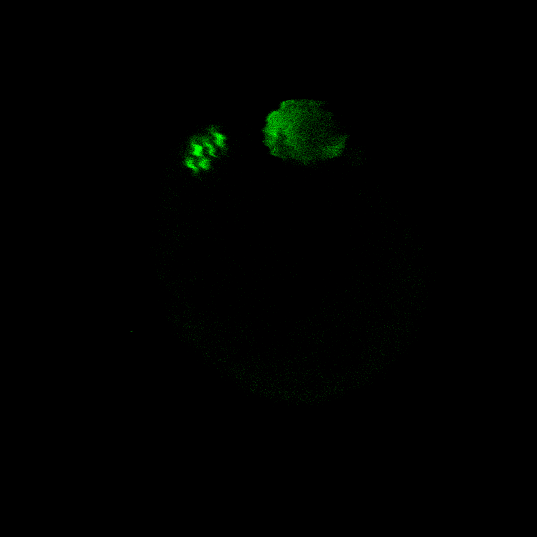

Supplement: Supplementary file 7 — Source data Fig. 2 [file 44318_2026_832_MOESM7_ESM.zip › L/12M-Tubulin.tif]

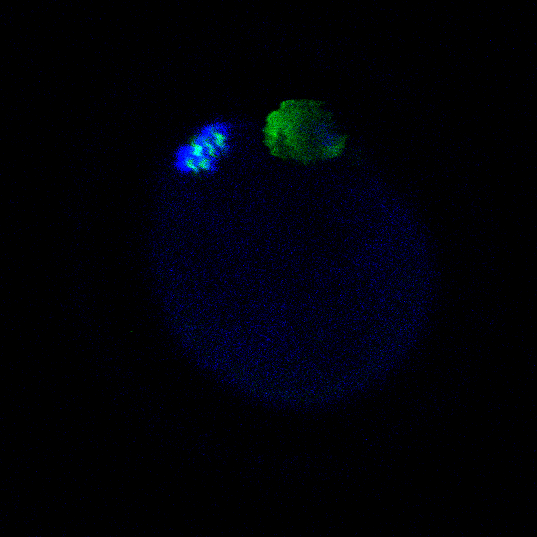

Supplement: Supplementary file 7 — Source data Fig. 2 [file 44318_2026_832_MOESM7_ESM.zip › L/12M.tif]

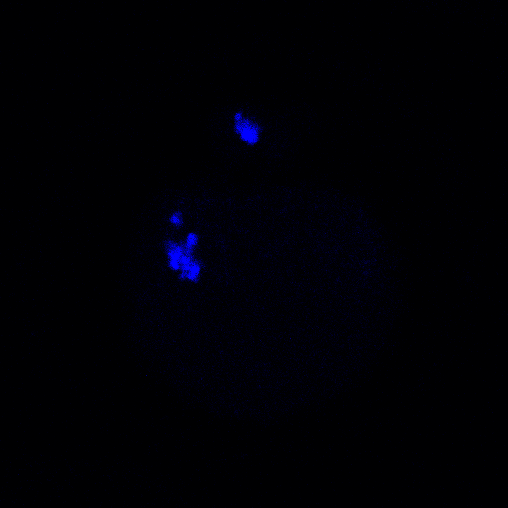

Supplement: Supplementary file 7 — Source data Fig. 2 [file 44318_2026_832_MOESM7_ESM.zip › L/4W-DAPI.tif]

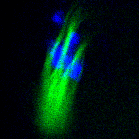

Supplement: Supplementary file 7 — Source data Fig. 2 [file 44318_2026_832_MOESM7_ESM.zip › L/4W-Large.tif]

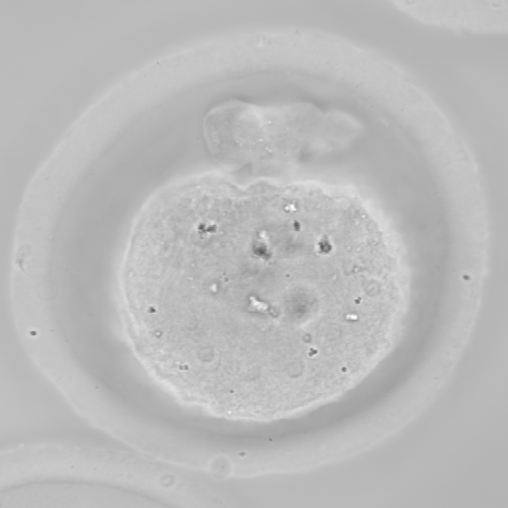

Supplement: Supplementary file 7 — Source data Fig. 2 [file 44318_2026_832_MOESM7_ESM.zip › L/4W-Light.tif]

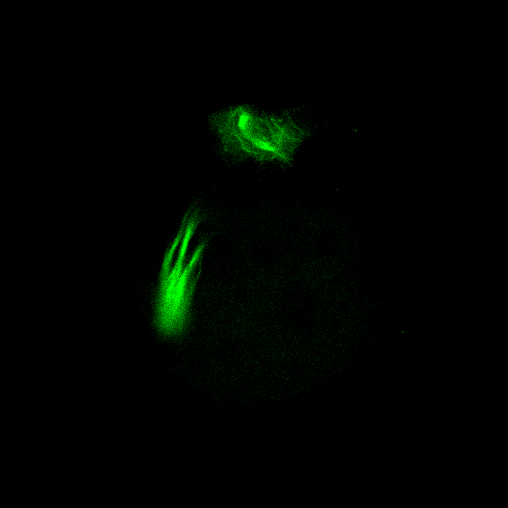

Supplement: Supplementary file 7 — Source data Fig. 2 [file 44318_2026_832_MOESM7_ESM.zip › L/4W-Tubulin.tif]

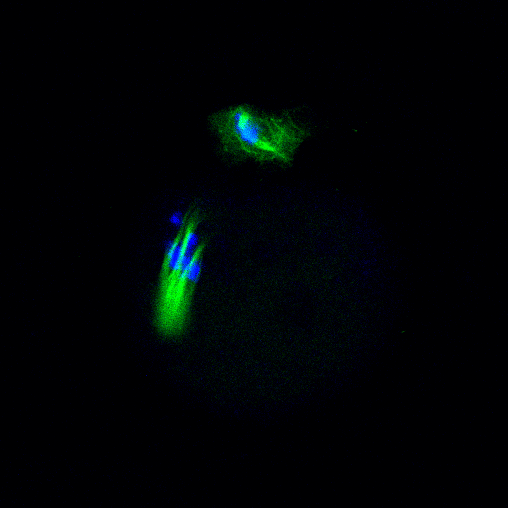

Supplement: Supplementary file 7 — Source data Fig. 2 [file 44318_2026_832_MOESM7_ESM.zip › L/4W.tif]

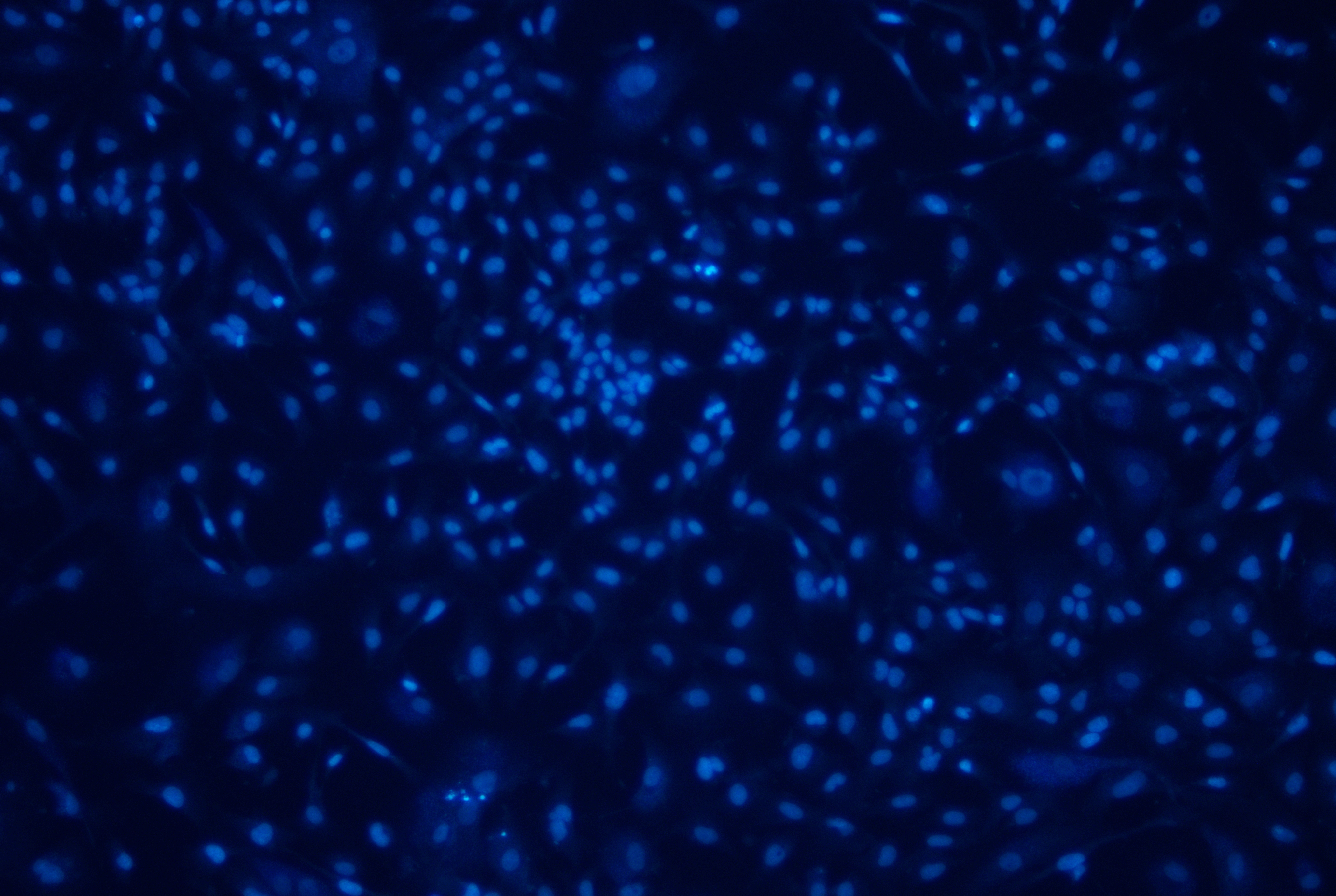

Supplement: Supplementary file 8 — Source data Fig. 3 [file 44318_2026_832_MOESM8_ESM.zip › A/G608G MSC+5HT-DAPI.jpg]

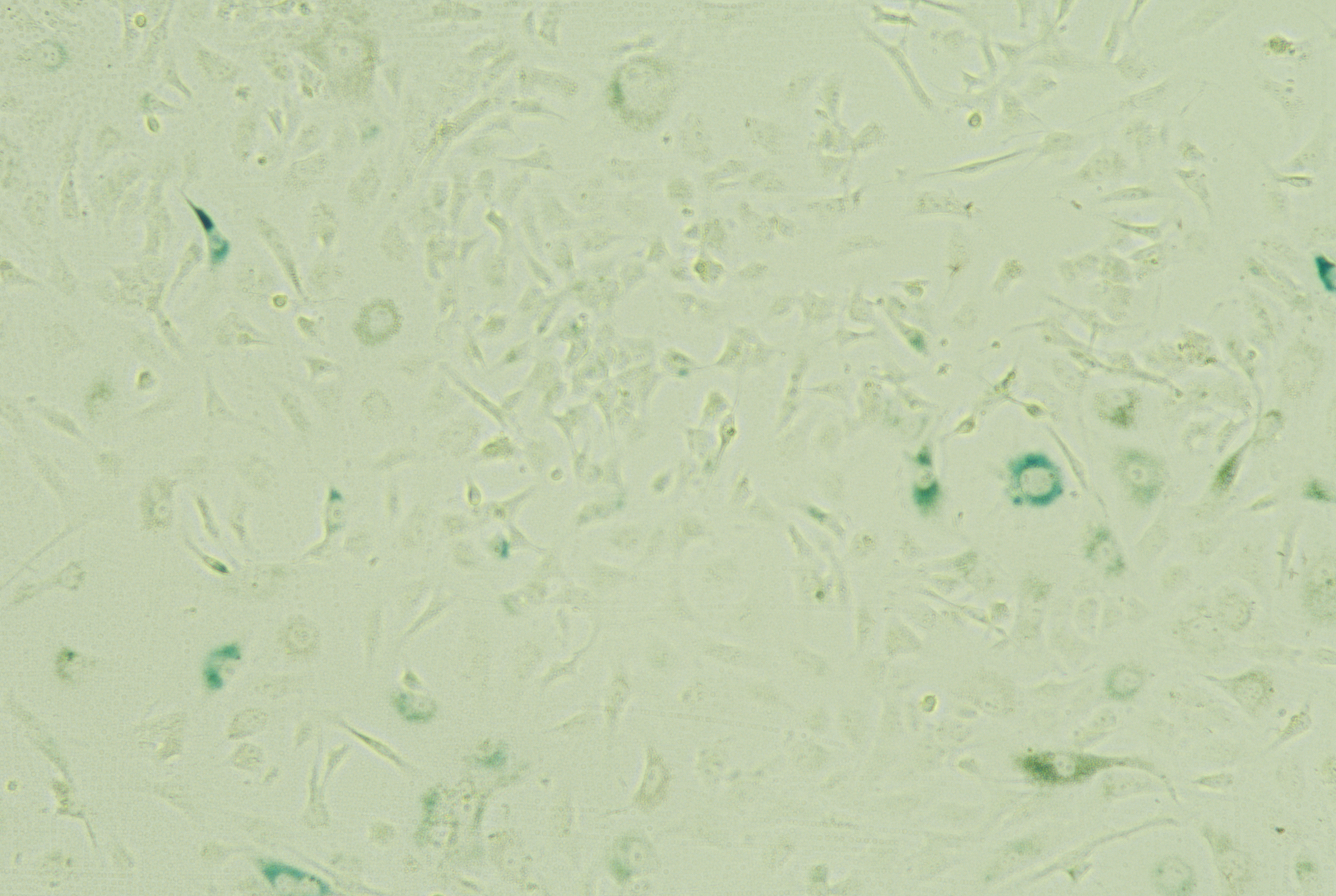

Supplement: Supplementary file 8 — Source data Fig. 3 [file 44318_2026_832_MOESM8_ESM.zip › A/G608G MSC+5HT.jpg]

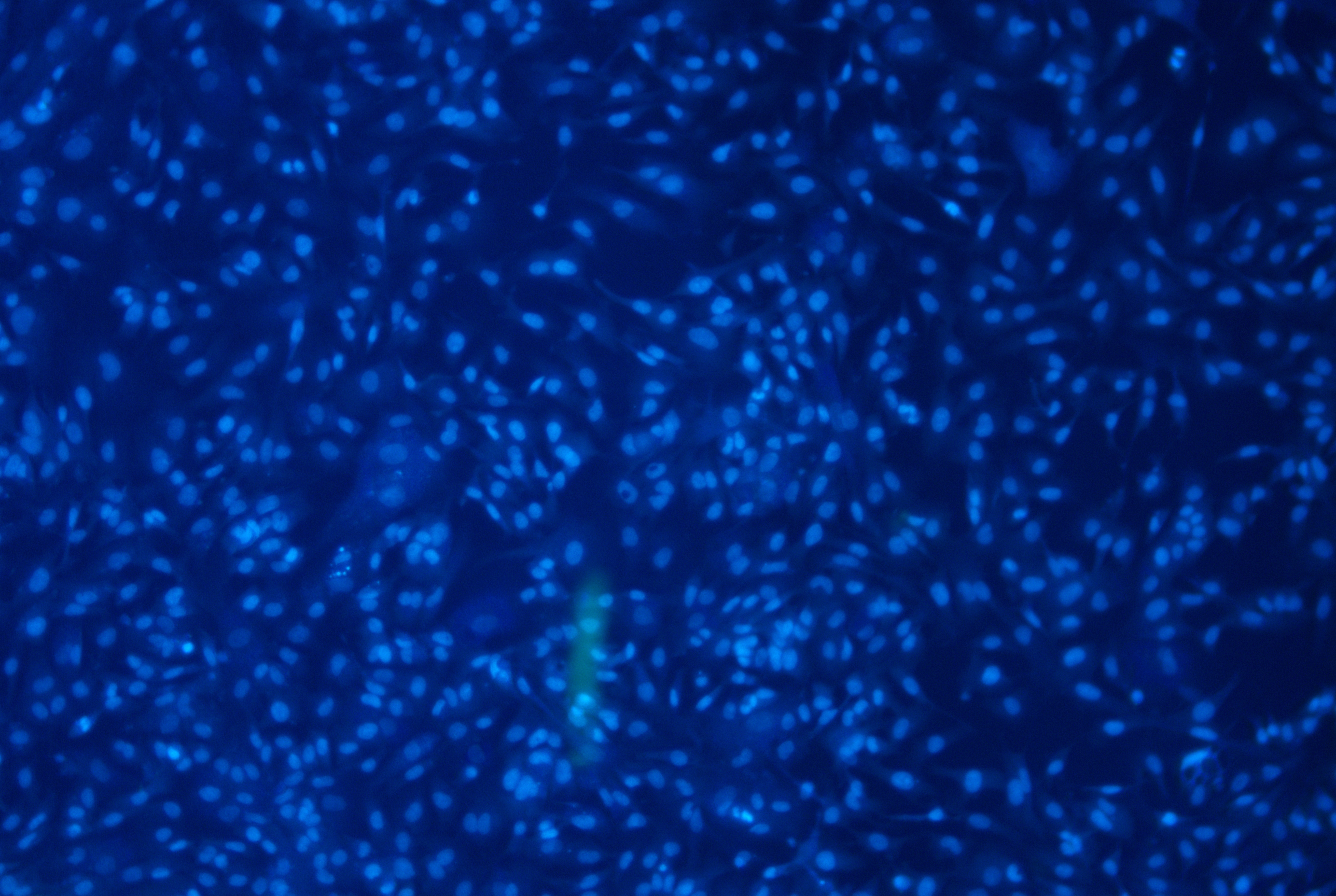

Supplement: Supplementary file 8 — Source data Fig. 3 [file 44318_2026_832_MOESM8_ESM.zip › A/G608G MSC-DAPI.jpg]

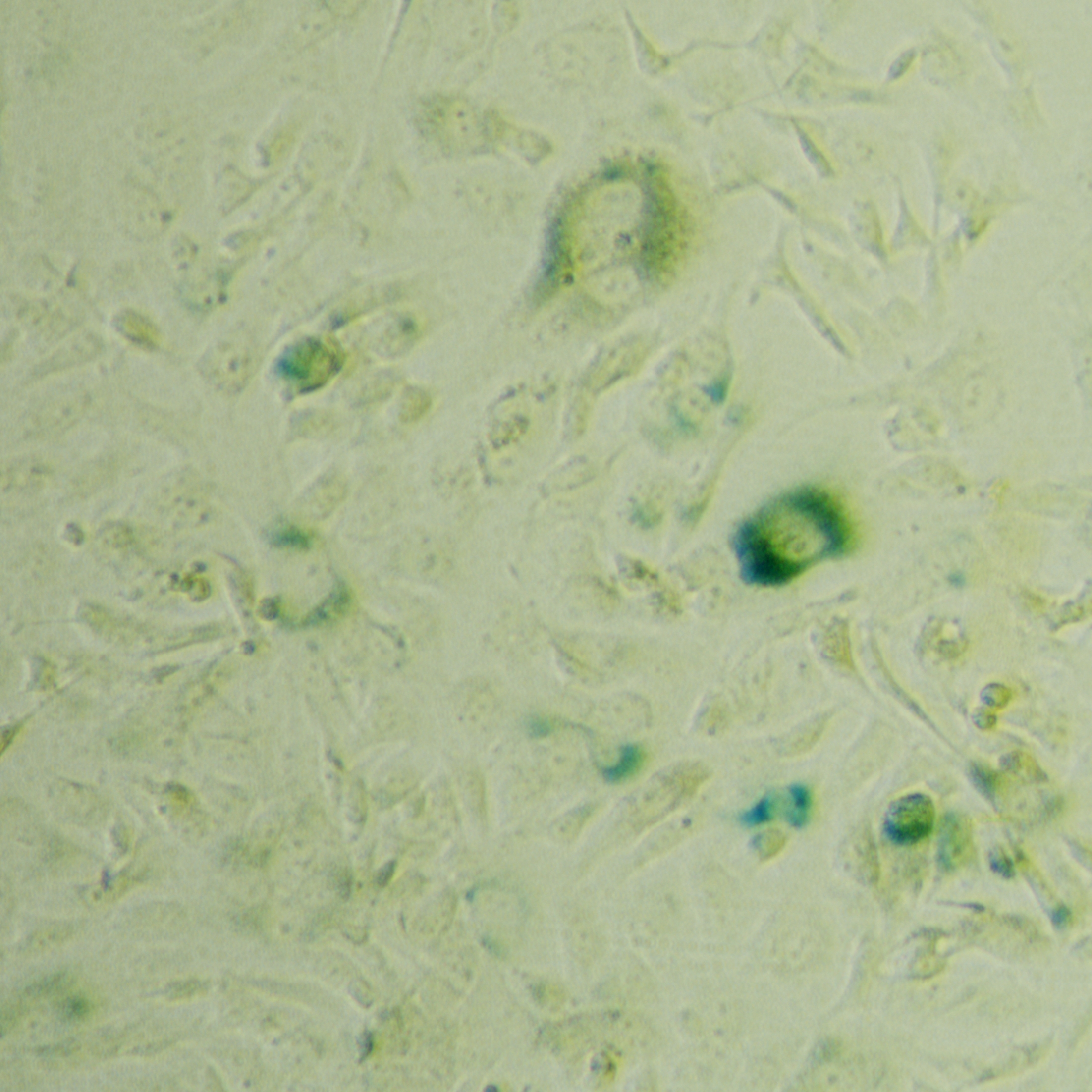

Supplement: Supplementary file 8 — Source data Fig. 3 [file 44318_2026_832_MOESM8_ESM.zip › A/G608G MSC-Large.tif]

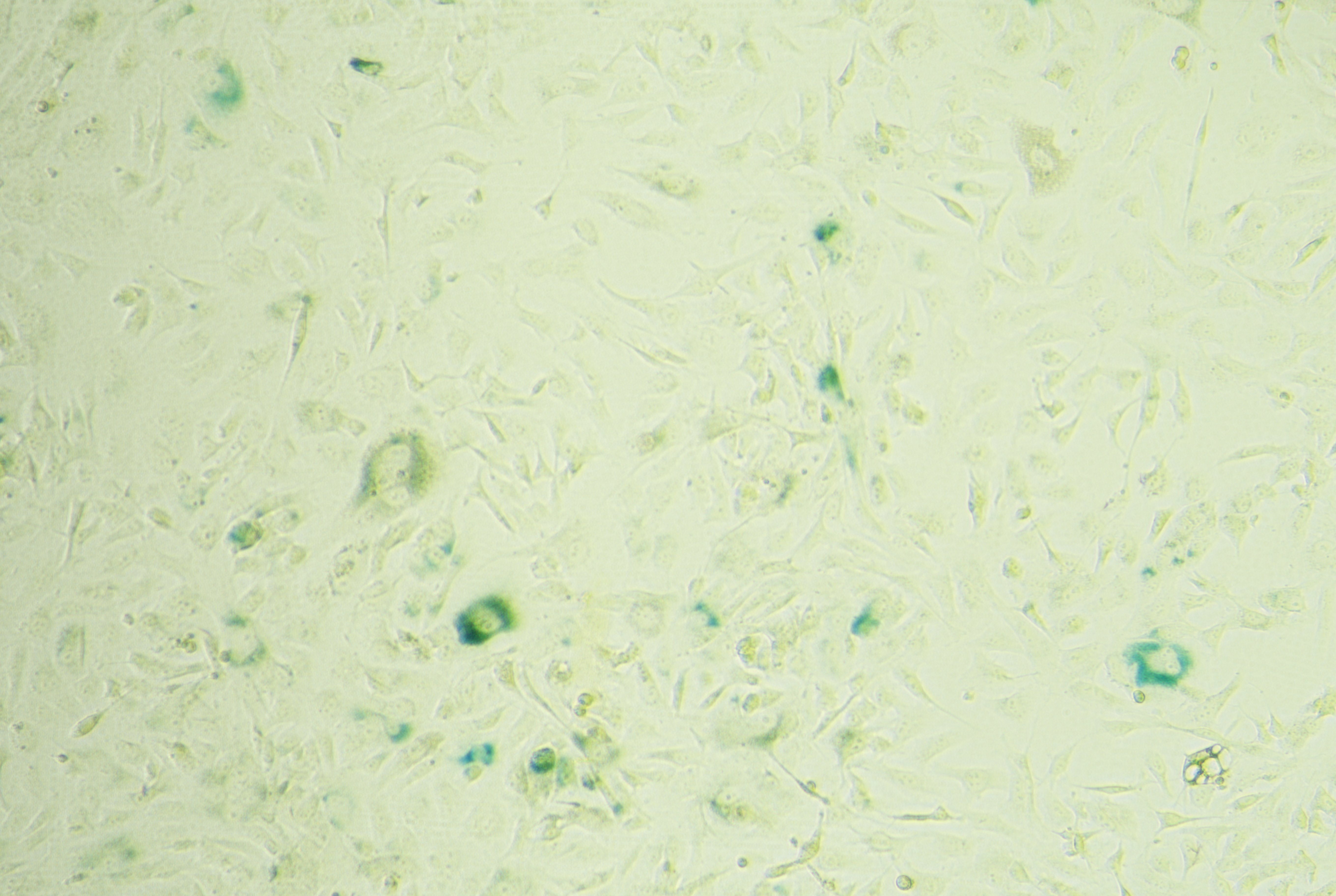

Supplement: Supplementary file 8 — Source data Fig. 3 [file 44318_2026_832_MOESM8_ESM.zip › A/G608G MSC.jpg]

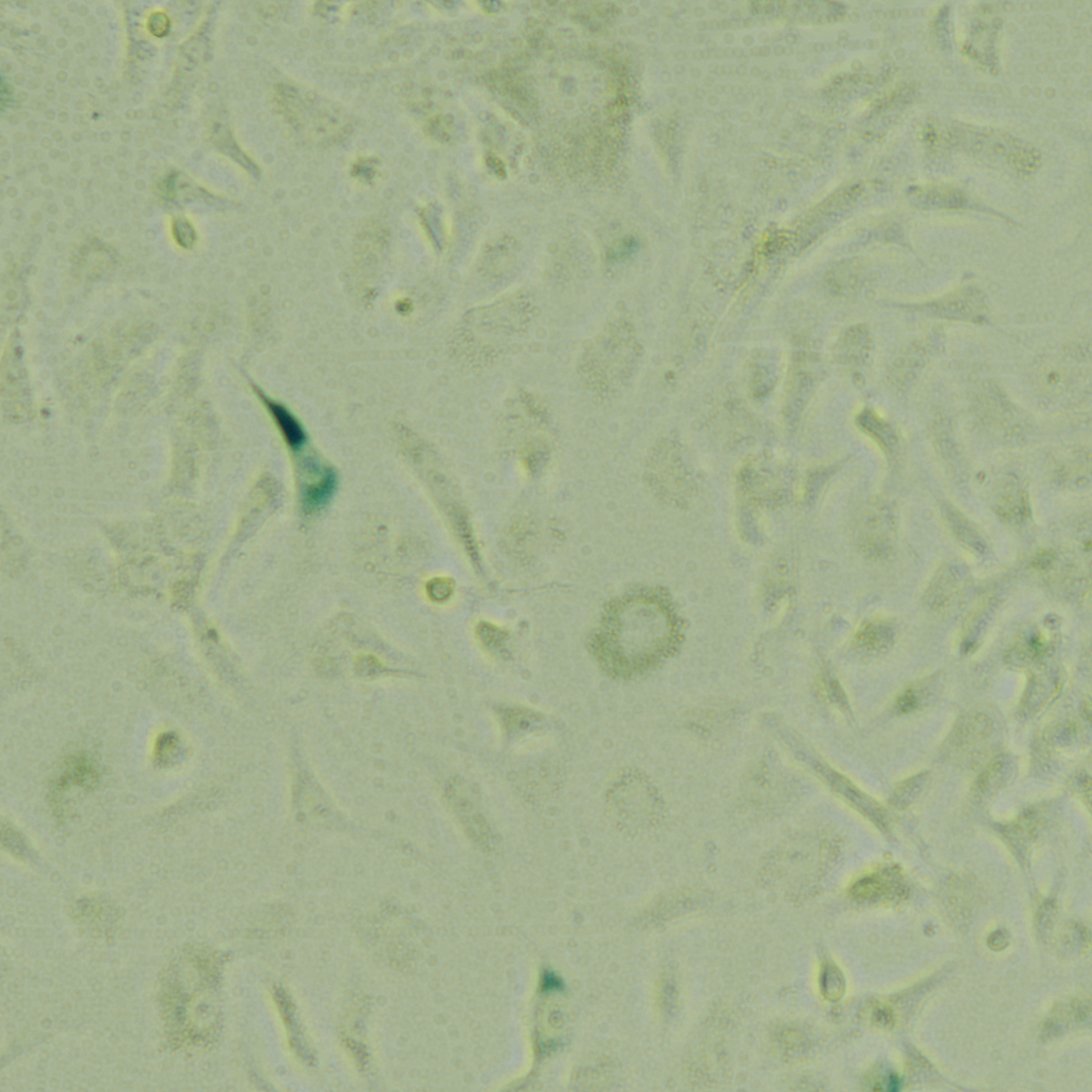

Supplement: Supplementary file 8 — Source data Fig. 3 [file 44318_2026_832_MOESM8_ESM.zip › A/G608G+5HT-Large.tif]

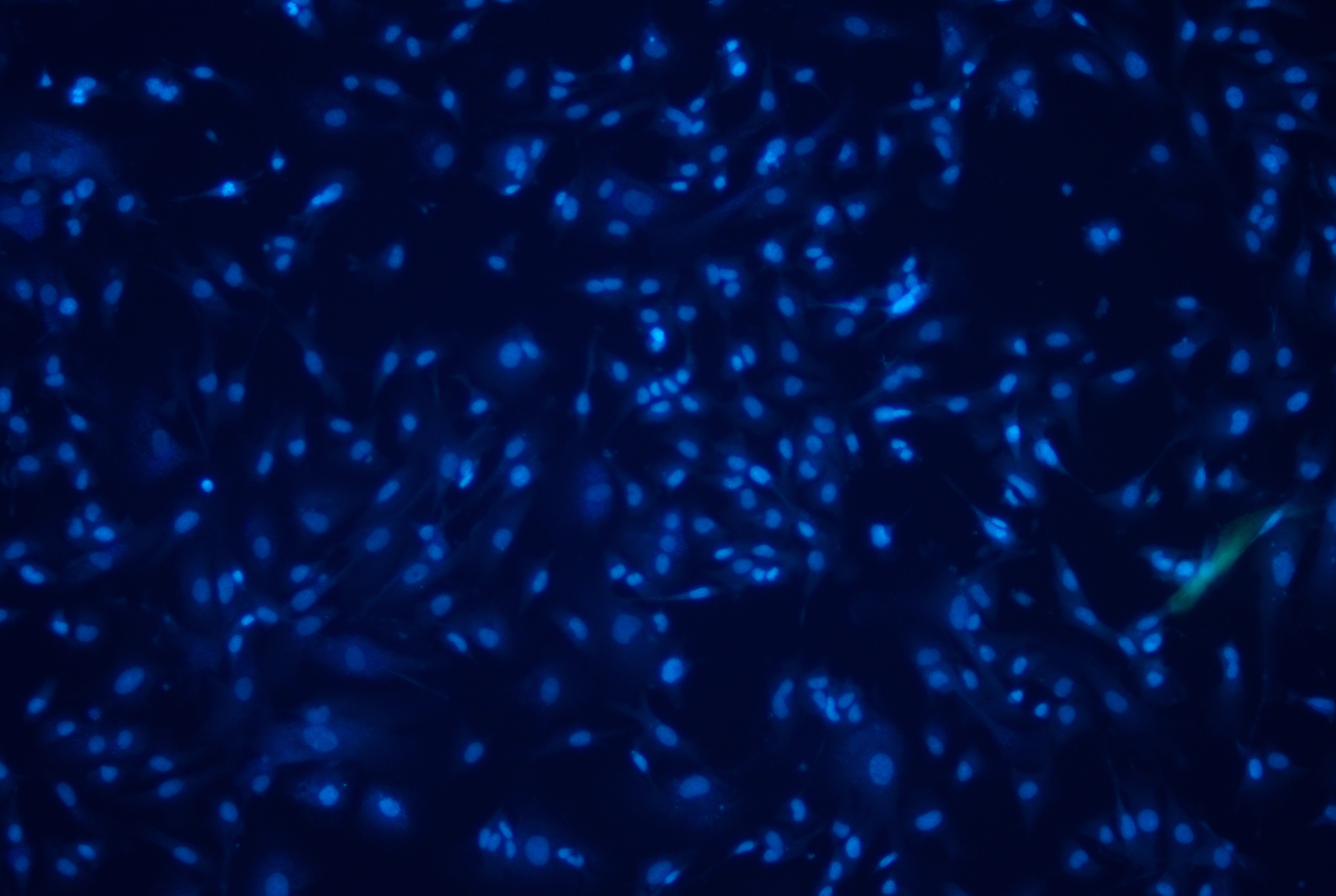

Supplement: Supplementary file 8 — Source data Fig. 3 [file 44318_2026_832_MOESM8_ESM.zip › A/WT MSC-DAPI.jpg]

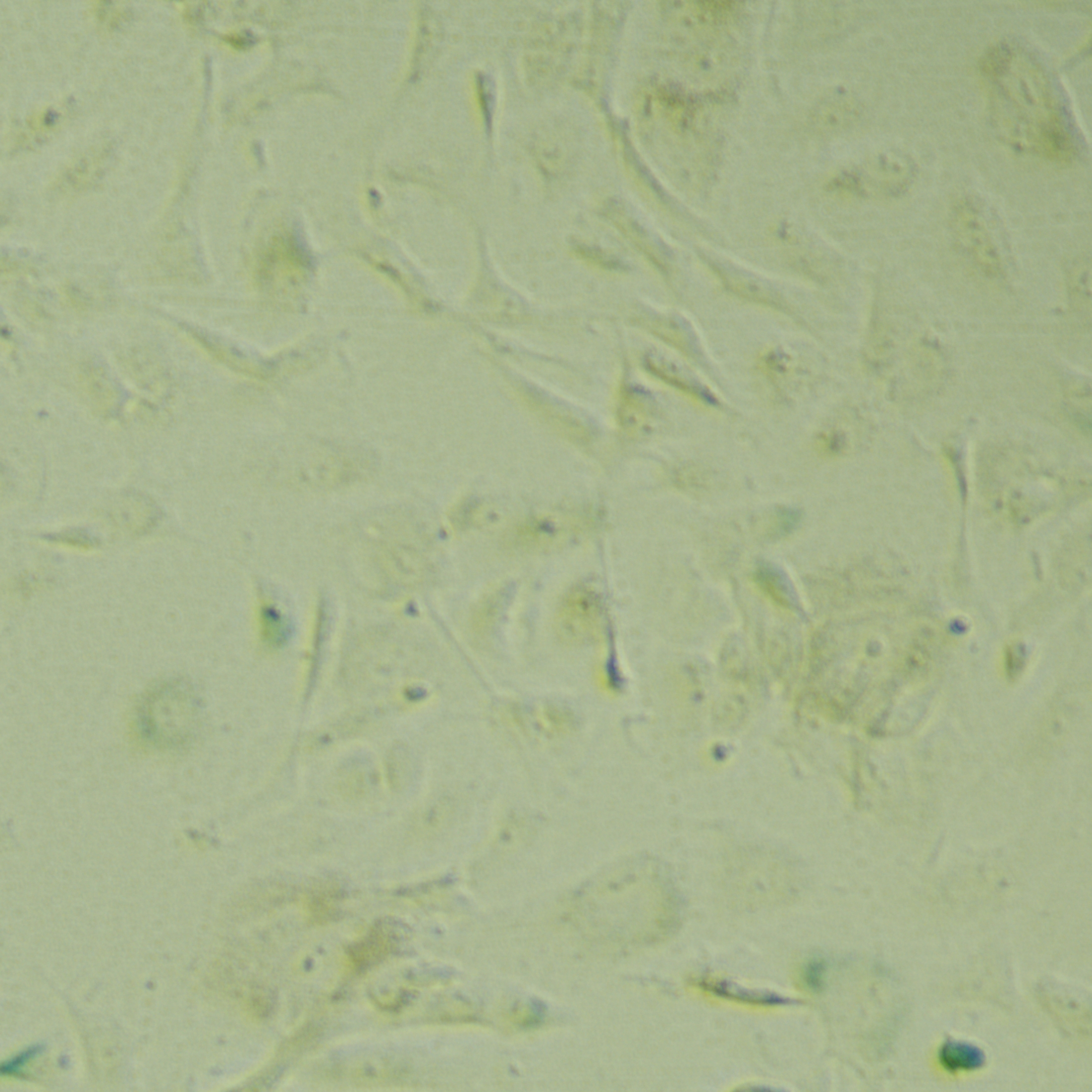

Supplement: Supplementary file 8 — Source data Fig. 3 [file 44318_2026_832_MOESM8_ESM.zip › A/WT MSC-Large.tif]

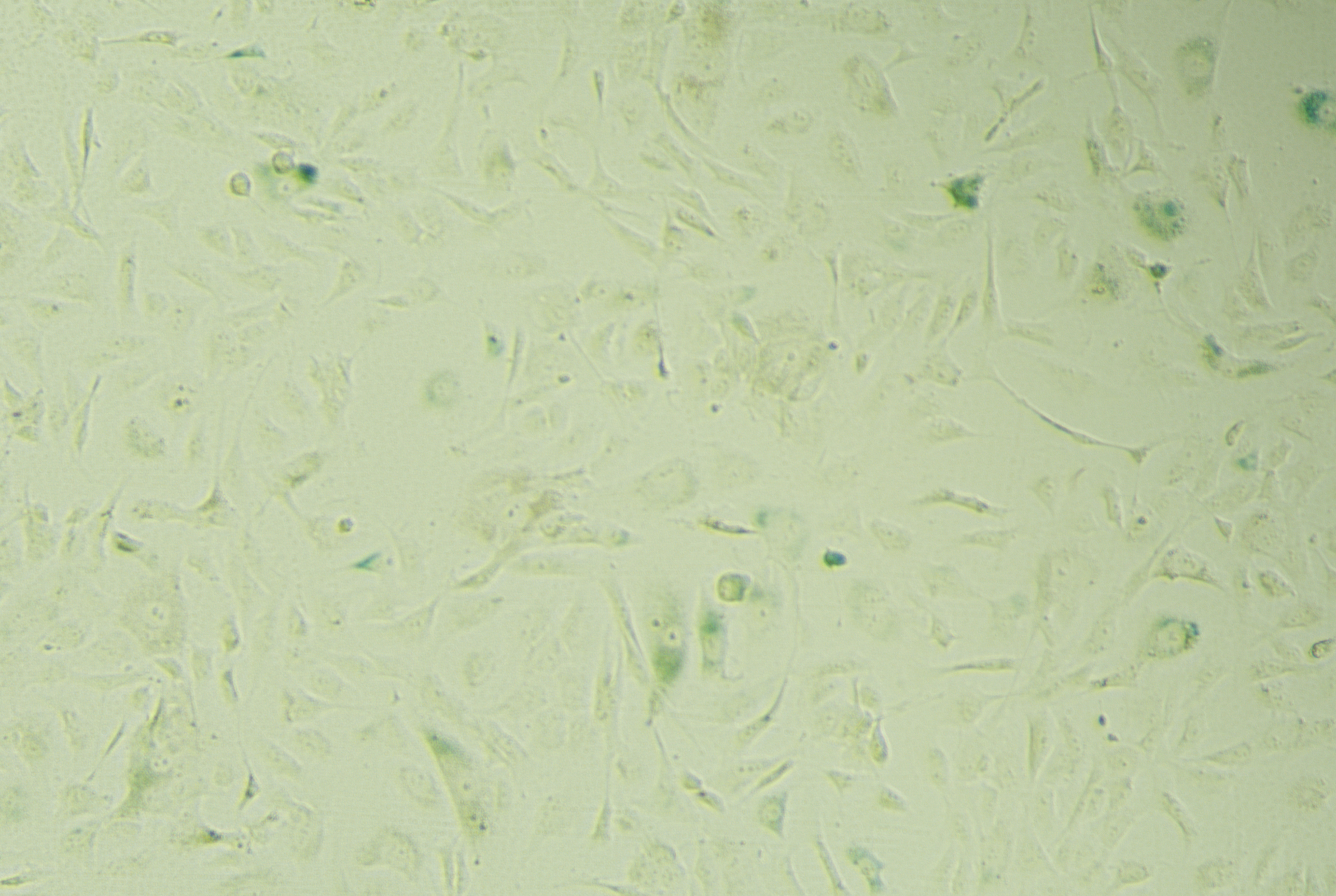

Supplement: Supplementary file 8 — Source data Fig. 3 [file 44318_2026_832_MOESM8_ESM.zip › A/WT MSC.jpg]

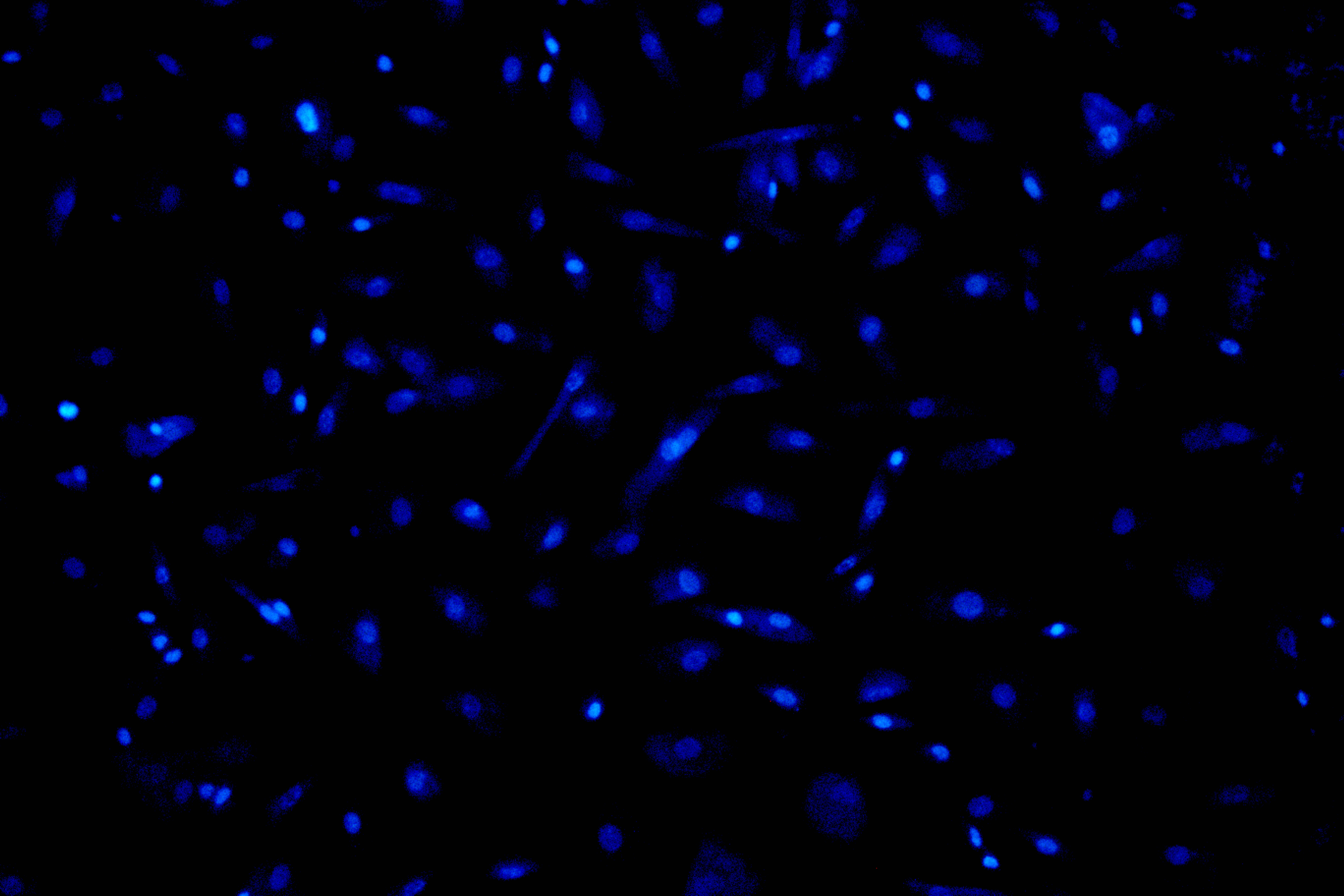

Supplement: Supplementary file 8 — Source data Fig. 3 [file 44318_2026_832_MOESM8_ESM.zip › E/WT MSC Old+5ht-DAPI.tif]

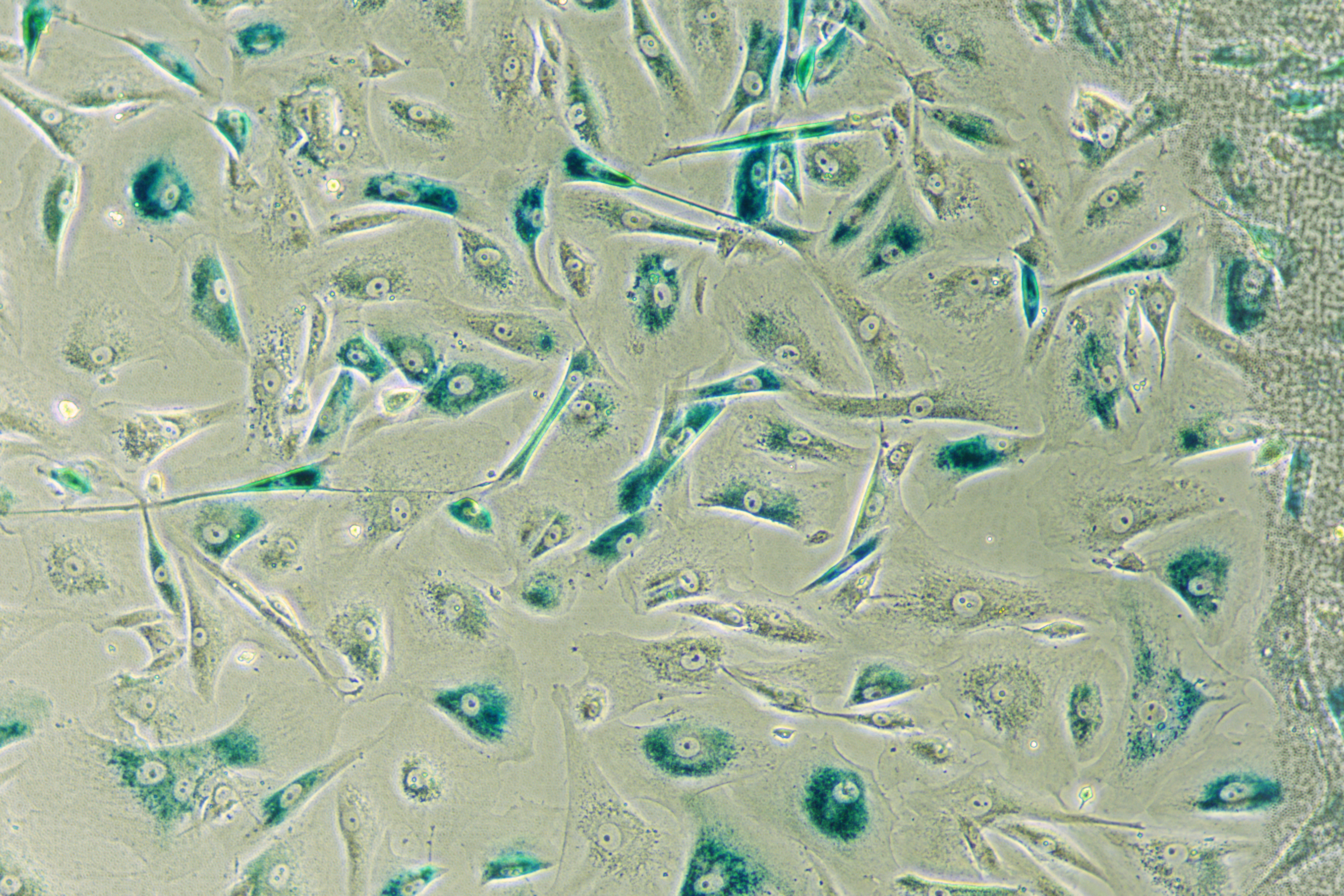

Supplement: Supplementary file 8 — Source data Fig. 3 [file 44318_2026_832_MOESM8_ESM.zip › E/WT MSC Old+5ht.tif]

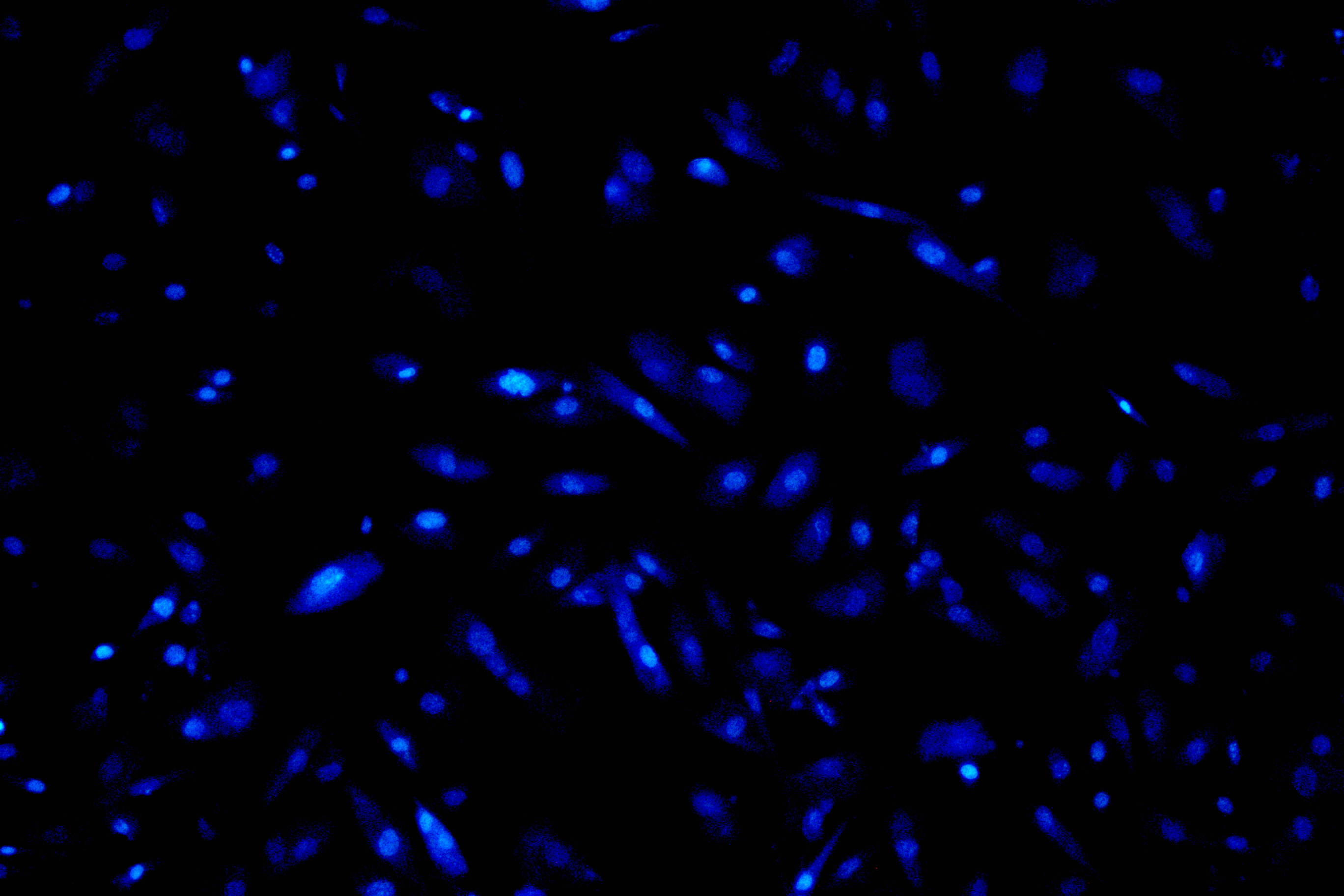

Supplement: Supplementary file 8 — Source data Fig. 3 [file 44318_2026_832_MOESM8_ESM.zip › E/WT MSC Old-DAPI.tif]

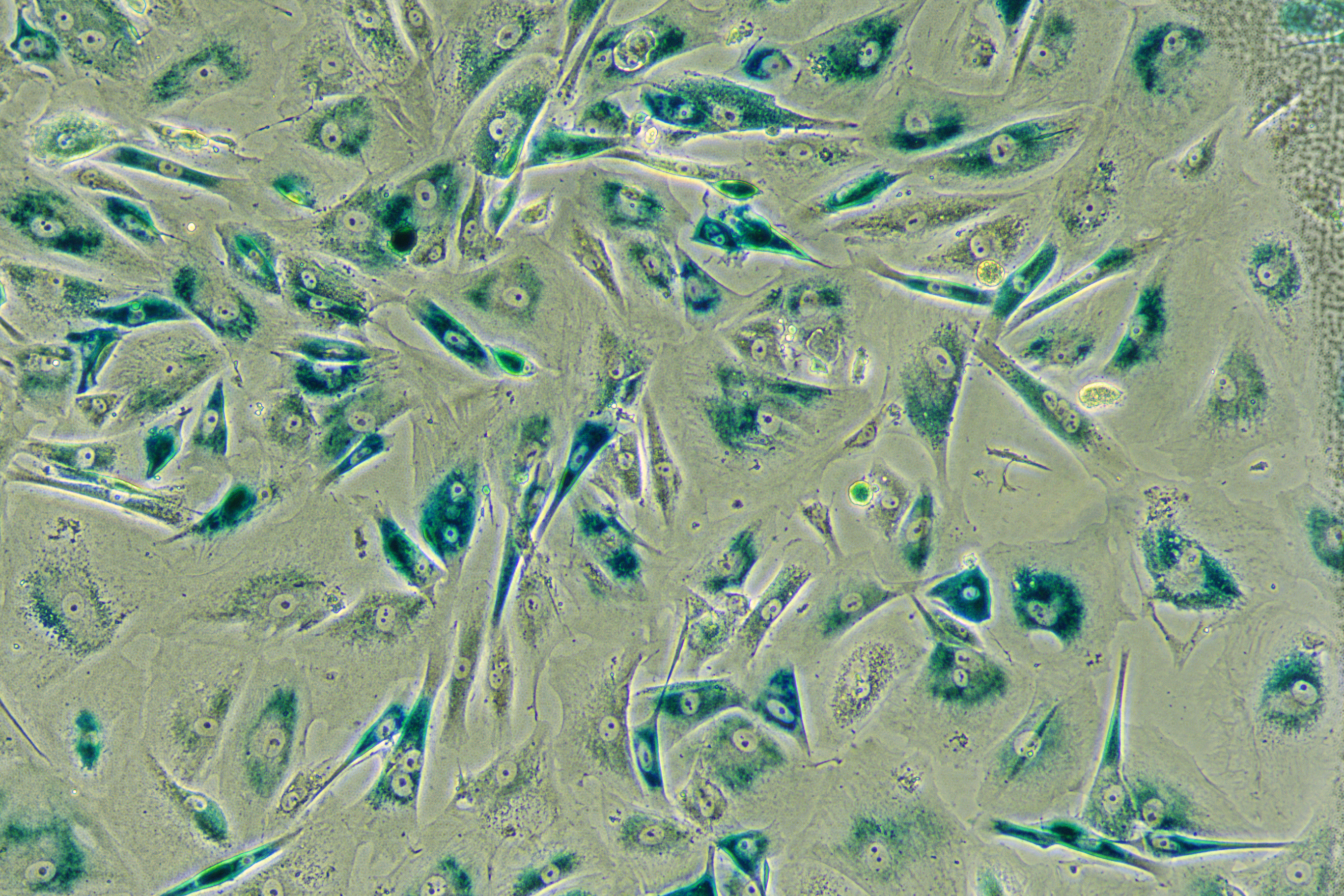

Supplement: Supplementary file 8 — Source data Fig. 3 [file 44318_2026_832_MOESM8_ESM.zip › E/WT MSC Old.tif]

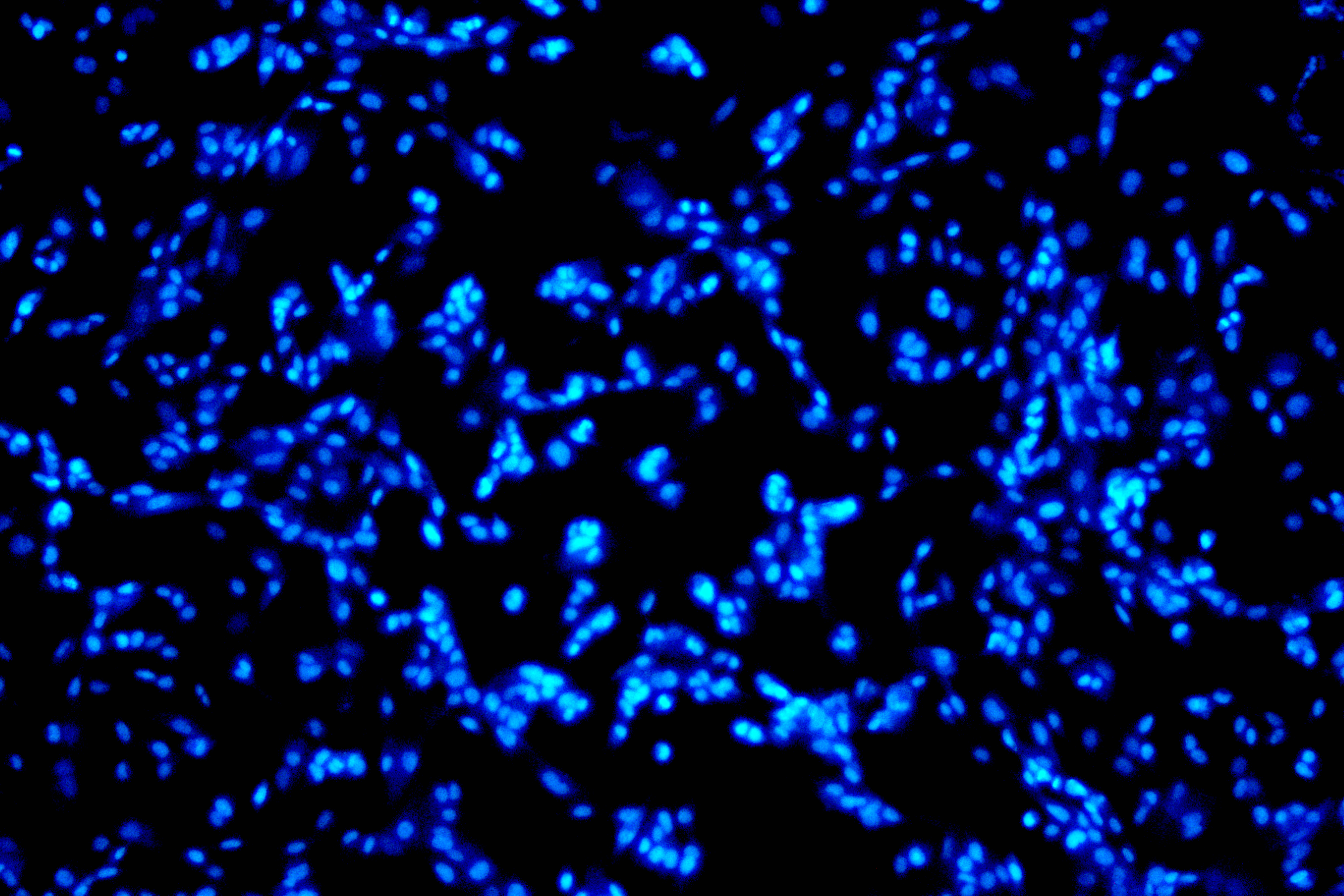

Supplement: Supplementary file 8 — Source data Fig. 3 [file 44318_2026_832_MOESM8_ESM.zip › E/WT MSC Young-DAPI.tif]

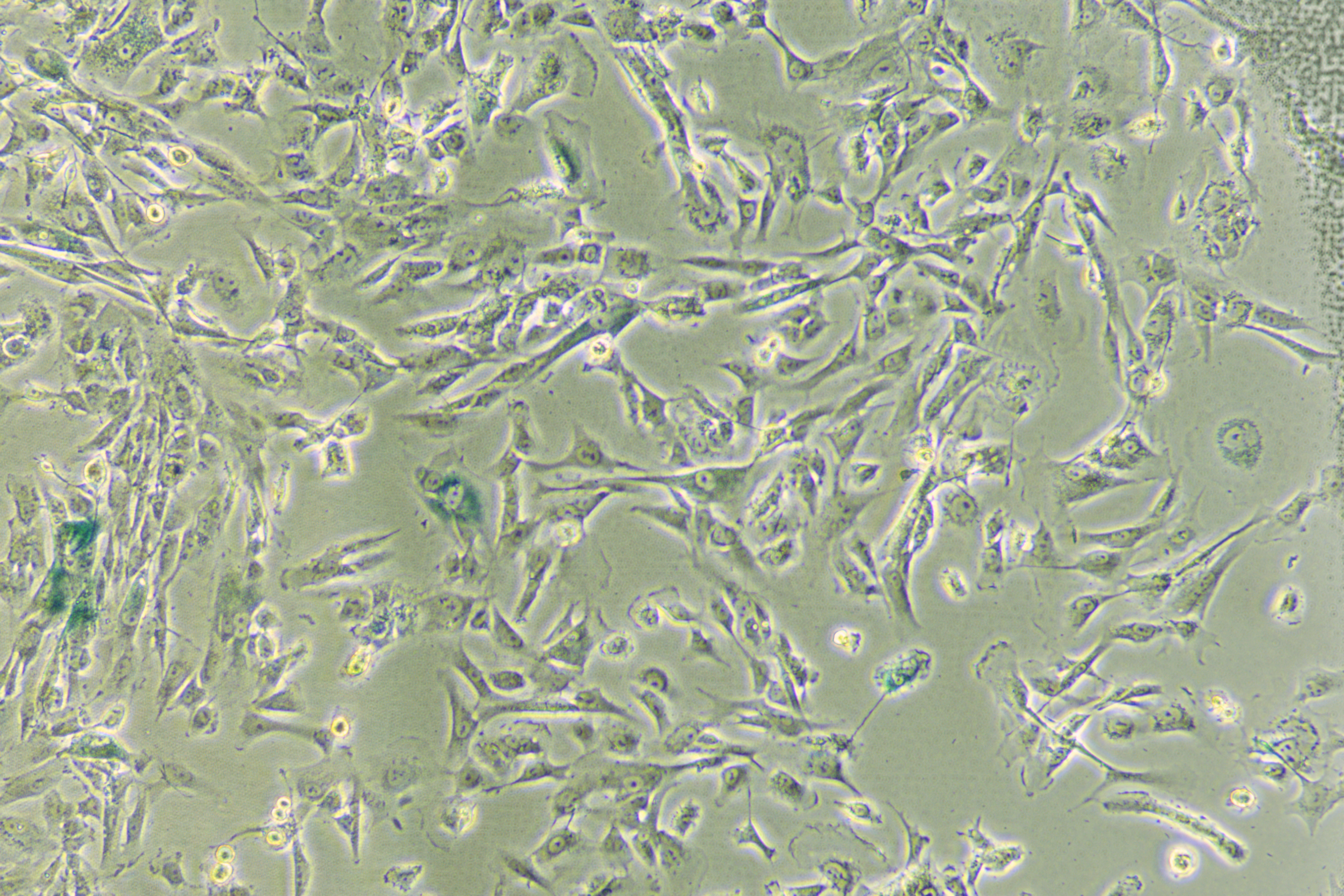

Supplement: Supplementary file 8 — Source data Fig. 3 [file 44318_2026_832_MOESM8_ESM.zip › E/WT MSC Young.tif]

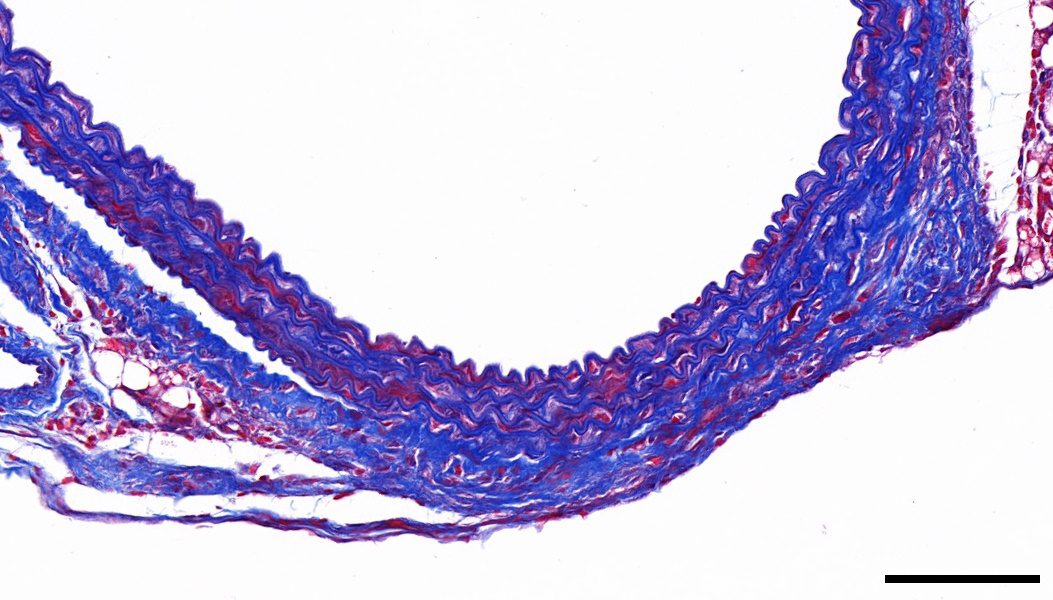

Supplement: Supplementary file 8 — Source data Fig. 3 [file 44318_2026_832_MOESM8_ESM.zip › L/G608G aorta.jpg]

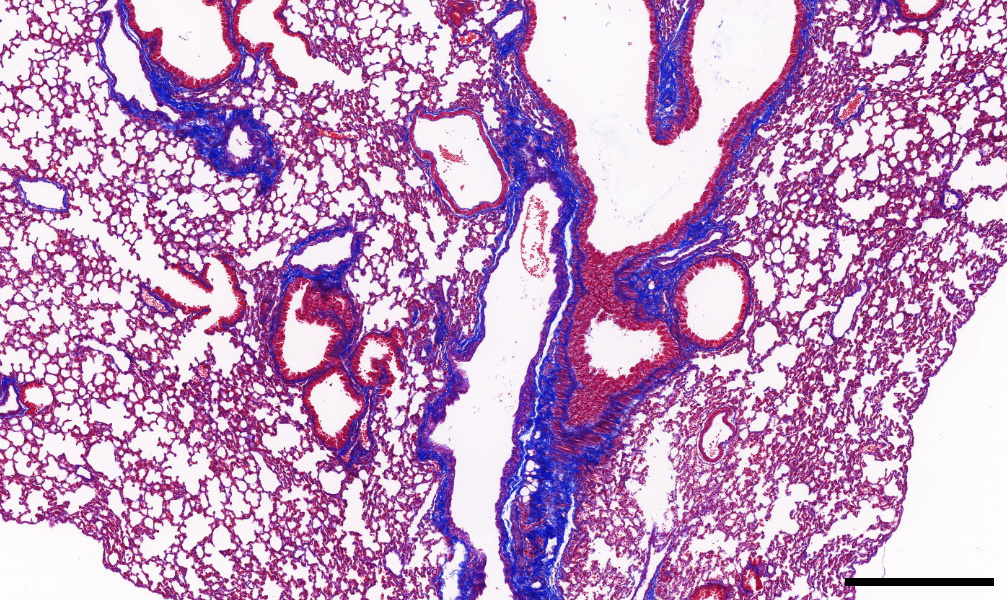

Supplement: Supplementary file 8 — Source data Fig. 3 [file 44318_2026_832_MOESM8_ESM.zip › L/G608G lung.jpg]

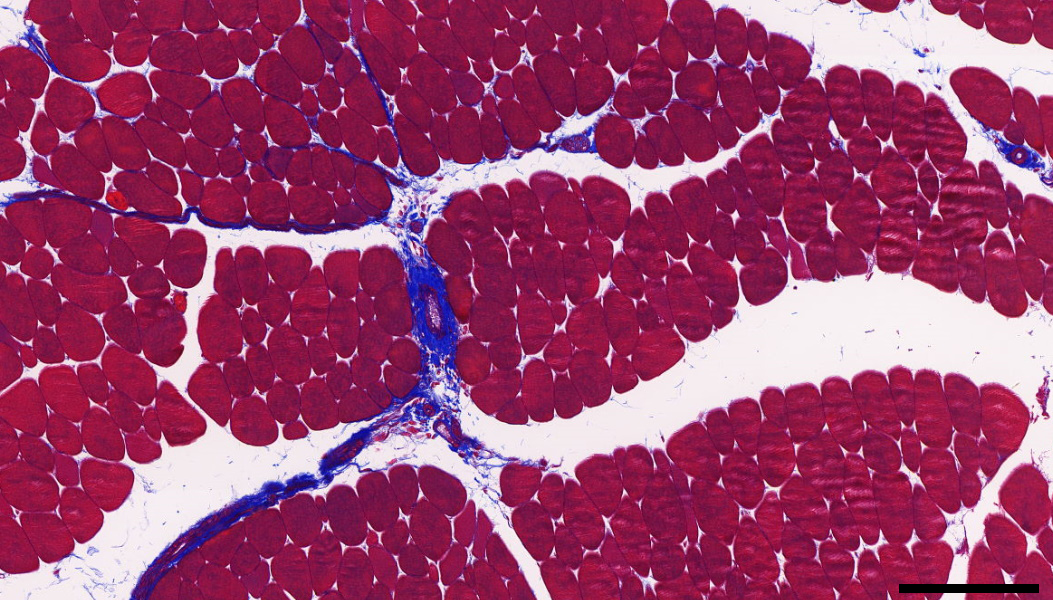

Supplement: Supplementary file 8 — Source data Fig. 3 [file 44318_2026_832_MOESM8_ESM.zip › L/G608G muscle.jpg]

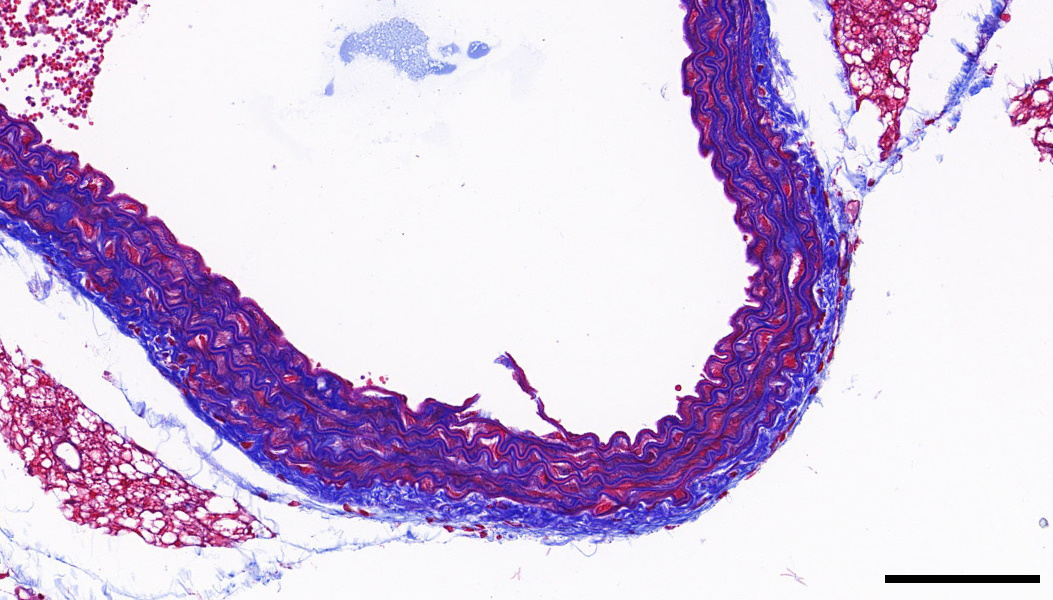

Supplement: Supplementary file 8 — Source data Fig. 3 [file 44318_2026_832_MOESM8_ESM.zip › L/G608G+5ht aorta.jpg]

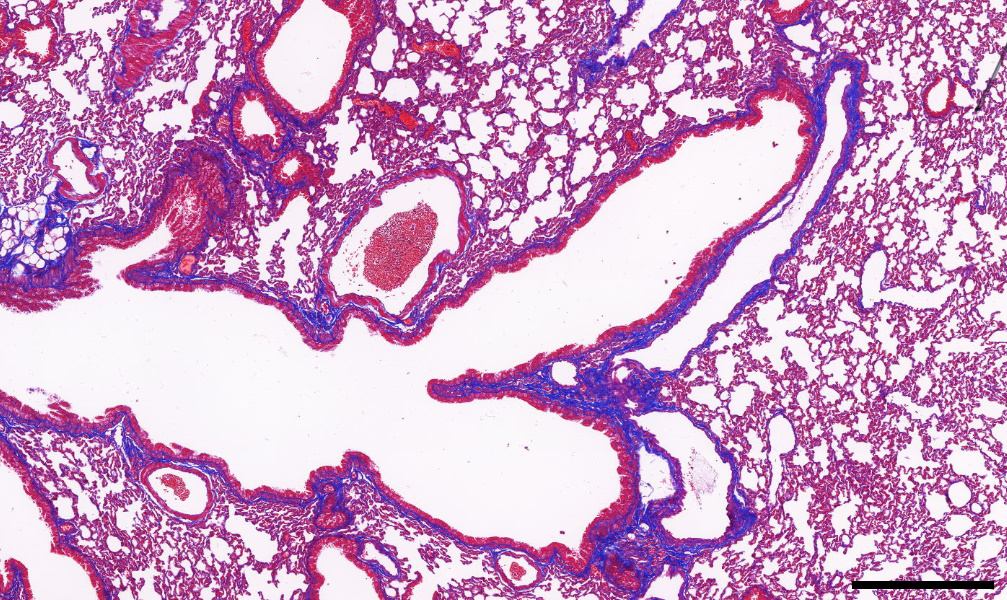

Supplement: Supplementary file 8 — Source data Fig. 3 [file 44318_2026_832_MOESM8_ESM.zip › L/G608G+5ht lung.jpg]

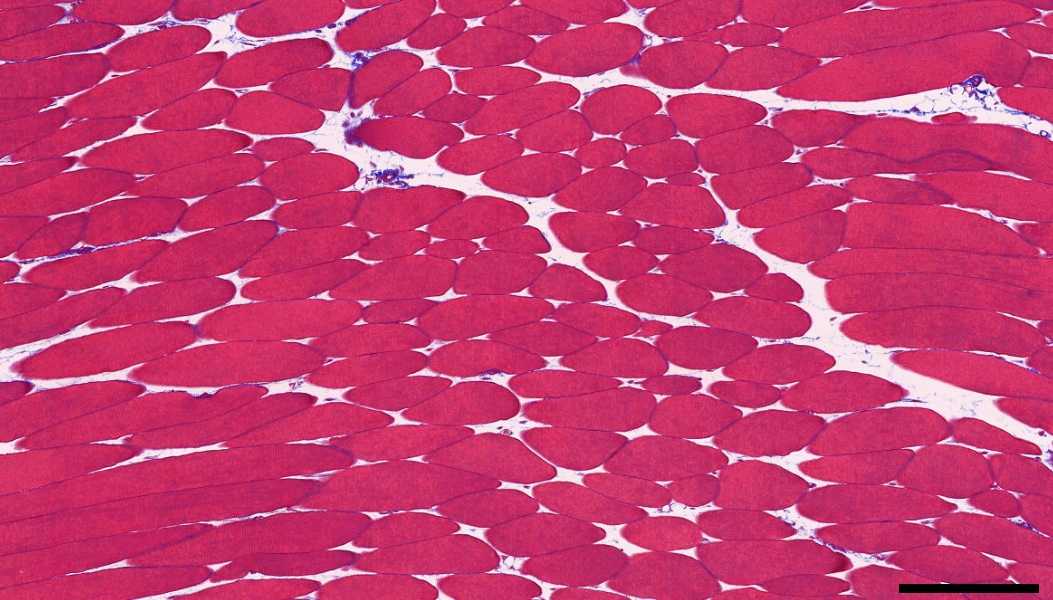

Supplement: Supplementary file 8 — Source data Fig. 3 [file 44318_2026_832_MOESM8_ESM.zip › L/G608G+5ht muscle.jpg]

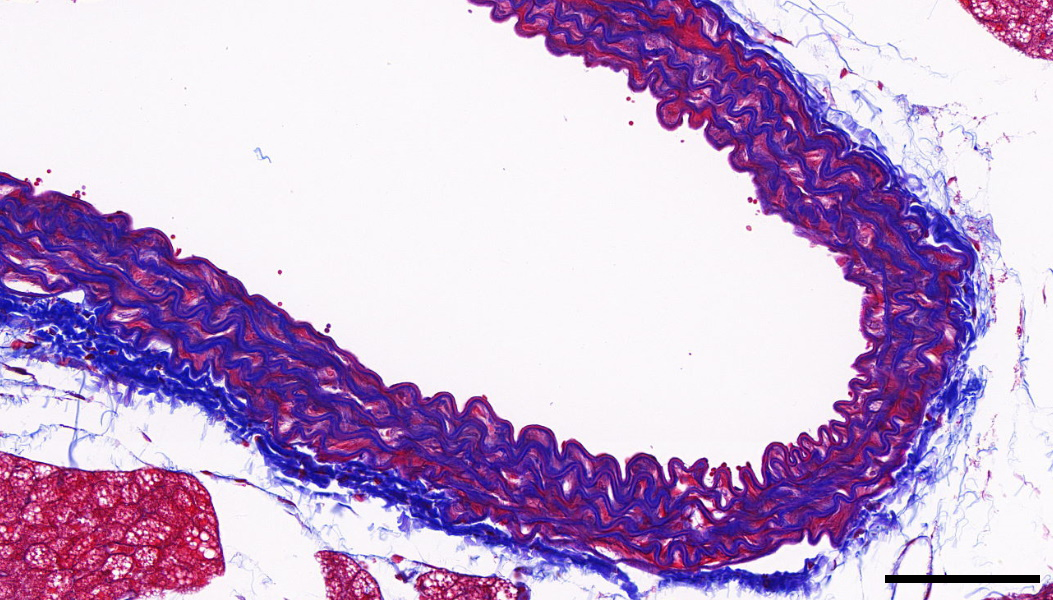

Supplement: Supplementary file 8 — Source data Fig. 3 [file 44318_2026_832_MOESM8_ESM.zip › L/WT aorta.jpg]

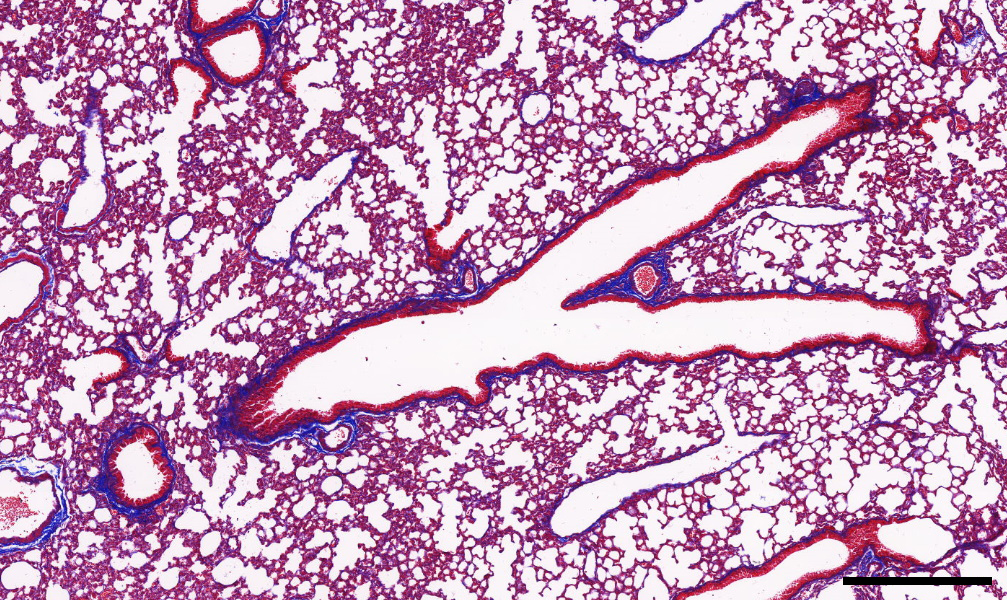

Supplement: Supplementary file 8 — Source data Fig. 3 [file 44318_2026_832_MOESM8_ESM.zip › L/WT lung .jpg]

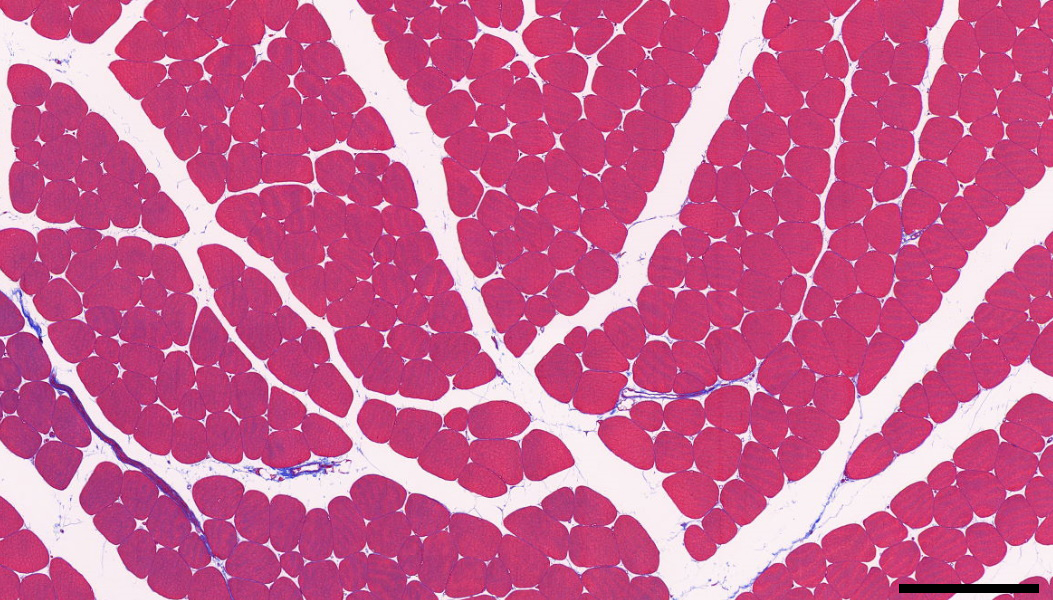

Supplement: Supplementary file 8 — Source data Fig. 3 [file 44318_2026_832_MOESM8_ESM.zip › L/WT muscle.jpg]

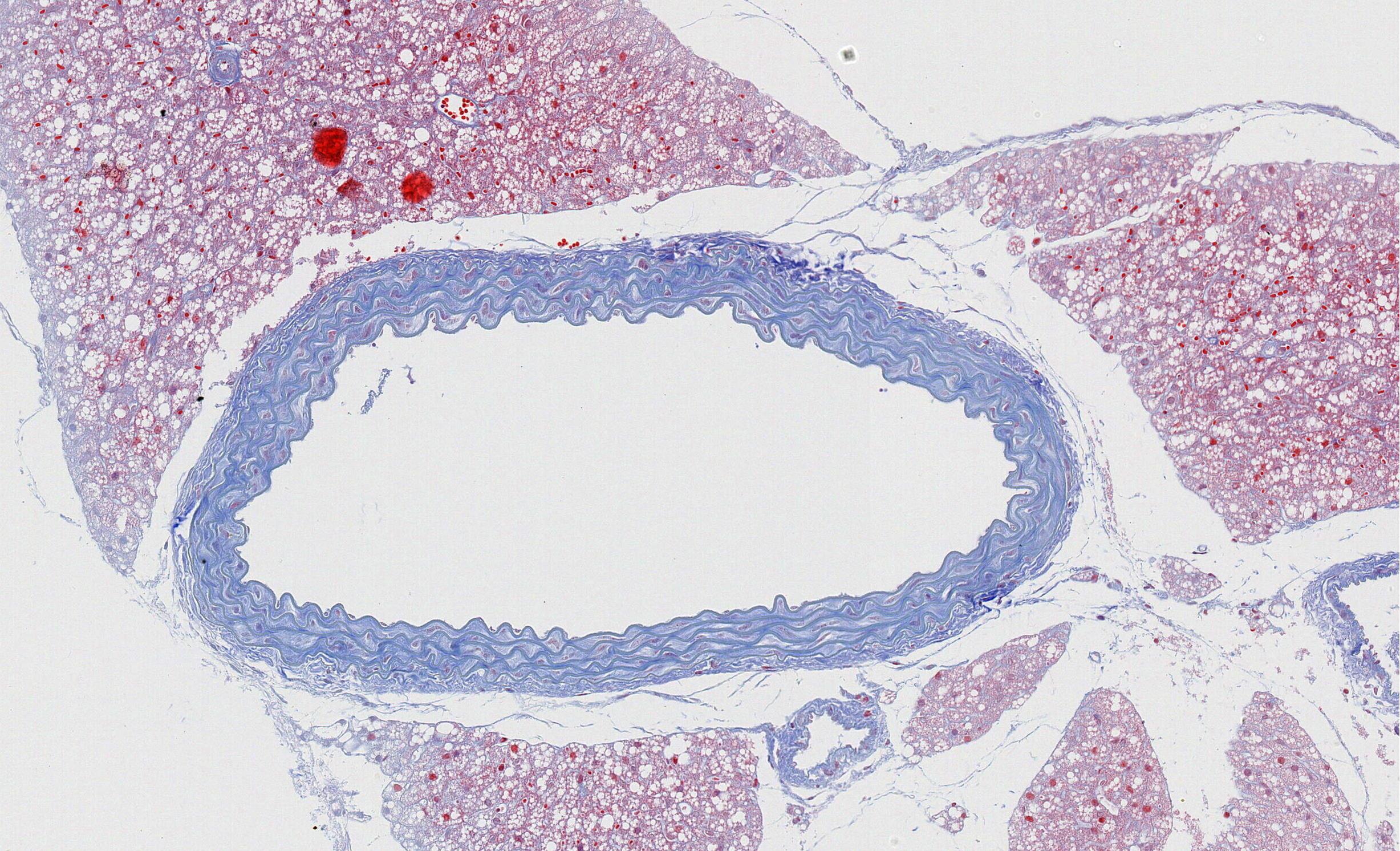

Supplement: Supplementary file 8 — Source data Fig. 3 [file 44318_2026_832_MOESM8_ESM.zip › P/4W aorta.jpg]

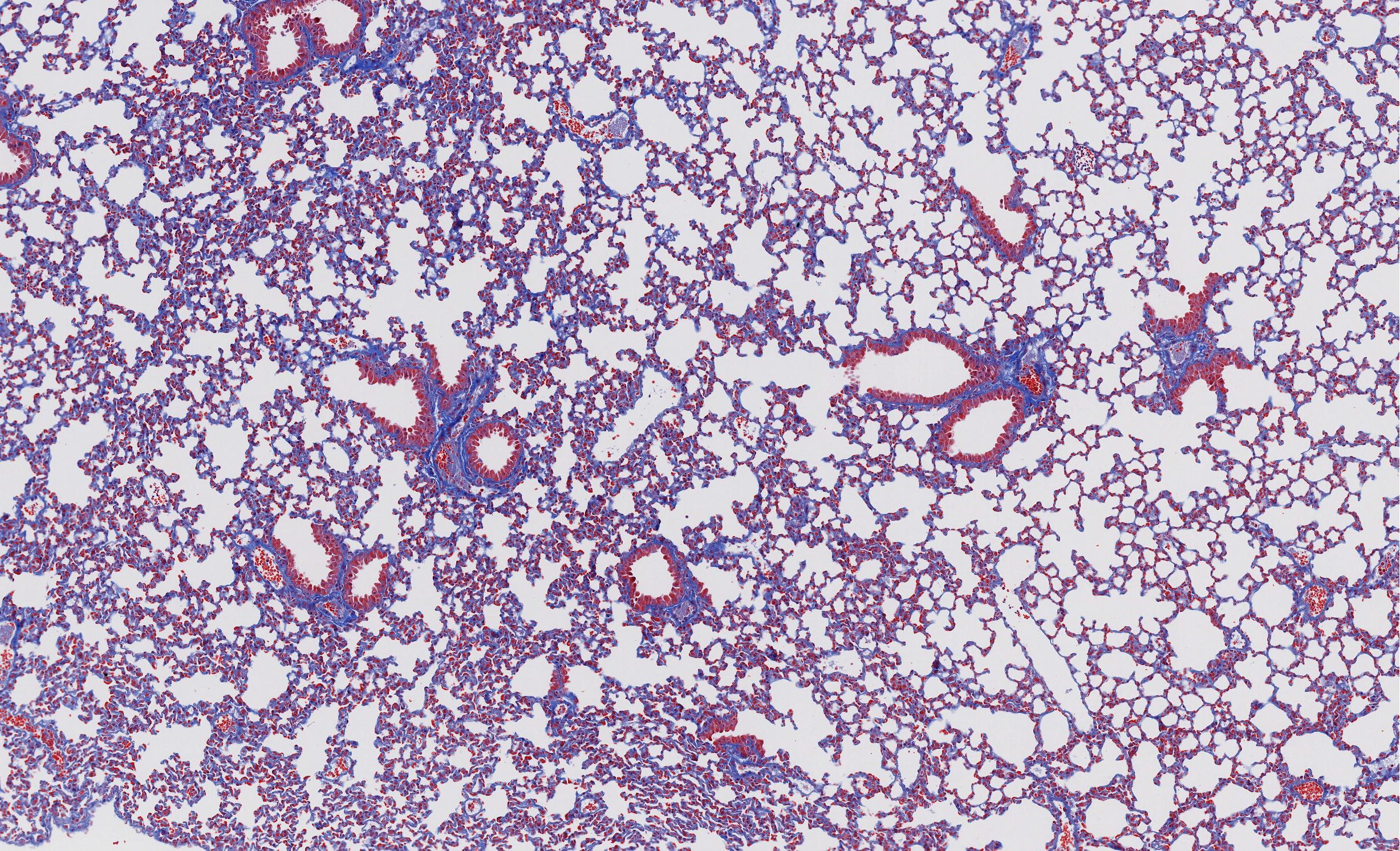

Supplement: Supplementary file 8 — Source data Fig. 3 [file 44318_2026_832_MOESM8_ESM.zip › P/4W lung.jpg]

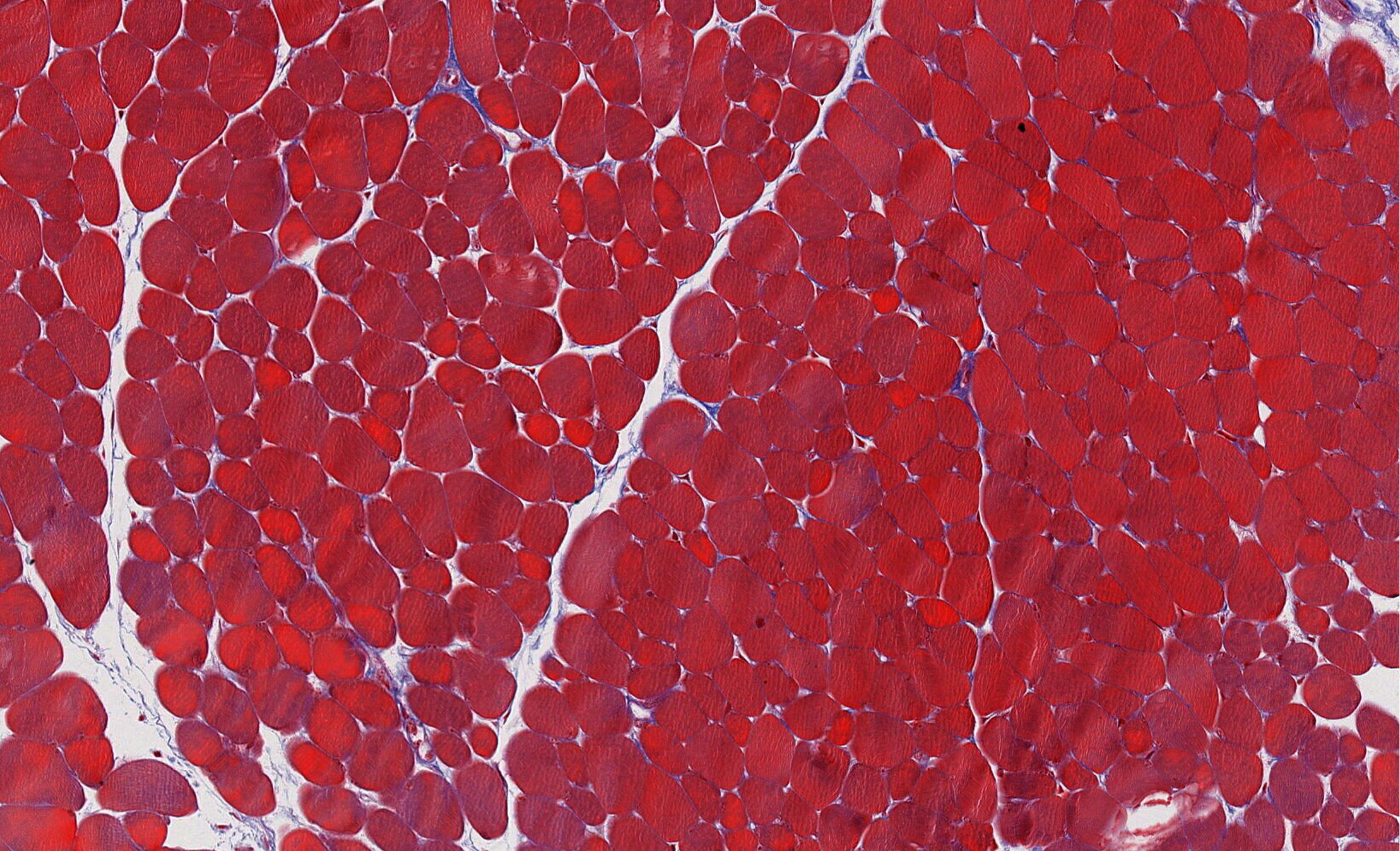

Supplement: Supplementary file 8 — Source data Fig. 3 [file 44318_2026_832_MOESM8_ESM.zip › P/4W muscle.jpg]

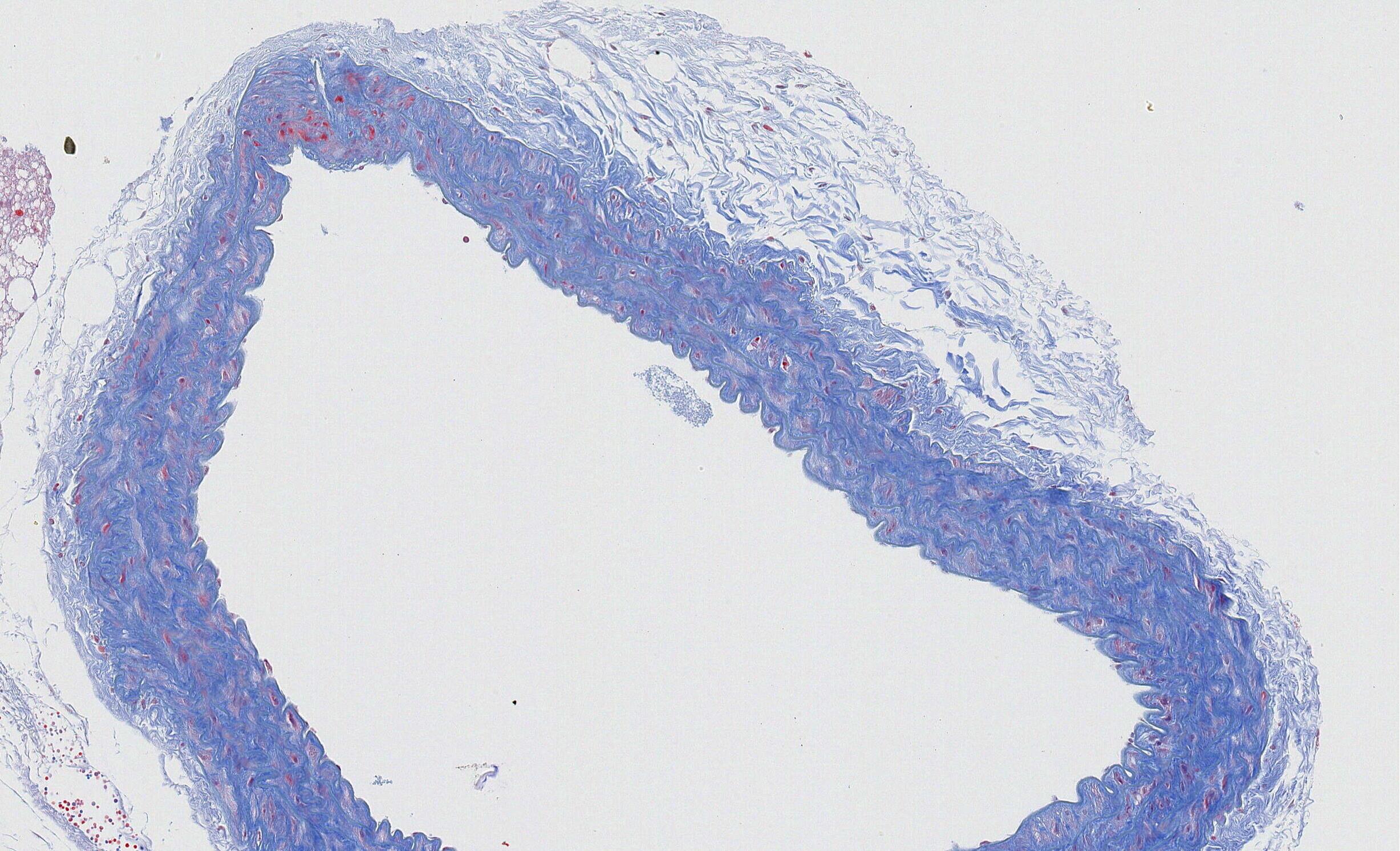

Supplement: Supplementary file 8 — Source data Fig. 3 [file 44318_2026_832_MOESM8_ESM.zip › P/WT old aorta.jpg]

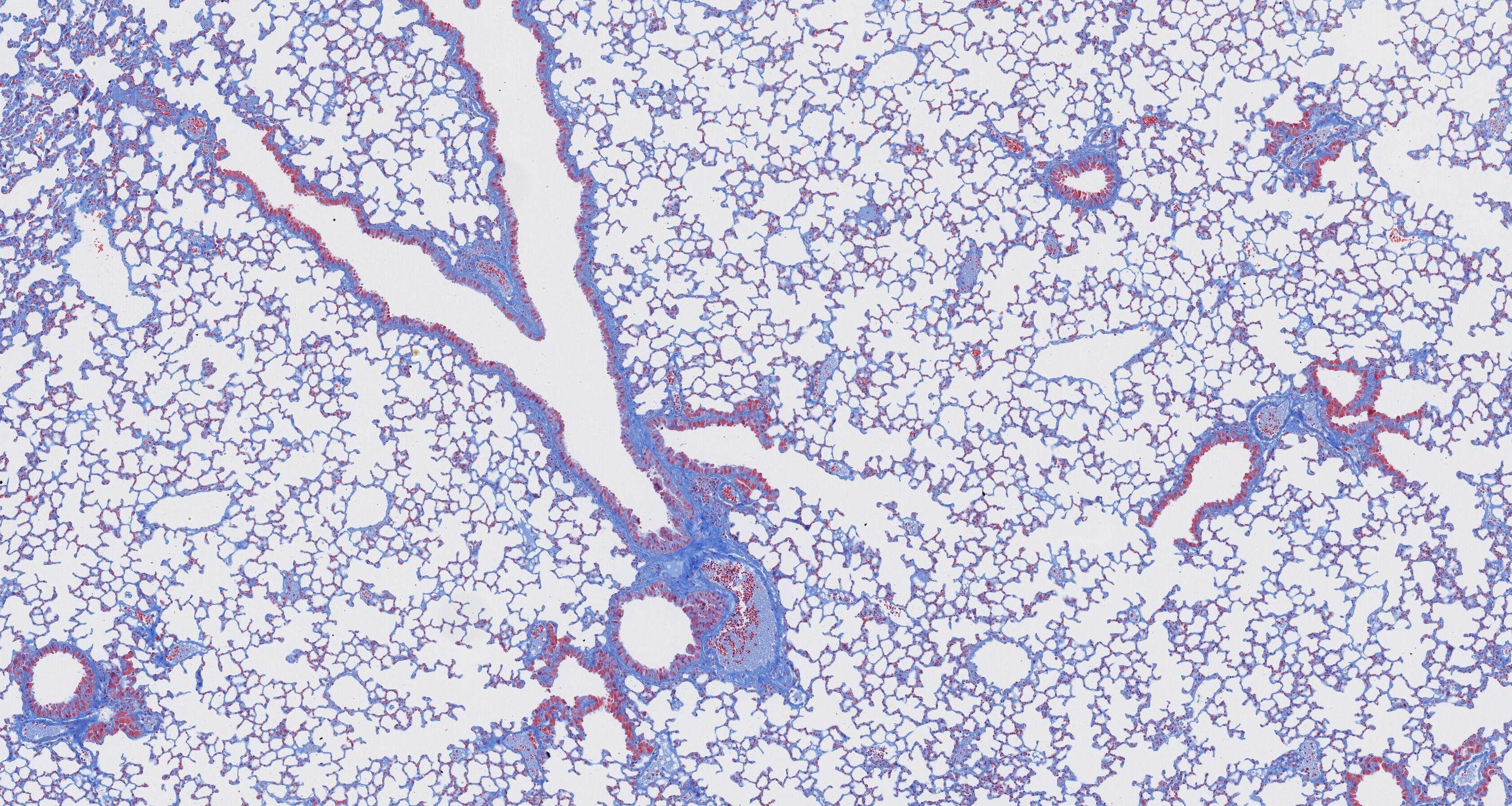

Supplement: Supplementary file 8 — Source data Fig. 3 [file 44318_2026_832_MOESM8_ESM.zip › P/WT old lung.jpg]

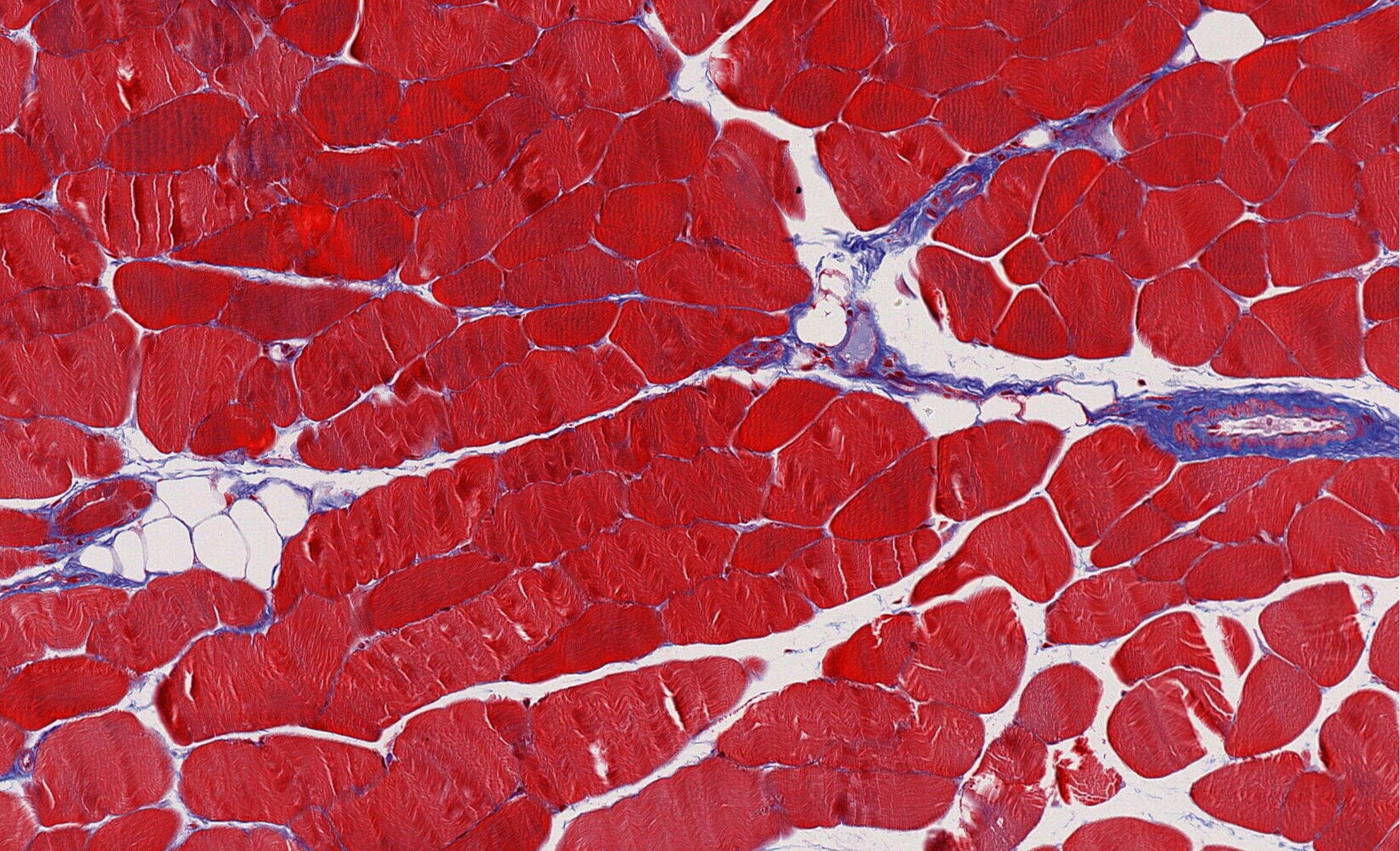

Supplement: Supplementary file 8 — Source data Fig. 3 [file 44318_2026_832_MOESM8_ESM.zip › P/WT old muscle.jpg]

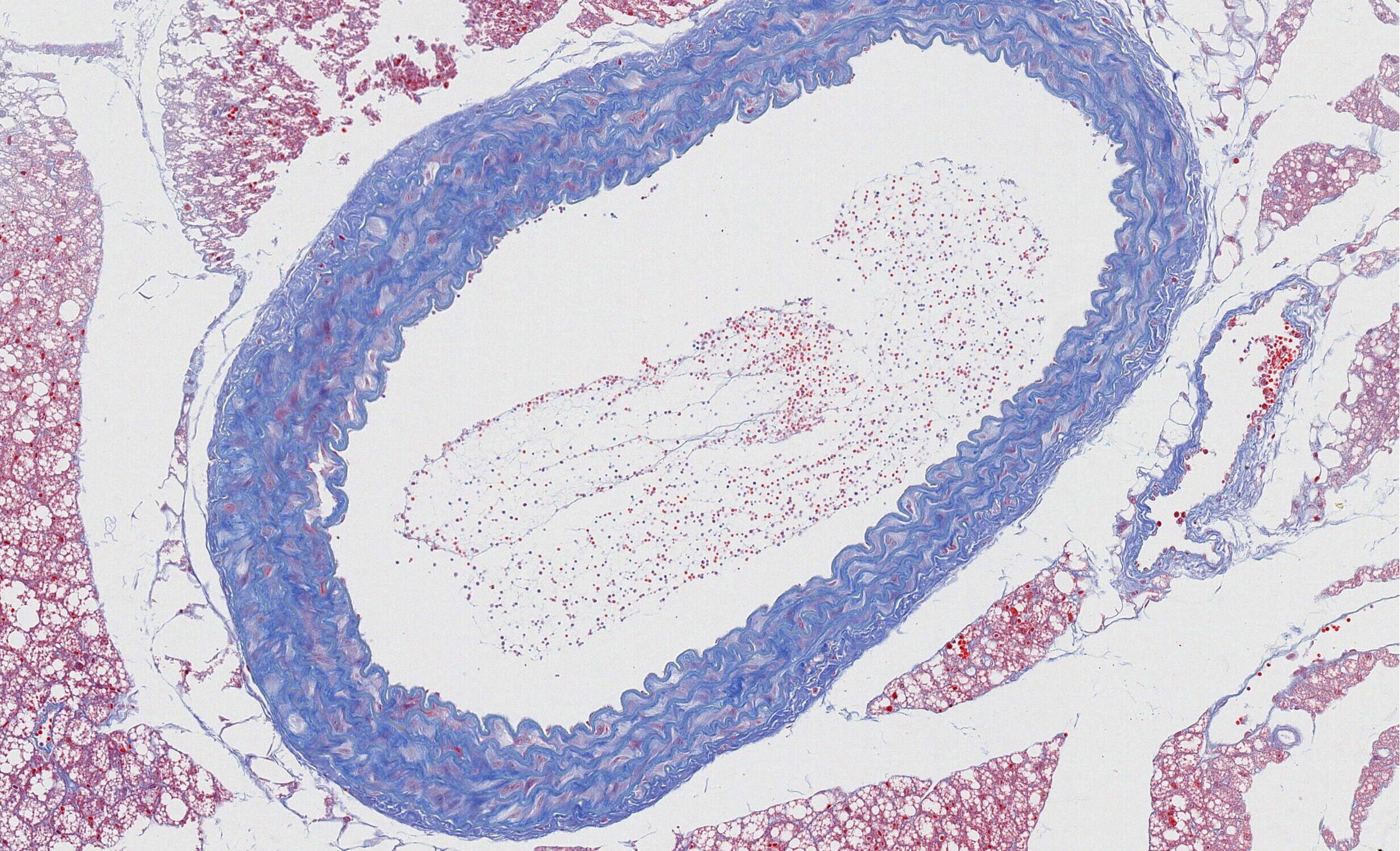

Supplement: Supplementary file 8 — Source data Fig. 3 [file 44318_2026_832_MOESM8_ESM.zip › P/WT old+5ht aorta.jpg]

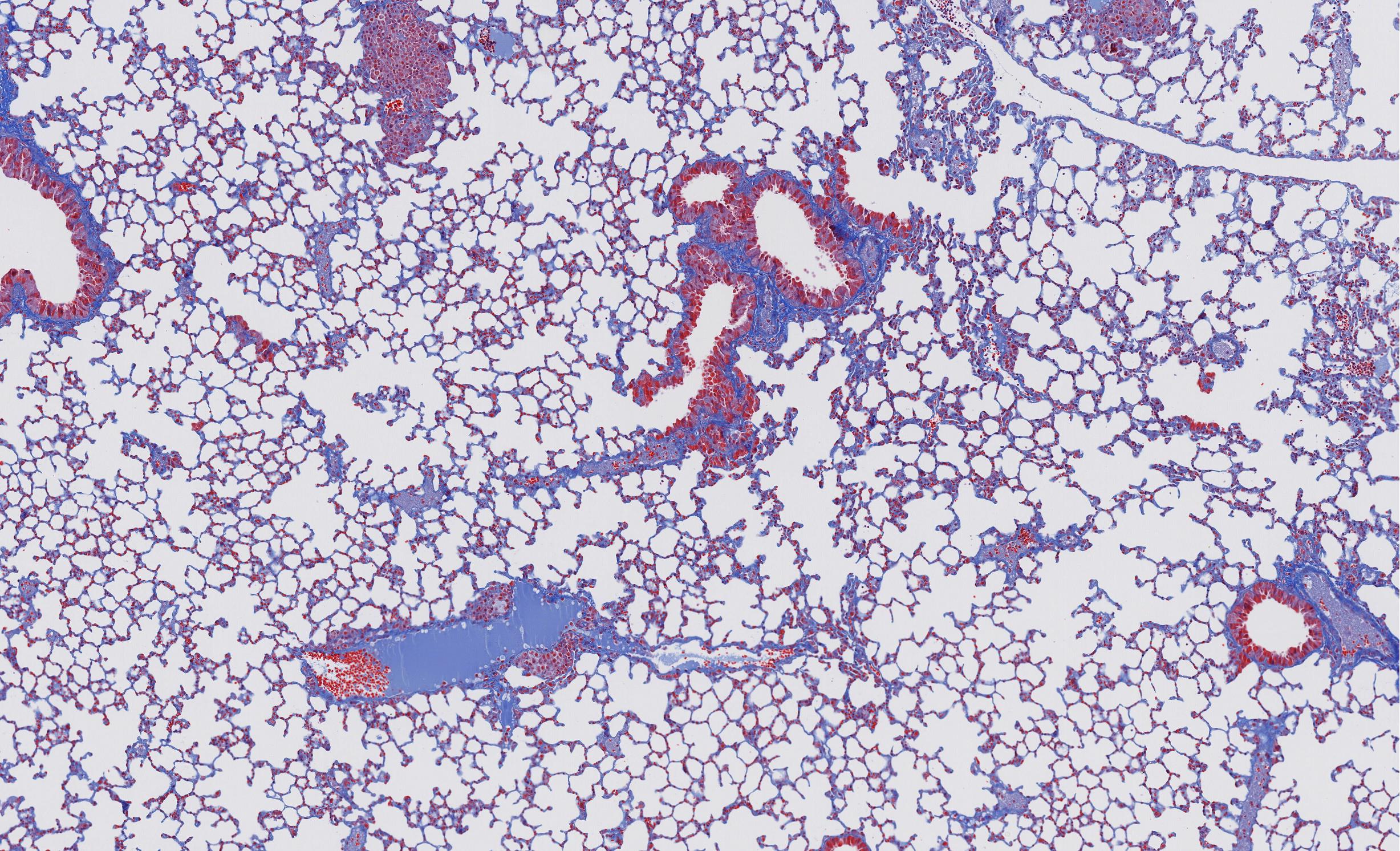

Supplement: Supplementary file 8 — Source data Fig. 3 [file 44318_2026_832_MOESM8_ESM.zip › P/WT old+5ht lung.jpg]

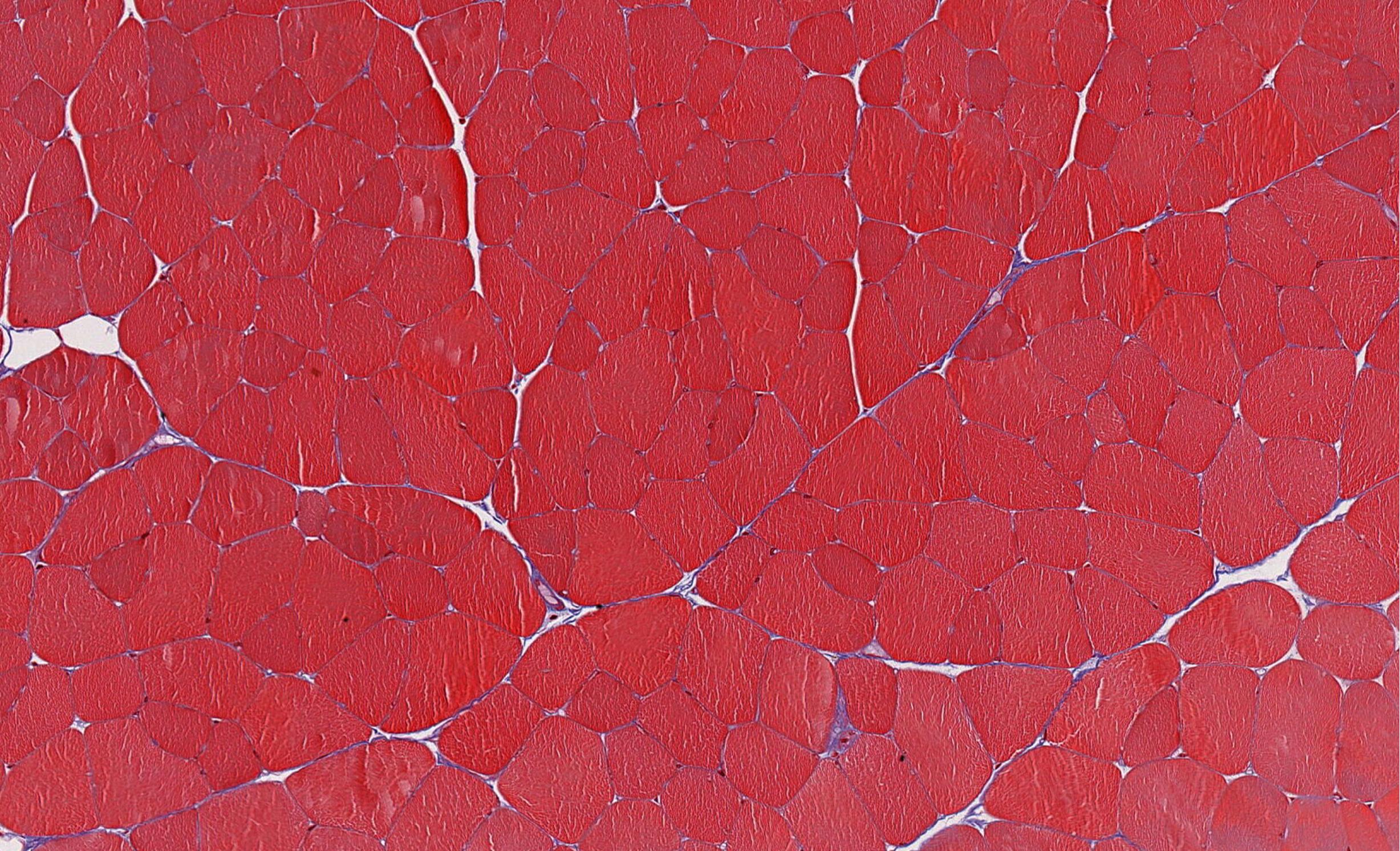

Supplement: Supplementary file 8 — Source data Fig. 3 [file 44318_2026_832_MOESM8_ESM.zip › P/WT old+5ht muscle.jpg]

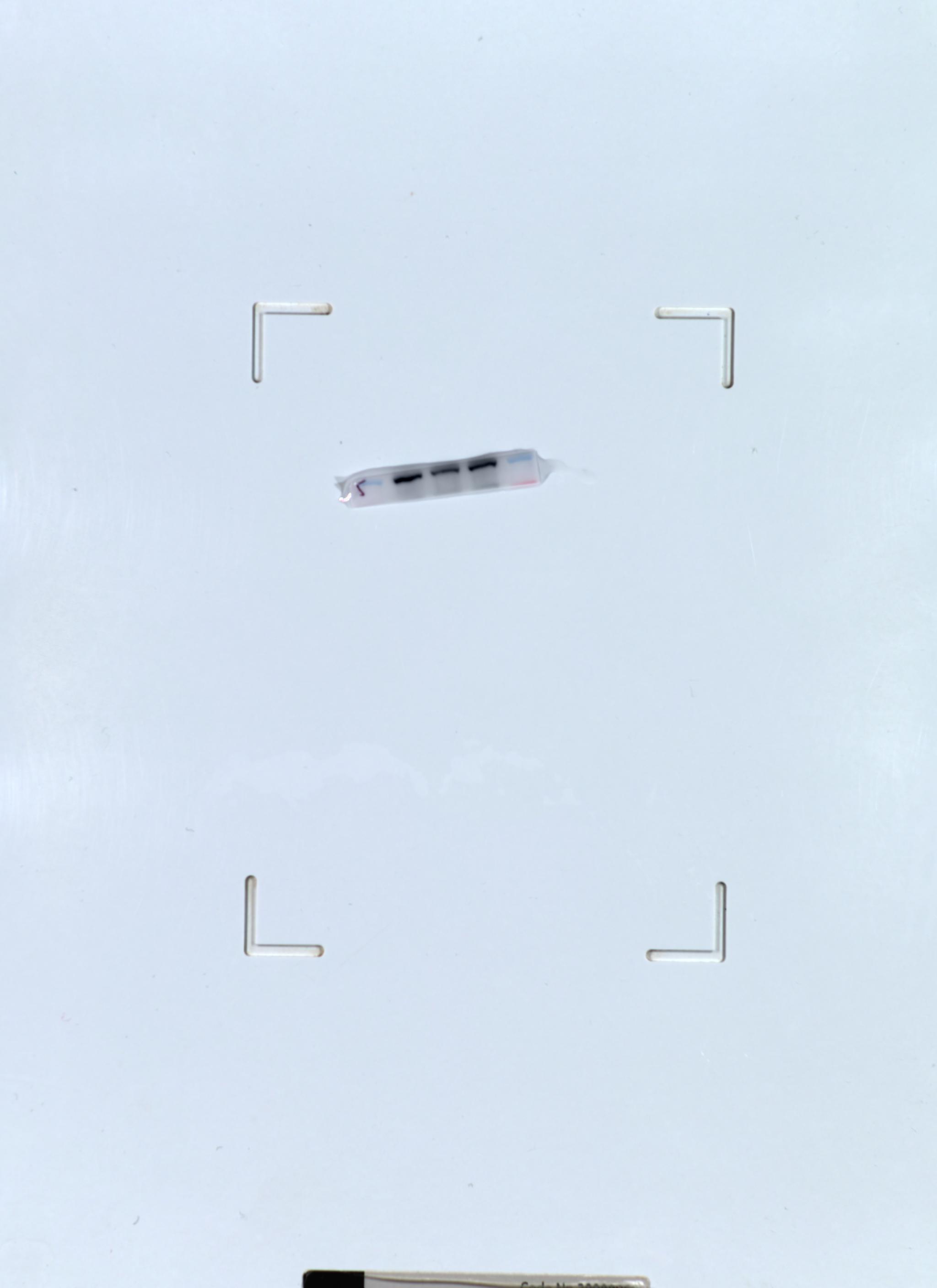

Supplement: Supplementary file 9 — Source data Fig. 4 [file 44318_2026_832_MOESM9_ESM.zip › C/PGC1a+Marker.jpg]

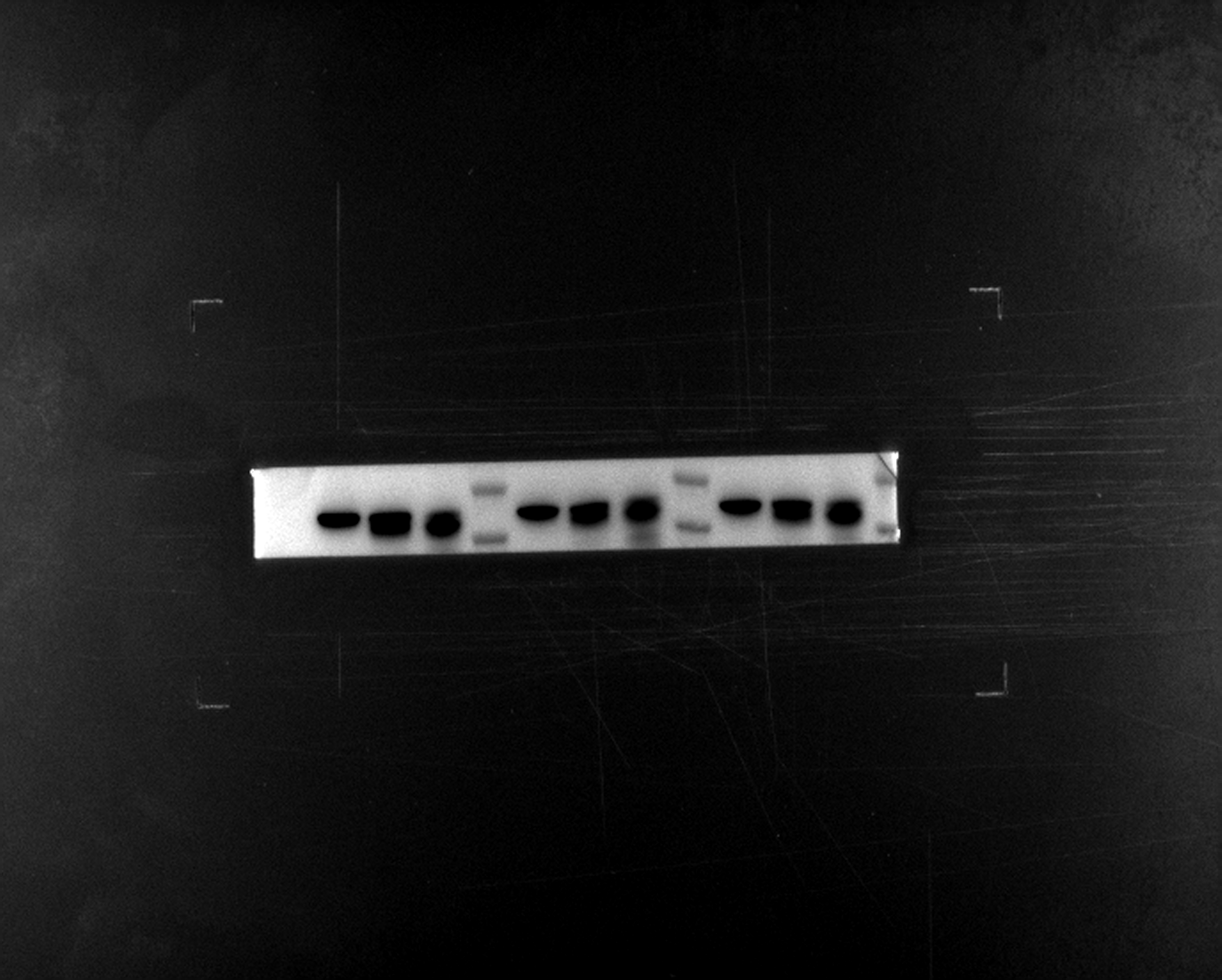

Supplement: Supplementary file 9 — Source data Fig. 4 [file 44318_2026_832_MOESM9_ESM.zip › C/replicate/5-1-ACTIN-3.tif]

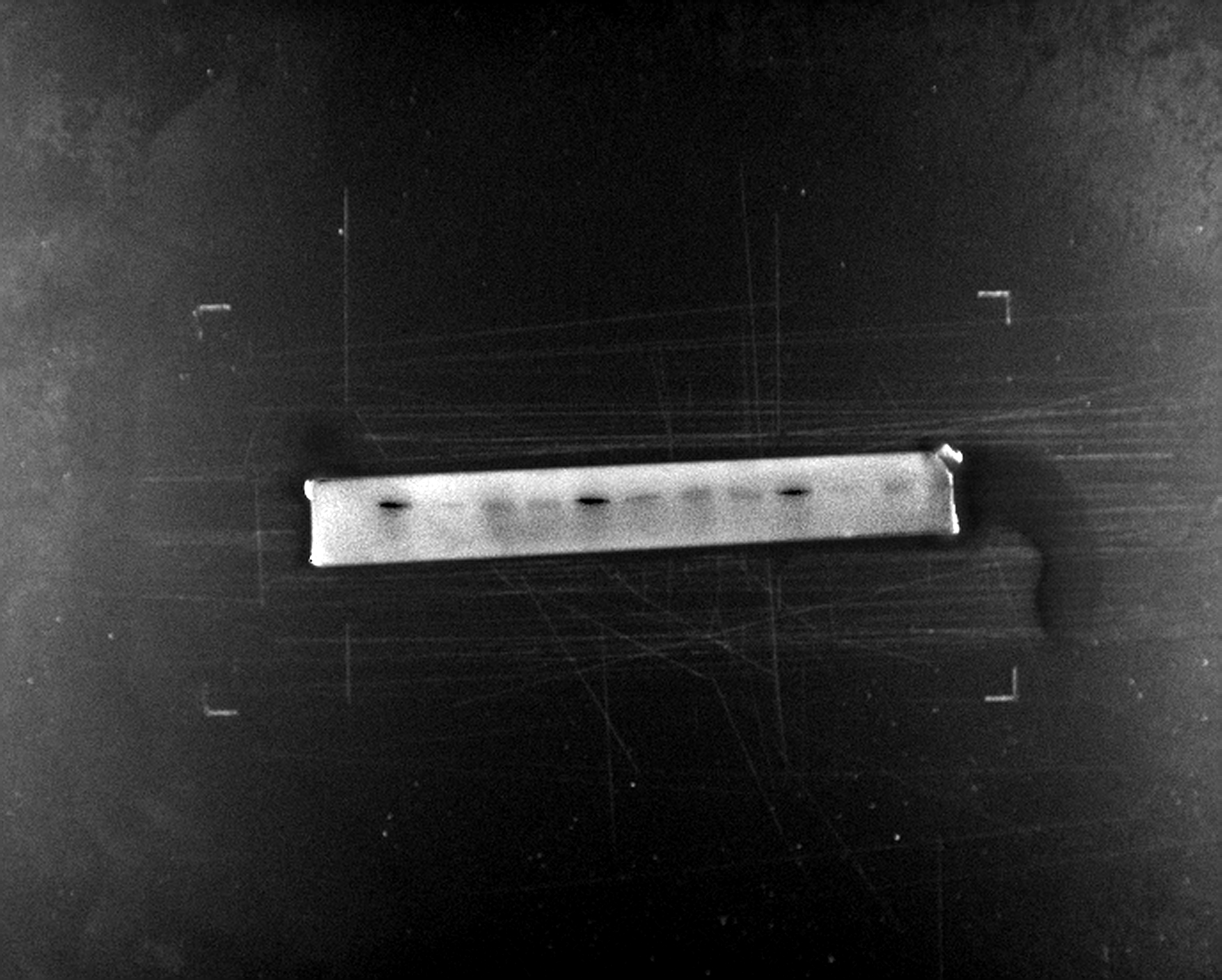

Supplement: Supplementary file 9 — Source data Fig. 4 [file 44318_2026_832_MOESM9_ESM.zip › C/replicate/5-1-PGC1A-5-3.tif]

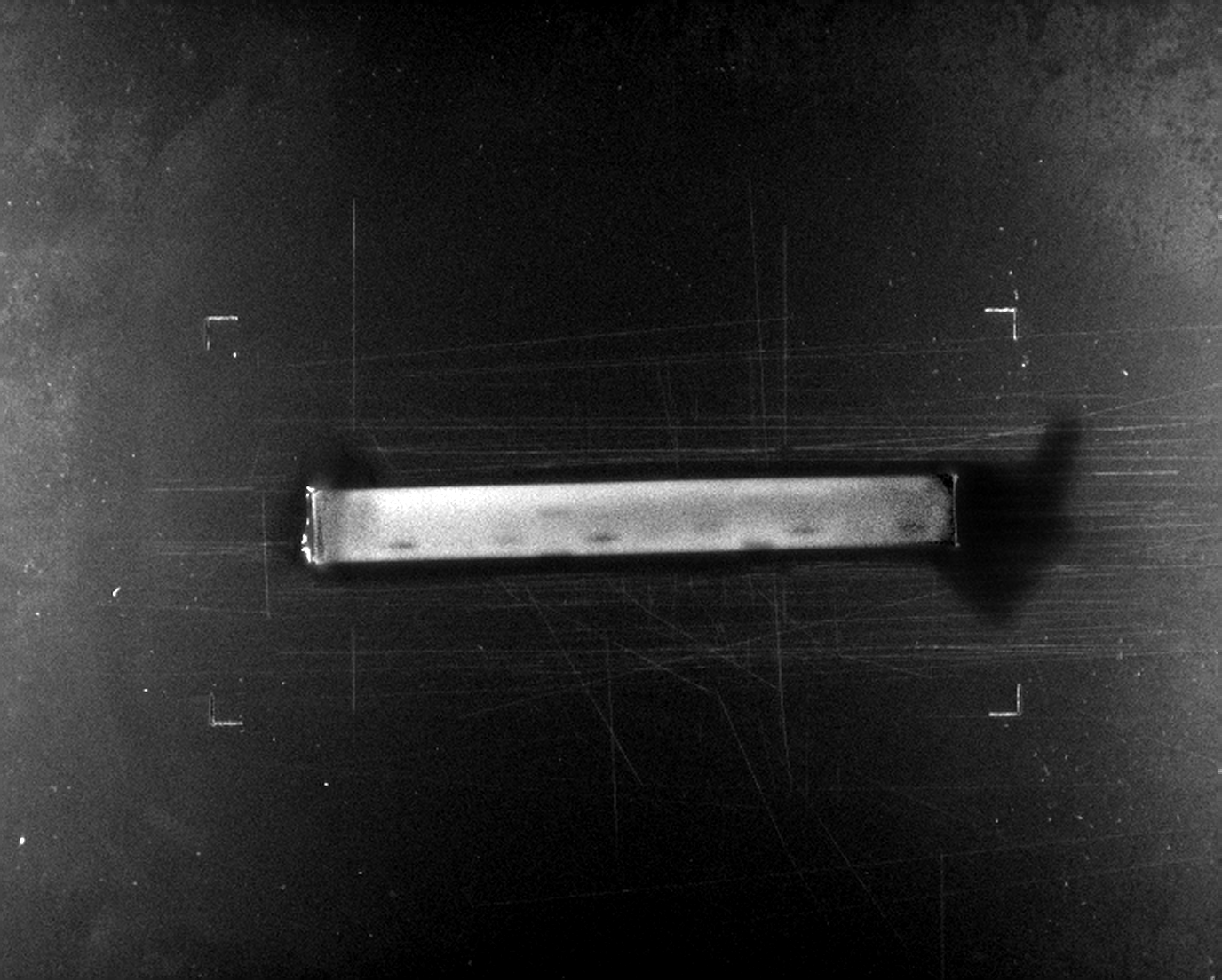

Supplement: Supplementary file 9 — Source data Fig. 4 [file 44318_2026_832_MOESM9_ESM.zip › C/replicate/5-1-TIMM23-4-3.tif]

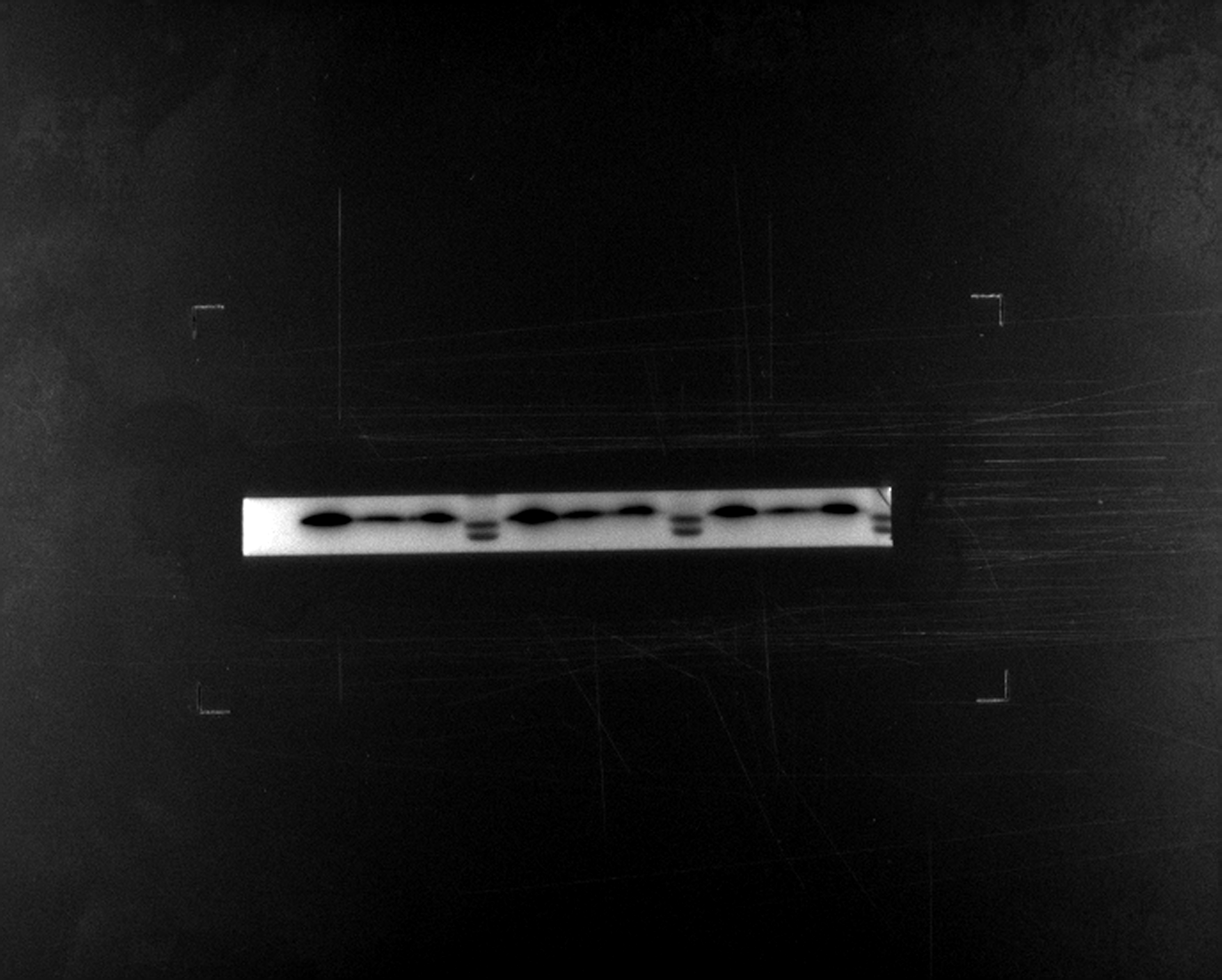

Supplement: Supplementary file 9 — Source data Fig. 4 [file 44318_2026_832_MOESM9_ESM.zip › C/replicate/5-1-TOMM20-3.tif]

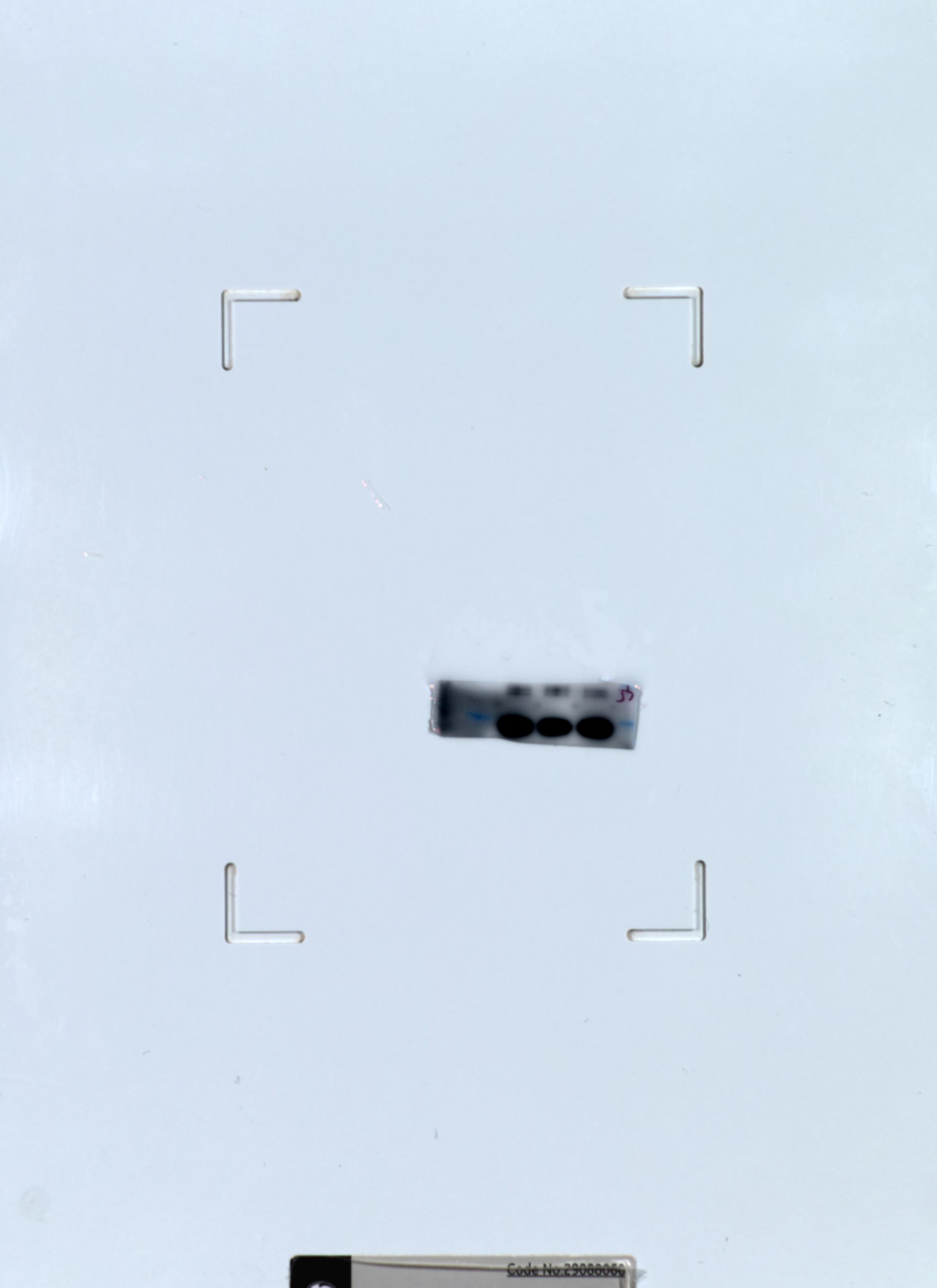

Supplement: Supplementary file 9 — Source data Fig. 4 [file 44318_2026_832_MOESM9_ESM.zip › C/TIMM23+marker.jpg]

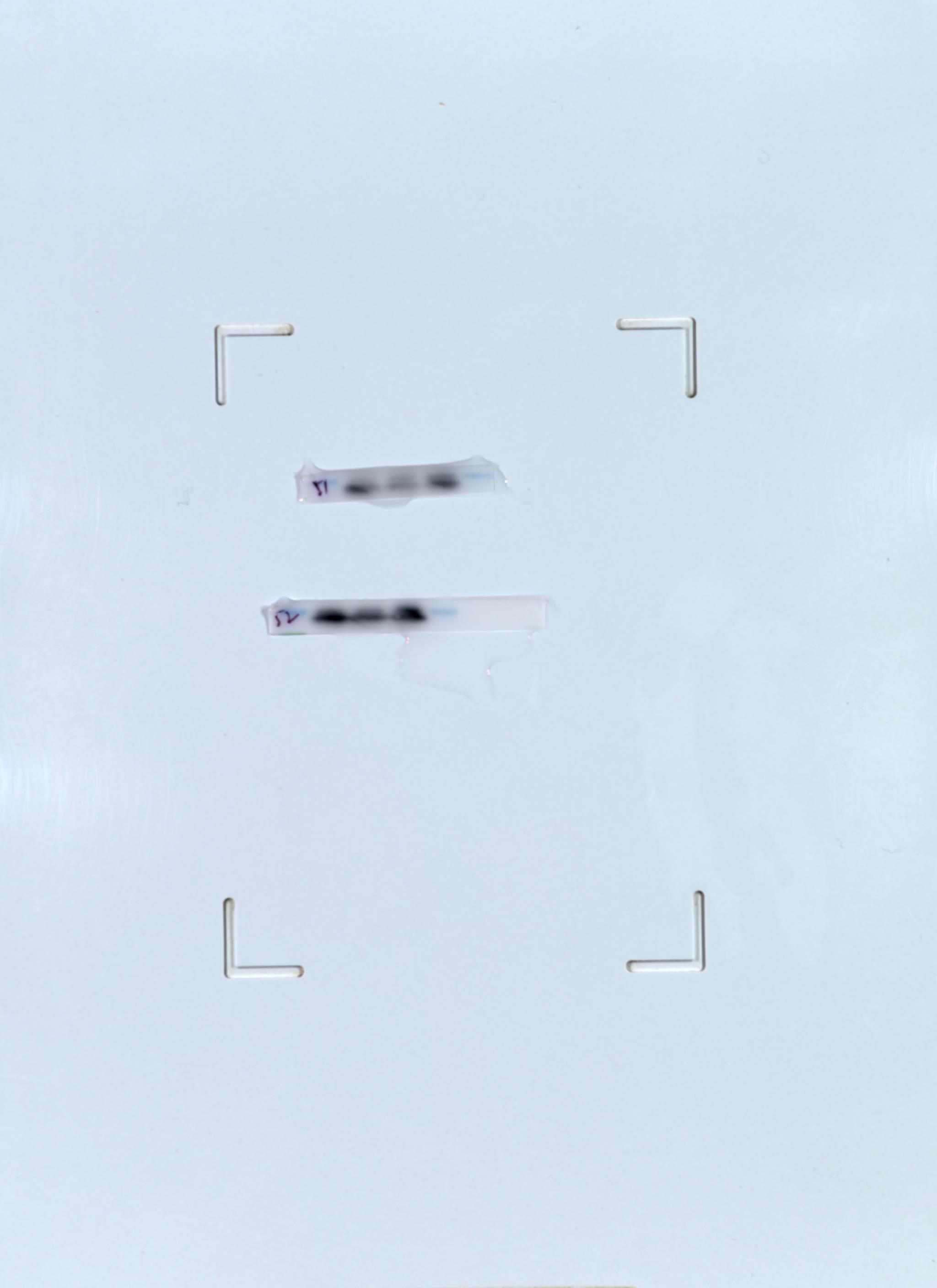

Supplement: Supplementary file 9 — Source data Fig. 4 [file 44318_2026_832_MOESM9_ESM.zip › C/TOMM20+Marker.jpg]

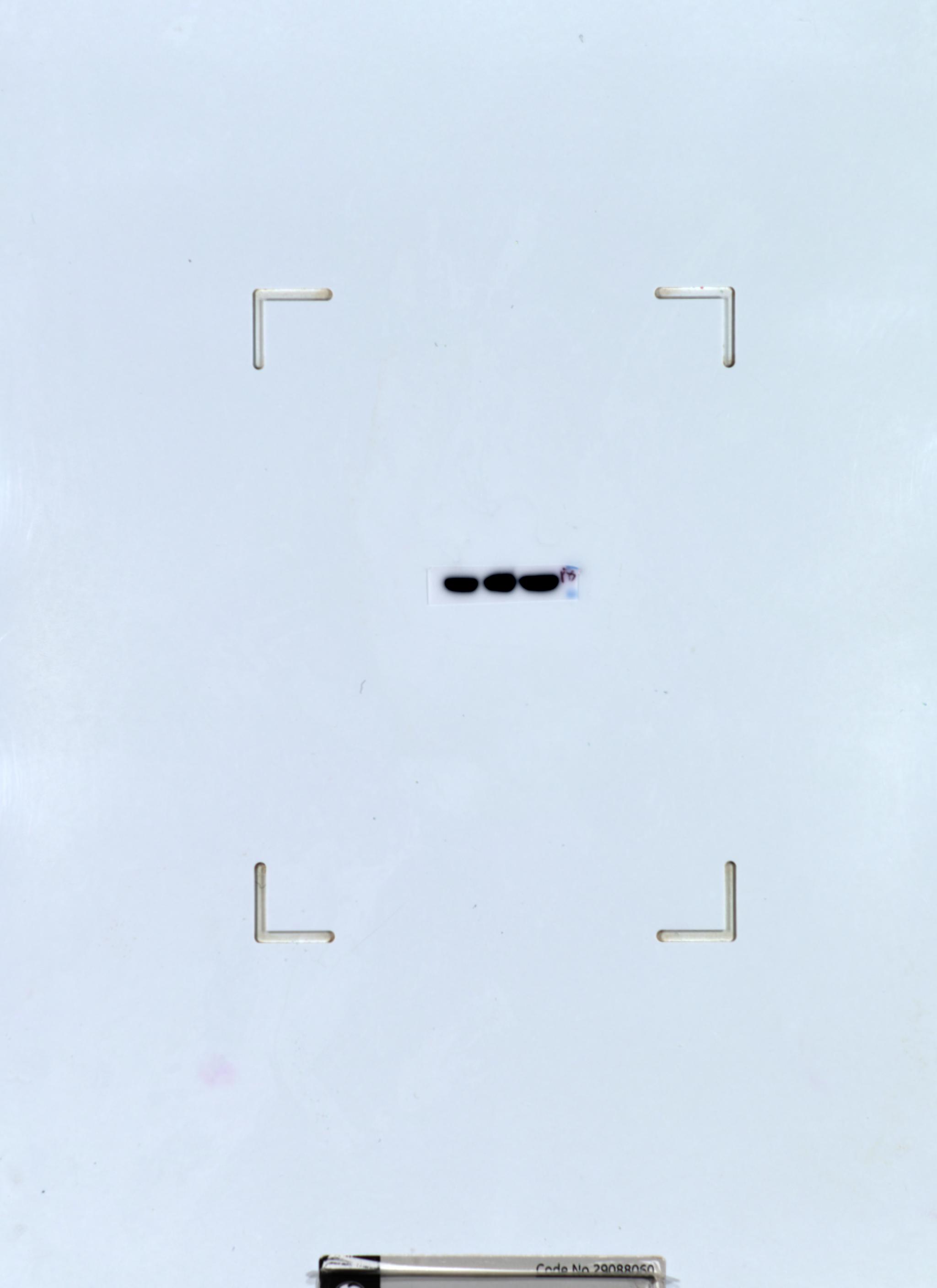

Supplement: Supplementary file 9 — Source data Fig. 4 [file 44318_2026_832_MOESM9_ESM.zip › C/β-actin+marker.jpg]

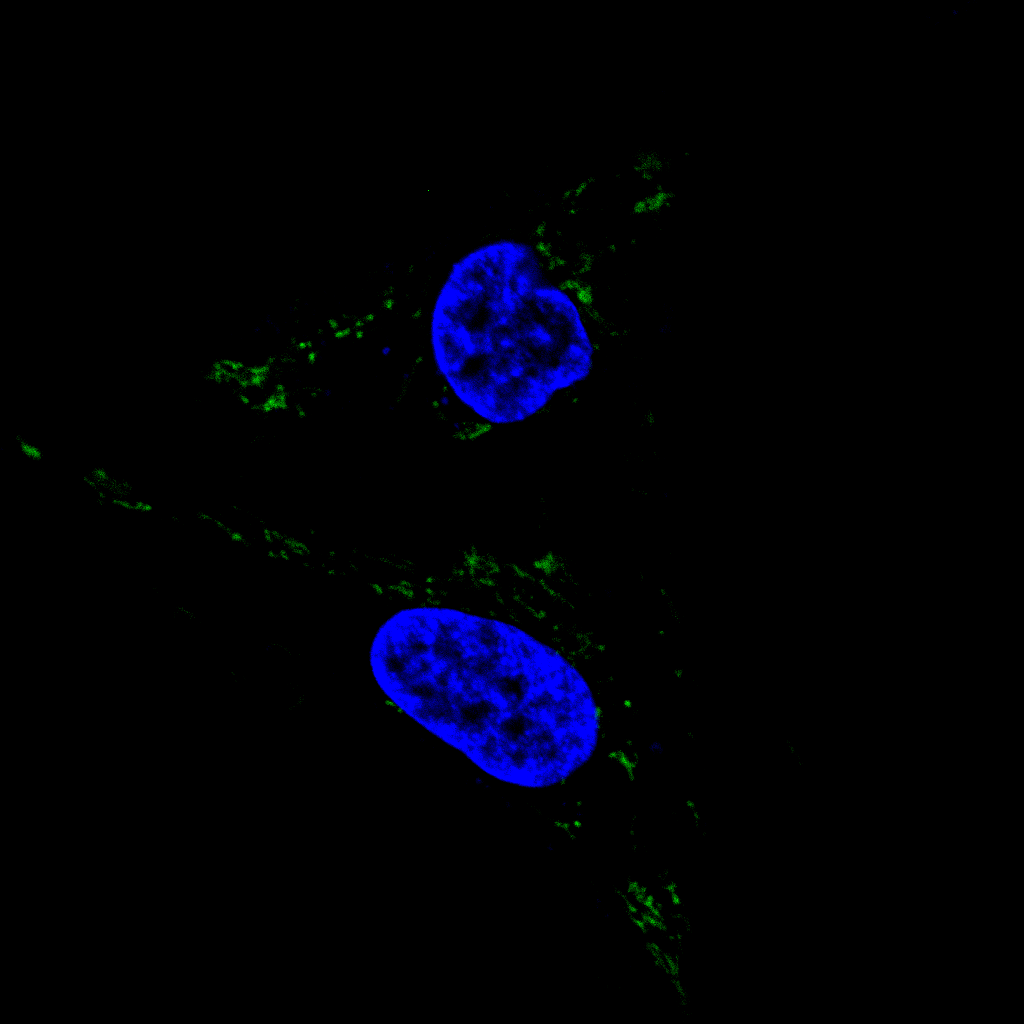

Supplement: Supplementary file 9 — Source data Fig. 4 [file 44318_2026_832_MOESM9_ESM.zip › H/G608G merge.tif]

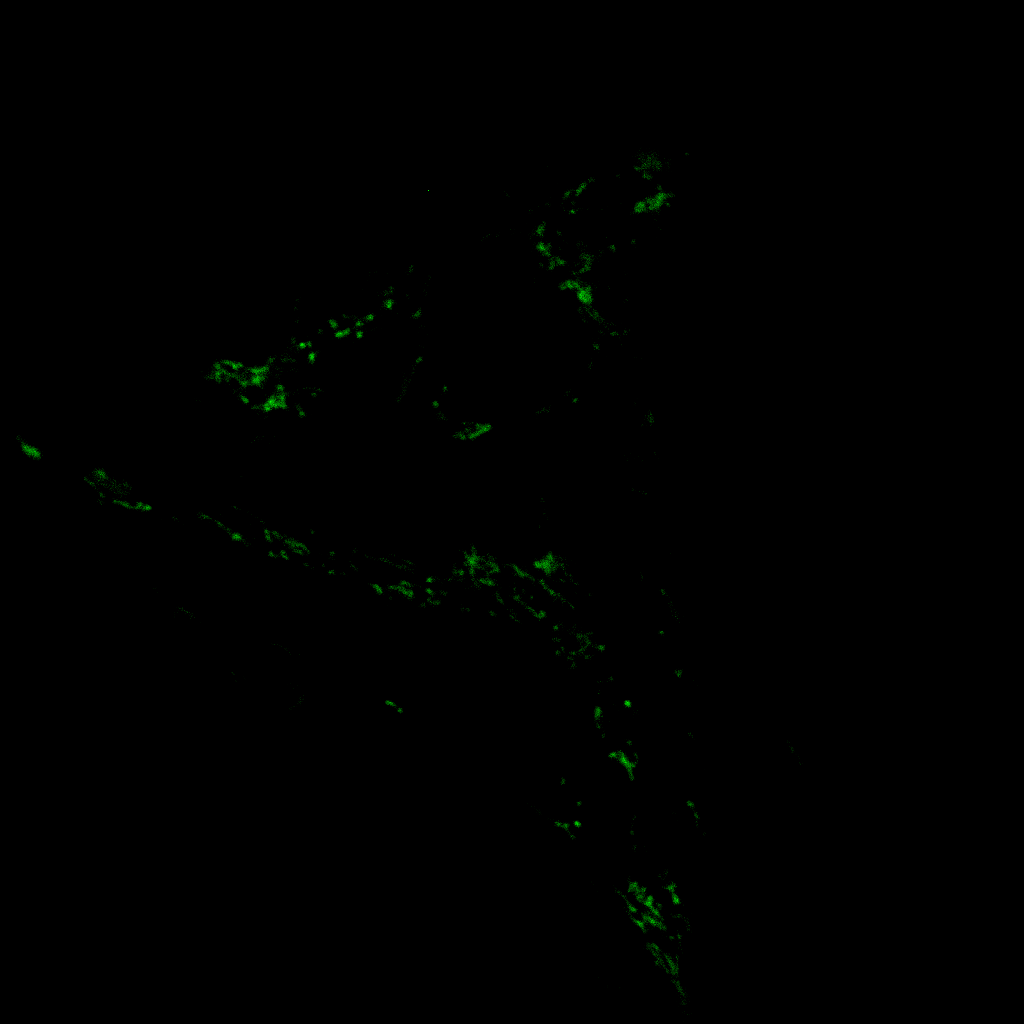

Supplement: Supplementary file 9 — Source data Fig. 4 [file 44318_2026_832_MOESM9_ESM.zip › H/G608G mitotracker.tif]

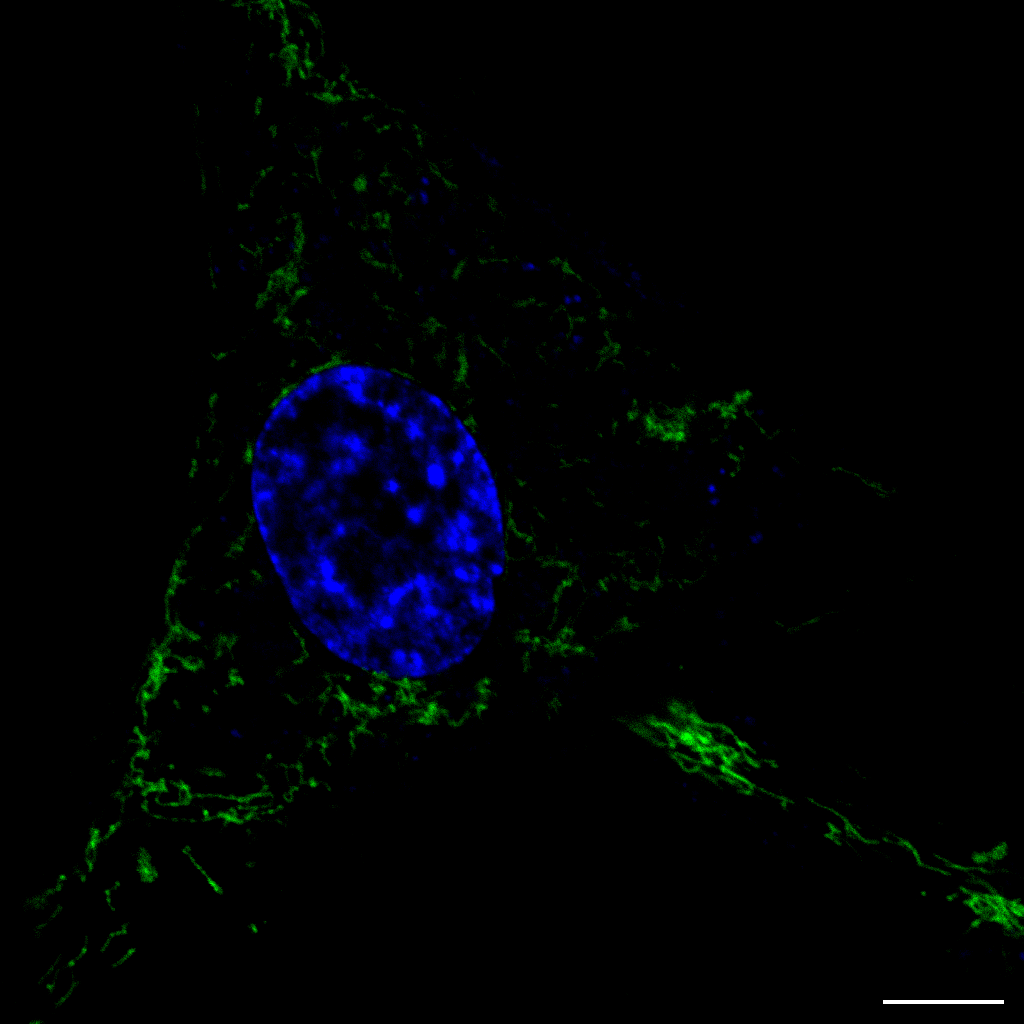

Supplement: Supplementary file 9 — Source data Fig. 4 [file 44318_2026_832_MOESM9_ESM.zip › H/G608G+5ht merge.gif]

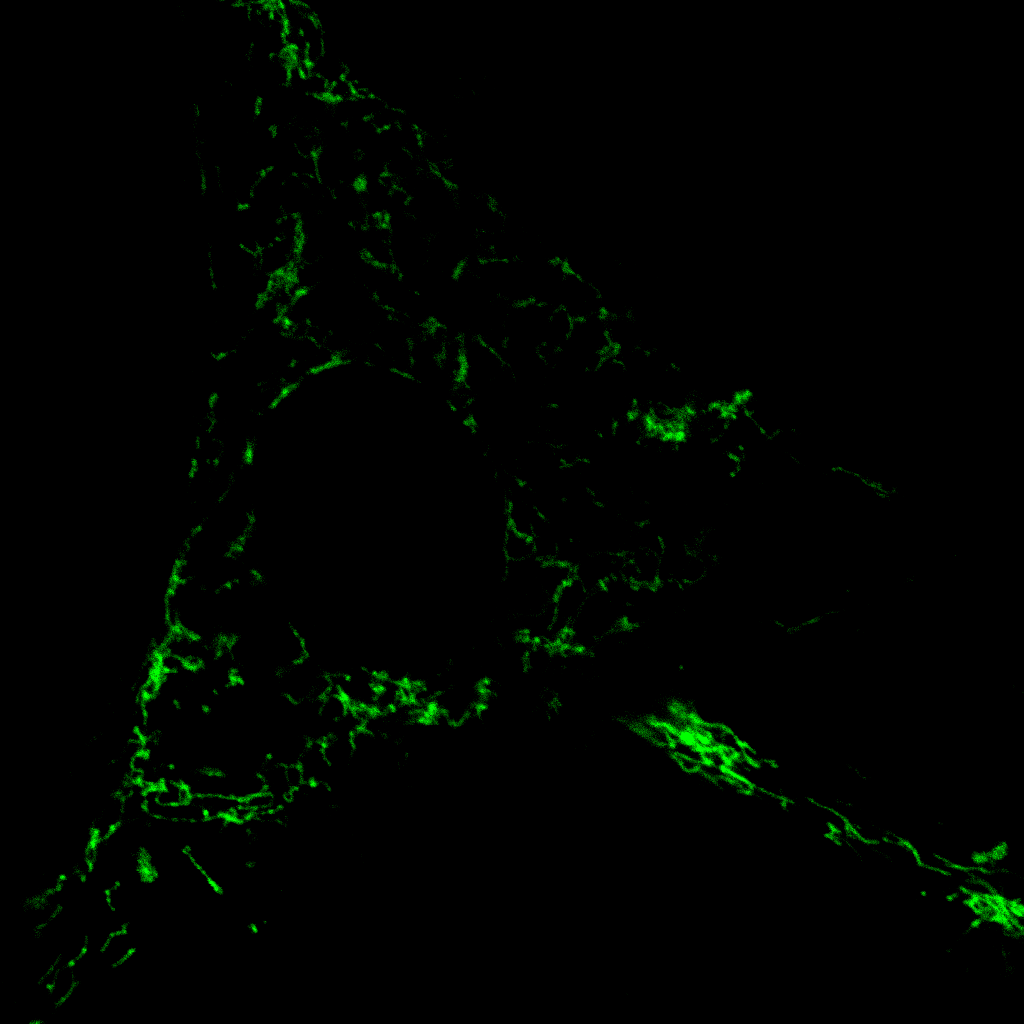

Supplement: Supplementary file 9 — Source data Fig. 4 [file 44318_2026_832_MOESM9_ESM.zip › H/G608G+5ht mitotracker.tif]

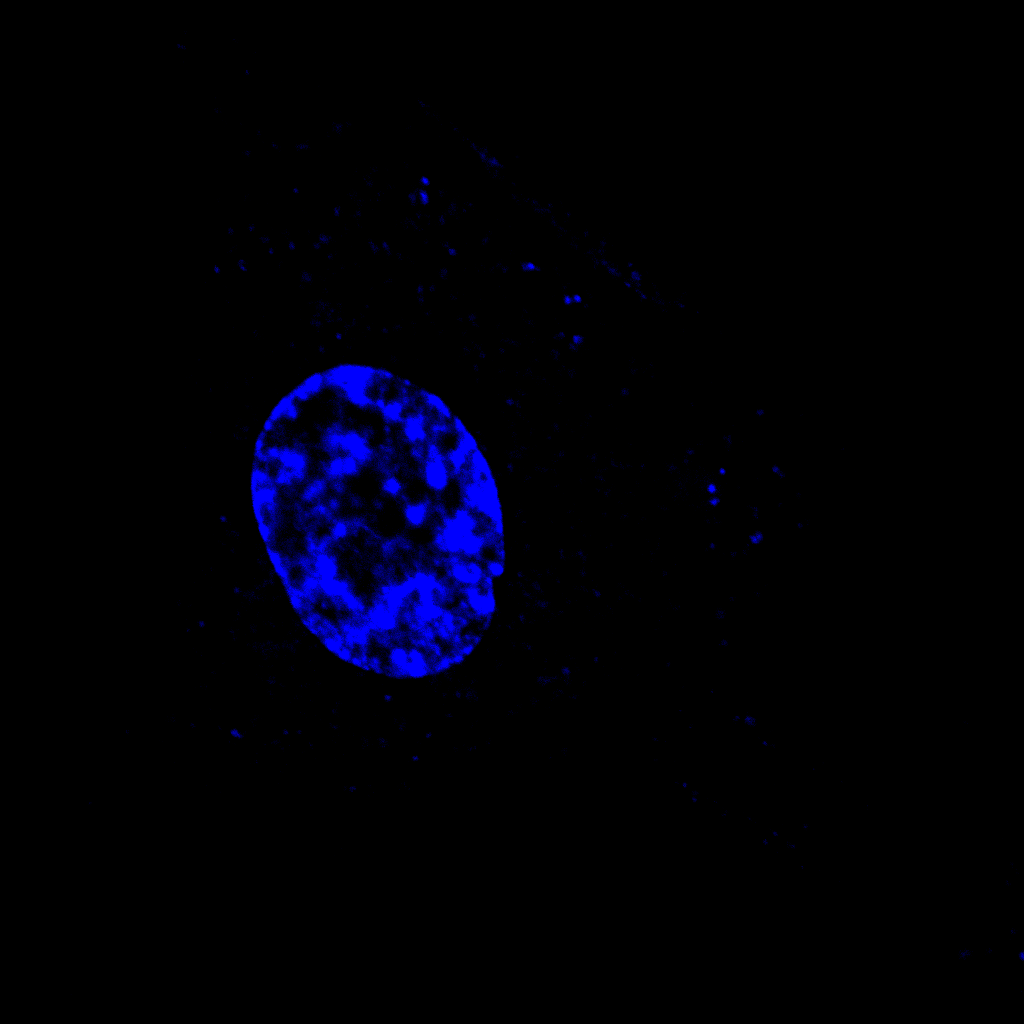

Supplement: Supplementary file 9 — Source data Fig. 4 [file 44318_2026_832_MOESM9_ESM.zip › H/G608G+5ht-DAPI.tif]

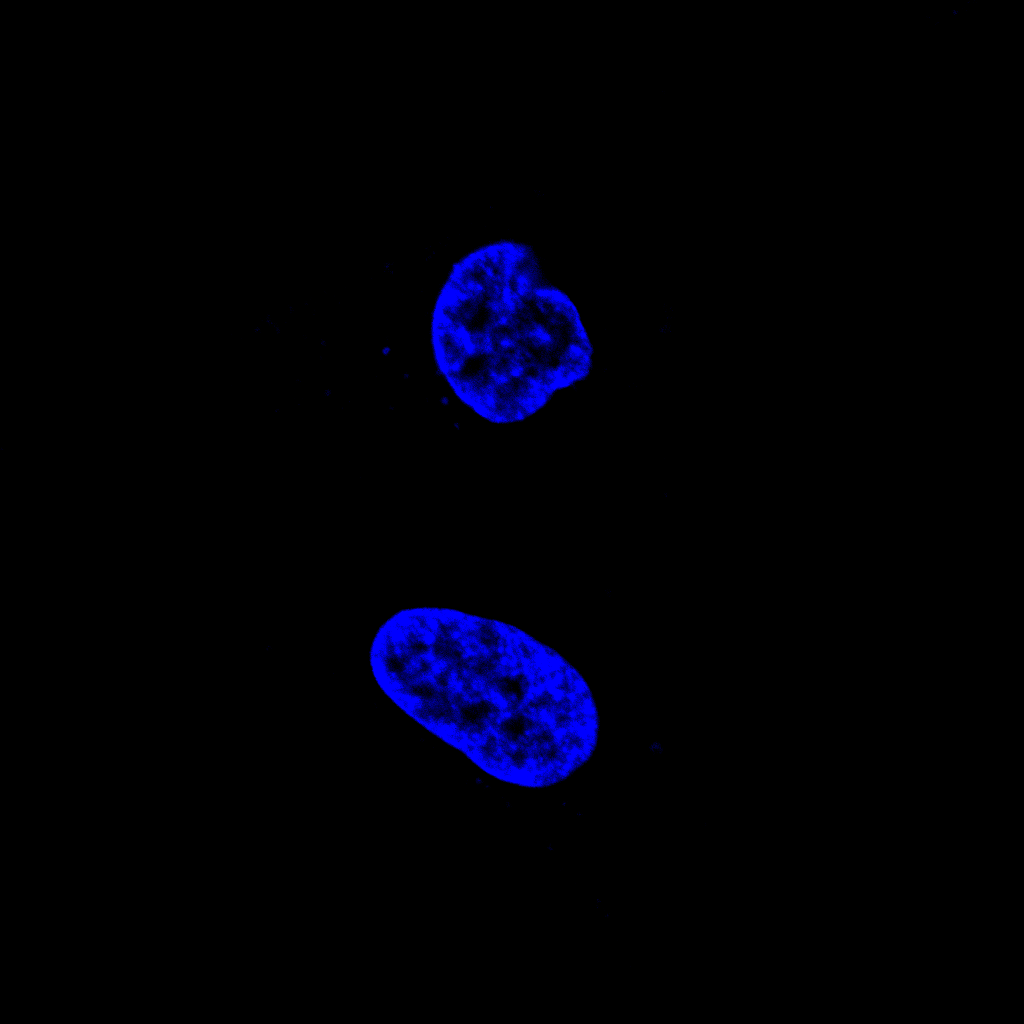

Supplement: Supplementary file 9 — Source data Fig. 4 [file 44318_2026_832_MOESM9_ESM.zip › H/G608G-DAPI.tif]

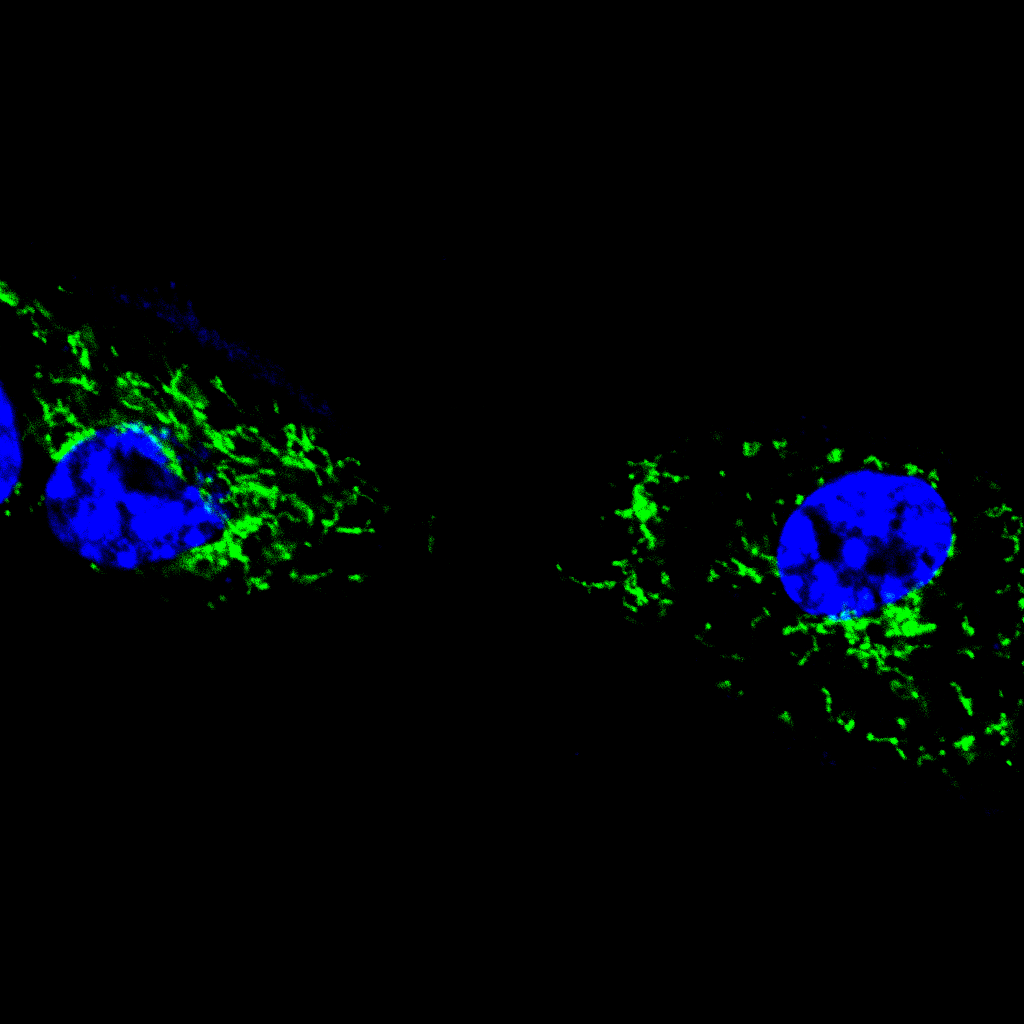

Supplement: Supplementary file 9 — Source data Fig. 4 [file 44318_2026_832_MOESM9_ESM.zip › H/WT merge.tif]

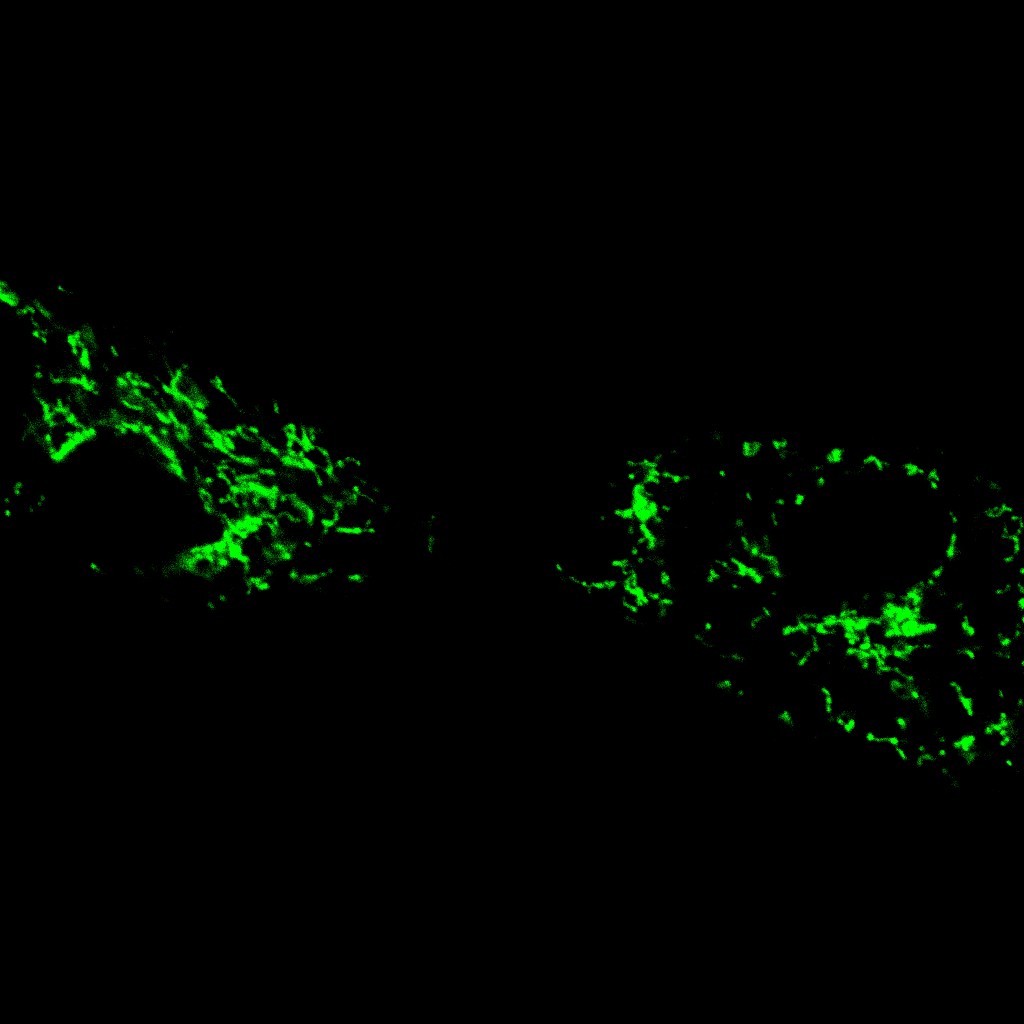

Supplement: Supplementary file 9 — Source data Fig. 4 [file 44318_2026_832_MOESM9_ESM.zip › H/WT mitotracker.tif]

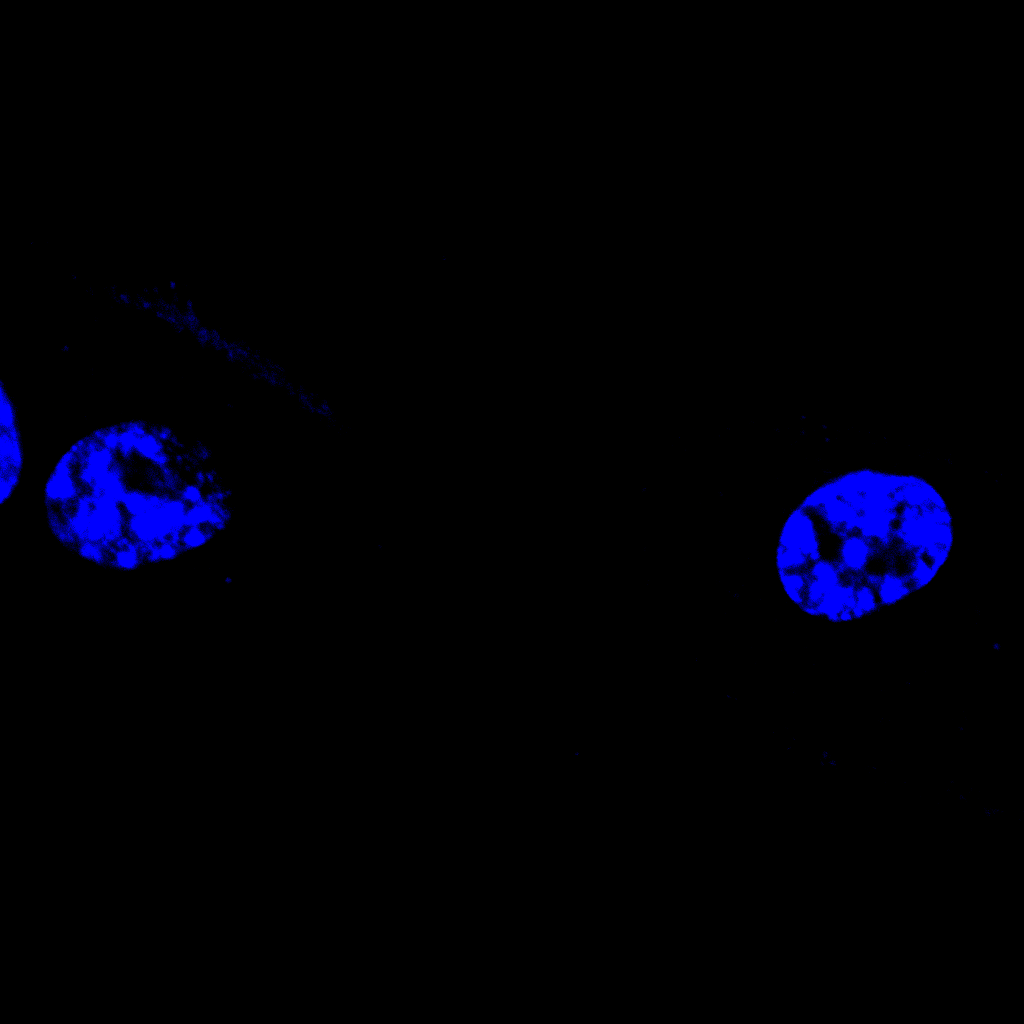

Supplement: Supplementary file 9 — Source data Fig. 4 [file 44318_2026_832_MOESM9_ESM.zip › H/WT-DAPI.tif]

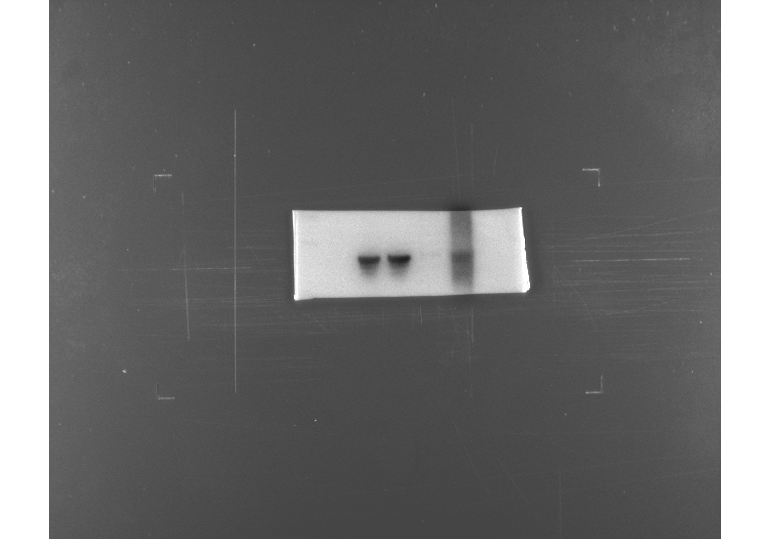

Supplement: Supplementary file 10 — Source data Fig. 5 [file 44318_2026_832_MOESM10_ESM.zip › E/HSP90β G608G.tif]

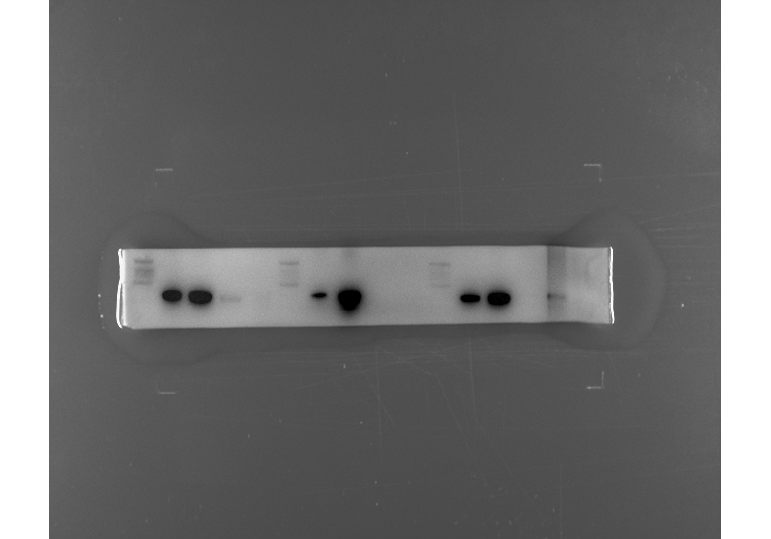

Supplement: Supplementary file 10 — Source data Fig. 5 [file 44318_2026_832_MOESM10_ESM.zip › E/HSP90β WT.tif]

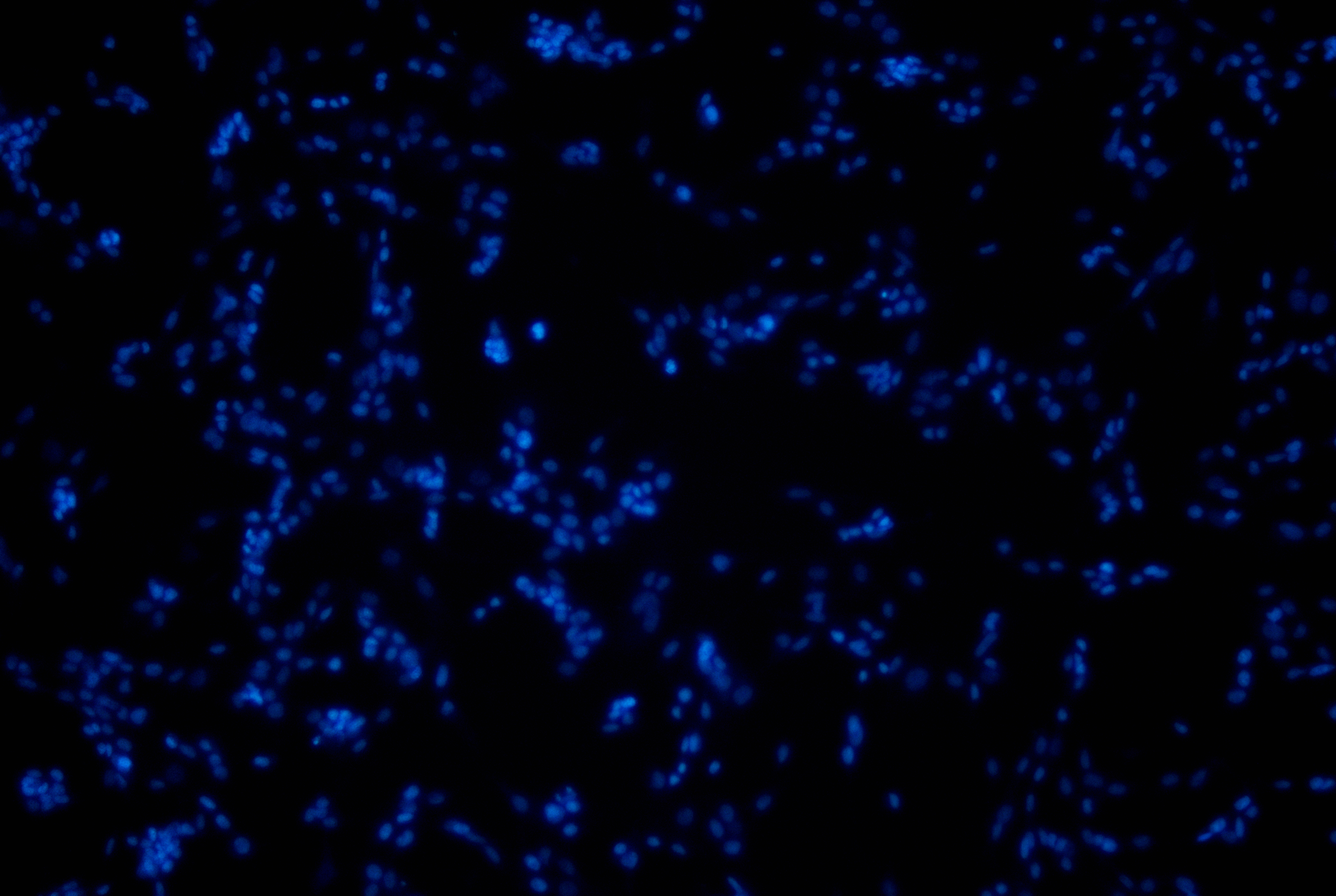

Supplement: Supplementary file 10 — Source data Fig. 5 [file 44318_2026_832_MOESM10_ESM.zip › G/G608G+5ht-DAPI.jpg]

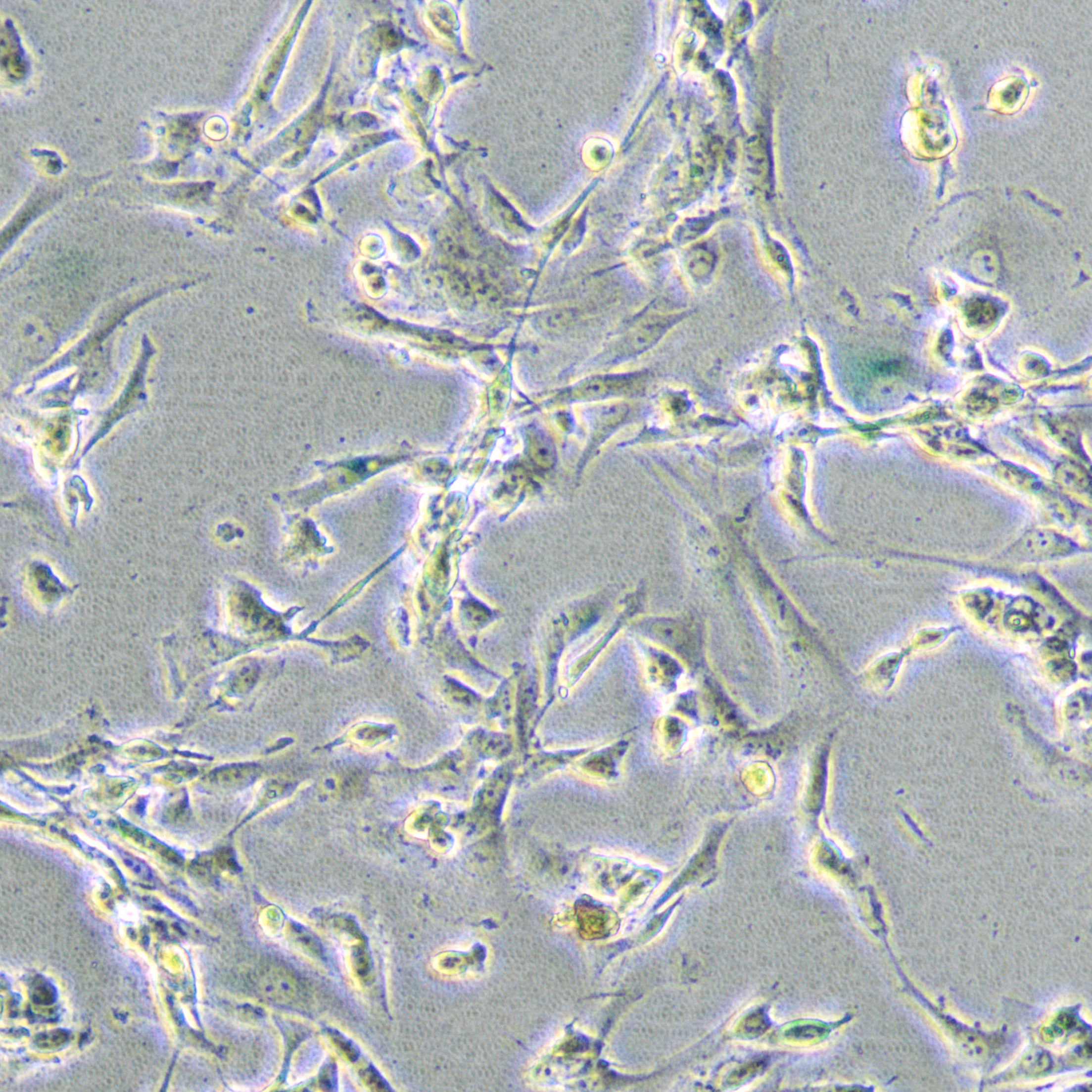

Supplement: Supplementary file 10 — Source data Fig. 5 [file 44318_2026_832_MOESM10_ESM.zip › G/G608G+5ht-large.tif]

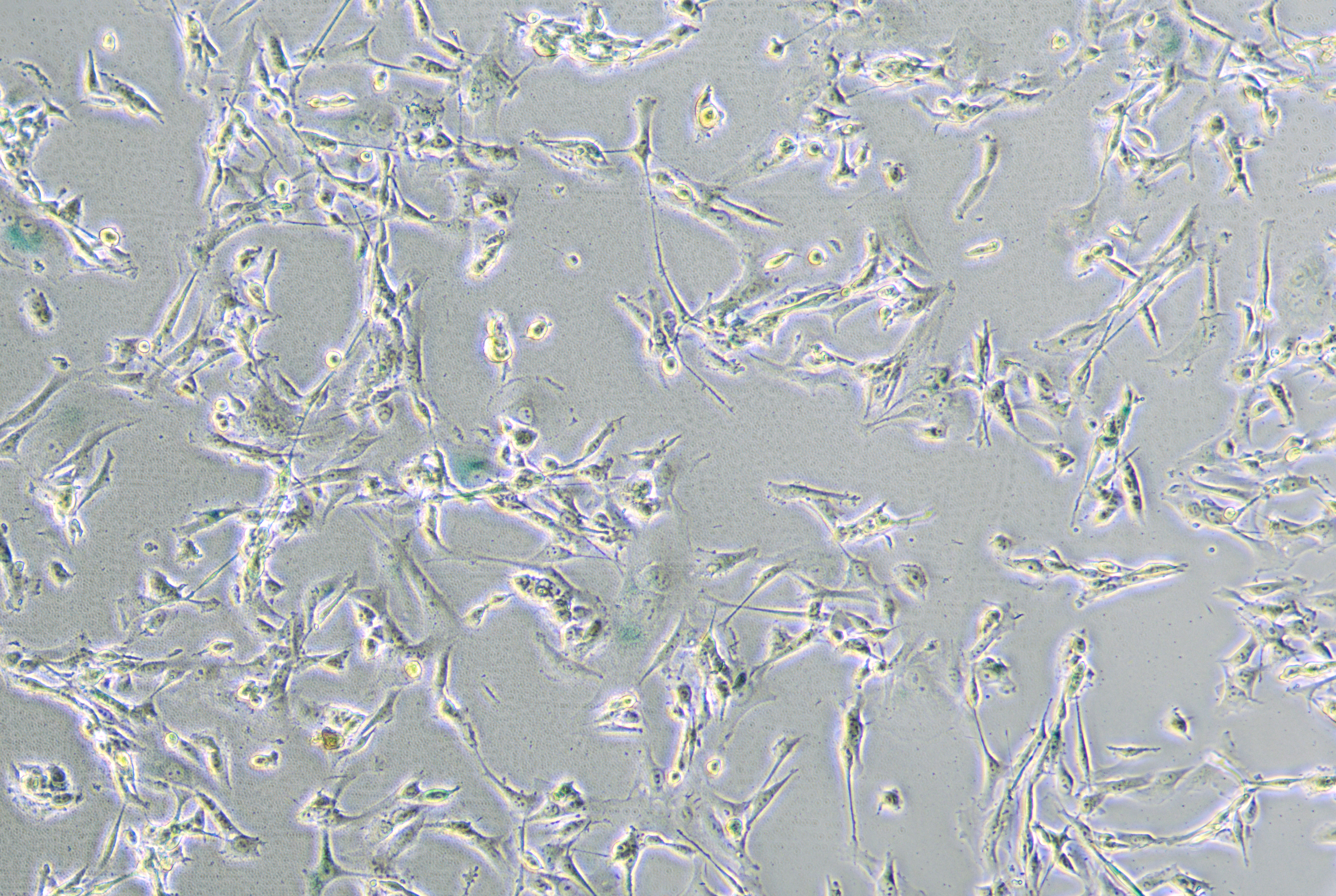

Supplement: Supplementary file 10 — Source data Fig. 5 [file 44318_2026_832_MOESM10_ESM.zip › G/G608G+5ht.jpg]

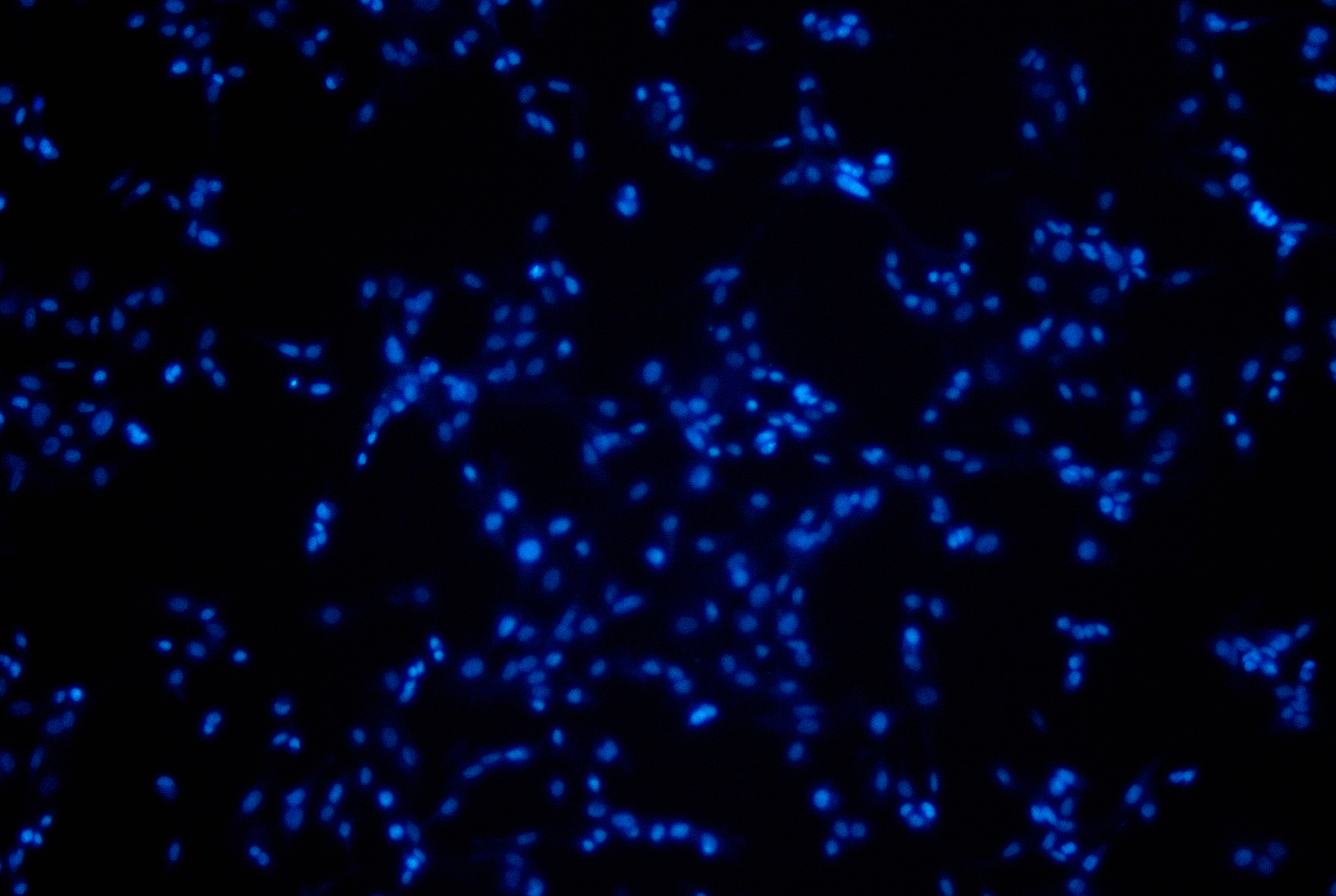

Supplement: Supplementary file 10 — Source data Fig. 5 [file 44318_2026_832_MOESM10_ESM.zip › G/G608G+LDN+5ht-DAPI.jpg]

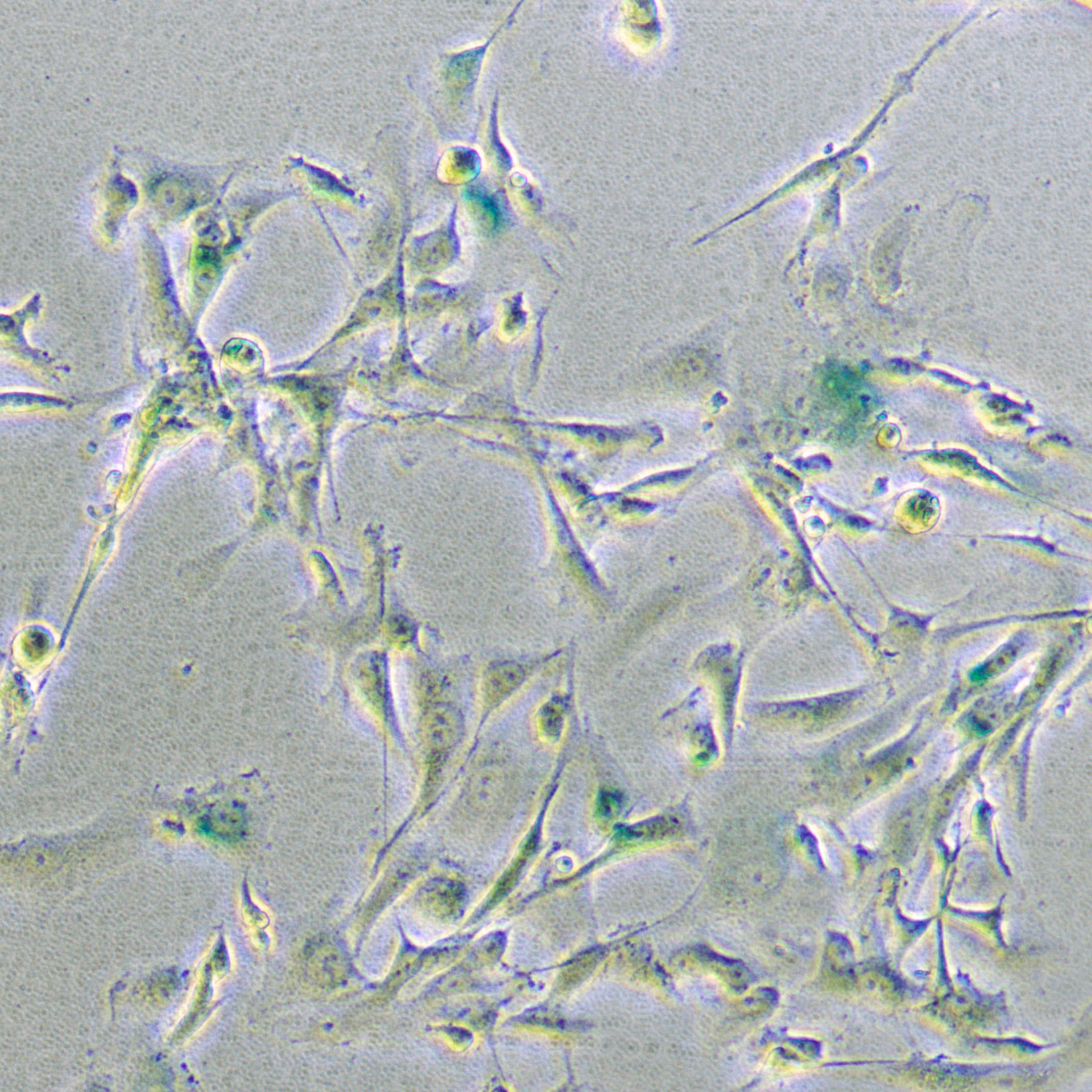

Supplement: Supplementary file 10 — Source data Fig. 5 [file 44318_2026_832_MOESM10_ESM.zip › G/G608G+LDN+5ht-large.tif]

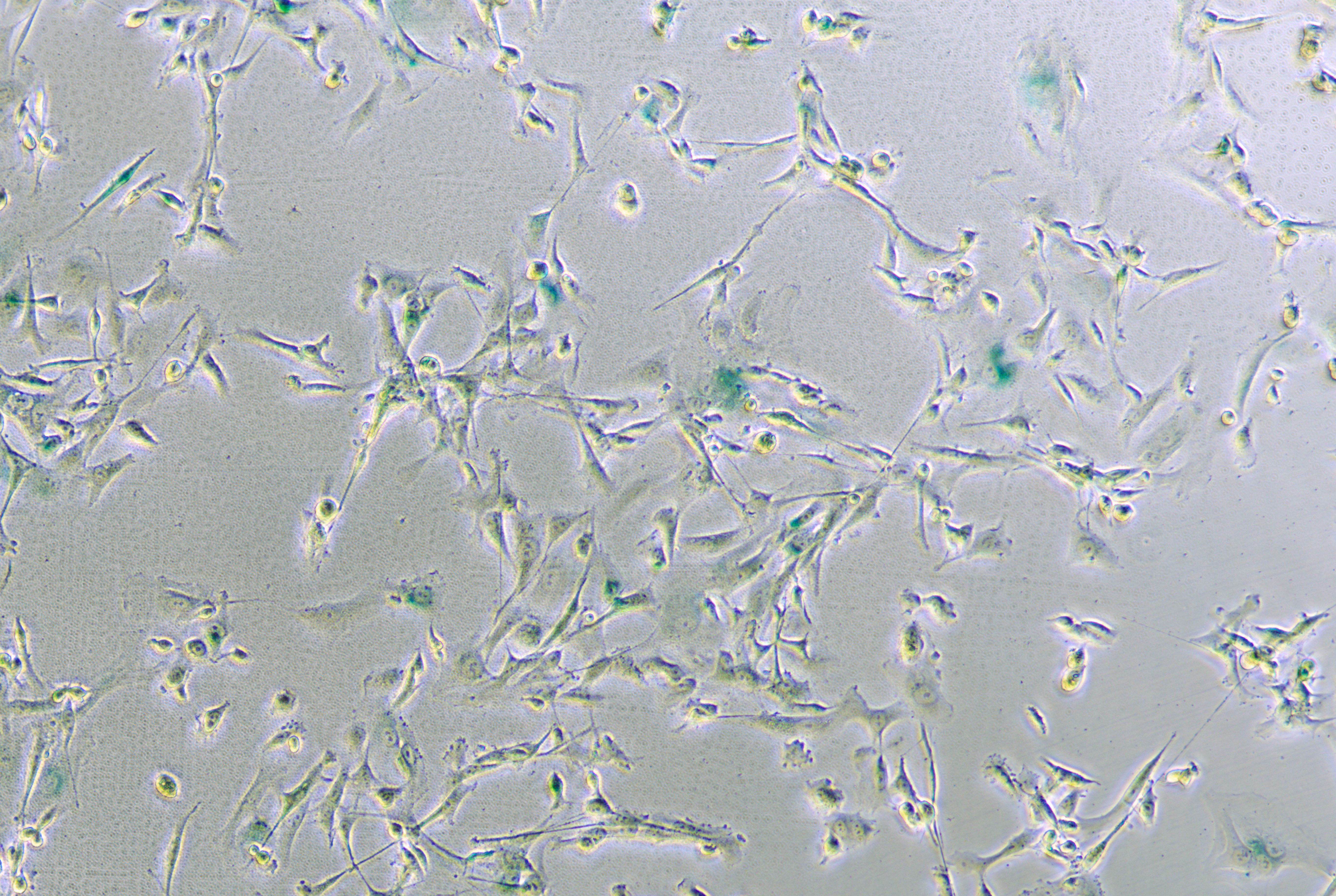

Supplement: Supplementary file 10 — Source data Fig. 5 [file 44318_2026_832_MOESM10_ESM.zip › G/G608G+LDN+5ht.jpg]
