# Supplementary figures and images for: The oocyte-enriched metabolite serotonin alleviates cellular senescence and aging phenotypes in the mouse (part 2 of 3)
Source: EMBO J. 2026 Jun 16;45(14):4849–86. doi: 10.1038/s44318-026-00832-x (PMC13373241; doi:10.1038/s44318-026-00832-x)

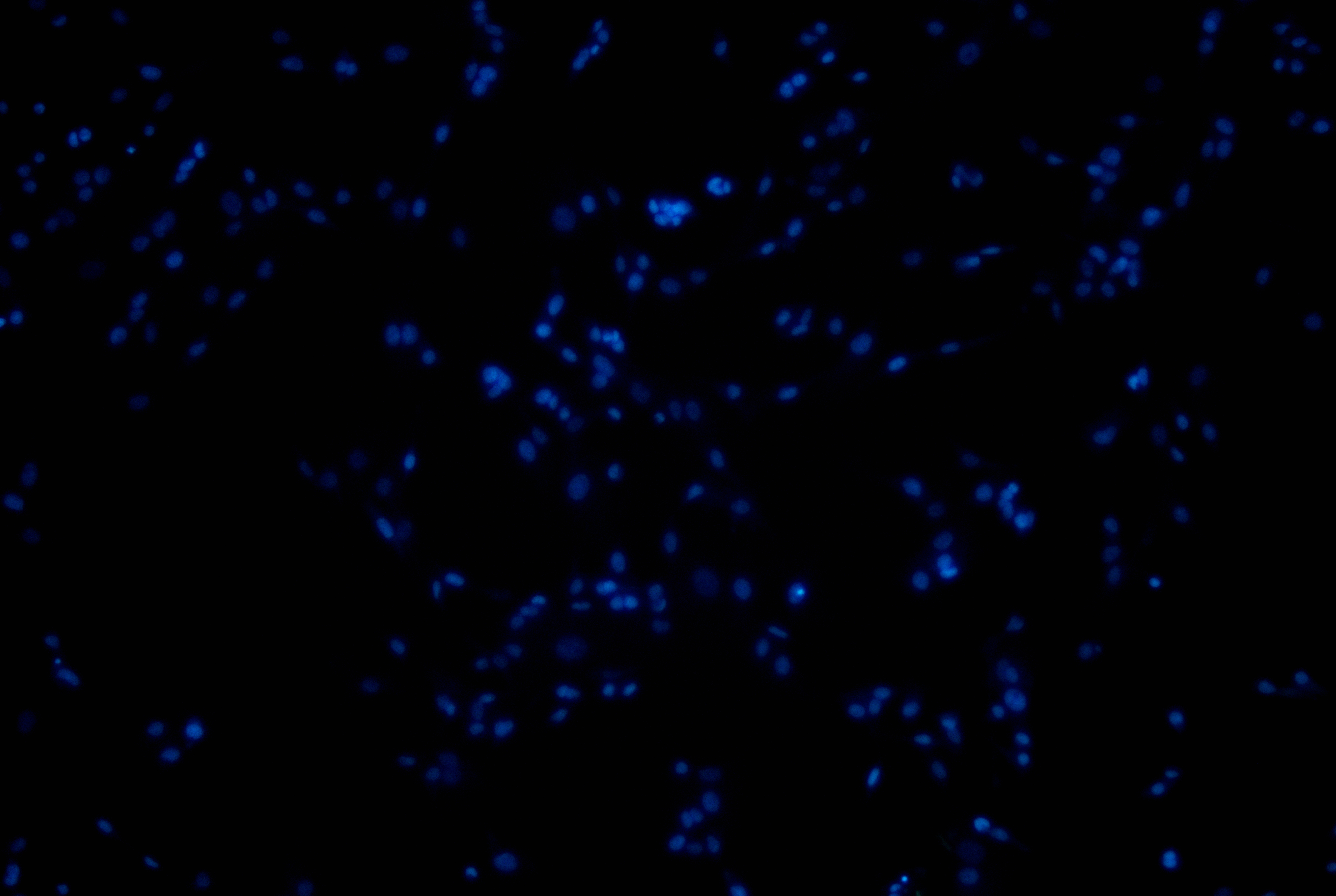

Supplement: Supplementary file 10 — Source data Fig. 5 [file 44318_2026_832_MOESM10_ESM.zip › G/G608G+LDN-DAPI.jpg]

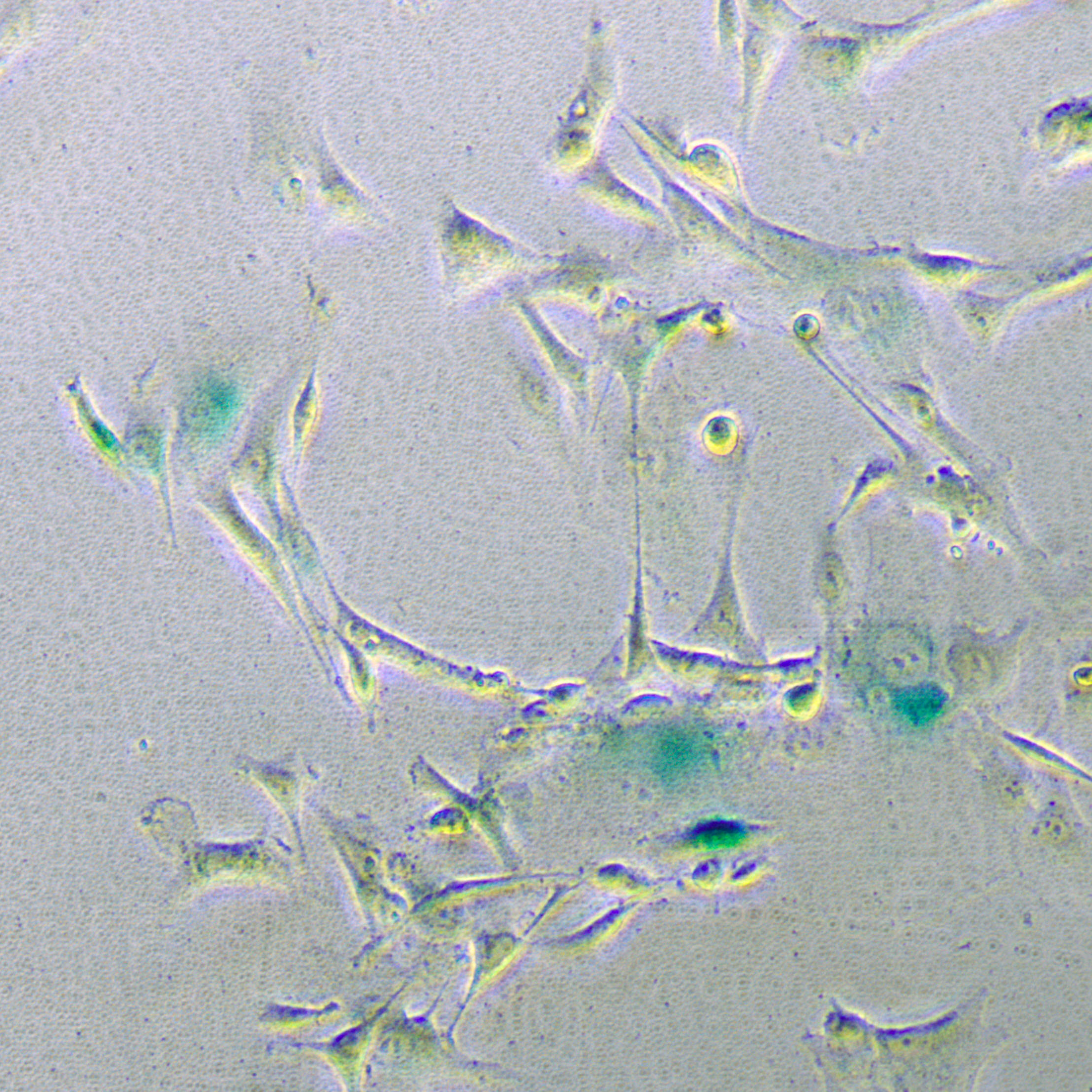

Supplement: Supplementary file 10 — Source data Fig. 5 [file 44318_2026_832_MOESM10_ESM.zip › G/G608G+LDN-large.tif]

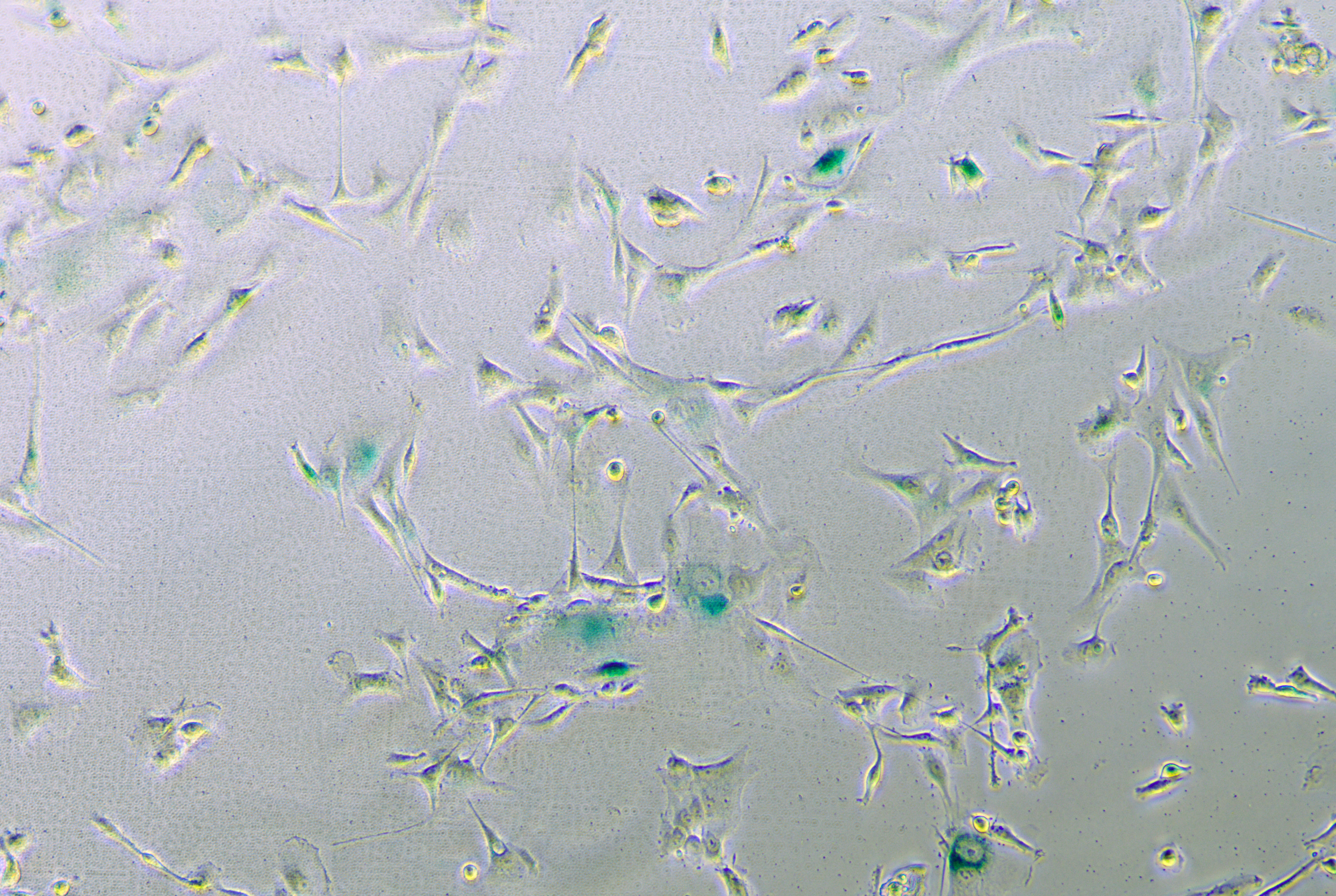

Supplement: Supplementary file 10 — Source data Fig. 5 [file 44318_2026_832_MOESM10_ESM.zip › G/G608G+LDN.jpg]

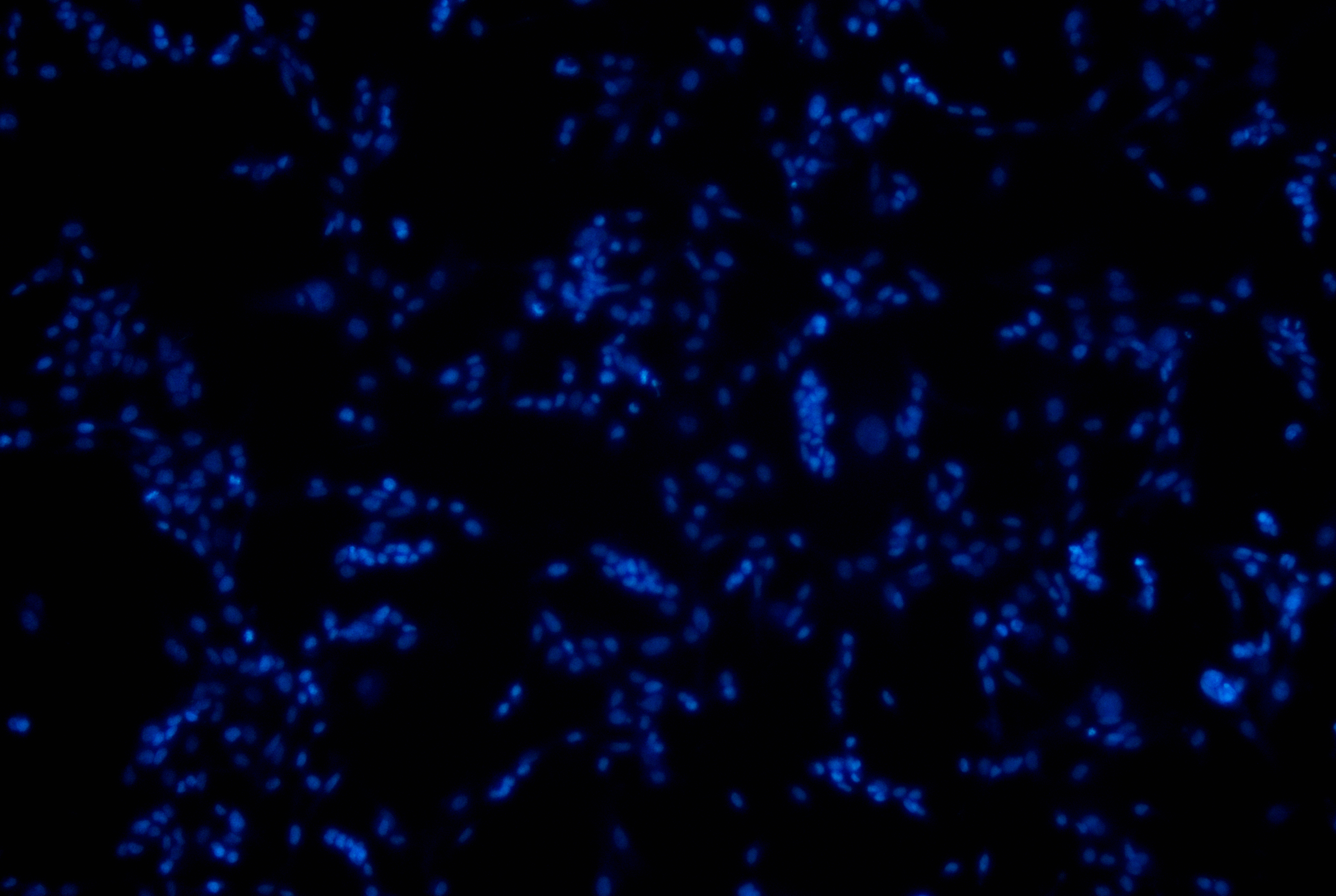

Supplement: Supplementary file 10 — Source data Fig. 5 [file 44318_2026_832_MOESM10_ESM.zip › G/G608G-DAPI.jpg]

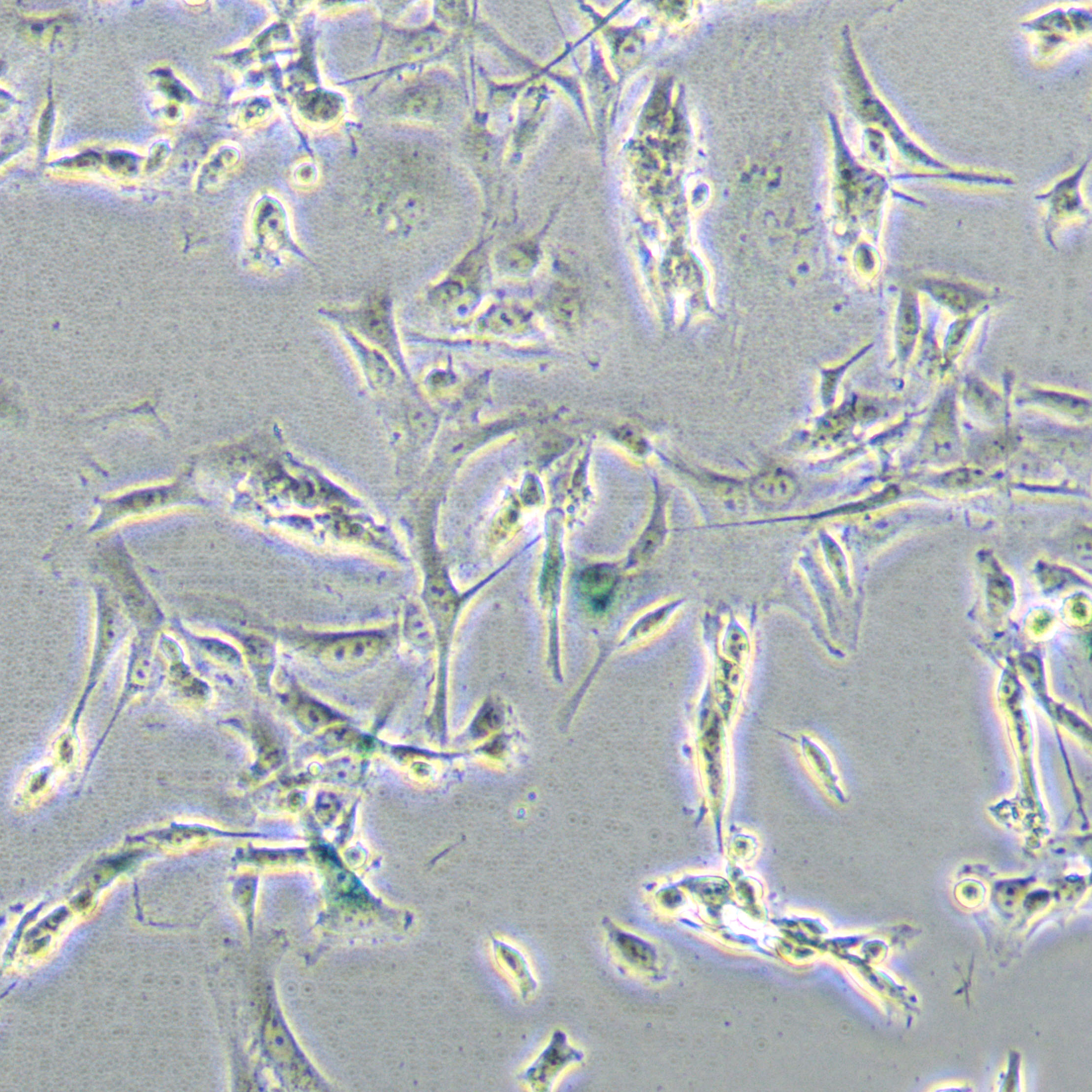

Supplement: Supplementary file 10 — Source data Fig. 5 [file 44318_2026_832_MOESM10_ESM.zip › G/G608G-large.tif]

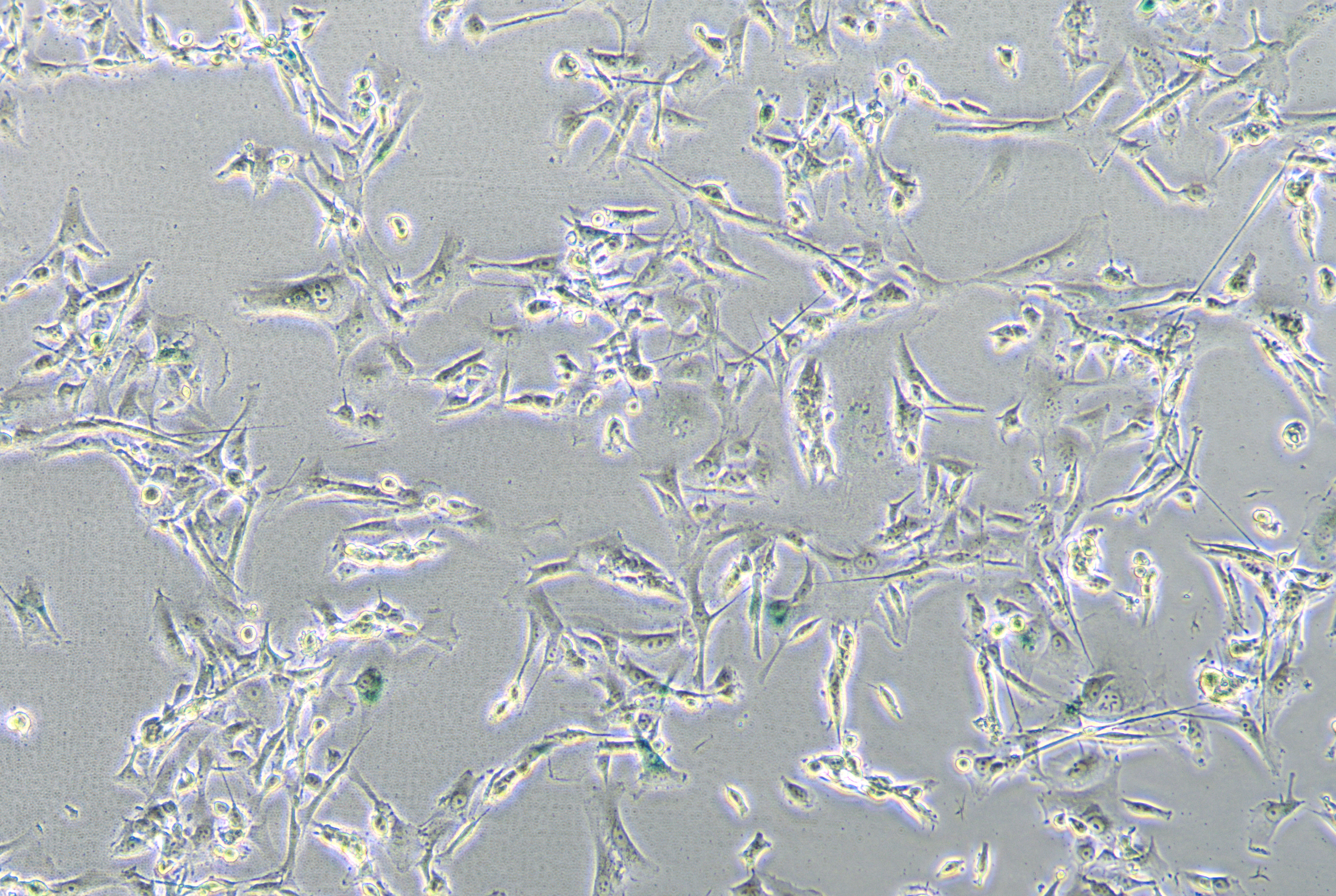

Supplement: Supplementary file 10 — Source data Fig. 5 [file 44318_2026_832_MOESM10_ESM.zip › G/G608G.jpg]

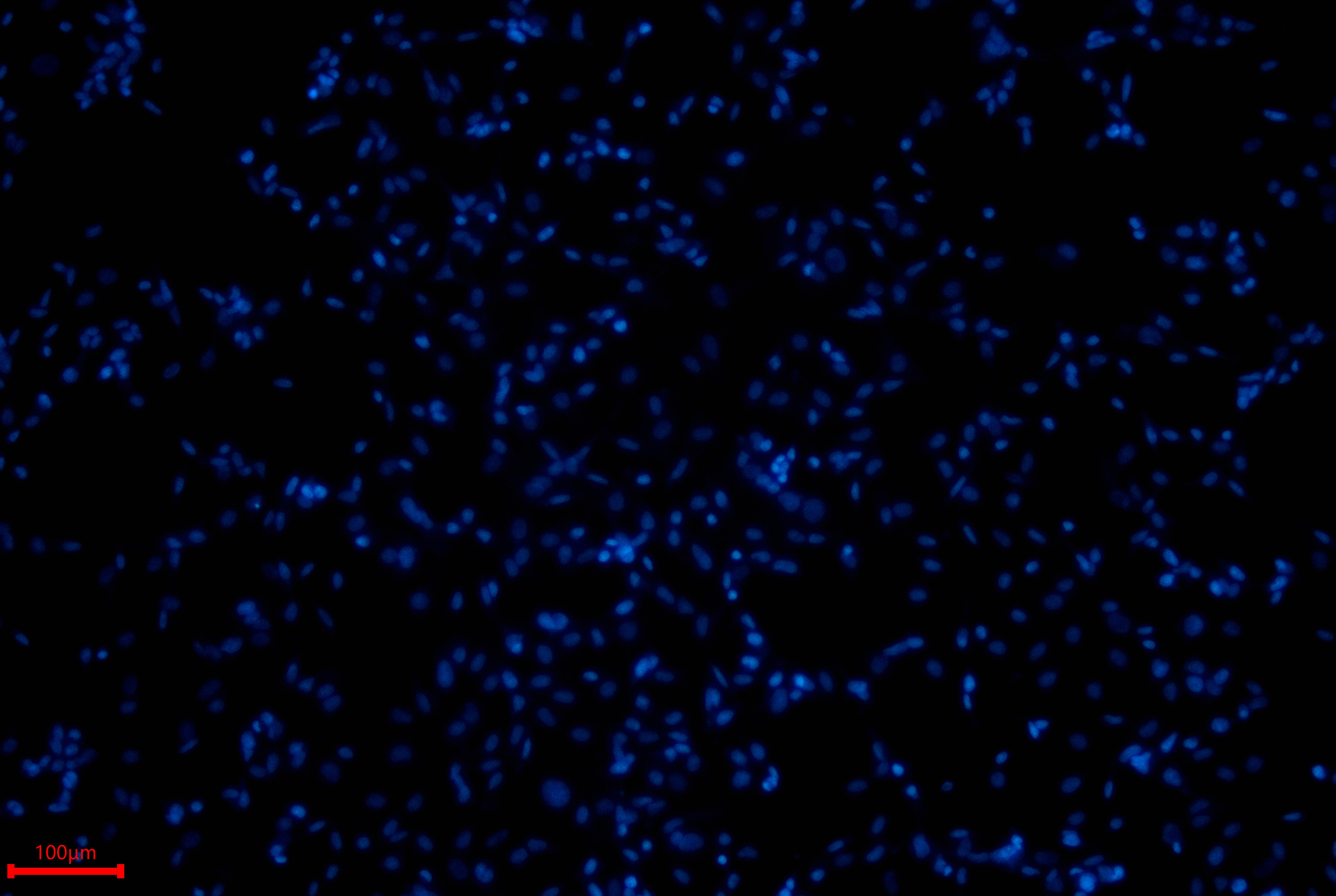

Supplement: Supplementary file 10 — Source data Fig. 5 [file 44318_2026_832_MOESM10_ESM.zip › G/WT+5-2 DAPI.jpg]

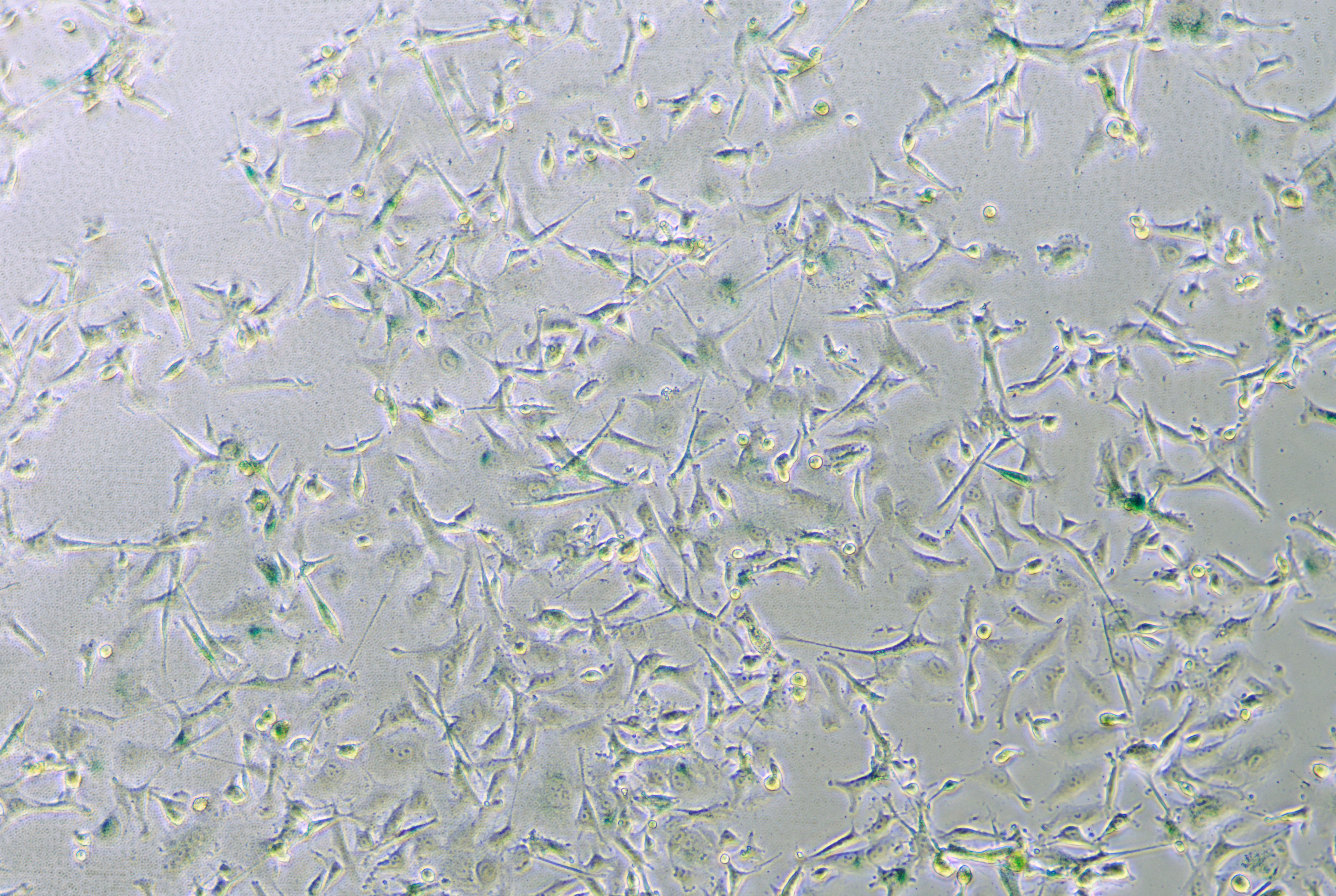

Supplement: Supplementary file 10 — Source data Fig. 5 [file 44318_2026_832_MOESM10_ESM.zip › G/WT+5-2.jpg]

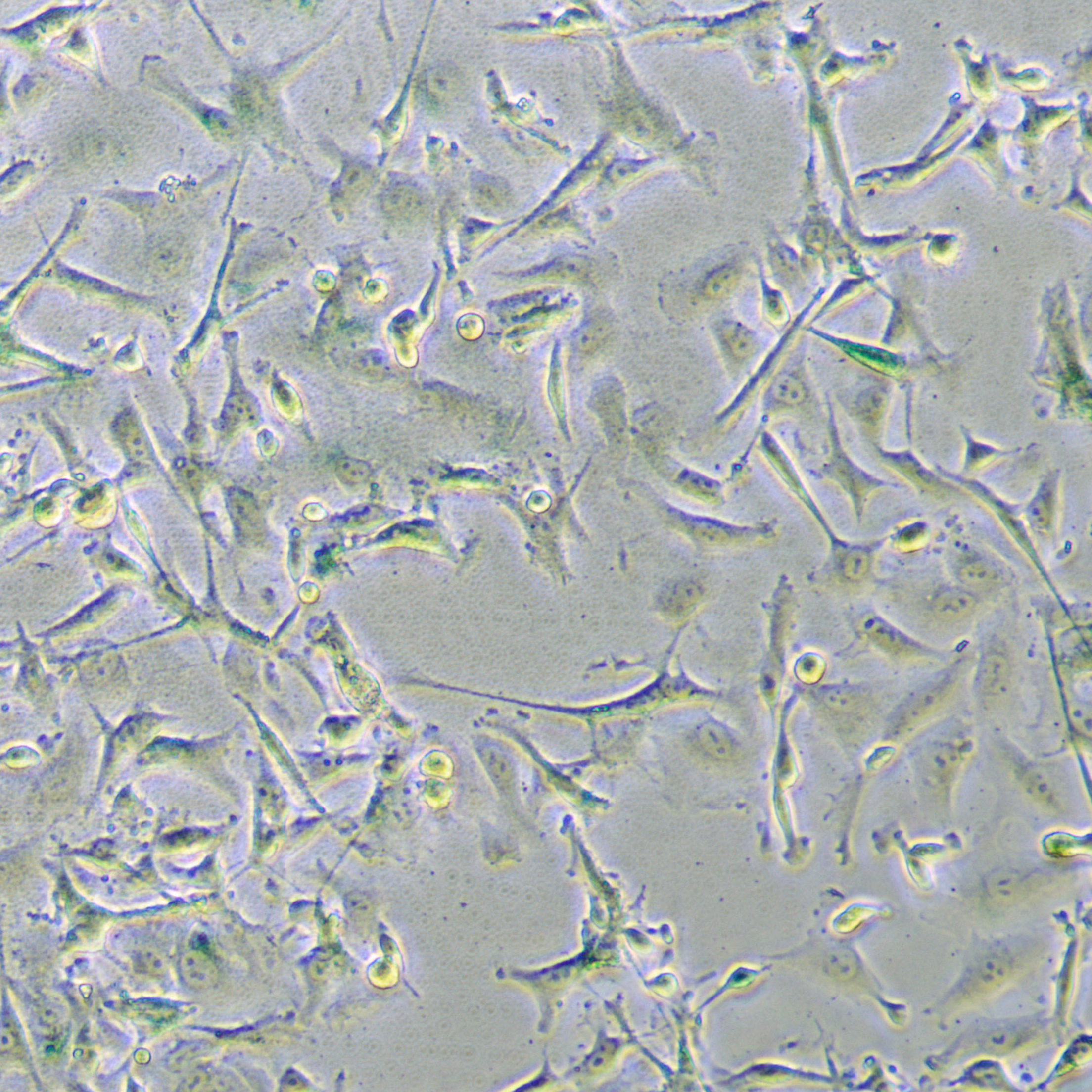

Supplement: Supplementary file 10 — Source data Fig. 5 [file 44318_2026_832_MOESM10_ESM.zip › G/WT+5ht-large.tif]

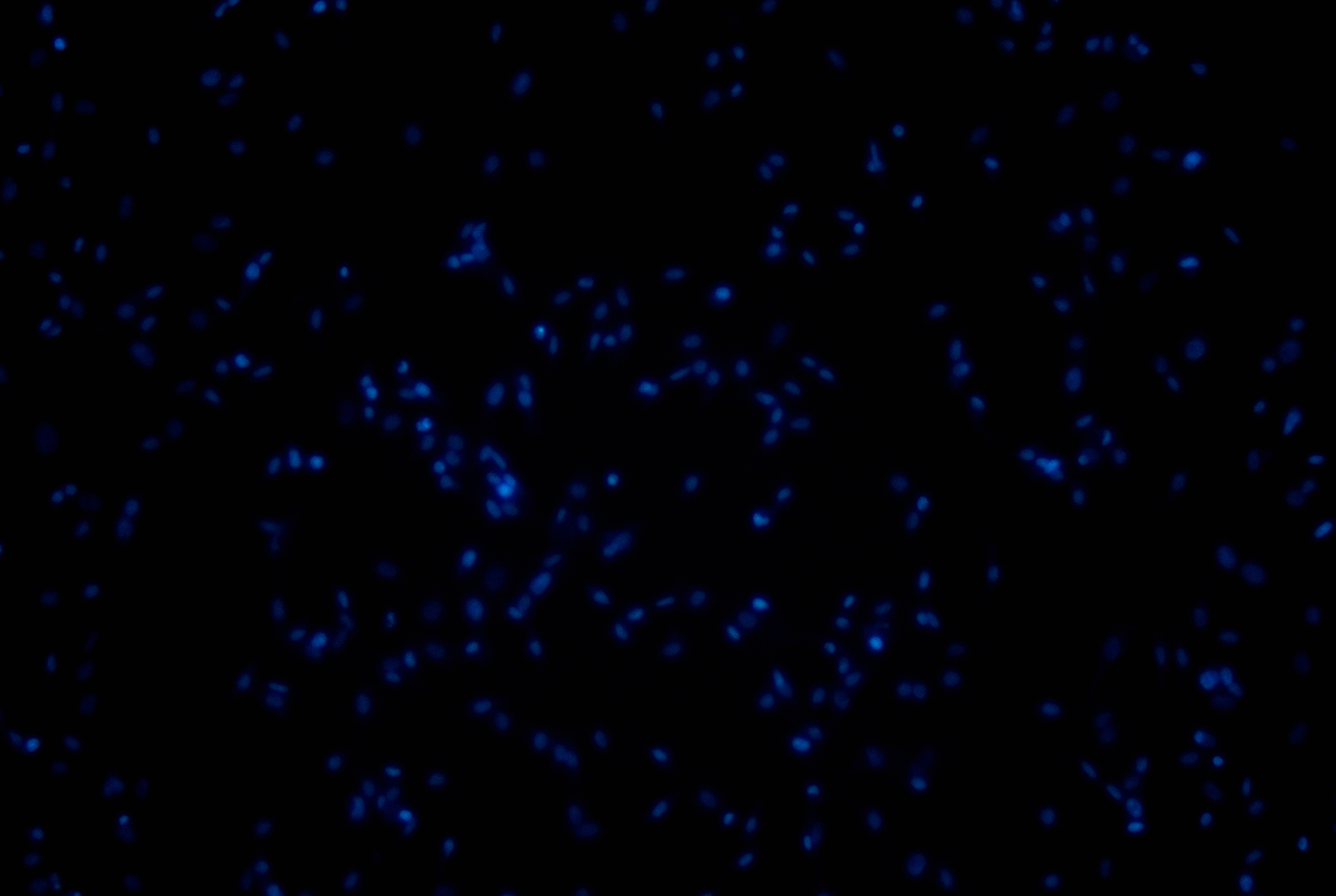

Supplement: Supplementary file 10 — Source data Fig. 5 [file 44318_2026_832_MOESM10_ESM.zip › G/WT+LDN+5ht-DAPI.jpg]

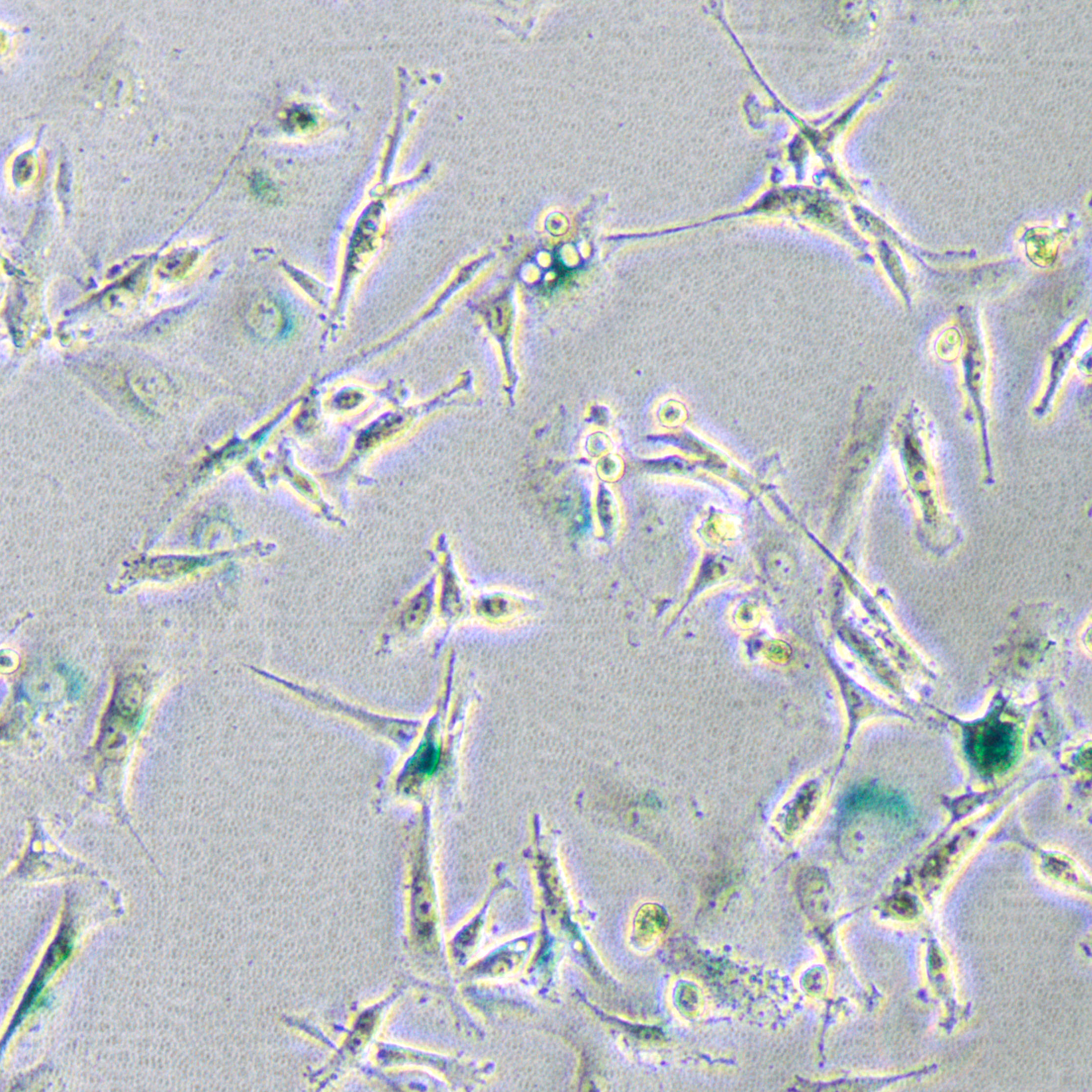

Supplement: Supplementary file 10 — Source data Fig. 5 [file 44318_2026_832_MOESM10_ESM.zip › G/WT+LDN+5ht-large.tif]

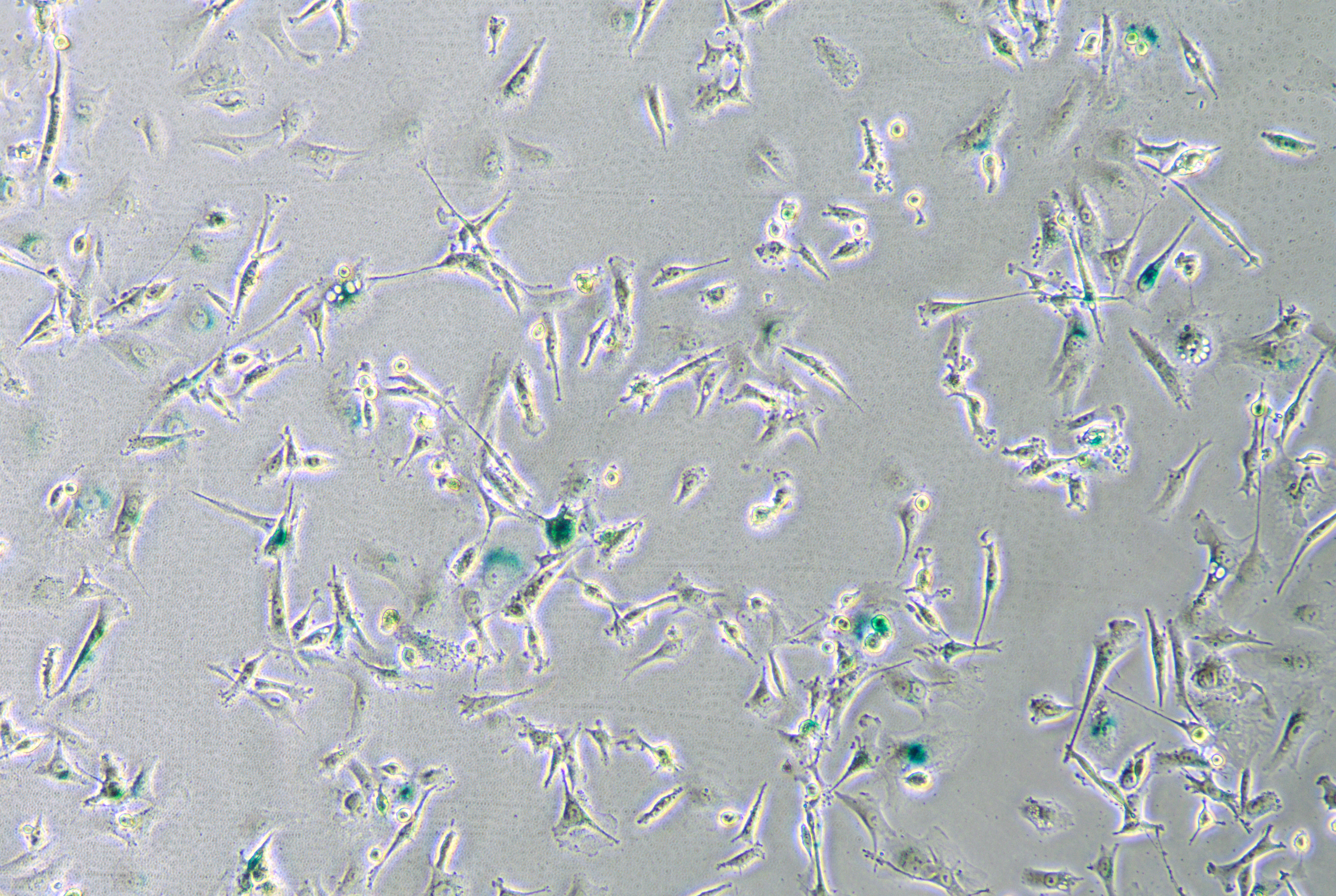

Supplement: Supplementary file 10 — Source data Fig. 5 [file 44318_2026_832_MOESM10_ESM.zip › G/WT+LDN+5ht.jpg]

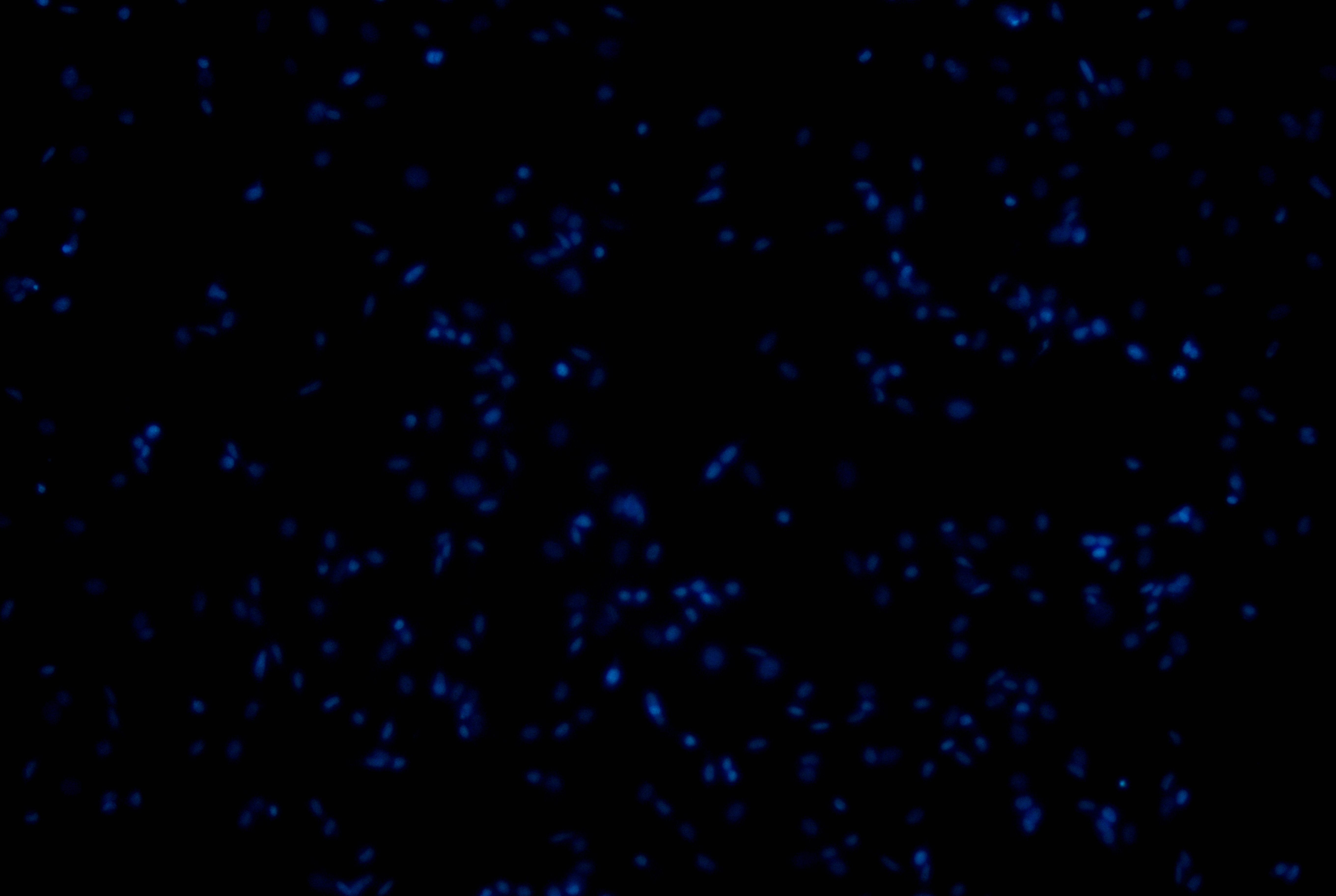

Supplement: Supplementary file 10 — Source data Fig. 5 [file 44318_2026_832_MOESM10_ESM.zip › G/WT+LDN-DAPI.jpg]

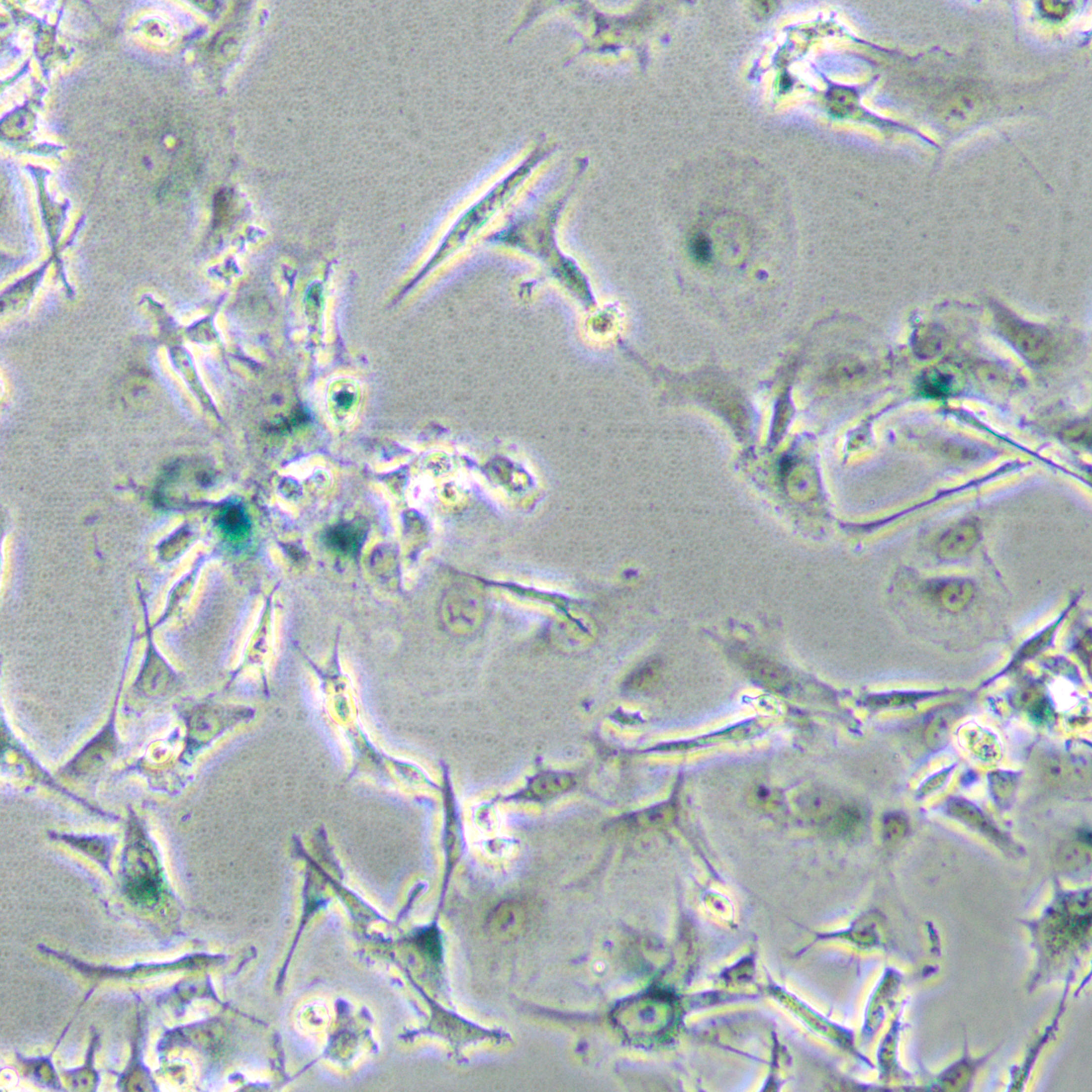

Supplement: Supplementary file 10 — Source data Fig. 5 [file 44318_2026_832_MOESM10_ESM.zip › G/WT+LDN-large.tif]

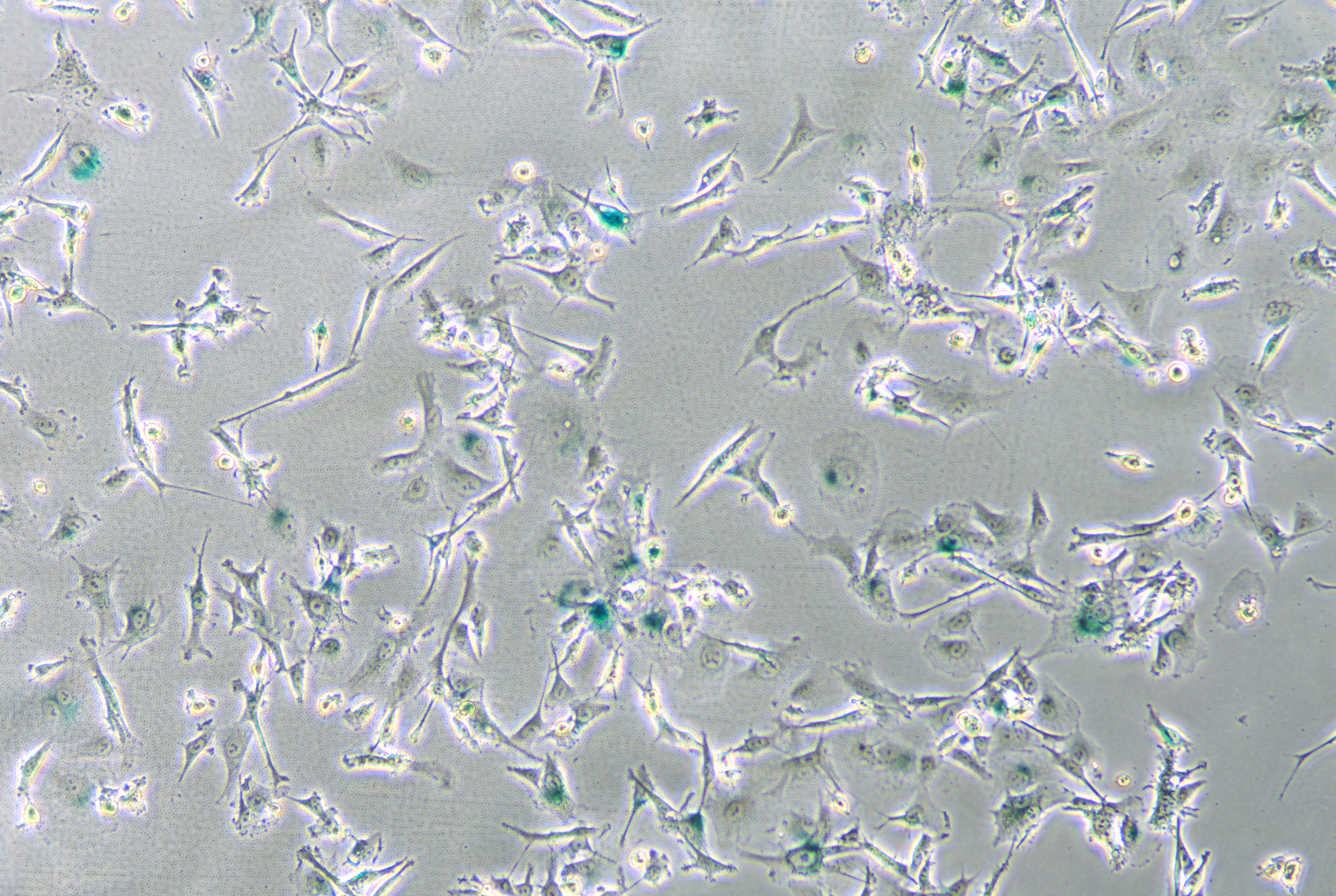

Supplement: Supplementary file 10 — Source data Fig. 5 [file 44318_2026_832_MOESM10_ESM.zip › G/WT+LDN.jpg]

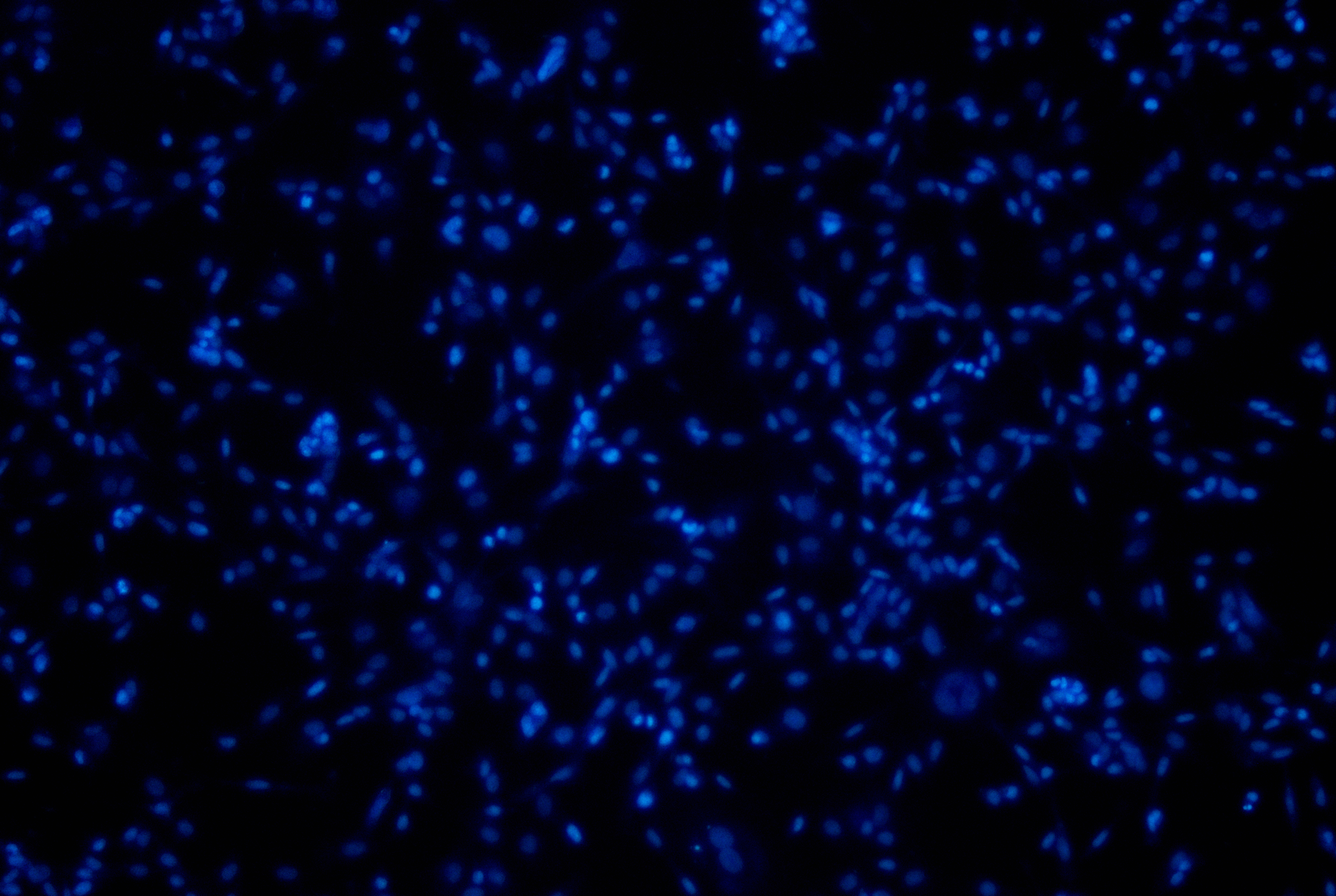

Supplement: Supplementary file 10 — Source data Fig. 5 [file 44318_2026_832_MOESM10_ESM.zip › G/WT-DAPI.jpg]

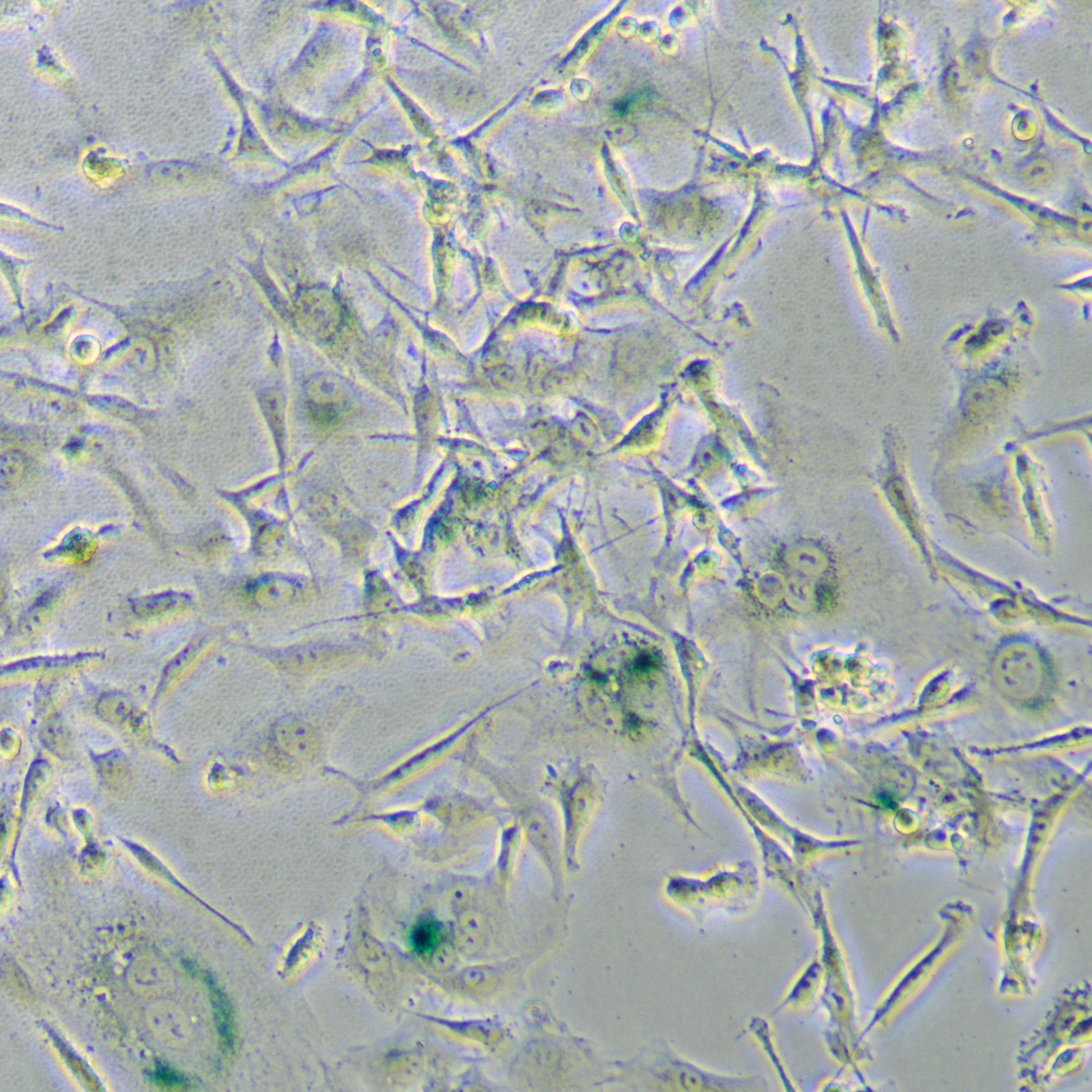

Supplement: Supplementary file 10 — Source data Fig. 5 [file 44318_2026_832_MOESM10_ESM.zip › G/WT-large.tif]

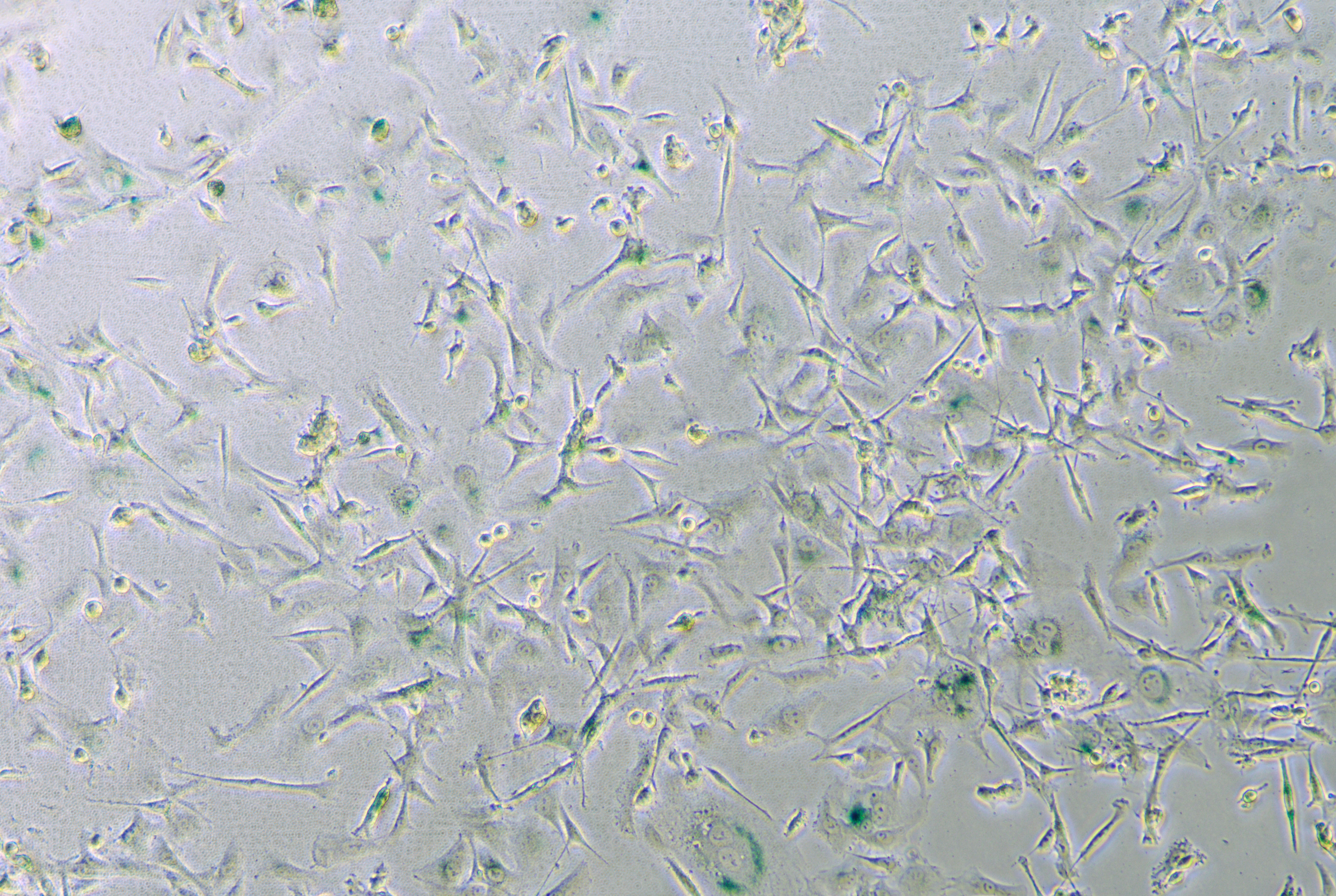

Supplement: Supplementary file 10 — Source data Fig. 5 [file 44318_2026_832_MOESM10_ESM.zip › G/WT.jpg]

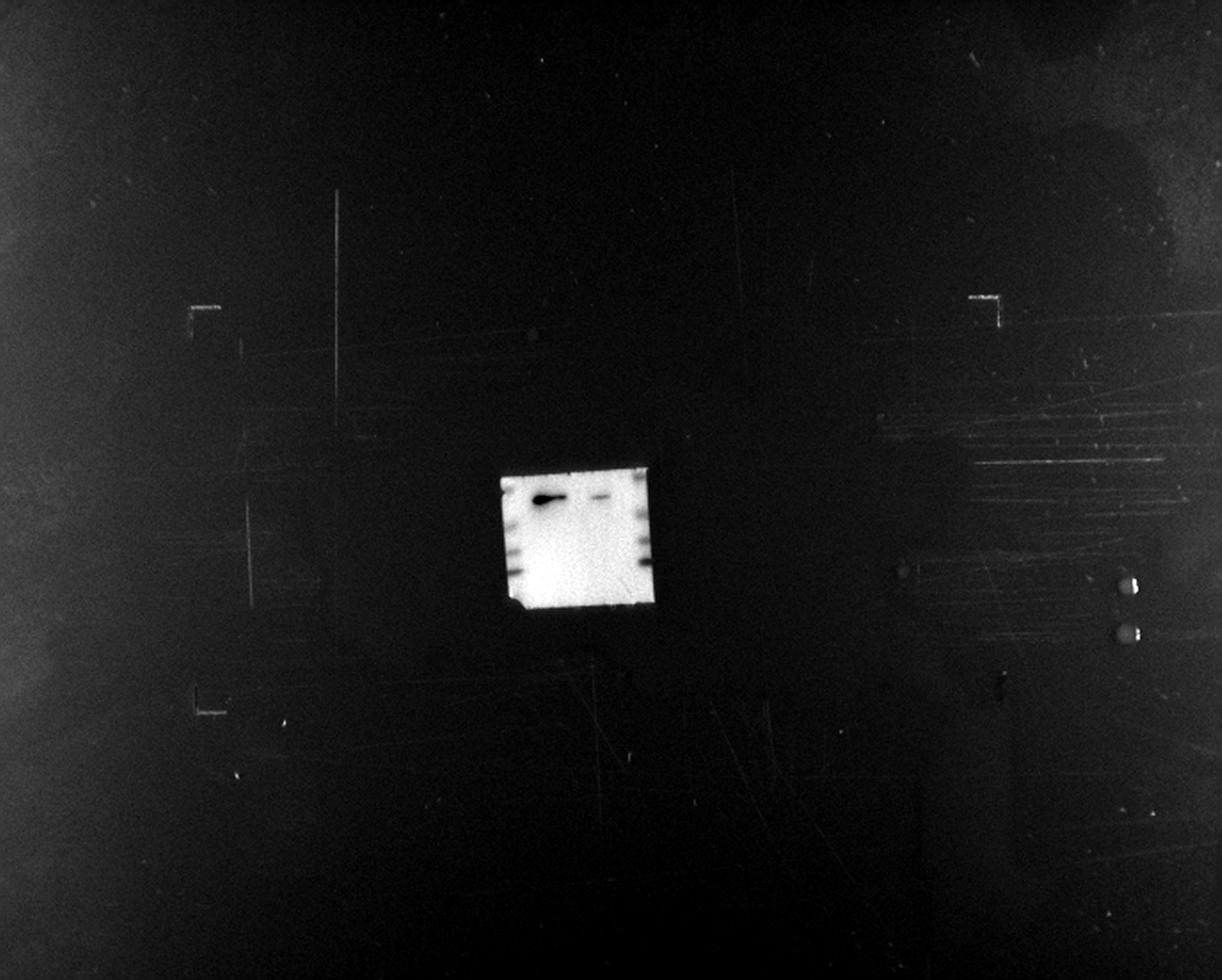

Supplement: Supplementary file 11 — Source data Fig. 6 [file 44318_2026_832_MOESM11_ESM.zip › B/G608G CHOP.tif]

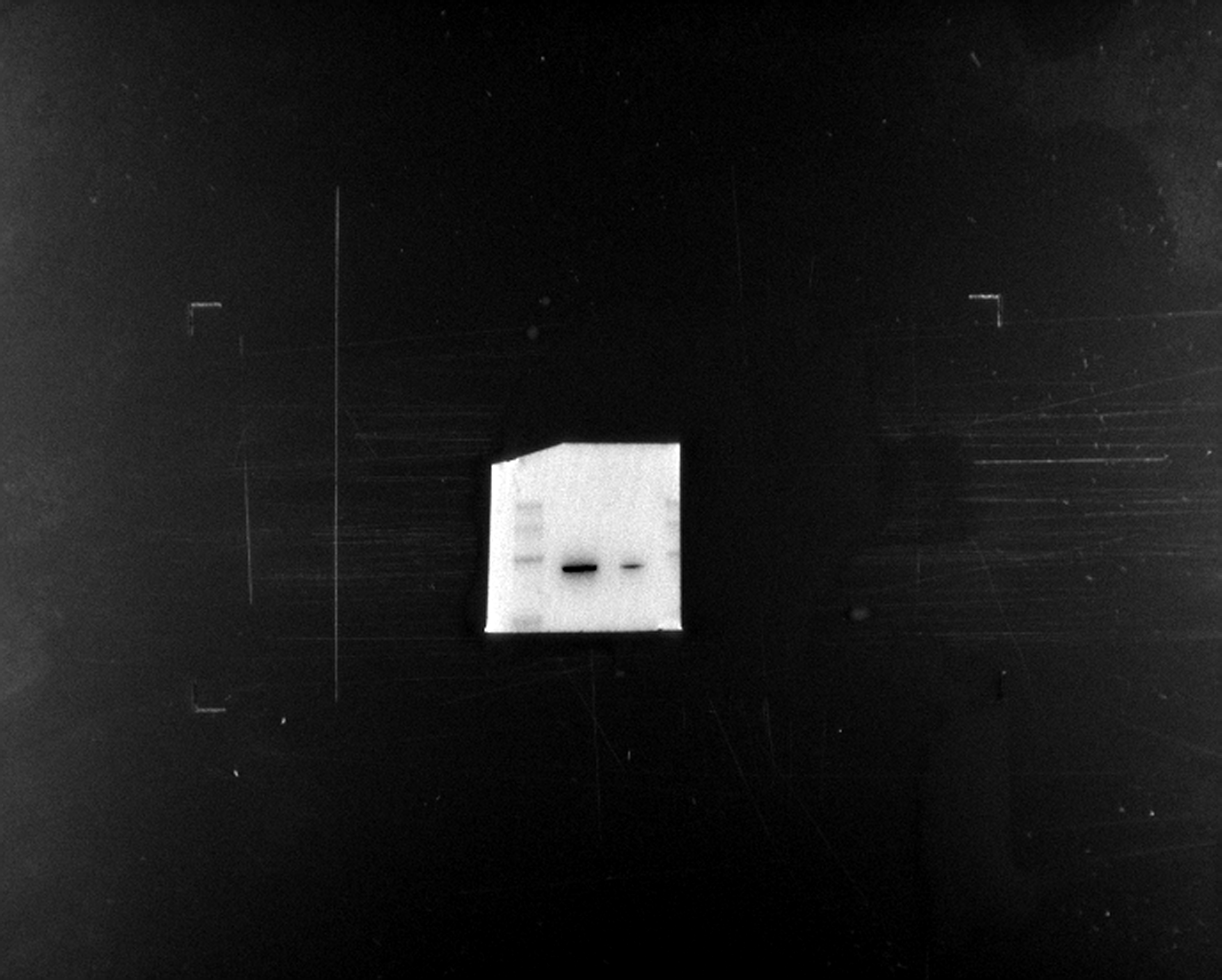

Supplement: Supplementary file 11 — Source data Fig. 6 [file 44318_2026_832_MOESM11_ESM.zip › B/G608G HSP90β.tif]

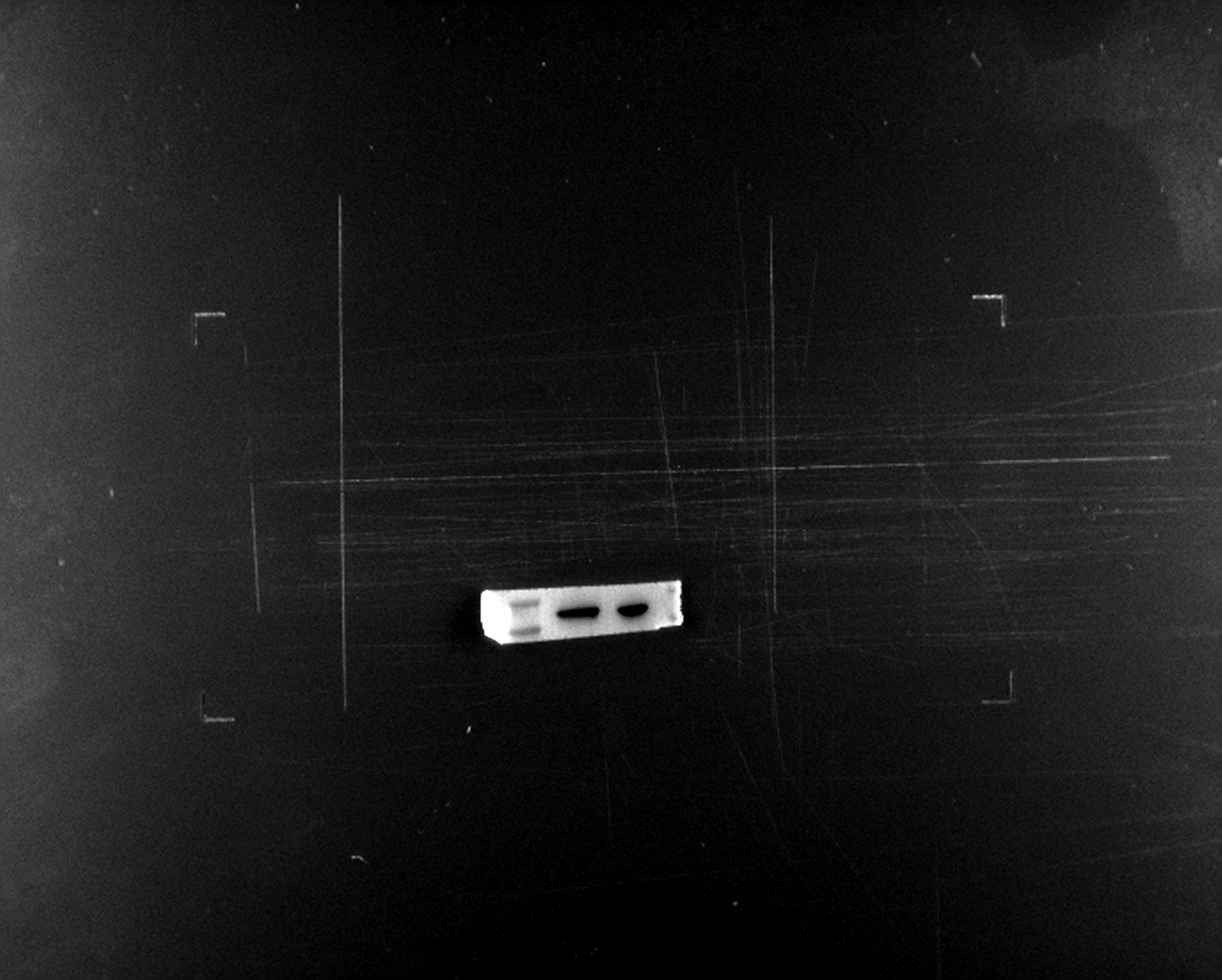

Supplement: Supplementary file 11 — Source data Fig. 6 [file 44318_2026_832_MOESM11_ESM.zip › B/G608G β-actin.tif]

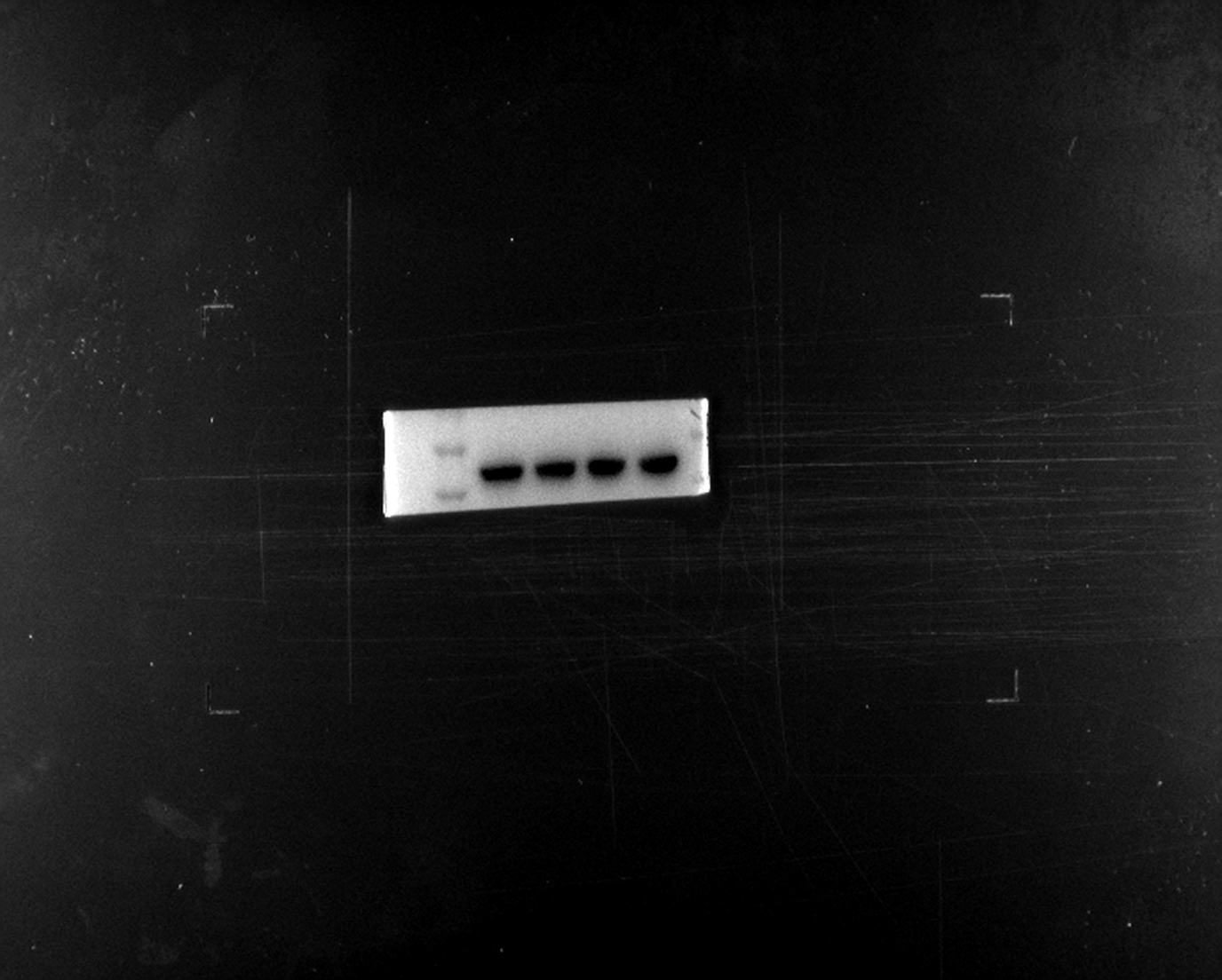

Supplement: Supplementary file 11 — Source data Fig. 6 [file 44318_2026_832_MOESM11_ESM.zip › B/replicates/A-ACTIN-WT WT WTKD WTKD -3.tif]

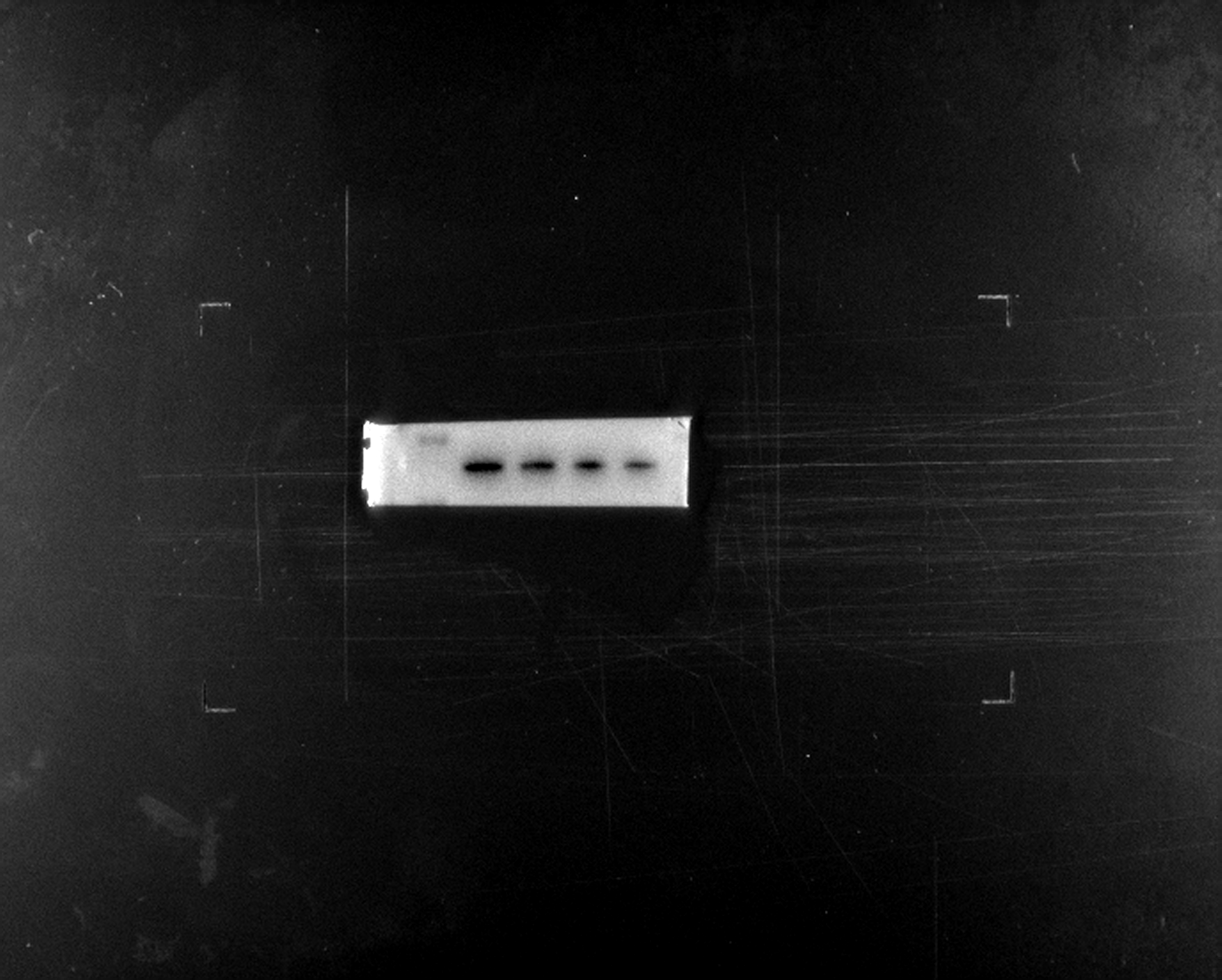

Supplement: Supplementary file 11 — Source data Fig. 6 [file 44318_2026_832_MOESM11_ESM.zip › B/replicates/A-DDIT3-WT WT WTKD WTKD -3.tif]

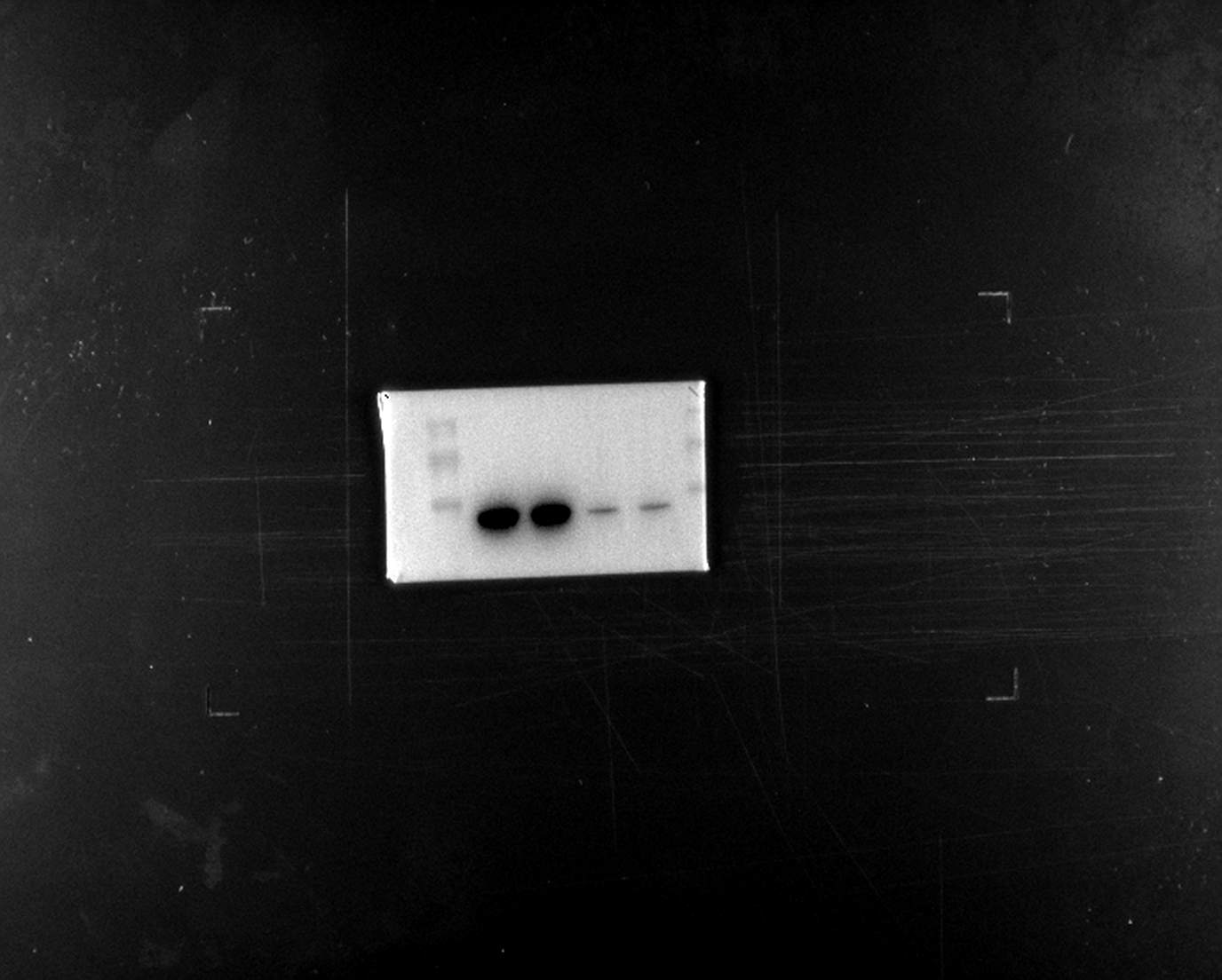

Supplement: Supplementary file 11 — Source data Fig. 6 [file 44318_2026_832_MOESM11_ESM.zip › B/replicates/A-HSP90-WT WT WTKD WTKD -3.tif]

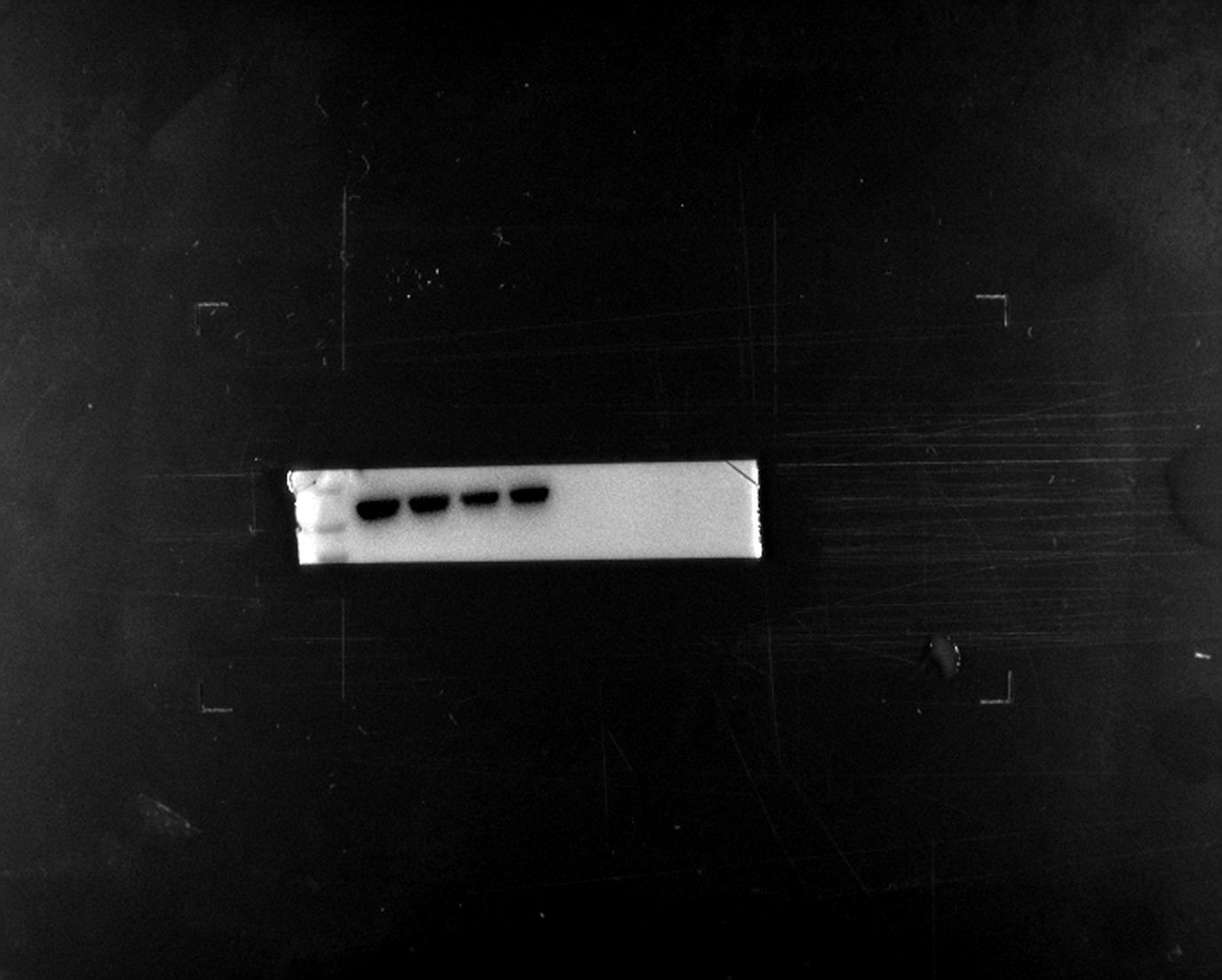

Supplement: Supplementary file 11 — Source data Fig. 6 [file 44318_2026_832_MOESM11_ESM.zip › B/replicates/B-ACTIN-G608G G608G G608GKD G608GKD-3.tif]

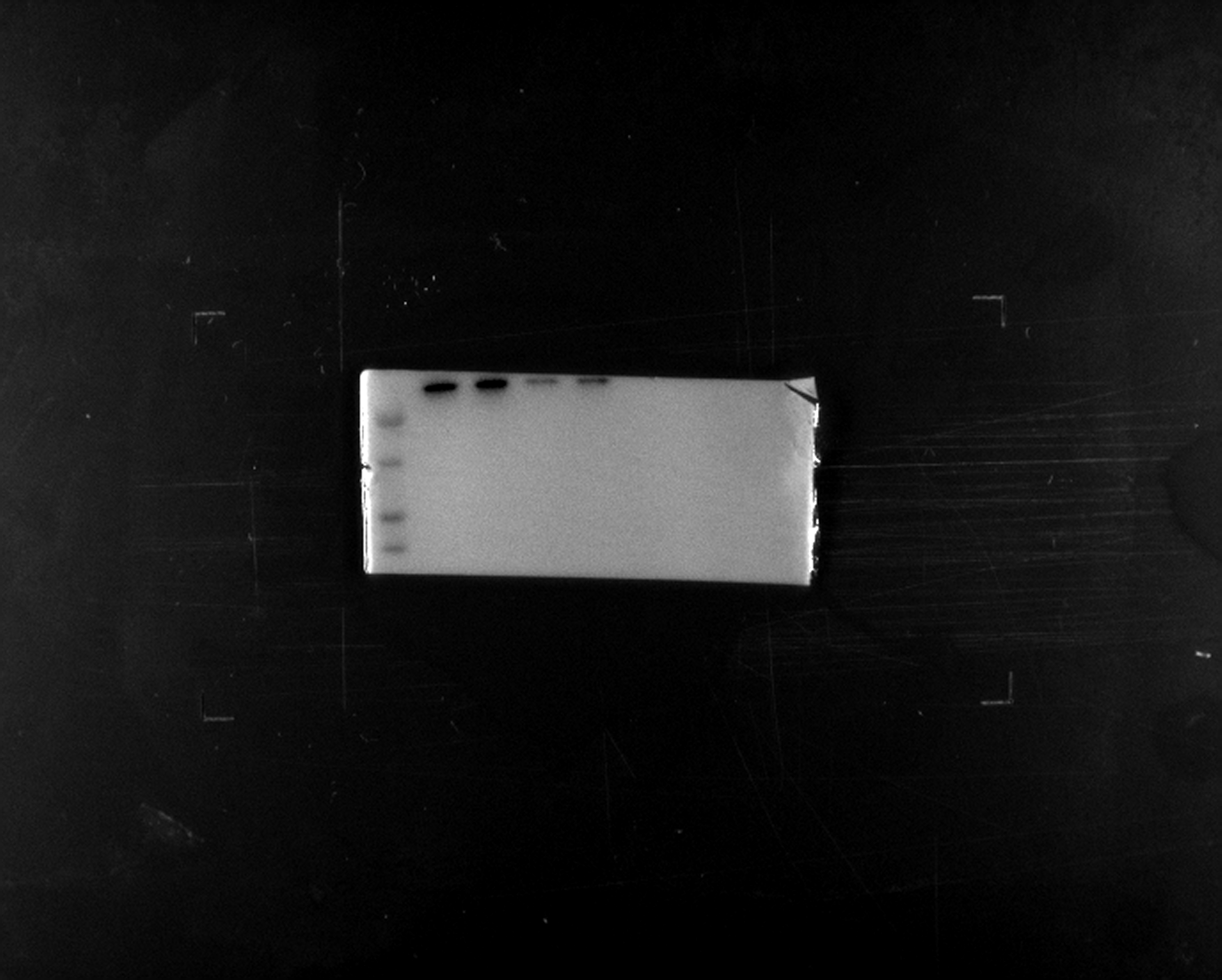

Supplement: Supplementary file 11 — Source data Fig. 6 [file 44318_2026_832_MOESM11_ESM.zip › B/replicates/B-DDIT3-G608G G608G G608GKD G608GKD-3.tif]

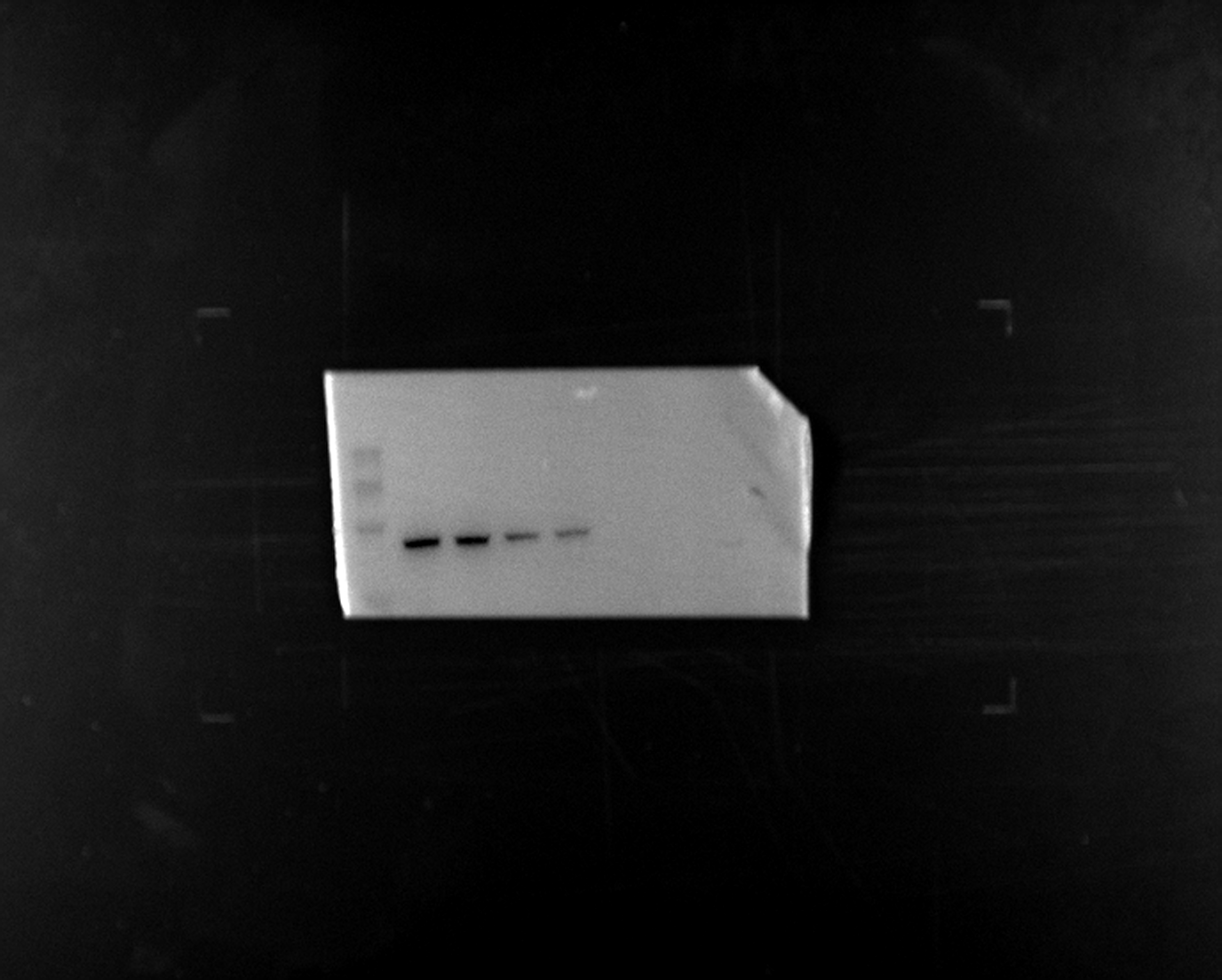

Supplement: Supplementary file 11 — Source data Fig. 6 [file 44318_2026_832_MOESM11_ESM.zip › B/replicates/B-HSP90-G608G G608G G608GKD G608GKD-3.tif]

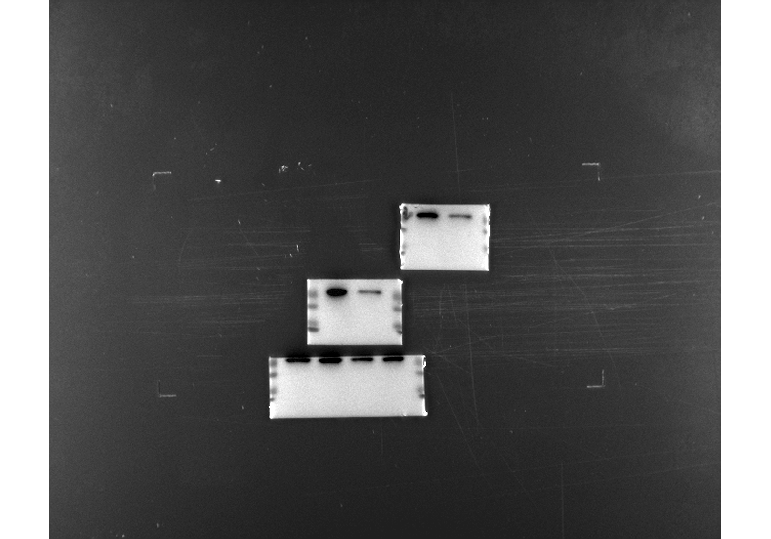

Supplement: Supplementary file 11 — Source data Fig. 6 [file 44318_2026_832_MOESM11_ESM.zip › B/WT CHOP.tif]

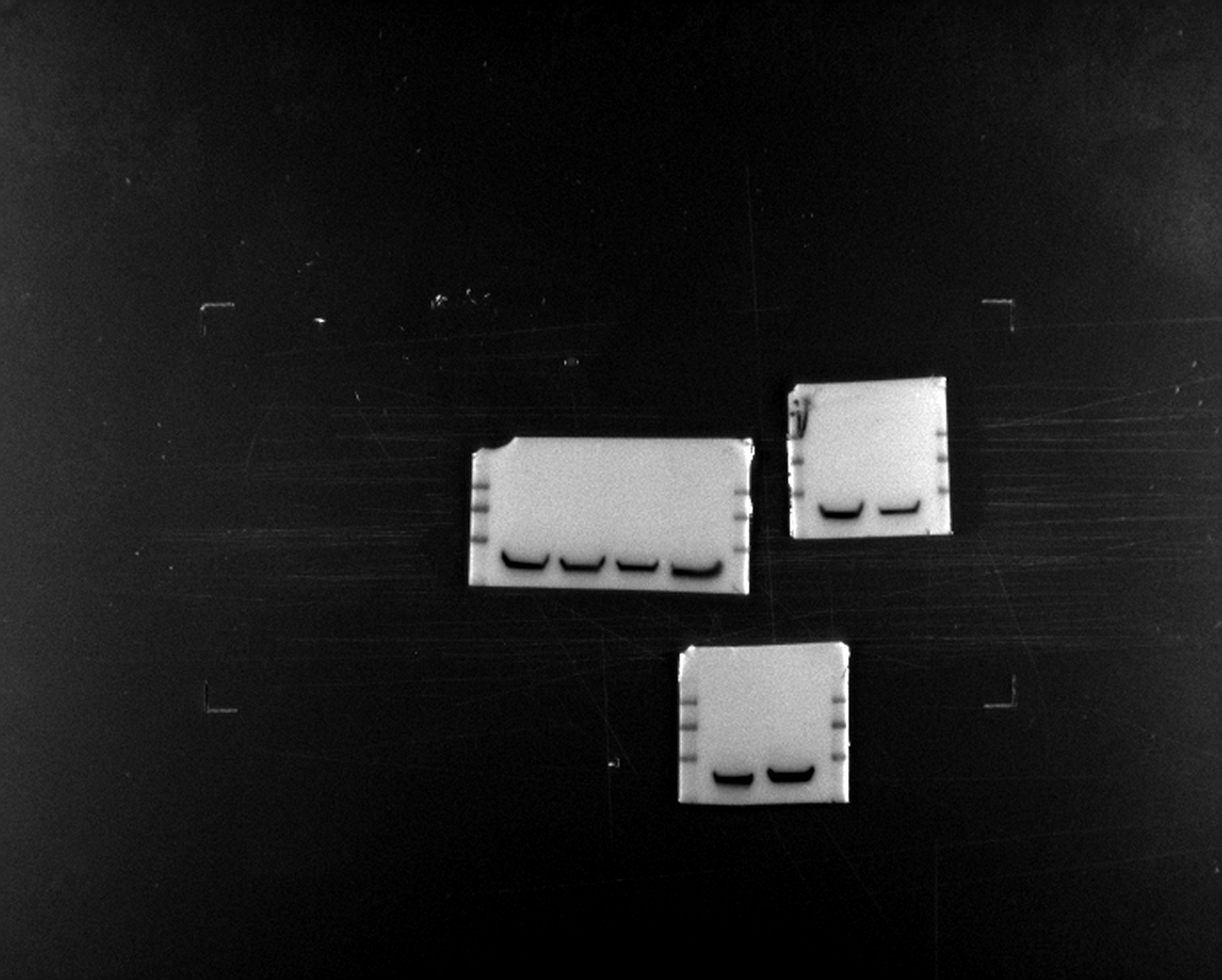

Supplement: Supplementary file 11 — Source data Fig. 6 [file 44318_2026_832_MOESM11_ESM.zip › B/WT HSP90β.tif]

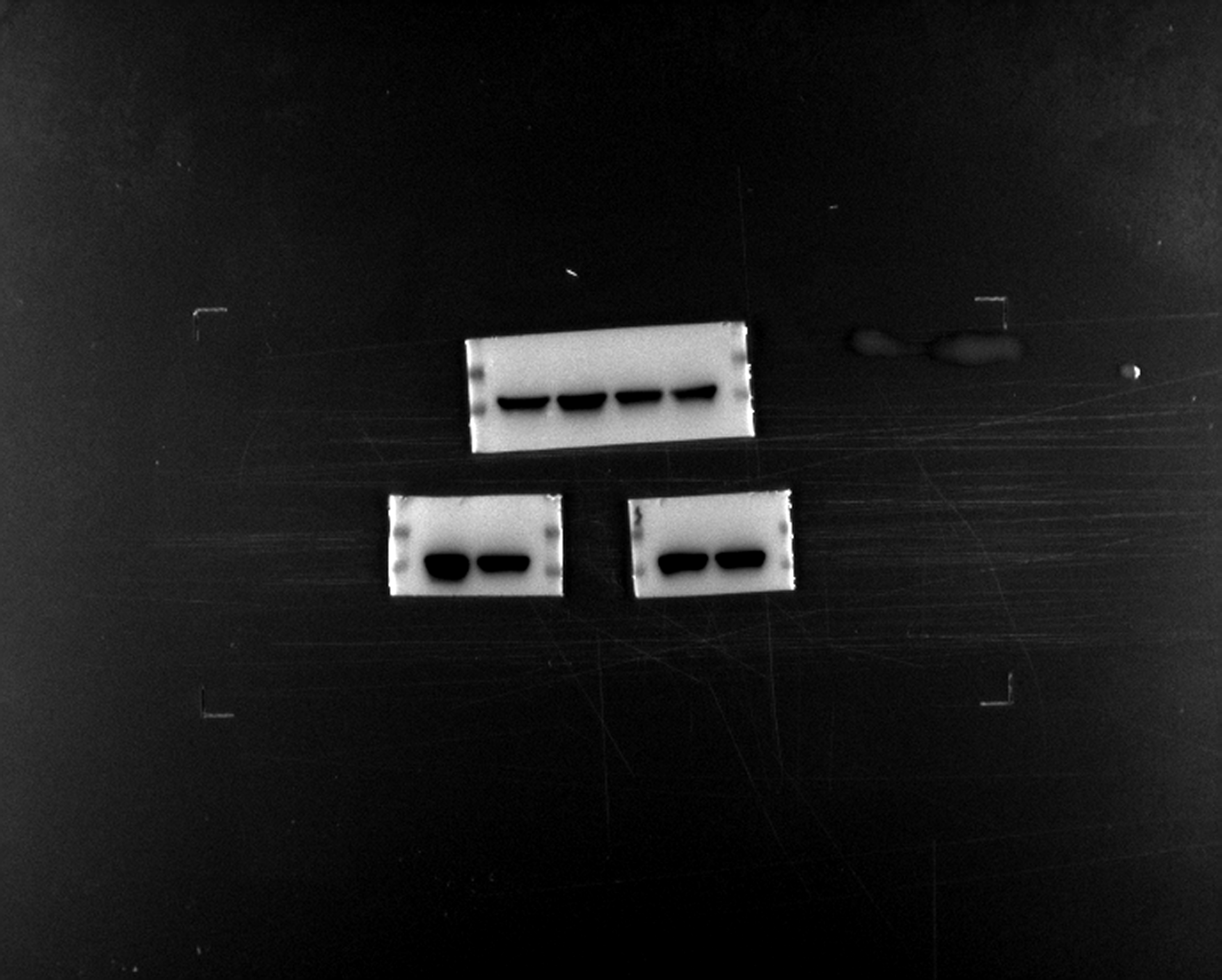

Supplement: Supplementary file 11 — Source data Fig. 6 [file 44318_2026_832_MOESM11_ESM.zip › B/WT β-actin.tif]

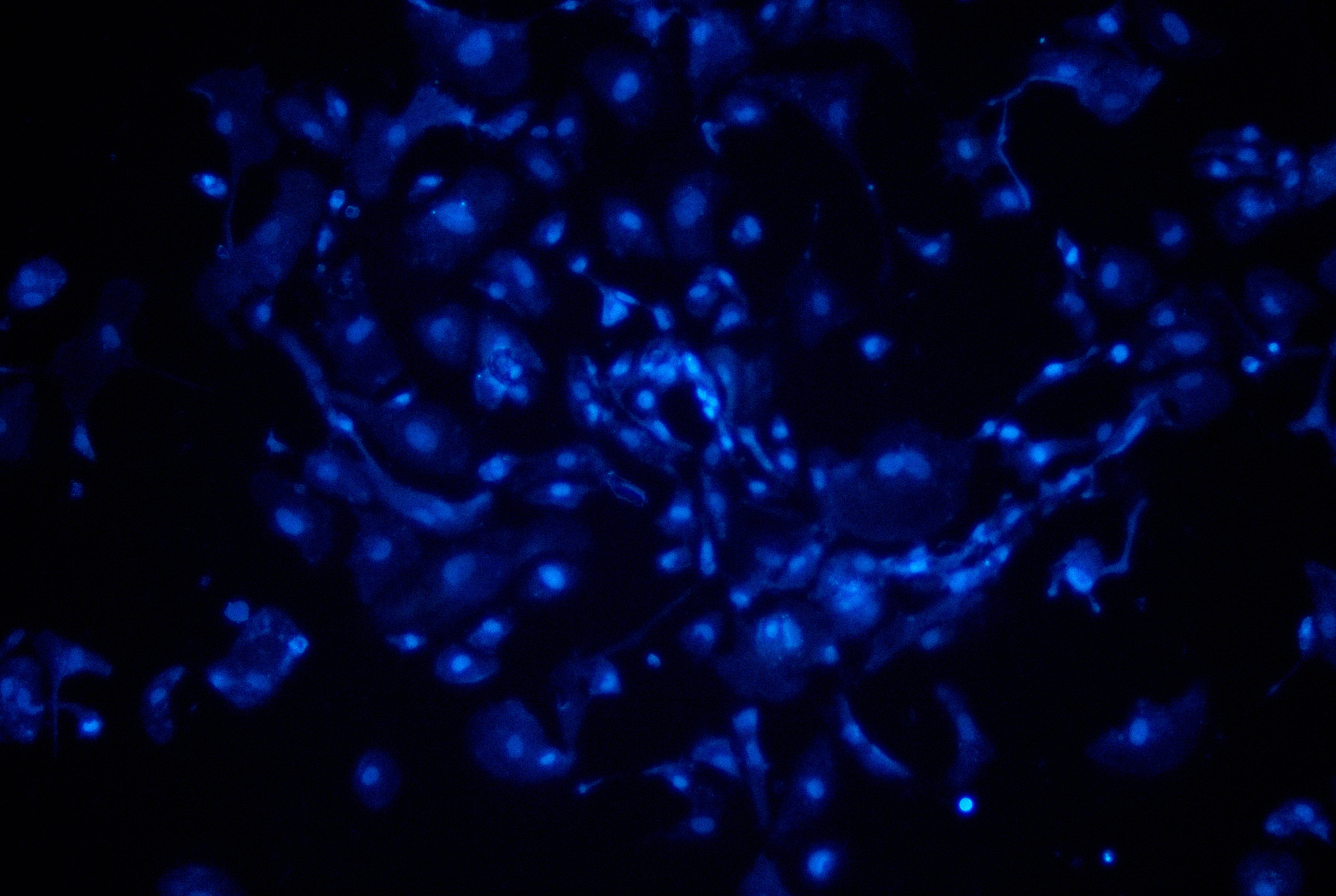

Supplement: Supplementary file 11 — Source data Fig. 6 [file 44318_2026_832_MOESM11_ESM.zip › C/G608G DAPI.jpg]

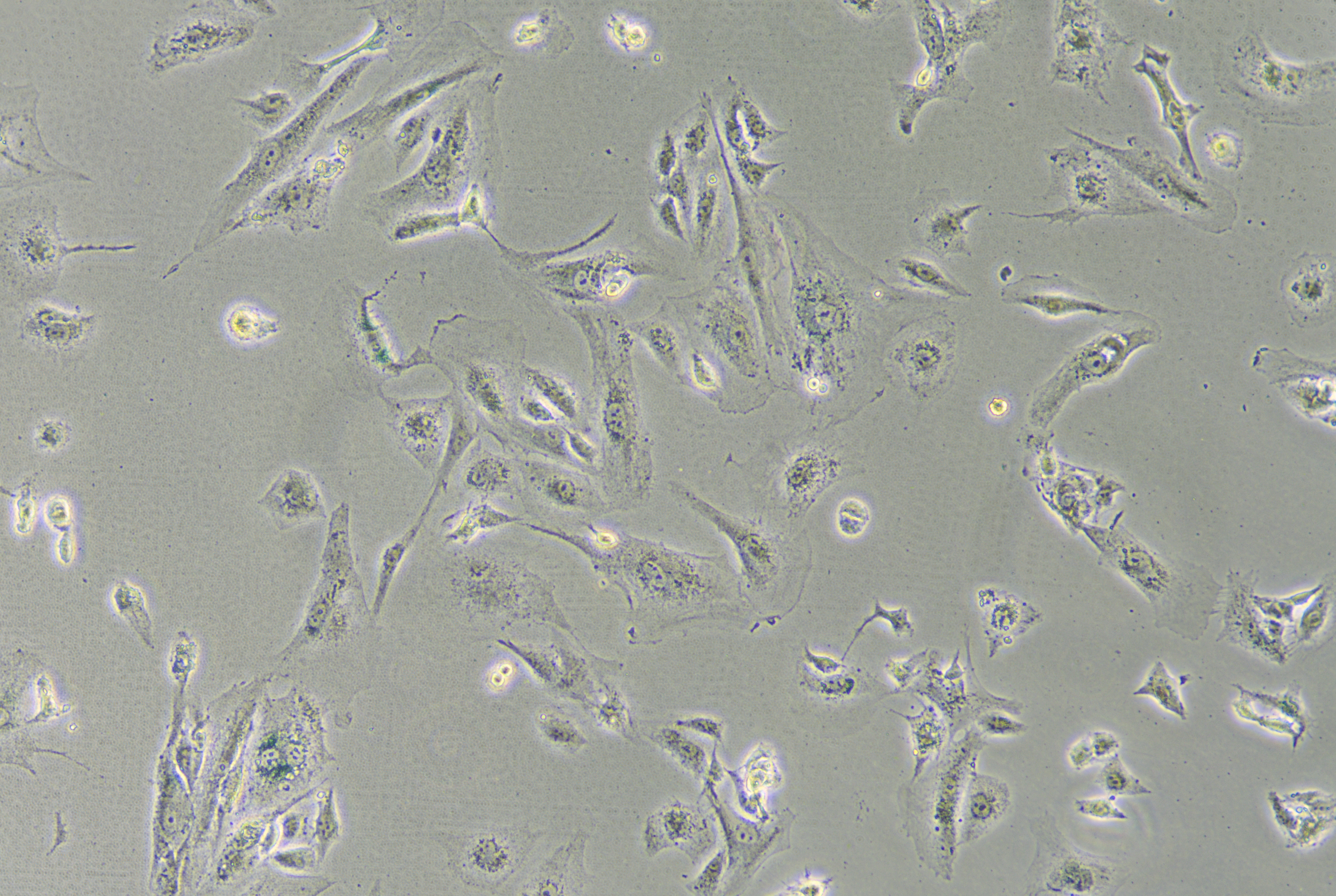

Supplement: Supplementary file 11 — Source data Fig. 6 [file 44318_2026_832_MOESM11_ESM.zip › C/G608G sh90.jpg]

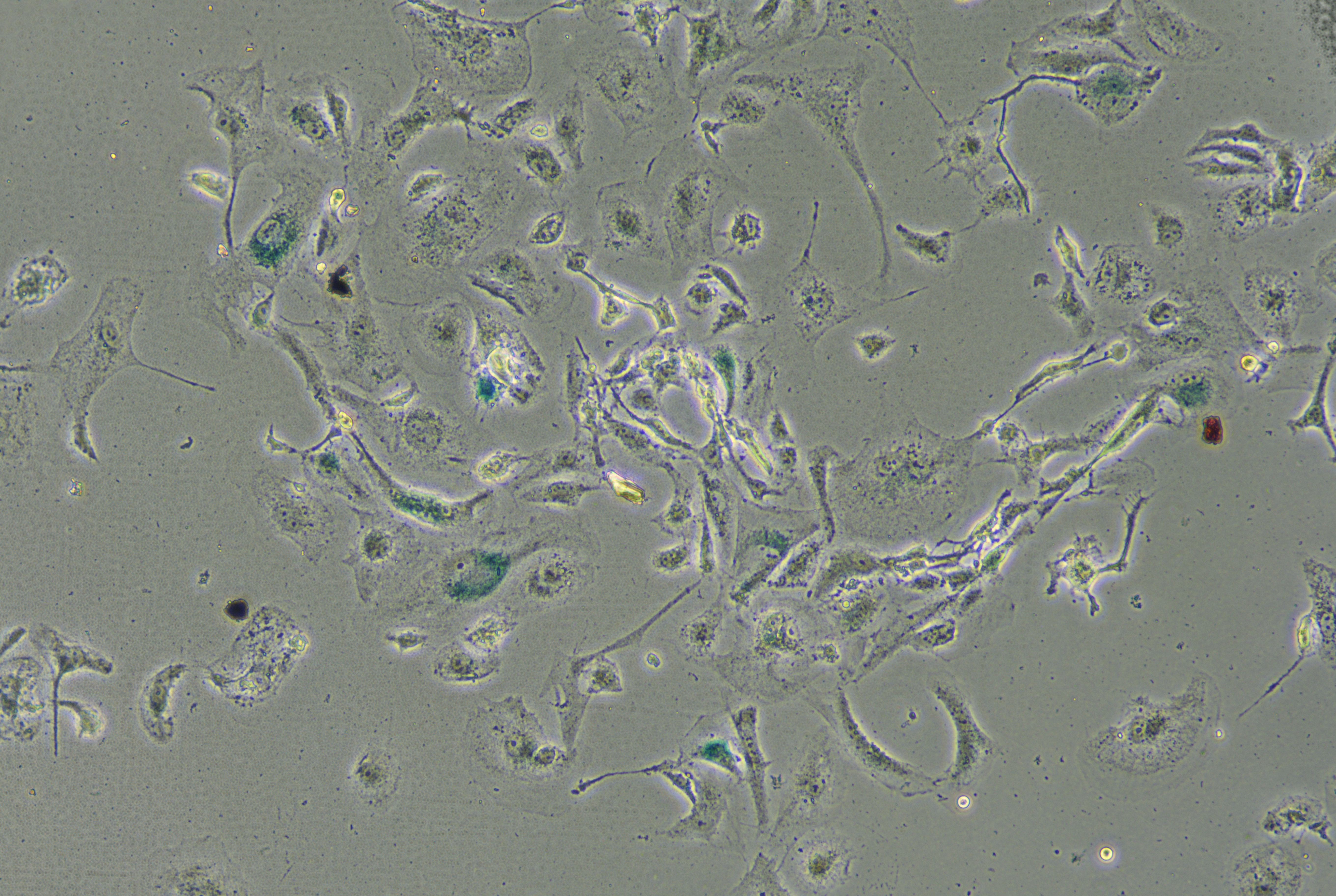

Supplement: Supplementary file 11 — Source data Fig. 6 [file 44318_2026_832_MOESM11_ESM.zip › C/G608G.jpg]

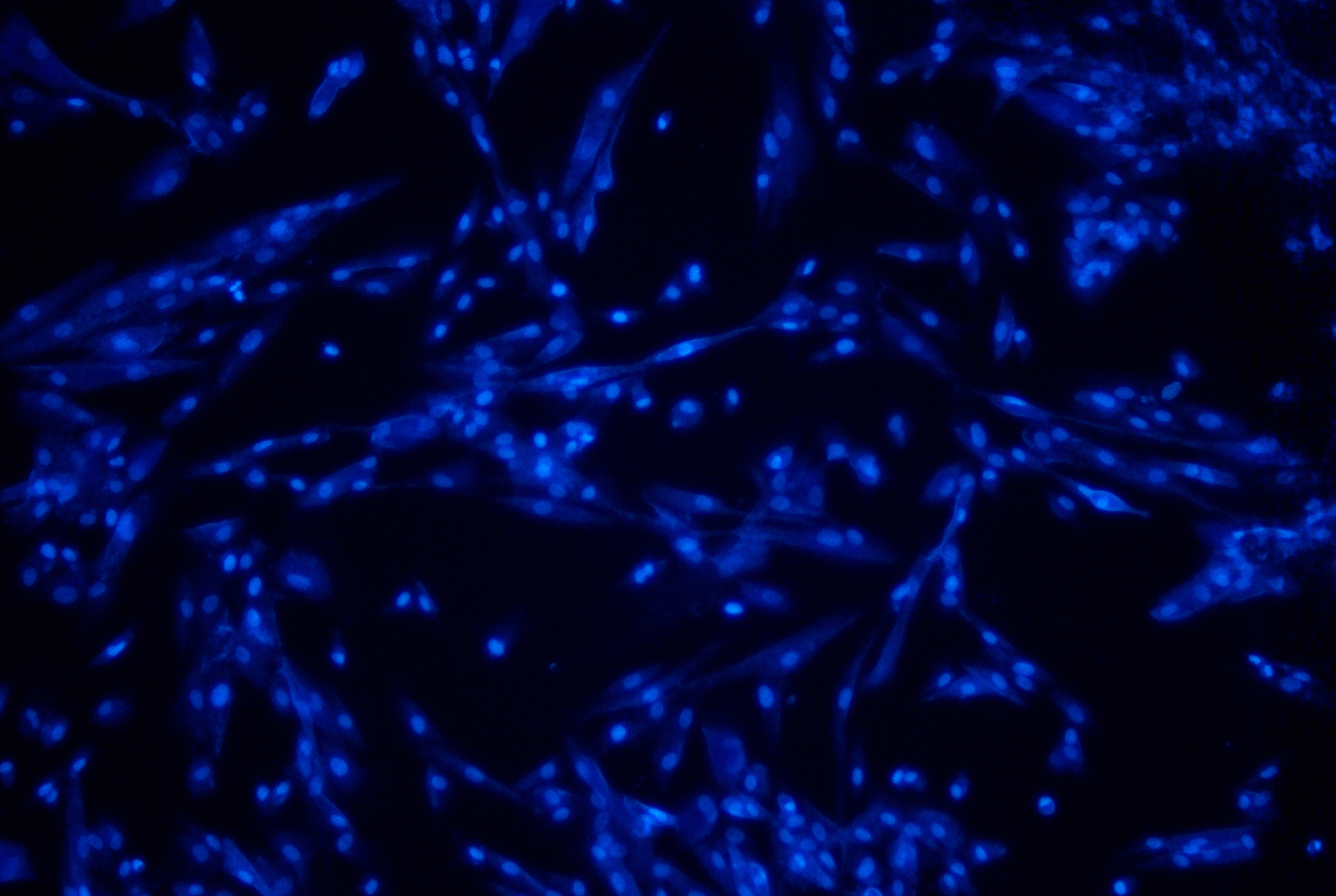

Supplement: Supplementary file 11 — Source data Fig. 6 [file 44318_2026_832_MOESM11_ESM.zip › C/WT DAPI.jpg]

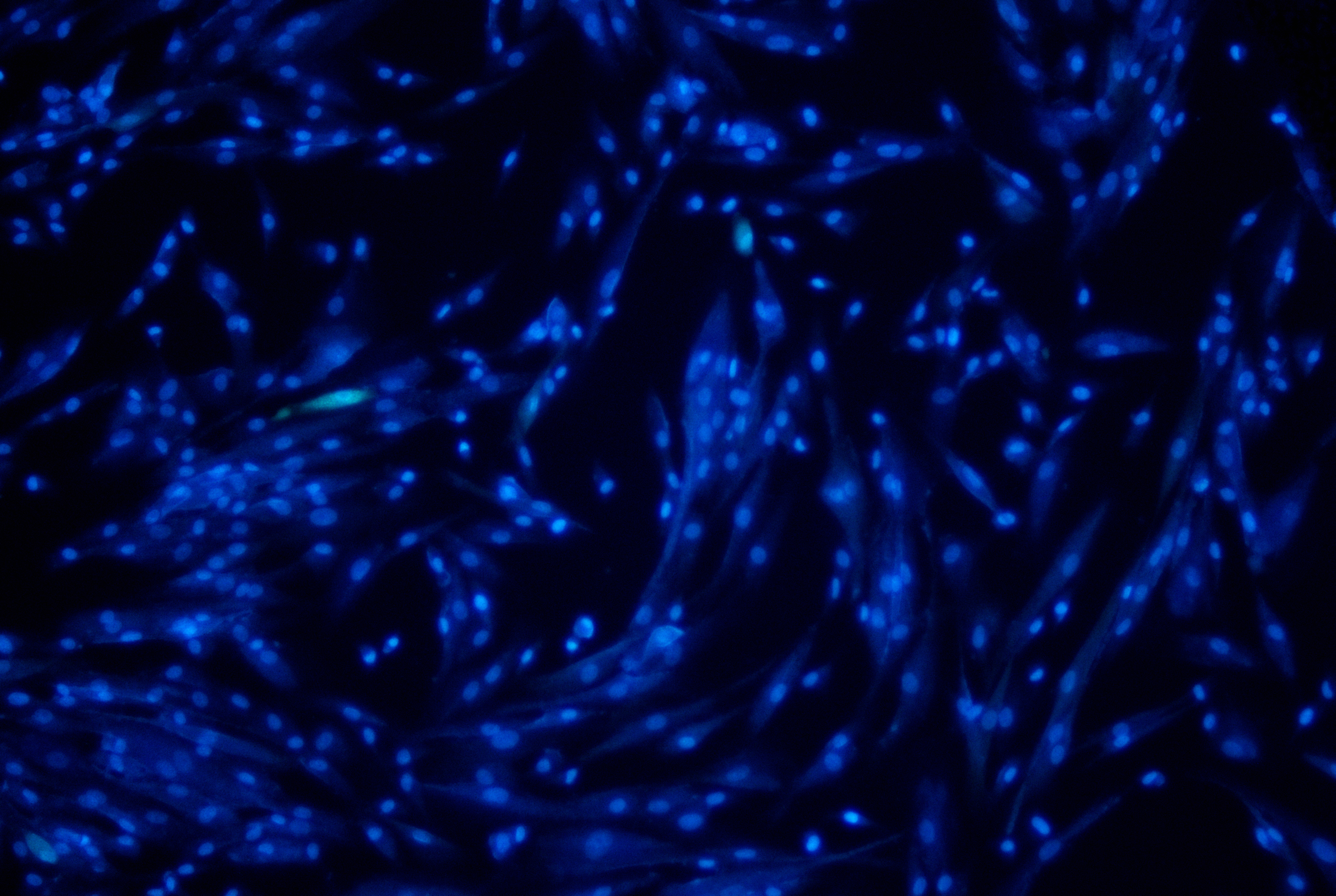

Supplement: Supplementary file 11 — Source data Fig. 6 [file 44318_2026_832_MOESM11_ESM.zip › C/WT sh90 DAPI.jpg]

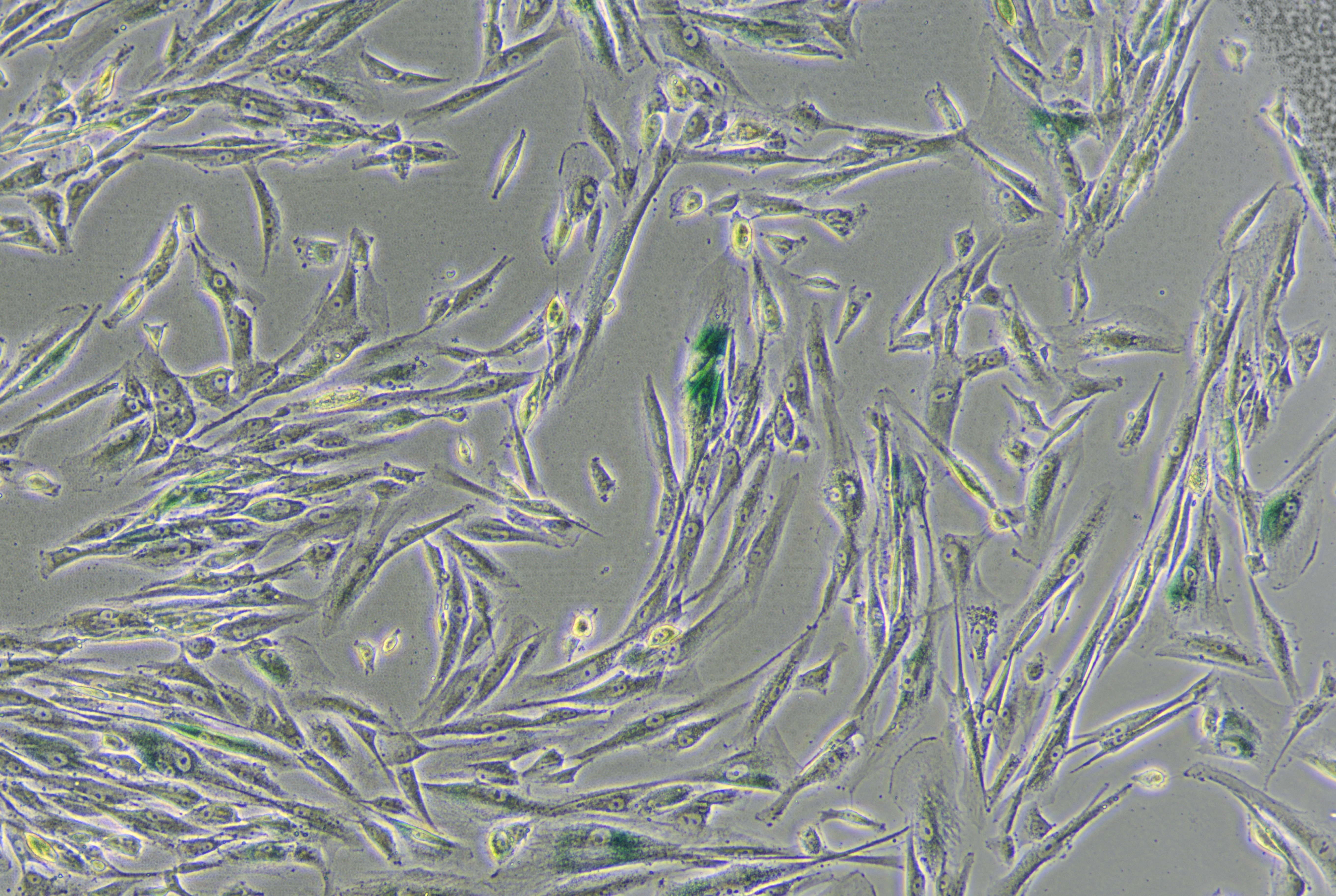

Supplement: Supplementary file 11 — Source data Fig. 6 [file 44318_2026_832_MOESM11_ESM.zip › C/WT sh90.jpg]

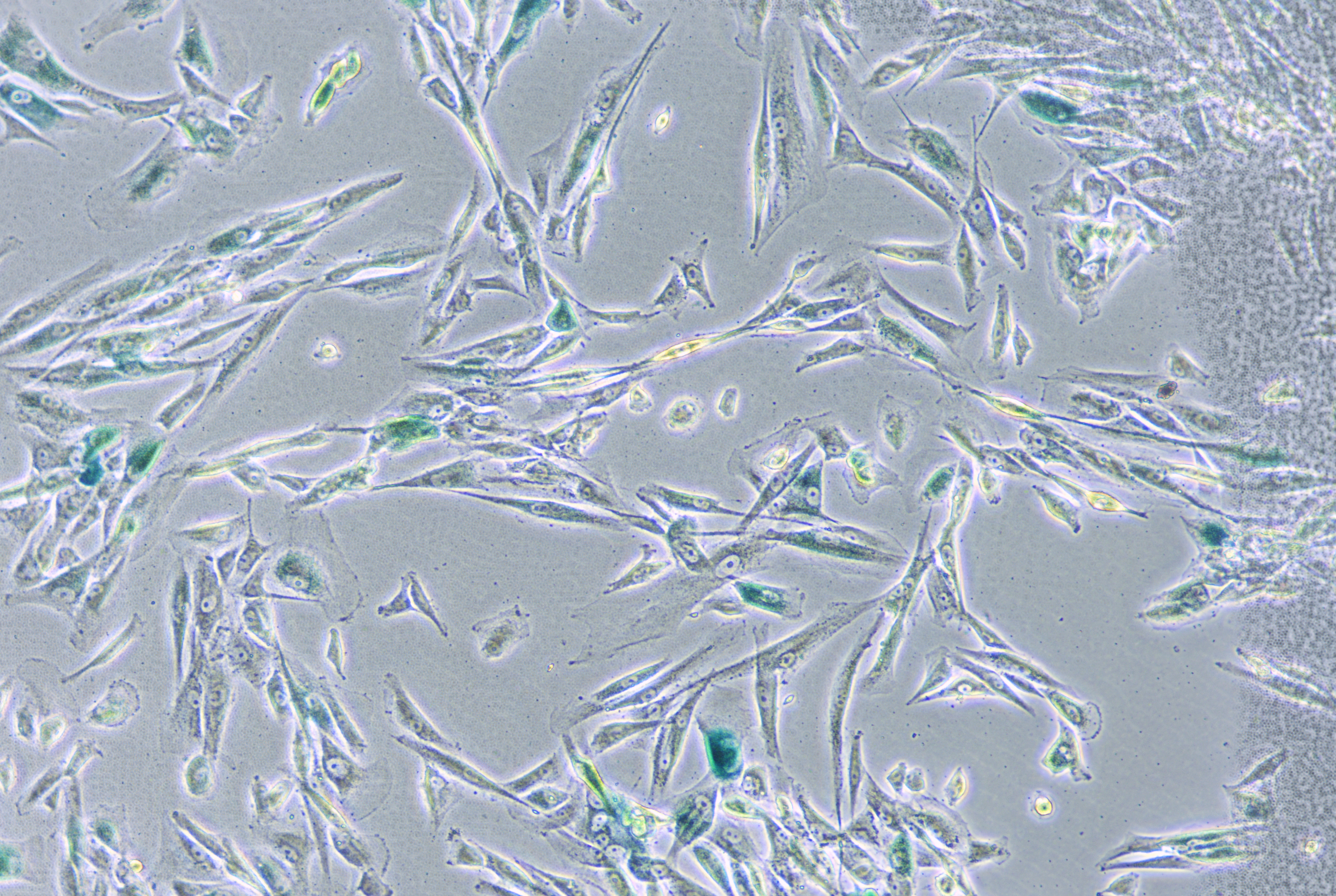

Supplement: Supplementary file 11 — Source data Fig. 6 [file 44318_2026_832_MOESM11_ESM.zip › C/WT.jpg]

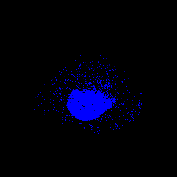

Supplement: Supplementary file 12 — Source data Fig. 7 [file 44318_2026_832_MOESM12_ESM.zip › A/G608G shHSP90+OE90+5ht-1.tif]

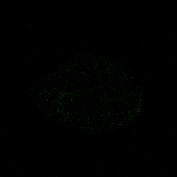

Supplement: Supplementary file 12 — Source data Fig. 7 [file 44318_2026_832_MOESM12_ESM.zip › A/G608G shHSP90+OE90+5ht-2.tif]

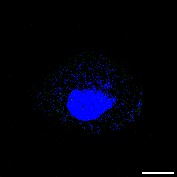

Supplement: Supplementary file 12 — Source data Fig. 7 [file 44318_2026_832_MOESM12_ESM.zip › A/G608G shHSP90+OE90+5ht-3.tif]

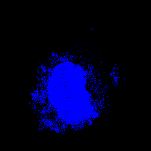

Supplement: Supplementary file 12 — Source data Fig. 7 [file 44318_2026_832_MOESM12_ESM.zip › A/G608G shHSP90+OE90-1.tif]

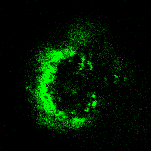

Supplement: Supplementary file 12 — Source data Fig. 7 [file 44318_2026_832_MOESM12_ESM.zip › A/G608G shHSP90+OE90-2.tif]

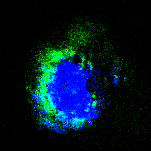

Supplement: Supplementary file 12 — Source data Fig. 7 [file 44318_2026_832_MOESM12_ESM.zip › A/G608G shHSP90+OE90-3.tif]

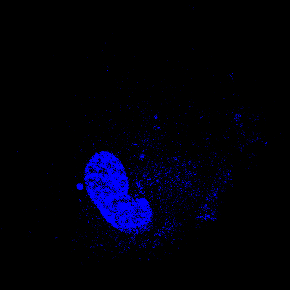

Supplement: Supplementary file 12 — Source data Fig. 7 [file 44318_2026_832_MOESM12_ESM.zip › A/G608G shHSP90+OEQ207A+5ht-1.tif]

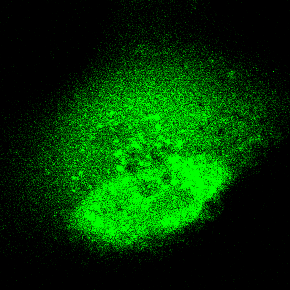

Supplement: Supplementary file 12 — Source data Fig. 7 [file 44318_2026_832_MOESM12_ESM.zip › A/G608G shHSP90+OEQ207A+5ht-2.tif]

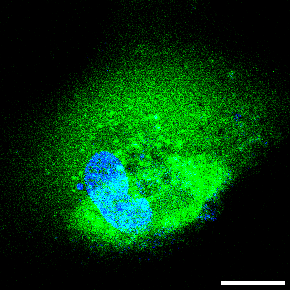

Supplement: Supplementary file 12 — Source data Fig. 7 [file 44318_2026_832_MOESM12_ESM.zip › A/G608G shHSP90+OEQ207A+5ht-3.tif]

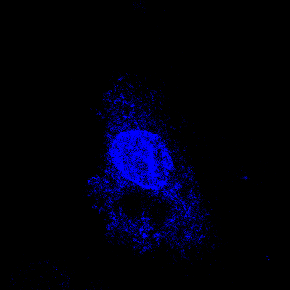

Supplement: Supplementary file 12 — Source data Fig. 7 [file 44318_2026_832_MOESM12_ESM.zip › A/G608G shHSP90+OEQ207A-1.tif]

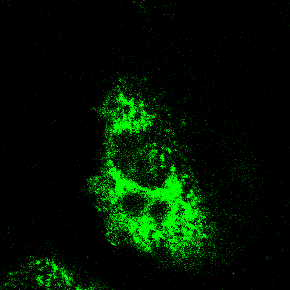

Supplement: Supplementary file 12 — Source data Fig. 7 [file 44318_2026_832_MOESM12_ESM.zip › A/G608G shHSP90+OEQ207A-2.tif]

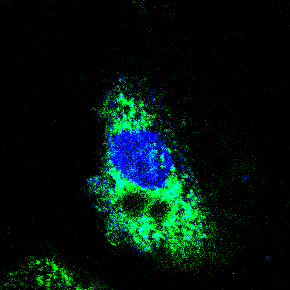

Supplement: Supplementary file 12 — Source data Fig. 7 [file 44318_2026_832_MOESM12_ESM.zip › A/G608G shHSP90+OEQ207A-3.tif]

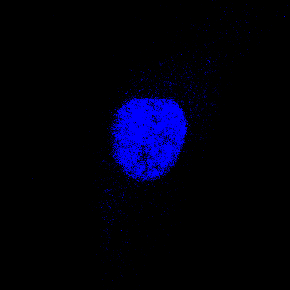

Supplement: Supplementary file 12 — Source data Fig. 7 [file 44318_2026_832_MOESM12_ESM.zip › A/G608G shHSP90+OEQ493A+5ht-1.tif]

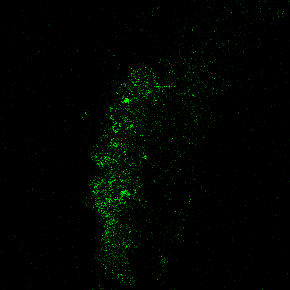

Supplement: Supplementary file 12 — Source data Fig. 7 [file 44318_2026_832_MOESM12_ESM.zip › A/G608G shHSP90+OEQ493A+5ht-2.tif]

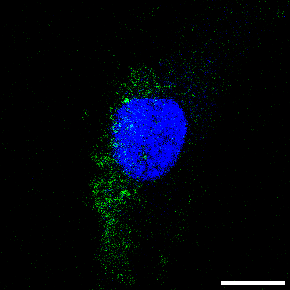

Supplement: Supplementary file 12 — Source data Fig. 7 [file 44318_2026_832_MOESM12_ESM.zip › A/G608G shHSP90+OEQ493A+5ht-3.tif]

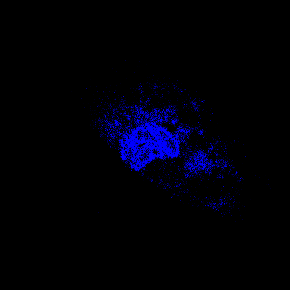

Supplement: Supplementary file 12 — Source data Fig. 7 [file 44318_2026_832_MOESM12_ESM.zip › A/G608G shHSP90+OEQ493A-1.tif]

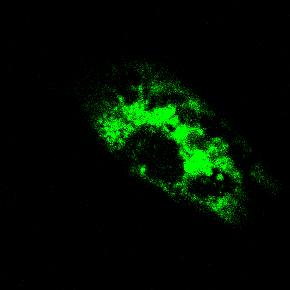

Supplement: Supplementary file 12 — Source data Fig. 7 [file 44318_2026_832_MOESM12_ESM.zip › A/G608G shHSP90+OEQ493A-2.tif]

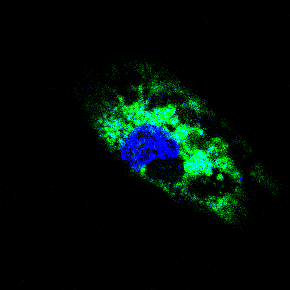

Supplement: Supplementary file 12 — Source data Fig. 7 [file 44318_2026_832_MOESM12_ESM.zip › A/G608G shHSP90+OEQ493A-3.tif]

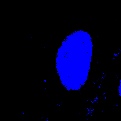

Supplement: Supplementary file 12 — Source data Fig. 7 [file 44318_2026_832_MOESM12_ESM.zip › A/G608G shHSP90-1-1.tif]

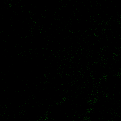

Supplement: Supplementary file 12 — Source data Fig. 7 [file 44318_2026_832_MOESM12_ESM.zip › A/G608G shHSP90-1-2.tif]

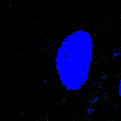

Supplement: Supplementary file 12 — Source data Fig. 7 [file 44318_2026_832_MOESM12_ESM.zip › A/G608G shHSP90-1-3.tif]

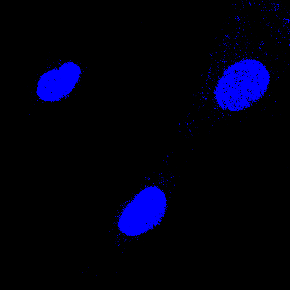

Supplement: Supplementary file 12 — Source data Fig. 7 [file 44318_2026_832_MOESM12_ESM.zip › A/G608G shHSP90-2-1.tif]

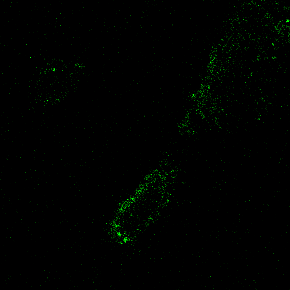

Supplement: Supplementary file 12 — Source data Fig. 7 [file 44318_2026_832_MOESM12_ESM.zip › A/G608G shHSP90-2-2.tif]

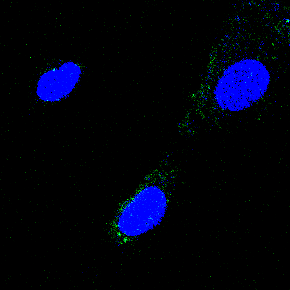

Supplement: Supplementary file 12 — Source data Fig. 7 [file 44318_2026_832_MOESM12_ESM.zip › A/G608G shHSP90-2-3.tif]

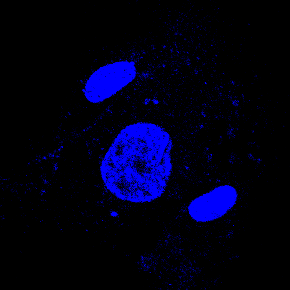

Supplement: Supplementary file 12 — Source data Fig. 7 [file 44318_2026_832_MOESM12_ESM.zip › A/G608G shHSP90-3-1.tif]

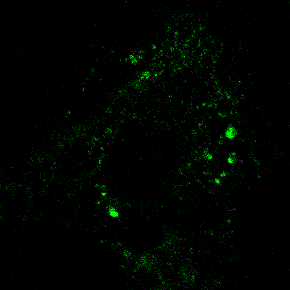

Supplement: Supplementary file 12 — Source data Fig. 7 [file 44318_2026_832_MOESM12_ESM.zip › A/G608G shHSP90-3-2.tif]

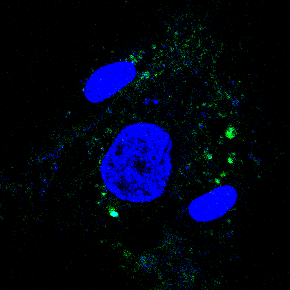

Supplement: Supplementary file 12 — Source data Fig. 7 [file 44318_2026_832_MOESM12_ESM.zip › A/G608G shHSP90-3-3.tif]

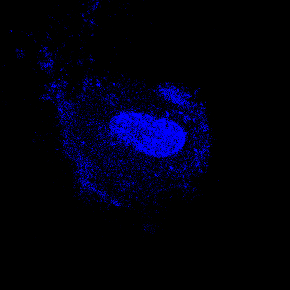

Supplement: Supplementary file 12 — Source data Fig. 7 [file 44318_2026_832_MOESM12_ESM.zip › B/WT shHSP90+OE Q207A+5ht-1.tif]

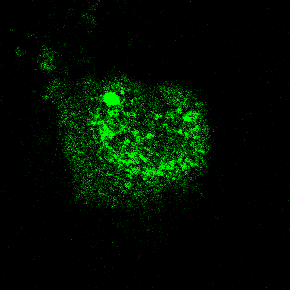

Supplement: Supplementary file 12 — Source data Fig. 7 [file 44318_2026_832_MOESM12_ESM.zip › B/WT shHSP90+OE Q207A+5ht-2.tif]

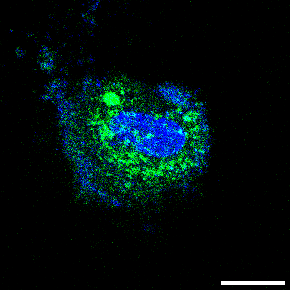

Supplement: Supplementary file 12 — Source data Fig. 7 [file 44318_2026_832_MOESM12_ESM.zip › B/WT shHSP90+OE Q207A+5ht-3.tif]

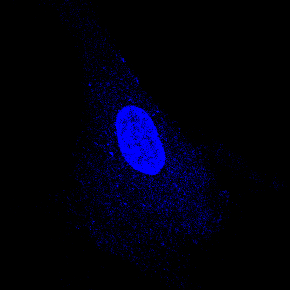

Supplement: Supplementary file 12 — Source data Fig. 7 [file 44318_2026_832_MOESM12_ESM.zip › B/WT shHSP90+OE Q207A-1.tif]

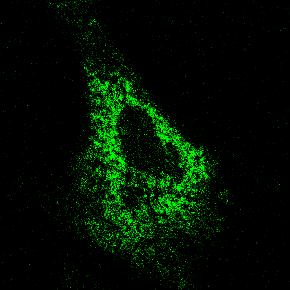

Supplement: Supplementary file 12 — Source data Fig. 7 [file 44318_2026_832_MOESM12_ESM.zip › B/WT shHSP90+OE Q207A-2.tif]

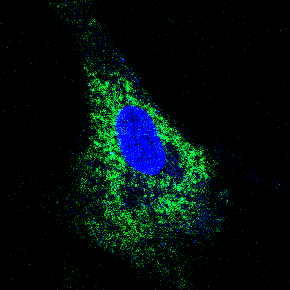

Supplement: Supplementary file 12 — Source data Fig. 7 [file 44318_2026_832_MOESM12_ESM.zip › B/WT shHSP90+OE Q207A3.tif]

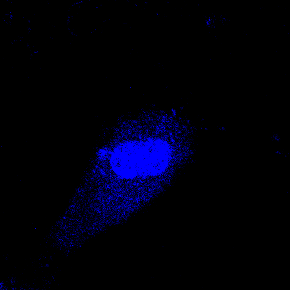

Supplement: Supplementary file 12 — Source data Fig. 7 [file 44318_2026_832_MOESM12_ESM.zip › B/WT shHSP90+OE Q493A-1.tif]

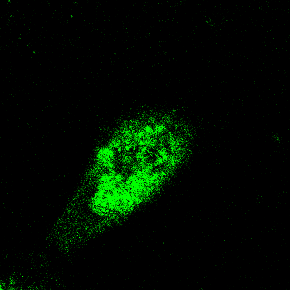

Supplement: Supplementary file 12 — Source data Fig. 7 [file 44318_2026_832_MOESM12_ESM.zip › B/WT shHSP90+OE Q493A-2.tif]

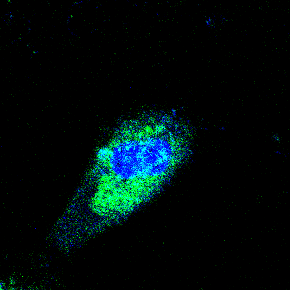

Supplement: Supplementary file 12 — Source data Fig. 7 [file 44318_2026_832_MOESM12_ESM.zip › B/WT shHSP90+OE Q493A-3.tif]

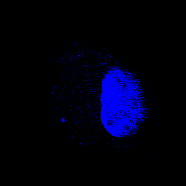

Supplement: Supplementary file 12 — Source data Fig. 7 [file 44318_2026_832_MOESM12_ESM.zip › B/WT shHSP90+OE90+5ht-1.tif]

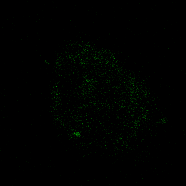

Supplement: Supplementary file 12 — Source data Fig. 7 [file 44318_2026_832_MOESM12_ESM.zip › B/WT shHSP90+OE90+5ht-2.tif]

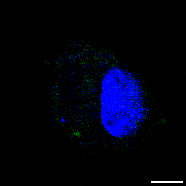

Supplement: Supplementary file 12 — Source data Fig. 7 [file 44318_2026_832_MOESM12_ESM.zip › B/WT shHSP90+OE90+5ht-3.tif]

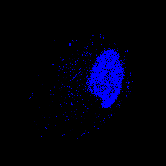

Supplement: Supplementary file 12 — Source data Fig. 7 [file 44318_2026_832_MOESM12_ESM.zip › B/WT shHSP90+OE90-1.tif]

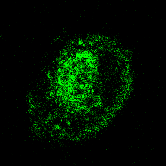

Supplement: Supplementary file 12 — Source data Fig. 7 [file 44318_2026_832_MOESM12_ESM.zip › B/WT shHSP90+OE90-2.tif]

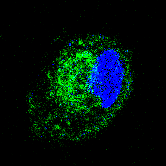

Supplement: Supplementary file 12 — Source data Fig. 7 [file 44318_2026_832_MOESM12_ESM.zip › B/WT shHSP90+OE90-3.tif]

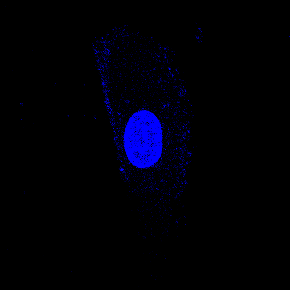

Supplement: Supplementary file 12 — Source data Fig. 7 [file 44318_2026_832_MOESM12_ESM.zip › B/WT shHSP90+OEQ493A+5ht-1.tif]

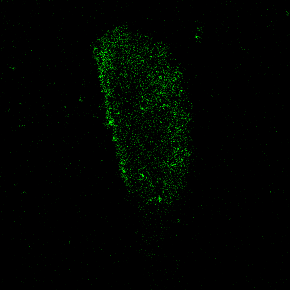

Supplement: Supplementary file 12 — Source data Fig. 7 [file 44318_2026_832_MOESM12_ESM.zip › B/WT shHSP90+OEQ493A+5ht-2.tif]

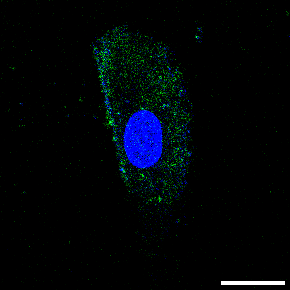

Supplement: Supplementary file 12 — Source data Fig. 7 [file 44318_2026_832_MOESM12_ESM.zip › B/WT shHSP90+OEQ493A+5ht-3.tif]

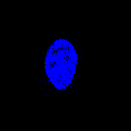

Supplement: Supplementary file 12 — Source data Fig. 7 [file 44318_2026_832_MOESM12_ESM.zip › B/WT shHSP90-1-1.tif]

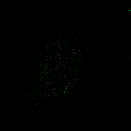

Supplement: Supplementary file 12 — Source data Fig. 7 [file 44318_2026_832_MOESM12_ESM.zip › B/WT shHSP90-1-2.tif]

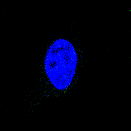

Supplement: Supplementary file 12 — Source data Fig. 7 [file 44318_2026_832_MOESM12_ESM.zip › B/WT shHSP90-1-3.tif]

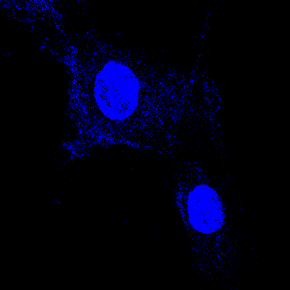

Supplement: Supplementary file 12 — Source data Fig. 7 [file 44318_2026_832_MOESM12_ESM.zip › B/WT shHSP90-2-1.tif]

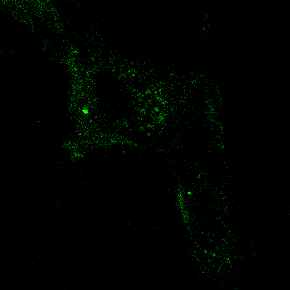

Supplement: Supplementary file 12 — Source data Fig. 7 [file 44318_2026_832_MOESM12_ESM.zip › B/WT shHSP90-2-2.tif]

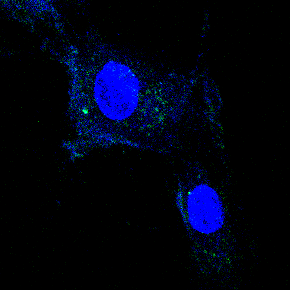

Supplement: Supplementary file 12 — Source data Fig. 7 [file 44318_2026_832_MOESM12_ESM.zip › B/WT shHSP90-2-3.tif]

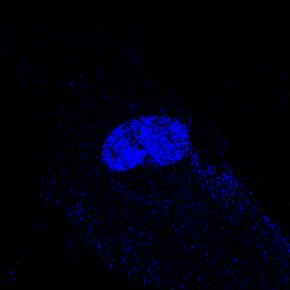

Supplement: Supplementary file 12 — Source data Fig. 7 [file 44318_2026_832_MOESM12_ESM.zip › B/WT shHSP90-3-1.tif]

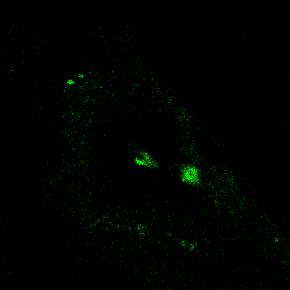

Supplement: Supplementary file 12 — Source data Fig. 7 [file 44318_2026_832_MOESM12_ESM.zip › B/WT shHSP90-3-2.tif]

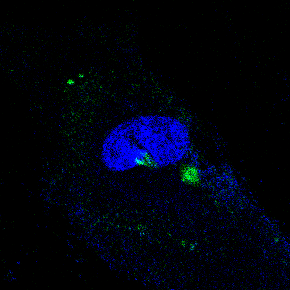

Supplement: Supplementary file 12 — Source data Fig. 7 [file 44318_2026_832_MOESM12_ESM.zip › B/WT shHSP90-3-3.tif]

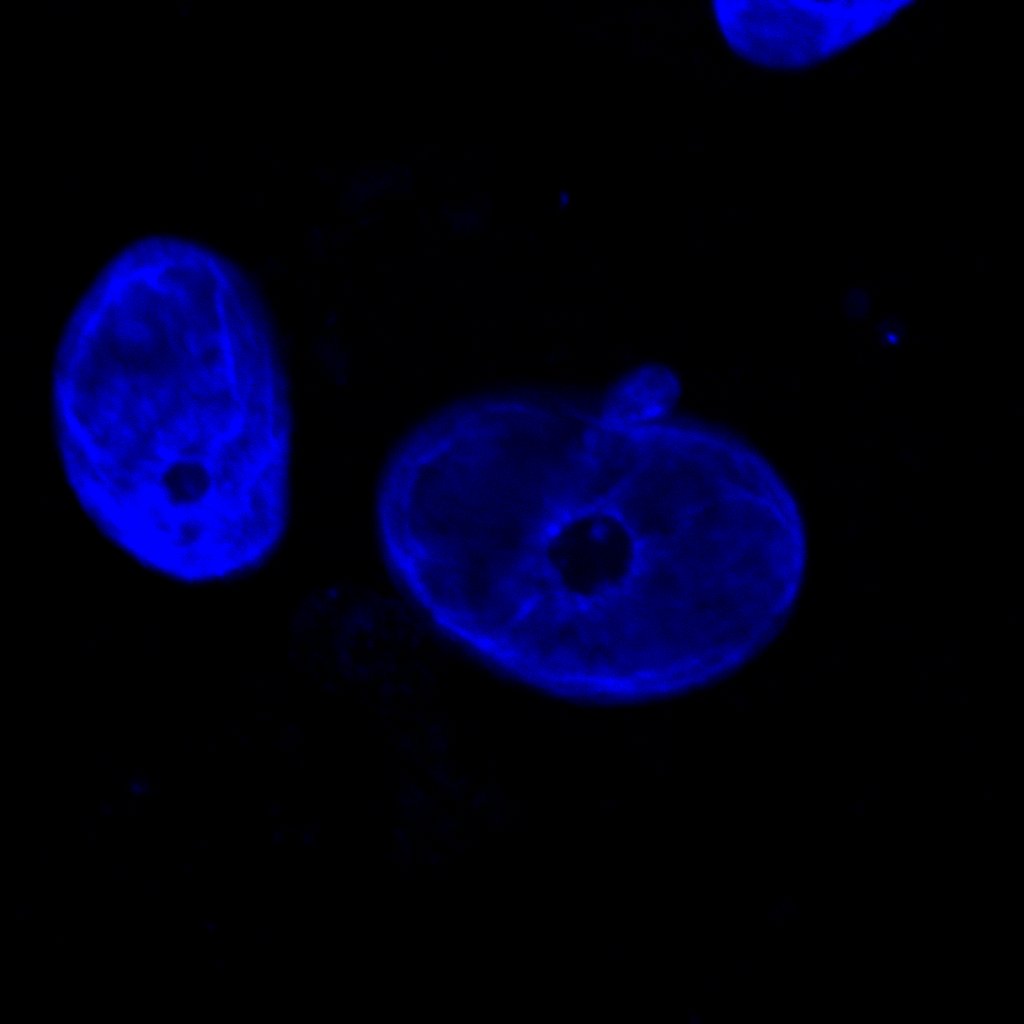

Supplement: Supplementary file 14 — Figure EV3 Source Data [file 44318_2026_832_MOESM14_ESM.zip › Expanded View Figure 3A/G608G DAPI.jpg]

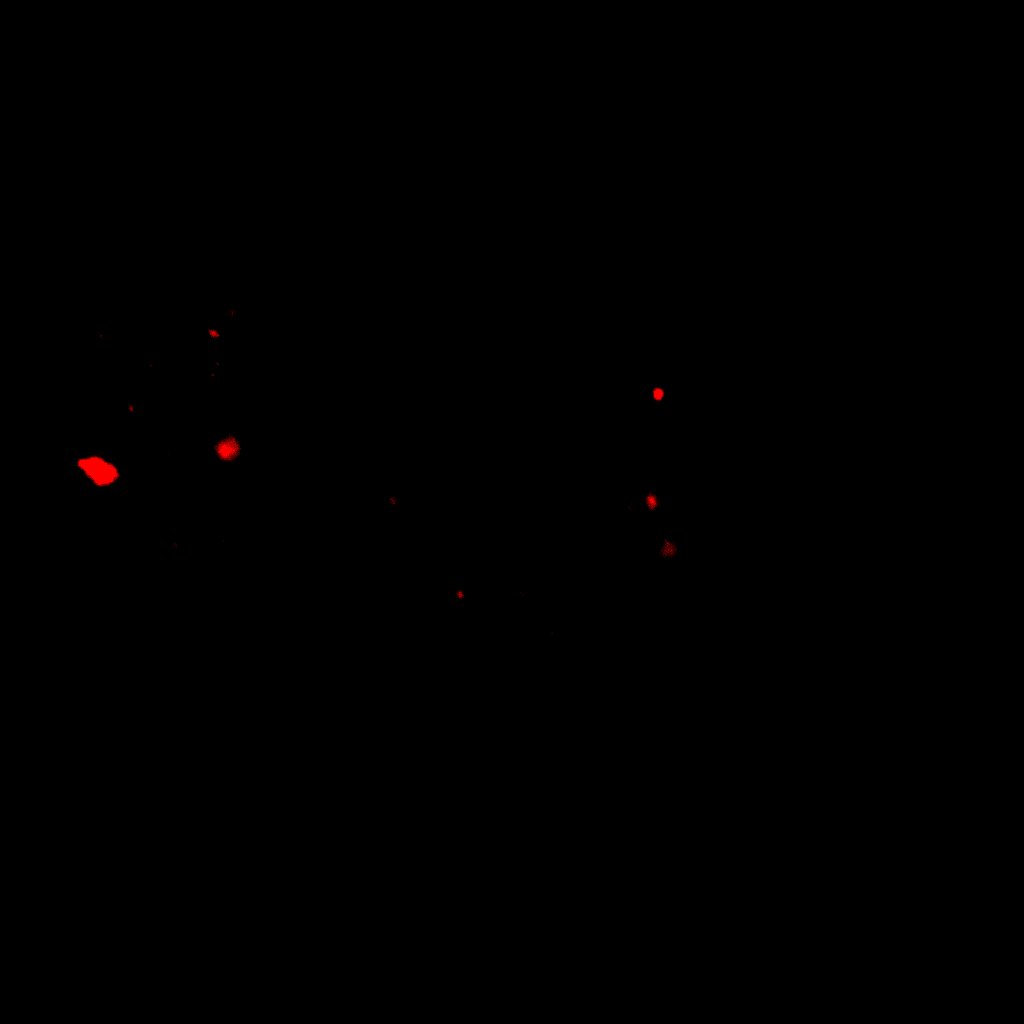

Supplement: Supplementary file 14 — Figure EV3 Source Data [file 44318_2026_832_MOESM14_ESM.zip › Expanded View Figure 3A/G608G H2AX.jpg]

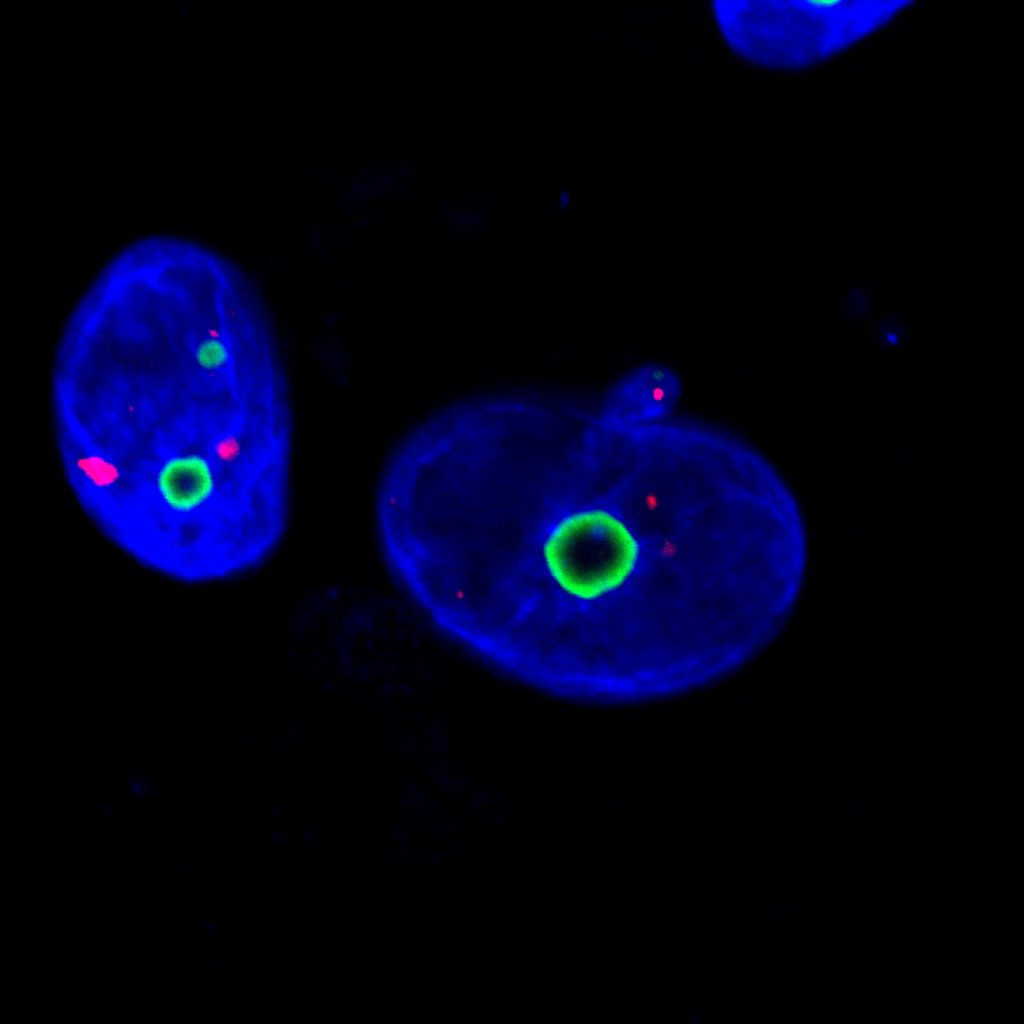

Supplement: Supplementary file 14 — Figure EV3 Source Data [file 44318_2026_832_MOESM14_ESM.zip › Expanded View Figure 3A/G608G merge.jpg]

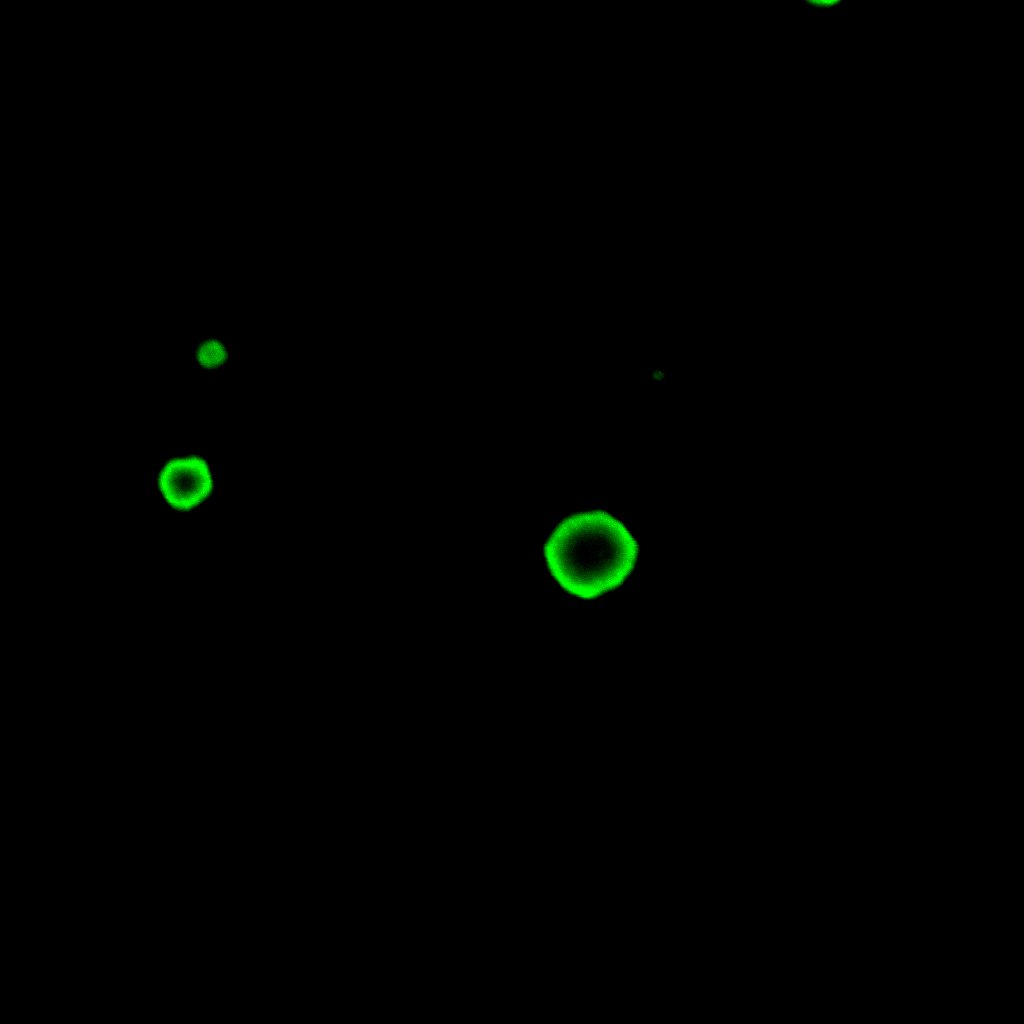

Supplement: Supplementary file 14 — Figure EV3 Source Data [file 44318_2026_832_MOESM14_ESM.zip › Expanded View Figure 3A/G608G NCL.jpg]

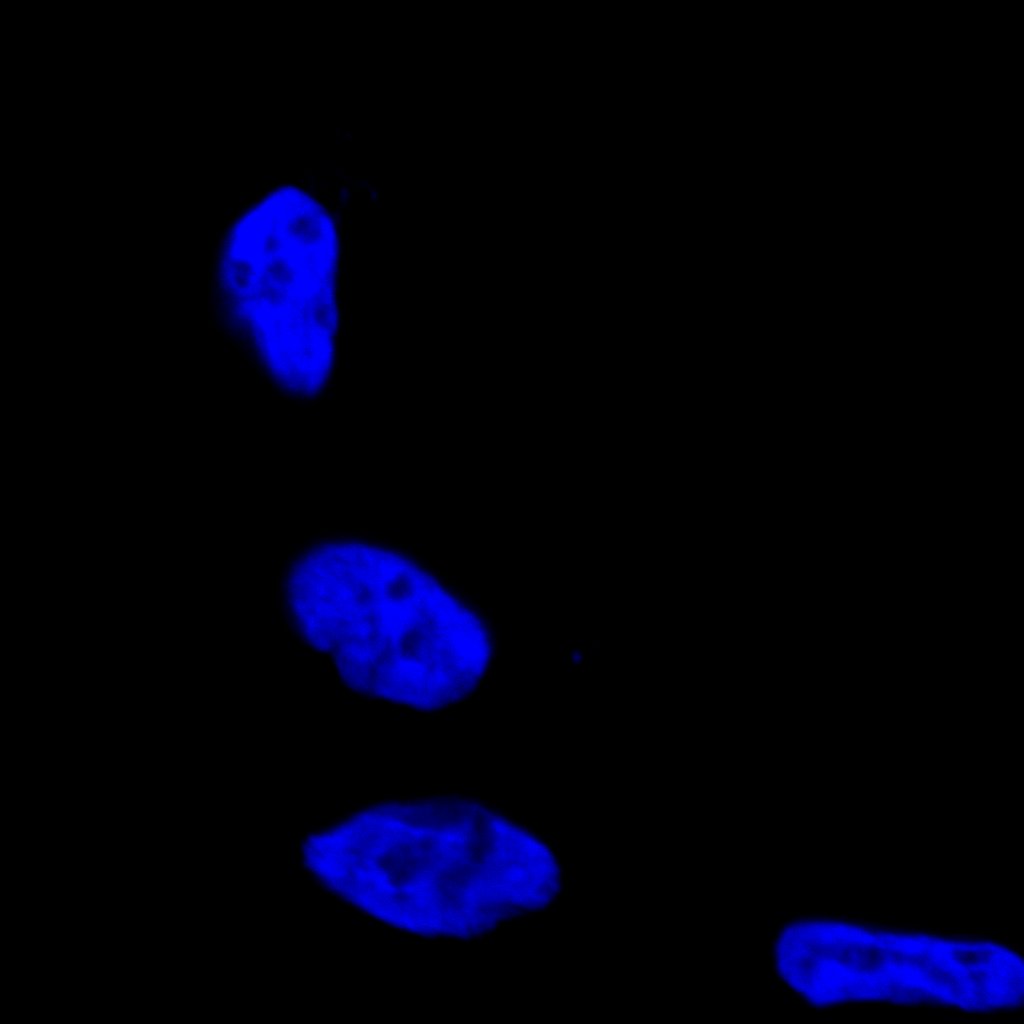

Supplement: Supplementary file 14 — Figure EV3 Source Data [file 44318_2026_832_MOESM14_ESM.zip › Expanded View Figure 3A/G608G+5ht DAPI.jpg]

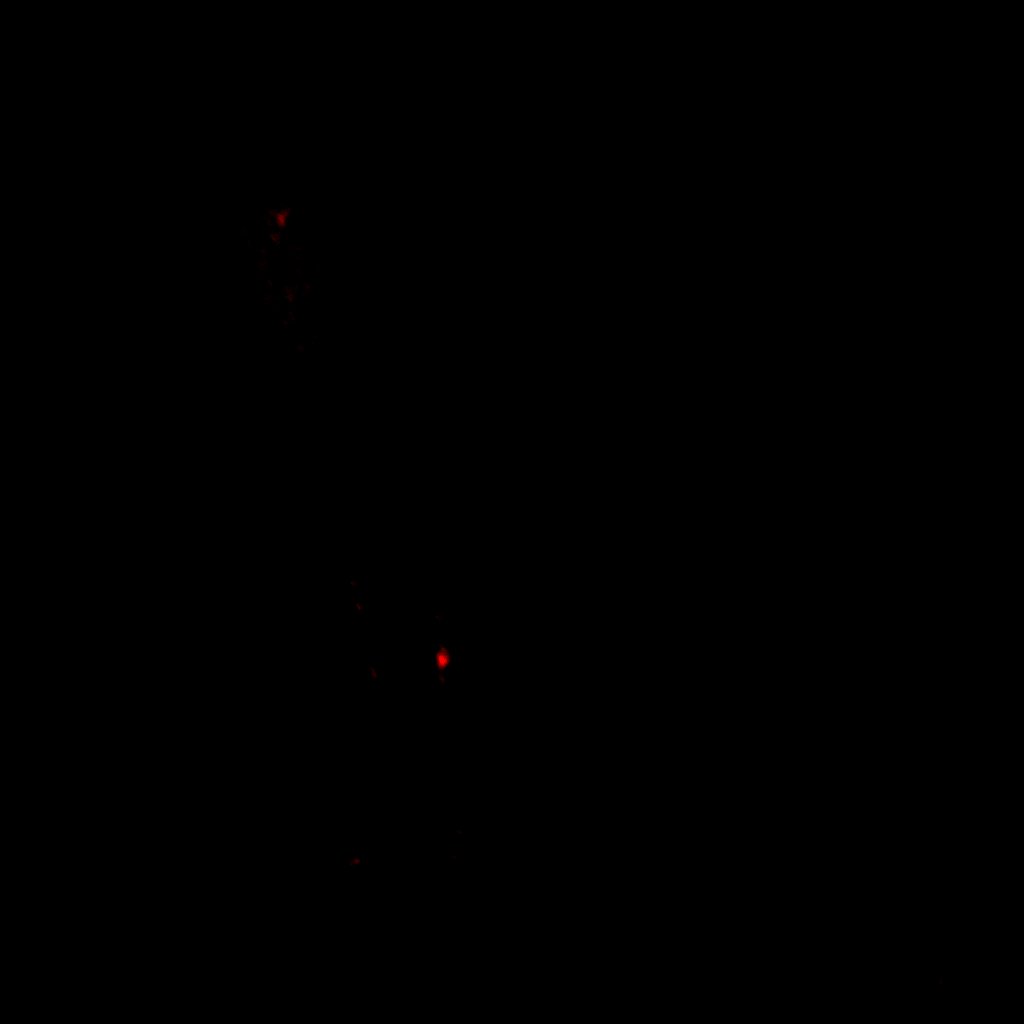

Supplement: Supplementary file 14 — Figure EV3 Source Data [file 44318_2026_832_MOESM14_ESM.zip › Expanded View Figure 3A/G608G+5ht H2AX.jpg]

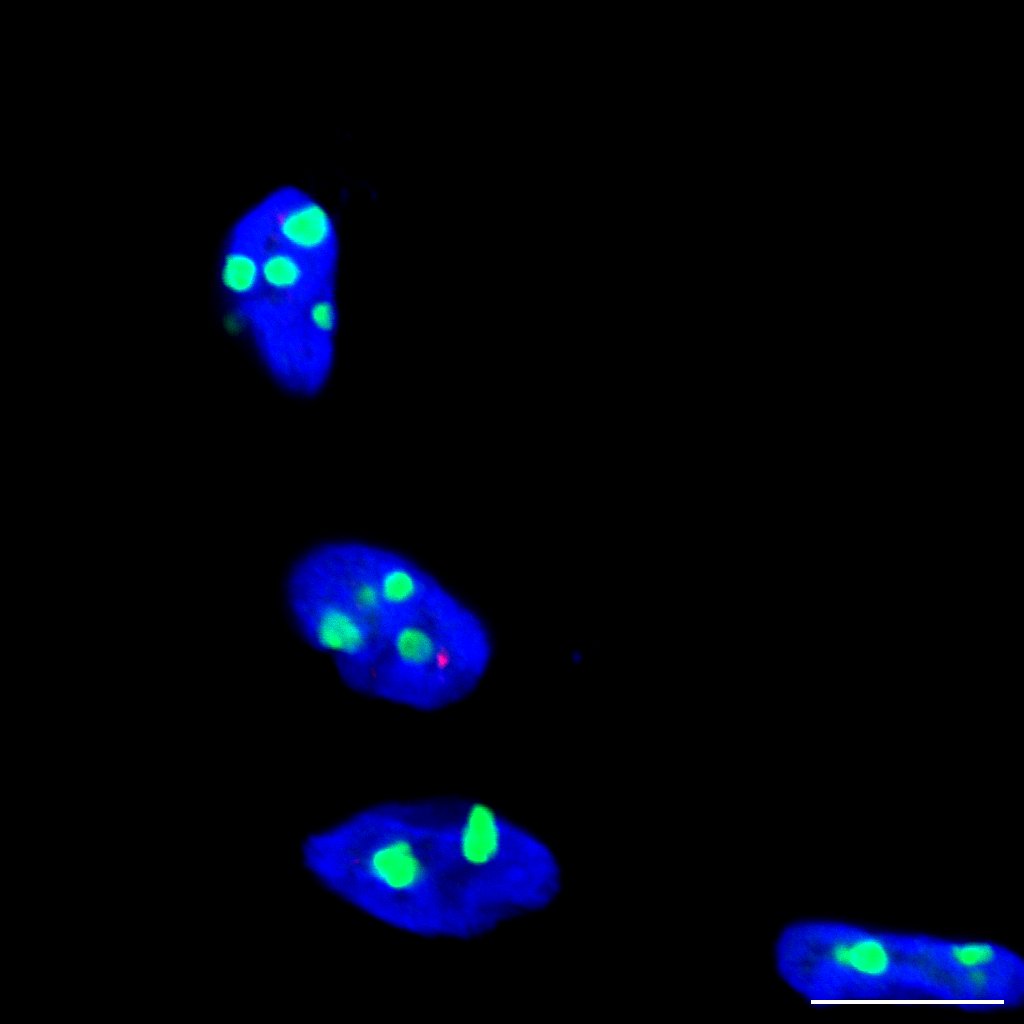

Supplement: Supplementary file 14 — Figure EV3 Source Data [file 44318_2026_832_MOESM14_ESM.zip › Expanded View Figure 3A/G608G+5ht merge.jpg]

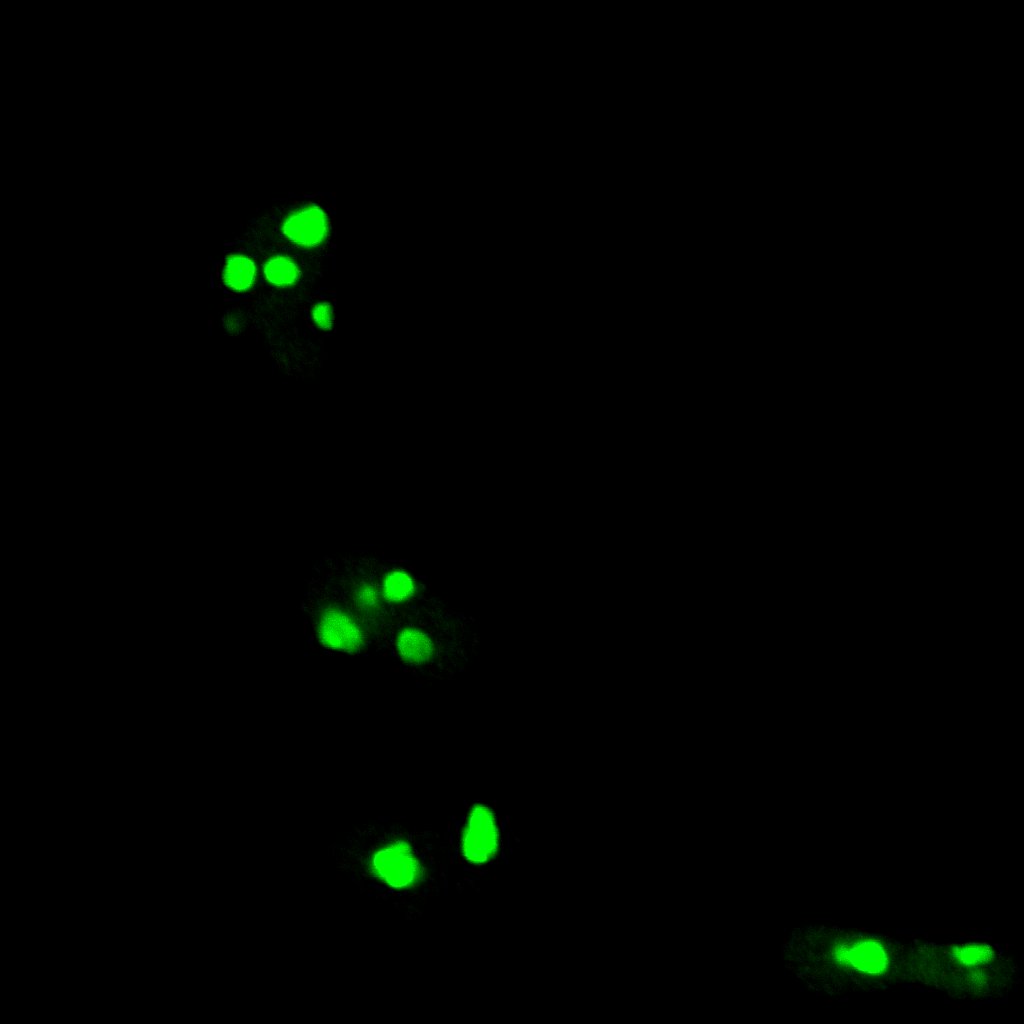

Supplement: Supplementary file 14 — Figure EV3 Source Data [file 44318_2026_832_MOESM14_ESM.zip › Expanded View Figure 3A/G608G+5ht NCL.jpg]

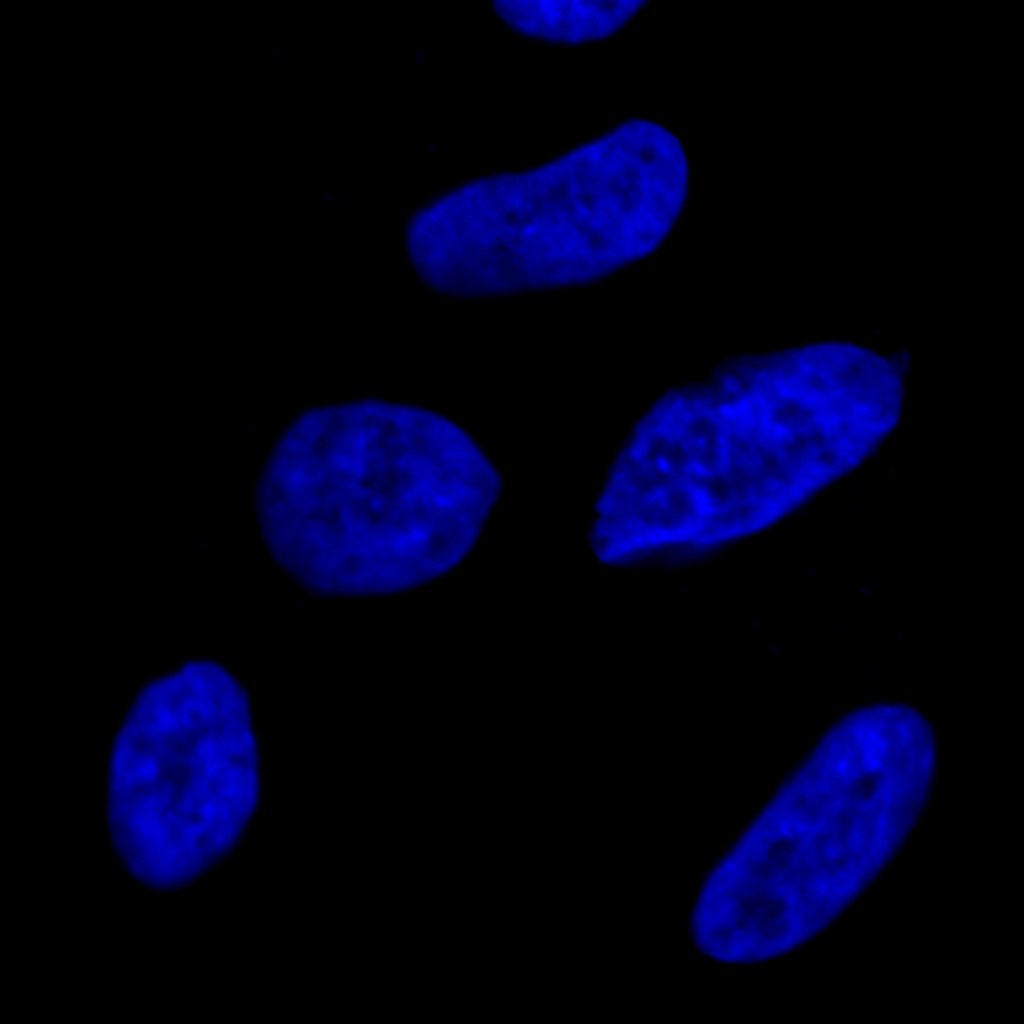

Supplement: Supplementary file 14 — Figure EV3 Source Data [file 44318_2026_832_MOESM14_ESM.zip › Expanded View Figure 3A/WT DAPI.jpg]
