# Supplementary figures and images for: The oocyte-enriched metabolite serotonin alleviates cellular senescence and aging phenotypes in the mouse (part 3 of 3)
Source: EMBO J. 2026 Jun 16;45(14):4849–86. doi: 10.1038/s44318-026-00832-x (PMC13373241; doi:10.1038/s44318-026-00832-x)

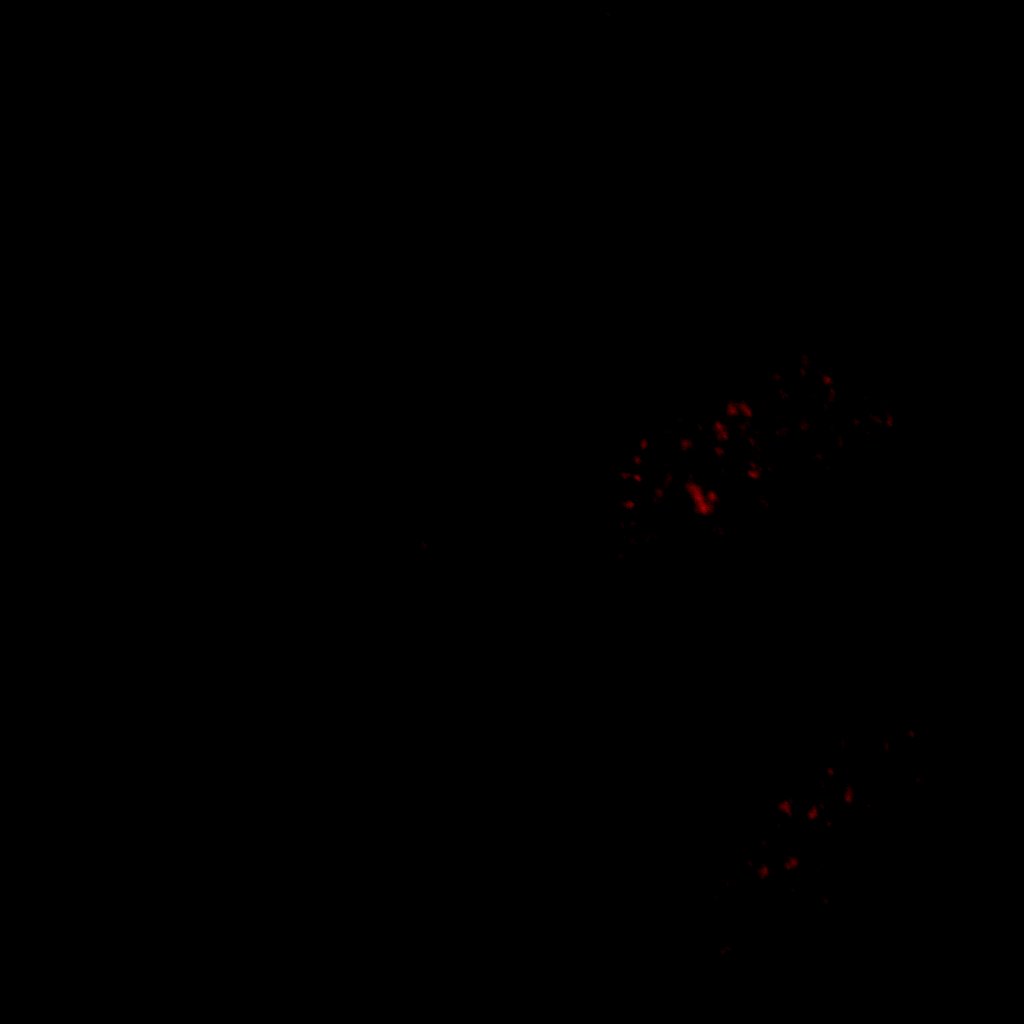

Supplement: Supplementary file 14 — Figure EV3 Source Data [file 44318_2026_832_MOESM14_ESM.zip › Expanded View Figure 3A/WT H2AX.jpg]

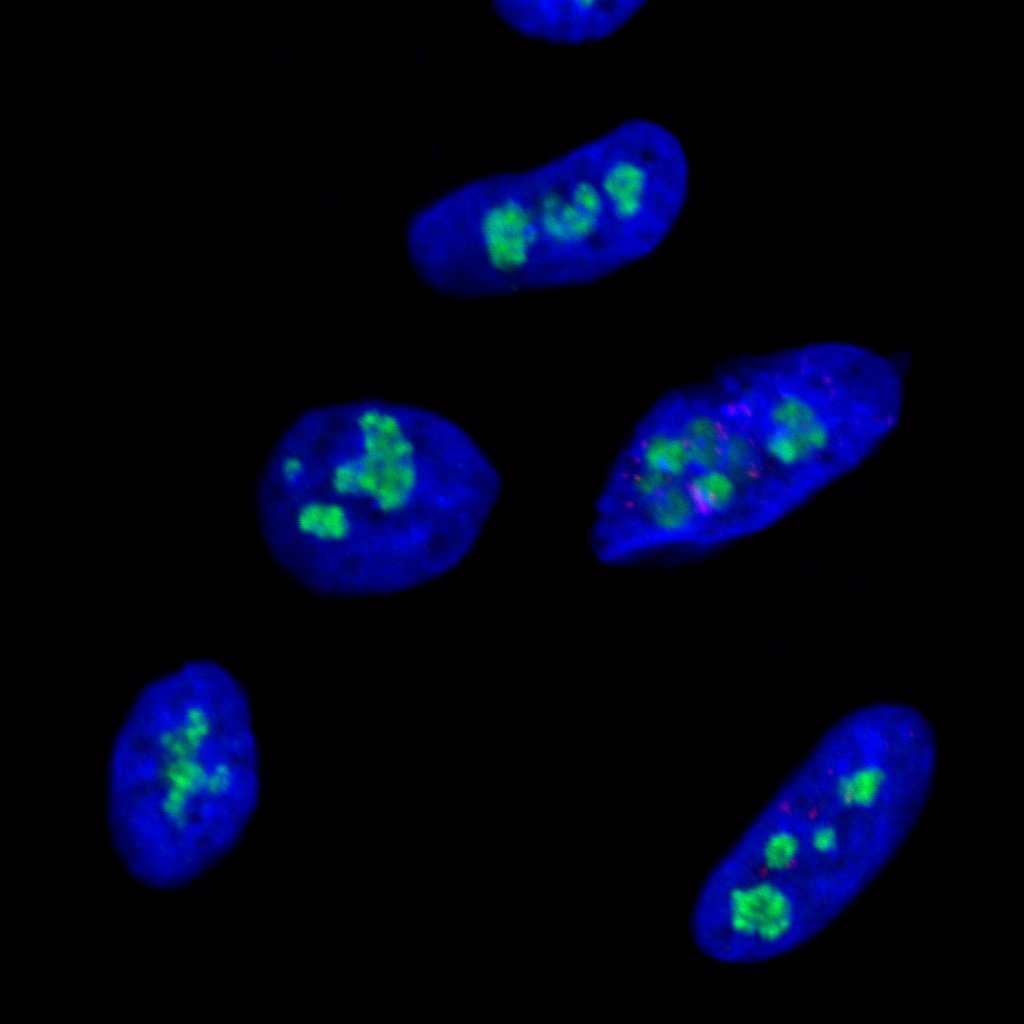

Supplement: Supplementary file 14 — Figure EV3 Source Data [file 44318_2026_832_MOESM14_ESM.zip › Expanded View Figure 3A/WT merge.jpg]

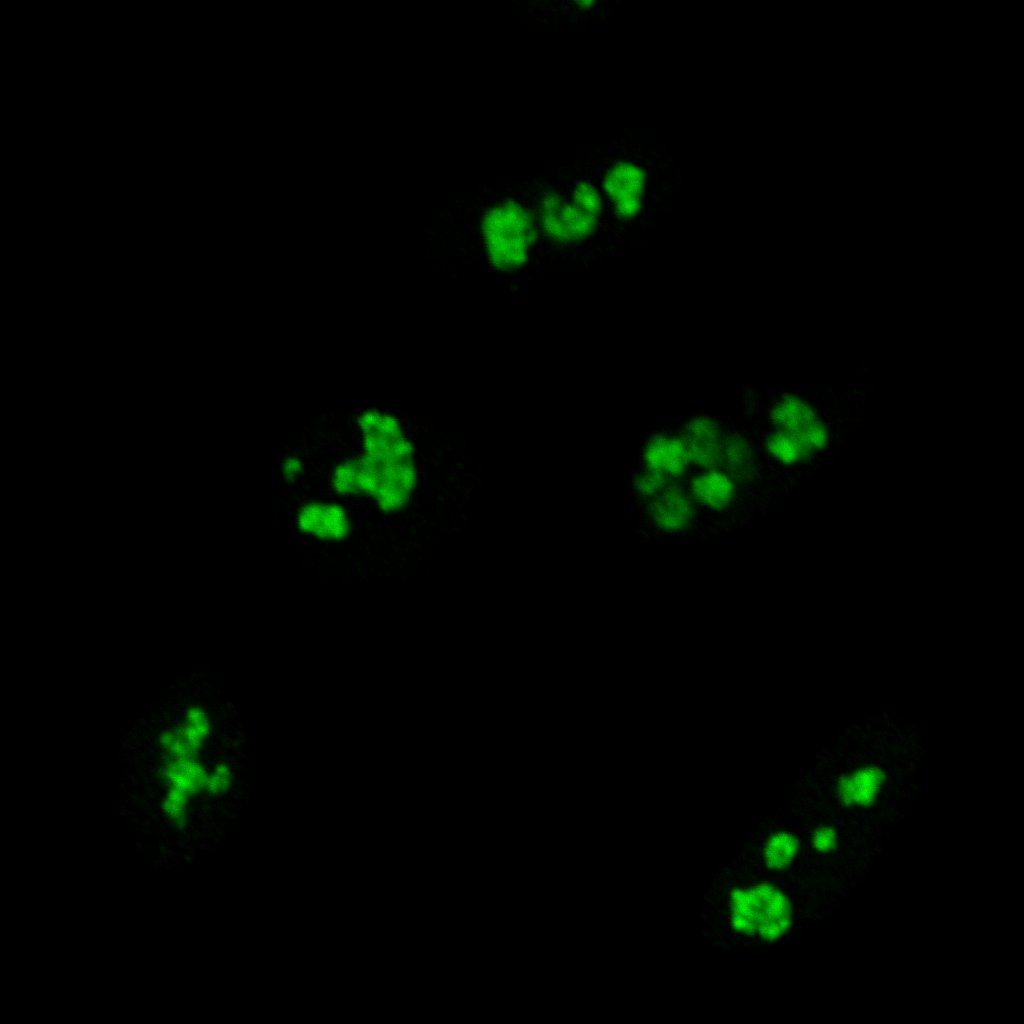

Supplement: Supplementary file 14 — Figure EV3 Source Data [file 44318_2026_832_MOESM14_ESM.zip › Expanded View Figure 3A/WT NCL.jpg]

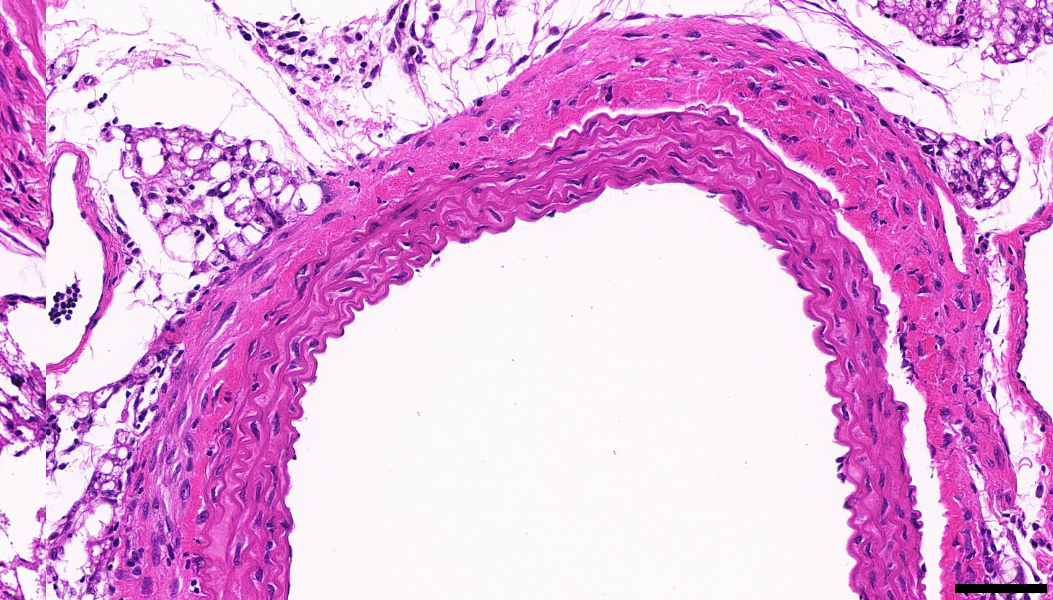

Supplement: Supplementary file 14 — Figure EV3 Source Data [file 44318_2026_832_MOESM14_ESM.zip › Expanded View Figure 3H/G608G aorta.jpg]

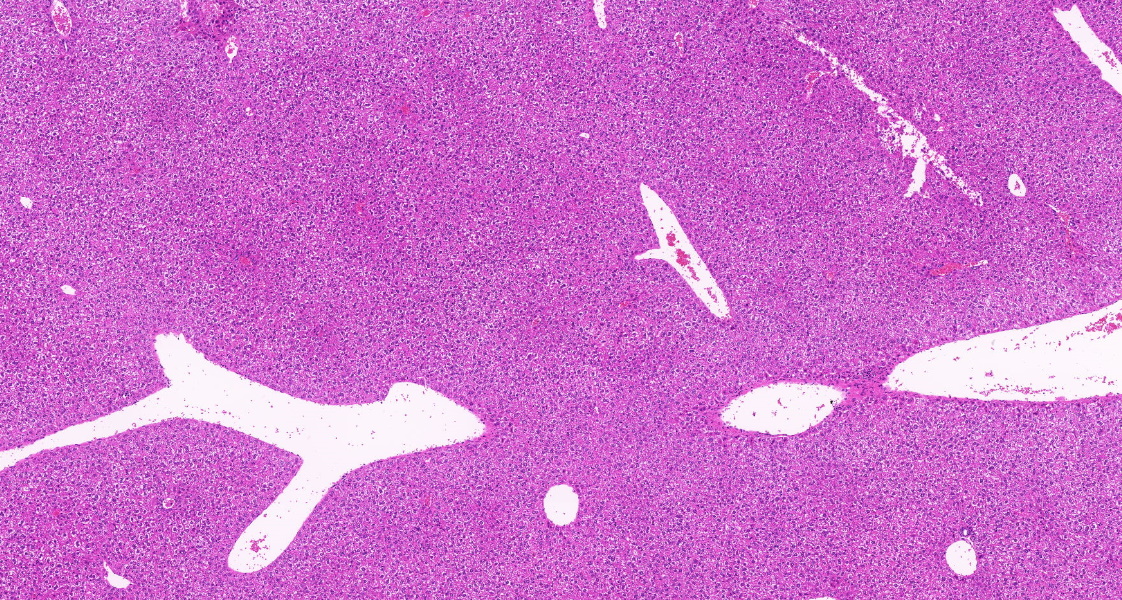

Supplement: Supplementary file 14 — Figure EV3 Source Data [file 44318_2026_832_MOESM14_ESM.zip › Expanded View Figure 3H/G608G liver.jpg]

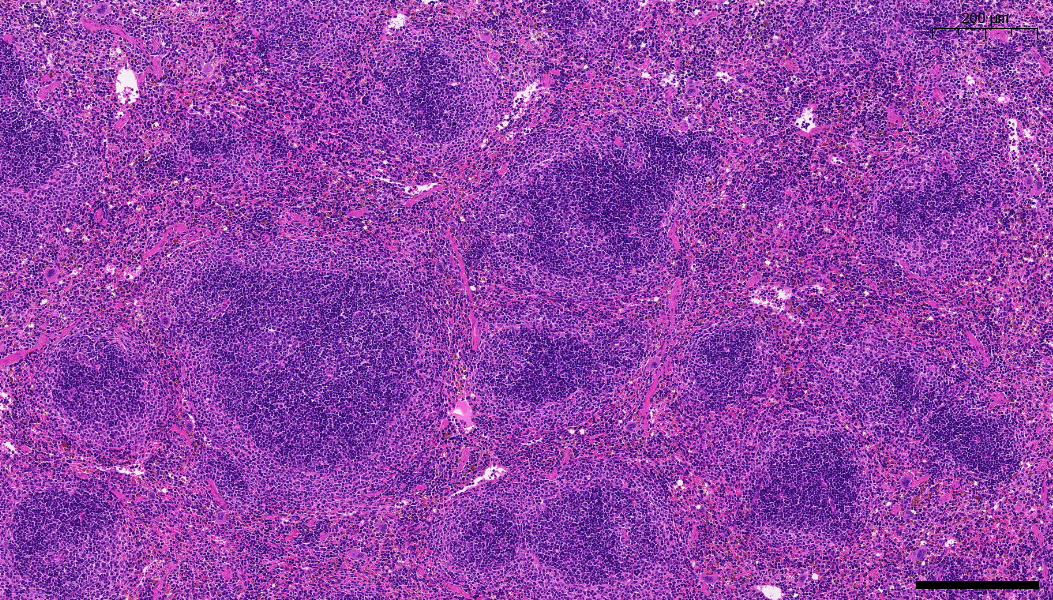

Supplement: Supplementary file 14 — Figure EV3 Source Data [file 44318_2026_832_MOESM14_ESM.zip › Expanded View Figure 3H/G608G spleen.jpg]

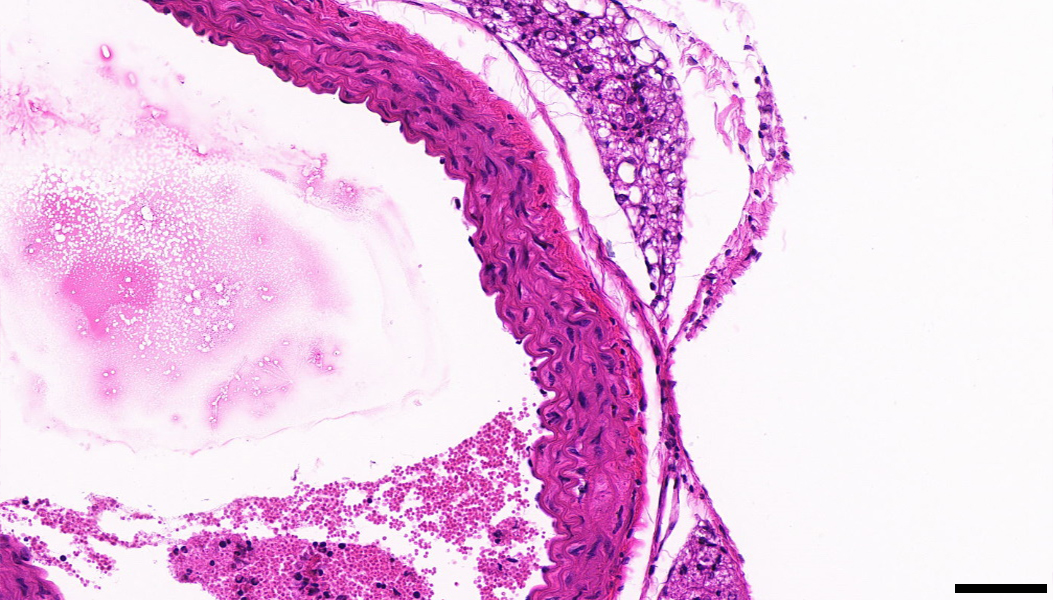

Supplement: Supplementary file 14 — Figure EV3 Source Data [file 44318_2026_832_MOESM14_ESM.zip › Expanded View Figure 3H/G608G+5ht aorta.jpg]

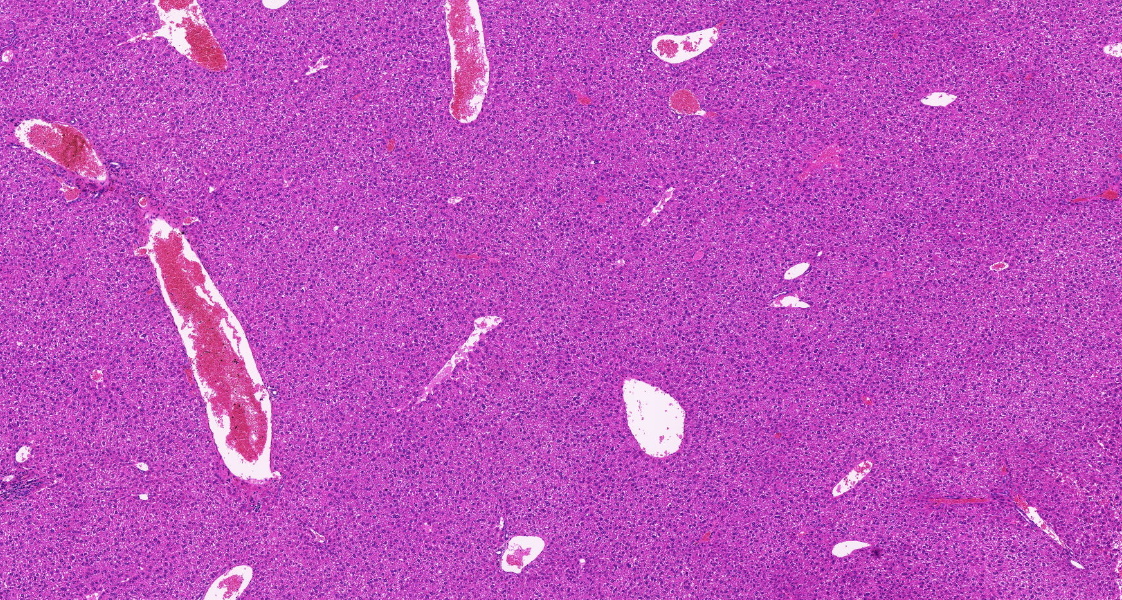

Supplement: Supplementary file 14 — Figure EV3 Source Data [file 44318_2026_832_MOESM14_ESM.zip › Expanded View Figure 3H/G608G+5ht liver.jpg]

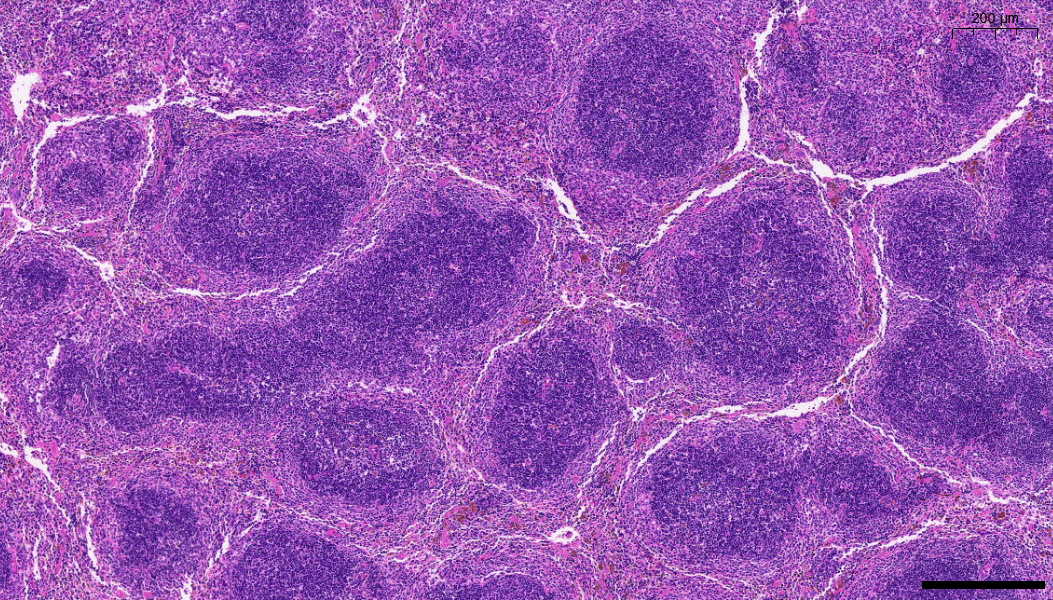

Supplement: Supplementary file 14 — Figure EV3 Source Data [file 44318_2026_832_MOESM14_ESM.zip › Expanded View Figure 3H/G608G+5ht spleen.jpg]

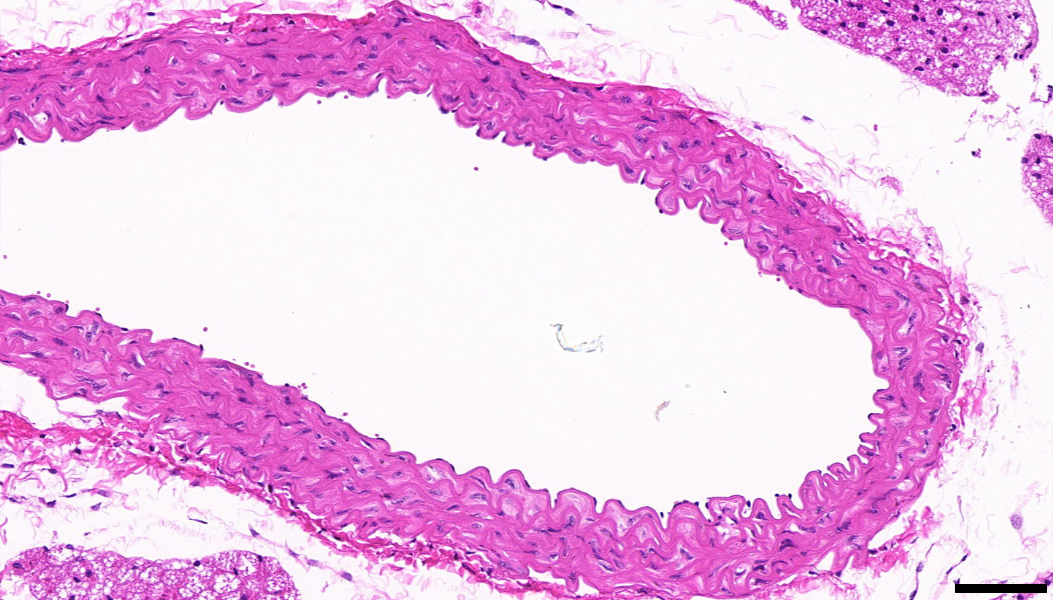

Supplement: Supplementary file 14 — Figure EV3 Source Data [file 44318_2026_832_MOESM14_ESM.zip › Expanded View Figure 3H/WT aorta.jpg]

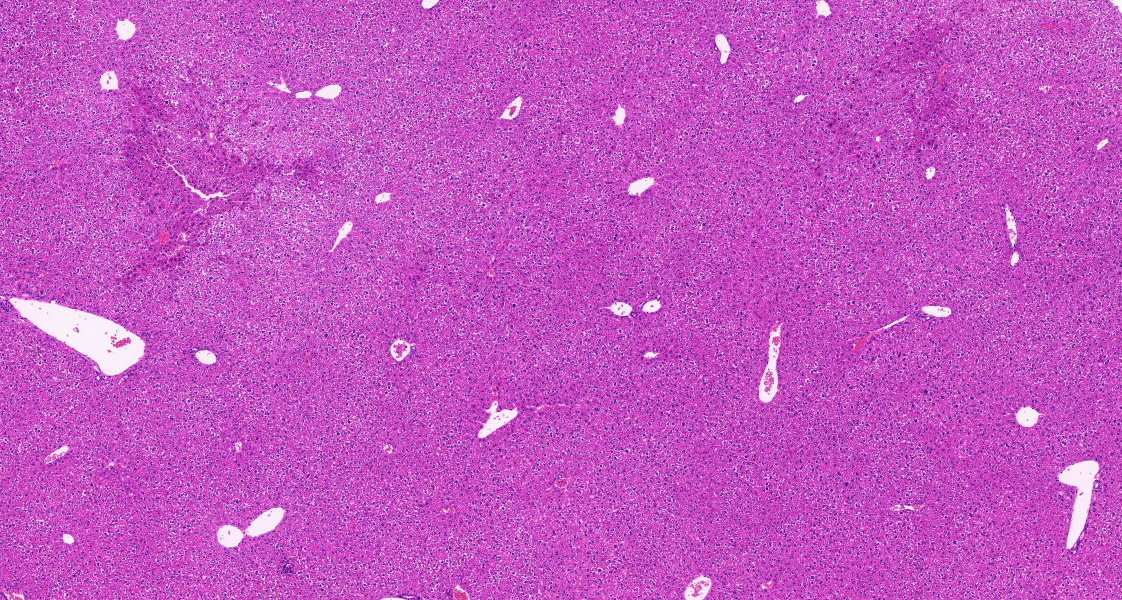

Supplement: Supplementary file 14 — Figure EV3 Source Data [file 44318_2026_832_MOESM14_ESM.zip › Expanded View Figure 3H/WT liver .jpg]

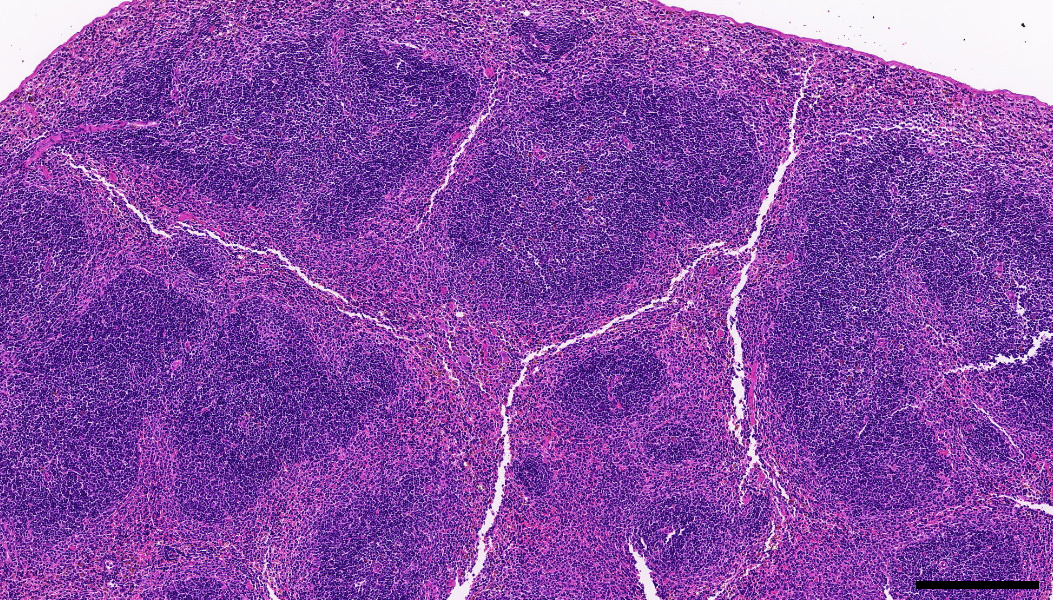

Supplement: Supplementary file 14 — Figure EV3 Source Data [file 44318_2026_832_MOESM14_ESM.zip › Expanded View Figure 3H/WT spleen.jpg]

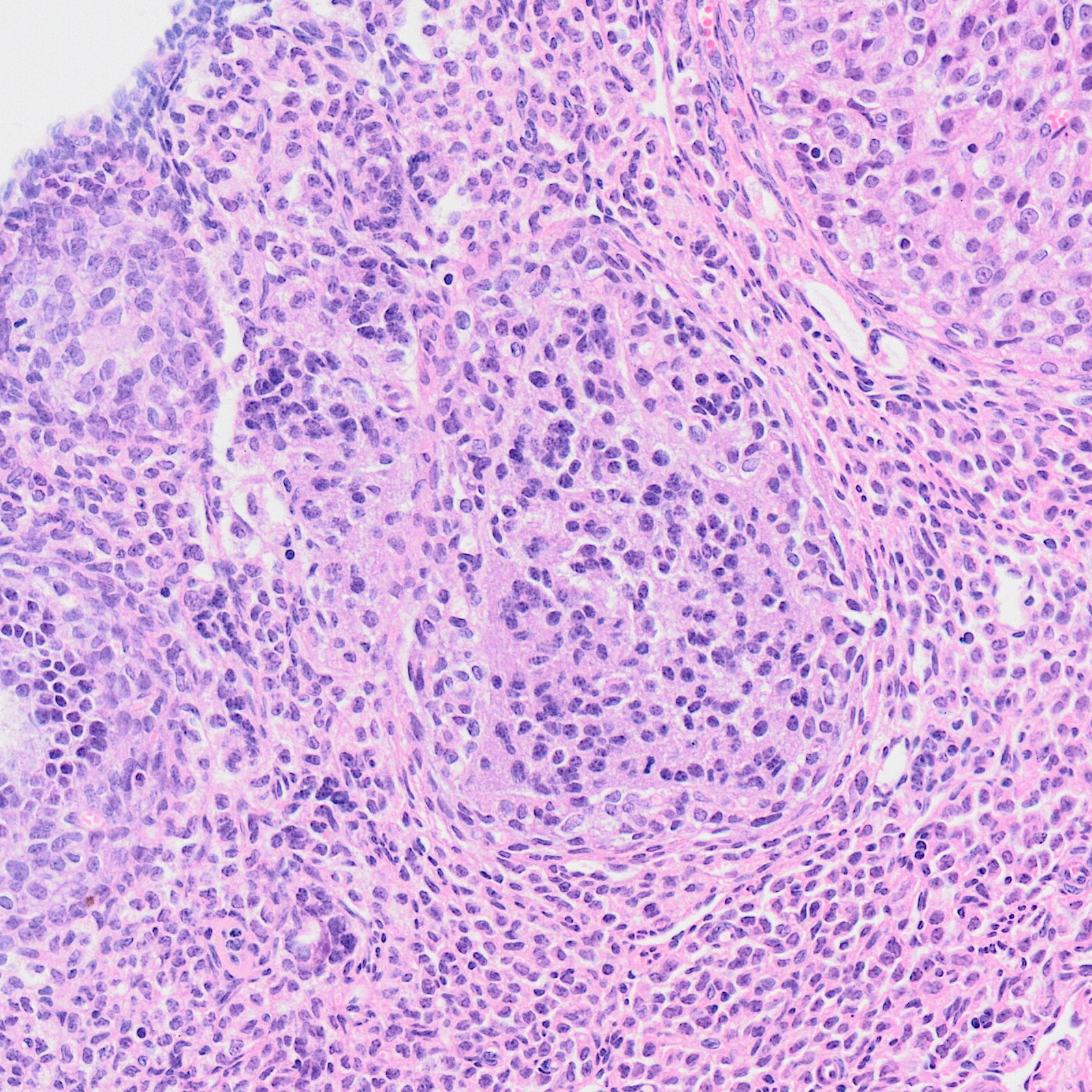

Supplement: Supplementary file 14 — Figure EV3 Source Data [file 44318_2026_832_MOESM14_ESM.zip › Expanded View Figure 3L/G608G ovary-large.tif]

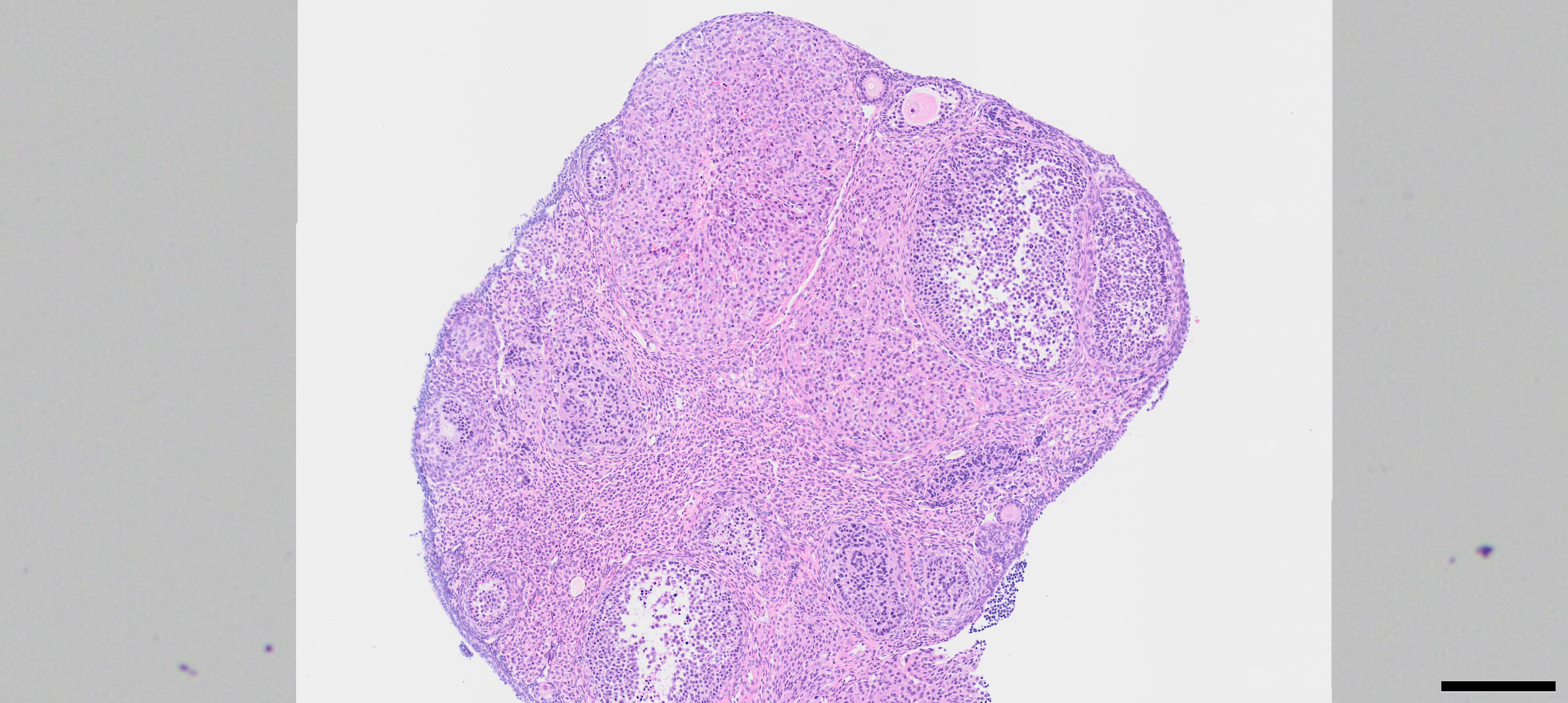

Supplement: Supplementary file 14 — Figure EV3 Source Data [file 44318_2026_832_MOESM14_ESM.zip › Expanded View Figure 3L/G608G ovary.tif]

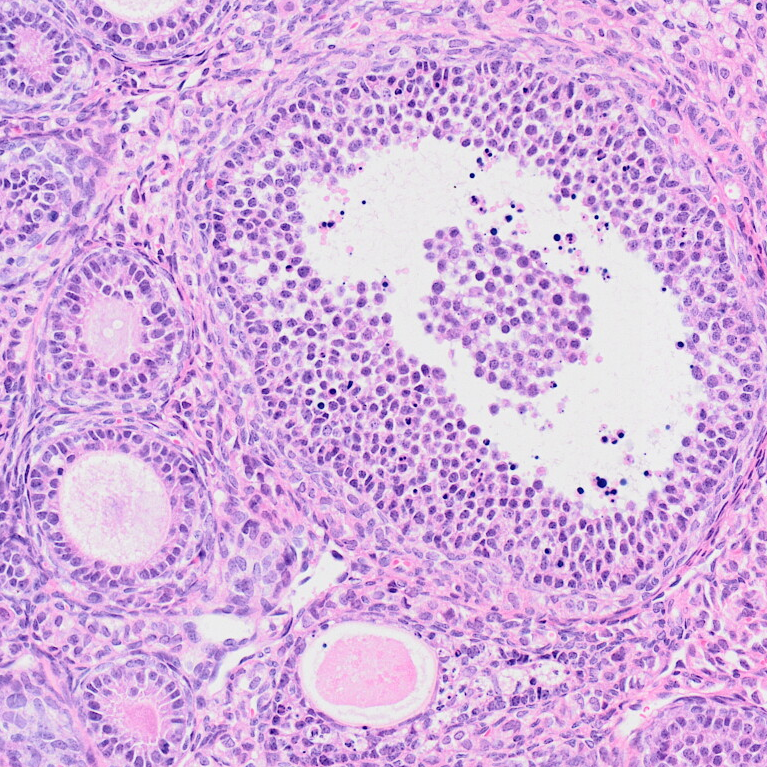

Supplement: Supplementary file 14 — Figure EV3 Source Data [file 44318_2026_832_MOESM14_ESM.zip › Expanded View Figure 3L/G608G+5ht ovary-large.tif]

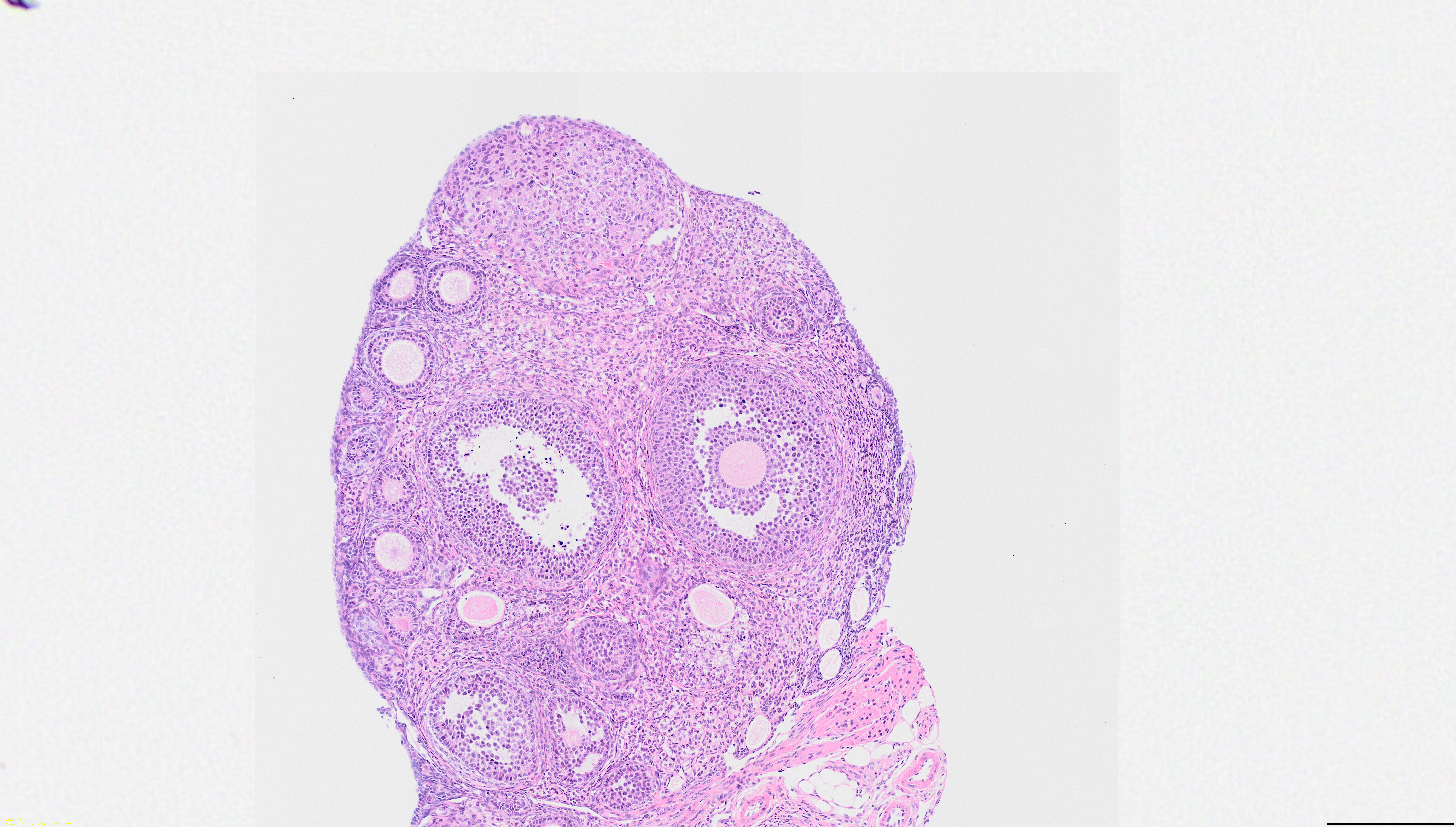

Supplement: Supplementary file 14 — Figure EV3 Source Data [file 44318_2026_832_MOESM14_ESM.zip › Expanded View Figure 3L/G608G+5ht ovary.tif]

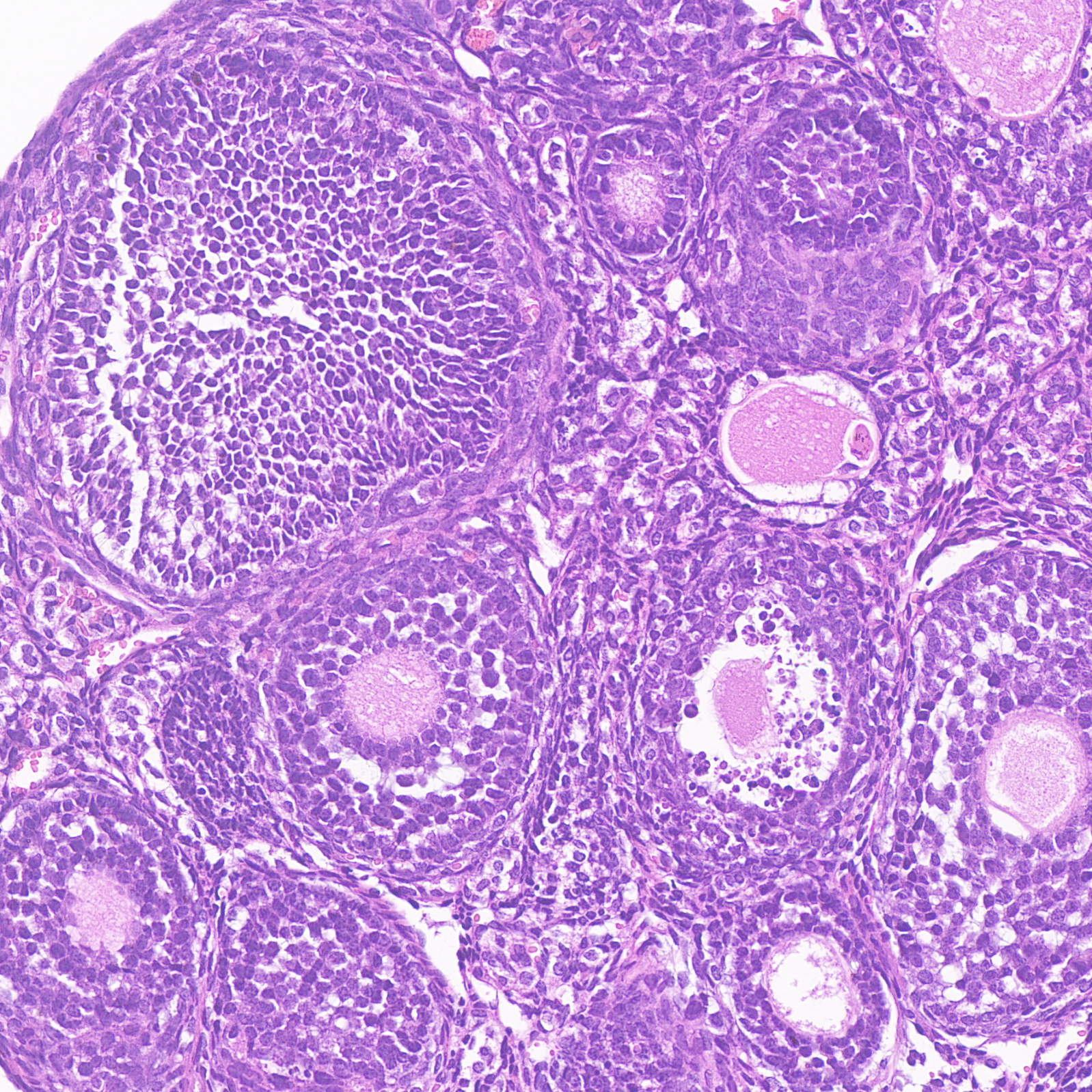

Supplement: Supplementary file 14 — Figure EV3 Source Data [file 44318_2026_832_MOESM14_ESM.zip › Expanded View Figure 3L/WT ovary-large.tif]

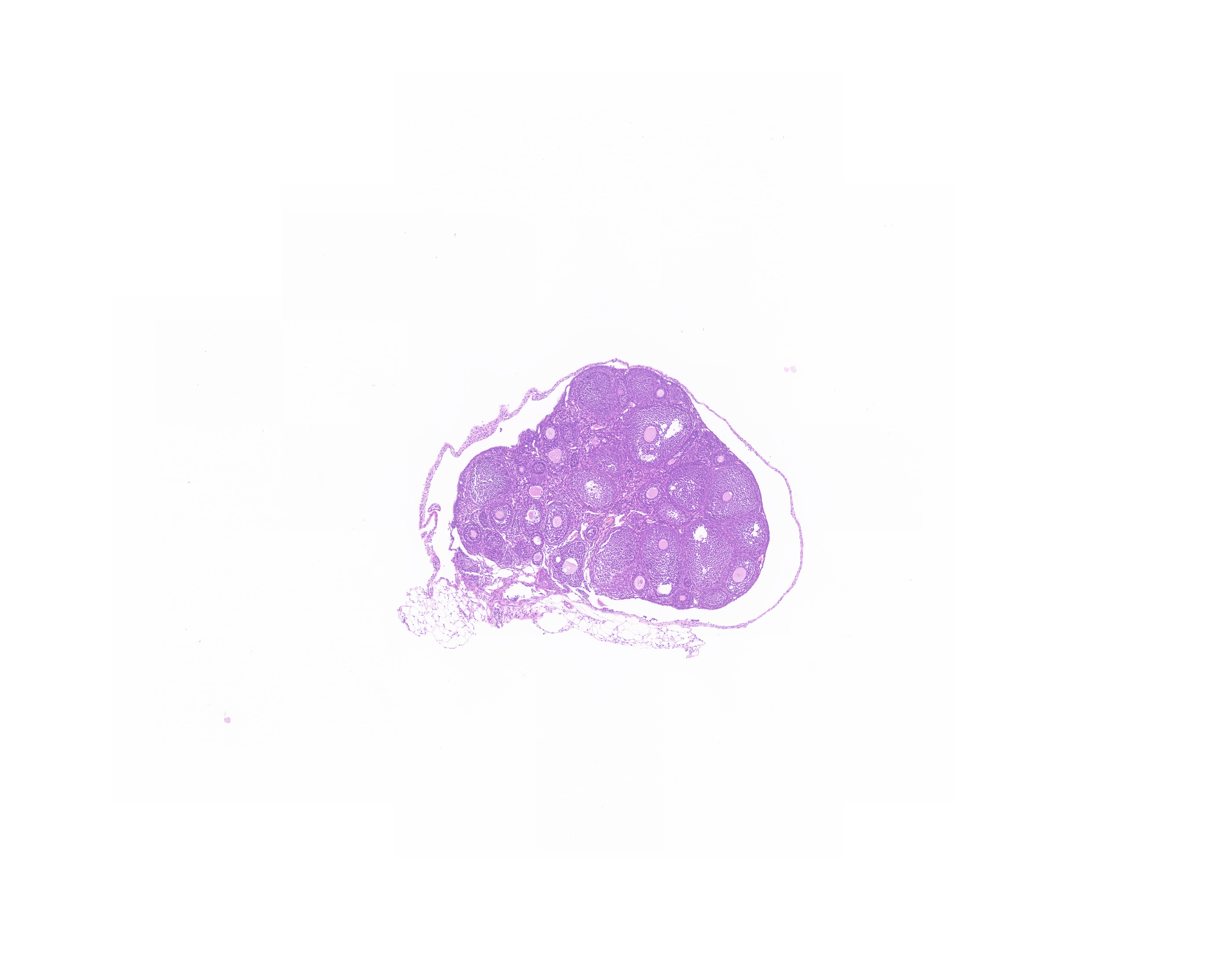

Supplement: Supplementary file 14 — Figure EV3 Source Data [file 44318_2026_832_MOESM14_ESM.zip › Expanded View Figure 3L/WT1卵 3 HE 徐雨雁 1620_5.0x.tif]

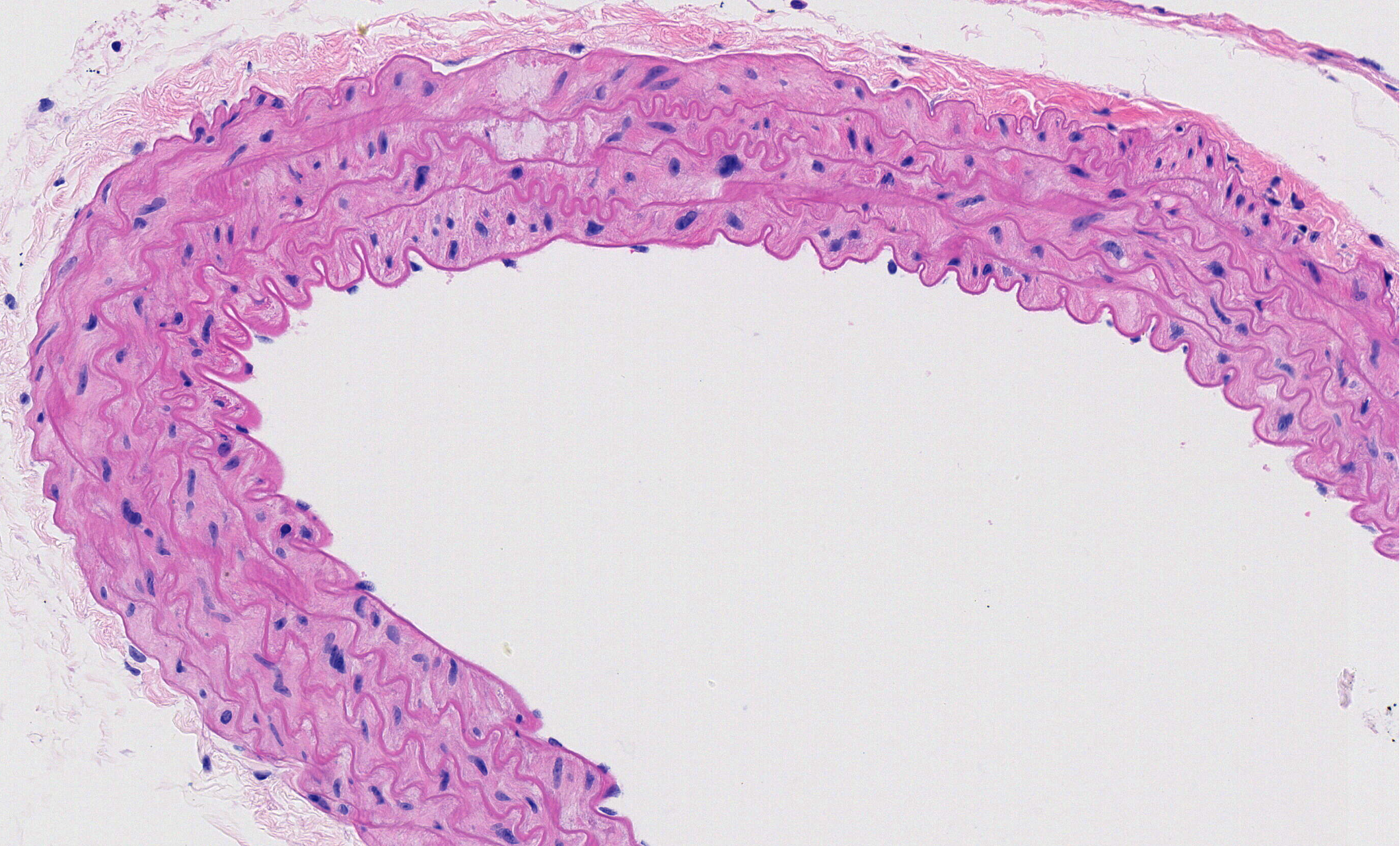

Supplement: Supplementary file 14 — Figure EV3 Source Data [file 44318_2026_832_MOESM14_ESM.zip › Expanded View Figure 3N/20M aorta.png]

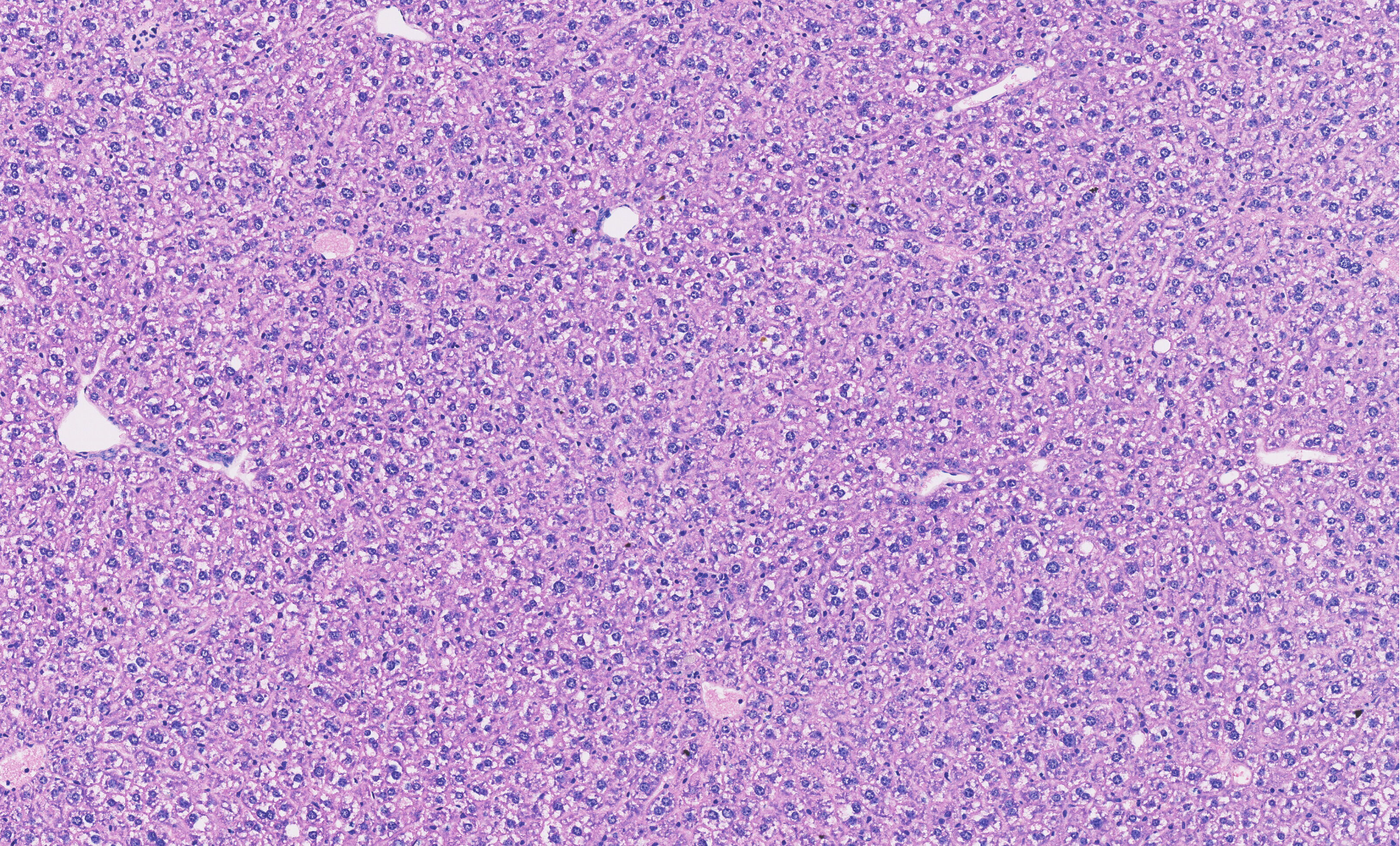

Supplement: Supplementary file 14 — Figure EV3 Source Data [file 44318_2026_832_MOESM14_ESM.zip › Expanded View Figure 3N/20M liver.png]

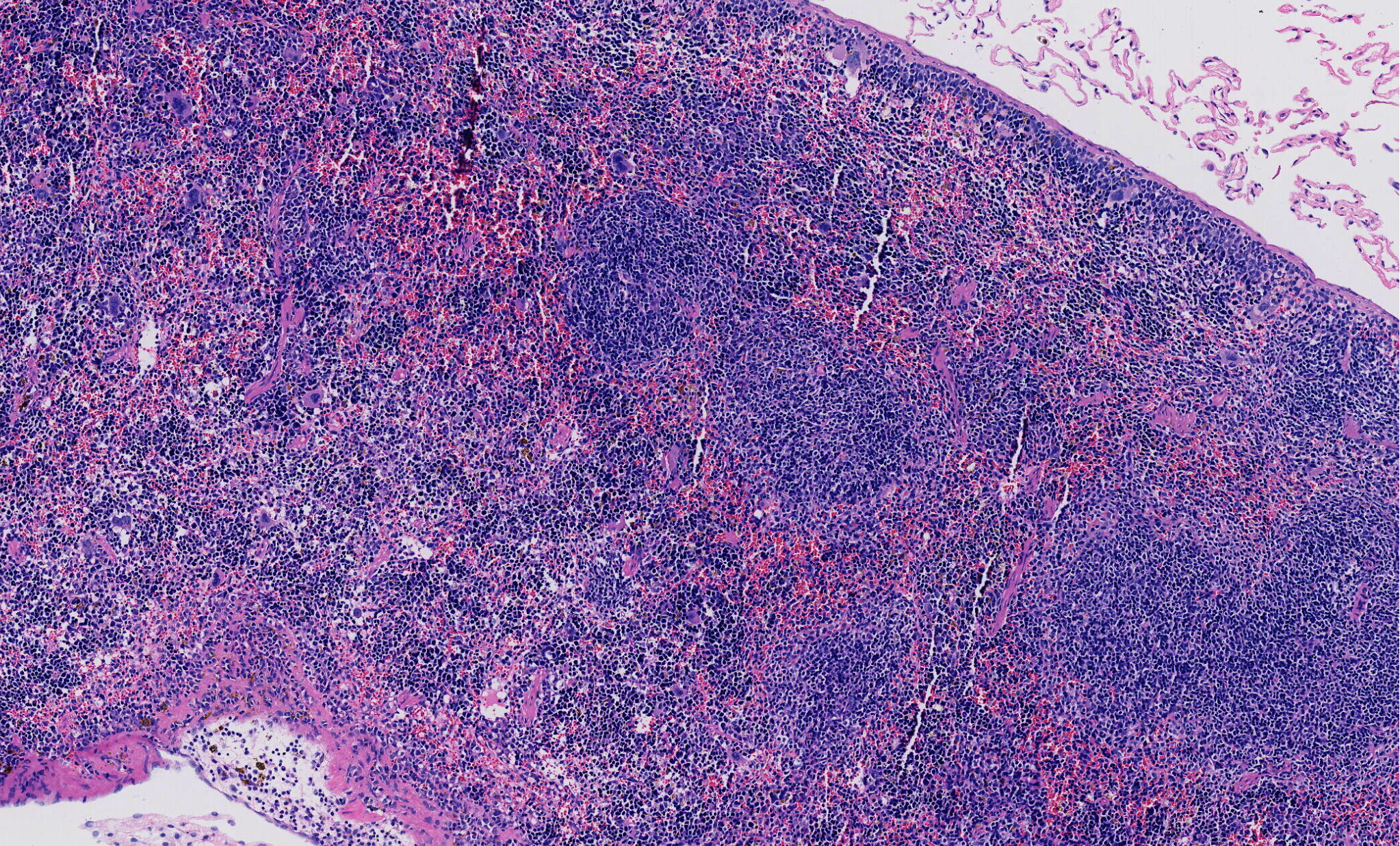

Supplement: Supplementary file 14 — Figure EV3 Source Data [file 44318_2026_832_MOESM14_ESM.zip › Expanded View Figure 3N/20M spleen.png]

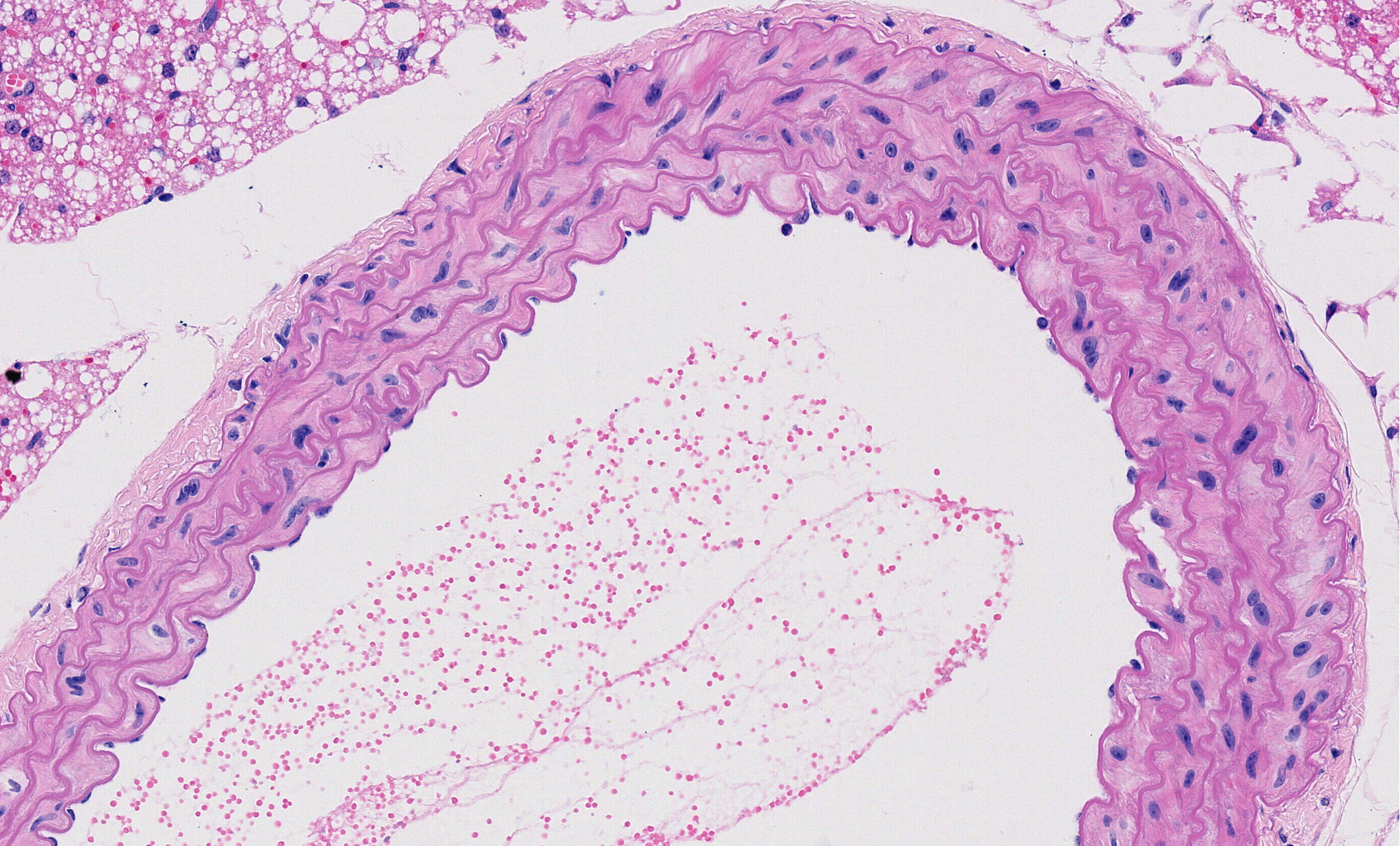

Supplement: Supplementary file 14 — Figure EV3 Source Data [file 44318_2026_832_MOESM14_ESM.zip › Expanded View Figure 3N/20M+5ht aorta.png]

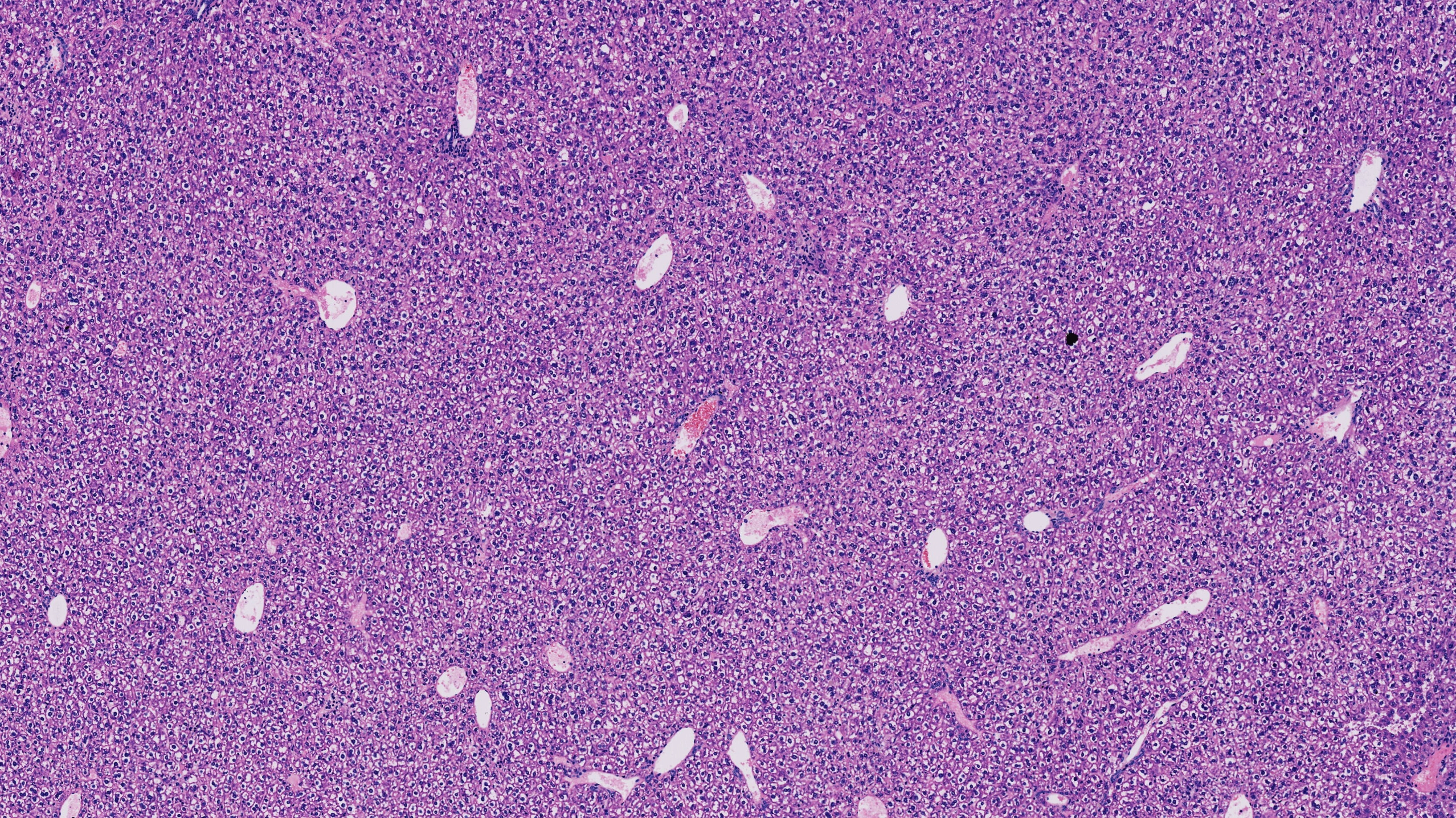

Supplement: Supplementary file 14 — Figure EV3 Source Data [file 44318_2026_832_MOESM14_ESM.zip › Expanded View Figure 3N/20M+5ht liver.png]

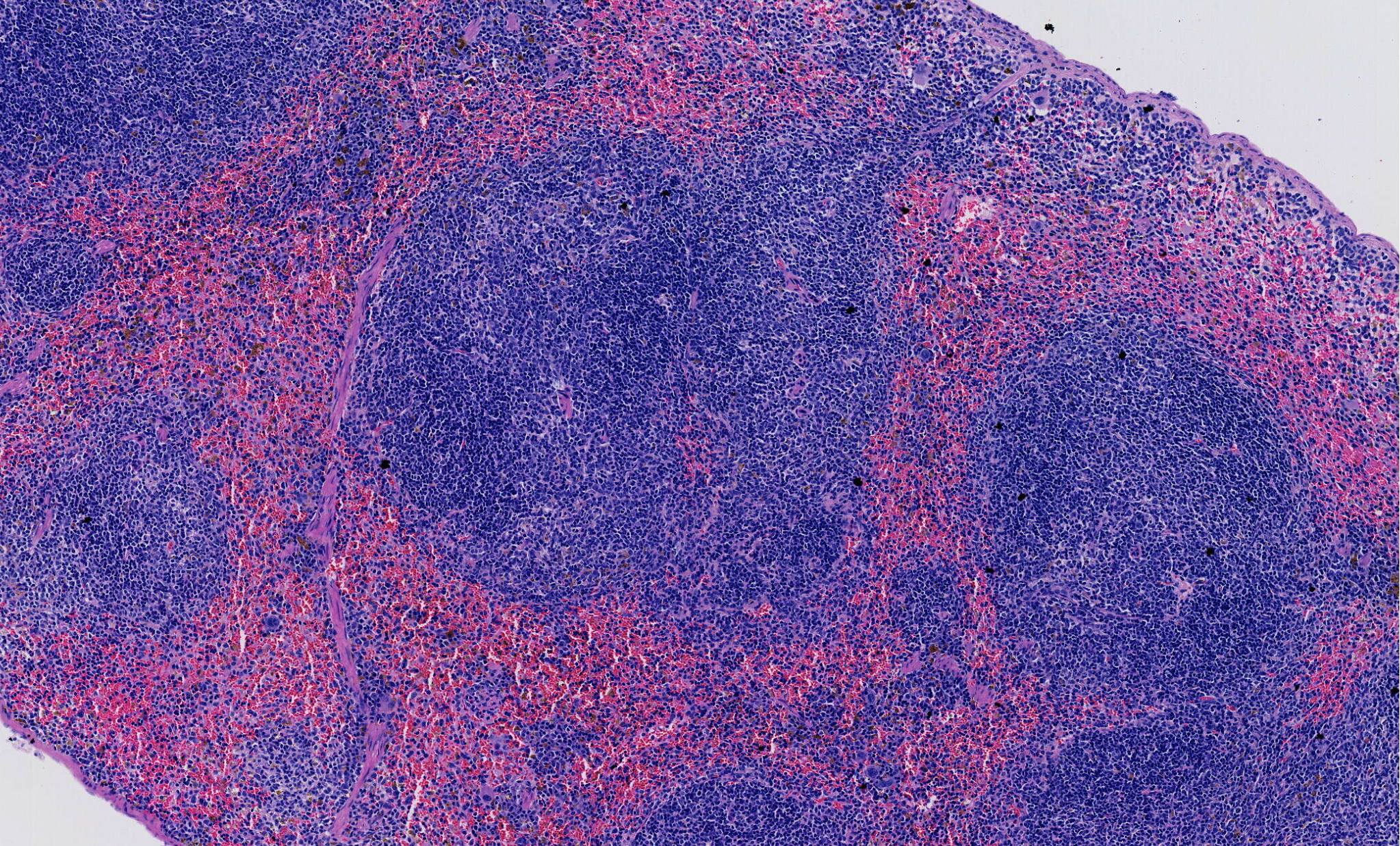

Supplement: Supplementary file 14 — Figure EV3 Source Data [file 44318_2026_832_MOESM14_ESM.zip › Expanded View Figure 3N/20M+5ht pleen.png]

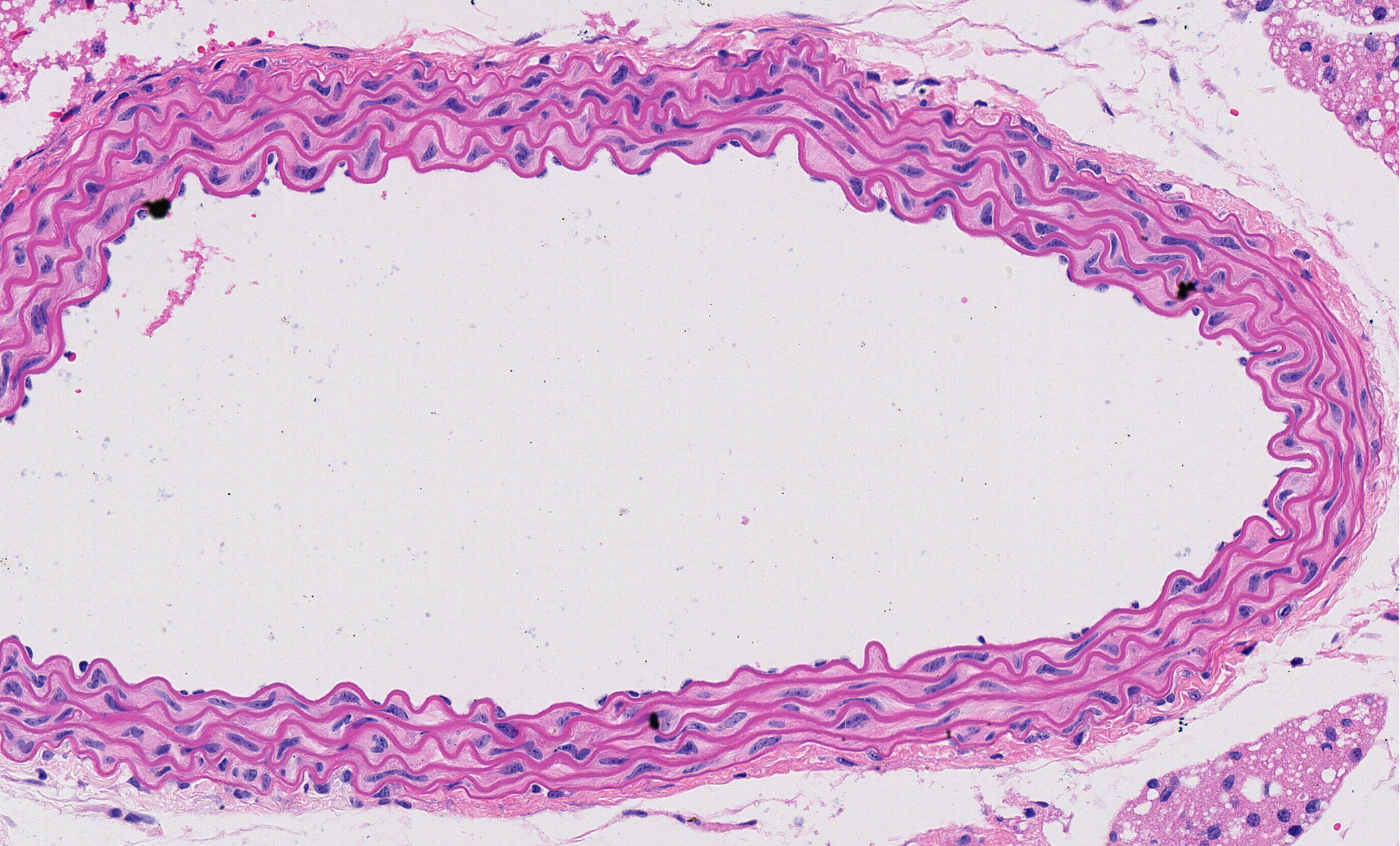

Supplement: Supplementary file 14 — Figure EV3 Source Data [file 44318_2026_832_MOESM14_ESM.zip › Expanded View Figure 3N/4w aorta.png]

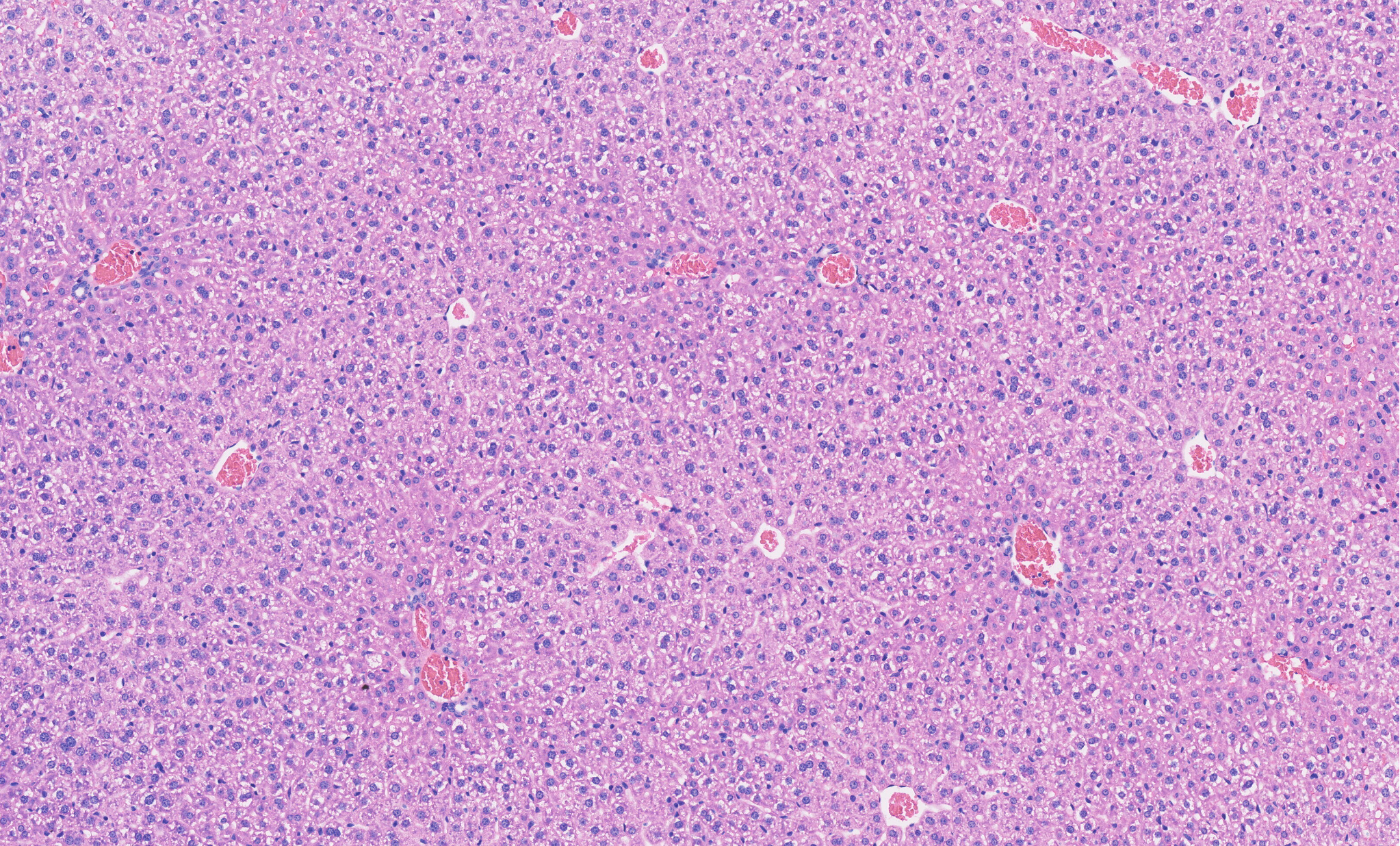

Supplement: Supplementary file 14 — Figure EV3 Source Data [file 44318_2026_832_MOESM14_ESM.zip › Expanded View Figure 3N/4w liver.png]

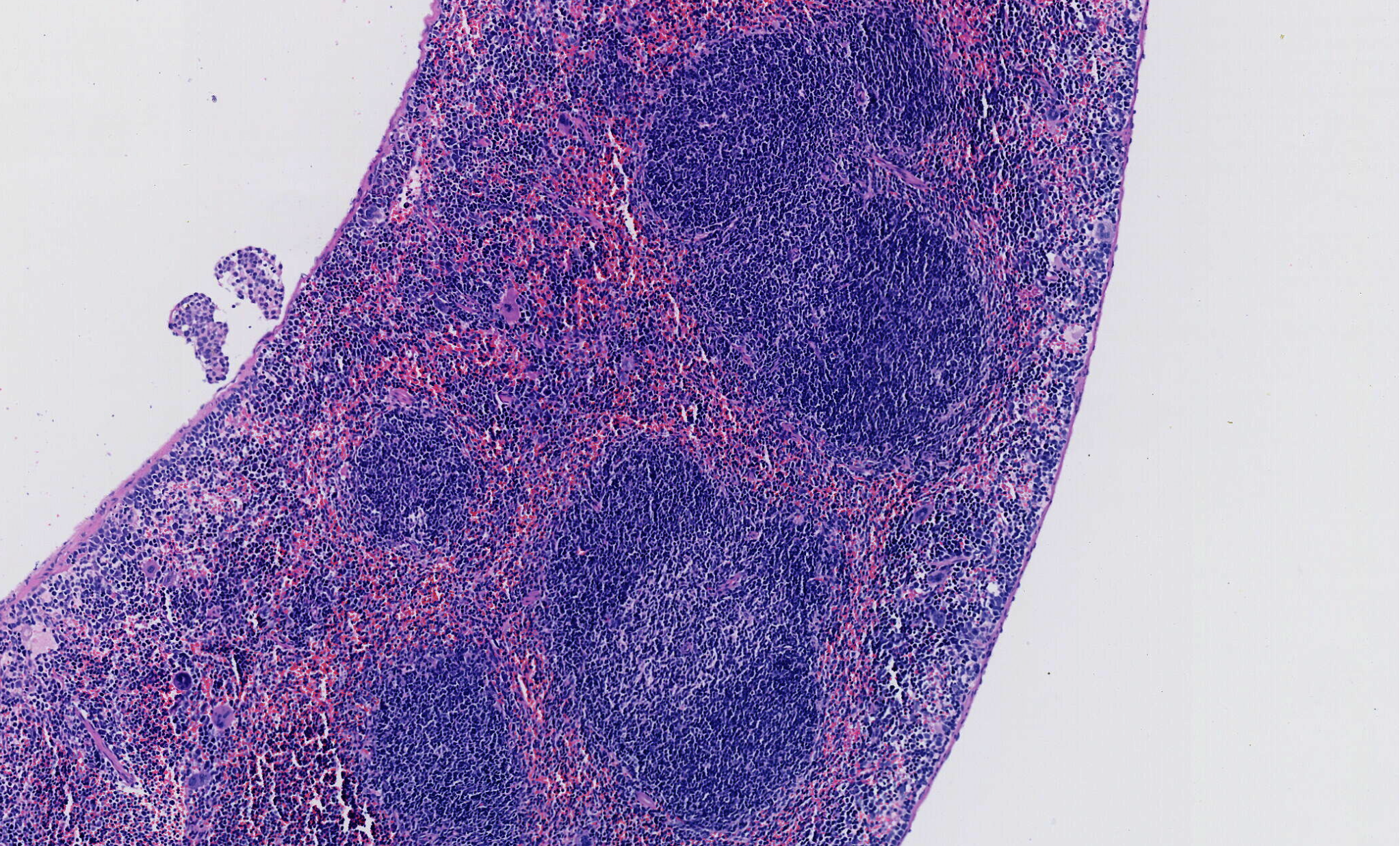

Supplement: Supplementary file 14 — Figure EV3 Source Data [file 44318_2026_832_MOESM14_ESM.zip › Expanded View Figure 3N/4w spleen.png]

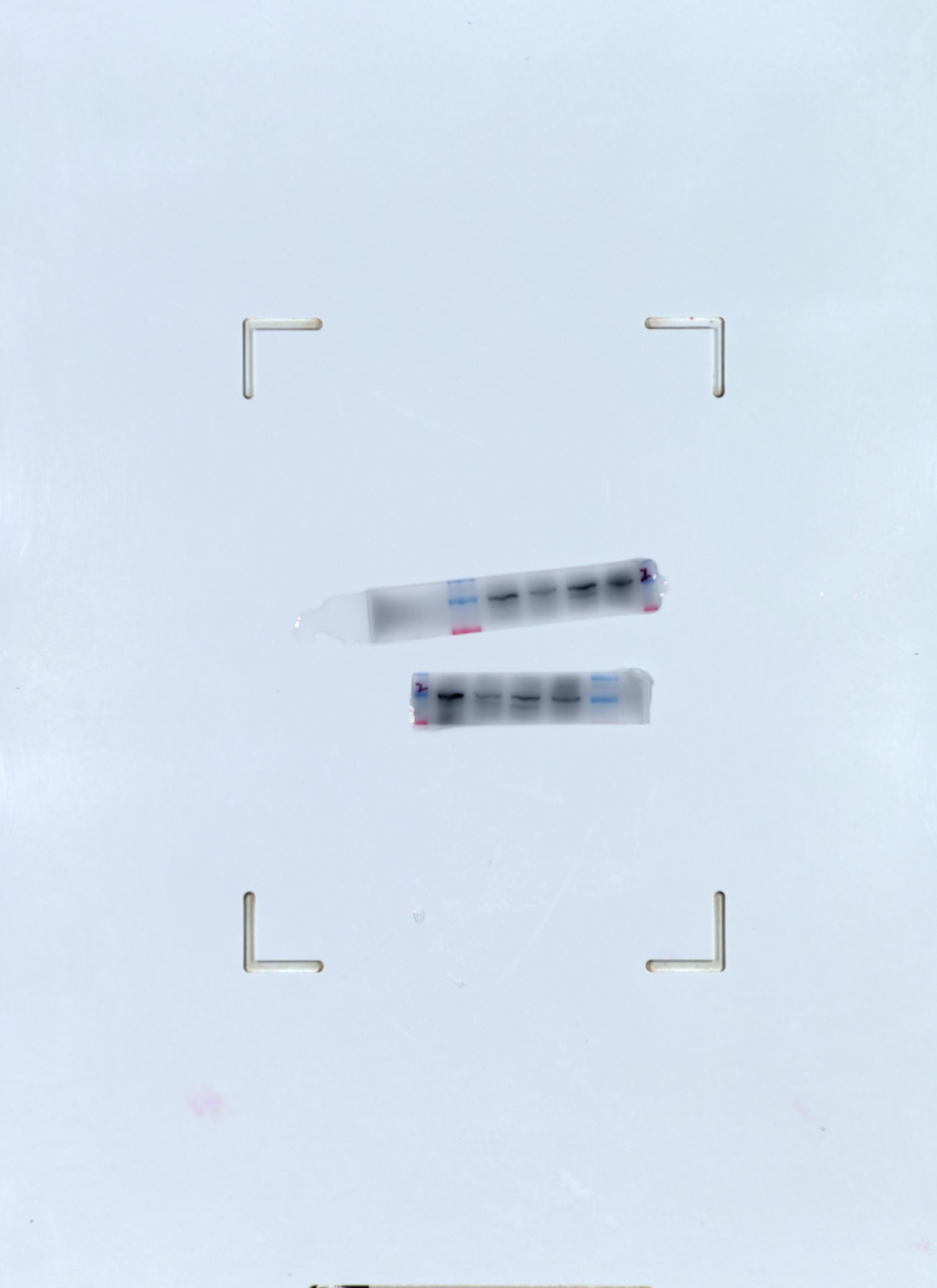

Supplement: Supplementary file 16 — Figure EV5 Source Data [file 44318_2026_832_MOESM16_ESM.zip › H/pgc1α+marker.jpg]

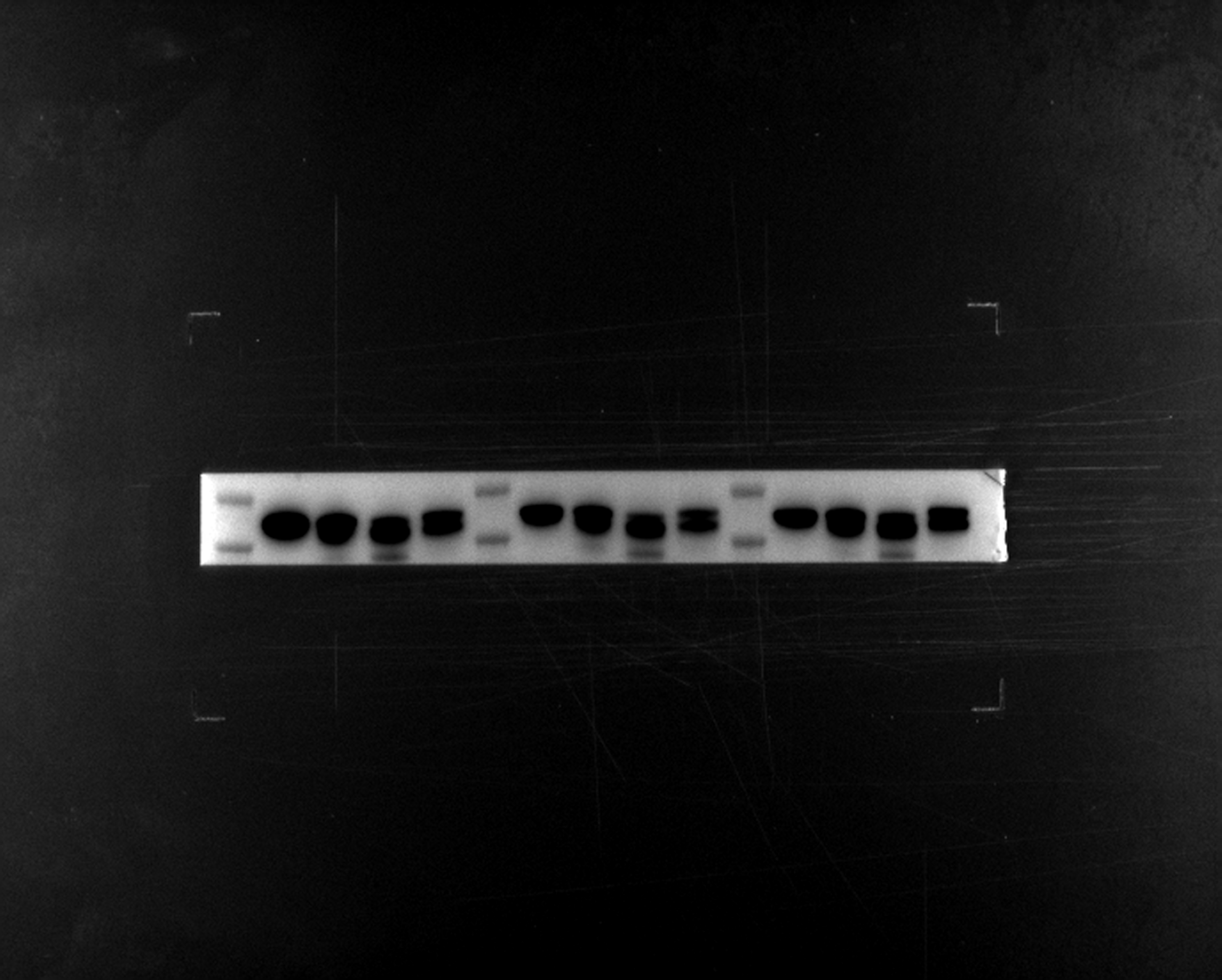

Supplement: Supplementary file 16 — Figure EV5 Source Data [file 44318_2026_832_MOESM16_ESM.zip › H/replicates/R-1-ACTIN-3-2.tif]

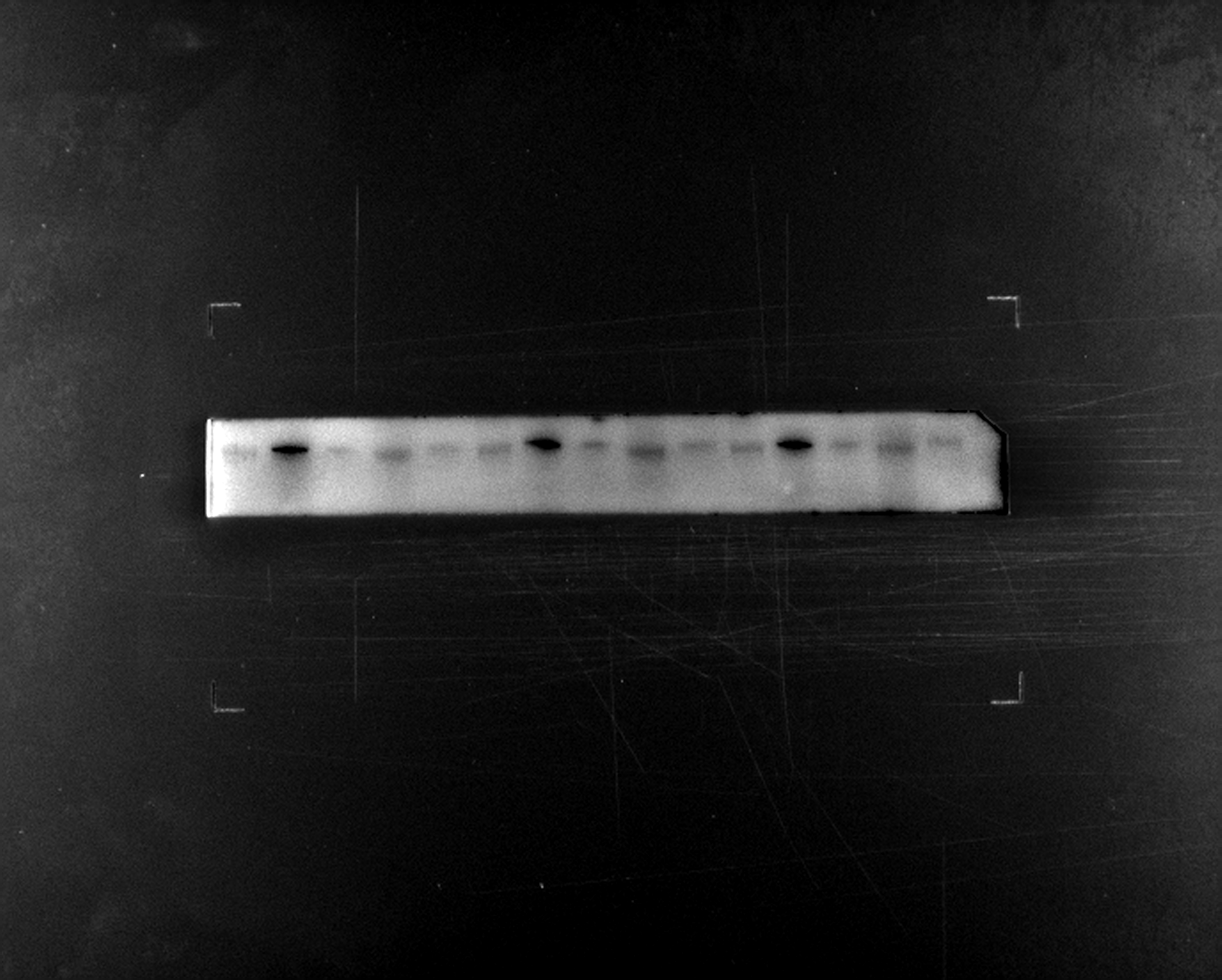

Supplement: Supplementary file 16 — Figure EV5 Source Data [file 44318_2026_832_MOESM16_ESM.zip › H/replicates/R-1-PGC1A-3.tif]

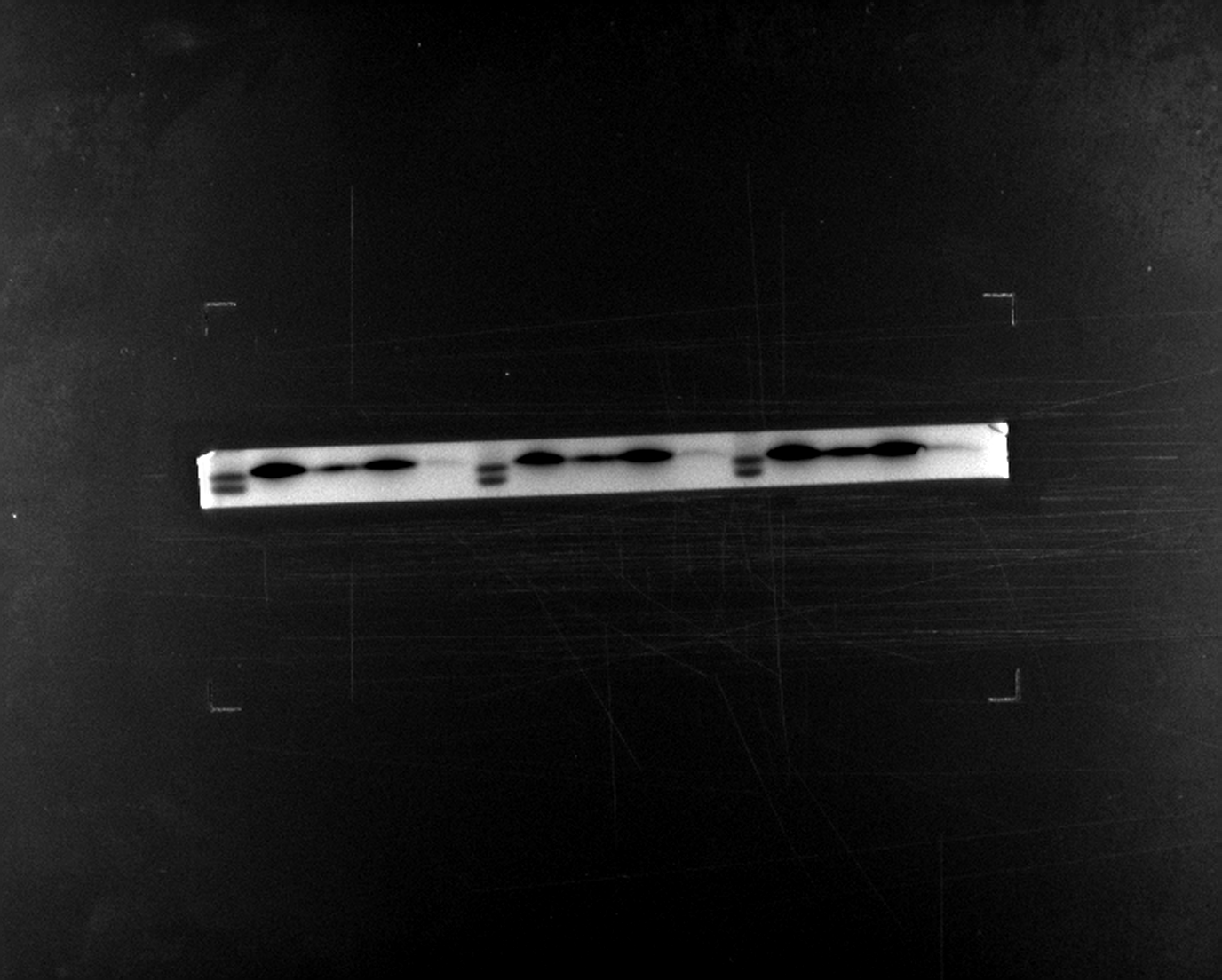

Supplement: Supplementary file 16 — Figure EV5 Source Data [file 44318_2026_832_MOESM16_ESM.zip › H/replicates/R-1-TOMM20-3.tif]

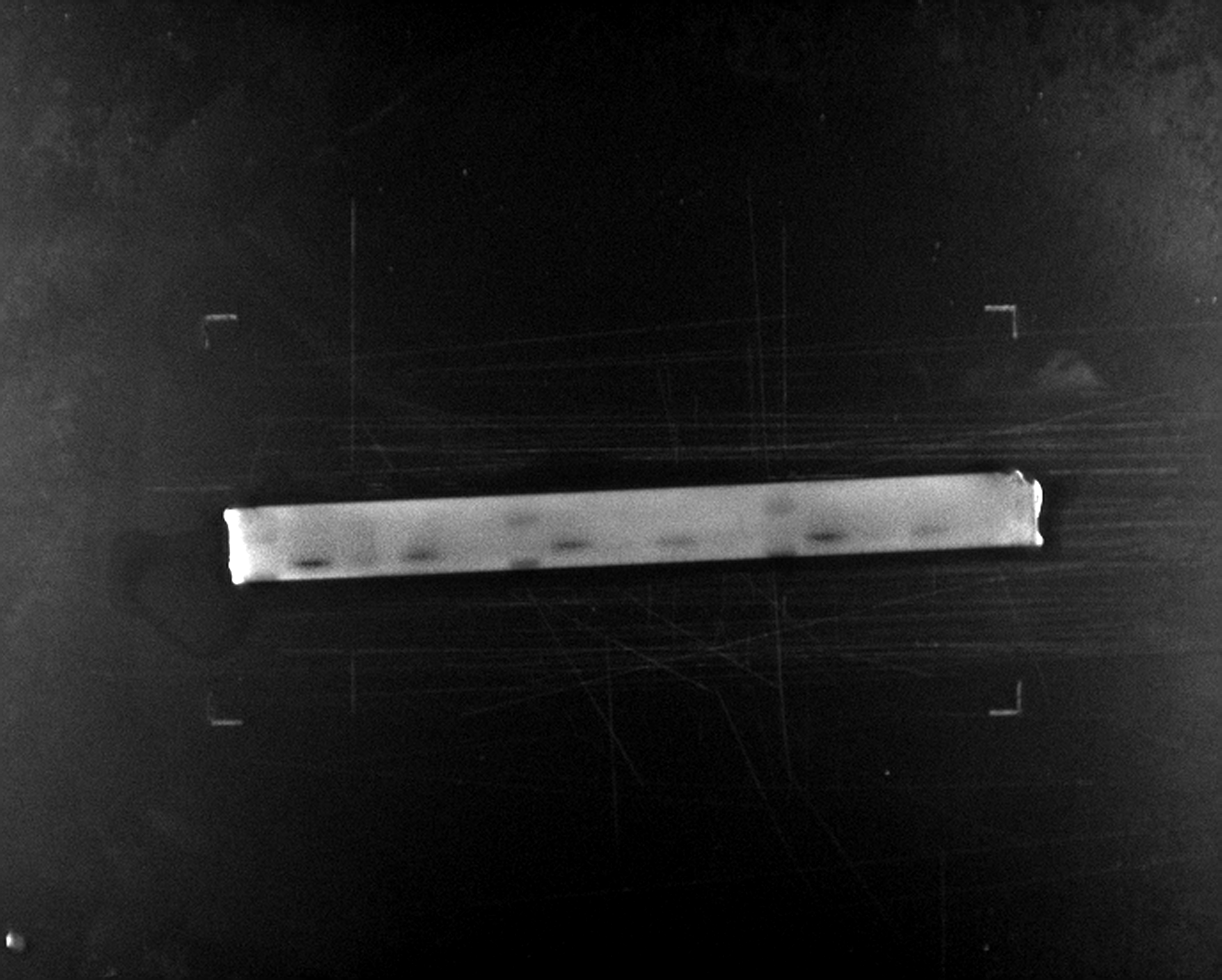

Supplement: Supplementary file 16 — Figure EV5 Source Data [file 44318_2026_832_MOESM16_ESM.zip › H/replicates/RR-TIMM23-6-3.tif]

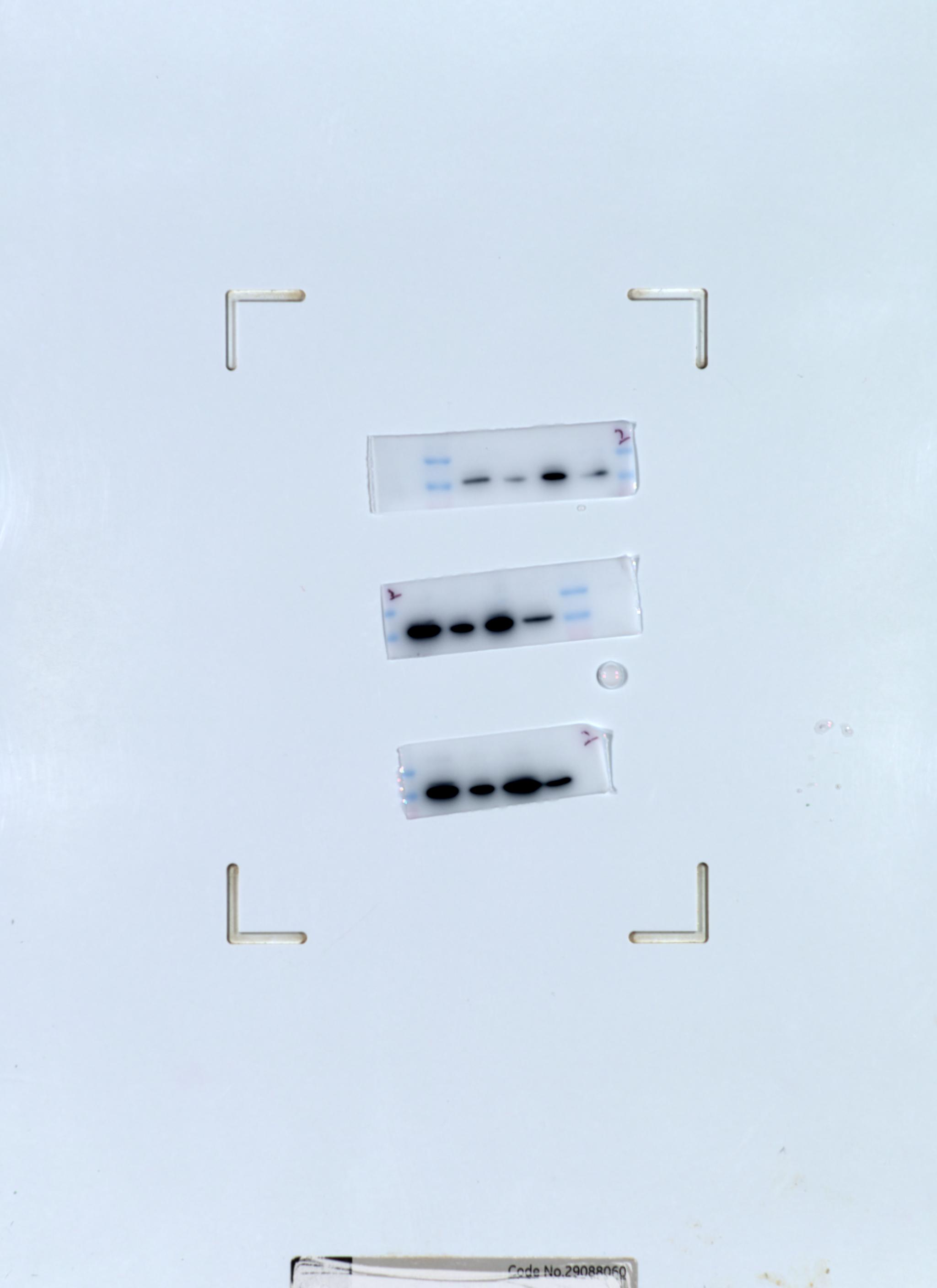

Supplement: Supplementary file 16 — Figure EV5 Source Data [file 44318_2026_832_MOESM16_ESM.zip › H/TIMM23+marker.jpg]

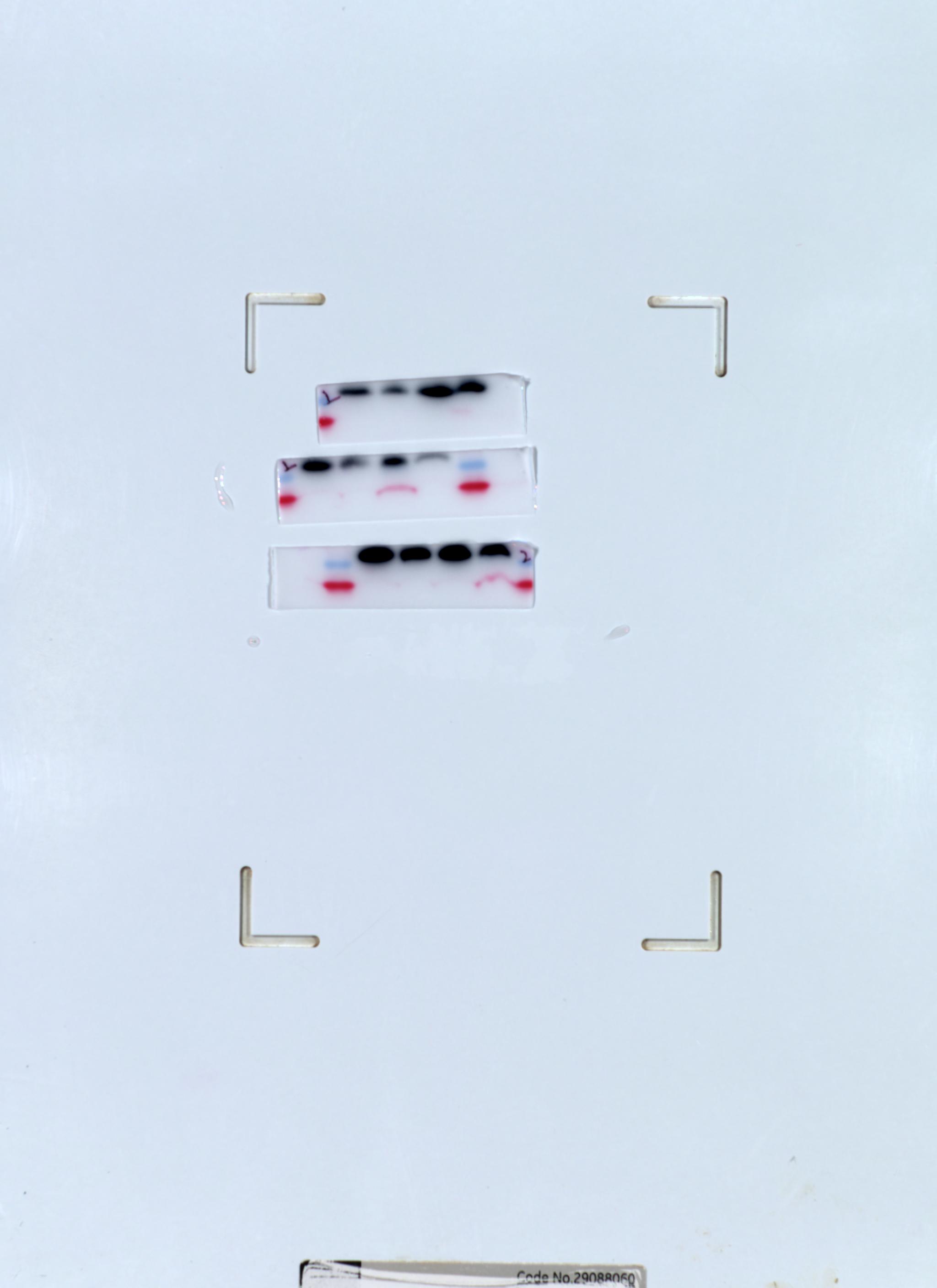

Supplement: Supplementary file 16 — Figure EV5 Source Data [file 44318_2026_832_MOESM16_ESM.zip › H/TOMM20+marker.jpg]

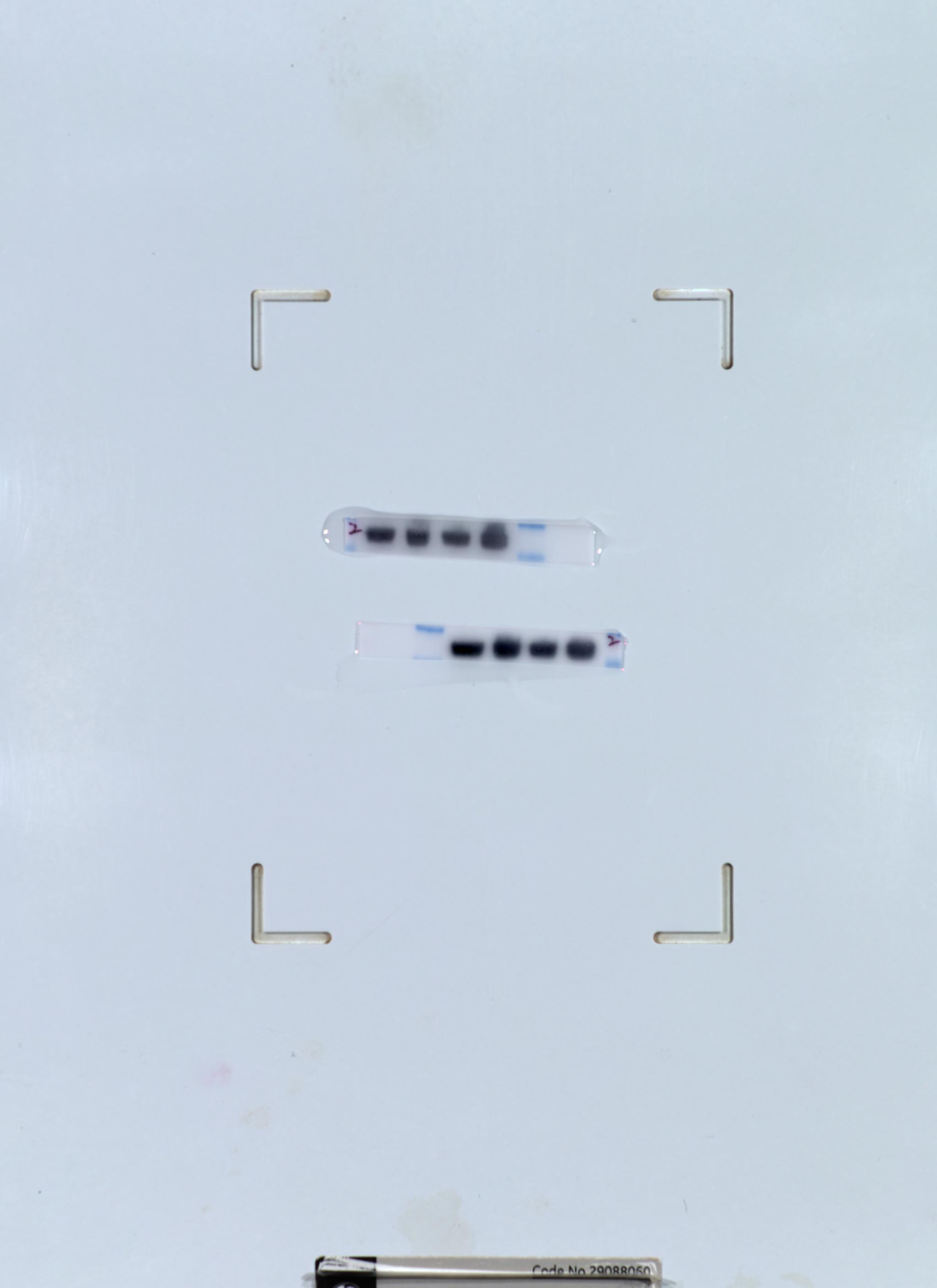

Supplement: Supplementary file 16 — Figure EV5 Source Data [file 44318_2026_832_MOESM16_ESM.zip › H/β-actin+marker.jpg]

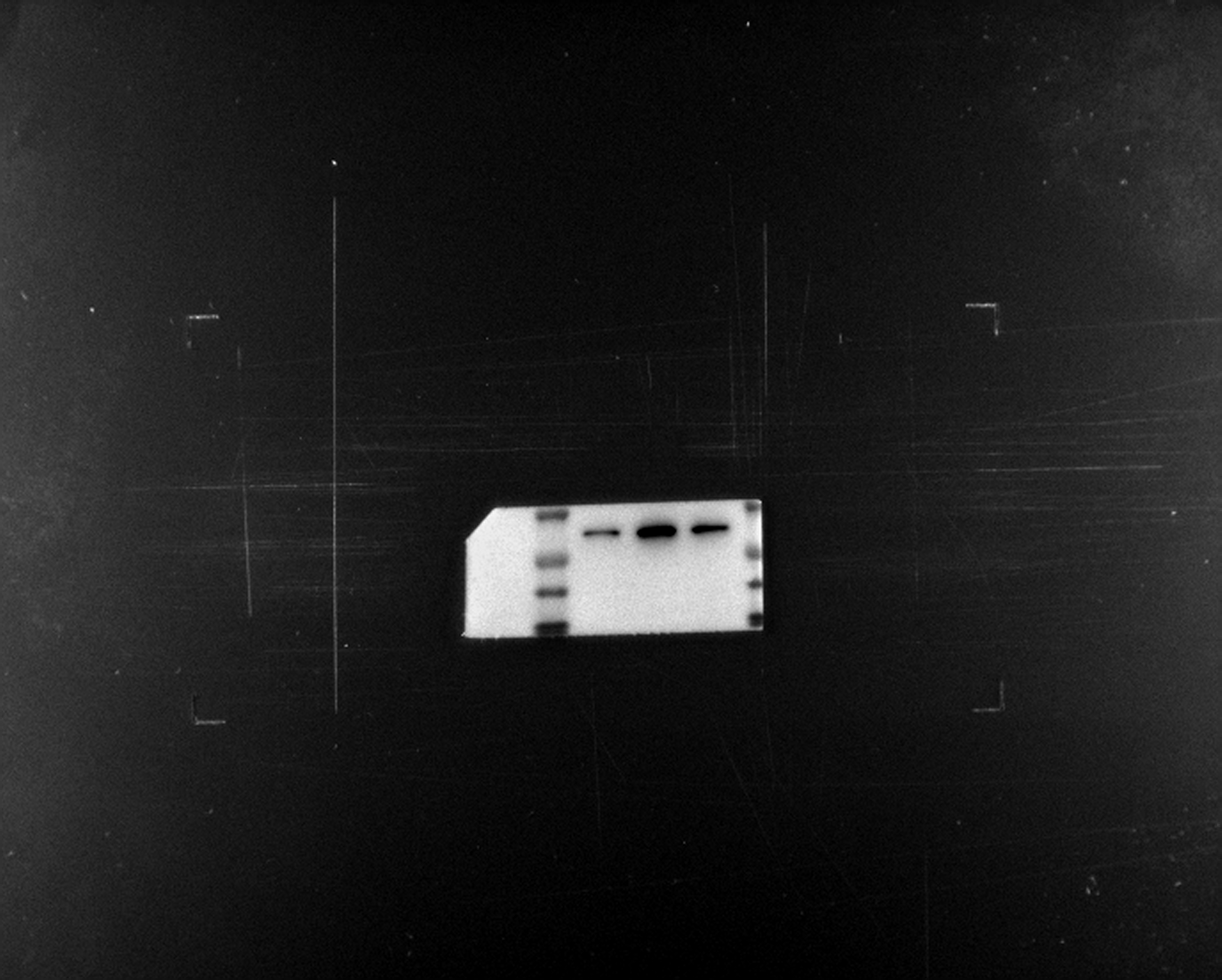

Supplement: Supplementary file 19 — Figure EV8 Source Data [file 44318_2026_832_MOESM19_ESM.zip › Figure EV8/C/G608G CHOP.tif]

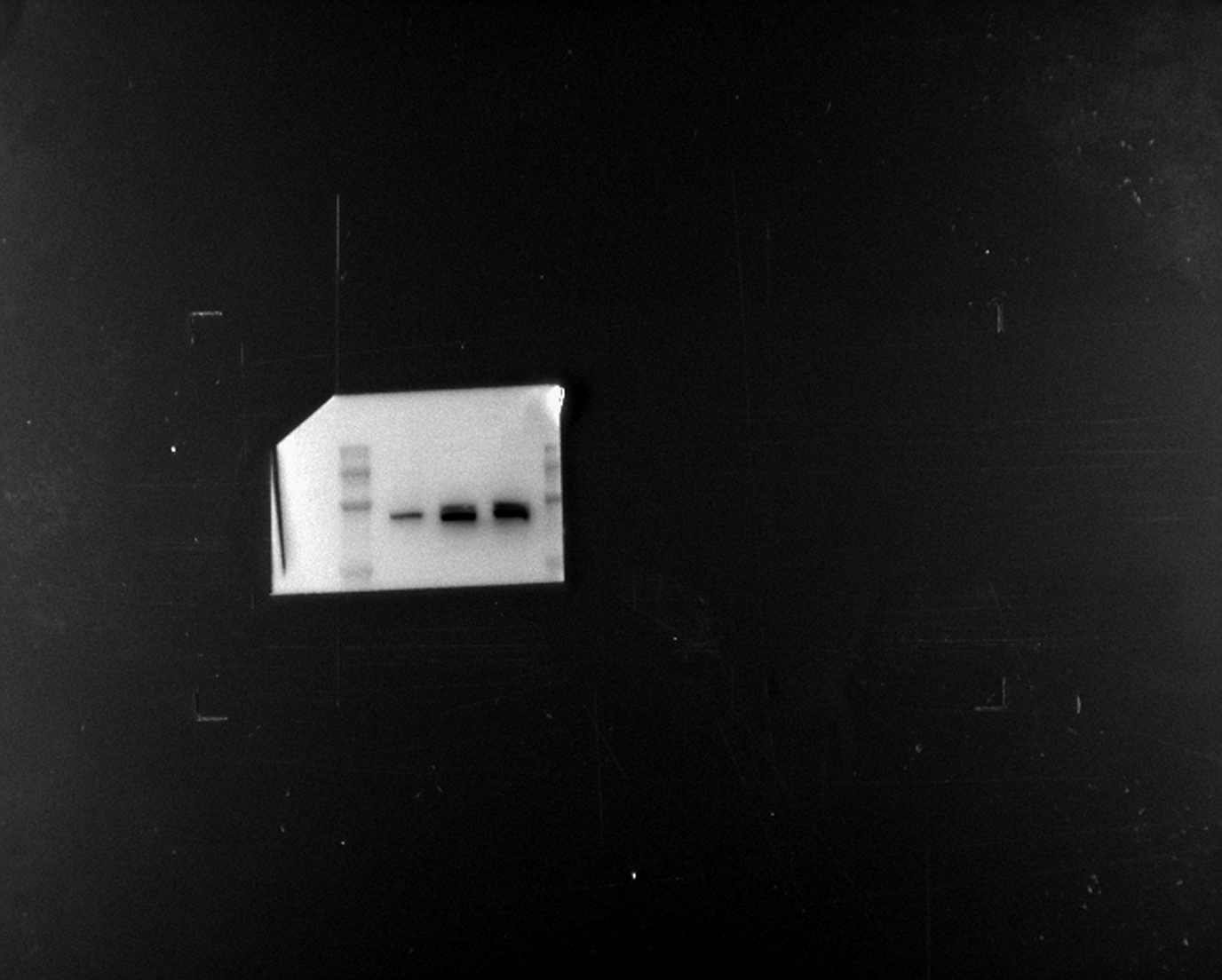

Supplement: Supplementary file 19 — Figure EV8 Source Data [file 44318_2026_832_MOESM19_ESM.zip › Figure EV8/C/G608G HSP90β.tif]

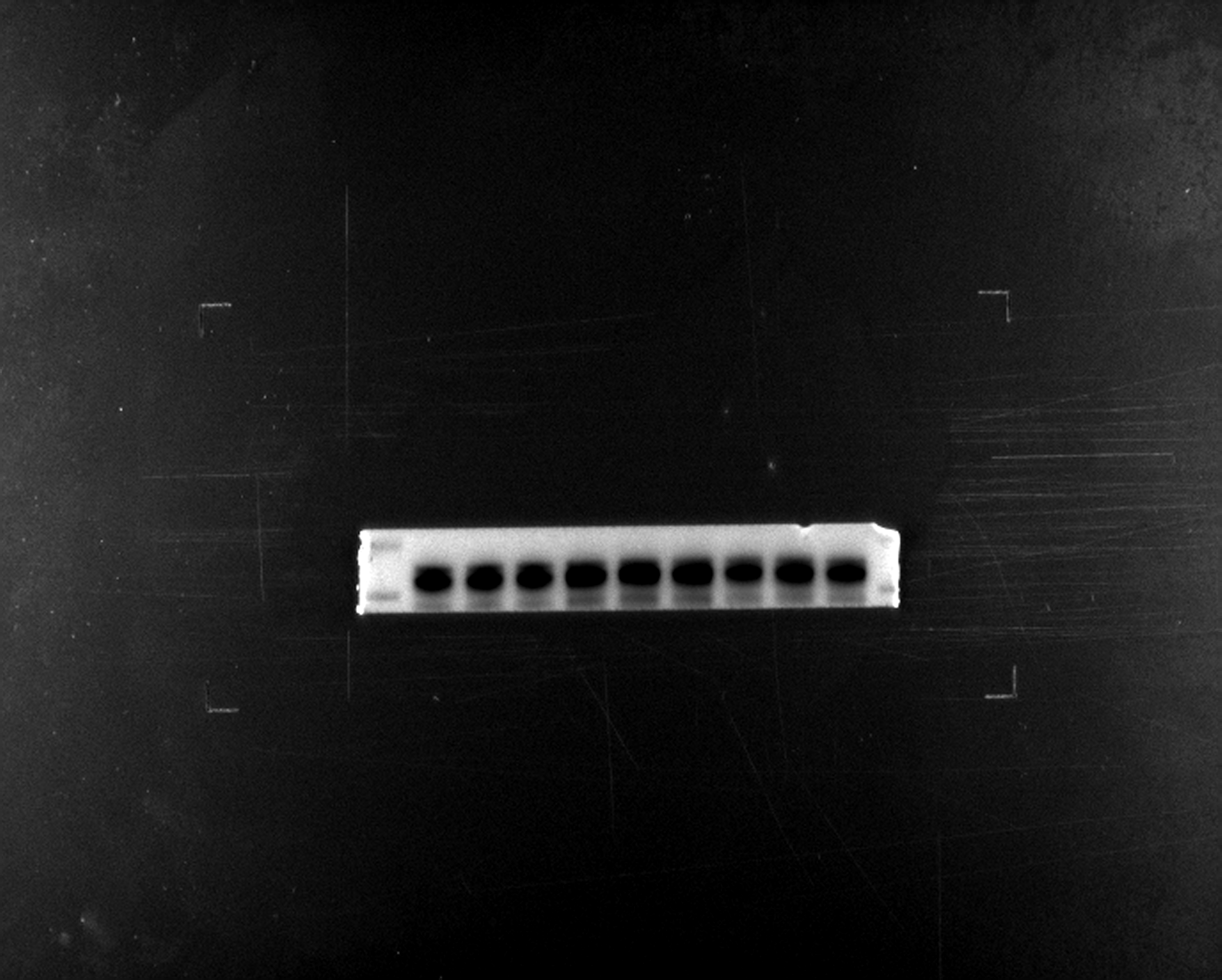

Supplement: Supplementary file 19 — Figure EV8 Source Data [file 44318_2026_832_MOESM19_ESM.zip › Figure EV8/C/replicates/1-1824OE-ACTIN-3.tif]

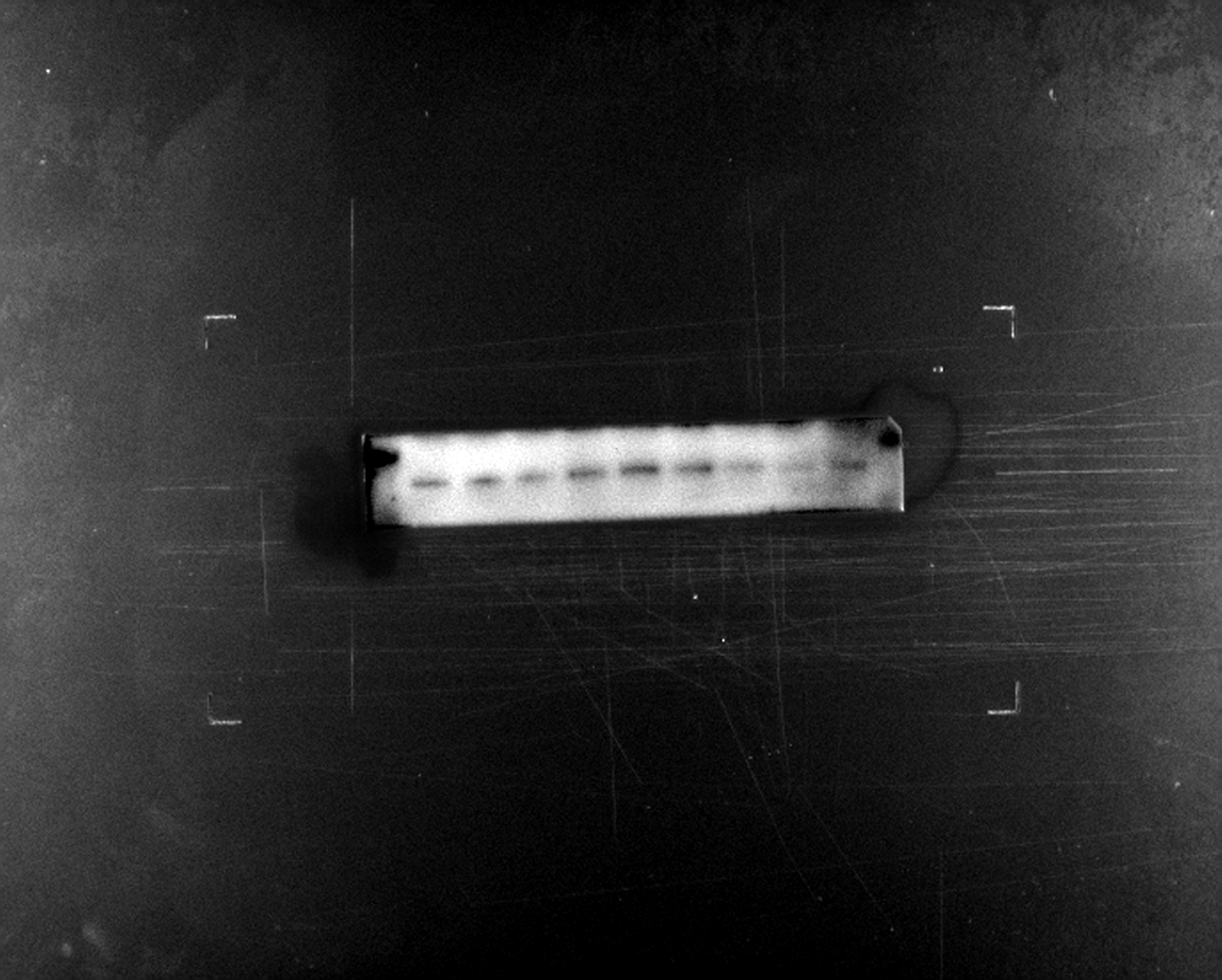

Supplement: Supplementary file 19 — Figure EV8 Source Data [file 44318_2026_832_MOESM19_ESM.zip › Figure EV8/C/replicates/1-1824OE-DDIT3-3.tif]

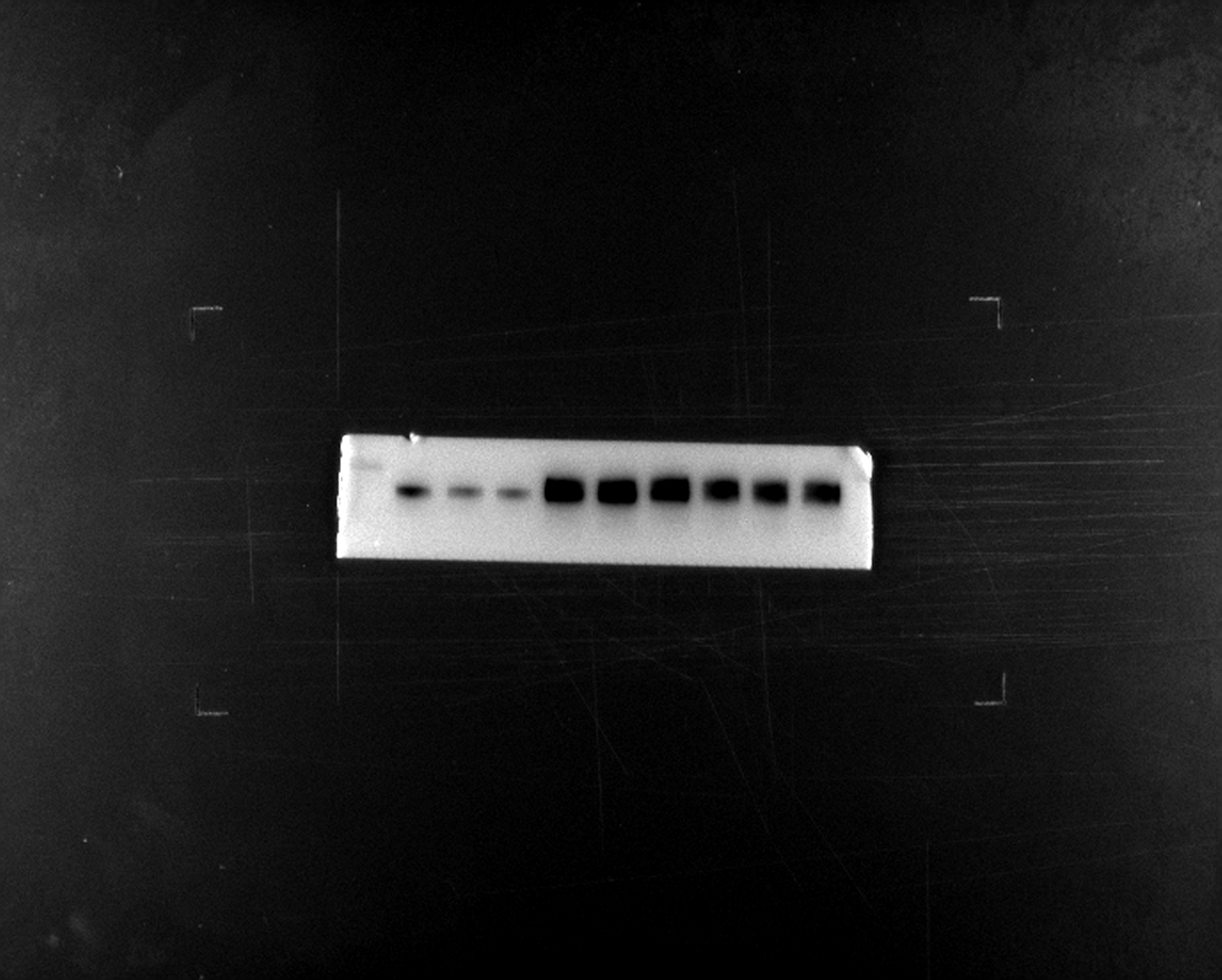

Supplement: Supplementary file 19 — Figure EV8 Source Data [file 44318_2026_832_MOESM19_ESM.zip › Figure EV8/C/replicates/1-1824OE-HSP90-3.tif]

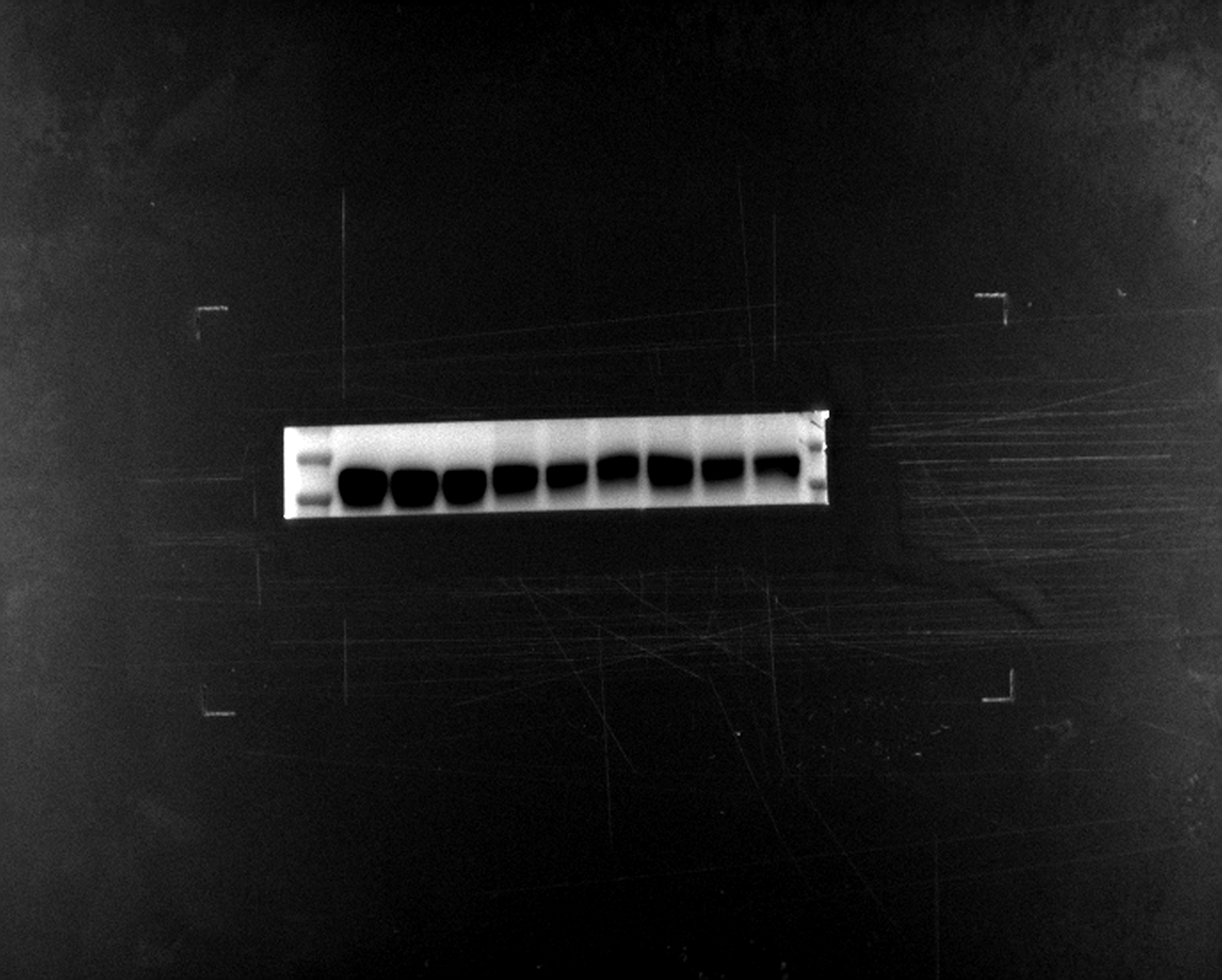

Supplement: Supplementary file 19 — Figure EV8 Source Data [file 44318_2026_832_MOESM19_ESM.zip › Figure EV8/C/replicates/2-WTOE-ACTIN-3.tif]

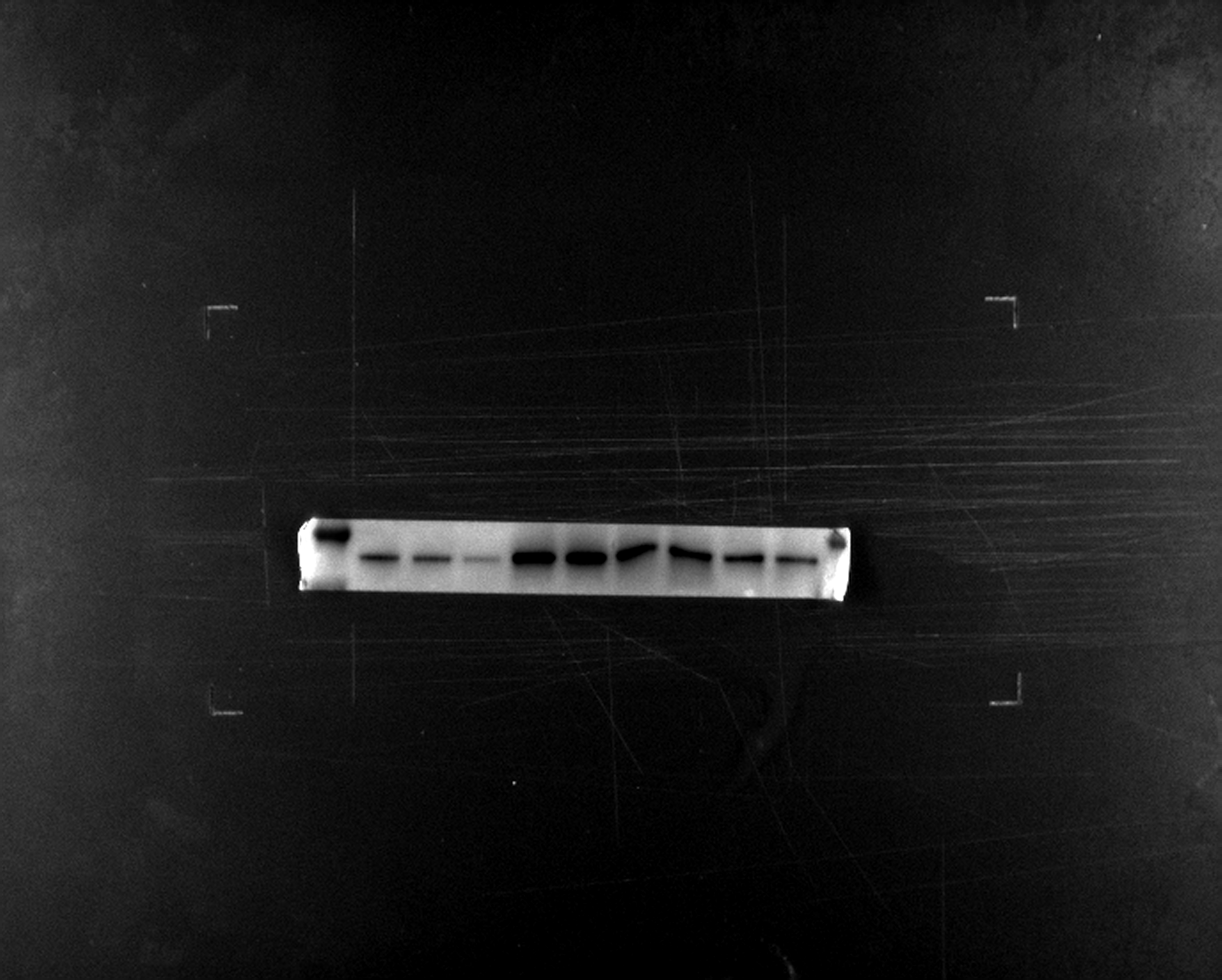

Supplement: Supplementary file 19 — Figure EV8 Source Data [file 44318_2026_832_MOESM19_ESM.zip › Figure EV8/C/replicates/2-WTOE-DDIT3-3.tif]

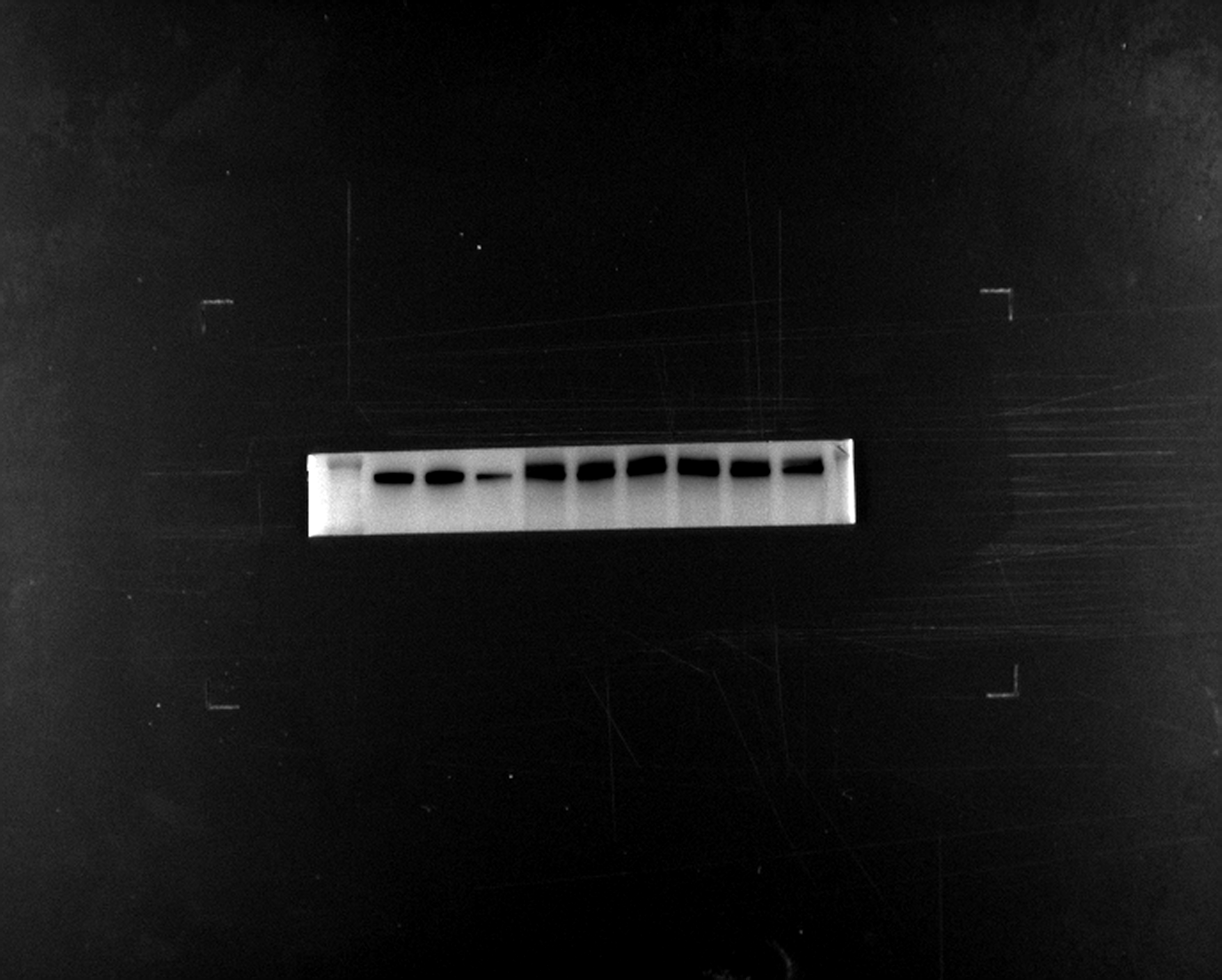

Supplement: Supplementary file 19 — Figure EV8 Source Data [file 44318_2026_832_MOESM19_ESM.zip › Figure EV8/C/replicates/2-WTOE-HSP90-3.tif]

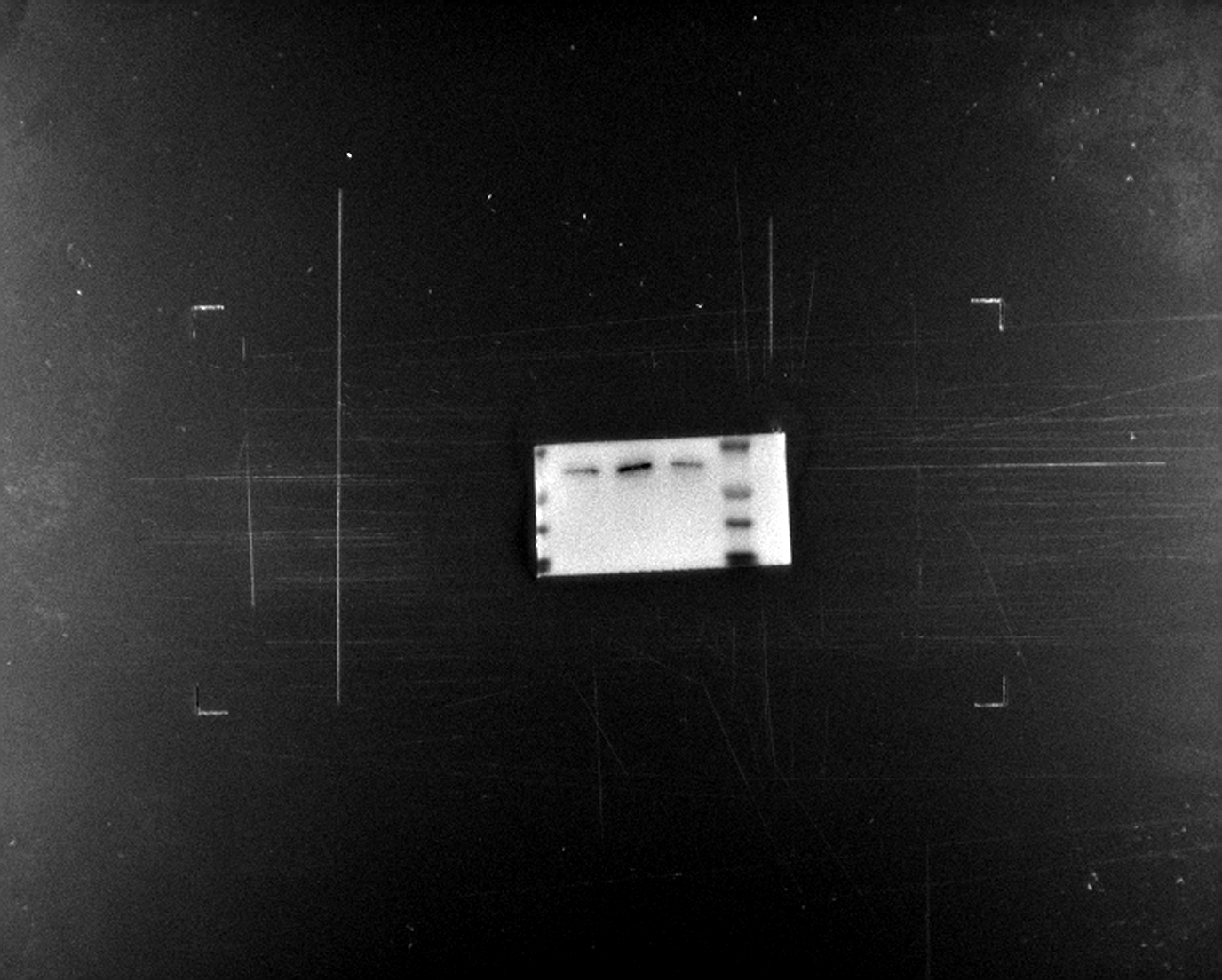

Supplement: Supplementary file 19 — Figure EV8 Source Data [file 44318_2026_832_MOESM19_ESM.zip › Figure EV8/C/WT CHOP.tif]

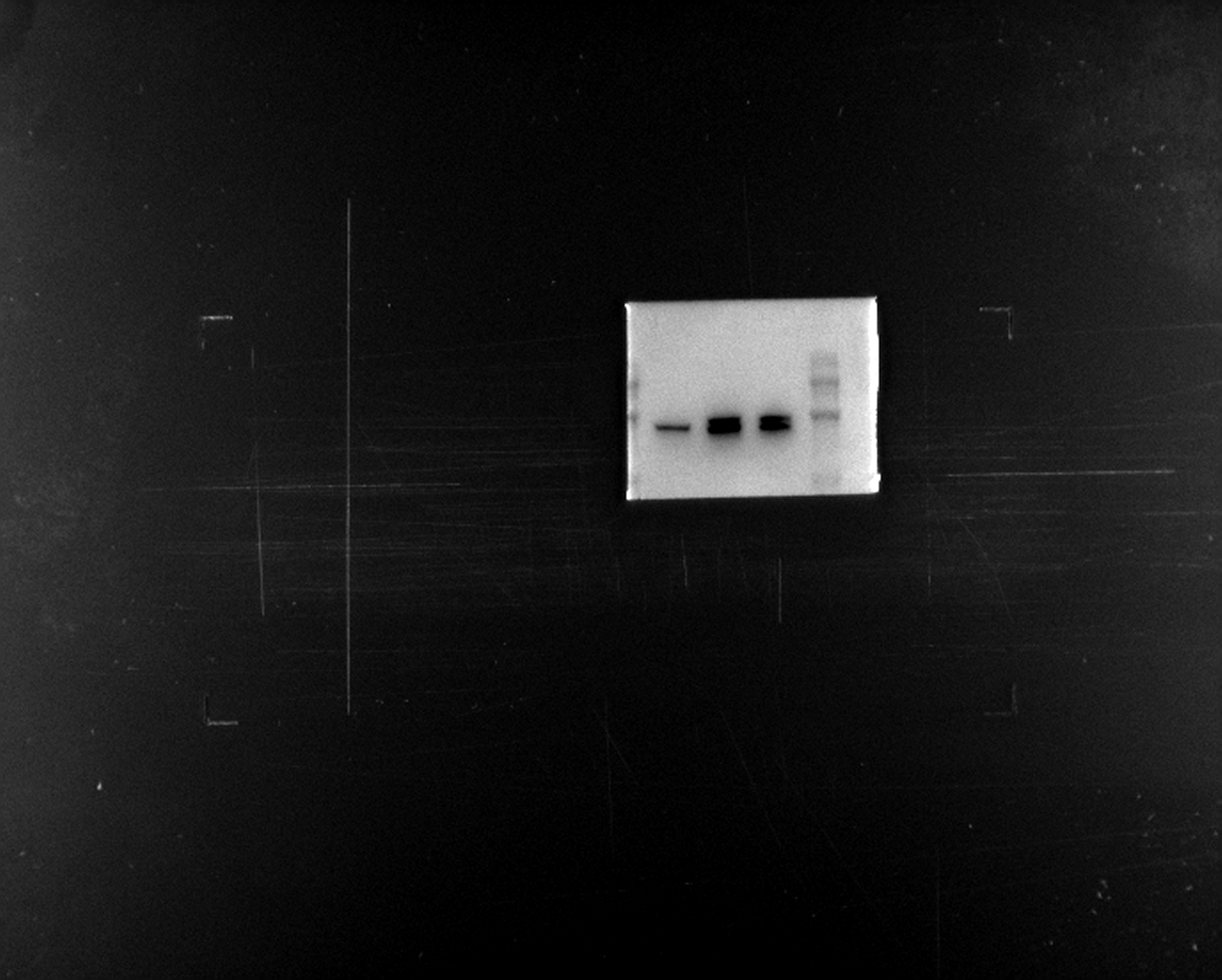

Supplement: Supplementary file 19 — Figure EV8 Source Data [file 44318_2026_832_MOESM19_ESM.zip › Figure EV8/C/WT HSP90β.tif]

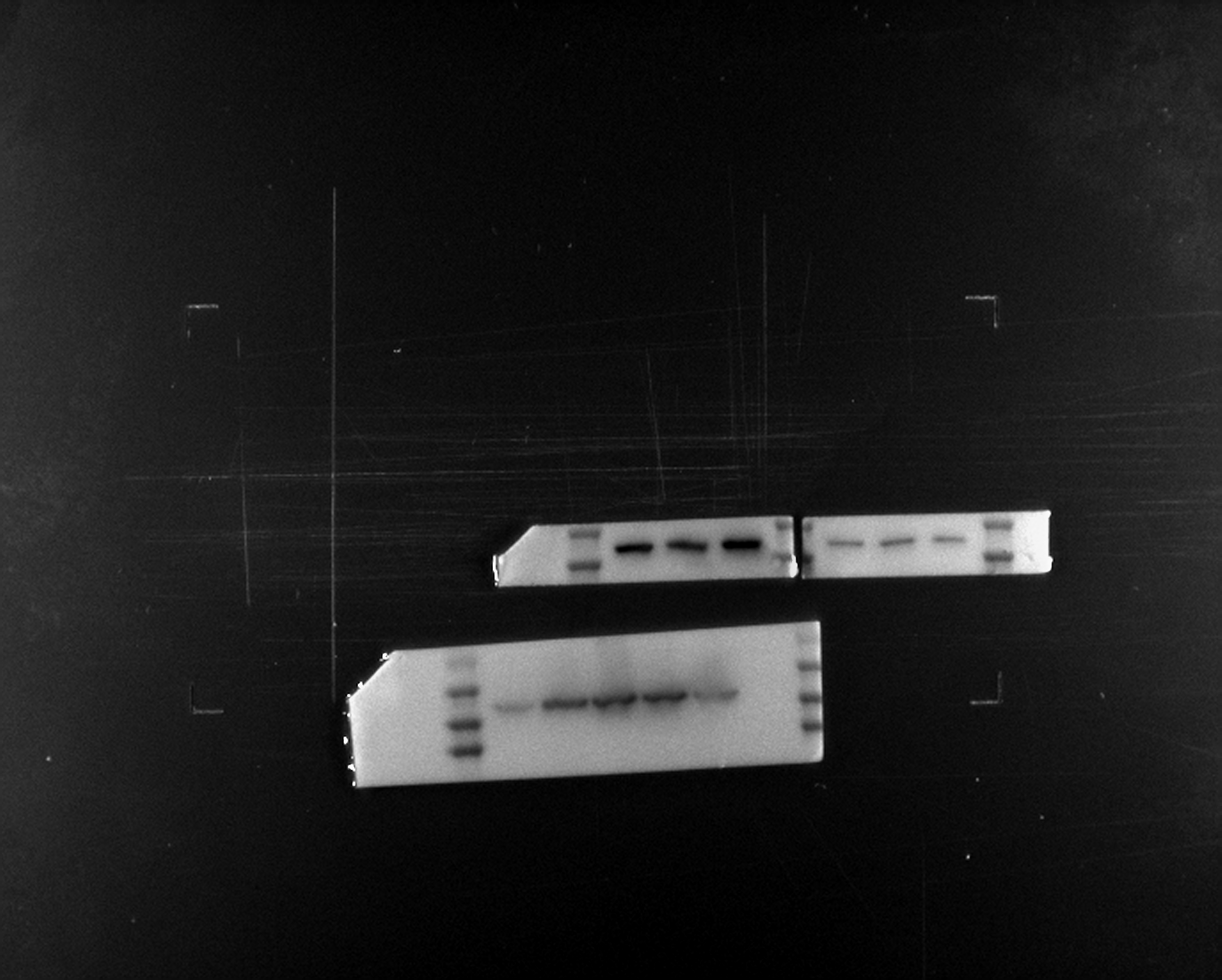

Supplement: Supplementary file 19 — Figure EV8 Source Data [file 44318_2026_832_MOESM19_ESM.zip › Figure EV8/C/β-actin.tif]

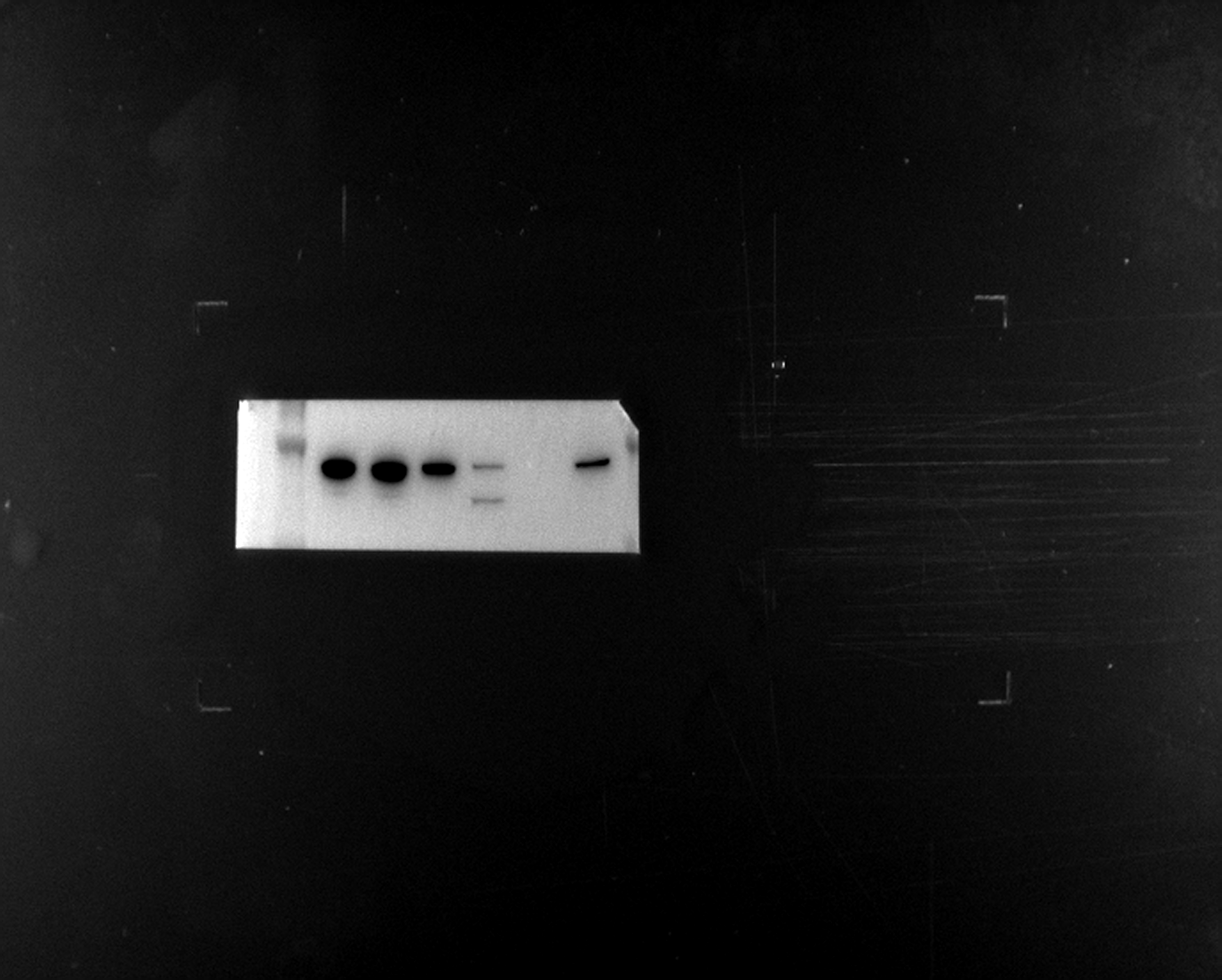

Supplement: Supplementary file 20 — Figure EV9 Source Data [file 44318_2026_832_MOESM20_ESM.zip › C/FLAG-2.tif]
