# Supplementary material for: The oocyte-enriched metabolite serotonin alleviates cellular senescence and aging phenotypes in the mouse
Source: EMBO J. 2026 Jun 16;45(14):4849–86. doi: 10.1038/s44318-026-00832-x (PMC13373241; doi:10.1038/s44318-026-00832-x)
Supplement: Supplementary file 21 — Expanded View Figures [file 44318_2026_832_MOESM21_ESM.pdf]

# Expanded View Figures

**Figure EV1. The metabolic landscape from oocytes to zygotes embryos stage.**

(**A**) QC chart of 10,000 FACS-sorted mouse cells. (**B**) Heatmap showing the relative abundance of metabolites between MII oocytes and 2PN zygote embryos. Peak areas for each detected metabolite were normalized against the total ion count of that sample. $n = 3$ biological replicates per stage. Each biological replicate was from 100 pooled embryos at either the MII or 2PN stage. (**C**) A PCA plot of MII oocytes and 2PN zygote embryos targeted metabolomics ($n = 124$ metabolites per sample). (**D**) Volcano plot analysis of MII oocytes and 2PN zygote embryos targeted metabolomics. Volcano plots were generated in MetaboAnalystR using a two-sample Student's $t$ test and fold change. Significant features were defined as $|FC| \geq 2$ with a raw (unadjusted) $P < 0.05$. $n = 3$ biological repeats. (**E–G**) Comparison of the relative abundance of metabolites in MII and 2PN embryos. *$P < 0.05$, **$P < 0.01$, ***$P < 0.001$ according to the two-tailed unpaired $t$ test. (**E**) $P = 0.05$; (**F**) $P = 0.08$; (**G**) $P = 0.006$. $n = 3$ biological repeats. Data are presented as mean ± SD. Source data are available online for this figure.

© The Author(s)

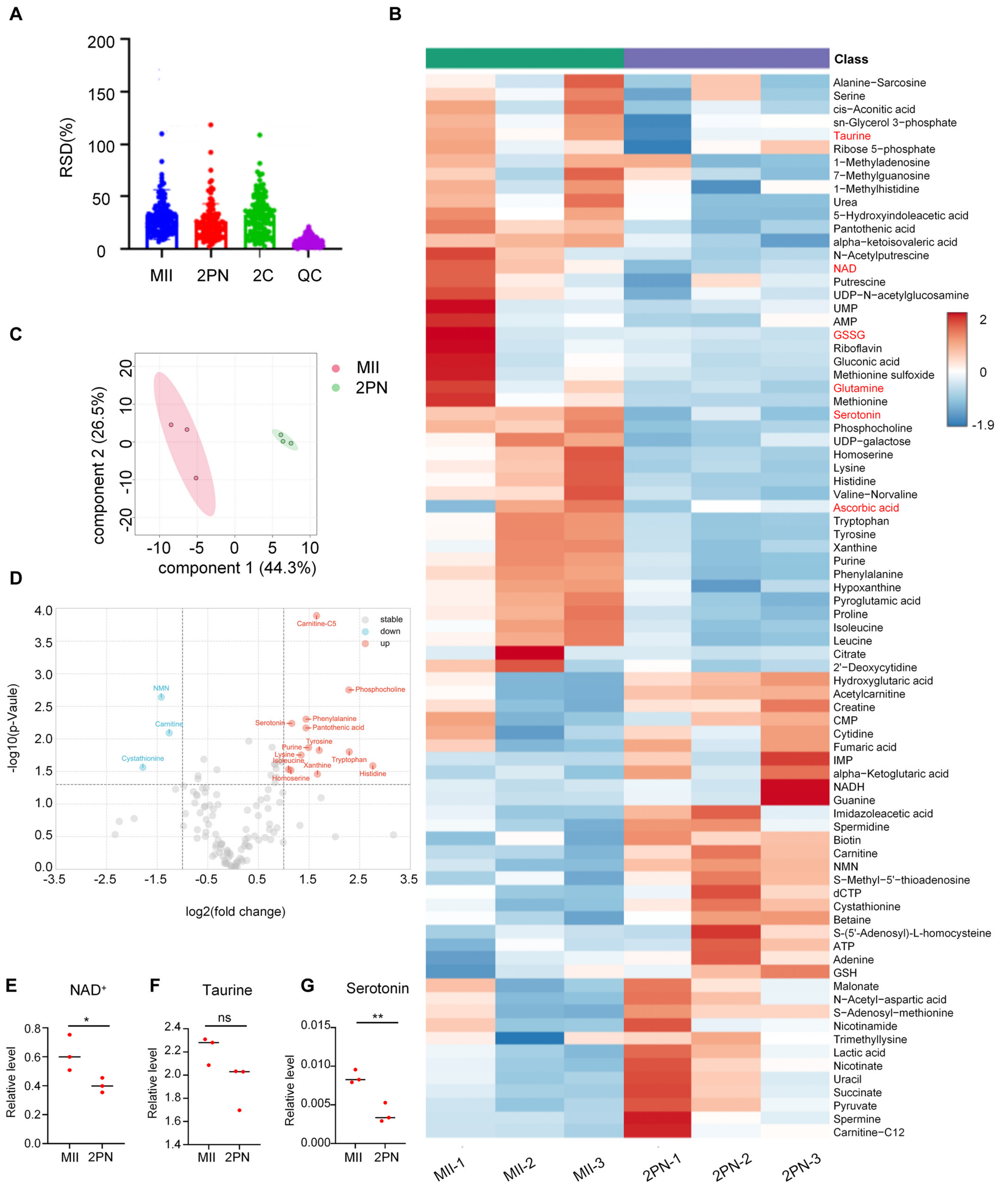
A
RSD(%)
200
150
100
50
0
MII
2PN
2C
QC
B
Class
Alanine−Sarcosine
Serine
cis−Aconitic acid
sn−Glycerol 3−phosphate
Taurine
Ribose 5−phosphate
1−Methyladenosine
7−Methylguanosine
1−Methylhistidine
Urea
5−Hydroxyindoleacetic acid
Pantothenic acid
alpha−ketoisovaleric acid
N−Acetylputrescine
NAD
Putrescine
UDP−N−acetylglucosamine
UMP
AMP
GSSG
Riboflavin
Gluconic acid
Methionine sulfoxide
Glutamine
Methionine
Serotonin
Phosphocholine
UDP−galactose
Homoserine
Lysine
Histidine
Valine−Norvaline
Ascorbic acid
Tryptophan
Tyrosine
Xanthine
Purine
Phenylalanine
Hypoxanthine
Pyroglutamic acid
Proline
Isoleucine
Leucine
Citrate
2'−Deoxycytidine
Hydroxyglutaric acid
Acetylcarnitine
Creatine
CMP
Cytidine
Fumaric acid
IMP
alpha−Ketoglutaric acid
NADH
Guanine
Imidazoleacetic acid
Spermidine
Biotin
Carnitine
NMN
S−Methyl−5'−thioadenosine
dCTP
Cystathionine
Betaine
S−(5'−Adenosyl)−L−homocysteine
ATP
Adenine
GSH
Malonate
N−Acetyl−aspartic acid
S−Adenosyl−methionine
Nicotinamide
Trimethyllysine
Lactic acid
Nicotinate
Uracil
Succinate
Pyruvate
Spermine
Carnitine−C12
2
0
-1.9
MII-1
MII-2
MII-3
2PN-1
2PN-2
2PN-3
C
MII
2PN
component 2 (26.5%)
component 1 (44.3%)
20
10
0
-10
-20
-10
-5
0
5
10
D
-log10(p-Vaule)
log2(fold change)
stable
down
up
Carnitine-C5
Phosphocholine
NMN
Carnitine
Cystathionine
Serotonin
Phenylalanine
Pantothenic acid
Tyrosine
Purine
Lysine
Isoleucine
Xanthine
Tryptophan
Histidine
Homoserine
4.0
3.5
3.0
2.5
2.0
1.5
1.0
0.5
0.0
-3.5
-2.5
-1.5
-0.5
0.5
1.5
2.5
3.5
E
NAD⁺
*
Relative level
0.8
0.6
0.4
0.2
0.0
MII
2PN
F
Taurine
ns
Relative level
2.4
2.2
2.0
1.8
1.6
1.4
MII
2PN
G
Serotonin
**
Relative level
0.015
0.010
0.005
0.000
MII
2PN

 © The Author(s)

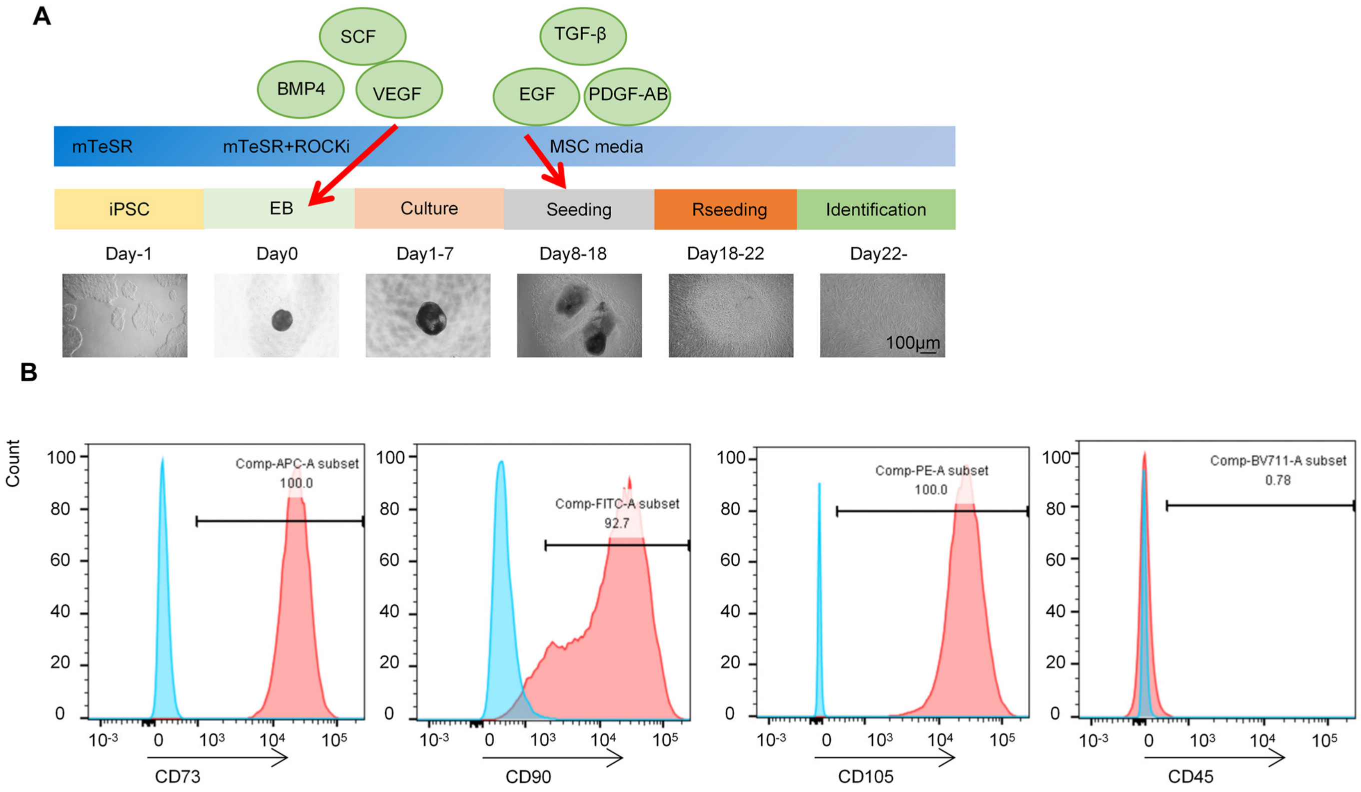


**Figure EV2. Process of differentiation of iPSC to MSC.**

(**A**) Flowchart of iPSC differentiation to MSCs(iPSC-derived mesenchymal stem/progenitor-like cells, $CD73^{+}/CD90^{+}/CD105^{+}/CD45^{-}$). Scale bar, 100 μm. (**B**) Flow cytometry analysis of CD73, CD90, CD105 and CD45 for MSCs.

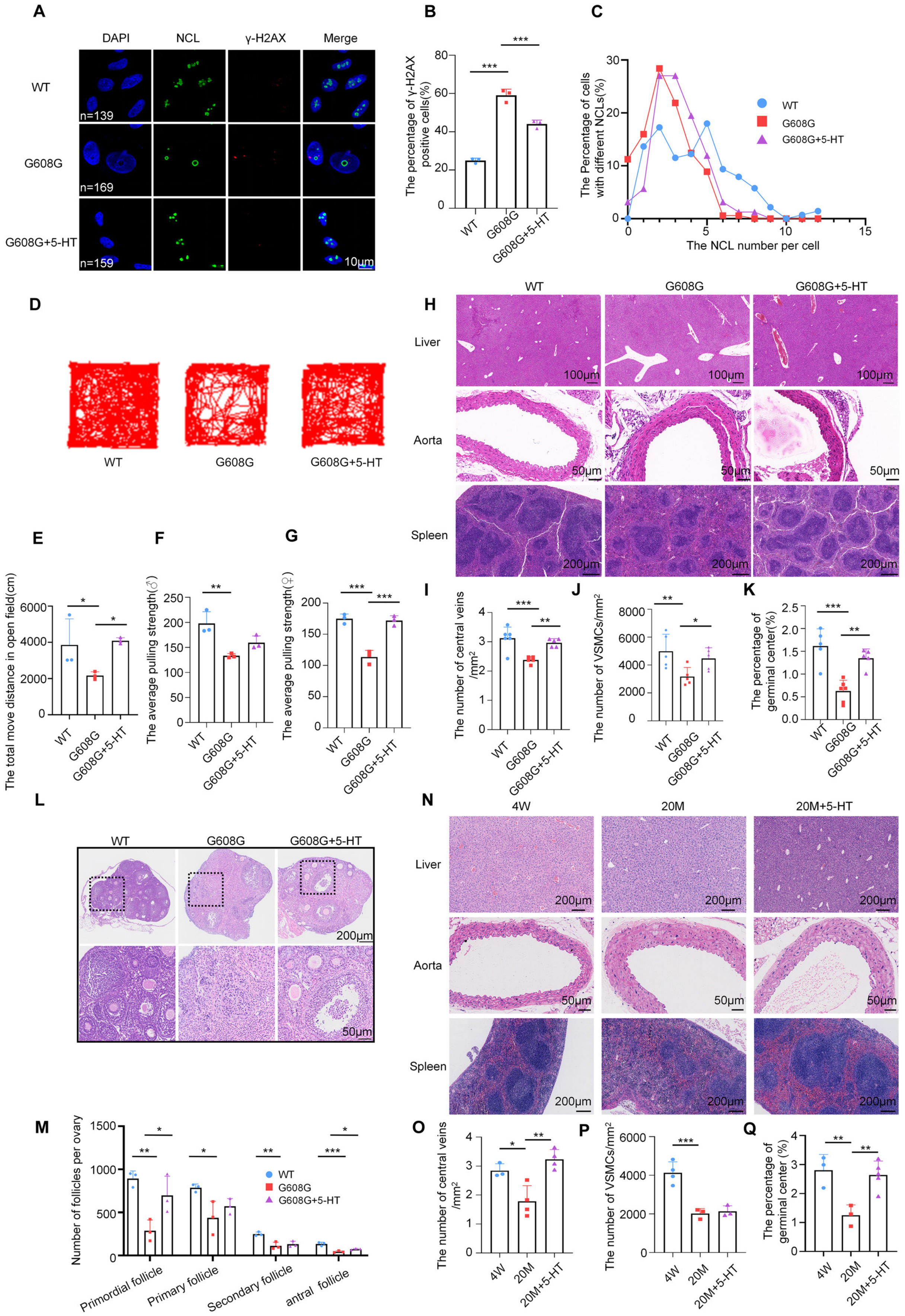
A
DAPI
NCL
γ-H2AX
Merge
WT
G608G
G608G+5-HT
n=139
n=169
n=159
10μm
B
The percentage of γ-H2AX positive cells(%)
C
The Percentage of cells with different NCLs(%)
The NCL number per cell
D
E
The total move distance in open field(cm)
F
The average pulling strength(♂)
G
The average pulling strength(♀)
H
Liver
Aorta
Spleen
I
The number of central veins /mm²
J
The number of VSMCs/mm²
K
The percentage of germinal center(%)
L
M
Number of follicles per ovary
Primordial follicle
Primary follicle
Secondary follicle
antral follicle
N
4W
20M
20M+5-HT
O
P
Q

 © The Author(s)

**Figure EV3. 5-HT affects classic hallmarks associated with aging.**

(**A–C**) Immunofluorescence analysis (**A**) and quantification of γ-H2AX (**B**) and NCL (**C**) in WT MSC and G608G MSC with or without 5-HT treatment. $^{*}P < 0.05$, $^{**}P < 0.01$, $^{***}P < 0.001$ according to the one-way ANOVA test. (**B**) $P < 0.001$, and $P < 0.001$. $n = 3$ biological repeats. Scale bar, 10 μm. (**D**, **E**) Open-field trajectory diagram (**D**) of 5-month-old WT and G608G mice treated with 5-HT or not (from 1-month-old to 5-month-old). Quantitative analysis of the total distance covered (**E**). $^{*}P < 0.05$, $^{**}P < 0.01$, $^{***}P < 0.001$ according to the one-way ANOVA test. (**E**) $P = 0.05$, and $P = 0.03$. $n = 3$ biological repeats. (**F**, **G**) Quantitative analysis of the average pulling strength of male (**F**) and female (**G**) 5-month-old WT and G608G mice treated with 5-HT or not, $^{*}P < 0.05$, $^{**}P < 0.01$, $^{***}P < 0.001$ according to the one-way ANOVA test. (**F**) $P = 0.003$, and $P = 0.09$; (**G**) $P < 0.001$, and $P < 0.001$. $n = 3$ biological repeats. (**H–K**) Histological analysis (HE) (**H**) of the liver, spleen, and aorta of 5-month-old WT and G608G mice treated with 5-HT or not; Quantitative analysis of the number of central veins per unit area ($mm^2$) in the liver (**I**), the average VSMC number per unit area ($mm^2$) in the aorta (**J**), and the percentage of germinal center area in the spleen (**K**). $^{*}P < 0.05$, $^{**}P < 0.01$, $^{***}P < 0.001$ according to the one-way ANOVA test. (**I**) $P < 0.001$, and $P = 0.006$; (**J**) $P = 0.008$, and $P = 0.05$; (**K**) $P < 0.001$, and $P = 0.003$. $n = 5$–6 biological repeats. Scale bar, 100, 200 and 50 μm. (**L**) HE section of the ovary of 4-month-old WT and G608G mice treated with 5-HT or not. Scale bar, 200 and 50 μm. (**M**) Quantitative analysis of the number of follicles in each layer of ovaries of G608G mice treated with 5-HT or not. $^{*}P < 0.05$, $^{**}P < 0.01$, $^{***}P < 0.001$ according to the one-way ANOVA test. $P$ values from left to right: $P = 0.008$, and $P = 0.04$; $P = 0.03$, and $P = 0.44$; $P = 0.007$, and $P = 0.8$; $P < 0.001$, and $P = 0.04$. $n = 3$ biological repeats. (**N–Q**) Histological analysis (HE) (**N**) of the liver, spleen, and aorta of 4-week-old and 20-month-old C57 mice treated with 5-HT or not (from 12-month-old to 20-month-old); Quantitative analysis of the number of central veins per unit area ($mm^2$) in the liver (**O**), the average VSMC number per unit area ($mm^2$) in the aorta (**P**), and the percentage of germinal center area in the spleen (**Q**). $^{*}P < 0.05$, $^{**}P < 0.01$, $^{***}P < 0.001$ according to the one-way ANOVA test. (**O**) $P = 0.02$, and $P = 0.003$; (**P**) $P < 0.001$, and $P = 0.94$; (**Q**) $P = 0.009$, and $P = 0.009$. $n = 3$–4 biological repeats. Scale bar, 200 and 50 μm. Data are presented as mean ± SD. Source data are available online for this figure.

© The Author(s)

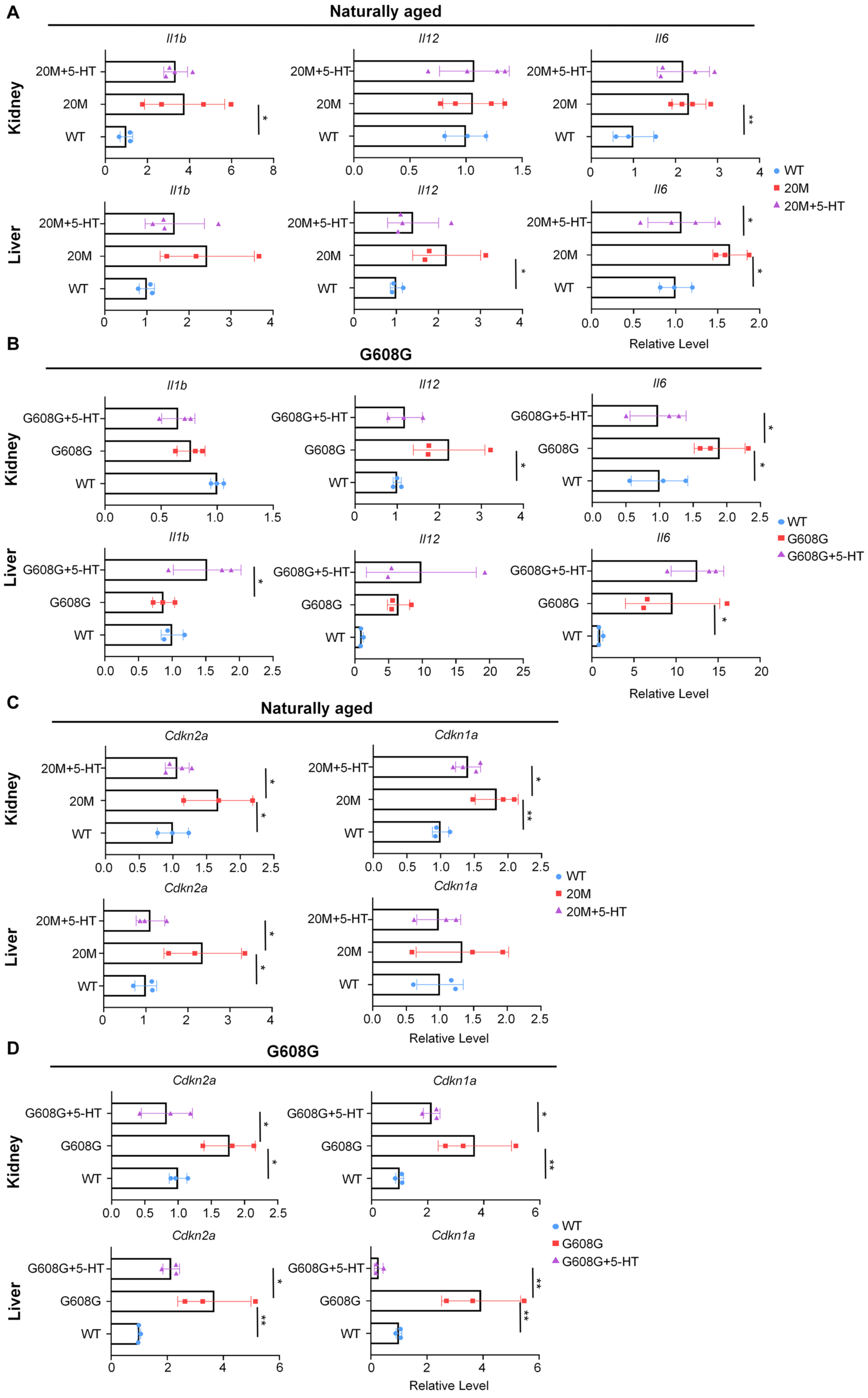
A
Naturally aged
Kidney
Liver
Il1b
Il12
Il6
20M+5-HT
20M
WT
Relative Level
B
G608G
G608G+5-HT
G608G
WT
Relative Level
C
Naturally aged
Cdkn2a
Cdkn1a
Relative Level
D
G608G
Cdkn2a
Cdkn1a
Relative Level

 © The Author(s)

**Figure EV4. 5-HT's lifespan-extending effects in G608G mice.**

(**A**, **B**) RT-qPCR analysis of *Il1b*, *Il12* and *Il6* mRNA expression of liver and kidney in naturally aged and G608G mice treated with 5-HT or not. *$P < 0.05$, **$P < 0.01$, ***$P < 0.001$ according to the one-way ANOVA test. $P$ values from left to right: (**A**) Kidney: $P = 0.02$, and $P = 0.64$; $P = 0.77$, and $P = 0.95$; $P = 0.01$, and $P = 0.72$. Liver: $P = 0.12$, and $P = 0.43$; $P = 0.04$, and $P = 0.12$; $P = 0.03$, and $P = 0.04$. (**B**) Kidney: $P = 0.05$, and $P = 0.28$; $P = 0.03$, and $P = 0.06$; $P = 0.04$, and $P = 0.03$. Liver: $P = 0.65$, and $P = 0.05$; $P = 0.21$, and $P = 0.42$; $P = 0.03$, and $P = 0.37$. $n = 3–4$ biological repeats. (**C**, **D**) RT-qPCR analysis of *Cdkn2a* and *Cdkn1a* mRNA expression of liver and kidney in naturally aged and G608G mice treated with 5-HT or not. *$P < 0.05$, **$P < 0.01$, ***$P < 0.001$ according to the one-way ANOVA test. (**C**) Kidney: $P = 0.04$, and $P = 0.04$; $P = 0.002$, and $P = 0.04$. Liver: $P = 0.03$, and $P = 0.04$; $P = 0.43$, and $P = 0.41$. (**D**) Kidney: $P = 0.03$, and $P = 0.01$; $P = 0.006$, and $P = 0.05$. Liver: $P = 0.005$, and $P = 0.05$; $P = 0.005$, and $P = 0.002$. $n = 3–4$ biological repeats. Data are presented as mean ± SD. Source data are available online for this figure.

© The Author(s)

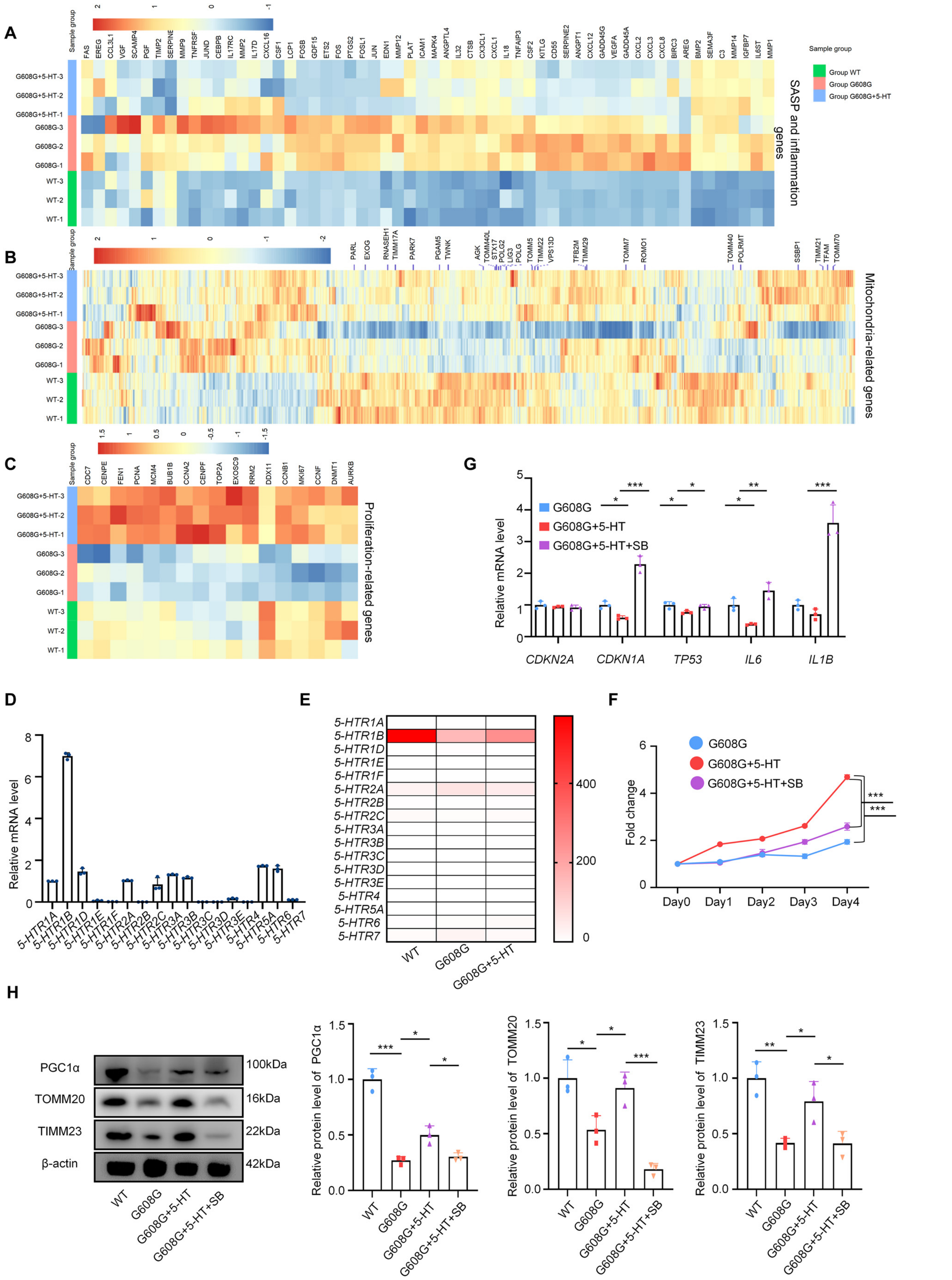
A
B
C
D
E
F
G
H
Sample group
Group WT
Group G608G
Group G608G+5-HT
SASP and inflammation genes
Mitochondria-related genes
Proliferation-related genes
Relative mRNA level
Fold change
Day0
Day1
Day2
Day3
Day4
G608G
G608G+5-HT
G608G+5-HT+SB
CDKN2A
CDKN1A
TP53
IL6
IL1B
PGC1α
TOMM20
TIMM23
β-actin
100kDa
16kDa
22kDa
42kDa
WT
Relative protein level of PGC1α
Relative protein level of TOMM20
Relative protein level of TIMM23

 © The Author(s)

**Figure EV5. Effects of 5-HT supplementation on transcriptome of G608G MSC.**

(**A–C**) Heatmaps of the genes related to inflammation (**A**), mitochondria (**B**), and proliferation (**C**) of WT MSC and G608G MSC treated with 5-HT or not. (**D**) RT-qPCR analysis of 5-HT receptor subtypes in MSC. $n = 3$ biological repeats. (**E**) Heatmap of expression levels of all 5-HT receptors in MSC from RNA sequencing results mentioned above. (**F**) CCK-8 evaluation of proliferation of G608G MSC treated with 5-HT or 5-HT + SB (SB: SB-224289 hydrochloride). *$P < 0.05$, **$P < 0.01$, ***$P < 0.001$ according to the two-way ANOVA test. G608G vs G608G + 5-HT: $P < 0.001$. G608G + 5-HT vs G608G + 5-HT + SB: $P < 0.001$. $n = 3$ biological repeats. (**G**) RT-qPCR analysis of *CDKN2A*, *CDKN1A*, *TP53*, *IL6* and *IL1B* mRNA expression in G608G MSC treated with 5-HT or 5-HT + SB. *$P < 0.05$, **$P < 0.01$, ***$P < 0.001$ according to the one-way ANOVA test. $P$ values from left to right: $P = 0.69$, and $P = 0.93$; $P = 0.02$, and $P < 0.001$; $P = 0.01$, and $P = 0.03$; $P = 0.02$, and $P = 0.001$; $P = 0.42$, and $P < 0.001$. $n = 3$ biological repeats. (**H**) Western blots of PGC1α, TOMM20, and TIMM23 protein levels in G608G MSC treated with 5-HT or 5-HT + SB. Right: Quantification of the protein levels relative to β-actin. *$P < 0.05$, **$P < 0.01$, ***$P < 0.001$ according to the one-way ANOVA test. PGC1α: $P < 0.001$, $P = 0.01$, and $P = 0.03$; TOMM20: $P = 0.01$, $P = 0.03$, and $P < 0.001$; TIMM23: $P = 0.003$, $P = 0.03$, and $P = 0.03$. $n = 3$ biological repeats. Data are presented as mean ± SD. Source data are available online for this figure.

© The Author(s)

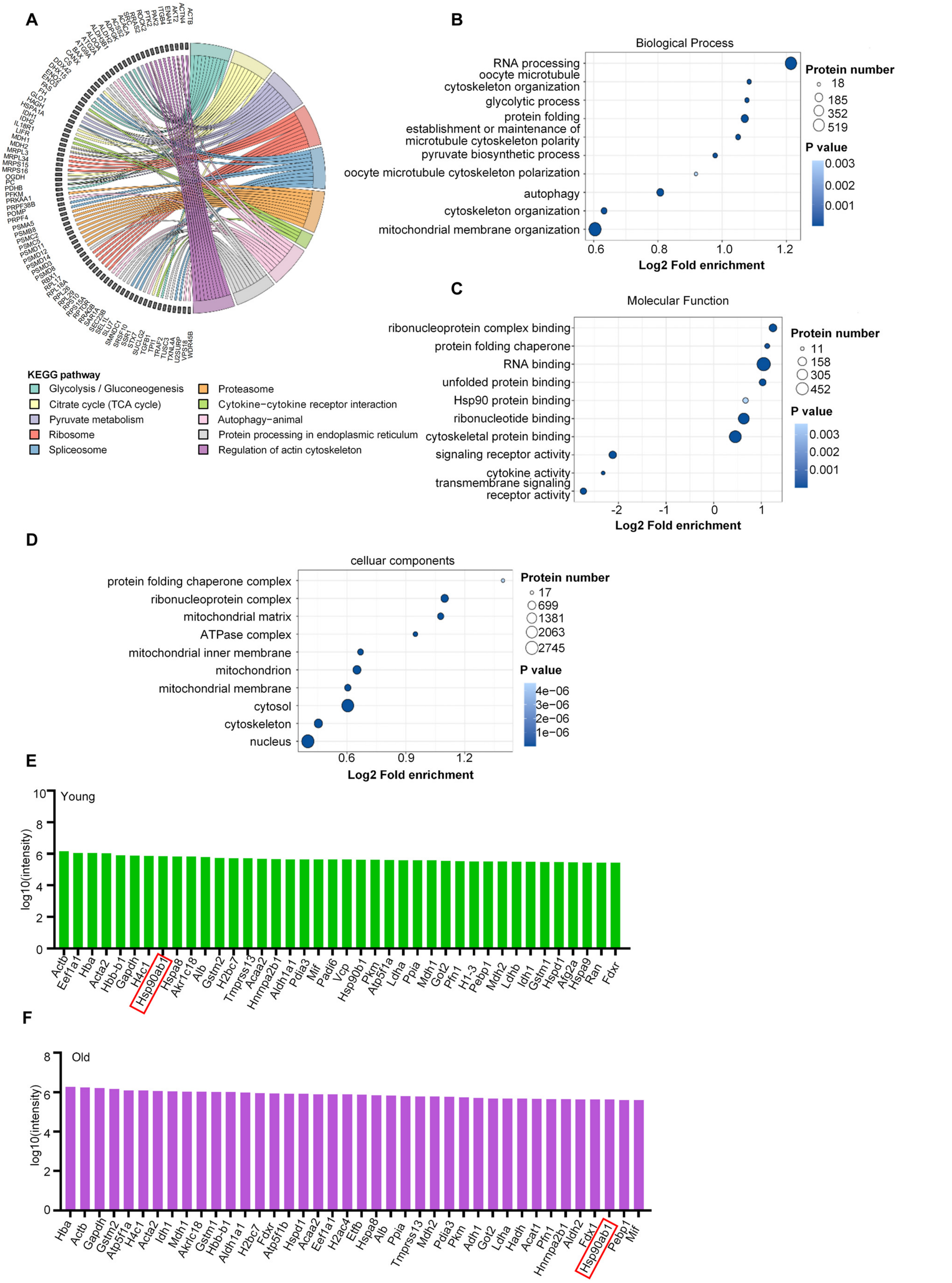
A
KEGG pathway
Glycolysis / Gluconeogenesis
Citrate cycle (TCA cycle)
Pyruvate metabolism
Ribosome
Spliceosome
Proteasome
Cytokine−cytokine receptor interaction
Autophagy−animal
Protein processing in endoplasmic reticulum
Regulation of actin cytoskeleton
B
Biological Process
RNA processing
oocyte microtubule cytoskeleton organization
glycolytic process
protein folding
establishment or maintenance of microtubule cytoskeleton polarity
pyruvate biosynthetic process
oocyte microtubule cytoskeleton polarization
autophagy
cytoskeleton organization
mitochondrial membrane organization
0.6 0.8 1.0 1.2
Log2 Fold enrichment
Protein number
18
185
352
519
P value
0.003
0.002
0.001
C
Molecular Function
ribonucleoprotein complex binding
protein folding chaperone
RNA binding
unfolded protein binding
Hsp90 protein binding
ribonucleotide binding
cytoskeletal protein binding
signaling receptor activity
cytokine activity
transmembrane signaling receptor activity
-2 -1 0 1
Log2 Fold enrichment
Protein number
11
158
305
452
P value
0.003
0.002
0.001
D
celluar components
protein folding chaperone complex
ribonucleoprotein complex
mitochondrial matrix
ATPase complex
mitochondrial inner membrane
mitochondrion
mitochondrial membrane
cytosol
cytoskeleton
nucleus
0.6 0.9 1.2
Log2 Fold enrichment
Protein number
17
699
1381
2063
2745
P value
4e−06
3e−06
2e−06
1e−06
E
Young
log10(intensity)
0 2 4 6 8 10
Actb Eef1a1 Hba Acta2 Hbb-b1 Gapdh H4c1 Hsp90ab1 Hspa8 Akr1c18 Alb Gstm2 H2bc7 Tmprss13 Acaa2 Hnrnpa2b1 Aldh1a1 Pdia3 Mif Padi6 Vcp Hsp90b1 Pkm Atp5f1a Ldha Ppia Mdh1 Got2 Pfn1 H1-3 Pebp1 Mdh2 Ldhb Idh1 Gstm1 Hspd1 Afg2a Hspa9 Ran Fdxr
F
Old
log10(intensity)
0 2 4 6 8
Hba Actb Gapdh Gstm2 Atp5f1a H4c1 Acta2 Idh1 Mdh1 Akr1c18 Gstm1 Hbb-b1 Aldh1a1 H2bc7 Fdxr Atp5f1b Hspd1 Acaa2 Eef1a1 H2ac4 Etfb Hspa8 Alb Ppia Tmprss13 Mdh2 Pdia3 Pkm Adh1 Got2 Ldha Hadh Acat1 Pfn1 Hnrnpa2b1 Aldh2 Fdx1 Hsp90ab1 Pebp1 Mif

 © The Author(s)

**Figure EV6. The potential targetes for serotonylation in MSCs.**

(**A**) KEGG analysis of seroyonylation proteins after mass spectrometry. (**B–D**) GO analysis of serotonylation proteins after mass spectrometry. GO enrichment was performed by over-representation analysis using a hypergeometric test (Fisher's exact test), followed by Benjamini–Hochberg FDR correction (adjusted *P* values/*q* values). (**E**, **F**) Heatmap of the top 40 enriched proteins of the ovaries 4-week-old (Young) and 12-month-old mice (Old). Source data are available online for this figure.

© The Author(s)

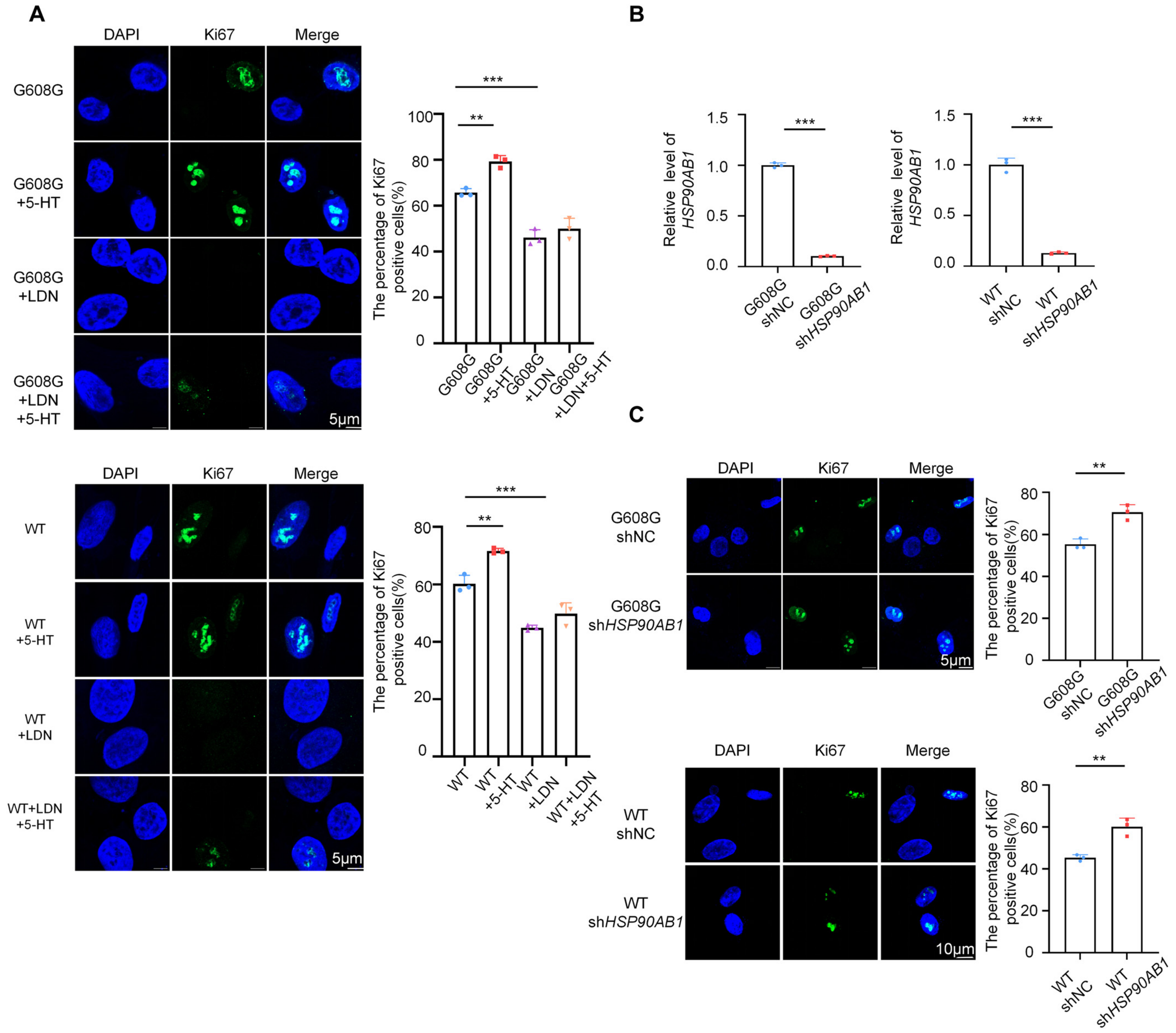


**Figure EV7. 5-HT block cellular senescence by serotonylation of HSP90β.**

(**A**) Immunofluorescence analysis (left) and quantification of Ki67 (right) in MSCs treated with 5-HT, LDN or LDN + 5-HT. upper: G608G MSCs, lower: WT MSCs. $*P < 0.05$, $**P < 0.01$, $***P < 0.001$ according to the one-way ANOVA test. G608G vs G608 + 5-HT: $P = 0.004$; G608G vs G608G + LDN: $P < 0.001$. WT vs WT + 5-HT: $P = 0.002$; WT vs WT + LDN: $P < 0.001$. $n = 3$ biological repeats. Scale bar, 5 μm. (**B**) RT-qPCR analysis of *HSP90AB1* mRNA expression in MSCs transfected with control shRNA or shRNA against HSP90AB1. Left: G608G MSCs, Right: WT MSCs. $*P < 0.05$, $**P < 0.01$, $***P < 0.001$ according to the two-tailed unpaired $t$ test. $P < 0.001$ (left), and $P < 0.001$ (right). $n = 3$ biological repeats. (**C**) Immunofluorescence analysis (left) and quantification of Ki67 (right) in MSCs transfected with control shRNA or shRNA against HSP90AB1. upper: G608G MSCs, lower: WT MSCs. $*P < 0.05$, $**P < 0.01$, $***P < 0.001$ according to the two-tailed unpaired $t$ test. $P = 0.004$ (upper), and $P = 0.004$ (lower). $n = 3$ biological repeats. Scale bar, 5 and 10 μm. Data are presented as mean ± SD. Source data are available online for this figure.

 © The Author(s)

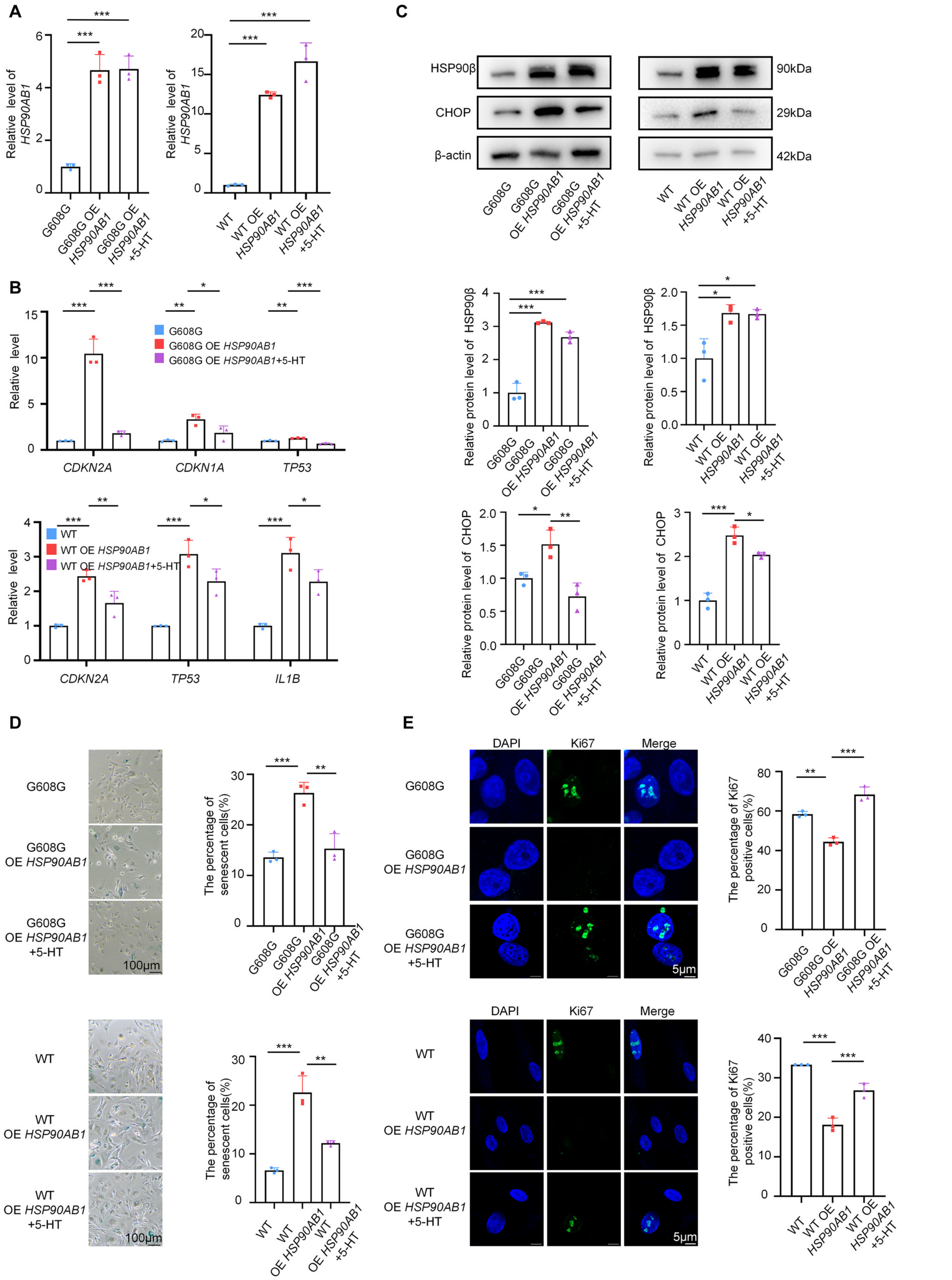
A
Relative level of HSP90AB1
G608G
G608G OE HSP90AB1
G608G OE HSP90AB1 +5-HT
WT
WT OE HSP90AB1
WT OE HSP90AB1 +5-HT
B
Relative level
CDKN2A
CDKN1A
TP53
IL1B
C
HSP90β
CHOP
β-actin
90kDa
29kDa
42kDa
Relative protein level of HSP90β
Relative protein level of CHOP
D
The percentage of senescent cells(%)
100μm
E
DAPI
Ki67
Merge
5μm
The percentage of Ki67 positive cells(%)

**Figure EV8. HSP90β is a potential target for inhibiting cell senescence.**

(**A**) RT-qPCR analysis of *HSP90AB1* mRNA expression in MSCs and HSP90AB1-overexpression MSCs with or without 5-HT treatment. Left: G608G MSCs, Right: WT MSCs. *$P < 0.05$, **$P < 0.01$, ***$P < 0.001$ according to the one-way ANOVA test. $P$ values from left to right: $P < 0.001$, and $P < 0.001$; $P < 0.001$, and $P < 0.001$. $n = 3$ biological repeats. (**B**) RT-qPCR analysis of *CDKN2A*, *TP53*, and *IL1B* or *CDKN1A* mRNA expression in MSCs and HSP90AB1-overexpression MSCs with or without 5-HT treatment. Upper: G608G MSCs, Lower: WT MSCs. *$P < 0.05$, **$P < 0.01$, ***$P < 0.001$ according to the one-way ANOVA test. $P$ values from left to right: G608G MSCs: $P < 0.001$, and $P < 0.001$; $P = 0.005$, and $P = 0.04$; $P = 0.002$, and $P < 0.001$. WT MSCs: $P < 0.001$, and $P = 0.003$; $P < 0.001$, $P = 0.02$; $P < 0.001$, and $P = 0.02$. $n = 3$ biological repeats. (**C**) Western blots of HSP90β, CHOP protein levels in MSCs and HSP90AB1-overexpression MSCs with or without 5-HT treatment. Left: G608G MSCs, Right: WT MSCs. Lower: Quantification of the protein levels relative to β-actin. *$P < 0.05$, **$P < 0.01$, ***$P < 0.001$ according to the one-way ANOVA test. HSP90β: $P < 0.001$, and $P < 0.001$ (left); $P = 0.01$, and $P = 0.01$ (right).CHOP: $P = 0.03$, and $P = 0.004$ (left); $P < 0.001$, $P = 0.03$ (right). $n = 3$ biological repeats. (**D**) SA-β-Gal staining (left) and quantification (right) of MSCs and HSP90AB1-overexpression MSCs with or without 5-HT treatment. Upper: G608G MSCs, Lower: WT MSCs. *$P < 0.05$, **$P < 0.01$, ***$P < 0.001$ according to the one-way ANOVA test. $P < 0.001$, and $P = 0.002$ (upper); $P < 0.001$, and $P = 0.002$ (lower). $n = 3$ biological repeats. Scale bar, 100 µm. (**E**) Immunofluorescence analysis (left) and quantification of Ki67 (right) in MSCs and HSP90AB1-overexpression MSCs with or without 5-HT treatment. upper: G608G MSCs, lower: WT MSCs. *$P < 0.05$, **$P < 0.01$, ***$P < 0.001$ according to the one-way ANOVA test. $P = 0.002$, and $P < 0.001$ (upper); $P < 0.001$, and $P < 0.001$ (lower). $n = 3$ biological repeats. Scale bar, 5 µm. Data are presented as mean ± SD. Source data are available online for this figure.

 © The Author(s)

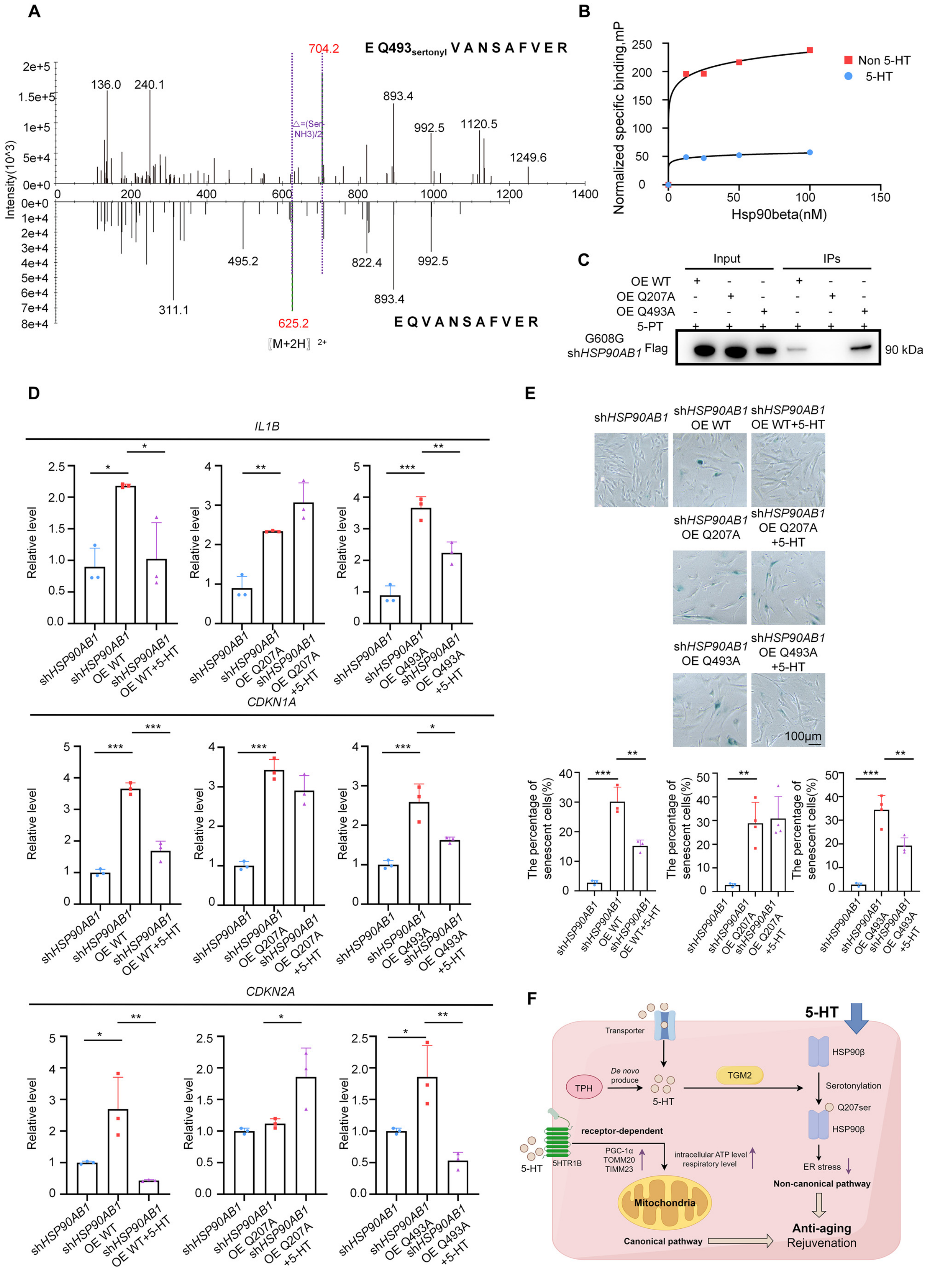
A
E Q493sertonyl V A N S A F V E R
704.2
136.0
240.1
893.4
992.5
1120.5
1249.6
Intensity(10^3)
495.2
822.4
992.5
893.4
311.1
625.2
〚M+2H〛2+
E Q V A N S A F V E R
B
Normalized specific binding,mP
Hsp90beta(nM)
Non 5-HT
5-HT
C
Input
IPs
OE WT
OE Q207A
OE Q493A
5-PT
G608G shHSP90AB1
Flag
90 kDa
D
IL1B
CDKN1A
CDKN2A
Relative level
shHSP90AB1
shHSP90AB1 OE WT
shHSP90AB1 OE WT+5-HT
shHSP90AB1 OE Q207A
shHSP90AB1 OE Q207A +5-HT
shHSP90AB1 OE Q493A
shHSP90AB1 OE Q493A +5-HT
E
100μm
The percentage of senescent cells(%)
F
5-HT
Transporter
HSP90β
De novo produce
TPH
5-HT
TGM2
Serotonylation
Q207ser
HSP90β
receptor-dependent
5-HT
5HTR1B
PGC-1α
TOMM20
TIMM23
intracellular ATP level
respiratory level
ER stress
Non-canonical pathway
Mitochondria
Canonical pathway
Anti-aging
Rejuvenation

**Figure EV9. Mutation of Gln207 at HSP90β inhibits the role of 5-HT in rejuvenation effects.**

(**A**) LC-MS/MS analysis at Gln493 of TGM2-transamidated serotonin to full-length HSP90β, serotonylation peptide (top), unserotonylation peptide (below). ρ = Ser-NH3 is the difference in mass-charge ratio between two parent ion, where the difference in mass-charge ratio is 159. (**B**) The ATPase activity of HSP90β. Fluorescence was measured at λex 485 nm, λex 530 nm using a Bio-Tek fluorescent microplate reader. (**C**) shHSP90AB1 G608G MSCs were transfected with mRFP-fused lentiviruses expressing either WT-HSP90β or Gln to Ala(Q207A, Q493A) mutated HSP90β. Cells were treated with 5 μM 5-PT or not. 5-PT was immunoprecipitated by beads, and Flag-HSP90β were detected. IP immunoprecipitation. (**D**) shHSP90AB1 G608G MSCs were transfected with mRFP-fused lentiviruses expressing either WT-HSP90β or Gln to Ala(Q207A, Q493A) mutated HSP90β. Cells were treated with 5-HT or not. *IL1B*, *CDKN1A* and *CDKN2A* mRNA expression was detected. *$P < 0.05$, **$P < 0.01$, ***$P < 0.001$ according to the one-way ANOVA test. $P$ values from left to right: *IL1B*: $P = 0.01$, and $P = 0.02$; $P = 0.005$, and $P = 0.08$; $P < 0.001$, and $P = 0.005$. *CDKN1A*: $P < 0.001$, and $P < 0.001$; $P < 0.001$, and $P = 0.13$; $P < 0.001$, and $P = 0.01$. *CDKN2A*: $P = 0.03$, and $P = 0.007$; $P = 0.85$, and $P = 0.04$; $P = 0.03$, and $P = 0.004$. $n = 3$ biological repeats. (**E**) SA-β-Gal staining (upper) and quantification (lower) of shHSP90AB1 G608G MSCs transfected with mRFP-fused lentiviruses expressing either WT-HSP90β or Gln to Ala (Q207A, Q493A) mutated HSP90β, cells were treated with 5-HT or not. *$P < 0.05$, **$P < 0.01$, ***$P < 0.001$ according to the one-way ANOVA test. $P$ values from left to right: $P < 0.001$, and $P = 0.003$; $P = 0.006$, and $P = 0.93$; $P < 0.001$, and $P = 0.002$. $n = 3$–4 biological repeats. Scale bar, 100 μm. (**F**) Schematics showing that 5-HT regulates cellular aging through both classical and non-classical functions, by Figdraw. Data are presented as mean ± SD. Source data are available online for this figure.

 © The Author(s)
