# Supplementary material for: The oocyte-enriched metabolite serotonin alleviates cellular senescence and aging phenotypes in the mouse
Source: EMBO J. 2026 Jun 16;45(14):4849–86. doi: 10.1038/s44318-026-00832-x (PMC13373241; doi:10.1038/s44318-026-00832-x)
Supplement: Supplementary file 2 — Peer Review File [file 44318_2026_832_MOESM2_ESM.pdf]

THE EMBO JOURNAL

The oocyte-enriched metabolite serotonin alleviates cellular senescence and aging phenotypes in the mouse

Yuyan Xu, Ling Zhang, Xufeng Liao, Yuhang Fan, Rouyi Shao, Mengchen Wu, Li Zhang, Min Zhang, Gang Wang, Hailing Zhang, Enze Li, Qixin Wu, Jing Zhao, Jia Zhang, Wenjie Wang, Limeng Cai, Jinjie Zhong, Lidan Hu, Jingjing Wang, Jianhua Mao, Bing Yang, and Jin Zhang

Corresponding authors: Ling Zhang (zling6@zju.edu.cn), Jin Zhang (zhgene@zju.edu.cn)

Review Timeline:

Submission Date:	24th Nov 25
Editorial Decision:	8th Apr 26
	14th Apr 26
Revision Received:	29th Apr 26
Accepted:	20th May 26

Editor: Daniel Klimmeck

Transaction Report:

Please note that the manuscript was previously reviewed at another journal. As EMBO Press has a transfer agreement with that journal, revision was invited based on the reports from that previous external submission.

(Note: With the exception of the correction of typographical or spelling errors that could be a source of ambiguity, letters and reports are not edited. Depending on transfer agreements, referee reports obtained elsewhere may or may not be included in this compilation. Referee reports are anonymous unless the Referee chooses to sign their reports.)

EMBOJ-2025-123120-T

Reviewers' comments:

Reviewer #1 (Remarks to the Author):

The manuscript by Xu et al. describes the potential role of serotonin in early embryonic development and in rescuing certain phenotypes in an aging-prone genetically engineered mouse model with lamin A deficiency. The authors conducted a metabolomic analysis of mouse MII oocytes, zygotes, and 2-cell stage embryos, discovering that several metabolites, which could positively impact cellular or organismal fitness, were enriched in oocytes. One such metabolite appeared to be serotonin, a neurotransmitter whose levels are known to decrease with age. Although serotonin is primarily recognized for its role in the central nervous system, several recent studies have indicated its involvement in regulating inflammation and immunity via serotonin receptors found on immune cells. The authors further investigate serotonin's role outside the central nervous system, proposing that it may contribute to the post-translational modification of HSP90, which they suggest is crucial in delaying age-related changes in G608G mice. Overall, the manuscript reads as two separate, incomplete studies, with significant methodological issues and conclusions not fully supported by the data.

Major points:

1.The authors obtained 2-cell stage embryos after natural mating, which could significantly affect the results due to the hormonal changes that occur in females during the mating process. To ensure more accurate results, all metabolite analyses at the 2-cell stage should be repeated using in vitro fertilization (IVF) of oocytes from the same pool, to allow for direct comparison.

2.In the knockdown experiments with siRNA, the authors aim to test their hypothesis in oocytes. However, it is unclear how this approach impacts the maternally deposited proteins, especially considering that the experiments are brief and involve oocytes that either do not divide or only divide once. Clear controls showing the efficiency of protein knockdowns are essential.

3.On what basis do the authors claim that metabolites found in oocytes could be relevant for rejuvenation or life extension? The transition from discussing oocytes to aging seems somewhat forced. Have the authors demonstrated that me born from eggs with reduced serotonin levels experience shorter lifespans?

4.The experiments analyzing senescence markers in mesenchymal stem cells (MSCs) are not sufficiently detailed. At what stage were these analyses conducted? Did different MSC cultures undergo similar numbers of cell divisions? What occurs after the fourth day of

culture? A more comprehensive cell cycle profile for the various conditions would be helpful. Additionally, the authors claim that lamin B1 levels increase after treatment with 5-HT, but this is not evident in Figure 3h. All panels of Figure 3 are missing a crucial control—wild-type (wt) MSCs and mice treated with 5-HT, which should be included.

5.The statement "However, these studies all focused on the role in neuronal cells and mechanisms of brain aging and cognitive decline; however, the systemic role of 5-HT in contributing to whole-body cellular senescence or rejuvenation, especially in non-neuronal cells, is unclear" is inaccurate. Several studies already highlight serotonin’s role in immune cell regulation.

6. The authors misuse the terms "rejuvenation" and "anti-aging" throughout the manuscript, as no such experiments are presented. The data only show that serotonin supplementation affects the fitness of aging-prone, genetically modified mice. Furthermore, neither of these terms should be used in the context of in vitro cell cultures, and these should be corrected.

7.The in vivo experiments with orally administered 5-HT are unsurprising, given that serotonin has been previously shown to have anti-inflammatory effects. If the authors propose a novel mechanism behind the lifespan extension observed in G608G mice, they need to exclude any influence of 5-HT on the immune system. Additionally, how does the proposed mechanism explain the observed phenotypes in vivo? Does treatment with an HSP90 inhibitor and a selective 5-HTR1B agonist produce similar effects? Can an inhibitor of TGM2 or a 5-HTR1B antagonist reverse the effects of 5-HT in vivo in G608G mice?

8.Since the analysis in Figures 5 and 6 compares MSCs in varying senescent states (greater senescence in G608G, less senescence in others), it is crucial to confirm that the observed differences are directly caused by 5-HT treatment and not simply due to varying cellular states. Does 5-HT treatment produce similar effects on replicative or other types of senescence in normal cell cultures?

9. Does HSP90 serotonylation occur in vivo, and if so, in which cell types? How stable is this modification both in vitro and in vivo? How does it relate to the observed effects of 5-HT in G608G mice?

10.The reprogramming experiment involving the four Yamanaka factors (Figure 3q,r) feels out of place, and no partial reprogramming experiments were conducted in G608G mice.

11.The title of Figure 3 is misleading, as it only demonstrates a delay in some age-related phenotypes, not a reversal.

Reviewer #2 (Remarks to the Author):

Manuscript by Xu et al described rescue of aging phenotypes in several tissues/cell types attributed to premature aging triggered by progeria (mutations in Lamin A) attributed to exposure to 5-HT (serotonin). Authors acknowledge that serotonin has been previously implicated in aging of the brain however its effects on other tissues were not explored. Authors thus tried to connect the metabolomics of oocytes with rejuvenation/function of organelles (mitochondria, ER….) during aging. The data observed represents a potpourri of experiments, which sometimes lack focus.

1、Title is a bit misleading and proposes that Oocytes are enriched in Serotonin. This is true compared to zygotes/2-cell embryos, but other cell types were not profiled. Also, linkage to reprogramming efficiency is not clear, as presumably there is low level of serotonin in zygotes. Yet, zygotes are the ones actively reprograming both paternal sperm nuclei as well as any other somatic cell nuclei inserted in their cytoplasm. Data for metabolites should be included with quantitation in the supplemental table.

2. Authors claim serotonin “rejuvenates” and/or prevents aging. Yet no aging studies were performed at all. All experiments were performed in progeria model of unknown age. This model, while often described as having accelerated aging has primarily a defect of nuclear laminas. Thus, secondary phenotypes triggered by laminopathies contribute to cell senescence in this model with additional secondary defects. True biological aging is driven by more complex mechanisms.

3.Are serotonin levels/ and or receptors affected by progeria mutations? Is there lack of serotonin in patients with laminopathies? Is there change of serotonin in aging? Do patients taking SSRI (antidepressants) exhibit any changes of aging phenotypes?

4.No n values are provided for several graphs (Fig 1h Fig2, f) or are extremely limited with sample size of 7-14 oocytes (Fig 1j), meaning there is likely no replication of experiments (just one trial). Fig 2 c has only 2 replicas and thus statistics cannot be performed.

5.Work with oocytes (using siRNA) is suboptimal. If authors wish to study serotonin pathway in oogenesis they should do so by interfering with its production genetically – in a conditional model. Use of parthenogenetic embryos for assessment of developmental competence is suboptimal and purely represents limitation of siRNA approaches.

6.For experiments with LDN – what is the control for specificity of this compound?

7.Some control experiments are missing. Authors should have performed all experiments with WT animals/cells also treated with 5-HT for proper comparison. Western blot data is not statistically quantitated (Fig 3h, 4c, 6b,7i).

8.The results of serotonylation experiments are intriguing. However, qRT-PCR data as a readout for UPR is not sufficient. Additional markers, cellular consequences should be

explored. Are there defects in any identified serotonylated proteins in progeria patients/animals or is this a product of cell culture environment? List of identified proteins was not provided (only bioinformatic analysis of KEGG. These data should also be made available.

Reviewer #3 (Remarks to the Author):

The authors document an in-vivo modeling of oocyte development and highlight serotonin as a metabolite that exhibits a range of critical roles in cellular reprogramming and potential cell rejuvenation. The article highlights the important role metabolites play, where historically proteins (sic – Yamanaka factors) have been seen as the main biomolecular contributors to phenotypical changes in oocyte biology – this is a novel finding. The authors then proceed to demonstrate that serotonylation of HSP90β has potential as an anti-aging metabolite.

The overall the article is original, written to a high standard, abstract is concise, figures are of a high quality, and the hypothesis and methodologies employed within this study are 'generally' suitable for consideration in this venue – but there are some strong reservations below that should be:

DATA/METHODS/STATS
The flowing corrections need to be addressed:

Comment 1
• Page 2 – The authors describe their approach as a 'comprehensive metabolomics landscape' yet they only measure 256 metabolites and provided data (in associated .xls file) on 124. This needs to be changed to accurately reflect the size of the chemical space that has been measured.

Comment 2
• Page 3 – Pooling 100 embryos into x3 biological replicates need to be justified from an effect size and experimental variability perspective. This is a critical point.
I understand that there can be a limitation in the number of animals/cells that can be extracted, but this is not a reason to compromise the significance in a study. This needs to be discussed and statistically evidenced alongside the use of technical replication in all studies performed.

Comment 3
• Page 10 – Authors mention serotonylation of HSP80β here as the targeted HSP protein – I think this is a typo. Also, is 5-HT really an anti-aging metabolite? Aside from this one

cell model more really needs to be done to confirm this – and this statement needs to be toned down.

Comment 4

• Page 15 – In the LC MS/MS methodology see no mention of QC samples being used – can the authors please explain. Also, there are precise details around the standards that were used in the analysis. This needs to be provided.

Comment 5

• Page 24 – The LC-MS/MS methodology mentions a homemade RP analytical column was used for the shotgun proteomics analysis. More details need to be provided around this. Information around stationary phase (particle size/gaps) where it was sourced and how it put together needs to be provided in order that readers can replicate the science.

Comment 6

• Page 24 – Database Search section. There are several cases of 'xx' being used as a numerical value. I am thinking data is missing here – please provide or explain why it is not there.

Comment 7

• Page 25 – Precise details (similar to the LC MS/MS section) need to be provided around how serotonylation was measured as this method will be markedly different than all other LC MS/MS methods described.

Comment 8

• Page 15 and 26 – Pathway enrichment analysis and Figure 1. Real care needs to be taken when performing enrichment when a targeted list of metabolites is used - all of the following are critical points than need to be addressed in the fullest. It is noted that 262 metabolites are monitored – then 256 are measured. I am presuming that 6 of them are duplicated across +/- modes? This full list needs to be provided as a supplementary alongside full MRM transition m/z values and quad transition voltages (and excel will suffice). Alongside this, a statement needs to be provided around the concept of pathway bias contained within the 256 metabolites that are analyzed. It is highly possible that by including more metabolites in a specific pathway in your list, you are automatically preselecting those pathways in any enrichment analysis – please justify this approach. Also, in section J the p-value red gradient bar doesn't allow for any meaningful level of significance to be determined – this should be adjusted or the specific p-value levels noted on the figure (and any subsequent figures that are similar).

REFERENCES – Overall appropriate use of references is present. There is however an issue between an inconsistent use of abbreviated journal titles – some are full title and others are abbreviated. Also the font is in TNR and the article is in a different font.

Authors' rebuttal response to Reviewers' comments:

Reviewer #1 (Remarks to the Author):

The manuscript by Xu et al. describes the potential role of serotonin in early embryonic development and in rescuing certain phenotypes in an aging-prone genetically engineered mouse model with lamin A deficiency. The authors conducted a metabolomic analysis of mouse MII oocytes, zygotes, and 2-cell stage embryos, discovering that several metabolites, which could positively impact cellular or organismal fitness, were enriched in oocytes. One such metabolite appeared to be serotonin, a neurotransmitter whose levels are known to decrease with age. Although serotonin is primarily recognized for its role in the central nervous system, several recent studies have indicated its involvement in regulating inflammation and immunity via serotonin receptors found on immune cells. The authors further investigate serotonin's role outside the central nervous system, proposing that it may contribute to the post-translational modification of HSP90, which they suggest is crucial in delaying age-related changes in G608G mice. Overall, the manuscript reads as two separate, incomplete studies, with significant methodological issues and conclusions not fully supported by the data.

Answer to the overall comment: *We appreciate the reviewer's critical comments. After carefully considering the suggestions, we acknowledge that some data are irrelevant and some are lack of evidence, we thus thoroughly rearrange the figures and more focused on the illustration of how 5-HT can delay aging , including providing the more complete and solid mechanistic data supporting the role of 5-HT in mitigating aging effects, removing the results of embryos development mediated by 5-HT so that the whole story reads more as a focused story. Thus, we think this new version of the manuscript addressed all the reviewer's comments.*

Major points:

1.The authors obtained 2-cell stage embryos after natural mating, which could significantly affect the results due to the hormonal changes that occur in females during the mating process. To ensure more accurate results, all metabolite analyses at the 2-cell stage should be repeated using in vitro fertilization (IVF) of oocytes from the same pool, to allow for direct comparison.

Answer to comment 1 of reviewer 1:
We appreciate the concern regarding the potential influence of hormonal changes during natural mating on the results of the study. However, the primary objective of this research was to identify metabolites from the **physiological conditions that exhibit the highest regenerative capacity**. Natural mating represents a physiologically relevant condition that closely mimics the natural reproductive environment, including the hormonal milieu that is essential for proper embryonic development.
While in vitro fertilization (IVF) offers a controlled environment, it may not fully replicate the

complex hormonal and physiological interactions that occur *in vivo*. These interactions are critical for understanding the regenerative potential of embryos in their natural state. By using naturally mated embryos, the study captures the holistic physiological context, which is essential for drawing conclusions about regenerative capacity under normal biological conditions.

Moreover, we acknowledge that IVF could provide additional insights, and future studies may include IVF-derived embryos for comparative analysis. However, for the current investigation, the focus on naturally mated embryos was to ensure that the findings are directly applicable to physiological conditions. This approach aligns with the study's overarching goal of exploring regeneration in a biologically relevant context, rather than in an artificially controlled setting.

2.In the knockdown experiments with siRNA, the authors aim to test their hypothesis in oocytes. However, it is unclear how this approach impacts the maternally deposited proteins, especially considering that the experiments are brief and involve oocytes that either do not divide or only divide once. Clear controls showing the efficiency of protein knockdowns are essential.

Answer to comment 2 of reviewer 1:

We would like to thank the reviewer for the pertinent comments. We acknowledge the limitations of the siRNA approach to study the effects of 5-HT on oocyte development, and the genetic model of conditional knockout is more suitable. Based on the focused idea of the whole paper to prove the effect of 5-HT on cell senescence and its related mechanism, we decided to remove this part of the data about the role of 5-HT and its related tryptophan metabolism pathway in oocyte development, so that we can make the story a more coherent and focused story on 5-HT's rejuvenation effects and mechanisms.

3.On what basis do the authors claim that metabolites found in oocytes could be relevant for rejuvenation or life extension? The transition from discussing oocytes to aging seems somewhat forced. Have the authors demonstrated that me born from eggs with reduced serotonin levels experience shorter lifespans?

Answer to comment 3 of reviewer 1:

The connection between oocyte metabolites to rejuvenation or life extension:

Thank you for your critical insights. Our reasoning is grounded in multiple lines of rationales: **Firstly, oocytes can reset the developmental clock of somatic nuclei during fertilization or somatic cell reprogramming, effectively achieving reprogramming and rejuvenation (there are many conserved mechanisms between cellular reprogramming and rejuvenation).** This remarkable property strongly suggests the presence of factors within oocytes that might promote rejuvenation. Previous studies have shown that the **brief expression of the OSKM reprogramming factors can significantly extend the life span of premature and naturally aged mice**[1], alleviate the level of tissue aging in natural mice[2], and achieve a reversion of the epigenetic clock and metabolic and transcriptomic changes[3], which provides a new idea for delaying aging. However, the

expression level of several pluripotent factors of OSKM in oocytes is very low, which also **means that other substances, such as metabolites, may have reprogramming capacity in oocytes**[4], which aroused our great interest and is one of the rationales for us to explore the metabolites from oocytes that can promote reprogramming/rejuvenation and delay aging.

Secondly, based on previous research on embryo metabolism[5], including our own[6], we demonstrated that early embryos have a more reductive metabolism characteristic and that this reductive state gradually decreases along with development. The earliest stage of early development starts from oocytes. Our metabolomic data also demonstrates that **oocytes exhibit a more reduced metabolic state compared to zygotes or two-cell embryos**. This reduced metabolic state aligns with known characteristics of anti-aging metabolites, such as VC[7] and Taurine[8], further supporting the rationale that oocyte-specific metabolites are likely involved in rejuvenation processes.

Together, these lines of evidence lead to the reasonable hypothesis that oocyte-specific metabolites, such as serotonin (5-HT), may regulate rejuvenation and aging.

The transition from oocytes to aging:

Reduced serotonin levels in oocytes and offspring lifespan:

We acknowledge the important question regarding whether offspring derived from eggs with reduced serotonin (5-HT) levels experience shorter lifespans. However, it is critical to clarify that the primary focus of this study was to investigate the role of 5-HT in delaying aging phenotypes. The study did not extend its scope to examine the long-term effects of reduced oocyte 5-HT levels on the lifespan of offspring. **While the findings demonstrate that 5-HT plays a significant role in mitigating aging-related phenotypes in oocytes, this does not necessarily imply that reduced 5-HT levels in oocytes would directly lead to shorter lifespans in offspring. <u>Lifespan is influenced by a multitude of genetic, epigenetic, and environmental factors that extend far beyond the initial conditions of oocyte quality.</u> Therefore, drawing a direct causal link between oocyte 5-HT levels and offspring lifespan would require additional, targeted investigations that are beyond the current scope of this study.**

While we have not directly demonstrated that offspring born from oocytes with reduced serotonin (5-HT) levels exhibit shorter lifespan, our hypothesis is supported by robust evidence, including our newly added data of 5-HT measurement and spatial metabolomics showing aging is indeed associated with lower level of 5-HT. Specifically:

Serotonin (5-HT) changes during natural aging:

The serotonin levels in the body decrease with age have been reported in a previous literature (Figure 1a as below) [9]. To further demonstrate the potential relationship between oocytes, 5-HT, and aging, **we examined the 5-HT content of MII oocytes from 4 weeks (4W, young) and 12 months (12M, aged) wild-type mice <u>and found that the 5-HT levels of MII from 12 months mice were significantly reduced compared to 4 weeks mice (Figure 1b as below, and Fig. 2e, f in the revised manuscript).</u>**

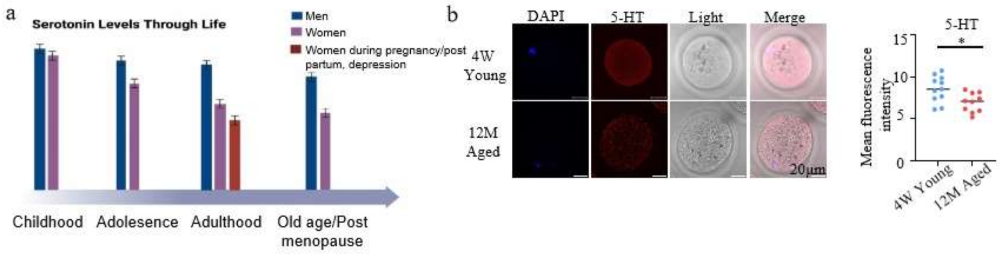

Figure 1

Figure 1: **a,** This infographic illustrates serotonin levels across different life stages using a bar graph[9]. **b,** Immunofluorescence analysis and quantification of 5-HT of 4-week-old C57 and 12-month-old C57 MII oocytes. Scale bar, 20μm. *p<0.05, **p<0.01, ***p<0.001 according to the two-tailed unpaired t-test. n = 10-11 biological repeats.

Spatial metabolomics analysis: We conducted spatial metabolomics profiling of **ovaries** from wild-type mice at 4 weeks, 12 months, and 18 months of age, as well as the brains of 4 weeks and 18 months mice as a control. While 5-HT itself was not detected, likely due to the limitations of non-targeted metabolomics, we observed a clear age-related decline in 5-HT-related pathway metabolites (5-HT is an intermediate metabolite of tryptophan metabolism pathway). These included upstream metabolite tryptophan and downstream metabolites such as 5-Hydroxytryptophol, 5-hydroxykynurenamine (Figure 2b as below). Notably, this decline was particularly pronounced in the ovaries, where it exhibited a gradient decrease of follicular positioning (Location of the red arrow in the figure) along aging. The location of the follicles can be defined by HE staining of adjacent sections (Figure 2a as below).

Broader age-related metabolic changes: In addition to 5-HT-related metabolites, our analysis revealed significant differences in the levels and spatial localization of many other metabolites in the brains and ovaries of mice across different ages (All the detected metabolites and their m/z values, as well as peak areas, are presented in Table S4). For example, Taurine, Vitamin C and GSH, which were known anti-aging metabolites, showed a clear age-related decline in both tissues (Figure 2d as below). These metabolites are also enriched in the oocytes compared with the later-stage embryos (Figure 2c as below and Fig.1d-i in the revised manuscript). The central carbon metabolism (Figure 3a as below), as well as the metabolism of nucleotides, including cAMP and GMP (Figure 3b as below), exhibits a declining trend with age in both the brain and ovaries. Ketone bodies, such as pyruvate and 2-ketobutyric acid (Figure 3c as below), along with certain lipids(Figure 3d as below)—particularly unsaturated fatty acids—show a pronounced age-related decrease in the brain. **These findings suggest that specific metabolites strongly associated with the aging process can serve as reliable biomarkers, and a subset of these aging-associated metabolites can be identified within the unique metabolic profile of oocytes.**

Figure 2 for reviewers removed.

Figure 3 for reviewers removed.

Figure 3: **a,** Central carbon metabolite of the brains and ovaries of 4-month-old, 12-month-old, and 18-month-old mice. The red arrow indicates the follicle. Scale bar, 1 and 3mm. **b,** Nucleotide metabolite of the brains and ovaries of 4-month-old, 12-month-old, and 18-month-old mice. The red arrow indicates the follicle. Scale bar, 1 and 3mm. **c,** Ketogenic metabolite of the brains and ovaries of 4-month-old, 12-month-old, and 18-month-old mice. The red arrow indicates the follicle. Scale bar, 1 and 3 mm. **d,** Lipid metabolite of the brains and ovaries of 4-month-old, 12-month-old, and 18-month-old mice. The red arrow indicates the follicle. Scale bar, 1 and 3 mm.

Together, these findings provide compelling evidence for the involvement of 5-HT-related pathways and broader metabolic shifts in aging. The observed age-related decline in 5-HT-related metabolites, particularly in the oocyte and ovary, underscores the relevance of these pathways to both reproductive biology and systemic aging.

4.The experiments analyzing senescence markers in mesenchymal stem cells (MSCs) are not sufficiently detailed. At what stage were these analyses conducted? Did different MSC cultures undergo similar numbers of cell divisions? What occurs after the fourth day of culture? A more comprehensive cell cycle profile for the various conditions would be helpful. Additionally, the authors claim that lamin B1 levels increase after treatment with 5-HT, but this is not evident in Figure 3h. All panels of Figure 3 are missing a crucial control—wild-type (wt) MSCs and mice treated with 5-HT, which should be included.

Answer to comment 4 of reviewer 1:
We would like to thank the reviewer for the pertinent comments. Our *in vitro* experiments included two models, pathological aging and replicative aging. The established MSCs recapitulated the aging phenotypes[10], such as the increased β-galactosidase positive cells, reduced cell proliferation rate, and upregulated senescence-related gene expression. **G608G MSCs of P9-P11 generations were used as the experimental group in the pathological aging model, and WT MSCs of the same generation were used as controls**. In the replicative aging model, **WT MSCs of P9-P11 generations were used as the experimental group, and P3-P5 WT MSCs differentiated from the same batch were used as the control group**, included in Figure 4 as below (Fig.3 and Extended Fig.3 in the revised manuscript).
The conditions for the passage of all cells were consistent, and cell count determined the number of cells. We further observed differences in cell cycle distribution between WT MSCs and G608G MSCs of the same generation, aged WT MSCs and young WT MSCs, and this difference was ameliorated after 72 hours of 5-HT treatment indicated by the prolonged S phase (Figure 5 as below, and Fig.3i, j in the revised manuscript).

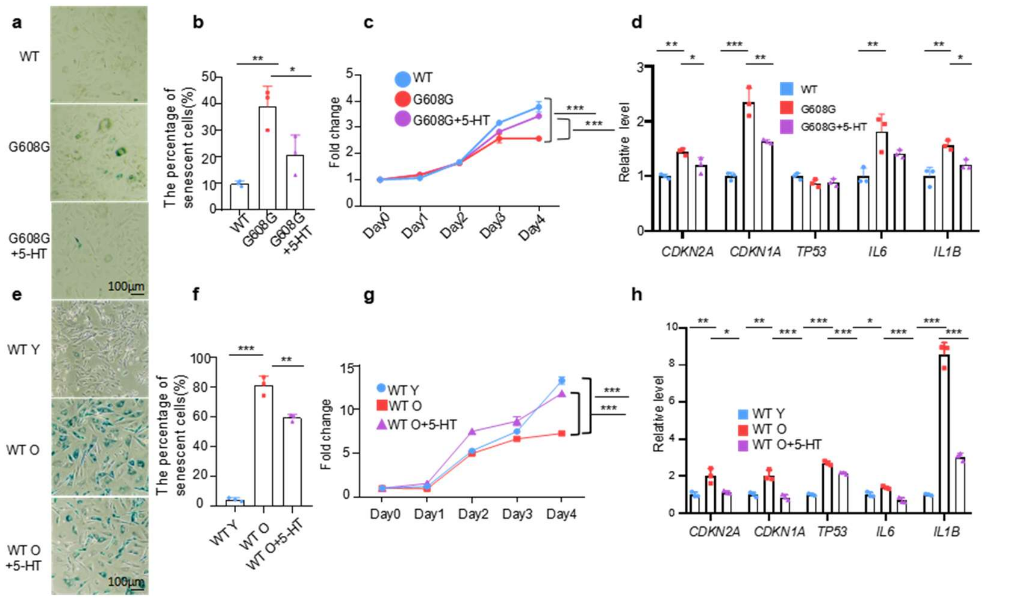

Figure 4

Figure 4: **a**, **b**, SA-β-Gal staining (**a**) and quantification (**b**) of WT MSC and G608G MSC with or without 5-HT treatment. *p<0.05, **p<0.01, ***p<0.001 according to the one-way ANOVA test. n = 3 biological repeats. Scale bar, 100μm. **c**, CCK-8 evaluation of proliferation of WT MSC and G608G MSC with or without 5-HT treatment. *p<0.05, **p<0.01, ***p<0.001 according to the two-way ANOVA test. n = 3 biological repeats. **d**, RT-qPCR analysis of *CDKN2A*, *CDKN1A*, *TP53*, *IL6*, and *IL1B* mRNA expression in WT MSC and G608G MSC with or without 5-HT treatment. *p<0.05, **p<0.01, ***p<0.001 according to the one-way ANOVA test. n = 3 biological repeats. **e**, **f**, SA-β-Gal staining (**e**) and quantification (**f**) of young generations of WT MSC(WT Y), old generations of WT MSC(WT O), and old generations of WT MSC treated with 5-HT(WT O+5-HT, 5μM). *p<0.05, **p<0.01, ***p<0.001 according to the one-way ANOVA test. n = 3 biological repeats. Scale bar, 100μm. **g**, CCK-8 evaluation of the proliferation of WT Y, WT O, and WT O+5-HT. The two-way ANOVA test was used for statistical analysis. n = 3 biological repeats. **h**, RT-qPCR analysis of *CDKN2A*, *CDKN1A*, *TP53*, *IL6*, and *IL1B* mRNA expression of WT Y, WT O, and WT O+5-HT. *p<0.05, **p<0.01, ***p<0.001 according to the one-way ANOVA test. n = 3 biological repeats.

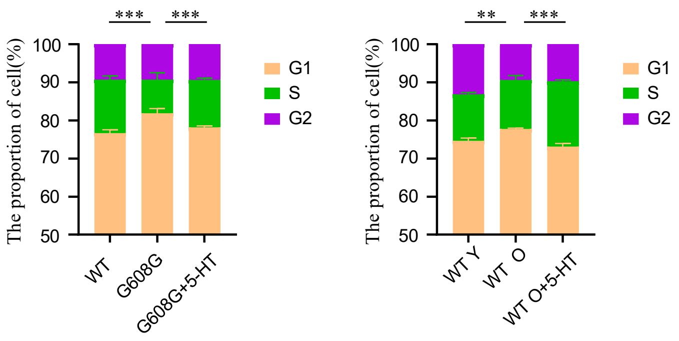

Figure 5

Figure 5: Cell cycle distribution patterns of WT MSCs and G608G MSCs of identical passage number (left) and young and aged WT MSCs (right). Data are the mean ± SD. *p<0.05, **p<0.01, ***p<0.001 according to the two-way ANOVA test. n = 3 biological repeats.

We have obtained relevant data for the *in vivo* experiment of WT mice. **The experimental procedure was that 12-month-old WT C57 mice were given 5-HT orally for 8 months**, and various organ tissues were obtained at 20 months for Masson or HE staining. The relevant results are as follows (Figure 6 as below, Fig.3p-s and Extended Data Fig.3n-q in the revised manuscript), 5-HT can effectively rescue the fibrosis level of multiple tissues of 20M naturally aged mice and the reduction of central veins number in the liver and germinal center area in the spleen. **Therefore, 5-HT can alleviate the aging phenotypes for both wild type and progeria cells,** and using the progeria models can accelerate the experiments. Since the life span of normal WT C57 mice is about 2-3 years, the life span experiment of 5-HT on WT mice requires a 2-3 years period of time, which can be illustrated using the accelerated progeria model. As for the lamin B1 protein level results, we admit that they were not significant enough, and we have removed them according to the reviewer's suggestion.

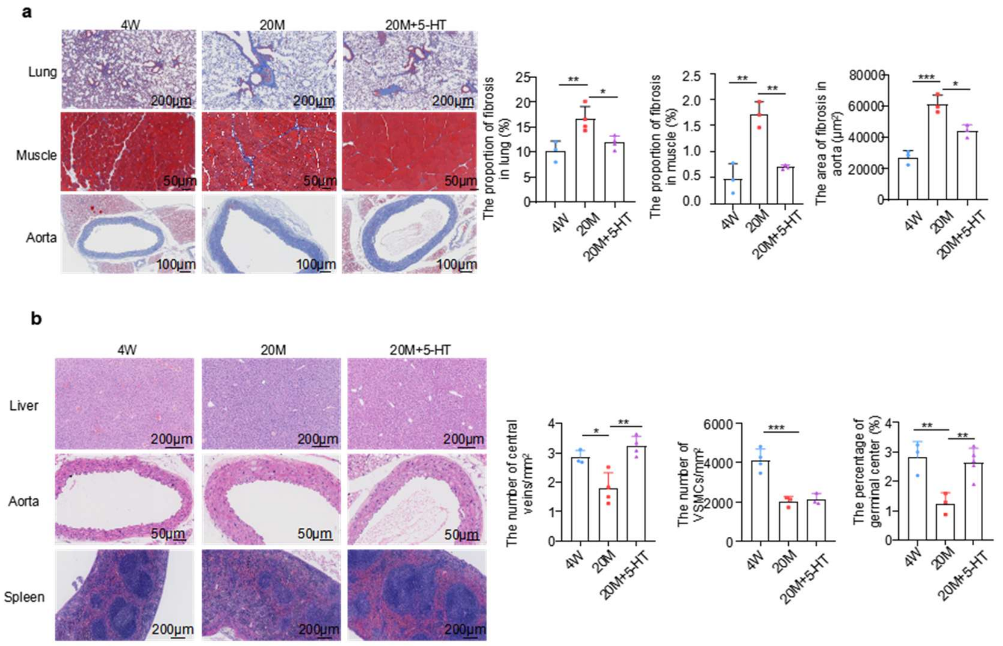

Figure 6

Figure 6: **a,** Masson staining and quantification of fibrosis area of lung, muscle, and aorta in 4-week-old and 20-month-old wild type mice treated with 5-HT or not (from 12-month-old to 20-month-old). *p<0.05, **p<0.01, ***p<0.001 according to the one-way ANOVA test. n = 3-4 biological repeats. Scale bar, 200, 50 and 100μm. **b**, Histological analysis (HE) of the liver, spleen, and aorta of 4-week-old and 20-month-old C57 mice treated with 5-HT or not (from 12-month-old to 20-month-old); Quantitative analysis of the number of central veins per unit area (mm^2) in the liver, the average VSMC number per unit area (mm^2) in the aorta, and the percentage of germinal center area in the spleen. *p<0.05, **p<0.01,

***p<0.001 according to the one-way ANOVA test. n = 3-4 biological repeats. Scale bar, 200 and 50 μm.

5.The statement "However, these studies all focused on the role in neuronal cells and mechanisms of brain aging and cognitive decline; however, the systemic role of 5-HT in contributing to whole-body cellular senescence or rejuvenation, especially in non-neuronal cells, is unclear" is inaccurate. Several studies already highlight serotonin's role in immune cell regulation.

Answer comment 5 to reviewer 1:
We appreciate the reviewer's comments and acknowledge that several studies have explored serotonin's (5-HT) regulatory roles in immune cells. Our initial statement was intended to emphasize the lack of comprehensive studies examining 5-HT's systemic effects on whole-body cellular senescence and rejuvenation beyond the nervous and immune systems. While 5-HT's influence on immune regulation is well-documented, its role in a broad range of non-neuronal and non-immune cells, remains relatively unexplored. Additionally, our study focuses on the post-translational modification by intracellular 5-HT in all cell types that contain 5-HT, whereas the 5-HT regulation in immune cells lies in extracellular 5-HT engaging on 5-HT and is dependent on the expression of 5-HT receptors on the target cells.
To clarify, we have revised our statement to reflect that serotonin has established roles in immune regulation, yet its impact on aging and rejuvenation mechanisms in a systemic context, particularly in non-immune cells, remains an open question. The revised version on pages 7-8 is as follows:
While previous literature has reported 5-HT as an immune function modulator, our *in vivo* transcriptome level analyses revealed tissue-specific and inconsistent alterations in pro-inflammatory cytokine expression (*Il1b*, *Il6*, *Il12*) across hepatic and kidney tissues following chronic 5-HT administration. This heterogeneous inflammatory response aligns with 5-HT's bidirectional regulatory capacity in inflammation, where context-dependent effects have been previously reported. In striking contrast, we observed robust and consistent downregulation of senescence-associated genes (*Cdkn2a* and *Cdkn1a*) in both hepatic and kidney tissues, suggesting that 5-HT's lifespan-extending effects *in vivo* are unlikely to be predominantly mediated through immunomodulatory pathways (Extended Data Fig.4a-d in the revised manuscript**). These data collectively provide compelling evidence that 5-HT administration effectively attenuates aging-associated phenotypes *in vivo*, pointing to a more comprehensive physiological role for serotonin in counteracting organismal aging.**

7. The authors misuse the terms "rejuvenation" and "anti-aging" throughout the manuscript, as no such experiments are presented. The data only show that serotonin supplementation affects the fitness of aging-prone, genetically modified mice. Furthermore, neither of these terms should be used in the context of in vitro cell cultures, and these should be corrected.

Answer to comment 6 of reviewer 1:

We appreciate your feedback and acknowledge the importance of precise terminology.

Terminology in aging alleviation studies

We have carefully investigated the terminology in the aging alleviation studies in literature. “Anti-aging” often refers to organismal/organ-level physiological improvements[11,12]. “Rejuvenation” is widely accepted in cell biology literature to describe cellular phenotype reversal, e.g., restored mitochondrial function, reduced senescence markers, including in vitro cultured cells [13,14].

Our experiments on aging alleviation:

We established two cell models of pathological aging and replicative aging (a common model for natural aging). The former used WT MSCs as the control while using G608G MSCs of the same passage as the experimental group, and the latter used WT MSCs of the younger passage (P3-P5) as the control while using older WT MSCs (P9-P11) as the experimental group. The established MSCs recapitulated the universal aging phenotypes [10,15]. Our results indicated that 5-HT treatment decreased β-galactosidase expression and increased cell proliferation of MSCs in pathological aging and replicative aging models (Figure 7 as below and Fig.3 in the revised manuscript). The mRNA levels of cellular senescence marker genes were reduced in the treated group compared with the control (Figure 7 as below and Fig.3 in the revised manuscript). These experiments are consistent with the conventional characteristics for rejuvenation studies.

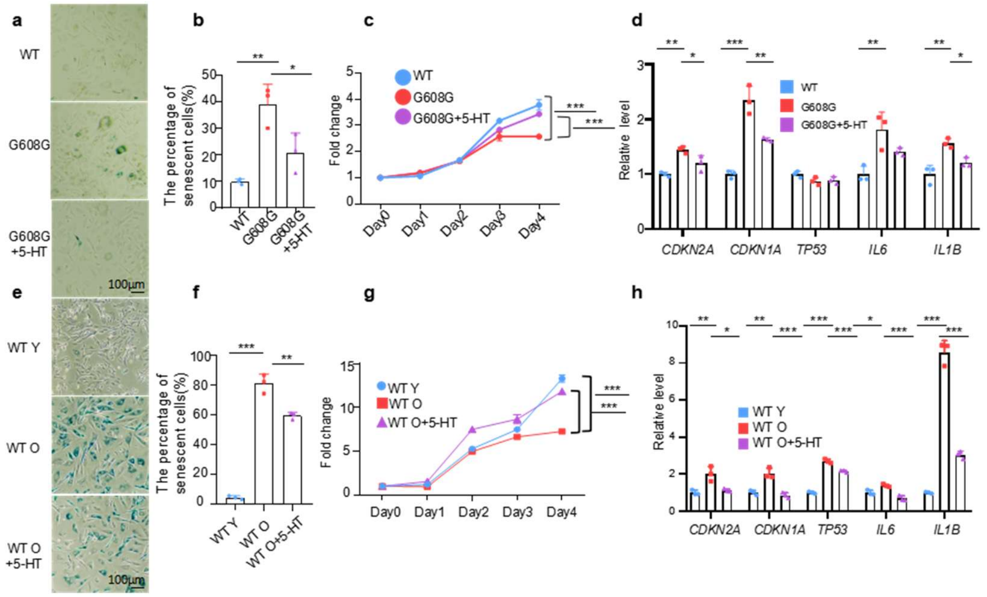

Figure 7

Figure 7: **a**, **b**, SA-β-Gal staining (**a**) and quantification (**b**) of WT MSC and G608G MSC with or without 5-HT treatment. *p<0.05, **p<0.01, ***p<0.001 according to the one-way ANOVA test. n = 3 biological repeats. Scale bar, 100μm. **c**, CCK-8 evaluation of proliferation of WT MSC and G608G MSC with or without 5-HT treatment. *p<0.05, **p<0.01, ***p<0.001 according to the two-way ANOVA test. n = 3 biological repeats. **d**, RT-qPCR analysis of *CDKN2A*, *CDKN1A*, *TP53*, *IL6*, and *IL1B* mRNA expression in WT

MSC and G608G MSC with or without 5-HT treatment. *p<0.05, **p<0.01, ***p<0.001 according to the one-way ANOVA test. n = 3 biological repeats. **e**, **f**, SA-β-Gal staining (**e**) and quantification (**f**) of young generations of WT MSC(WT Y), old generations of WT MSC(WT O), and old generations of WT MSC treated with 5-HT(WT O+5-HT, 5μM). *p<0.05, **p<0.01, ***p<0.001 according to the one-way ANOVA test. n = 3 biological repeats. Scale bar, 100μm. **g**, CCK-8 evaluation of the proliferation of WT Y, WT O, and WT O+5-HT. The two-way ANOVA test was used for statistical analysis. n = 3 biological repeats. **h**, RT-qPCR analysis of *CDKN2A*, *CDKN1A*, *TP53*, *IL6*, and *IL1B* mRNA expression of WT Y, WT O, and WT O+5-HT. *p<0.05, **p<0.01, ***p<0.001 according to the one-way ANOVA test. n = 3 biological repeats.

Apart from the *in vitro* cell experiments and the relevant experiments on using 5 - HT to delay aging in G608G mutant mice, we also conducted *in vivo* experiments on **naturally aged mice**. The experimental protocol involved orally administering 5-HT to **12-month-old wild-type (WT) C57 mice** for 8 months. Subsequently, various organ tissues were harvested at the age of 20 months for Masson or hematoxylin and eosin (HE) staining. The corresponding results are presented as follows (Figure 6 as above, Fig.3p-s and Extended Data Fig.3n-q in the revised manuscript). 5 - HT can effectively mitigate the fibrosis levels in multiple tissues of 20-month-old naturally aged mice. Moreover, it can reverse the reduction in the number of central veins in the liver and the shrinkage of the germinal center area in the spleen. In conclusion, our study demonstrates that serotonin, through the serotonylation of HSP90β, delays cellular senescence and alleviates tissue aging phenotypes in mice by mitigating ER stress and promoting mitochondria regeneration. While these findings are consistent with rejuvenation-associated processes, we agree to refine the terminology to reflect the scope of our results better and avoid overstating their implications. Thank you for your constructive feedback.

7.The in vivo experiments with orally administered 5-HT are unsurprising, given that serotonin has been previously shown to have anti-inflammatory effects. If the authors propose a novel mechanism behind the lifespan extension observed in G608G mice, they need to exclude any influence of 5-HT on the immune system. Additionally, how does the proposed mechanism explain the observed phenotypes in vivo? Does treatment with an HSP90 inhibitor and a selective 5-HTR1B agonist produce similar effects? Can an inhibitor of TGM2 or a 5-HTR1B antagonist reverse the effects of 5-HT in vivo in G608G mice?

Answer to comment 7 of reviewer 1:

We appreciate the reviewer's insightful comments regarding the role of serotonin (5-HT) in inflammation and its potential impact on the immune system. Below, we address these concerns and provide evidence supporting our findings.

Specific findings in our study are distinct from immune cells and cannot be explained by immune cells:

Our study focuses on a novel mechanism through which 5-HT extends lifespan in G608G mice, primarily mediated by HSP90β serotonylation and its downstream effects on

proteostasis and endoplasmic reticulum (ER) stress. **While 5-HT is known to influence the immune system, our data suggests that its effects in this model are not primarily driven by immune modulation because of the following evidences**. Specifically:

1. Inflammatory cytokine levels included *Il1b*, *Il6*, and *Il12* in the tissues of mice subjected to long-term oral 5-HT administration exhibited varying and inconsistent minor changes, with some showing an increase while others showed a decrease, or without a consistent significant difference across tissues (Figure 8 as below, and Extended Data Fig.4a, b in the revised manuscript). This observation aligns with the previously mentioned bidirectional regulatory role of 5-HT in inflammation[16-18]. However, aging-related genes *Cdkn2a* and *Cdkn1a* displayed a consistent and significant downward trend in both the kidney and liver (Figure 9 as below and Extended Data Fig.4c, d in the revised manuscript), indicating that the lifespan extension effects are unlikely to be solely immune cell-mediated.
2. The serotonylation of HSP90β is a non-immune mechanism that mitigates persistent ER stress, a key hallmark of cellular aging, and does not directly involve immune pathways. And we can demonstrate that HSP90β serotonylation also occurs *in vivo* in non-immune cells (Figure 10 as below, and Extended Data Fig.6e, f in the revised manuscript).

Conclusion:

Our study identifies a novel mechanism of action for 5-HT in aging, involving HSP90β serotonylation and reduced ER stress. While serotonin's role in inflammation is documented, our findings provide strong evidence that its lifespan extension effects in this model are mediated through non-immune pathways. To address these concerns, we have clarified this distinction in the revised manuscript in pages 7-8 as the following "While previous literature has established 5-HT as a potent modulator of immune function, our in vivo transcriptome level analyses revealed tissue-specific and inconsistent alterations in pro-inflammatory cytokine expression (*Il1b*, *Il6*, *Il12*) across hepatic and kidney tissues following chronic 5-HT administration. This heterogeneous inflammatory response aligns with 5-HT's well-documented bidirectional regulatory capacity in inflammation30-32, where context-dependent effects have been previously reported. In striking contrast, we observed robust and consistent downregulation of senescence-associated genes (*Cdkn2a* and *Cdkn1a*) in both hepatic and kidney tissues, suggesting that 5-HT's lifespan-extending effects in vivo are unlikely to be predominantly mediated through immunomodulatory pathways (Figure 8, 9 as below, Extended Data Fig.4a-d in the revised manuscript). These data collectively provide compelling evidence that 5-HT administration effectively attenuates aging-associated phenotypes in vivo, pointing to a more comprehensive physiological role for serotonin in counteracting aging at the organismal level.."

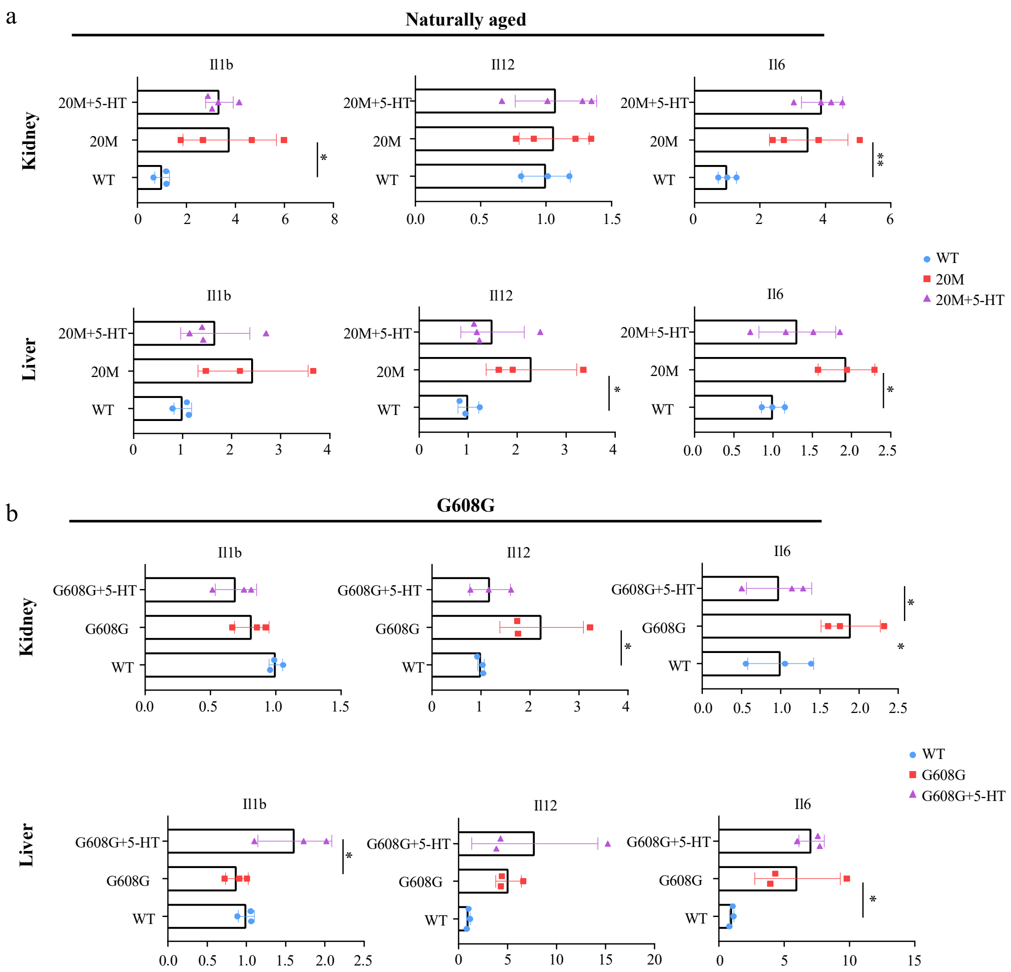

Figure 8

Figure 8: **a, b,** RT-qPCR analysis of *Il1b*, *Il6*, and *Il12* mRNA expression of liver and kidney in naturally aged and G608G mice treated with 5-HT or not. *p<0.05, **p<0.01, ***p<0.001 according to the one-way ANOVA test. n = 3-4 biological repeats.

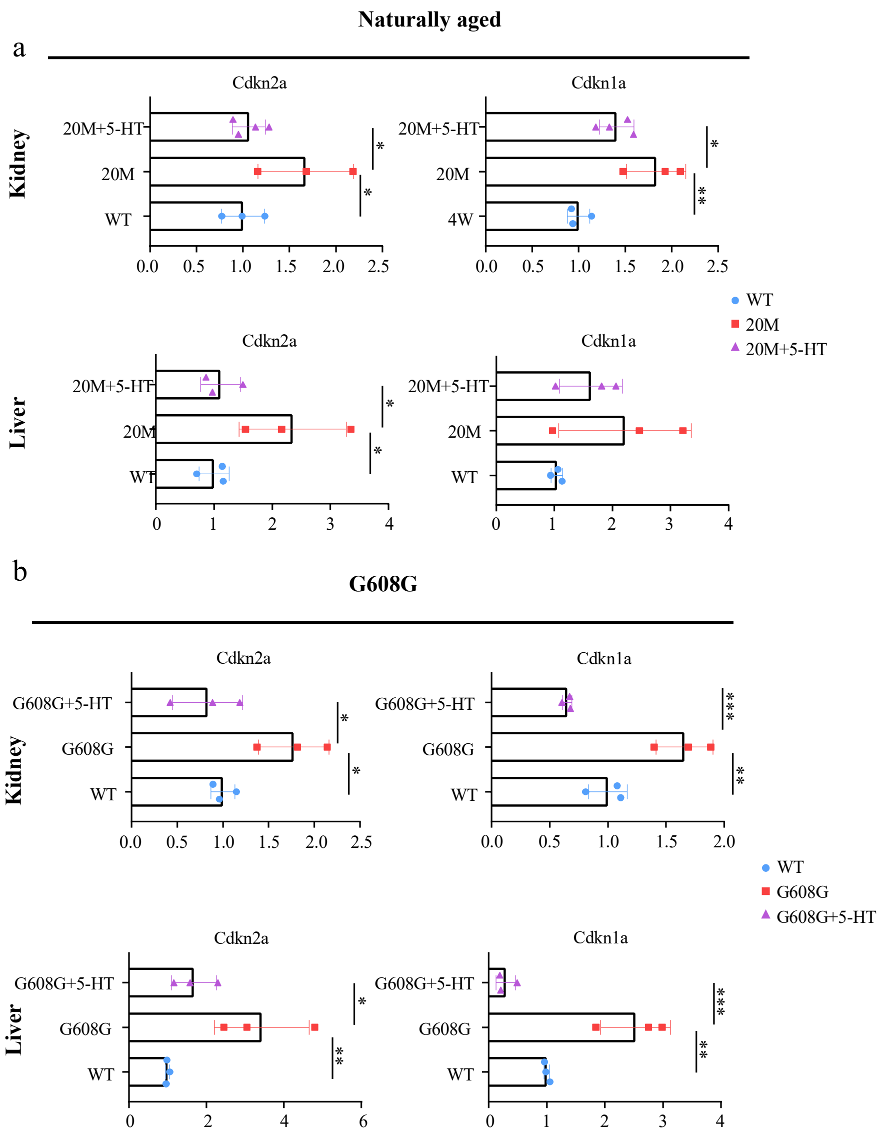

Figure 9

Figure 9: **a, b,** RT-qPCR analysis of *Cdkn2a* and *Cdkn1a* mRNA expression of liver and kidney in naturally aged and G608G mice treated with 5-HT or not. *p<0.05, **p<0.01, ***p<0.001 according to the one-way ANOVA test. n = 3-4 biological repeats.

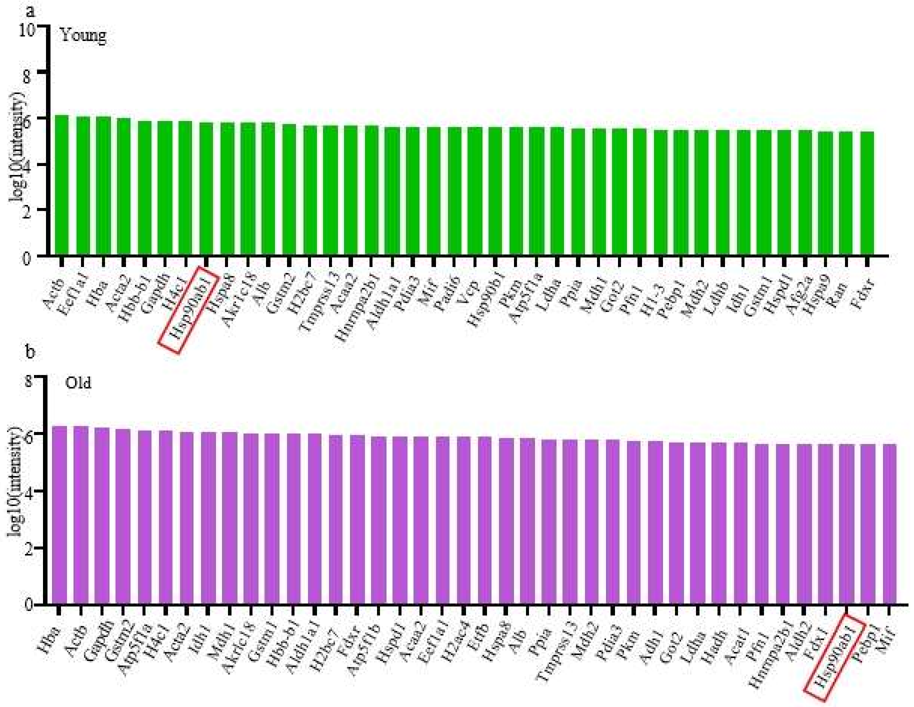

Figure 10

Figure 10: Heatmap of the top 40 enriched proteins of the ovaries 4-week-old (Young) and 12-month-old mice (Old).

Regarding HSP90 inhibitors and 5-HTR1B agonists:

HSP90 inhibitors have demonstrated significant anti-aging properties as reported by Luo et al[19], where inhibition of HSP90 activated the heat shock response and reduced protein aggregation. In particular, Georges E. Janssens et al. identified two HSP90 inhibitors, monorden and tanespimycin, which not only extended lifespan but also improved health in C. elegans (Figure 11a as below)[20]. Furthermore, the HSP90 inhibitor alvespimycin has been shown to effectively reduce kidney senescence and tubulointerstitial fibrosis in a mouse model of diabetic kidney disease, highlighting the potential of HSP90 inhibitors in combating age-related conditions(Figure 11b as below)[21].

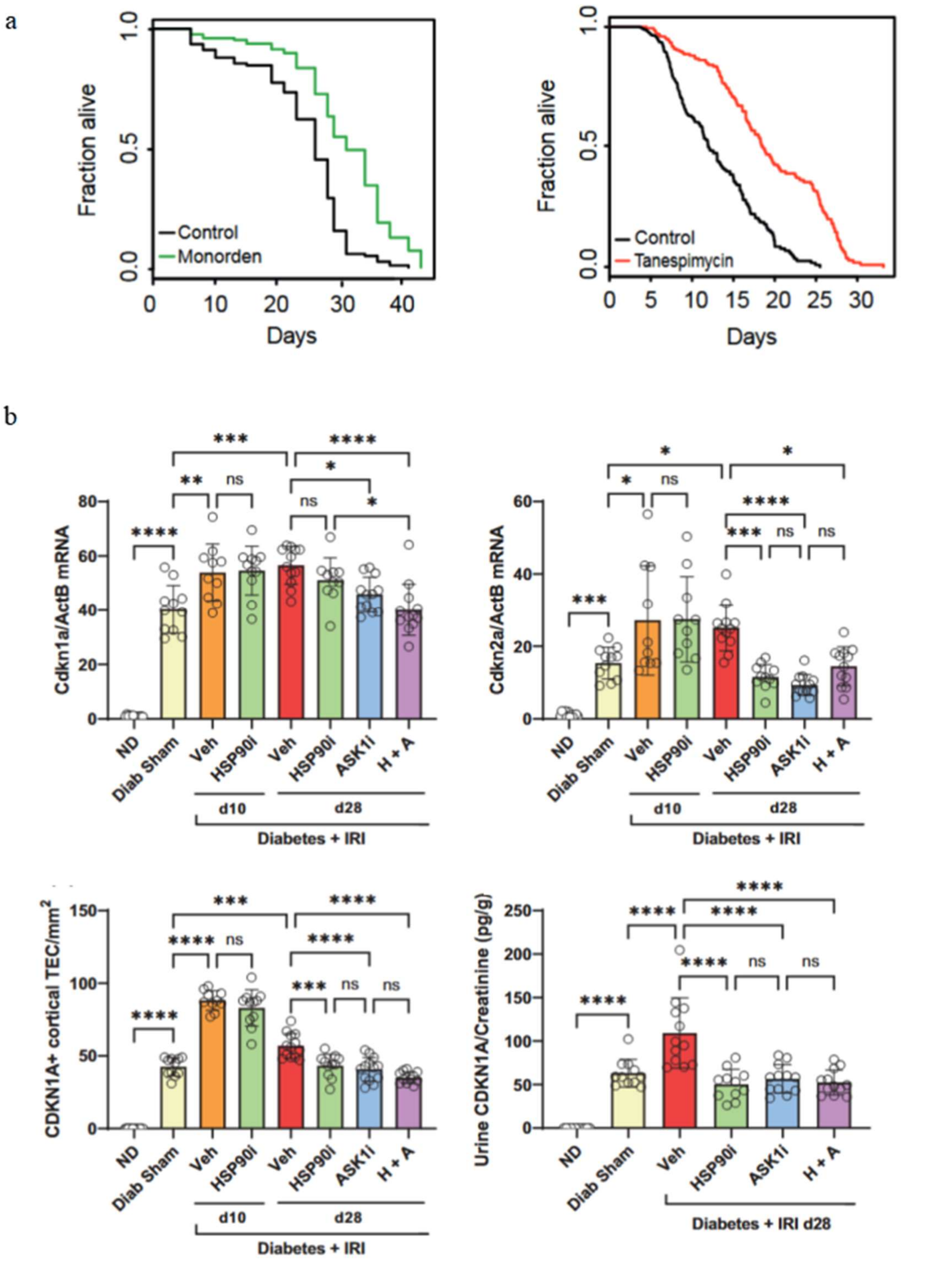

Figure 11

Figure 11: **a,** The survival curve of monorden-treated or tanespimycin-treated worms, generated using the manual scoring method of prodding with a platinum wire. **b,** Analysis of diabetic mice at days 10 and 28 after IRI identified the impact of alvespimycin (HSP90i, green bars), GS-444217 (ASK1i, blue bars), and combined therapy (H+A, purple bars) on kidney gene expression of *Cdkn1a* and *Cdkn2a*, immunostaining of *CDKN1A*+ tubular cells, and urine excretion of *CDKN1A*. Non-diabetic mice (ND, white bars), sham-operated

diabetic mice (Diab Sham, yellow bars) and vehicle-treated mice with diabetes and IRI (Veh, red bars) were used as controls. Data = individual data points with mean ± SD; n=10–12; ****p<0.0001, ***p<0.001, **p<0.01, *p<0.05; ns = non-significant.

Regarding 5-HTR1B, the 2008 study by Savelieva et al. provides compelling evidence that 5-HTR1B plays a protective role in aging. Their key findings demonstrate that 5-HTR1B knockout mice exhibit (1) significantly shortened lifespan (15% reduction) (Figure 12a as below), and (2) early-onset motor deficits (Figure 12b as below). These results collectively establish 5-HTR1B signaling as a critical regulator of healthy aging processes in mice[22].

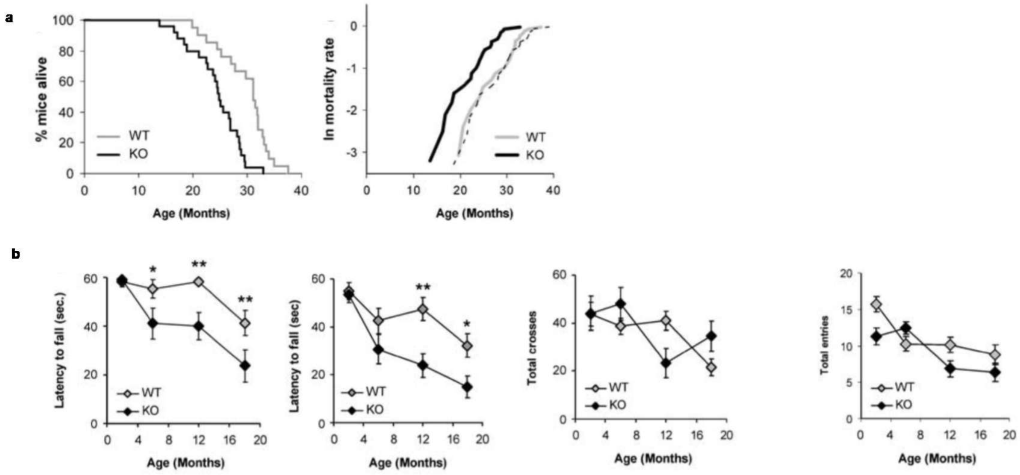

Figure 12

Figure 12: **a,** left: Kaplan–Meier survival curves revealed a significant decrease in longevity in Htr1bKO mice ($p < 0.0001$). Right: Mortality curves: Htr1bKO mice displayed a 3.75-fold increased hazard ratio ($p < 0.0005$). Hatched curve represents Htr1bKO mortality curve superimposed on the WT curve (WT, n = 21; KO, n = 24). **b,** 5Htr1bKO mice displayed early age-related motor impairment in the rotarod and coat hanger tests. Post hoc tests: *$p < 0.05$, **$p < 0.005$. Total activity decreased with age in WT and Htr1bKO mice in the open field and EPM test.

These findings collectively confirmed the roles of HSP90 and 5-HTR1B in promoting cellular resilience and attenuating aging-associated deterioration.

While no existing literature has directly tested TGM2 inhibitors or 5-HTR1B antagonists in G608G mice, our *in vitro* cellular experiments provide reasonable grounds to hypothesize that these interventions could reverse 5-HT's effects *in vivo*. Notably, these small molecules often have more than one target, and the consequences accumulated to reflect lifespan might be complicated. Nevertheless, we have discussed these relevant small molecules in the discussion as follows in the revised manuscript "Page 12".

8.Since the analysis in Figures 5 and 6 compares MSCs in varying senescent states (greater senescence in G608G, less senescence in others), it is crucial to confirm that the observed differences are directly caused by 5-HT treatment and not simply due to varying cellular states. Does 5-HT treatment produce similar effects on replicative or other types of senescence in normal cell cultures?

Answer to comment 8 of reviewer 1:

We appreciate the reviewer's concern about distinguishing between treatment effects and baseline cellular differences. We would like to clarify that our experimental approach carefully addresses this important distinction.

For young WT MSCs, as well as old WT MSCs and G608G MSCs of the same generation, we simultaneously conducted 5-HT treatment. The results demonstrated that 5-HT effectively delayed cellular senescence phenotypes in both replicative (normal) aging and pathological aging models, including the reduction of *CDKN1A*, *CDKN2A* expression and decreased proportion of β-gal positive cells. However, its effects were not significant in cells that were already young (Figure 13 as below). Therefore, in subsequent experiments, we conducted exploration and verification separately in these two models and our study employed consistent cell models throughout all experiments. **G608G MSCs of P9-P11 generations were used as the experimental group in the pathological aging model, and WT MSCs of the same generation were used as controls**. In the replicative aging model, **WT MSCs of P9-P11 generations were used as the experimental group, and P3-P5 WT MSCs differentiated from the same batch were used as the control group.** Originally, we placed the results of pathological aging in the main figure and the results of replicative aging (mimicking natural aging) in the supplementary figure. Now, we have reformatted most of the content to present the results of both aging models in the main figure as much as possible, as shown in Figure 14-17 as below, and Fig.3, Fig.5, Fig.6 and Extended Data Fig.8 in the revised manuscript.

We have indeed demonstrated that 5-HT produces similar anti-senescence effects in normal cells undergoing replicative senescence, as shown in Figure 14 as below. These data demonstrate that 5-HT treatment attenuates multiple hallmarks of cellular senescence in normal MSCs, including reduced SA-β-gal activity, decreased expression of *CDKN2A, CDKN1A,* and *TP53*, and improved proliferative capacity, accompanied by an increased proportion of senescent cells in the S phase of the cell cycle. Similarly, our findings demonstrate that serotonin (5-HT)-mediated serotonylation of HSP90β occurs in both pathological aging models (G608G MSCs) and replicative senescence models of normal cells (WT MSCs) (Figure 15 as below). The inhibition of LDN significantly restricts the anti-aging efficacy of 5-HT (Figure 15 as below). Furthermore, HSP90 overexpression exacerbates cellular senescence in both cell types, while supplementation with 5-HT effectively rescues this senescent phenotype (Figure 16 as below). Conversely, HSP90β knockdown promotes cellular rejuvenation (Figure 17 as below).

The parallel responses observed in both our replicative senescence and pathological senescence models provide compelling evidence for a consistent aging alleviation mechanism of 5-HT that transcends specific senescence induction methods, thereby strengthening the overall conclusions of our study.

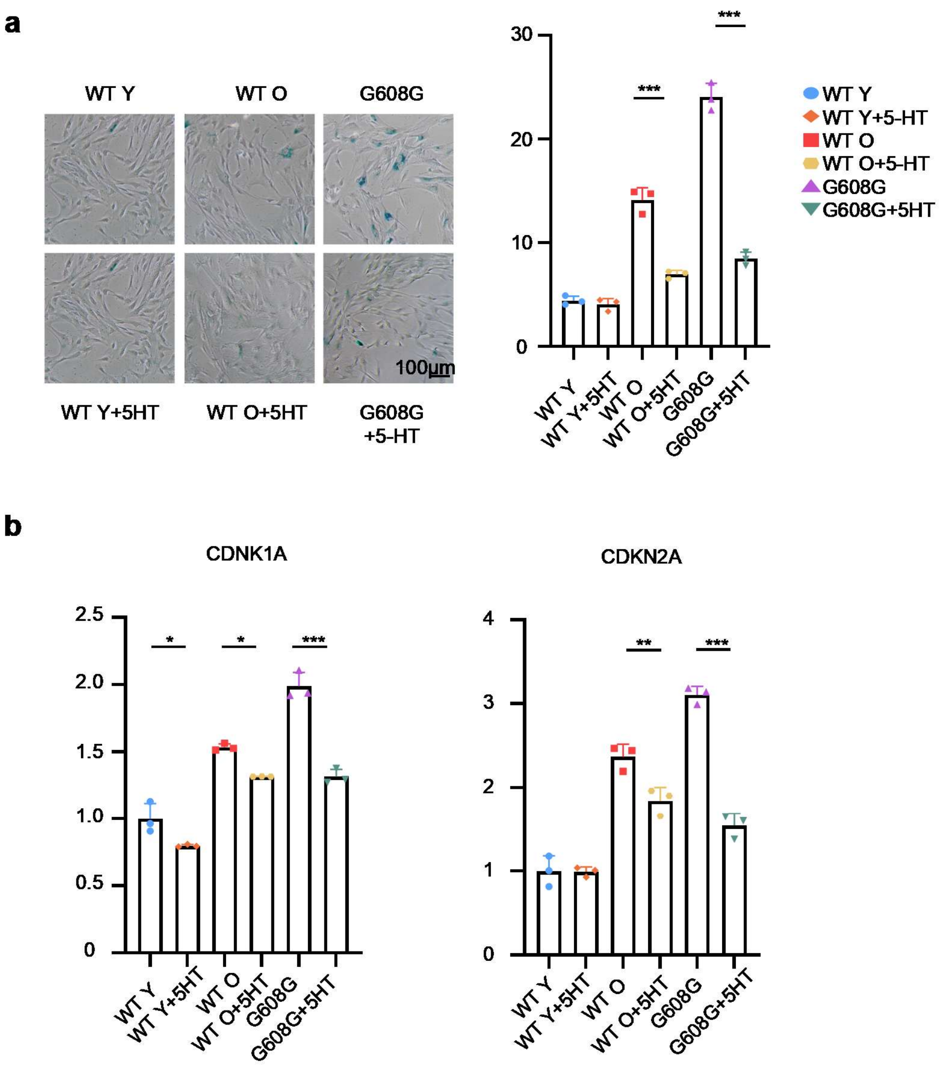

Figure 13

Figure 13: **a**,β-Gal staining (left) and quantification (right) of WT Y, WT O and G608G MSCs treated with 5-HT or not. *p<0.05, **p<0.01, ***p<0.001 according to the one-way ANOVA test. n = 3 biological repeats. Scale bar, 100 μm. **b**, RT-qPCR analysis of *CDKN2A*, *CDKN1A*, mRNA expression of WT Y, WT O and G608G MSCs treated with 5-HT or not. *p<0.05, **p<0.01, ***p<0.001 according to the one-way ANOVA test. n = 3 biological repeats

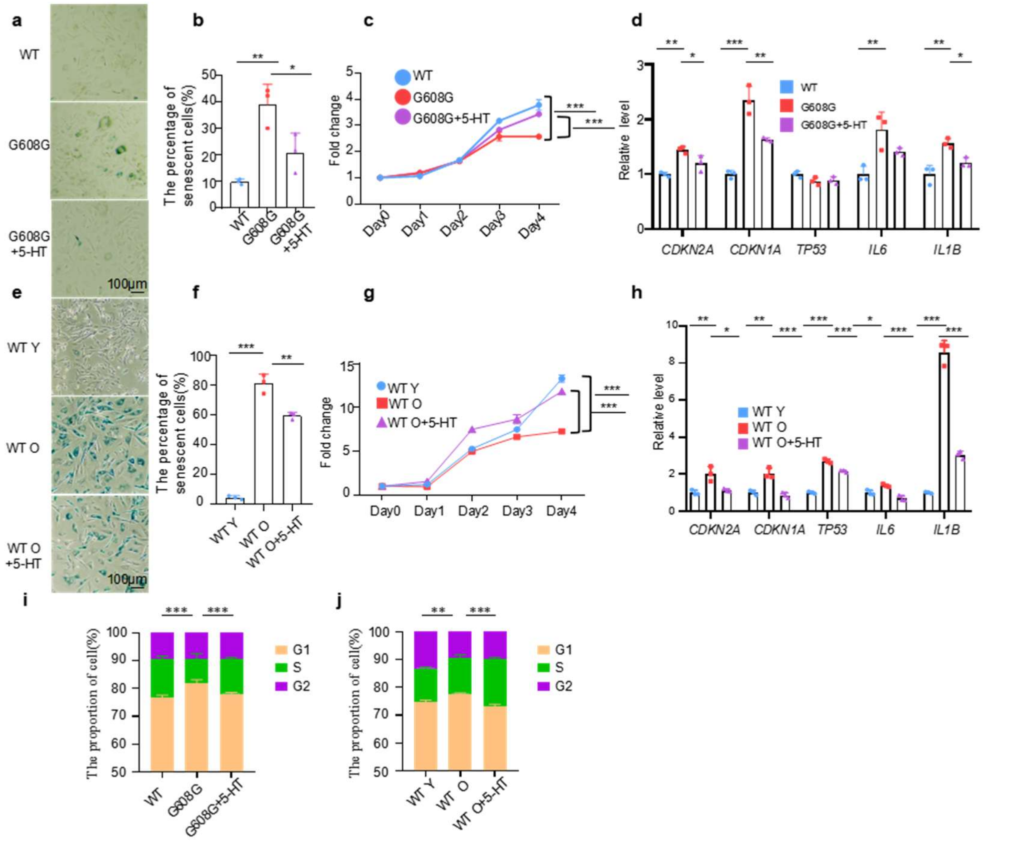

Figure 14

Figure 14: **a**, **b**, SA-β-Gal staining (**a**) and quantification (**b**) of WT MSC and G608G MSC with or without 5-HT treatment. *p<0.05, **p<0.01, ***p<0.001 according to the one-way ANOVA test. n = 3 biological repeats. Scale bar, 100μm. **c**, CCK-8 evaluation of proliferation of WT MSC and G608G MSC with or without 5-HT treatment. *p<0.05, **p<0.01, ***p<0.001 according to the two-way ANOVA test. n = 3 biological repeats. **d**, RT-qPCR analysis of *CDKN2A*, *CDKN1A*, *TP53*, *IL6*, and *IL1B* mRNA expression in WT MSC and G608G MSC with or without 5-HT treatment. *p<0.05, **p<0.01, ***p<0.001 according to the one-way ANOVA test. n = 3 biological repeats. **e**, **f**, SA-β-Gal staining (**e**) and quantification (**f**) of young generations of WT MSC(WT Y), old generations of WT MSC(WT O), and old generations of WT MSC treated with 5-HT(WT O+5-HT, 5μM). *p<0.05, **p<0.01, ***p<0.001 according to the one-way ANOVA test. n = 3 biological repeats. Scale bar, 100μm. **g**, CCK-8 evaluation of the proliferation of WT Y, WT O, and WT O+5-HT. The two-way ANOVA test was used for statistical analysis. n = 3 biological repeats. **h**, RT-qPCR analysis of *CDKN2A*, *CDKN1A*, *TP53*, *IL6*, and *IL1B* mRNA expression of WT Y, WT O, and WT O+5-HT. *p<0.05, **p<0.01, ***p<0.001 according to the one-way ANOVA test. n = 3 biological repeats. **i**,**j**, Cell cycle distribution patterns of WT MSCs and G608G MSCs of identical passage number (**i**) and young and aged WT MSCs (**j**). *p<0.05, **p<0.01, ***p<0.001 according to the two-way ANOVA test. n = 3 biological repeats.

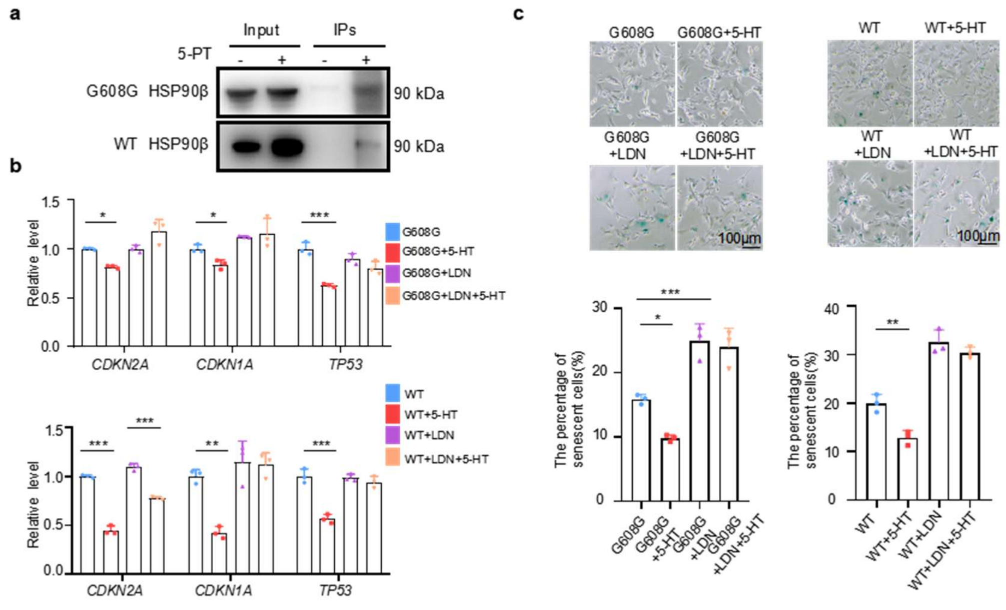

Figure 15

Figure 15: **a**, MSCs were cultured for 72 h, with or without 5 μmol/L 5-PT. 5-PT was immunoprecipitated by beads, and HSP90β was detected. IP, immunoprecipitation. Upper: G608G MSCs, Lower: WT MSCs. **b**, RT-qPCR analysis of *CDKN2A*, *CDKN1A*, and *TP53* mRNA expression in MSCs treated with 5-HT, LDN or LDN+5-HT, LDN: LDN-27219, TGM2 inhibitor. Upper: G608G MSCs, Lower: WT MSCs. *p<0.05, **p<0.01, ***p<0.001 according to the one-way ANOVA test. n = 3 biological repeats. **c**, SA-β-Gal staining (upper) and quantification (lower) of MSCs treated with LDN or LDN+5-HT. Left: G608G MSCs, Right: WT MSCs. *p<0.05, **p<0.01, ***p<0.001 according to the one-way ANOVA test. n = 3 biological repeats. Scale bar, 100 μm.

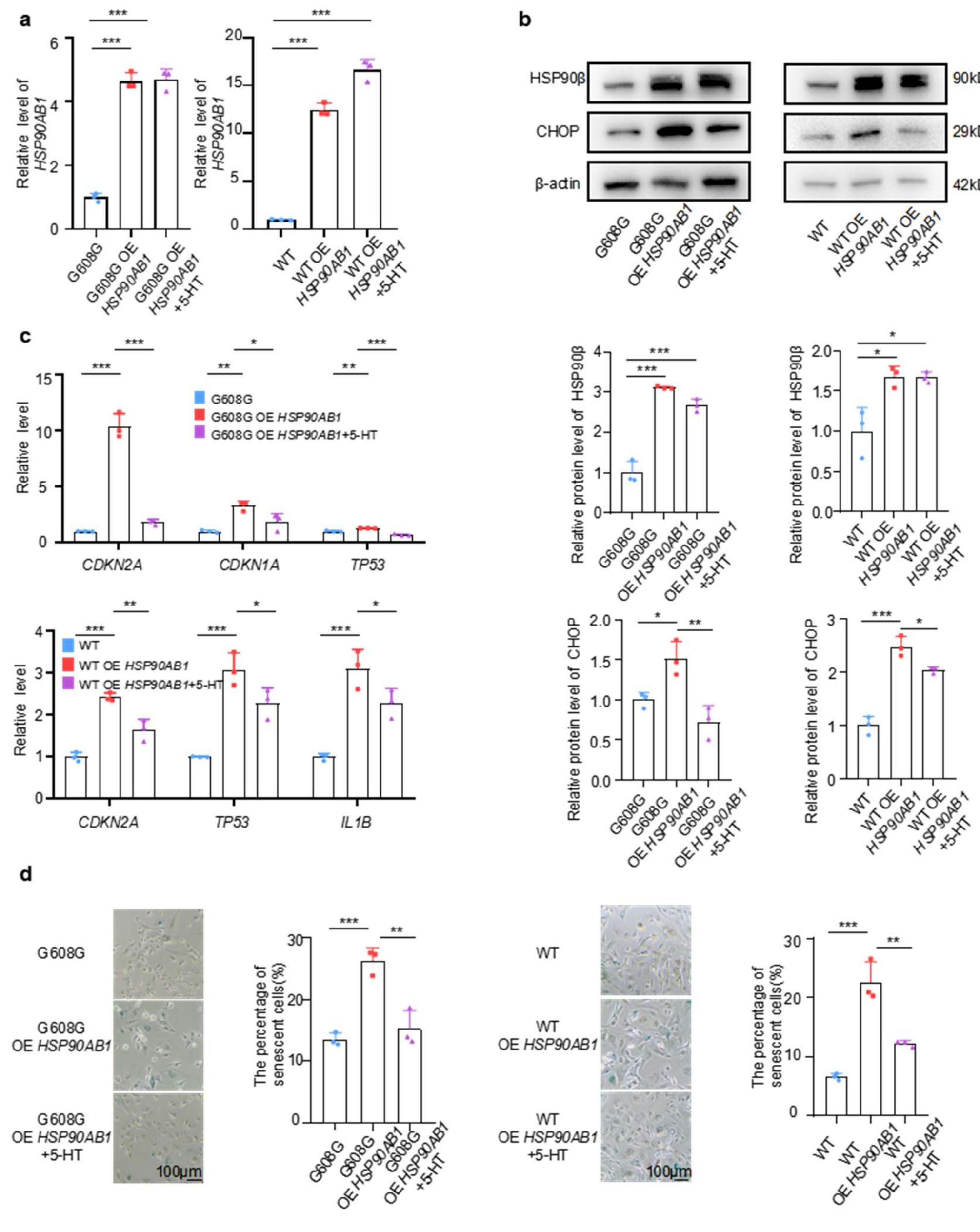

Figure 16

Figure 16: **a**, RT-qPCR analysis of *HSP90AB1* mRNA expression in MSCs and *HSP90AB1*-overexpression MSCs with or without 5-HT treatment. Left: G608G MSCs, Right: WT MSCs. *p<0.05, **p<0.01, ***p<0.001 according to the one-way ANOVA test. n = 3 biological repeats. **b**, Western blots of HSP90β, CHOP protein levels in MSCs and *HSP90AB1*-overexpressing MSCs with or without 5-HT treatment. Left: G608G MSCs, Right: WT MSCs. Lower: Quantification of the protein levels relative to β-actin. Data are presented as mean ± SD. *p<0.05, **p<0.01, ***p<0.001 according to the one-way ANOVA test. **c**, RT-qPCR analysis of *CDKN2A*, *TP53*, and *IL1B* or *CDKN1A* mRNA expression in MSCs and *HSP90AB1*-overexpression MSCs with or without 5-HT treatment. Upper: G608G MSCs, Lower: WT MSCs. *p<0.05, **p<0.01, ***p<0.001 according to the one-way

ANOVA test. n = 3 biological repeats. **d**, SA-β-Gal staining(left) and quantification(right) of MSCs and *HSP90AB1*-overexpression MSCs with or without 5-HT treatment. Left: G608G MSCs, Right: WT MSCs. *p<0.05, **p<0.01, ***p<0.001 according to the one-way ANOVA test. n = 3 biological repeats. Scale bar, 100 μm.

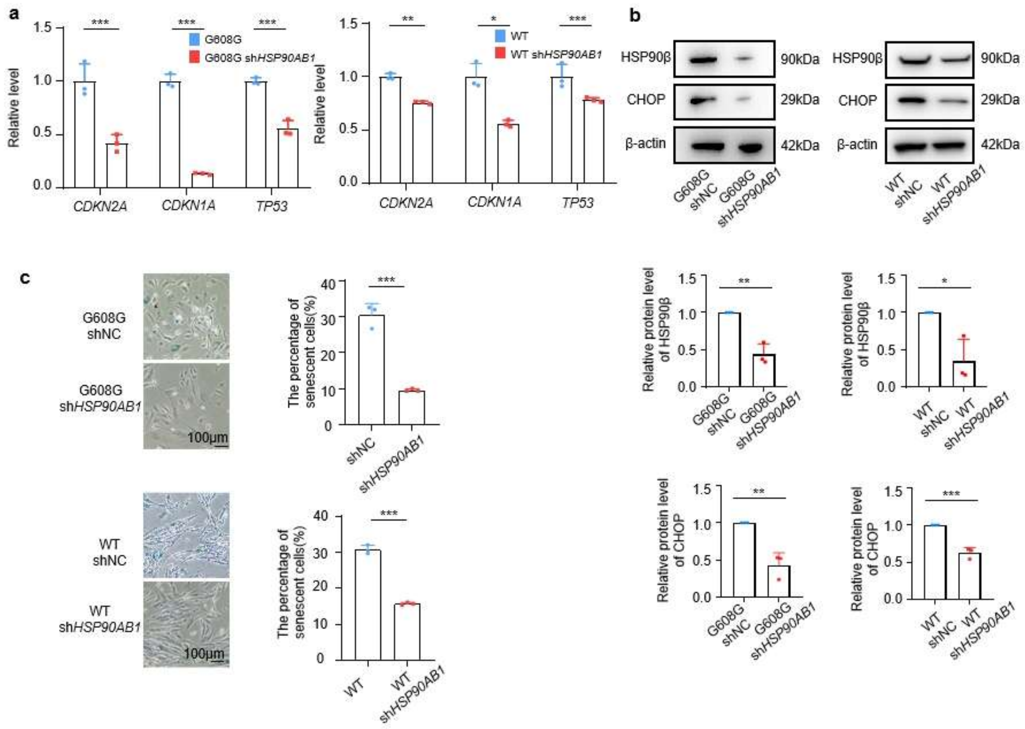

Figure 17

Figure 17: **a**, RT-qPCR analysis of *CDKN2A*, *CDKN1A*, and *TP53* mRNA expression in MSCs transfected with control shRNA or shRNA against *HSP90AB1*. Left: G608G MSCs, Right: WT MSCs. *p<0.05, **p<0.01, ***p<0.001 according to the two-way ANOVA test. n = 3 biological repeats. **b**, Western blots of HSP90β, and CHOP proteins level in MSCs transfected with control shRNA or shRNA against *HSP90AB1*. Left: G608G MSCs, Right: WT MSCs. Lower: Quantification of the protein levels relative to β-actin. Data are presented as mean ± SD. *p<0.05, **p<0.01, ***p<0.001 according to the two-tailed unpaired t-test. n = 3 biological repeats. **c**, SA-β-Gal staining(left) and quantification(right) of MSCs transfected with control shRNA or shRNA against *HSP90AB1*. Upper: G608G MSCs, Lower: WT MSCs. *p<0.05, **p<0.01, ***p<0.001.

9. Does HSP90 serotonylation occur in vivo, and if so, in which cell types? How stable is this modification both in vitro and in vivo? How does it relate to the observed effects of 5-HT in G608G mice?

Answer to comment 9 of reviewer 1:

Thank you for the insightful questions. Here is our detailed response:

We appreciate the reviewer's thoughtful comments and would like to address these points in detail:

HSP90 serotonylation *in vivo*:

To investigate whether HSP90β serotonylation occurs *in vivo*, we performed new Click chemistry experiments using **ovarian tissues from 4-week-old (4W) and 12-month-old (12M) C57 mice**. These mice received intraperitoneal injections of 5-PT (a derivative of 5-HT, 4 mg/kg/day) for three consecutive days, and the ovarian tissue was used to perform IP experiments using an HSP90β antibody. Subsequent mass spectrometry analyses confirmed the presence of serotonylated HSP90β *in vivo* (Figure 18a, b as below and Extended Data Fig. 6f, g in the revised manuscript). Furthermore, relative quantification revealed that the levels of HSP90β serotonylation were significantly reduced in aging ovarian tissues compared to younger tissues, indicating an age-associated decline in this modification *in vivo* (Figure 18c as below).

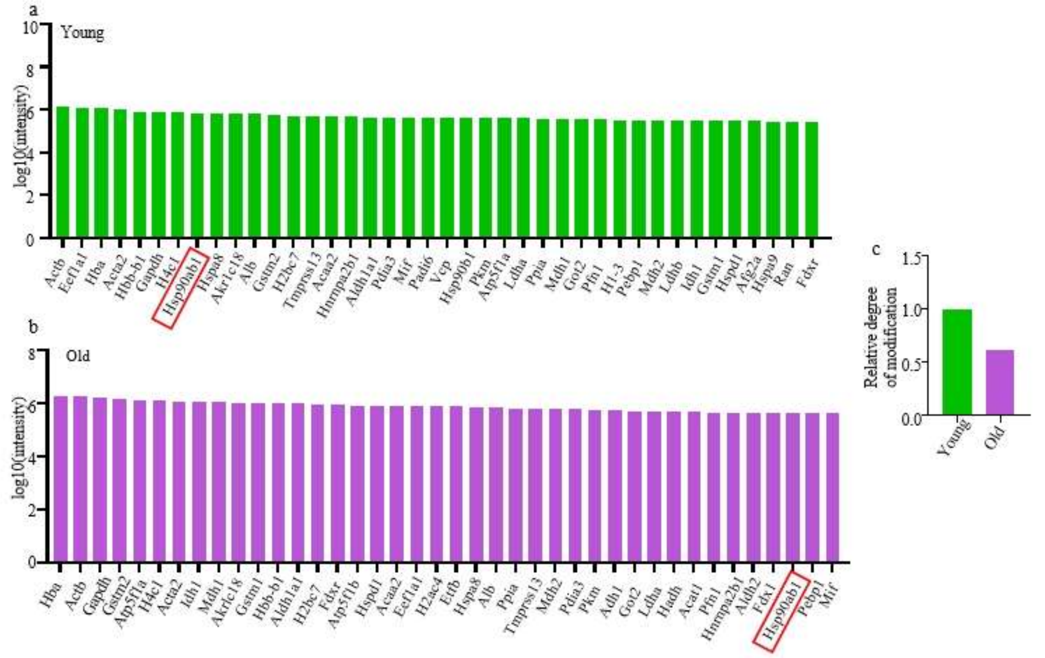

Figure 18

Figure 18: **a, b,** Heatmap of the top 40 enriched proteins of the ovaries 4-week-old (Young) and 12-month-old mice (Old). **c,** Quantification of the mass spectrometry intensity indicating HSP90β serotonylation in the ovaries of 4-week-old and 12-month-old mice.

Cell-specific serotonylation:

While our results confirm the occurrence of HSP90β serotonylation in ovarian tissue, we have not specifically investigated whether this modification is restricted to cell types. Theoretically, serotonylation can occur in any cell type that contains 5-HT and transglutaminase 2 (TGM2), the enzyme responsible for mediating the covalent attachment of serotonin to specific glutamine residues. However, we acknowledge the need for future experiments to verify this in other cell types.

Stability of serotonylation in vitro and *in vivo*:

Serotonylation involves the formation of a covalent bond between the amine group of serotonin and the side chain of glutamine residues in proteins, resulting in N-ε-(γ-glutamyl)-5-hydroxytryptamine (5-HT-Gln)[23,24]. Covalent bonds are generally robust and not easily disrupted by typical chemical or enzymatic pathways, suggesting that serotonylation is

relatively stable[25,26]. While we have not directly measured the stability of HSP90 serotonylation *in vitro* or *in vivo*, we infer its durability based on the inherent characteristics of covalent bonds. Due to the scope of the current manuscript, we haven't provided experimental data specifically assessing the stability of serotonylation or the possibility of reversal of this modification, which will be investigated in future studies.

Relevance to 5-HT effects in G608G mice:

Our *in vivo* confirmation of HSP90β serotonylation (Figure 18 as above) and its significant decline with aging aligns perfectly with the protective effects of 5-HT observed in aged mice. Serotonylation of HSP90β mitigates endoplasmic reticulum stress and promotes proteostasis, key mechanisms underlying the delayed senescence, and improved tissue health observed in our study. Although HGPS exhibits a more rapid progression of aging phenotypes, many of its cellular hallmarks (e.g., nuclear abnormalities, impaired proteostasis, and increased senescence) overlap with those observed in normal aging [27]. The age-related decline in serotonylation may partly explain the increased susceptibility to aging phenotypes, while 5-HT supplementation restores this modification and alleviates these effects in the G608G mice.

In conclusion, our study demonstrates that HSP90β serotonylation occurs *in vivo*, decreases with aging, and is a stable covalent modification. While we acknowledge the limitations in our ability to provide data on cell-type specificity and precise stability, these findings strongly support the role of HSP90β serotonylation in mediating the anti-aging effects of 5-HT. Thank you again for your constructive feedback.

10.The reprogramming experiment involving the four Yamanaka factors (Figure 3q,r) feels out of place, and no partial reprogramming experiments were conducted in G608G mice.

Answer to comment 10 of reviewer 1:

We want to thank the reviewer for the pertinent comments. We are also aware of the disconnection in this part of the reprogramming experiment, and we have removed this part of the results from the entire article to make it a more focused and coherent story.

11.The title of Figure 3 is misleading, as it only demonstrates a delay in some age-related phenotypes, not a reversal.

Answer to comment 11 of reviewer 1:

We would like to thank the reviewer for the pertinent comments. We have changed the title to **"5-HT block the senescence phenotype of MSCs"** of Figure 3 to make the wording as precise as possible.

Reviewer #2 (Remarks to the Author):

Manuscript by Xu et al described rescue of aging phenotypes in several tissues/cell types attributed to premature aging triggered by progeria (mutations in Lamin A) attributed to exposure to 5-HT (serotonin). Authors acknowledge that serotonin has been previously

implicated in aging of the brain however its effects on other tissues were not explored. Authors thus tried to connect the metabolomics of oocytes with rejuvenation/function of organelles (mitochondria, ER….) during aging. The data observed represents a potpourri of experiments, which sometimes lack focus.

Answer to the overall comment: We appreciate the reviewer's constructive comments, after carefully reading the suggestions, we acknowledge that our previous manuscript is not focused, we thus thoroughly reordered the figures and more focused on addressing the aging alleviating role of 5-HT, but not the physiological role of 5-HT and its related tryptophan metabolism in oocyte development. We think in the modified version of manuscript, our scientific question is more clearly illustrated, and the data presented are more focused and coherent.

1、Title is a bit misleading and proposes that Oocytes are enriched in Serotonin. This is true compared to zygotes/2-cell embryos, but other cell types were not profiled. Also, linkage to reprogramming efficiency is not clear, as presumably there is low level of serotonin in zygotes. Yet, zygotes are the ones actively reprograming both paternal sperm nuclei as well as any other somatic cell nuclei inserted in their cytoplasm. Data for metabolites should be included with quantitation in the supplemental table.

Answer to comment 1 of reviewer 2:

We appreciate the reviewer's comment. While we directly compare serotonin levels in oocytes to zygotes/2-cell embryos, we can provide additional context regarding serotonin abundance relative to other cell types.

In our targeted metabolomics analysis, we compared the relative abundance of serotonin to L-2-HG in oocytes. Based on our previous work[6], where we established absolute quantification of L-2-HG in MII-stage oocytes, we can estimate that the absolute concentration of serotonin in oocytes exceeds **3-5μM**. This estimation approach uses L-2-HG as an internal reference point whose absolute concentration was previously determined by us[6]. When compared to published serotonin concentrations in other cells and tissues documented in the literature, this level (>3-5μM) represents a considerable concentration(Table1 as below). This comparison provides evidence that serotonin is present at physiologically relevant levels in oocytes, even when benchmarked against other cell types and tissues known to utilize serotonin signaling (Table 1 as below).

Furthermore, the underlying context of our research focuses specifically on embryos with the highest regenerative capacity. By comparing metabolic profiles between oocytes and 1-cell/2-cell embryos, we can address the central questions relevant to our research focus. The oocyte is commonly considered to have reprogramming capacity. Importantly, the reprogramming capacity is "stored" in the oocyte, as it is demonstrated by the somatic cell nuclear transfer (SCNT) experiment that maternal factors in the oocyte mediate somatic cell reprogramming. The stored factors could include proteins, RNA and metabolites. The maternal protein and RNA have been intensively studied, but the stored metabolites have been poorly studied so far. In contrast to oocytes, **zygotes have not been recognized as having reprogramming capacity, as enucleated zygotes receiving nuclei from embryos beyond the two-cell stage fail to develop into blastocysts**[28,29]. We have also discussed this rationale of choosing oocytes as a tissue with the highest reprogramming

capacity in the ***Answer to comment 3 of reviewer 1.***

As the reviewer suggested, we have included the data for metabolites with quantification in the supplemental data (Table S1 of the revised manuscript supplemental data). This raw metabolic data we provide are further quantified in the following way, (taking the MII-1 sample as an example), the sum (Asum) of all metabolite's peak areas (intensity of the measured signal) measured in the sample was first calculated, and then the percentage of the peak area (An/Asum) of one metabolite in the MII-1 sample out of the sum of all measured metabolite peak areas in that sample was calculated (An/Asum) *100. In this way, we obtained a normalized metabolite quantification that could be compared across different samples.

Region	Concentration	Species	Reference
**MII（Estimated value）**	2-4μM	mice	[6]
**Serum**	5-30μM	human	[30]
**Frontal cortex**	1-2μM	rat	[31]
**Blood**	4-5μM	rat	[31]
**Brain**	4-5μM	mice	[32]
**Duodenum**	100-140μM	mice	[32]
**Spleen**	85-110μM	mice	[32]
**Regions of CNS**	0.119-68.4nM	mice	[33]

Table 1

3. Authors claim serotonin “rejuvenates” and/or prevents aging. Yet no aging studies were performed at all. All experiments were performed in progeria model of unknown age. This model, while often described as having accelerated aging has primarily a defect of nuclear laminas. Thus, secondary phenotypes triggered by laminopathies contribute to cell senescence in this model with additional secondary defects. True biological aging is driven by more complex mechanisms.

Answer to comment 2 of reviewer 2:

We appreciate the reviewer’s thoughtful feedback and would like to clarify our study design and findings. We respectfully disagree with the statement that we did not study aging. Our research specifically incorporated both *in vitro* and *in vivo* models to investigate aging-related processes comprehensively:

Progeria as a model of aging

While Hutchinson-Gilford Progeria Syndrome (HGPS) primarily results from mutations in

the LMNA gene, leading to the production of the truncated lamin A protein, progerin, research shows that progerin is also expressed at low levels in normal aging tissues. Its accumulation has been implicated in various age-related cellular phenotypes, even in non-HGPS individuals [34,35]. Thus, although HGPS exhibits a more rapid progression of aging phenotypes, many of its cellular hallmarks (e.g., nuclear abnormalities, impaired proteostasis, and increased senescence) overlap with those observed in normal aging [27]. This overlap supports the use of HGPS models to study mechanisms relevant to the broader aging process. We acknowledge that the primary defect in HGPS arises from LMNA mutations. However, the downstream consequences—such as oxidative stress, DNA damage, and mitochondrial dysfunction—are also central features of biological aging[12]. These secondary effects in HGPS offer insights into processes applicable to normal aging, as progerin accumulation in otherwise healthy fibroblasts can recapitulate aging-associated nuclear blebbing, telomere attrition, and elevated senescence markers[36]. The expression of progerin in both HGPS and normal aging tissues underscores the relevance of laminopathy-based models for studying universal aging pathways [27,35,36]. Progerin accumulation in normally aged cells has been linked to nuclear lamina disruption, chromosome mislocalization, and DNA damage—all hallmarks of aging[12]. Consequently, the molecular mechanisms identified in HGPS can inform broader aging research, particularly when combined with conventional aging models.

Recognizing that no single model fully captures the complexity of aging, we indeed incorporated both normal aging and premature aging models.

In vitro aging models:

Pathological aging model: We used wild-type (WT) MSCs as the control group and G608G MSCs as the experimental group, ensuring the same generation for direct comparison.

Replicative normal aging model: We used younger WT MSCs (Passage 3-5) as the control group and older WT MSCs (Passage 9-11) as the experimental group.

These MSC models recapitulate normal aging phenotypes as described in many literature [10,15].

Our results demonstrated that 5-HT treatment significantly decreased β-galactosidase expression, increased MSC proliferation in both aging models (Figure 19 as below and Fig.3a-c and 3e-g in the revised manuscript), reduced mRNA levels of cellular senescence markers and promote cell cycle in treated groups compared to controls (Figure 19 as below and Fig. 3d-3j in the revised manuscript). These findings provide robust evidence of 5-HT's ability to mitigate cellular senescence. **Nevertheless, as the reviewer suggested, we have toned down our statement to say that 5-HT's effect is to alleviate aging phenotypes or cellular senescence phenotypes, instead of straightly saying prevent aging, such as "alleviates aging-related" on page 8.**

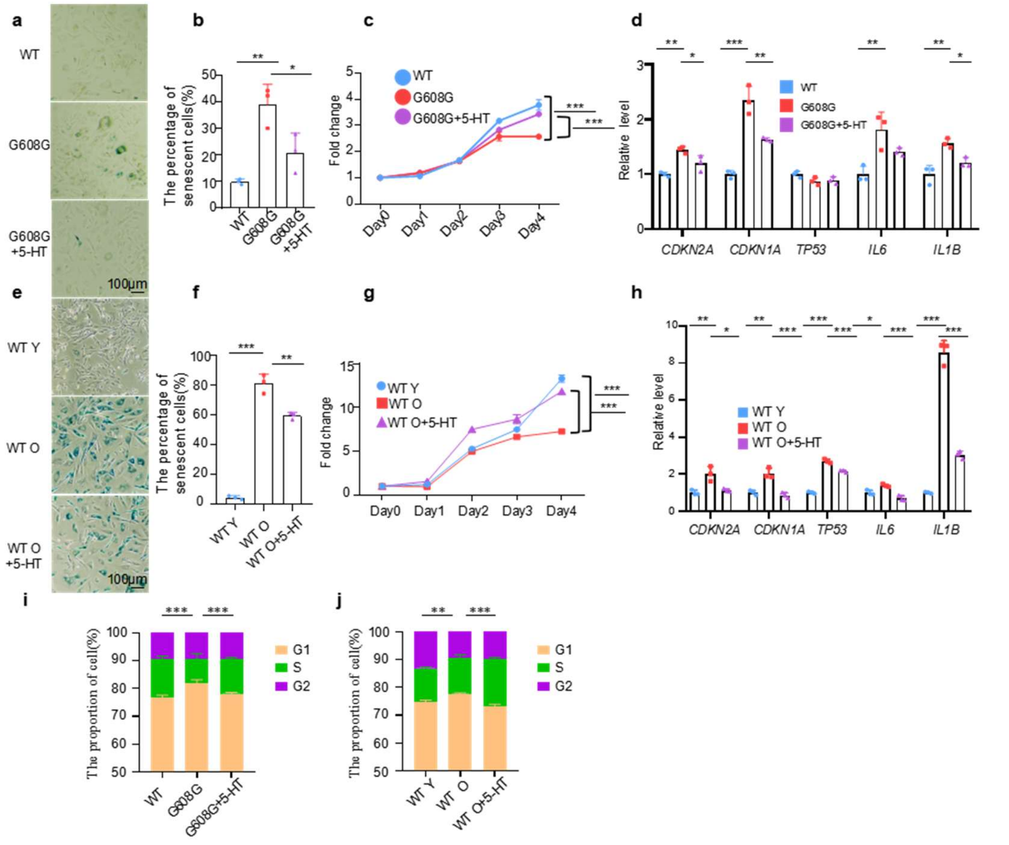

Figure 19

Figure 19: **a**, **b**, SA-β-Gal staining (**a**) and quantification (**b**) of WT MSC and G608G MSC with or without 5-HT treatment. *p<0.05, **p<0.01, ***p<0.001 according to the one-way ANOVA test. n = 3 biological repeats. Scale bar, 100μm. **c**, CCK-8 evaluation of proliferation of WT MSC and G608G MSC with or without 5-HT treatment. *p<0.05, **p<0.01, ***p<0.001 according to the two-way ANOVA test. n = 3 biological repeats. **d**, RT-qPCR analysis of *CDKN2A*, *CDKN1A*, *TP53*, *IL6*, and *IL1B* mRNA expression in WT MSC and G608G MSC with or without 5-HT treatment. *p<0.05, **p<0.01, ***p<0.001 according to the one-way ANOVA test. n = 3 biological repeats. **e**, **f**, SA-β-Gal staining (**e**) and quantification (**f**) of young generations of WT MSC(WT Y), old generations of WT MSC(WT O), and old generations of WT MSC treated with 5-HT(WT O+5-HT, 5μM). *p<0.05, **p<0.01, ***p<0.001 according to the one-way ANOVA test. n = 3 biological repeats. Scale bar, 100μm. **g**, CCK-8 evaluation of the proliferation of WT Y, WT O, and WT O+5-HT. The two-way ANOVA test was used for statistical analysis. n = 3 biological repeats. **h**, RT-qPCR analysis of *CDKN2A*, *CDKN1A*, *TP53*, *IL6*, and *IL1B* mRNA expression of WT Y, WT O, and WT O+5-HT. *p<0.05, **p<0.01, ***p<0.001 according to the one-way ANOVA test. n = 3 biological repeats. **i**,**j**, Cell cycle distribution patterns of WT MSCs and G608G MSCs of identical passage number (**i**) and young and aged WT MSCs (**j**). *p<0.05, **p<0.01, ***p<0.001 according to the two-way ANOVA test. n = 3 biological repeats.

In vivo aging studies:

G608G mouse model: We administered 5-HT orally to G608G homozygous mice starting at 1 month of age until 5 months (for lifespan studies, treatment continued until natural death). Anatomical and histological analyses were performed to assess aging-related indicators at the tissue level (Fig. 3l-3o, Extended data Fig. 3h-k in the revised manuscript). Detailed information about the mice's age and duration of administration has been added to the figure legends. We acknowledge that G608G mice primarily exhibit laminopathy-driven senescence, which may not fully capture the complexity of biological aging. However, due to their short lifespan (~7 months), they serve as a convenient and widely used model in aging-related studies[2,37,38]. The use of G608G mice allowed us to evaluate the effects of 5-HT on lifespan and aging-related phenotypes.

Normal aging model: To complement the G608G model, we conducted additional experiments using wild-type (WT) mice. Specifically, 12-month-old WT C57 mice were given 5-HT orally for 8 months. At 20 months of age, organ tissues were harvested for Massion or H&E staining (Figure 20 as below, Fig.3p-s and Extended Data Fig.3n-q in the revised manuscript**). This provided further evidence for the effects of alleviating aging phenotypes by 5-HT in a normal aging context.**

We fully acknowledge that biological aging is a complex, multifaceted physiological process involving numerous mechanisms. **Therefore, we employed a combination of in vitro and *in vivo* models, including both pathological and normal aging contexts**, **to comprehensively evaluate the effects of 5-HT on aging.** While no single model can capture the entirety of aging biology, the integration of these approaches strengthens the relevance and robustness of our findings.

Nevertheless, as the reviewer suggested, we have toned down our statement to say that 5-HT's effect is to alleviate aging phenotypes or cellular senescence phenotypes, instead of straightly saying prevent aging.

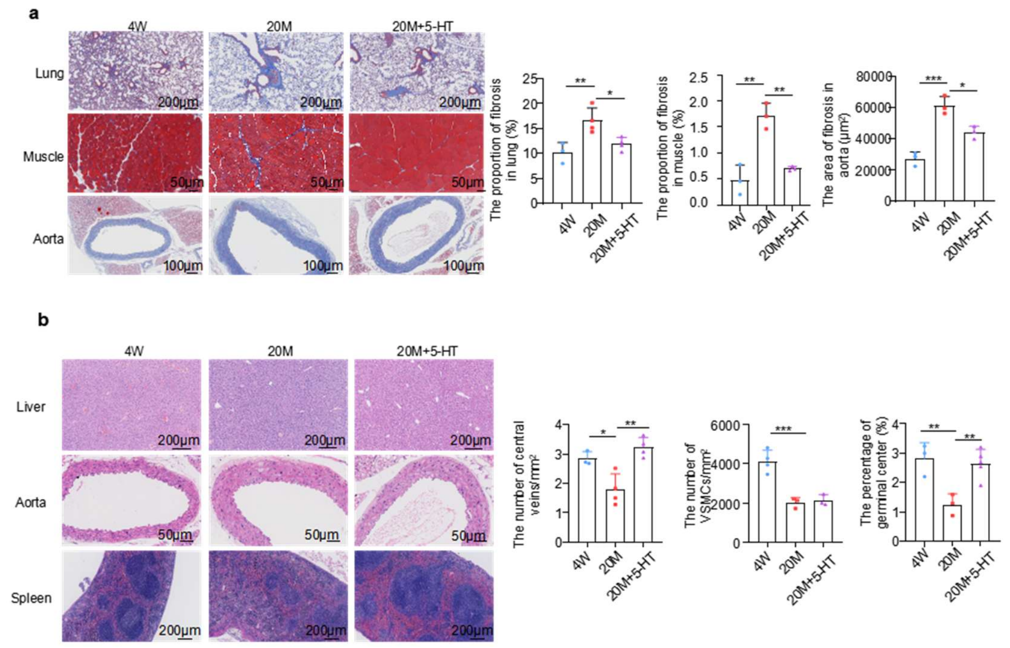

Figure 20

Figure 20: **a,** Masson staining and quantification of fibrosis area of lung, muscle, and aorta in 4-week-old and 20-month-old wild type mice treated with 5-HT or not (from 12-month-old to 20-month-old). *p<0.05, **p<0.01, ***p<0.001 according to the one-way ANOVA test. n = 3-4 biological repeats. Scale bar, 200, 50 and 100μm. **b**, Histological analysis (HE) of the liver, spleen, and aorta of 4-week-old and 20-month-old C57 mice treated with 5-HT or not (from 12-month-old to 20-month-old); Quantitative analysis of the number of central veins per unit area (mm^2) in the liver, the average VSMC number per unit area (mm^2) in the aorta, and the percentage of germinal center area in the spleen. *p<0.05, **p<0.01, ***p<0.001 according to the one-way ANOVA test. n = 3-4 biological repeats. Scale bar, 200 and 50 μm.

3.Are serotonin levels/ and or receptors affected by progeria mutations? Is there lack of serotonin in patients with laminopathies? Is there change of serotonin in aging? Do patients taking SSRI (antidepressants) exhibit any changes of aging phenotypes?

Answer to comment 3 of reviewer 2:

Thank you for your thoughtful comments and questions. Below is our detailed response

Serotonin levels/receptors and progeria mutations:

We appreciate the question regarding the relationship between serotonin and progeria mutations. Although the specific impact of progeria mutations (such as those in LMNA) on serotonin levels or receptors has not been extensively studied, our RNA-seq results suggest that expression of certain serotonin receptors such as 5HTR1B and their downstream mitochondrial functional pathways are decreased in G608G MSCs (Figure 21 as below, Extended Data Fig.5e, b in the revised manuscript).

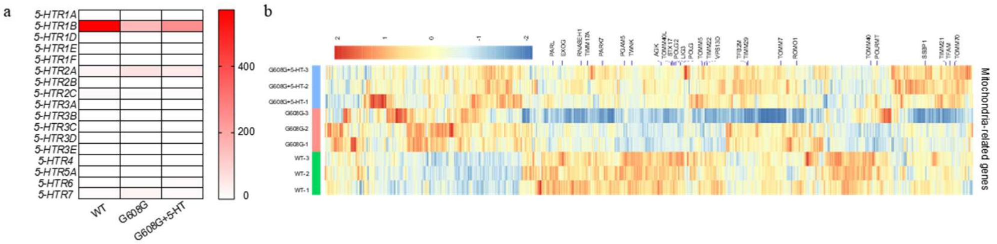

Figure 21

Figure 21: **a,** Heat map of expression levels of all 5-HT receptors in MSC from RNA sequencing results above mentioned. **b,** Heatmaps of the genes related to mitochondria of WT MSC and G608G MSC treated with 5-HT or not.

Serotonin levels in laminopathy patients:

To our knowledge, there is no clinical or biochemical evidence demonstrating consistently reduced serotonin levels in HGPS or other laminopathy patients. Serotonin levels do reduce in normal aging as described later below (Figure 22 as below, <u>Fig. 2e, f in the revised manuscript</u>). What' more, 5-HT and aging phenotypes are not directly linked to laminopathies. The mechanisms by which serotonin influences aging are independent of the primary defects in laminopathies, which involve mutations in the LMNA gene and nuclear envelope dysfunction. While our current study does not investigate serotonin levels

in human laminopathy patients, it provides a mechanistic framework suggesting that serotonin pathways may play a role in mitigating aging-related phenotypes in such conditions.

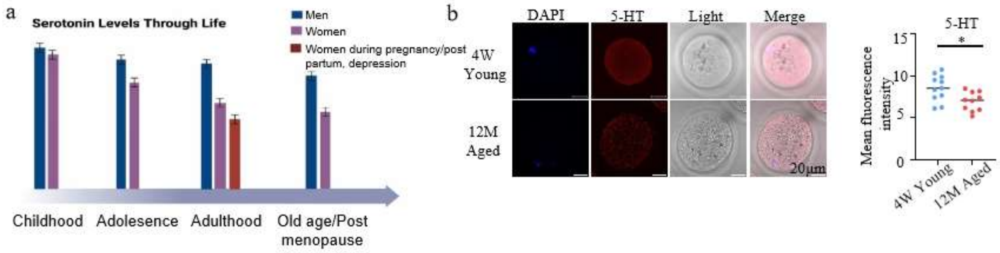

Figure 22

Figure 22: **a,** This infographic illustrates serotonin levels across different life stages using a bar graph[9]. **b,** Immunofluorescence analysis and quantification of 5-HT of 4-week-old C57 and 12-month-old C57 MII oocytes. Scale bar, 20μm. *p<0.05, **p<0.01, ***p<0.001 according to the two-tailed unpaired t-test. n = 10-11 biological repeats.

Changes in serotonin with aging:

Our data support the idea that metabolites in serotonin-related pathways decline with age. Specifically, spatial metabolomics of mouse ovarian tissue revealed an age-associated decline in serotonin-related metabolites, including tryptophan (upstream) and several downstream metabolites (Figure 2 as above). Immunofluorescence analysis also showed that the level of 5-HT in oocytes of old female mice was decreased compared with that of young mice (also discussed in detail in Figure 1 above in the ***Answer to comment 3 of reviewer 1***). These findings align with prior studies suggesting that serum serotonin levels decrease with age[9], which could contribute to increased susceptibility to age-related stress and dysfunction [39]. This observation is consistent with our hypothesis that serotonin pathways are involved in aging regulation.

SSRI use and aging phenotypes:

The relationship between selective serotonin reuptake inhibitors (SSRIs) and aging phenotypes is not well understood, as SSRIs function by selectively inhibiting serotonin reuptake transporters (SERT) in the central nervous system, thereby increasing 5-HT concentrations specifically within the synaptic clefts of neurons[40,41]. This mechanism primarily affects neurotransmission in brain regions associated with mood regulation, rather than elevating systemic serotonin levels throughout the entire body[42]. Limited evidence suggests that SSRI administration may confer some protection against age-related cognitive decline and inflammation, but these effects are likely mediated through indirect mechanisms. One particularly intriguing clinical study tracked 20 non-diabetic patients with depression, examining insulin sensitivity parameters before and after antidepressant treatment. Both intravenous and oral glucose tolerance tests (FSIGT and OGTT, respectively) demonstrated significantly enhanced insulin sensitivity following SSRI administration (Figure 23 as below)[43], suggesting that serotonergic signaling may influence susceptibility to age-related pathologies. This suggests that serotonin signaling can modify the risk of several major age-related human disorders. Our research, however, focuses specifically on how 5-HT delays cellular aging through both classical

receptor-mediated pathways and non-classical mechanisms such as the intracellular serotonylation of HSP90β mediated by TGM2. This intracellular serotonylation process represents a distinct molecular mechanism unlikely to be substantially influenced by SSRI treatment, which primarily targets neurotransmission in the central nervous system rather than peripheral cellular aging processes or intracellular posttranslational modifications. We have briefly discussed these points in the revised manuscript.

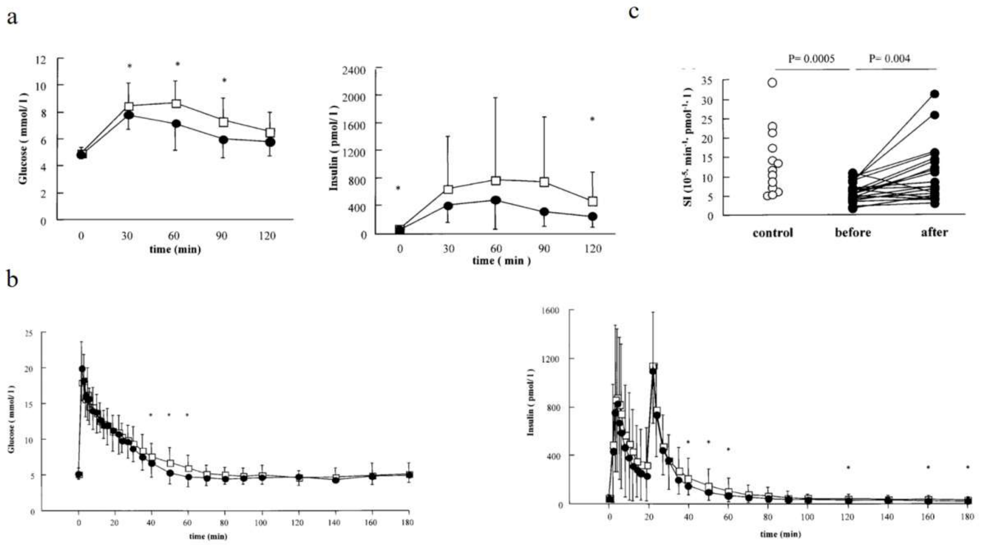

Figure 23

Figure 23: **a,** Glucose (left) and insulin (right) responses during the OGTT in 20 patients with depression before ( ) and after ( ) treatment of depression. Data are the mean±SD. Fisher's PLSD test was applied after ANOVA with repeated measures. *Statistically significant at p <0.05. **b,** Glucose (left) and insulin (right) responses during the FSIGT in 20 patients with depression before ( ) and after ( ) treatment of depression. Data are the mean±SD. Fisher's PLSD test was applied after ANOVA with repeated measures. *Statistically significant at p <0.05. **c,** Individual measurements of insulin sensitivity derived from minimal model analysis in control subjects and in patients with depression before and after treatment. Student's t-test was used for unpaired comparisons, and the paired t-test was used for paired comparisons. Data are cited from the paper of Okamura, F. et al.

4.No n values are provided for several graphs (Fig 1h Fig2, f) or are extremely limited with sample size of 7-14 oocytes (Fig 1j), meaning there is likely no replication of experiments (just one trial). Fig 2 c has only 2 replicas and thus statistics cannot be performed.

Answer to comment 4 of reviewer 2:

We want to thank the reviewer for the pertinent comments. Based on the core idea of the whole paper to prove the effect of 5-HT on cell senescence and its related mechanism, we decided to remove this part of the data about 5-HT's function in maintaining oocyte development. For the rest of the paper and figures, we all have sufficient number of replicates to draw statistically significant conclusions.

5.Work with oocytes (using siRNA) is suboptimal. If authors wish to study serotonin pathway in oogenesis they should do so by interfering with its production genetically – in a conditional model. Use of parthenogenetic embryos for assessment of developmental competence is suboptimal and purely represents limitation of siRNA approaches.

Answer to comment 5 of reviewer 2:
We want to thank the reviewer for the pertinent comments. We acknowledge the limitations of the siRNA approach to study the effects of 5-HT on oocyte development, and the genetic model of conditional knockout is more suitable. However, based on the core idea of the whole paper to prove the effect of 5-HT on cell senescence and its related mechanism, we decided to remove this part of the data about 5-HT maintaining oocyte development to make it a more focused and coherent story.

6.For experiments with LDN – what is the control for specificity of this compound?

Answer to comment 6 of reviewer 2:
We would like to thank the reviewer for the pertinent comments. LDN-27219 is a reversible inhibitor of slow-binding TGase. **LDN-27219 inhibited human TGase with an IC50 of 0.6 μM**. Previous studies have shown that LDN-27219 (30 μM; 12 min) promotes the closed conformation of TGM2[44]. Xu Wang et al. also used LDN-27219 to assess whether TGM2-involved serotonylation is essential in their study of serotonylation of GAPDH for 5-HT-induced CD8+ T cell activation[45]. Thus it is an established TGM2 specific inhibitor from the previous literatures.

7.Some control experiments are missing. Authors should have performed all experiments with WT animals/cells also treated with 5-HT for proper comparison. Western blot data is not statistically quantitated (Fig 3h, 4c, 6b,7i).

Answer to comment 7 of reviewer 2:
We would like to thank the reviewer for the pertinent comments. We have included the WT treatment experiments in the revised manuscript. Specifically, to demonstrate the effect of 5-HT on senescent cells, we established two *in vitro* models of senescent cells, one for pathological senescence and the other for replicative senescence. For young WT MSCs, as well as old WT MSCs and G608G MSCs of the same generation, we simultaneously conducted 5-HT treatment. The results demonstrated that 5-HT effectively delayed cellular senescence phenotypes in both replicative (normal) aging and pathological aging models, including the reduction of *CDKN1A*, *CDKN2A* expression and decreased proportion of β-gal positive cells. However, its effects were not significant in cells that were already young (Figure 24 as below). Therefore, in subsequent experiments, we conducted exploration and verification separately in these two models and our study employed consistent cell models throughout all experiments. **WT MSC of the same generation** was used as the control group and G608G MSC was used as the experimental group. In the latter group, **the young WT MSC of P3-P5 was used as the control group**, and the old WT MSC of P9-P12 was used as the experimental group. Regarding the validation of 5-HT *in vivo*

models, except for the G608G premature aging model, we have **completed validation in a natural aged mouse model**. Anatomical experiments were performed after 12 months of oral administration of C57 and 8 months of 5-HT (0.375mg/kg/day), and the relevant data are shown in Figure 25 as below (Fig.3p-s and Extended Data Fig.3n-q in the revised manuscript). In addition, the mechanism of serotonylation of HSP90β was also demonstrated in two in vitro cell models, as shown to Figure 26, 27, 28 as below, and Fig.5, 6, and Extended Data Fig.8 respectively in the revised manuscript

Regarding WB quantitative information, we have completed three repeated experiments, as shown in the figure below (Figure 29 as below)

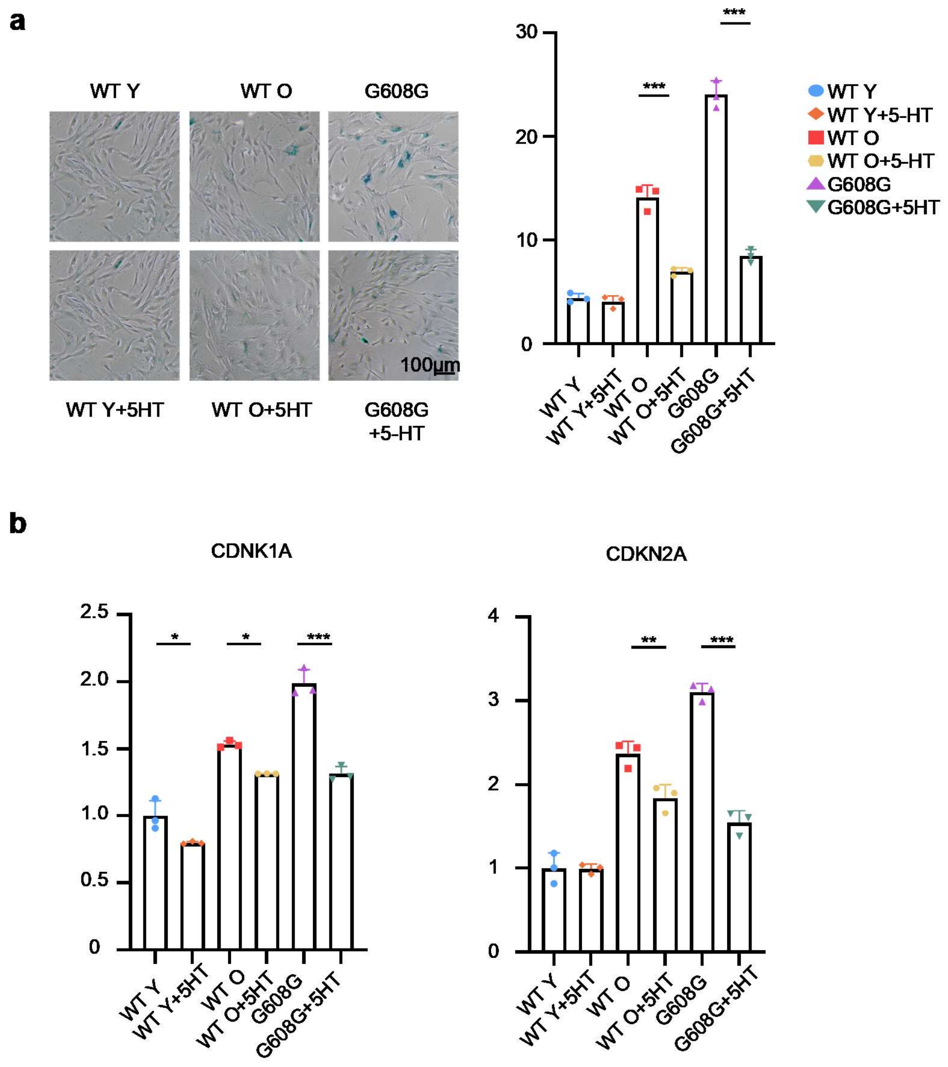

Figure 24

Figure 24: **a**,β-Gal staining (left) and quantification (right) of WT Y, WT O and G608G MSCs treated with 5-HT or not. *p<0.05, **p<0.01, ***p<0.001 according to the one-way ANOVA test. n = 3 biological repeats. Scale bar, 100 μm. **b**, RT-qPCR analysis of *CDKN2A*, *CDKN1A*, mRNA expression of WT Y, WT O and G608G MSCs treated with 5-HT or not. *p<0.05, **p<0.01, ***p<0.001 according to the one-way ANOVA test. n = 3 biological repeats

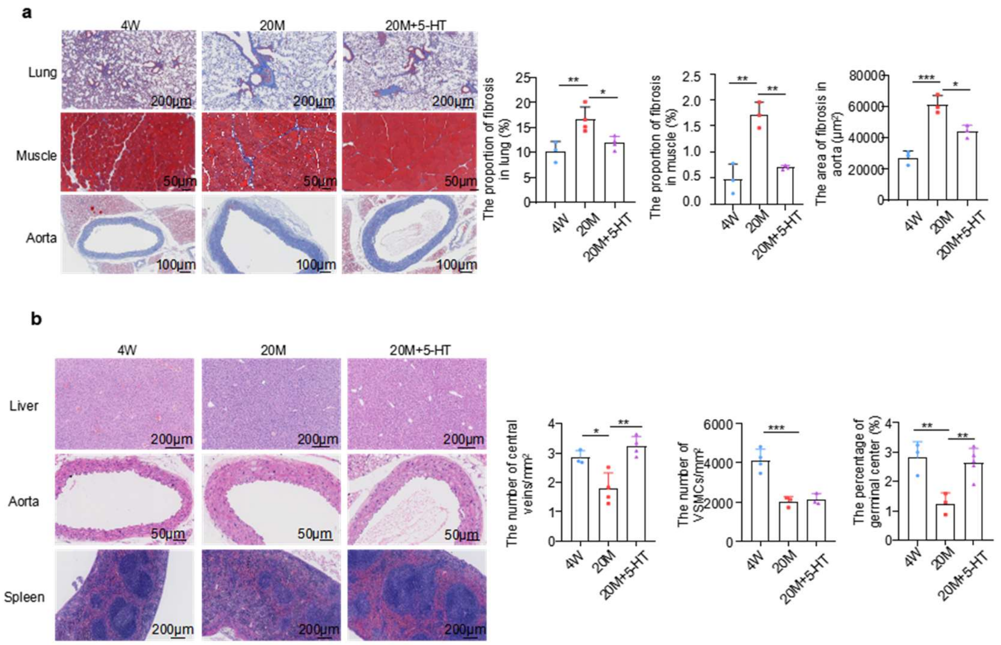

Figure 25

Figure 25: **a,** Masson staining and quantification of fibrosis area of lung, muscle, and aorta in 4-week-old and 20-month-old wild type mice treated with 5-HT or not (from 12-month-old to 20-month-old). *p<0.05, **p<0.01, ***p<0.001 according to the one-way ANOVA test. n = 3-4 biological repeats. Scale bar, 200, 50 and 100μm. **b**, Histological analysis (HE) of the liver, spleen, and aorta of 4-week-old and 20-month-old C57 mice treated with 5-HT or not (from 12-month-old to 20-month-old); Quantitative analysis of the number of central veins per unit area (mm^2) in the liver, the average VSMC number per unit area (mm^2) in the aorta, and the percentage of germinal center area in the spleen. *p<0.05, **p<0.01, ***p<0.001 according to the one-way ANOVA test. n = 3-4 biological repeats. Scale bar, 200 and 50 μm.

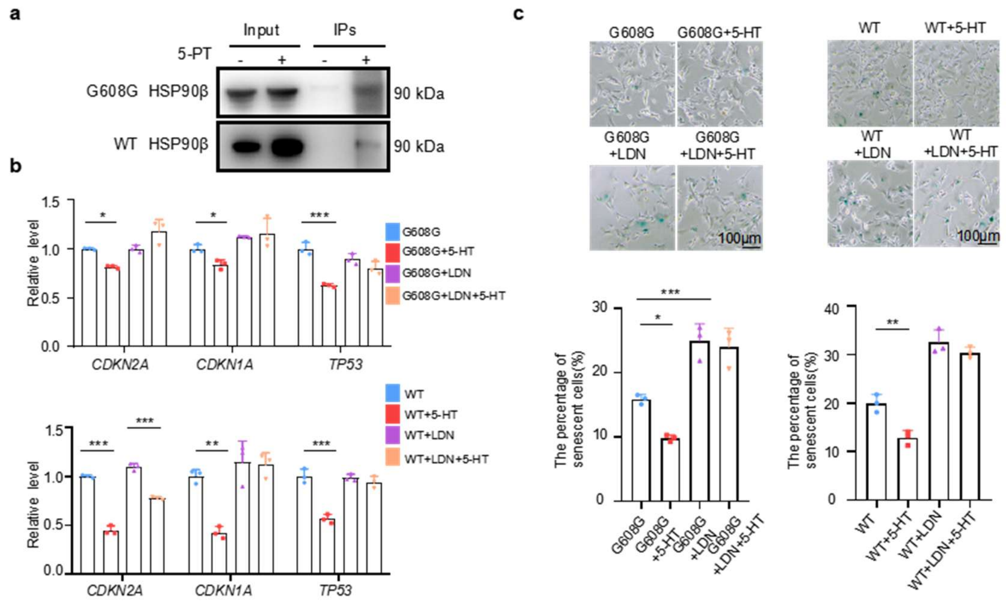

Figure 26

Figure 26: **a**, MSCs were cultured 72 h, with or without 5 μmol/L 5-PT. 5-PT was immunoprecipitated by beads, and HSP90β were detected. IP, immunoprecipitation. Upper: G608G MSCs, Lower: WT MSCs. **b**, RT-qPCR analysis of *CDKN2A*, *CDKN1A*, and *TP53* mRNA expression in MSCs treated with 5-HT, LDN or LDN+5-HT, LDN: LDN-27219, TGM2 inhibitor. Upper: G608G MSCs, Lower: WT MSCs. *p<0.05, **p<0.01, ***p<0.001 according to the one-way ANOVA test. n = 3 biological repeats. **c**, SA-β-Gal staining (upper) and quantification (lower) of MSCs treated with LDN or LDN+5-HT. Left: G608G MSCs, Right: WT MSCs. *p<0.05, **p<0.01, ***p<0.001 according to the one-way ANOVA test. n = 3 biological repeats. Scale bar, 100 μm.

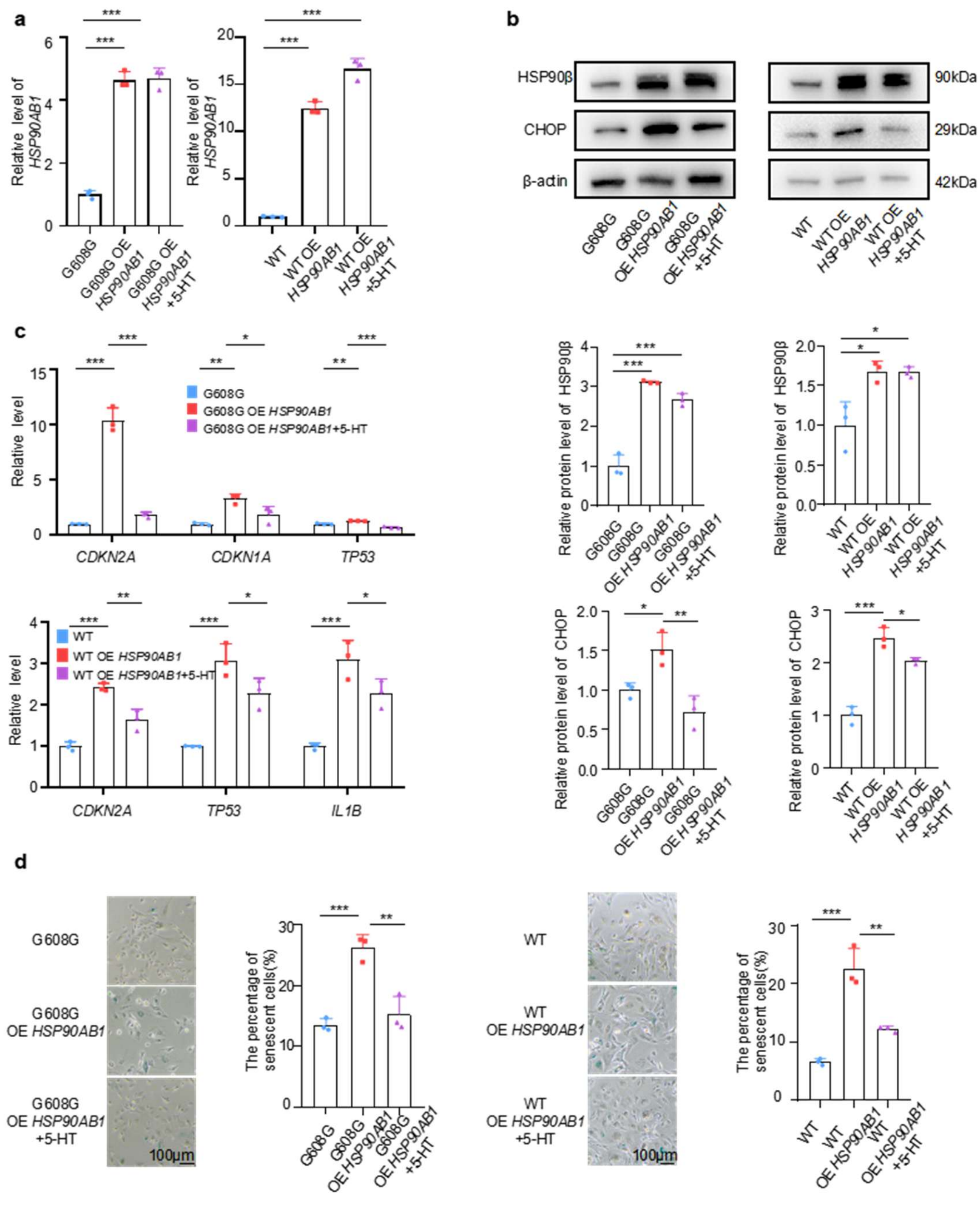

Figure 27

Figure 27: **a**, RT-qPCR analysis of *HSP90AB1* mRNA expression in MSCs and *HSP90AB1*-overexpression MSCs with or without 5-HT treatment. Left: G608G MSCs, Right: WT MSCs. *p<0.05, **p<0.01, ***p<0.001 according to the one-way ANOVA test. n = 3 biological repeats. **b**, Western blots of HSP90β, CHOP protein levels in MSCs and *HSP90AB1*-overexpressing MSCs with or without 5-HT treatment. Left: G608G MSCs, Right: WT MSCs. Lower: Quantification of the protein levels relative to β-actin. Data are presented as mean ± SD. *p<0.05, **p<0.01, ***p<0.001 according to the one-way ANOVA test. **c**, RT-qPCR analysis of *CDKN2A*, *TP53*, and *IL1B* or *CDKN1A* mRNA expression in MSCs and *HSP90AB1*-overexpression MSCs with or without 5-HT treatment. Upper: G608G MSCs, Lower: WT MSCs. *p<0.05, **p<0.01, ***p<0.001 according to the one-way

ANOVA test. n = 3 biological repeats. **d**, SA-β-Gal staining(left) and quantification(right) of MSCs and *HSP90AB1*-overexpression MSCs with or without 5-HT treatment. Left: G608G MSCs, Right: WT MSCs. *p<0.05, **p<0.01, ***p<0.001 according to the one-way ANOVA test. n = 3 biological repeats. Scale bar, 100 μm.

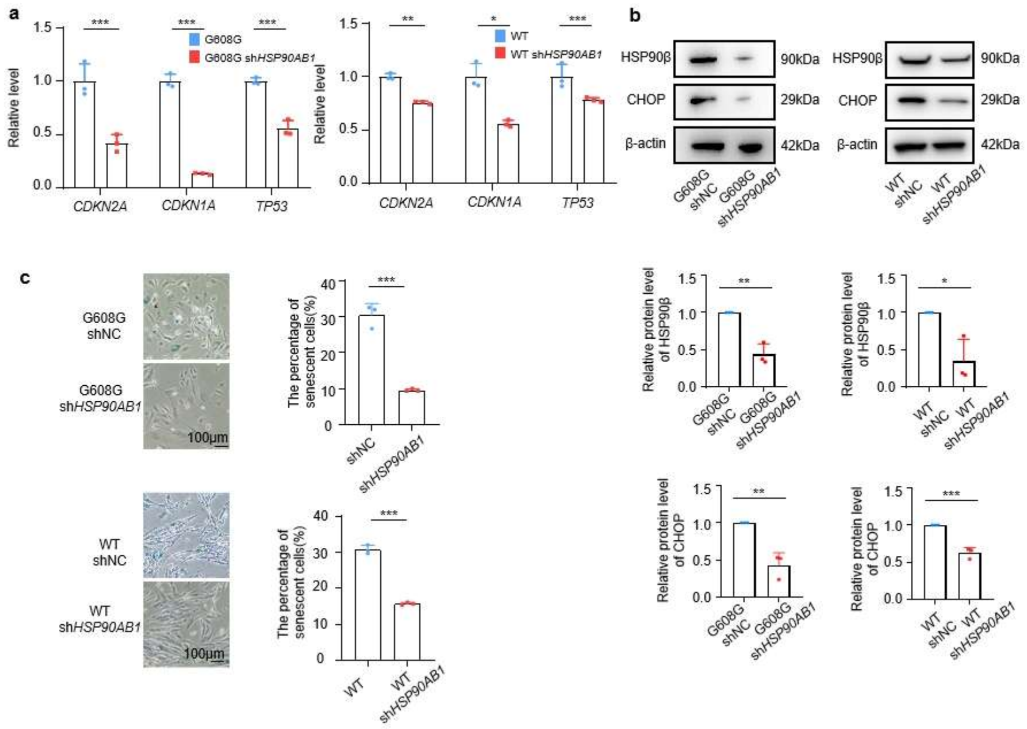

Figure 28

Figure 28: **a**, RT-qPCR analysis *CDKN2A*, *CDKN1A*, and *TP53* mRNA expression in MSCs transfected with control shRNA or shRNA against *HSP90AB1*. Left: G608G MSCs, Right: WT MSCs. *p<0.05, **p<0.01, ***p<0.001 according to the two-way ANOVA test. n = 3 biological repeats. **b**, Western blots of HSP90β, and CHOP proteins level in MSCs transfected with control shRNA or shRNA against *HSP90AB1*. Left: G608G MSCs, Right: WT MSCs. Lower: Quantification of the protein levels relative to β-actin. Data are presented as mean ± SD. *p<0.05, **p<0.01, ***p<0.001 according to the two-tailed unpaired t-test. n = 3 biological repeats. **c**, SA-β-Gal staining(left) and quantification(right) of MSCs transfected with control shRNA or shRNA against *HSP90AB1*. Upper: G608G MSCs, Lower: WT MSCs. *p<0.05, **p<0.01, ***p<0.001.

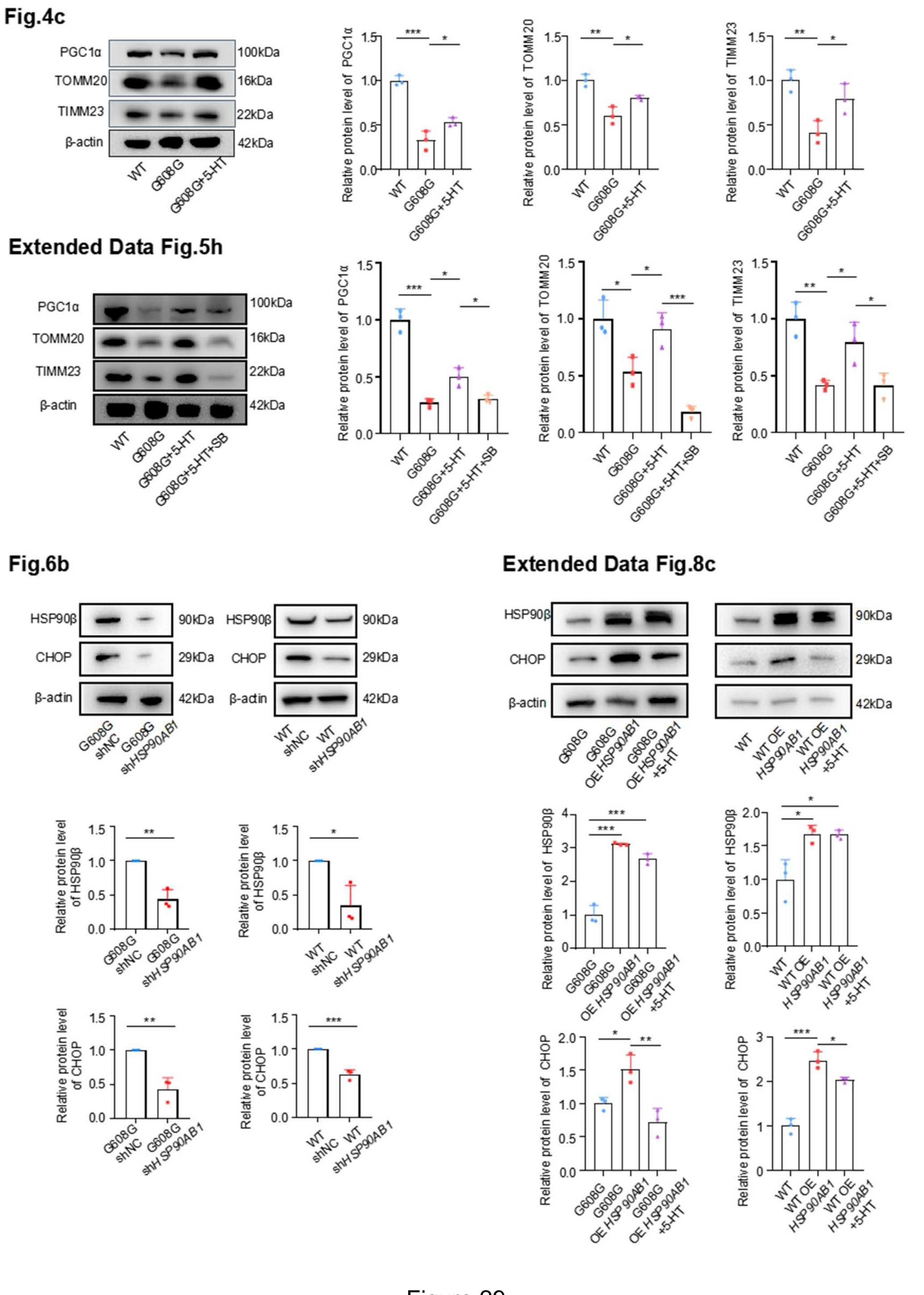

Figure 29

Figure 29: The results of WB and statistical quantitative results of all WB experiments.

8.The results of serotonylation experiments are intriguing. However, qRT-PCR data as a readout for UPR is not sufficient. Additional markers, cellular consequences should be explored. Are there defects in any identified serotonylated proteins in progeria patients/animals or is this a product of cell culture environment? List of identified proteins

was not provided (only bioinformatic analysis of KEGG. These data should also be made available.

Answer to comment 8 of reviewer 2:

We would like to thank the reviewer for the pertinent comments. In addition to qRT-PCR to verify transcriptome levels of URP-associated proteins, we selected intracellular **calcium ion concentrations as an alternative readout of the UPR**. ER stress will cause calcium ions in the ER to flow out into the cytoplasm, and the depletion of calcium ions in the ER will further aggravate ER stress (Figure 30 as below, Fig 7a, b in the revised manuscript). Therefore, there is a positive feedback loop between ER stress and calcium ion signals. In the following picture with our new experimental data, panel a is using G606G progeria cells, and panel b is using WT cells. Fura-2AM staining indicates calcium signal and ER stress. Endogenous HSP90AB1 was knocked down, and then WT HSP90AB1 and Q207A (the serotonylation site mutant) or Q493A (irrelevant control mutant) mutant HSP90AB1 were overexpressed. 5-HT treatment alleviated ER stress when WT HSP90AB1 was present, and failed to do so when Q207A mutant HSP90AB1 was present. These data strongly demonstrated that 5-HT alleviated ER stress through serotonylation of HSP90AB1 protein' Q207 amino acid.

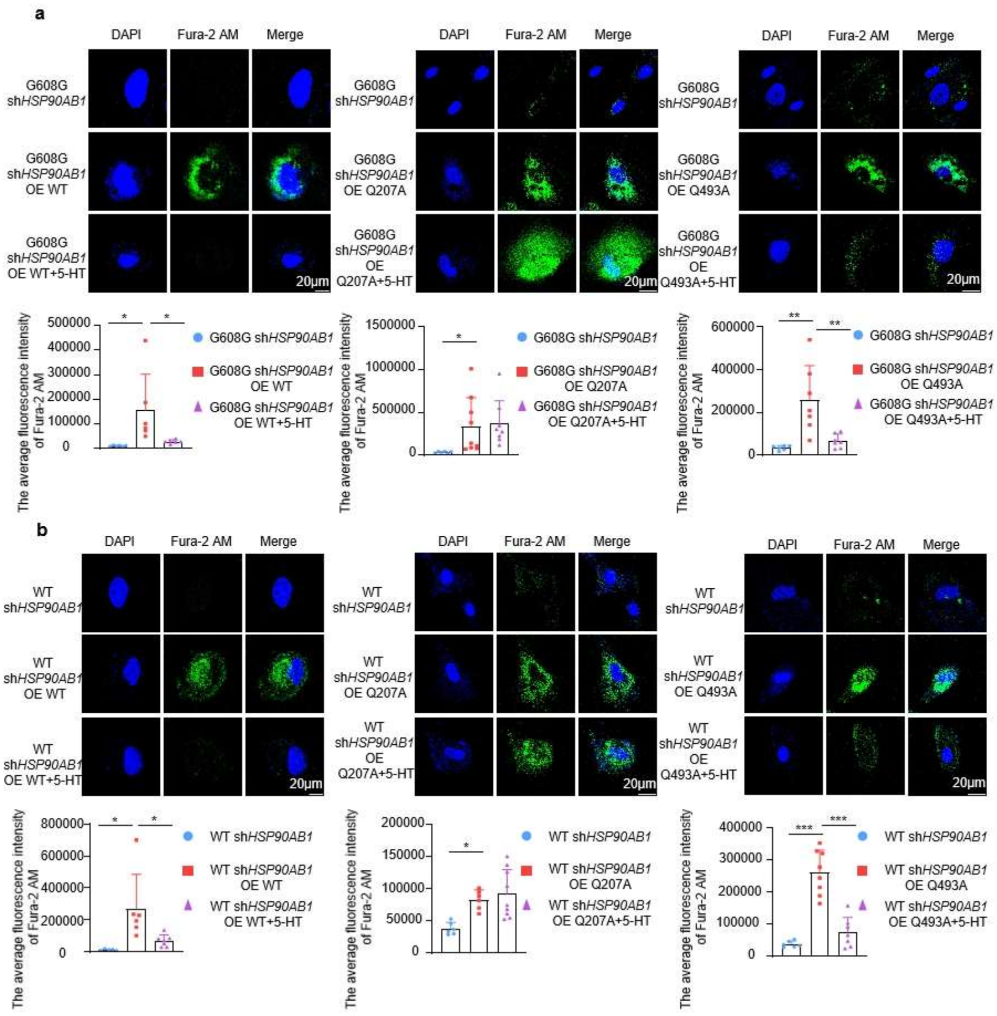

Figure 30

Figure 30: **a**, sh*HSP90AB1* G608G MSCs were transfected with mRFP-fused lentiviruses expressing either WT-HSP90β or Gln to Ala(Q207A, Q493A) mutated HSP90β. Cells were treated with 5-HT or not. Immunofluorescence analysis and quantification of Fura-2 AM. *p<0.05, **p<0.01, ***p<0.001 according to the one-way ANOVA test. n ≥ 3 biological repeats.Scale bar, 20 μm. **b**, sh*HSP90AB1* WT MSCs were transfected with mRFP-fused lentiviruses expressing either WT-HSP90β or Gln to Ala(Q207A, Q493A) mutated HSP90β. Cells were treated with 5-HT or not. Immunofluorescence analysis and quantification of Fura-2 AM. *p<0.05, **p<0.01, ***p<0.001 according to the one-way ANOVA test. n ≥ 3 biological repeats. Scale bar, 20 μm. Data are presented as mean ± SD.

Our IP experiment results show that (shown in Figure 31 as below and also in Fig.5e of the revised manuscript) the 5-HT in the G608G MSC and WT MSC can both have serotonylation of HSP90β. At present, there is no other literature indicating that there is a deficiency of serotonylation in patients/animals with premature aging, and we simply use progeria as a model of accelerated aging. There is indeed evidence of a decline of 5-HT in normal aging as illustrated above. **To demonstrate whether there is a decline of**

serotonylation during normal aging, we obtained ovarian tissue and performed Click chemistry experiments on 4W and 12M C57 mice after intraperitoneal injection of 5-PT (4mg/kg/day) for three consecutive days. Subsequent mass spectrometry results showed that HSP90β *in vivo* also had serotonylation modification (Figure 32a, b as below and Extended Data Fig.6e,f in the revised manuscript), and relative quantitative results showed that the amount of HSP90β modified by serotonylation decreased in aging ovaries (Figure 32c as below). Therefore, we do not think this post-translational modification only occurs during cell culture.

Tables S2 and S3 in the revised manuscript respectively provide the list of serotonylation modified proteins identified in the in vitro and in vivo experiments.

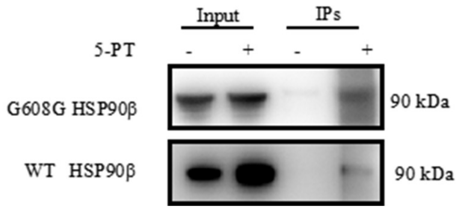

Figure 31

Figure 31: MSCs were cultured 72 h, with or without 5 μmol/L 5-PT. 5-PT was immunoprecipitated by beads, and HSP90β were detected. IP, immunoprecipitation. Upper: G608G MSCs, Lower: WT MSCs

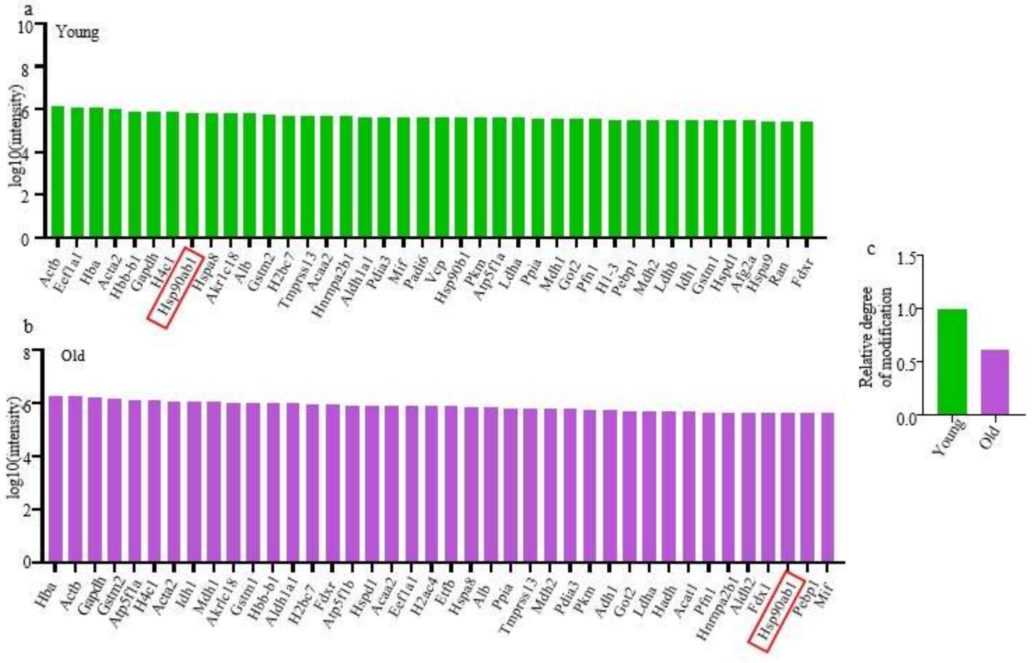

Figure 32

Figure 32: **a,b,** Heatmap of the top 40 enriched proteins of the ovaries of 4-week-old and 12-month-old mice. **c,** Quantification of the mass spectrometry intensity indicating HSP90β serotonylation in the ovaries of 4-week-old and 12-month-old mice.

Reviewer #3 (Remarks to the Author):
Oocyte-Enriched Metabolite Serotonin-Inspired Rejuvenation Strategy through1 Serotonylation of HSP90β

SUMMARY OF KEY RESULTS
The authors document an in-vivo modeling of oocyte development and highlight serotonin as a metabolite that exhibits a range of critical roles in cellular reprogramming and potential cell rejuvenation. The article highlights the important role metabolites play, where historically proteins (sic – Yamanaka factors) have been seen as the main biomolecular contributors to phenotypical changes in oocyte biology – this is a novel finding. The authors then proceed to demonstrate that serotonylation of HSP90β has potential as an anti-aging metabolite.

The overall the article is original, written to a high standard, abstract is concise, figures are of a high quality, and the hypothesis and methodologies employed within this study are 'generally' suitable for consideration in this venue – but there are some strong reservations below that should be:

Answer to the overall comment: We highly appreciate the reviewer's strongly positive comment that out study is **"a novel finding"**, also the comments that our manuscript **" is original, written to a high standard, abstract is concise, and figures are of high quality, and are generally suitable for consideration in this venue".**

DATA/METHODS/STATS
The flowing corrections need to be addressed:

Comment 1 of reviewer 3
• Page 2 – The authors describe their approach as a 'comprehensive metabolomics landscape' yet they only measure 256 metabolites and provided data (in associated .xls file) on 124. This needs to be changed to accurately reflect the size of the chemical space that has been measured.

Answer comment 1 of reviewer 3:
Response to Reviewer Comment

Thank you for your insightful comment regarding our metabolomics approach description. We apologize for any potential mischaracterization and have revised our manuscript accordingly to more accurately reflect the scope of our study.

To clarify, when initially establishing this metabolomics methodology, we developed an analytical framework targeting 262 metabolites, as mentioned in our previous publications. For metabolite identification, we optimized the parent/product ions of MRM transitions by

injecting analytical standards into the MS spectrometer and determined retention times by injecting standards into the LC-MS system. Finally, 256 common and important metabolic pathway metabolites were included in this method.

In our embryo samples specifically, we detected 124 metabolites (77 ion transitions in the positive mode and 47 ion transitions in the negative mode), which were included in the associated Excel file. We have revised our language throughout the manuscript to refer to this as a"targeted metabolomics analysis" rather than a "comprehensive metabolomics landscape" to more accurately reflect the scale of our chemical profiling.

Thank you for highlighting this important distinction, as precision in describing analytical scope is essential for proper interpretation of our findings.

Comment 2 of reviewer 3

• Page 3 – Pooling 100 embryos into x3 biological replicates need to be justified from an effect size and experimental variability perspective. This is a critical point.

I understand that there can be a limitation in the number of animals/cells that can be extracted, but this is not a reason to compromise the significance in a study. This needs to be discussed and statistically evidenced alongside the use of technical replication in all studies performed.

Answer comment 2 of reviewer 3:

We would like to thank the reviewer for the pertinent comments. We collected three sets of biological replicates (harvested separately and independently on different days), with a standard of 100 embryos per set, and these numbers of replicates and embryos per replicate were derived from our previous research[6]. In those studies, we titrated a range of zygotes from 15 to 240 embryos (Figure 33a as below), and we were able to achieve strong correlations between the number of embryos and the MS signal for most of the metabolites even at lower input ranges of the gradients (Figure 33b as below), indicating good quantification of those metabolites with this approach. The number of three replicates for each stage of embryo was also proven to be sufficient to obtain consistent results with reasonable variability in those studies [6,46]. In this study, we have also made sure that the difference of metabolite abundance, particularly 5-HT, is statistically significant with a large number of replicates using other independent measuring method such as immunoimaging to corroborate our findings (Figure 34 as below, Fig.2e, f in the revised manuscript). We have added the discussion and statistical evidence in the revised manuscript on page 3 and Fig.1c-k, as the following: "Notably, oocytes displayed substantially higher levels of NAD+, NADH, taurine, serotonin (5-HT), glutathione (GSH), and vitamin C, all of which are known to play crucial roles in cellular redox balance and antioxidant defense. Conversely, 2-cell embryos exhibited elevated concentrations of pyruvate and succinate, reflecting shifts in energy metabolism as development progress".

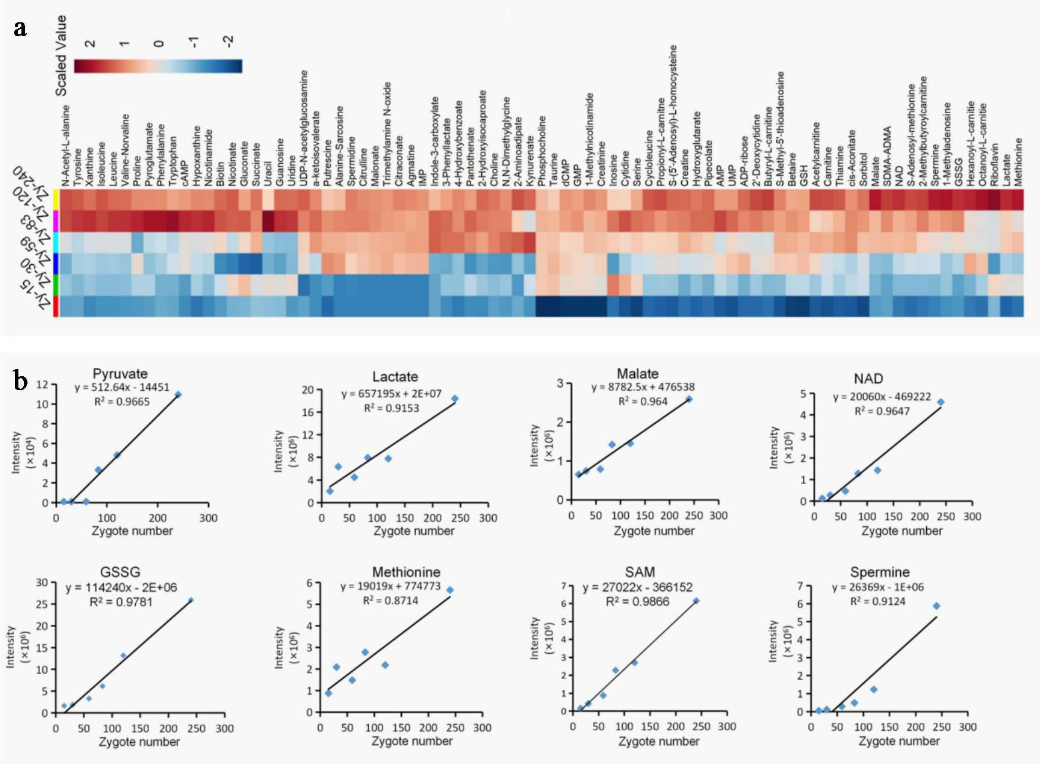

Figure 33

Figure 33: **a,** Metabolites from 15 to 240 zygotes were extracted and titrated for targeted metabolomics.

The heat map shows that relative metabolite abundance was normalized with MetaboAnalyst 4.0. Z. **b,** Representative metabolites were shown to correlate the number of embryos and relative mass spectrometry intensity obtained. R is the correlation coefficient[6].

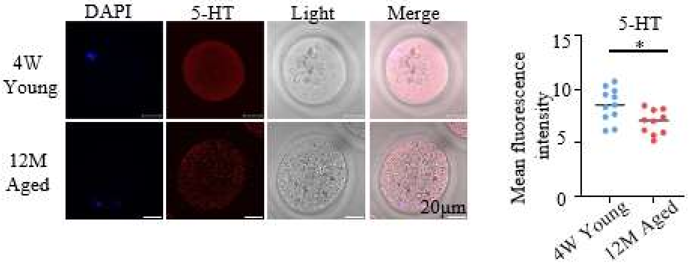

Figure 34

Figure 34: Immunofluorescence analysis and quantification of 5-HT of 4-week-old C57 and 12-month-old C57 MII oocytes. Scale bar, 20μm. *p<0.05, **p<0.01, ***p<0.001 according to the two-tailed unpaired t-test. n = 10-11 biological repeats.

Comment 3 of reviewer 3

• Page 10 – Authors mention serotonylation of HSP80β here as the targeted HSP protein – I think this is a typo. Also, is 5-HT really an anti-aging metabolite? Aside from this one

cell model more really needs to be done to confirm this – and this statement needs to be toned down.

Answer to comment 3 of reviewer 3:

We apologize for the typo in HSP80. We have changed it to HSP90β accordingly. We believe that 5-HT does have the effect of aging alleviation. **In fact, our study included two common models of cellular aging, pathological aging and replicative aging (natural aging), both *in vivo* and *ex vivo*.** You can refer to Methods for a more detailed description. In addition, we explored the effect of 5-HT in vivo in the progeria mouse model (shown in Fig.3l-o of the revised manuscript). Moreover, here in the revised manuscript, we further provide the effect of 5-HT **on naturally aged mice, as shown in Figure 35 below (**Fig.3p-s and Extended Data Fig.3n-q in the revised manuscript**).** However, we acknowledge that the statement needs to be toned down. In the revised version, we rephrased that the effect of 5-HT is that it can alleviate aging phenotypes, as the following, in the revised manuscript on pages 5-7.

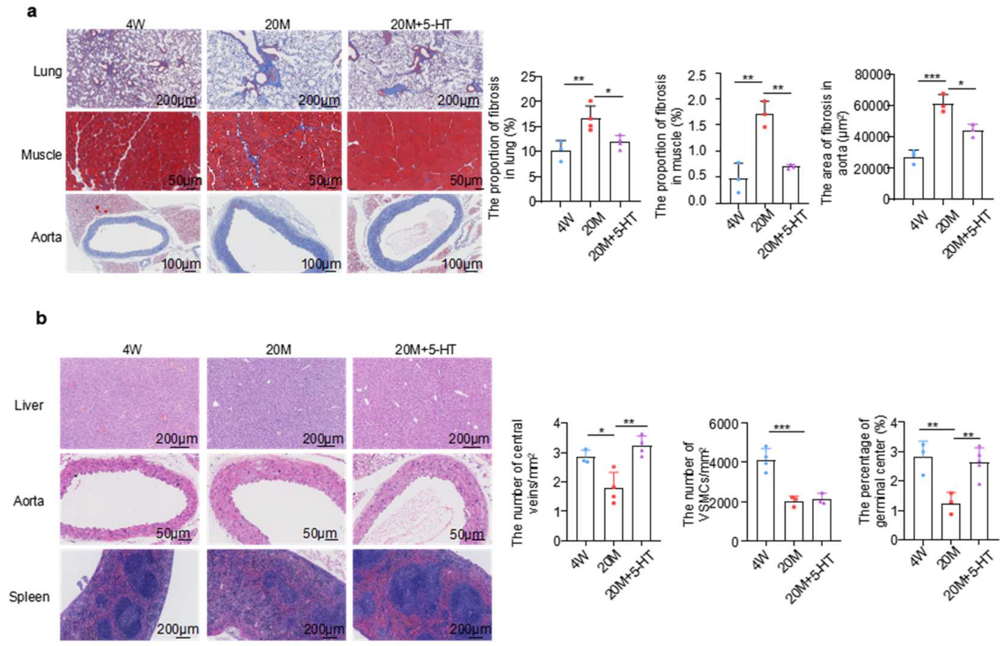

Figure 35

Figure 35: **a,** Masson staining and quantification of fibrosis area of lung, muscle, and aorta in **4-week-old and 20-month-old wild-type C57 mice** treated with 5-HT or not (from 12-month-old to 20-month-old). *p<0.05, **p<0.01, ***p<0.001 according to the one-way ANOVA test. n = 3 biological repeats. Scale bar, 200, 50 and 100μm. **b**, Histological analysis (HE) of the liver, spleen, and aorta of **4-week-old and 20-month-old wild-type C57 mice** treated with 5-HT or not (from 12-month-old to 20-month-old); Quantitative analysis of the number of central veins per unit area (mm^2) in the liver, the average VSMC number per unit area (mm^2) in the aorta and the percentage of germinal center area in the spleen. *p<0.05, **p<0.01, ***p<0.001 according to the one-way ANOVA test. n = 3 biological repeats. Scale bar, 200 and 50 μm.

Comment 4 of reviewer 3

• Page 15 – In the LC MS/MS methodology see no mention of QC samples being used – can the authors please explain. Also, there are precise details around the standards that were used in the analysis. This needs to be provided.

Answer to comment 4 of reviewer 3:

We would like to thank the reviewer for the valuable comments. Because the embryo samples were very precious, 10,000 FACS-sorted mouse cells instead were used as QC to monitor the stability of the instrument state during sample sequence collection. During the testing of this batch of samples including QC samples and embryo samples, the RSD (relative standard deviation) of more than 99% metabolites in the QC samples was less than 20%, indicating good instrument stability (Figure 36 as below and also in Extended Data Fig.1a of the revised manuscript).

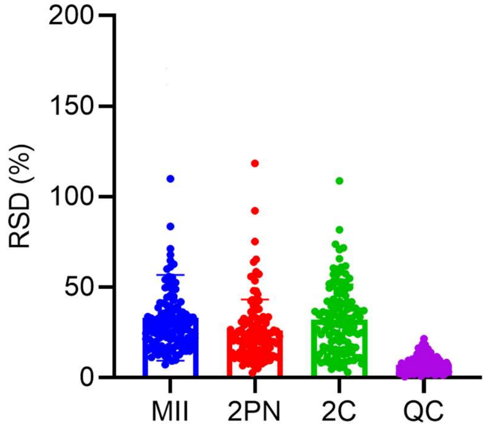

Figure 36

Figure 36: QC chart of 10,000 FACS-sorted mouse cells.

Comment 5 of reviewer 3

• Page 24 – The LC-MS/MS methodology mentions a homemade RP analytical column was used for the shotgun proteomics analysis. More details need to be provided around this. Information around stationary phase (particle size/gaps) where it was sourced and how it put together needs to be provided in order that readers can replicate the science.

Answer to comment 5 of reviewer 3:

We would like to thank the reviewer for the pertinent comments. A more detailed description of the LC-MS/MS methodology mentioning a homemade RP analytical column has been updated in the Methods on page 23 of the revised manuscript and also as the following: "The tryptic peptides were dissolved in solvent A, directly loaded onto a home-made reversed-phase analytical column (25-cm length, 100 μm i.d.). Home-made reversed-phase analytical column (100 μm i.d. × 25 cm) was packed with 1.9 μm/120 Å ReproSil-PurC18 resins (Dr. Maisch GmbH, Ammerbuch, Germany). The mobile phase consisted of solvent A (0.1% formic acid, 2% acetonitrile/in water) and solvent B (0.1% formic acid, 90%

acetonitrile/in water). Peptides were separated with the following gradient: 0-22.5 min，6%-22%B；22.5-26.5 min，22%-34%B；26.5-28.5 min，34%-80%B；28.5-30 min，80%B, and all at a constant flow rate of 700 nl/min on an EASY-nLC 1200 UPLC system (ThermoFisher Scientific).”

Comment 6 of reviewer 3

• Page 24 – Database Search section. There are several cases of ‘xx’ being used as a numerical value. I am thinking data is missing here – please provide or explain why it is not there.

Answer to comment 6 of reviewer 3:

We would like to thank the reviewer for the pertinent comments. The “xx” placeholders were indeed an oversight in our initial draft, where some points were inadvertently left unpopulated. Specific information about several "XX" has been completed in the method of LC-MS/MS for identifying target proteins bound to 5-hydroxytryptamine, as also shown as the following “The DIA data was processed using DIA-NN search engine (v.1.8). Tandem mass spectra were searched against the Homo_sapiens_9606_SP_20231220.fasta (20429 entries) concatenated with reverse decoy database. Trypsin/P was specified as a cleavage enzyme allowing up to 1 missing cleavage. Excision on N-term Met and carbamidomethyl on Cys were specified as fixed modifications. FDR was adjusted to < 1%.”

.

Comment 7 of reviewer 3

• Page 25 – Precise details (similar to the LC MS/MS section) need to be provided around how serotonylation was measured as this method will be markedly different than all other LC MS/MS methods described.

Answer to comment 7 of reviewer 3:

Thank you for your feedback regarding the measurement of serotonylation. We have added detailed information in the methods section on page 25 of the revised manuscript, including sample preparation, chromatographic conditions, mass spectrometry parameters, and data analysis techniques. These additions clarify how our approach differs from other LC-MS/MS methods. It is also shown here as the following: “

Sample preparation

The gel is cut into small pieces and decolorized using a solution of 25% acetonitrile (ACN) and 50 mM ammonium bicarbonate (ABC). Next, the gel pieces are dehydrated with ACN. The proteins are reduced by adding 5 mM dithiothreitol (DTT) and incubating at 37°C for 1 hour, followed by another dehydration step. Then, alkylation is performed with 11 mM iodoacetamide (IAM) for 45 minutes at room temperature in the dark.

After dehydration, 200 μl of trypsin solution (at a concentration of 10 ng/μl) is added and digestion is performed overnight. Subsequently, the peptides are eluted once with 150 μl of Elution Buffer I (50% ACN, 5% formic acid), followed by a single elution with 150 μl of Elution Buffer II (75% ACN, 0.1% formic acid), and then eluted twice with 150 μl of Elution Buffer III (100% ACN). The elutions are combined and dried down, then Stage-Tip desalting is performed. Finally, the peptide fragments are dissolved in 0.1% formic acid for analysis.

Tandem MS

MS experiments were performed on Q Exactive HF-X instrument (ThermoFisher) coupled

with an Easy-nLC 1200 system. Mobile phases A and B were water and 80% acetonitrile, respectively, with 0.1% formic acid. Digested peptides were loaded directly onto an analytical column (75 µm × 15 cm, 1.9 µm C18, 5 µm tip) at a flow rate of 300 nl min–1. All peptides samples were separated using a linear gradient of 4–6% phase B for 1 min, 6–26% B for 42 min, 26–35% B in 12 min, 35%-90% B for 2 min and 90% B for 3min. Survey scans of peptide precursors were performed from 400 to 1,400 m/z at 60,000 full width at half-maximum resolution with a 3×10^6 ion count target and a maximum injection time of 45 ms. The instrument was set to run in top-speed mode with 1-s cycles for the survey and the MS/MS scans. After a survey scan, tandem MS was then performed on the most abundant precursors exhibiting a charge state from 2 to 7 of greater than 1×10^5 intensity by isolating them in the quadrupole mode at 1.6 m/z. Higher energy collisional dissociation fragmentation was applied with 27% collision energy and resulting fragments were detected in the Orbitrap detector at a resolution of 15,000. The maximum injection time limit was 22 ms, and dynamic exclusion was set to 30 s with a 10-ppm mass tolerance around the precursor. Mass spectrometry data were searched by PD (ThermoFisher)."

Comment 8 of reviewer 3

• Page 15 and 26 – Pathway enrichment analysis and Figure 1. Real care needs to be taken when performing enrichment when a targeted list of metabolites is used - all of the following are critical points than need to be addressed in the fullest. It is noted that 262 metabolites are monitored – then 256 are measured. I am presuming that 6 of them are duplicated across +/- modes? This full list needs to be provided as a supplementary alongside full MRM transition m/z values and quad transition voltages (and excel will suffice). Alongside this, a statement needs to be provided around the concept of pathway bias contained within the 256 metabolites that are analyzed. It is highly possible that by including more metabolites in a specific pathway in your list, you are automatically preselecting those pathways in any enrichment analysis – please justify this approach. Also, in section J the p-value red gradient bar doesn't allow for any meaningful level of significance to be determined – this should be adjusted or the specific p-value levels noted on the figure (and any subsequent figures that are similar).

Answer to comment 8 of reviewer 3:

To clarify, when initially establishing this metabolomics methodology, we developed an analytical framework targeting 262 metabolites, as mentioned in our previous publications [6]. For metabolite identification, we optimized the parent/product ions of MRM transitions by injecting analytical standards into the MS spectrometer and determined retention times by injecting standards into the LC-MS system. Finally, 256 metabolites common and important metabolic pathways metabolites were in this method.

In our embryo samples specifically, we detected 124 metabolites (77 ion transitions in positive mode and 47 ion transitions in negative mode), which were included in the associated Excel file (Table S1). We have revised our language throughout the manuscript to refer to this as a "targeted metabolomics analysis" rather than a "comprehensive metabolomics landscape" to more accurately reflect the scale of our chemical profiling. Figure 1 in the article shows a total of 124 metabolites. The red gradient bar of Section J

has been adjusted. The full list includes metabolite data and a supplementary alongside full MRM transition m/z values and quad transition voltages that have been offered in the supplementary Table S1 in the revised manuscript
We acknowledge that there is certain level of bias for enrichment analysis using targeted metabolomics, therefore, we intentionally designed to cover major metabolic pathways, including central carbon metabolism, nucleotide metabolism, amino acid metabolism, one-carbon metabolism, and lipid metabolism, in order to minimize this bias. According to the reviewer’s suggestion, we would like to clarify this point and tune it down, and have added a statement on page 18 and also as the following: “We acknowledge that our targeted metabolomics approach is based on a preselected metabolite pool (n=124). However, this selection was carefully designed to cover major metabolic pathways, including central carbon metabolism, nucleotide metabolism, amino acid metabolism, one-carbon metabolism, and lipid metabolism. In addition, the metabolite pool was established based on previously published data capturing metabolites detectable in early embryonic tissues, oocytes, and embryonic stem cells [6]. By focusing on these well-characterized embryonic-relevant metabolites and pathways, we aimed to minimize potential bias while maintaining biological relevance to early development."
What’s more, thanks for your feedback on the p-value red gradient bar in section J. We’ve adjusted its range for a more appropriate display of significance levels (shown in Fig.1I of the revised manuscript).

Reference

1 Sahu, S. K. *et al.* Targeted partial reprogramming of age-associated cell states improves markers of health in mouse models of aging. *Science Translational Medicine* **16**, eadg1777, doi:10.1126/scitranslmed.adg1777 (2024).

2 Chondronasiou, D. *et al.* Multi-omic rejuvenation of naturally aged tissues by a single cycle of transient reprogramming. *Aging Cell* **21**, e13578, doi:10.1111/acel.13578 (2022).

3 Browder, K. C. *et al.* In vivo partial reprogramming alters age-associated molecular changes during physiological aging in mice. *Nature Aging*, doi:10.1038/s43587-022-00183-2 (2022).

4 Shinagawa, T. *et al.* Histone variants enriched in oocytes enhance reprogramming to induced pluripotent stem cells. *Cell Stem Cell* **14**, 217-227, doi:10.1016/j.stem.2013.12.015 (2014).

5 Sharpley, M. S., Chi, F., Hoeve, J. T. & Banerjee, U. Metabolic plasticity drives development during mammalian embryogenesis. *Dev Cell* **56**, 2329-2347 e2326, doi:10.1016/j.devcel.2021.07.020 (2021).

6 Zhao, J. *et al.* Metabolic remodelling during early mouse embryo development. *Nat Metab*, doi:10.1038/s42255-021-00464-x (2021).

7 Li, Y. *et al.* Vitamin C alleviates aging defects in a stem cell model for Werner syndrome. *Protein Cell* **7**, 478-488, doi:10.1007/s13238-016-0278-1 (2016).

8 Singh, P. *et al.* Taurine deficiency as a driver of aging. *Science* **380**, eabn9257, doi:10.1126/science.abn9257 (2023).

9 Tahiri, J. *et al.* Serotonin in depression and Alzheimer's disease: Focus on SSRI's

beneficial effects. *Ageing Research Reviews* **101**, 102537, doi:10.1016/j.arr.2024.102537 (2024).

10 Wu, Z. *et al.* Differential stem cell aging kinetics in Hutchinson-Gilford progeria syndrome and Werner syndrome. *Protein Cell* **9**, 333-350, doi:10.1007/s13238-018-0517-8 (2018).

11 Lopez-Otin, C., Blasco, M. A., Partridge, L., Serrano, M. & Kroemer, G. Hallmarks of aging: An expanding universe. *Cell* **186**, 243-278, doi:10.1016/j.cell.2022.11.001 (2023).

12 Lopez-Otin, C., Blasco, M. A., Partridge, L., Serrano, M. & Kroemer, G. The hallmarks of aging. *Cell* **153**, 1194-1217, doi:10.1016/j.cell.2013.05.039 (2013).

13 Frobel, J. *et al.* Epigenetic rejuvenation of mesenchymal stromal cells derived from induced pluripotent stem cells. *Stem Cell Reports* **3**, 414-422, doi:10.1016/j.stemcr.2014.07.003 (2014).

14 Conboy, I. M. *et al.* Rejuvenation of aged progenitor cells by exposure to a young systemic environment. *Nature* **433**, 760-764 (2005).

15 Liu, G.-H. *et al.* Recapitulation of premature ageing with iPSCs from Hutchinson-Gilford progeria syndrome. *Nature* **472**, 221-225, doi:10.1038/nature09879 (2011).

16 Herr, N., Bode, C. & Duerschmied, D. The Effects of Serotonin in Immune Cells. *Front Cardiovasc Med* **4**, 48, doi:10.3389/fcvm.2017.00048 (2017).

17 Flanagan, T. W. & Nichols, C. D. Psychedelics as anti-inflammatory agents. *Int Rev Psychiatry* **30**, 363-375, doi:10.1080/09540261.2018.1481827 (2018).

18 Ahern, G. P. 5-HT and the immune system. *Curr Opin Pharmacol* **11**, 29-33, doi:10.1016/j.coph.2011.02.004 (2011).

19 Luo, W., Sun, W., Taldone, T., Rodina, A. & Chiosis, G. Heat shock protein 90 in neurodegenerative diseases. *Mol Neurodegener* **5**, 24, doi:10.1186/1750-1326-5-24 (2010).

20 Janssens, G. E. *et al.* Transcriptomics-Based Screening Identifies Pharmacological Inhibition of Hsp90 as a Means to Defer Aging. *Cell Rep* **27**, 467-480 e466, doi:10.1016/j.celrep.2019.03.044 (2019).

21 Tesch, G. H., Ma, F. Y., Ozols, E. & Nikolic-Paterson, D. J. Intervention treatment reducing cellular senescence inhibits tubulointerstitial fibrosis in diabetic mice following acute kidney injury. *Clin Sci (Lond)* **138**, 309-326, doi:10.1042/CS20231698 (2024).

22 Sibille, E. *et al.* Lack of serotonin1B receptor expression leads to age-related motor dysfunction, early onset of brain molecular aging and reduced longevity. *Mol Psychiatry* **12** (2007).

23 Bader, M. Serotonylation: Serotonin Signaling and Epigenetics. *Front Mol Neurosci* **12**, 288, doi:10.3389/fnmol.2019.00288 (2019).

24 Walther, D. J. *et al.* Serotonylation of small GTPases is a signal transduction pathway that triggers platelet alpha-granule release. *Cell* **115**, 851-862 (2003).

25 Lorand, L. & Conrad, S. M. Transglutaminases. *Mol Cell Biochem* **58** (1984).

26 Lorand, L. & Graham, R. M. Transglutaminases: crosslinking enzymes with pleiotropic functions. *Nature Reviews. Molecular Cell Biology* **4**, 140-156 (2003).

27 Goldman, R. D. *et al.* Accumulation of mutant lamin A causes progressive changes

in nuclear architecture in Hutchinson-Gilford progeria syndrome. *Proceedings of the National Academy of Sciences of the United States of America* **101**, 8963-8968 (2004).

28 McGrath, J. & Solter, D. Inability of mouse blastomere nuclei transferred to enucleated zygotes to support development in vitro. *Science (New York, N.Y.)* **226**, 1317-1319 (1984).

29 Greda, P., Karasiewicz, J. & Modlinski, J. A. Mouse zygotes as recipients in embryo cloning. *Reproduction* **132**, 741-748 (2006).

30 Kanova, M. & Kohout, P. Serotonin-Its Synthesis and Roles in the Healthy and the Critically Ill. *International Journal of Molecular Sciences* **22**, doi:10.3390/ijms22094837 (2021).

31 Collins, C. M., Kloek, J. & Elliott, J. M. Parallel changes in serotonin levels in brain and blood following acute administration of MDMA. *J Psychopharmacol* **27**, 109-112, doi:10.1177/0269881112463123 (2013).

32 Mordhorst, A. *et al.* Phenylalanine hydroxylase contributes to serotonin synthesis in mice. *FASEB Journal : Official Publication of the Federation of American Societies For Experimental Biology* **35**, e21648, doi:10.1096/fj.202100366R (2021).

33 Zhao, S. & Piatkevich, K. D. Techniques for in vivo serotonin detection in the brain: State of the art. *J Neurochem* **166**, 453-480, doi:10.1111/jnc.15865 (2023).

34 Scaffidi, P. & Misteli, T. Lamin A-dependent nuclear defects in human aging. *Science (New York, N.Y.)* **312**, 1059-1063 (2006).

35 McClintock, D. *et al.* The mutant form of lamin A that causes Hutchinson-Gilford progeria is a biomarker of cellular aging in human skin. *PLoS One* **2**, e1269 (2007).

36 Cao, K. *et al.* Progerin and telomere dysfunction collaborate to trigger cellular senescence in normal human fibroblasts. *The Journal of Clinical Investigation* **121**, 2833-2844, doi:10.1172/JCI43578 (2011).

37 Liu, X. *et al.* Resurrection of endogenous retroviruses during aging reinforces senescence. *Cell* **186**, doi:10.1016/j.cell.2022.12.017 (2023).

38 Liu, Z. *et al.* Cross-species metabolomic analysis identifies uridine as a potent regeneration promoting factor. *Cell Discov* **8**, 6, doi:10.1038/s41421-021-00361-3 (2022).

39 Ramirez, M. J., Lai, M. K. P., Tordera, R. M. & Francis, P. T. Serotonergic therapies for cognitive symptoms in Alzheimer's disease: rationale and current status. *Drugs* **74**, 729-736, doi:10.1007/s40265-014-0217-5 (2014).

40 Mattson, M. P., Maudsley, S. & Martin, B. BDNF and 5-HT: a dynamic duo in age-related neuronal plasticity and neurodegenerative disorders. *Trends Neurosci* **27**, 589-594 (2004).

41 Mattson, M. P., Maudsley, S. & Martin, B. A neural signaling triumvirate that influences ageing and age-related disease: insulin/IGF-1, BDNF and serotonin. *Ageing Research Reviews* **3**, 445-464 (2004).

42 Blier, P. & El Mansari, M. Serotonin and beyond: therapeutics for major depression. *Philos Trans R Soc Lond B Biol Sci* **368**, 20120536, doi:10.1098/rstb.2012.0536 (2013).

43 Okamura, F. *et al.* Insulin resistance in patients with depression and its changes

during the clinical course of depression: minimal model analysis. *Metabolism* **49**, 1255-1260 (2000).

44 Pinilla, E. *et al.* Transglutaminase 2 Inhibitor LDN 27219 Age-Dependently Lowers Blood Pressure and Improves Endothelium-Dependent Vasodilation in Resistance Arteries. *Hypertension* **77**, 216-227, doi:10.1161/HYPERTENSIONAHA.120.15352 (2021).

45 Wang, X. *et al.* A GAPDH serotonylation system couples CD8(+) T cell glycolytic metabolism to antitumor immunity. *Mol Cell*, doi:10.1016/j.molcel.2023.12.015 (2024).

46 Zhang, L. *et al.* Low-input lipidomics reveals lipid metabolism remodelling during early mammalian embryo development. *Nature Cell Biology* **26**, 278-293, doi:10.1038/s41556-023-01341-3 (2024).

Dear Dr Zhang,

Thank you again for the submission of your amended manuscript (EMBOJ-2025-123120-T) to The EMBO Journal. We have carefully assessed your manuscript, and the point-by-point response provided to the referee concerns that were raised before during review at a different journal. In addition, and as mentioned before, we decided to send the revised version of your work back to the original reviewer #3 as well as an independent arbitrating advisor for reassessment with respect to technical robustness, and overall suitability of your work for publication in The EMBO Journal. We have now received their feedback which I enclose below. As you will see, the experts are in favour of the work and supportive of publication at The EMBO Journal, pending satisfactory minor revision.

We are thus pleased to inform you that we can swiftly move forward towards acceptance of this work at The EMBO Journal, pending adequate minor amendment of the study.

Please consider the remaining points by the experts carefully by
>> better annotating mesenchymal stem cell populations characterised (Arbitrating expert)
>> adjusting the discussion of the results in light of potential contributing parameters, introducing caveats and amendments where appropriate (former Referee #3).

We also now need you to take care of a number of minor points related to formatting and data annotation, which I will share shortly in a separate message, together with additional changes and requests by our production team for Source Data provision.

Please submit a revised version of the manuscript using the link enclosed below, addressing the advisor's comments.

As you might have seen on our web page, every paper at the EMBO Journal now includes a 'Synopsis', displayed on the html and freely accessible to all readers. The synopsis includes a 'model' figure as well as 2-5 one-short-sentence bullet points that summarize the article. I would appreciate if you could provide this figure and the bullet points.

Please let me know any time should you have additional questions regarding above points.

Thank you again for giving us the chance to consider your manuscript for The EMBO Journal, I look forward to hearing from you and receiving your final revised version of the manuscript.

Best regards,

Daniel Klimmeck

Daniel Klimmeck PhD
Senior Editor
The EMBO Journal

****

Please remember: Digital image enhancement is acceptable practice, as long as it accurately represents the original data and conforms to community standards. If a figure has been subjected to significant electronic manipulation, this must be noted in the figure legend or in the 'Methods' section. The editors reserve the right to request original versions of figures and the original images that were used to assemble the figure.

EMBOJ-2025-123120-T, Former Referee #3's additional comments:

This study addresses an important and timely question around whether metabolites enriched in early developmental stages, particularly serotonin, contribute to cellular and organismal ageing phenotypes through defined molecular mechanisms. By combining targeted metabolomics of mouse oocytes and early embryos with cellular, proteomic and in vivo ageing models, the authors seek to link developmental metabolic states with resilience to ageing, acting through both canonical receptor dependent pathways and serotonylation based regulation of HSP90β. The conceptual framework is novel and potentially impactful, and the breadth of experimental systems employed is impressive.

However, several minor issues relating to experimental design, statistical inference, metabolomic scope and interpretation currently weaken the strength of the conclusions. In particular, aspects of the metabolomics design and its downstream interpretation require clearer justification and more precise framing to ensure that the claims made are commensurate with the data presented.
An unresolved concern relates to the pooling strategy used for the metabolomics analysis, specifically the use of 100 embryos per biological replicate without adequate statistical justification. The manuscript does not provide power calculations, variance estimates or effect size reasoning to justify this approach, nor does it clearly acknowledge how pooling limits inference. In addition, pooled samples are implicitly treated as equivalent to biological replication at the level of individual embryos, despite the absence of statistical discussion of biological variability across embryos. Although instrument quality control metrics are reported, biological variability is not formally addressed, and the biological unit of inference, whether the pooled sample or the individual embryo, is not explicitly defined in either the Results or Discussion. Without clearer justification of the pooling strategy and transparent acknowledgment of its limitations, the strength and generalisability of the conclusions drawn from the metabolomics analysis remain a little difficult to fully assess.

With respect to the interpretation of pathway enrichment analysis, although the authors acknowledge the inherent pathway bias associated with targeted metabolomics, the subsequent interpretation remains overstretched. Enrichment results continue to be visually and rhetorically presented as discovery level biological findings rather than as conditional analyses that are constrained by the composition of the targeted panel. In addition, no quantitative sensitivity analyses or alternative framing are provided to demonstrate robustness to metabolite selection. Greater care is therefore required to ensure that pathway enrichment is clearly positioned as descriptive and hypothesis supporting, rather than as evidence of unbiased pathway discovery.

Overall, the paper is a significant discovery and by addressing the comments above will be a strong addition to the ageing metabolism literature.

EMBOJ-2025-123120-T, arbitrating advisor's comments:

I took a while to get through all the material. So my main concern was the use of an MSC model, to address the anti-aging mechanism, the paper seemed a bit schizophrenic. And MSCs are not a single cell type...However, having gone through everything, I think the part on the systemic aging is actually looking reasonable.

I think the paper actually does contain a strong message and they have gone a long way to address the comments. Perhaps they could make a bit more effort to clean up the MSCs and qualify exactly what progenitor population they are assessing as "aged MSCs.

Dear Dr Zhang,

Further to below, I attach the mentioned list of formatting changes required for your final resubmission.

Please let us know any time should you have additional questions related.

Best regards,

Daniel Klimmeck

Daniel Klimmeck PhD
Senior Editor
The EMBO Journal

******

Formatting changes required for the revised version of the manuscript:

>> Author information: Please add an author account for Q. Wu in our system. Please replace the email address for the co-corresponding author L. Z. with the institutional email address.

>> Author Contributions: Remove the author contributions information from the manuscript text. Note that CRediT has replaced the traditional author contributions section as of now because it offers a systematic machine-readable author contributions format that allows for more effective research assessment. and use the free text boxes beneath each contributing author's name to add specific details on the author's contribution.

More information is available in our guide to authors.
https://www.embopress.org/page/journal/14602075/authorguide

>> Adjust the title of the 'Competing Interests' section to 'Disclosure and Competing Interests Statement'

>> Please correct the order and the heading of the sections in the manuscript to: Abstract / The Paper Explained / Introduction / Results / Discussion / Methods / Data Availability / Acknowledgements / Disclosure and Competing Interests Statement / References / Figure Legends / Tables / Expanded View Figure Legends

>> Add a separate 'Statistical Analysis' section to the Methods part, detailing the algorithms and statistical tests applied.

>> References: please adjust reference format to EMBO Journal format, 10 authors et al.

>> Please add a Reagents and Tools table to the Methods section, as a separate file using the existing template in the Guide For Authors, listing key reagents, experimental models, software and relevant equipment.

Please compare also our Guide-to-Authors instructions:
https://www.embopress.org/page/journal/14602075/authorguide#structuredmethods

>> Funding: Merge with the Acknowledgments and add the Natural Science Foundation of Jiangsu Province (Grants No BK20210150 to W.G) and the National Natural Science

Foundation of China (82300800 to W.G.) to the list of funders in our system.

>> Dataset EV legends: Rename Tables S1 - S4 "Dataset EV1" - "Dataset EV4" and add a legend to each excel file, in a separate tab/worksheet.

>> Please provide source data for the study as to the separate request e-mail. Source data should be uploaded as one (zipped) file per figure.

>> Provide a completed Author Checklist.

>> Add complete annotation of human consent annotation to the Methods and adjust the Author Checklist sections Ethics and Data Availability accordingly.

>> Data availability section: specify all databases used (CNBC) and add hyperlinks.
Remove referee tokens and make sure datasets are made publicly accessible.

>> Please indicate redisplay of data from in Extended Data Fig 2. between B and H in the figure legend of Extended Data Fig 2H.

>> Figure callouts: There is a callout for a Table 1 but there is no such table. Callouts for the Tables S1-4/Dataset EV1-4 are missing, please add.

>> Figures in separate files: Move the figure legends to the manuscript text and upload the figures are separate high res figure files. Rename the supplementary figures "Figure EV1" - "Figure EV9". The legends should also be placed in the manuscript text, after the main figure legends and under the heading "Expanded View Figure Legends".

>> Consider additional changes and comments from our production team as indicated below:

- DAS:
1. Please note that the specific URLs for GSE268560, OMIX011107 and OMIX011121 datasets are not provided in the data availability statement.
2. Please note that reviewer access codes for GSE268560, OMIX011107 and OMIX011121 datasets are not provided in the data availability statement.
- Figure legends:
1. Please define the annotated p values ****/***/**/* as well as provide the exact p-values for the same in the legend of figure 3G as appropriate.
2. Please note that the exact p values are not provided in the legends of figures 1D, E, F, G, I, J, K; 2A-D, F, j, k; 3B, C, D, F, G, H, I-K, M-O, Q-S; 4C, D, F, G, I; 5F, G; 6A, B, C, F; 7A, B; EDF 1G, EDF 3B, E, F, G, I, J, K, M, O, P, Q; EDF 4A-D; EDF 5F-H; EDF 8A-E; EDF 9D, E
3. Please indicate the statistical test used for data analysis in the legends of figures 1C, I; 4B, 5A, B; EDF 1D, EDF 6 B-D
4. Please note that information related to n is missing in the legends of figures 1C, EDF 5D
5. Please note that the arrow heads are not defined in the legend of figure 2G. This needs to be rectified.
6. Please note that the red arrows are not defined in the legend of EDF 2G. This needs to be rectified.

Further information is available in our Guide For Authors: https://link.springer.com/journal/44318/submission-guidelines

Response to the Editors and Reviewers

We thank the editors, the arbitrating expert, and the reviewers for their constructive and insightful comments, which have helped us to clarify and strengthen the manuscript entitled:
"Oocyte-enriched metabolite serotonin-inspired aging alleviation strategy through serotonylation of HSP90β"by Yuyan Xu et al. In this revised version, we have (i) more clearly annotated and defined the mesenchymal stem cell (MSC) populations used in our study, and (ii) revised the Discussion to explicitly limitations, and to appropriately soften statements regarding translational application.

All changes are indicated in the revised manuscript, and major textual additions are quoted in the responses below. Page and line numbers refer to the revised manuscript.

1. Arbitrating expert: "better annotating mesenchymal stem cell populations characterised"
Response: *We appreciate this constructive suggestion, we have clarified the origin and characterization of the MSC populations throughout the manuscript:*
Results section: we have added the sentence "To investigate the potential role of oocyte-derived metabolites in counteracting cellular aging, we established an experimental model using mesenchymal-like stem cells (MSCs) differentiated from human induced pluripotent stem cell(iPSCs) (CD73^+/CD90^+/CD105^+/CD45^-; see Extended Data Fig. 2), hereafter referred to as iPSC-derived MSCs." in the revised text on page 5, lines 122–126.
Methods section: we have added a concise paragraph describing their marker-based characterization and references in the revised text on page 16, lines 475–480.
Discussion section: we have discussed the limitations regarding the cellular and mouse model used in our study, the sentence"Our conclusions are primarily based on iPSC-derived MSCs and mouse models, which may not fully recapitulate the complexity of human tissue aging in vivo. iPSC-derived MSCs represent a developmentally reset and culture-adapted cell population, and their epigenetic and metabolic states may differ from those of primary tissue-resident MSCs." are added in the revised text on page14, lines 403–406..
Terminology clarified: "WT MSCs" and "G608G MSCs" refer to iPSC-derived MSC populations generated from healthy donors and Hutchinson Gilford Progeria Syndrome (HGPS) patients, respectively. The sentence are added in the revised text on page 5, lines 126-128.
Figure legends: Updated to include the origin and marker profile of MSCs. Revised in Figure EV2.
All together, these changes provide clearer and more accurate annotation of the MSC populations without over claiming characterization that was not performed.

2. Former Referee #3: This study addresses an important and timely question around whether metabolites enriched in early developmental stages, particularly serotonin, contribute to cellular and organismal ageing phenotypes through defined molecular mechanisms. By combining targeted metabolomics of mouse oocytes and early embryos with cellular, proteomic and in vivo ageing models, the authors seek to link developmental metabolic states with resilience to ageing, acting through both canonical receptor dependent pathways and serotonylation based regulation of HSP90β. The conceptual framework is novel and potentially impactful, and the breadth of

experimental systems employed is impressive.

Response: *We highly appreciate the reviewer's strongly positive comments that our study is "novel and potentially impactful", and the comments of our experiments systems employed is "impressive".*

However, several minor issues relating to experimental design, statistical inference, metabolomic scope and interpretation currently weaken the strength of the conclusions. In particular, aspects of the metabolomics design and its downstream interpretation require clearer justification and more precise framing to ensure that the claims made are commensurate with the data presented.

An unresolved concern relates to the pooling strategy used for the metabolomics analysis, specifically the use of 100 embryos per biological replicate without adequate statistical justification. The manuscript does not provide power calculations, variance estimates or effect size reasoning to justify this approach, nor does it clearly acknowledge how pooling limits inference. In addition, pooled samples are implicitly treated as equivalent to biological replication at the level of individual embryos, despite the absence of statistical discussion of biological variability across embryos. Although instrument quality control metrics are reported, biological variability is not formally addressed, and the biological unit of inference, whether the pooled sample or the individual embryo, is not explicitly defined in either the Results or Discussion. Without clearer justification of the pooling strategy and transparent acknowledgment of its limitations, the strength and generalisability of the conclusions drawn from the metabolomics analysis remain a little difficult to fully assess.

Response： *Thanks for the reviewer's suggestion. In our previous study, we have already tested the range of optimized ultralow-input metabolomics approach (PMID:34650276,PMID:38302721), specifically, we titrated a range of mouse ES cells from 2500 to 80000 cells or a range of zygotes from 15 to 240 embryos, we have obtained strong correlations between the number of cells/embryos and the MS signa for the most of the metabolites, indicating good quantification of those metabolites with this approach(Figure.A-C). We thus collected 100 embryos as our low-input metabolomics study. Besides, in our lipidomics study, the PCA results of the n =3 and n=7 biological replicates are highly comparable(Figure.D,E), so that the effect size n=3 is reasonable. In addition, there are two papers showing clustering of different embryo development stages using metabolomics data or metabolic gene transcriptome data (PMID:34428339,PMID:36167681), the within-stage variance all exists(Figure. F,G). We have added more description about the variance among replicates across embryos, and we explicitly state that our metabolomics measurements were obtained from pooled samples of 100 oocytes or embryos per biological replicate. What's more , we have added the sentence: "It is important to note that our metabolomics measurements were obtained from pooled samples of 100 oocytes or embryos per replicate. While this approach is necessary for early-embryo metabolomics due to extremely low per-cell metabolite abundance, it does not capture embryo-to-embryo variability and limits inference to population-level metabolic trends. These data therefore support broad metabolic differences between stages, but do not provide variance estimates at the level of individual embryos" in the revised text on page 12-13， lines 368-373.*

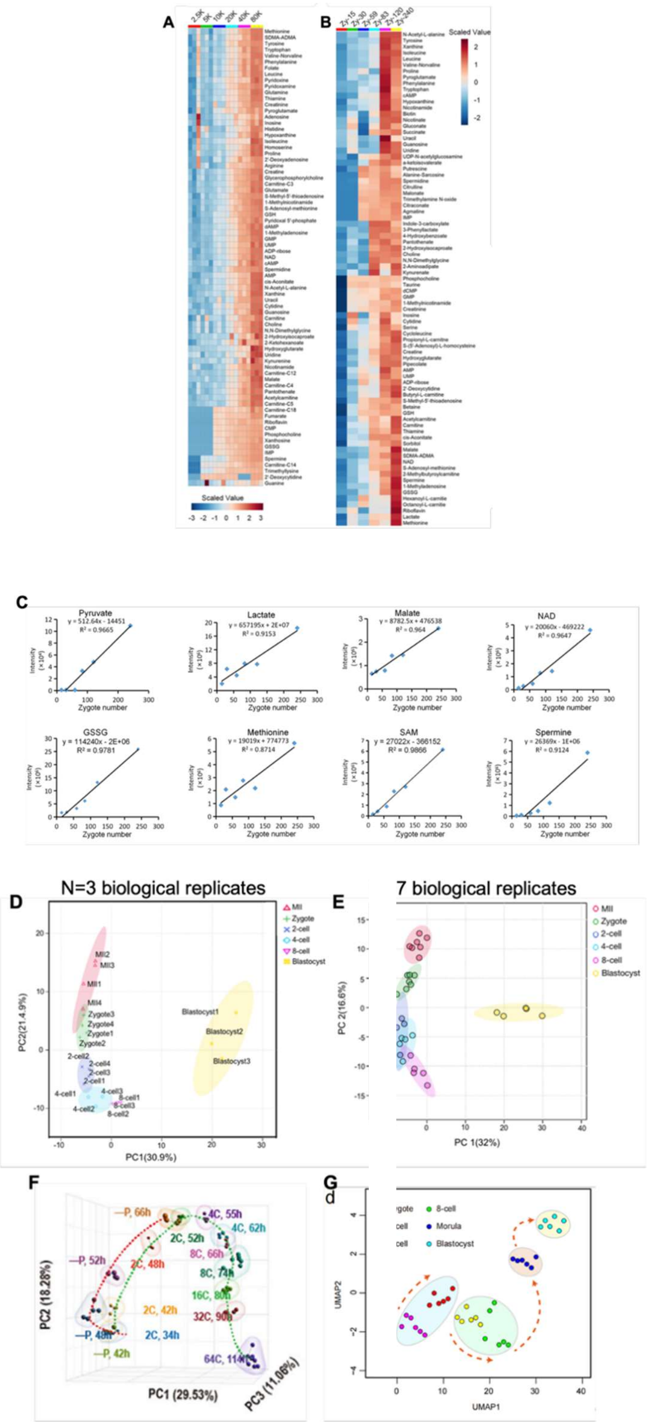

Figure: A-B: Metabolites from 2.5 K to 80 K mouse embryonic stem cells or 15 to 240 zygotes were extracted and titrated for targeted metabolomics.Heatmap showing relative metabolite abundance normalized with MetaboAnalyst 4.0. Zy: zygote.C: Representative metabolites were shown for correlation between the number of embryos and relative mass spectrometry intensity obtained. R is the correlation coefficient. D-E:PCA plot of n=3 biological replicates and n=7 biological replicates in our previous published paper. F-G: PCA plot showing the variance among replicates across embryos in literatures.

With respect to the interpretation of pathway enrichment analysis, although the authors acknowledge the inherent pathway bias associated with targeted metabolomics, the subsequent interpretation remains overstretched. Enrichment results continue to be visually and rhetorically presented as discovery level biological findings rather than as conditional analyses that are constrained by the composition of the targeted panel. In addition, no quantitative sensitivity analyses or alternative framing are provided to demonstrate robustness to metabolite selection. Greater care is therefore required to ensure that pathway enrichment is clearly positioned as descriptive and hypothesis supporting, rather than as evidence of unbiased pathway discovery.

Overall, the paper is a significant discovery and by addressing the comments above will be a strong addition to the ageing metabolism literature."

Response: *Thanks for this suggestions, We have emphasized that enriched pathways are viewed as hypothesis-supporting rather than discovery-level evidence, we have added more discussion about enrichment metabolic pathway analysis, the sentence: "What' s more, the pathway enrichment analysis in this study is descriptive and intrinsically constrained by the targeted metabolite panel. Therefore, enriched pathways should be viewed as hypothesis-supporting rather than discovery-level evidence, and further unbiased metabolomic profiling will be needed to confirm these trends" are added in the revised text on page13, lines 373-377. As for the metabolites selection, we chose the metabolites based on the comparison of their relative abundance in oocytes, zygotes and 2-cell stage embryos, p value is less than 0.05, besides, the volcano plot showing* log_2 *fold change (FC) values between oocyte and 2-cell embryos and P value for the comparison of each individual metabolite is less than 0.05 by metaboanalyst 4.0.*

And here are the review access details for the original datasets:

OMIX011107 (https://ngdc.cncb.ac.cn/omix/preview/GwvXg4Iy)

*OMIX011121 (*https://ngdc.cncb.ac.cn/omix/preview/5N3daW16*)*

*OMIX016492 (*https://ngdc.cncb.ac.cn/omix/preview/l0zYfAFV*)*

For GEO record GSE268560 (private), the secure reviewer token is ojclmkcgtvgxpwr.

Dear Dr Zhang,

Thank you for submitting the revised version of your manuscript. I have now evaluated your amended manuscript and concluded that the remaining minor concerns have been sufficiently addressed.

I am thus pleased to inform you that your manuscript has been accepted for publication in the EMBO Journal.

Your manuscript will be processed for publication by EMBO Press. It will be copy edited and you will receive page proofs prior to publication. Please note that you will be contacted by Springer Nature Author Services to complete licensing and payment information.

Please note that it is The EMBO Journal policy for the transcript of the editorial process (containing referee reports and your response letter) to be published as an online supplement to each paper. If you do NOT want this, you will need to inform the Editorial Office via email immediately. More information is available here: https://www.embopress.org/transparent-process#Review_Process

You may qualify for financial assistance for your publication charges - either via a Springer Nature fully open access agreement or an EMBO initiative. Check your eligibility: https://www.embopress.org/page/journal/14602075/authorguide#chargesguide

Should you be planning a Press Release on your article, please notify the production team via this form in order to coordinate publication and release dates: https://forms.cloud.microsoft/e/eH4A8JVP6g

If you have any questions, please do not hesitate to contact the Editorial Office. Thank you for your contribution to The EMBO Journal.

Thank you again for this contribution to The EMBO Journal and congratulations on a successful publication! Please consider us again in the future for your most exciting work.

Best regards,

Daniel Klimmeck

Daniel Klimmeck, PhD
Senior Editor
The EMBO Journal
EMBO
Postfach 1022-40
Meyerhofstrasse 1
D-69117 Heidelberg
contact@embojournal.org

*****
Please note that it is The EMBO Journal policy for the transcript of the editorial process (containing referee reports and your response letters) to be published as an online supplement to each paper. If you should prefer removal of any referee-only figures included in the point-by-point response(s), e.g. because they may still be used for future publication or because they have been reproduced from published work by others, please do let us know immediately via response email.
More information is available here: https://link.springer.com/partners/embo-press/editorial-policies#Peer%20review

EMBO Press Author Checklist

Corresponding Author Name: Jin Zhang	
Journal Submitted to: The EMBO Journal	
Manuscript Number: EMBOJ-2025-123120R	

USEFUL LINKS FOR COMPLETING THIS FORM
The EMBO Journal - Author Guidelines
EMBO Reports - Author Guidelines
Molecular Systems Biology - Author Guidelines
EMBO Molecular Medicine - Author Guidelines

Reporting Checklist for Life Science Articles (updated January
This checklist is adapted from Materials Design Analysis Reporting (MDAR) Checklist for Authors. MDAR establishes a minimum set of requirements in transparent reporting in the life sciences (see Statement of Task: 10.31222/osf.io/9sm4x). Please follow the journal's guidelines in preparing your
Please note that a copy of this checklist will be published alongside your article.

Abridged guidelines for figures
1. Data
The data shown in figures should satisfy the following conditions:
- the data were obtained and processed according to the field's best practice and are presented to reflect the results of the experiments in an accurate and unbiased manner.
- ideally, figure panels should include only measurements that are directly comparable to each other and obtained with the same assay.
- plots include clearly labeled error bars for independent experiments and sample sizes. Unless justified, error bars should not be shown for technical
- if n<5, the individual data points from each experiment should be plotted. Any statistical test employed should be justified.
- Source Data should be included to report the data underlying figures according to the guidelines set out in the authorship guidelines on Data

2. Captions
Each figure caption should contain the following information, for each panel where they are relevant:
- a specification of the experimental system investigated (eg cell line, species name).
- the assay(s) and method(s) used to carry out the reported observations and measurements.
- an explicit mention of the biological and chemical entity(ies) that are being measured.
- an explicit mention of the biological and chemical entity(ies) that are altered/varied/perturbed in a controlled manner.
- the exact sample size (n) for each experimental group/condition, given as a number, not a range;
- a description of the sample collection allowing the reader to understand whether the samples represent technical or biological replicates (including how many animals, litters, cultures, etc.).
- a statement of how many times the experiment shown was independently replicated in the laboratory.
- definitions of statistical methods and measures:
  - common tests, such as t-test (please specify whether paired vs. unpaired), simple χ2 tests, Wilcoxon and Mann-Whitney tests, can be unambiguously identified by name only, but more complex techniques should be described in the methods section;
  - are tests one-sided or two-sided?
  - are there adjustments for multiple comparisons?
  - exact statistical test results, e.g., P values = x but not P values < x;
  - definition of 'center values' as median or average;
  - definition of error bars as s.d. or s.e.m.

Please complete ALL of the questions below.
Select "Not Applicable" only when the requested information is not relevant for your study.

Materials

Newly Created Materials	Information included in the manuscript?	In which section is the information available? (Reagents and Tools Table, Materials and Methods, Figures, Data Availability Section)
New materials and reagents need to be available; do any restrictions apply?	Not Applicable	

Antibodies	Information included in the manuscript?	In which section is the information available? (Reagents and Tools Table, Materials and Methods, Figures, Data Availability Section)
For **antibodies** provide the following information:<br>- Commercial antibodies: RRID (if possible) or supplier name, catalogue number and or/clone number<br>- Non-commercial: RRID or citation	Yes	Reagents and Tools Table

DNA and RNA sequences	Information included in the manuscript?	In which section is the information available? (Reagents and Tools Table, Materials and Methods, Figures, Data Availability Section)
**Short novel DNA or RNA including primers, probes**: provide the sequences.	Yes	Reagents and Tools Table

Cell materials	Information included in the manuscript?	In which section is the information available? (Reagents and Tools Table, Materials and Methods, Figures, Data Availability Section)
**Cell lines:** Provide species information, strain. Provide accession number in repository **OR** supplier name, catalog number, clone number, and/**OR** RRID.	Yes	Reagents and Tools Table
**Primary cultures:** Provide species, strain, sex of origin, genetic modification status.	Not Applicable	
Report if the cell lines were recently **authenticated** (e.g., by STR profiling) and tested for mycoplasma contamination.	Yes	The cell lines were routinely tested and confirmed mycoplasma-free. Authentication by STR was performed by the supplier or previously validated.

Experimental animals	Information included in the manuscript?	In which section is the information available? (Reagents and Tools Table, Materials and Methods, Figures, Data Availability Section)
**Laboratory animals or Model organisms:** Provide species, strain, sex, age, genetic modification status. Provide accession number in repository **OR** supplier name, catalog number, clone number, **OR** RRID.	Yes	Reagents and Tools Table
**Animal observed in or captured from the field:** Provide species, sex, and age where possible.	Not Applicable	
Please detail **housing and husbandry conditions**.	Yes	Materials and Methods

Plants and microbes	Information included in the manuscript?	In which section is the information available? (Reagents and Tools Table, Materials and Methods, Figures, Data Availability Section)
**Plants:** provide species and strain, ecotype and cultivar where relevant, unique accession number if available, and source (including location for collected wild specimens).	Not Applicable	
**Microbes:** provide species and strain, unique accession number if available, and source.	Not Applicable	

Human research participants	Information included in the manuscript?	In which section is the information available? (Reagents and Tools Table, Materials and Methods, Figures, Data Availability Section)
If collected and within the bounds of privacy constraints report on age, sex and gender or ethnicity for all study participants.	Not Applicable	

Core facilities	Information included in the manuscript?	In which section is the information available? (Reagents and Tools Table, Materials and Methods, Figures, Data Availability Section)
If your work benefited from core facilities, was their service mentioned in the acknowledgments section?	Yes	Materials and Methods

Design

Study protocol	Information included in the manuscript?	In which section is the information available? (Reagents and Tools Table, Materials and Methods, Figures, Data Availability Section)
If study protocol has been **pre-registered, provide DOI in the manuscript**. For clinical trials, provide the trial registration number **OR** cite DOI.	Not Applicable	
Report the **clinical trial registration number** (at ClinicalTrials.gov or equivalent), where applicable.	Not Applicable	

Laboratory protocol	Information included in the manuscript?	In which section is the information available? (Reagents and Tools Table, Materials and Methods, Figures, Data Availability Section)
Provide DOI OR other citation details if **external detailed step-by-step protocols** are available.	Not Applicable	

Experimental study design and statistics	Information included in the manuscript?	In which section is the information available? (Reagents and Tools Table, Materials and Methods, Figures, Data Availability Section)
Include a statement about **sample size** estimate even if no statistical methods were used.	Yes	Materials and Methods（"Next-generation sequencing analysis""Statistics in figure legends""Metabolomics analysis")Sample size rationale：No statistical method was used to predetermine sample size; sample sizes
Were any steps taken to minimize the effects of subjective bias when allocating animals/samples to treatment (e.g. **randomization procedure**)? If yes, have they been described?	Yes	No randomization was performed. Allocation of samples or animals into groups was determined by predefined characteristics such as genotype, age, or treatment conditions, which inherently dictated group assignment and made randomization not applicable.
Include a statement about **blinding** even if no blinding was done.	Yes	Investigators were not blinded to group allocation during experiments or outcome assessment, as the experimental design (e.g., defined genotypes and treatment groups) made blinding impractical. However, data
Describe **inclusion/exclusion criteria** if samples or animals were excluded from the analysis. Were the criteria pre-established?<br><br>If sample or data points were omitted from analysis, report if this was due to attrition or intentional exclusion and provide justification.	Yes	No pre-established inclusion or exclusion criteria were used. All samples and animals that met basic experimental quality standards (e.g., viability, successful processing, adequate signal quality for imaging or omics datasets) were included. Any omitted data points resulted solely from technical failure (e.g., poor sequencing quality, LC–MS instrument dropout) and were not due to intentional exclusion.
For every figure, are **statistical tests** justified as appropriate? Do the data meet the assumptions of the tests (e.g., normal distribution)? Describe any methods used to assess it. Is there an estimate of variation within each group of data? Is the variance similar between the groups that are being statistically compared?	Yes	Statistical tests were chosen according to data type and distribution and are described in the figure legends. Data are presented as mean ± SD unless otherwise stated. Normality and variance were assessed using standard QC metrics within the software packages used (e.g., DESeq2 for RNA-seq, MetaboAnalyst for metabolomics). Comparisons between two groups used two-tailed Student's t-tests; multiple-group comparisons used

Sample definition and in-laboratory replication	Information included in the manuscript?	In which section is the information available? (Reagents and Tools Table, Materials and Methods, Figures, Data Availability Section)
In the figure legends: state number of times the experiment was **replicated** in laboratory.	Yes	All experiments include at least three biological replicates. Exact replicate numbers are provided in each figure legend.
In the figure legends: define whether data describe **technical or biological replicates**.	Yes	

Ethics

Ethics	Information included in the manuscript?	In which section is the information available? (Reagents and Tools Table, Materials and Methods, Figures, Data Availability Section)
Studies involving **human participants**: State details of **authority granting ethics approval** (IRB or equivalent committee(s), provide reference number for approval.	Yes	Materials and Methods
Studies involving **human participants**: Include a statement confirming that **informed consent** was obtained from all subjects and that the experiments conformed to the principles set out in the WMA Declaration of Helsinki and the Department of Health and Human Services Belmont Report.	Not Applicable	Not applicable. No new human samples were collected; only pre-existing iPSC lines were used under IRB approval(2023-IRB-0186-P-01)
Studies involving **human participants: For publication of patient photos**, include a statement confirming that consent to publish was obtained.	Not Applicable	
Studies involving experimental **animals**: State details of **authority granting ethics approval** (IRB or equivalent committee(s), provide reference number for approval. Include a statement of compliance with ethical regulations.	Yes	Materials and Methods. All mice were handled according to Zhejiang University Animal Research Institute Committee guidelines (protocol number: ZJU20250819).
Studies involving **specimen and field samples:** State if relevant **permits** obtained, provide details of authority approving study; if none were required, explain why.	Not Applicable	

Dual Use Research of Concern (DURC)	Information included in the manuscript?	In which section is the information available? (Reagents and Tools Table, Materials and Methods, Figures, Data Availability Section)
Could your study fall under dual use research restrictions? Please check biosecurity documents and list of **select agents and toxins** (CDC): https://www.selectagents.gov/sat/list.htm	Not Applicable	
If you used a select agent, is the security level of the lab appropriate and reported in the manuscript?	Not Applicable	
If a study is subject to dual use research of concern regulations, is the name of the **authority granting approval and reference number** for the regulatory approval provided in the manuscript?	Not Applicable	

Reporting

The MDAR framework recommends adoption of discipline-specific guidelines, established and endorsed through community initiatives. Journals have their own policy about requiring specific guidelines and recommendations to complement MDAR.

Adherence to community standards	Information included in the manuscript?	In which section is the information available? (Reagents and Tools Table, Materials and Methods, Figures, Data Availability Section)
State if relevant guidelines or checklists (e.g., **ICMJE, MIBBI, ARRIVE, PRISMA**) have been followed or provided.	Yes	We adhered to the relevant community standards for the types of experiments performed in this study. Animal experiments followed the ARRIVE guidelines where applicable. Metabolomics data acquisition and reporting followed MIBBI and Metabolomics Standards Initiative (MSI)
For **tumor marker prognostic studies,** we recommend that you follow the **REMARK** reporting guidelines (see link list at top right). See author guidelines, under 'Reporting Guidelines'. Please confirm you have followed these guidelines.	Not Applicable	
For **phase II and III randomized controlled trials**, please refer to the **CONSORT** flow diagram (see link list at top right) and submit the CONSORT checklist (see link list at top right) with your submission. See author guidelines, under 'Reporting Guidelines'. Please confirm you have submitted this list.	Not Applicable	

Data Availability

Data availability	Information included in the manuscript?	In which section is the information available? (Reagents and Tools Table, Materials and Methods, Figures, Data Availability Section)
Have **primary datasets** been deposited according to the journal's guidelines (see 'Data Deposition' section) and the respective accession numbers provided in the Data Availability Section?	Yes	• RNA-seq → GEO GSE268560<br>• LC-MS/MS proteomics → OMIX011107, OMIX011121,OMIX016492
Were **human clinical and genomic datasets** deposited in a public access-controlled repository in accordance to ethical obligations to the patients and to the applicable consent agreement?	Not Applicable	
Are **computational models** that are central and integral to a study available without restrictions in a machine-readable form? Were the relevant accession numbers or links provided?	Not Applicable	
If publicly available data were reused, provide the respective **data citations in the reference list**.	Not Applicable	
